# Supplementary material for: Deoxygenative photochemical alkylation of secondary amides enables a streamlined synthesis of substituted amines
Source: Nat Commun. 2025 Jan 22;16:948. doi: 10.1038/s41467-025-56234-w (PMC11754598; doi:10.1038/s41467-025-56234-w)
Supplement: Supplementary file 1 — Supplementary Information [file 41467_2025_56234_MOESM1_ESM.pdf]

Supplementary Information

Deoxygenative Photochemical Alkylation of Secondary Amides Enables a Streamlined Synthesis of Substituted Amines

*Antonio Pulcinella,[1,†] Stefano Bonciolini,[1,†] Robin Stuhr,[1,2] Damiano Diprima,[1] Minh Thao Tran,[3] Magnus Johansson,[4] Axel Jacobi von Wangelin,[2] and Timothy Noël[1,]**

[1] Flow Chemistry Group, Van 't Hoff Institute for Molecular Sciences (HIMS), University of Amsterdam, Science Park 904, 1098 XH Amsterdam, The Netherlands.

[2] Department of Chemistry, University of Hamburg, Martin-Luther-King Platz 6, 20146 Hamburg, Germany.

[3] Janssen Pharmaceutica NV, Beerse 2340, Belgium.

[4] Medicinal Chemistry, Research and Early Development, Cardiovascular, Renal and Metabolism (CVRM), BioPharmaceuticals R&D, AstraZeneca, Gothenburg, Sweden.

[†] These authors contributed equally to this work.

* *Email: t.noel@uva.nl*

Table of Contents

1. General information 3
2. Chart of starting materials used in the scope 4
3. Synthesis of Starting Materials 6
   3.1 General procedure (**GP1**) for the preparation of secondary amides (**1a-1q**) from benzoyl chloride 6
   3.2 General procedure (**GP2**) for the preparation of secondary amides (**2a-2ae**) from carboxylic acids 6
   3.3 General procedure (**GP3**) for the preparation of amides used for the cyclization strategy (**3a-3h**) 6
   3.4 General Procedure (**GP4**) for the preparation of alkyl iodides 7
   3.5 Synthesis of amides via multistep sequences 7
4. Reaction setup 11
5. Reaction Optimization 13
   5.1 Optimization of the deoxygenative semi-reduction of *sec*-amides to imines 13
   5.2 Preliminary attempts of the deoxygenative alkylation of *sec*-amides 17
   5.3 Optimization of the photochemical alkylation 18
6. General procedure 5 (**GP5**): Deoxygenative alkylation of amides (activation at 0 ˚C) 23
7. General procedure 6 (**GP6**): Deoxygenative alkylation of amides (activation at -78 ˚C) 23
8. General procedure 7 (**GP7**): Deoxygenative alkylation of amides (activation at 0 ˚C) and subsequent cyclization: *N*-substituted piperidines synthesis. 24
9. Mechanistic investigation 25
   9.1 UV-Vis Characterization 25
   9.2 TEMPO radical trapping 30
   9.3 Radical Clock Experiment 31
10. Scale-up procedure for compounds **5**, **79** 33
   10.1 Re-optimization of the reaction conditions in batch for compound **5** 33
   10.2 Optimization and scale-up in continuous flow of compound **5**, **79** 38
      10.2.1 General procedure (**GP7**) for the deoxygenative alkylation of amides in continuous flow 38
      10.2.2 Scale-up (10 mmol) of compound **5** in flow 42
      10.2.3 Scale-up (10 mmol) of compound **79** in flow (cyclization) 43
11. Characterization data of synthesized compound 45
   11.1 Characterization of newly synthesized secondary amides 45
   11.2 Characterization of compounds **5-42** (*sec*-amides varying amine or carboxylic acid) 51
   11.3 Characterization of compounds **43-54** (alkyl iodides scope) 61
   11.4 Characterization of compounds **55-63** (drug-like sec-amides) 65
   11.5 Characterization of compounds **64-71** (cyclization strategy) 68
12. Limitation of the scope 71
13. NMR spectra secondary amides 72
14. NMR spectra of products (**5-63**) 108
15. NMR spectra of products (**64-71**, **79**) 174
16. References 183

1. General information

All reagents and solvents were used as received without further purification, unless stated otherwise. Reagents and solvents were bought from Sigma Aldrich, TCI, Fluorochem and Fisher Scientific and, if applicable, kept under nitrogen atmosphere. Technical solvents were bought from VWR International and Biosolve, and were used as received. Disposable syringes were purchased from Laboratory Glass Specialist. Product isolation was performed manually, using silica (P60, SILICYCLE) or automatically, using Biotage® Isolation Four, with Biotage® SNAP KP-Sil 4 or 10 g flash chromatography cartridges. TLC analysis was performed using Silica on aluminum foils TLC plates (F254, Supelco Sigma-Aldrich™) with visualization under ultraviolet light (254 nm and 365 nm) or appropriate TLC staining (cerium ammonium molybdate or potassium permanganate). ^{1}H (400 MHz), ^{13}C (101 MHz), ^{19}F NMR (376 MHz), ^{11}B NMR (128 MHz) spectra were recorded unless stated otherwise at ambient temperature using a Bruker AV400 III, Bruker AV400 or a Bruker AV300. ^{1}H NMR spectra are reported in parts per million (ppm) downfield relative to $CDCl_3$ (7.26 ppm) and all ^{13}C NMR spectra are reported in ppm relative to $CDCl_3$ (77.16 ppm) unless stated otherwise. The following abbreviations have been adopted to describe the multiplicity: bs (broad singlet), s (singlet), d (doublet), t (triplet), q (quartet), p (pentet), h (hextet), hept (heptet), m (multiplet), dd (double doublet), td (triple doublet), tt (triplet of triplets). Coupling constants (*J*) are reported in hertz (Hz). NMR data were processed using the MestReNova 14.1.0 software package. Known products were characterized through comparison with the corresponding ^{1}H NMR and ^{13}C NMR from literature. High resolution mass spectra (HRMS) were collected on an AccuTOF LC, JMS-T100LP Mass spectrometer (JEOL, Japen. UV-Vis spectra were recorded with a double beam spectrophotometer Shimadzu UV2600 equipped with a deuterium lamp (190-350 nm), a halogen lamp (330-900 nm) and a photomultiplier (Hamamatsu R928). Iminium ion were monitored by reversed-phase (RP) UPLC-MS. Analytical RP-UPLC-MS was performed on a Waters: Acquity UPLC-DAD and SQD2 mass spectrometer using the following column: Waters BEH 1.7 µm, 2.1 × 50 mm at a flow rate of 0.8 mL/min at 55 °C. A linear gradient of mobile phase from 100% A to 5% A in 1.3 min, hold 0.7min (A: 10 mM CH_3COONH_4 in 95% H_2O + 5% CH_3CN and B = CH_3CN) with detection from 210 - 350 nm.

The names of all products were generated using the PerkinElmer ChemBioDraw Ultra v.12.0.2 software package.

For the photochemical batch experiments and scale-up (1 to 5 mmol), a 3D-printed (PLA) reactor internally coated with aluminum foil and equipped with a specific 3D-printed (PLA) lid serving as vials holder and lamp holder was used (see section 4 for details).

2. Chart of starting materials used in the scope

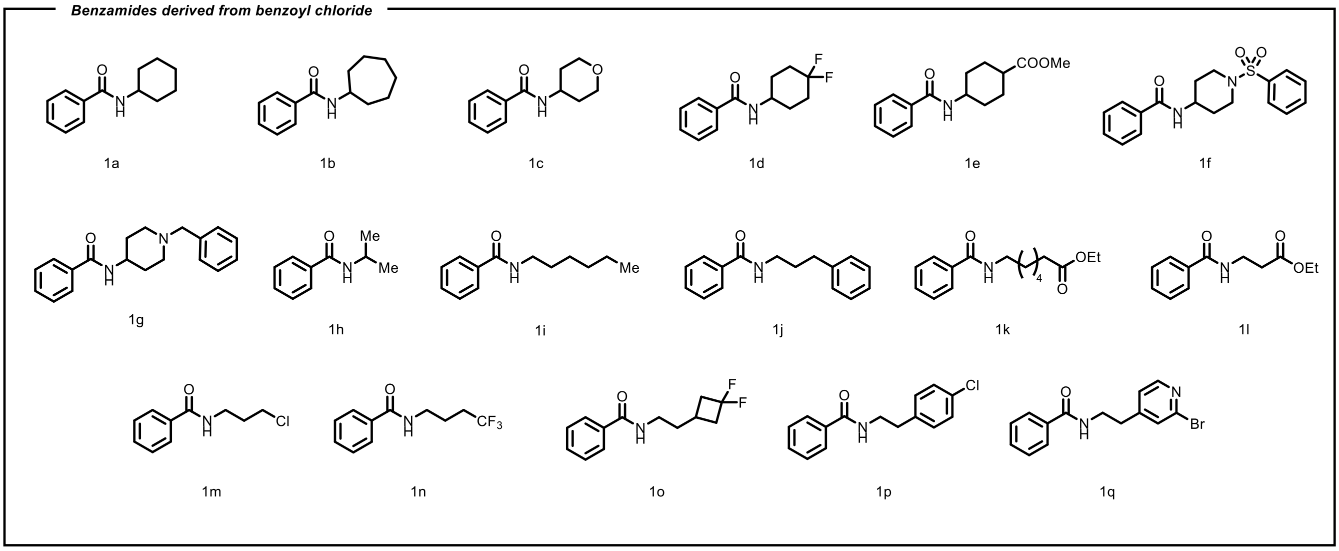

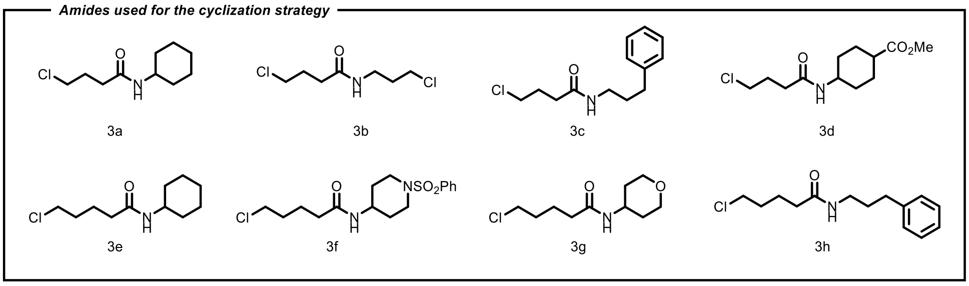

Amides derived from activation of carboxylic acids

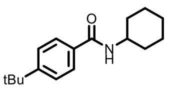
2a

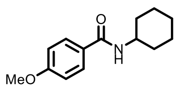
2b

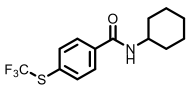
2c

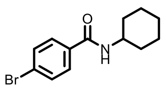
2d

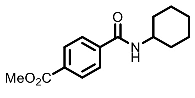
2e

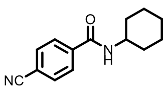
2f

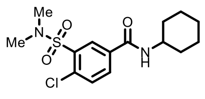
2g

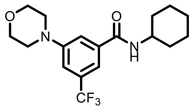
2h

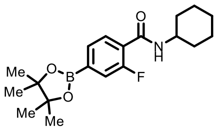
2i

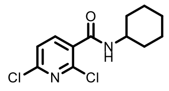
2j

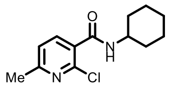
2k

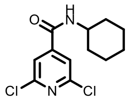
2l

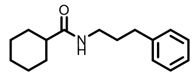
2m

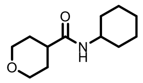
2n

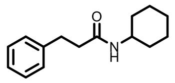
2o

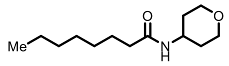
2p

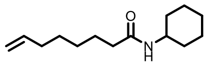
2q

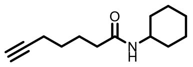
2r

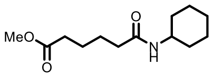
2s

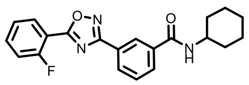
2t

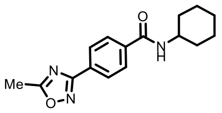
2u

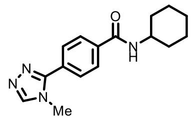
2v

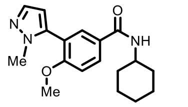
2z

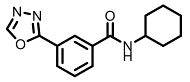
2aa

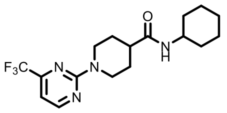
2ab

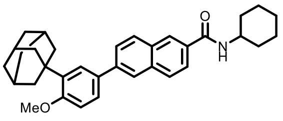
2ac

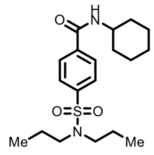
2ad

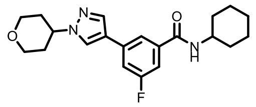
2ae

Alkyl Iodides

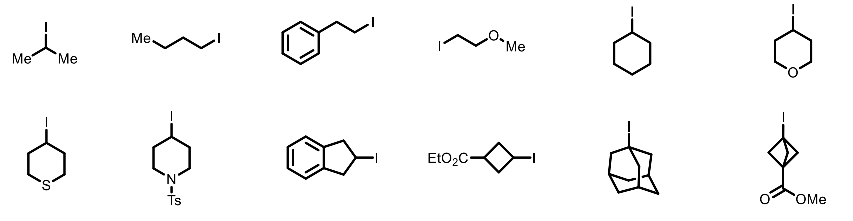

3. Synthesis of Starting Materials

3.1 General procedure (GP1) for the preparation of secondary amides (1a-1q) from benzoyl chloride

To an oven-dried round-bottom flask equipped with a septum and a stirring-bar was added the amine (1.1 equiv.) and dry CH_2Cl_2 (0.2 M), under N_2 atmosphere. The solution was cooled to 0 °C and triethylamine (1.5 equiv.) was added. Then, benzoyl chloride (1.5-30 mmol, 1.0 equiv.) was slowly added dropwise via a syringe and the reaction was then warmed up to room temperature and stirred overnight. The reaction mixture was quenched by addition of a saturated aqueous Na_2CO_3 solution until pH~10-11 and then diluted with CH_2Cl_2. The mixture was transferred to a separation funnel and extracted with CH_2Cl_2 (2x). The combined organic layers were washed with 1 M HCl (2x) and aq. NaCl sat. (1x). The final organic layers were dried over Na_2SO_4, filtered and the solvent was removed under reduced pressure to afford the desired secondary amide. For all newly reported secondary benzamides see section 11.1 for characterization data.

3.2 General procedure (GP2) for the preparation of secondary amides (2a-2ae) from carboxylic acids

To a stirring solution of carboxylic acid (1.5-30 mmol, 1.0 equiv.) in dry CH_2Cl_2 (0.2 M), oxalyl chloride (1.2 equiv.) and few drops of dry DMF were added dropwise at 0 °C under N_2 atmosphere. Then, the reaction mixture was stirred for 90 minutes at room temperature. Upon completion, the solvent was removed under reduced pressure to afford the crude carbonyl chloride. The residue was dissolved in dry CH_2Cl_2 (0.2 M), and the cyclohexylamine (1.1 equiv., 0.865 g/mL) was added, followed by triethylamine (1.5 equiv.) at 0 °C under N_2 atmosphere. Then, the reaction mixture was stirred overnight at room temperature. The reaction mixture was quenched by addition of a saturated aqueous Na_2CO_3 solution until pH~10-11 and then diluted with CH_2Cl_2. The mixture was transferred to a separation funnel and extracted with CH_2Cl_2 (2x). The combined organic layers were washed with 1 M HCl (2x), aq. NaCl sat. (1x). The final organic layers were dried over Na_2SO_4, filtered and the solvent was removed under reduced pressure to afford the desired secondary amide. For all newly reported secondary benzamides see section 11.1 for characterization data.

N.B. For substrates presenting basic functional group (like pyridines) the 1M HCl wash was omitted. Instead, the final crude was washed with a 20:1 n-pentane:ethyl acetate mixture.

3.3 General procedure (GP3) for the preparation of amides used for the cyclization strategy (3a-3h)

To an oven-dried round-bottom flask equipped with a septum and a stirring-bar was added the amine (1.1 equiv.) and dry CH_2Cl_2 (0.2 M) under N_2 atmosphere. The solution was cooled to 0 °C and triethylamine (1.5 equiv.) was added. Then, the corresponding chloro-alkylcarbonyl chloride (1.0 equiv.) was slowly added via a syringe dropwise and the reaction was then warmed up to room temperature and stirred overnight. The reaction was quenched by addition of a saturated aqueous Na_2CO_3 solution until pH~10-11 and then diluted with CH_2Cl_2. The mixture was transferred to a separation funnel and extracted with CH_2Cl_2 (2x). The organic layers were combined and washed with 1 M HCl (2x), aq. NaCl sat. (1x). The final organic layers were dried over Na_2SO_4, filtered and the solvent was removed under reduced pressure to afford the desired secondary amide without any further purification. For all newly reported secondary chloro-*N*-(alkyl) alkylamides see section 11.1 for characterization data.

3.4 General Procedure (GP4) for the preparation of alkyl iodides

The alkyl iodides used in the scope were synthesized adapting a procedure reported in the literature.[1]
To an oven-dried round-bottom flask equipped with a septum and a stirring-bar was added the alcohol (1.0 equiv.), PPh_3 (1.2 equiv.) and imidazole (1.2 equiv.) under N_2 atmosphere. CH_2Cl_2 (0.1 M) was added, and the reaction was cooled to 0 °C. I_2 (1.2 equiv.) was added portion-wise. The reaction was stirred overnight at room temperature and then diluted with a saturated aqueous $Na_2S_2O_3$ solution. The layers were separated and the aqueous layer was extracted with CH_2Cl_2 (x3). The combined organic layers were dried over Na_2SO_4, filtered and the solvent was removed under reduced pressure. Purification by flash column chromatography on silica gel affords the desired alkyl iodide.

3.5 Synthesis of amides via multistep sequences

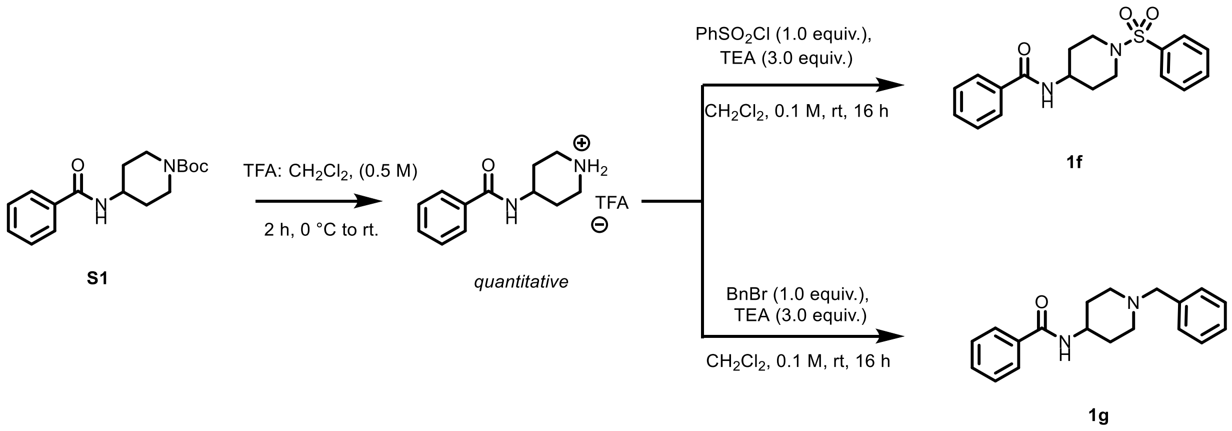

Amide **S1** was synthesized according to GP1.

Spectroscopic data are in accordance with those reported in literature.[2]
^{1}H NMR (300 MHz, $CDCl_3$) δ 7.80 – 7.71 (m, 2H), 7.54 – 7.37 (m, 3H), 6.07 (d, J = 7.9 Hz, 1H), 4.17 – 3.91 (m, 3H), 2.90 (t, J = 12.7 Hz, 2H), 2.08 – 1.93 (m, 2H), 1.52 – 1.26 (m, 11H).

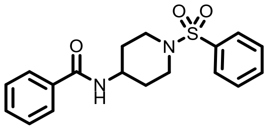

***N*-(1-(phenylsulfonyl)piperidin-4-yl)benzamide (1f)**
To a cooled solution (0 °C) of 2,2,2-trifluoroacetaldehyde, 4-benzamidopiperidin-1-ium salt (1.52 g, 5.0 mmol, 1.0 equiv.) in dichloromethane (50 mL) benzenesulfonyl chloride (641 µL, 5.0 mmol, 1.0 equiv.) was added followed by dropwise addition of triethylamine (2.10 mL, 15.0 mmol, 3.0 equiv.). Then, the homogeneous solution was removed from the ice bath and stirred at room temperature for 16 h. The reaction mixture was washed with 1 M HCl (3 x) followed by saturated aqueous $NaHCO_3$ (2 x) and finally with brine. The combined organic layers were dried over Na_2SO_4, filtered, and concentrated under reduced pressure to obtain the product as a white solid (775 mg, 2.25 mmol, 45%)

^{1}H NMR (400 MHz, $CDCl_3$) δ 7.77 (dd, J = 7.0, 1.6 Hz, 2H), 7.75 – 7.71 (m, 2H), 7.67 – 7.61 (m, 1H), 7.56 (dd, J = 8.2, 6.6 Hz, 2H), 7.51 – 7.45 (m, 1H), 7.41 (dd, J = 8.2, 6.6 Hz, 2H), 6.11 (d, J = 8.0 Hz, 1H), 3.99 – 3.87 (m, 1H), 3.83 (d, J = 12.0 Hz, 2H), 2.45 (td, J = 12.0, 2.5 Hz, 2H), 2.09 (d, J = 12.7 Hz, 2H), 1.76 – 1.56 (m, 2H).
^{13}C NMR (101 MHz, $CDCl_3$) δ 167.1, 136.0, 134.3, 133.1, 131.8, 129.3, 128.7, 127.7, 127.0, 46.5, 45.7, 31.7.
HRMS (ESI+) (m/z): $[M+H]^+$ calcd. for $C_{18}H_{20}N_2O_3S$, 345.1273; found: 345.1272.

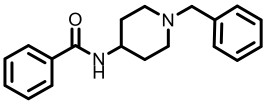

N-(1-benzylpiperidin-4-yl)benzamide **(1g)**
To a cooled solution (0 °C) of 2,2,2-trifluoroacetaldehyde, 4-benzamidopiperidin-1-ium salt (1.52 g, 5.0 mmol, 1.0 equiv.) in dichloromethane (50 mL) benzyl bromide (594 µL, 5.0 mmol, 1.0 equiv.) was added followed by dropwise addition of triethylamine (2.10 mL, 15.0 mmol, 3.0 equiv.). Then, the homogeneous solution was removed from the ice bath and stirred at room temperature for 16 h. The reaction mixture was washed with 1 M HCl (3 x) followed by saturated aqueous $NaHCO_3$ (2 x) and finally with brine. The combined organic layers were dried over Na_2SO_4, filtered, and concentrated under reduced pressure to obtain the product as a white solid (1.21 g, 4.1 mmol, 82%)

^{1}H NMR (400 MHz, $CDCl_3$) δ 7.79 – 7.73 (m, 2H), 7.50 – 7.27 (m, 8H), 6.48 (d, *J* = 8.0 Hz, 1H), 4.11 – 4.03 (m, 1H), 3.72 (s, 2H), 3.05 (d, *J* = 12.2 Hz, 2H), 2.40 (td, *J* = 11.9, 2.7 Hz, 2H), 2.05 (dd, *J* = 13.5, 3.9 Hz, 2H), 1.86 (qd, *J* = 11.6, 3.8 Hz, 2H).
^{13}C NMR (101 MHz, $CDCl_3$) δ 167.1, 135.0, 134.5, 131.6, 130.0, 128.7, 128.6, 128.2, 127.1, 62.4, 52.1, 46.4, 31.2.
HRMS (ESI+) (m/z): $[M+H]^+$ calcd. for $C_{19}H_{22}N_2O$, 295.1814; found: 295.1810.

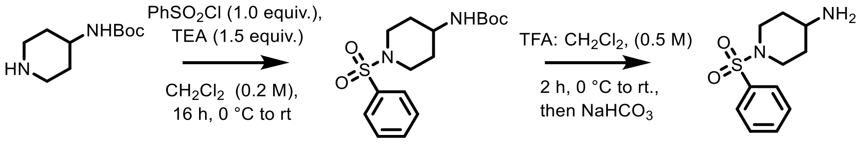

S2, 82%   **S3**. 90%

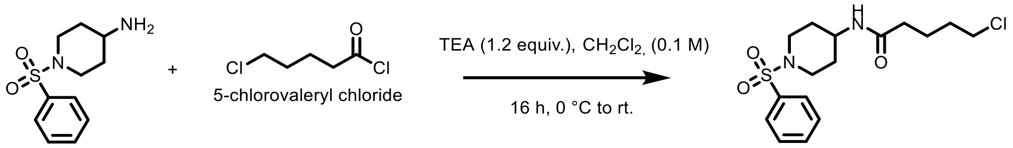

S3   **3f**, 68%

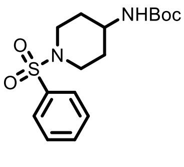

S2 was synthesized adapting a procedure reported in the literature.[3]
Sulfonyl chloride (641 µL, 5.00 mmol) was added dropwise at to a stirred solution of tert-butyl piperidin-4-ylcarbamate (2.56 g, 19.8 mmol) and triethylamine (1.0 g, 5.0 mmol) in 25 mL of dry CH_2Cl_2 (0.2 M) at 0 °C. The resulting mixture was stirred at room temperature for 16 h. After, water (10 mL) was added and the aqueous phase was extracted with CH_2Cl_2. The organic layer was washed with Na_2CO_3 and 1M HCl. The combined organic layers were dried over Na_2SO_4, filtered and the solvent was removed under reduced pressure to afford the desired product as a white solid. (1.40 g, 4.10 mmol, 82%).

Spectroscopic data for compound **S2** are in accordance with those reported in literature.[3]
^{1}H NMR (300 MHz, $CDCl_3$) δ 7.80 – 7.75 (m, 2H), 7.63 – 7.47 (m, 3H), 4.39 (s, 1H), 3.70 (d, *J* = 11.8 Hz, 2H), 3.38 (s, 1H), 2.44 (t, *J* = 11.7 Hz, 2H), 2.08 – 1.91 (m, 2H), 1.58 – 1.43 (m, 2H), 1.41 (s, 9H).

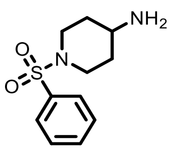

To a stirring solution of **S3** (1.36 g, 4.00 mmol) in dichloromethane (4 mL, 1.0 M), trifluoroacetic acid (4 mL, final 0.05 M) was added slowly at 0 ˚C. Then, the solution was stirred at room temperature for 2h (caution evolution of gas). Then, the residual trifluoroacetic acid was removed under reduced pressure and the crude mixture was neutralized using a saturated $NaHCO_3$ solution. The mixture was transferred to a separation funnel. The aqueous layer was extracted with CH_2Cl_2 (3x). Then, the organic layers were combined, dried over Na_2SO_4, filtered and the solvent was removed under reduced pressure to afford the desired product as colorless oil (865 mg, 2.60 mmol, 90%).

Spectroscopic data for compound **S3** are in accordance with those reported in literature.[3]
^{1}H NMR (300 MHz, $CDCl_3$) δ 7.81 – 7.69 (m, 2H), 7.61 – 7.44 (m, 3H), 3.73 – 3.54 (m, 2H), 2.69 – 2.57 (m, 1H), 2.42 (td, *J* = 11.6, 2.8 Hz, 2H), 1.89 – 1.77 (m, 2H), 1.51 – 1.34 (m, 2H), 1.25 (bs, 2H).

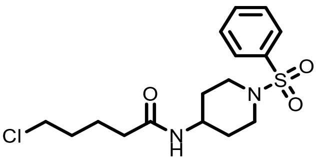

5-chloro-N-(1-(phenylsulfonyl)piperidin-4-yl)pentanamide **(3f).** Prepared according to GP3 from **S3** (264 mg, 2.75 mmol, 1.1 equiv.) and 5-chloropentanoyl chloride (320 µL, 2.5 mmol, 1.0 equiv.) to afford the product as a white solid (610 mg, 68% yield).

^{1}H NMR (400 MHz, $CDCl_3$) δ 7.74 (d, *J* = 7.7 Hz, 2H), 7.65 – 7.60 (m, 1H), 7.58 – 7.52 (m, 2H), 5.74 (d, *J* = 8.1 Hz, 1H), 3.84 – 3.65 (m, 3H), 3.54 – 3.46 (m, 2H), 2.37 (dd, *J* = 13.0, 10.3 Hz, 2H), 2.21 – 2.10 (m, 2H), 2.03 – 1.93 (m, 2H), 1.79 – 1.71 (m, 4H), 1.62 – 1.49 (m, 2H).
^{13}C NMR (101 MHz, $CDCl_3$) δ 172.0, 135.8, 133.2, 129.3, 127.7, 45.8, 45.7, 44.7, 35.8, 32.0, 31.6, 23.0.
HRMS (ESI+) (m/z): $[M+H]^+$ calcd. for $C_{16}H_{23}ClN_2O_3S$, 359.1196; found: 359.1199.

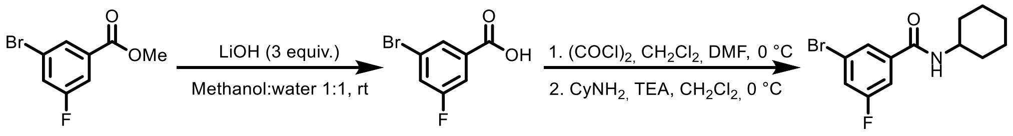

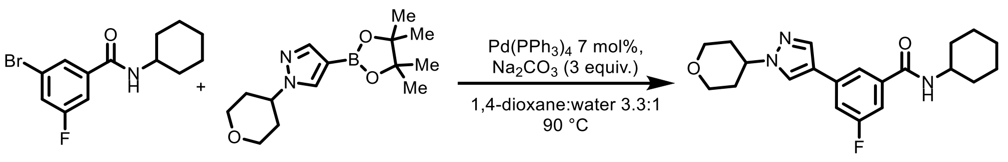

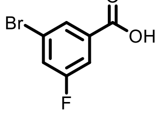

To a stirring solution of methyl 3-bromo-5-fluorobenzoate (4.66 g, 20.0 mmol, 1.0 equiv.) in 100 mL methanol : water (1:1, 0.20 M), LiOH (1.44 g, 60.0 mmol, 3.0 equiv.) was added. The corresponding mixture was stirred at rt for 3 hours. After, HCl 2 M was added until pH~2-3 and the mixture was transferred to a separation funnel. The aqueous layer was extracted with CH_2Cl_2 (3x). Then, the organic layers were combined, dried over Na_2SO_4, filtered and the solvent was removed under reduced pressure to afford the desired product as a white solid (4.10 g, 18.7 mmol, 94%).

Spectroscopic data for the product are in accordance with those reported in literature.[4]
^{1}H NMR (400 MHz, $CDCl_3$) δ 8.06 (t, *J* = 1.6 Hz, 1H), 7.74 (ddd, *J* = 8.6, 2.5, 1.4 Hz, 1H), 7.51 (dt, *J* = 8.0, 2.1 Hz, 1H).
^{19}F NMR (376 MHz, $CDCl_3$) δ -109.3.

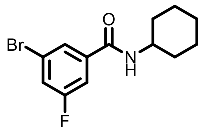

3-bromo-N-cyclohexyl-5-fluorobenzamide (**S4**) Prepared according to **GP2** from 3-bromo-5-fluorobenzoic acid (2.19 g, 10.0 mmol, 1.0 equiv.). Isolated as a white solid (2.75 g, 92% yield).

^{1}H NMR (400 MHz, $CDCl_3$) δ 7.63 (t, *J* = 1.7 Hz, 1H), 7.41 (ddd, *J* = 8.8, 2.4, 1.4 Hz, 1H), 7.35 (ddd, *J* = 7.8, 2.4, 1.7 Hz, 1H), 5.99 (d, *J* = 7.4 Hz, 1H), 3.93 (tdt, *J* = 10.7, 8.0, 4.0 Hz, 1H), 2.07 – 1.92 (m, 2H), 1.81 – 1.71 (m, 2H), 1.70 – 1.61 (m, 1H), 1.49 – 1.33 (m, 2H), 1.30 – 1.16 (m, 3H).
^{13}C NMR (101 MHz, $CDCl_3$) δ 164.1 (d, *J* = 2.5 Hz), 162.6 (d, *J* = 252.6 Hz), 138.7 (d, *J* = 7.1 Hz), 125.9 (d, *J* = 3.3 Hz), 123.0 (d, *J* = 9.2 Hz), 121.9 (d, *J* = 24.5 Hz), 113.5 (d, *J* = 22.7 Hz), 49.3, 33.2, 25.6, 25.0.
^{19}F NMR (377 MHz, $CDCl_3$) δ -109.3.
HRMS (ESI+) (m/z): $[M+H]^+$ calcd. for $C_{13}H_{15}BrFNO$, 300.0399; found: 300.0410.

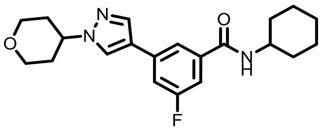

N-cyclohexyl-3-fluoro-5-(1-(tetrahydro-2H-pyran-4-yl)-1H-pyrazol-4-yl)benzamide (**2ae**) To a solution of 1-(tetrahydro-2H-pyran-4-yl)-4-(4,4,5,5-tetramethyl-1,3,2-dioxaborolan-2-yl)-1H-pyrazole (306 mg, 1.10 mmol, 1.1 equiv.), 3-bromo-N-cyclohexyl-5-fluorobenzamide **S4** (300 mg, 1.0 mmol, 1.0 equiv.) in 1,4-dioxane (5 mL) was added a freshly prepared solution of Na_2CO_3 (1.5 mL, 2.0 M, 3.0 equiv.) in water, followed by the addition of tetrakis(triphenylphosphine)palladium(0) (81 mg, 0.07 mmol, 0.07 equiv.). The corresponding mixture was degassed with nitrogen for 5 minutes and stirred and heated at 90 ˚C for 2 h. Then, the reaction mixture was diluted with water and extracted with ethyl acetate (3x). The combined organic layers were dried over Na_2SO_4, filtered and the solvent was removed under reduced pressure. The crude reaction mixture was purified by flash column chromatography on silica gel (gradient from Heptane:Ethyl Acetate 80:20 to 0:100) to afford the product as a white solid with an inseparable minor aromatic impurity (255 mg, 69% yield). It was used without any further purification for the deoxygenative alkylation step.

^{1}H NMR (400 MHz, $CDCl_3$) δ 7.80 (s, 1H), 7.73 (s, 1H), 7.66 – 7.62 (m, 1H), 7.30 – 7.19 (m, 2H), 6.01 (d, *J* = 8.1 Hz, 1H), 4.37 (tt, *J* = 10.3, 5.0 Hz, 1H), 4.17 – 4.08 (m, 2H), 3.97 (tq, *J* = 10.8, 4.0 Hz, 1H), 3.55 (td, *J* = 11.5, 3.2 Hz, 2H), 2.18 – 1.98 (m, 6H), 1.84 – 1.70 (m, 2H), 1.70 – 1.60 (m, 1H), 1.51 – 1.35 (m, 2H), 1.32 – 1.13 (m, 3H).
^{13}C NMR (101 MHz, $CDCl_3$) δ 165.6 (d, *J* = 2.8 Hz), 163.6 (d, *J* = 247.0 Hz), 136.7, 132.2 (d, *J* = 9.9 Hz), 128.6 (d, *J* = 12.0 Hz), 124.2, 121.3 (d, *J* = 2.4 Hz), 120.0 (d, *J* = 2.7 Hz), 114.9 (d, *J* = 22.4 Hz), 111.5 (d, *J* = 22.9 Hz), 66.9, 58.6, 49.1, 33.4, 33.3, 25.7, 25.1.
^{19}F NMR (377 MHz, $CDCl_3$) δ -112.2.
HRMS (ESI+) (m/z): $[M+H]^+$ calcd. for $C_{21}H_{26}FN_3O_2$, 372.2087; found: 372.2078.

4. Reaction setup

4-8 vials photoreactor (UFO reactor) used at the University of Amsterdam and Janssen Pharmaceutica
Four reactions were irradiating simultaneously using the photoreactor described below. A 40W Kessil PR160L-390 nm or 456 nm was used as LED lamp, while the temperature was maintained around 30-35 °C via a fan positioned under the reactor. The assembled set-up was placed behind UV-light shielding amber acrylic for all duration of the reaction.[5]

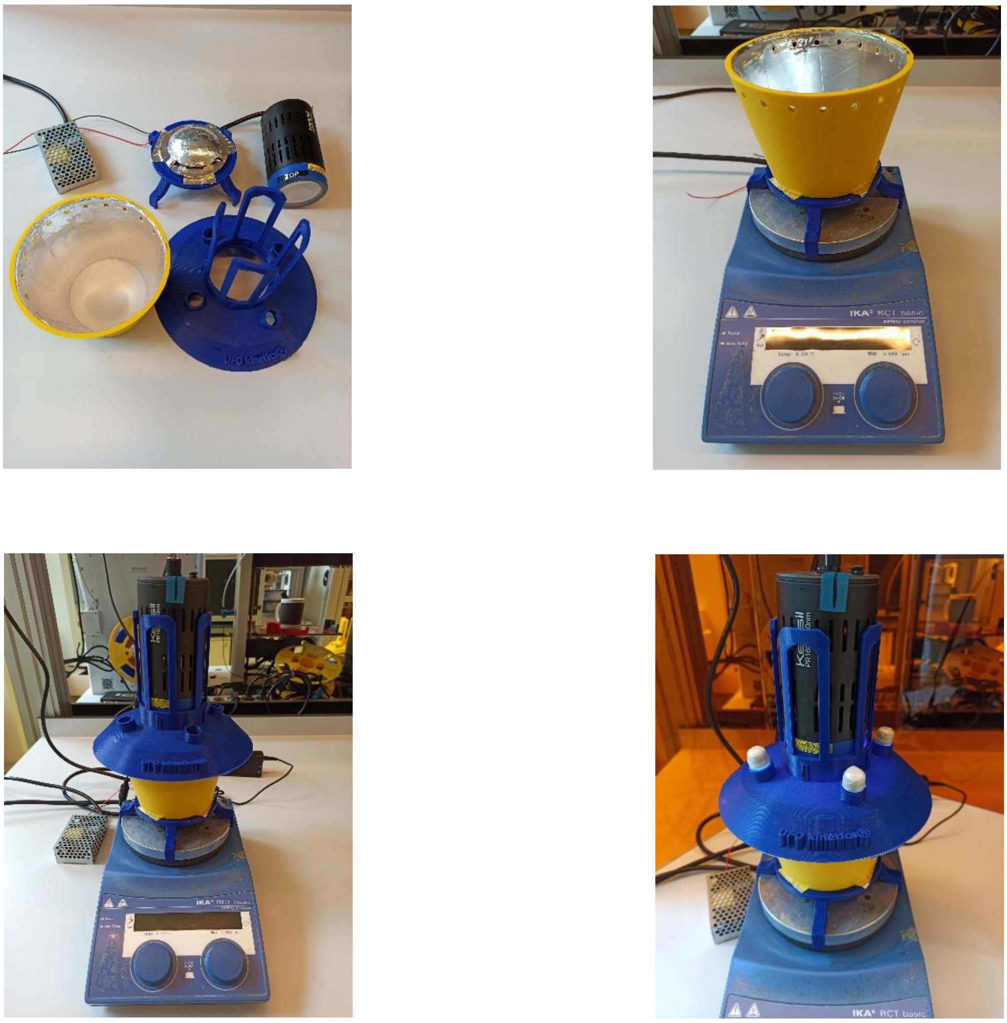

Supplementary Figure 1. Photoreactor used for the optimization and scope.

4 vials photoreactor (1 to 5 mmol scale) used at the University of Amsterdam

The oven-dried 30 mL vial equipped with a stirring bar was irradiated using the photoreactor described below. A 40W Kessil PR160L-390 nm was used as LED lamp, while the temperature was maintained around 30-35 °C via a fan positioned under the reactor. The assembled set-up was placed behind UV-light shielding amber acrylic for all duration of the reaction. 4 vials can be irradiated simultaneously to ensure a productivity of 4 to 20 mmol scale on a per-reactor basis.

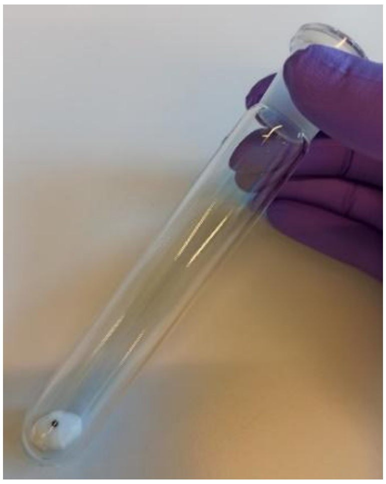

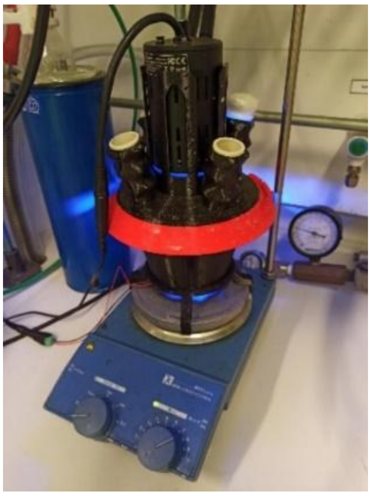

Supplementary Figure 2. Photoreactor set-up used for 1 to 5 mmol scale.

5. Reaction Optimization

5.1 Optimization of the deoxygenative semi-reduction of *sec*-amides to imines

Supplementary Table 1. Formation of imine int-A.

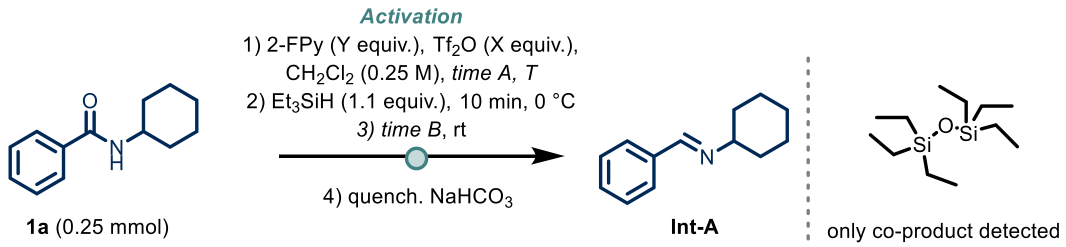

Tf_2O equiv.	2-FPy equiv.	T (°C)	time A	time B	Yield[a]
1.05	1.1	-78 to 0	10 min (-78) + 10 min (0)	5h	87%
1.1	1.2	0	20 min	5h	100%
1.1	1.2	0	20 min	2h	100%
1.1	1.2	0	20 min	1h	100%
1.1	1.2	0	20 min	16h	100%

[a]Determined via ^1H NMR using trichloroethylene as external standard.

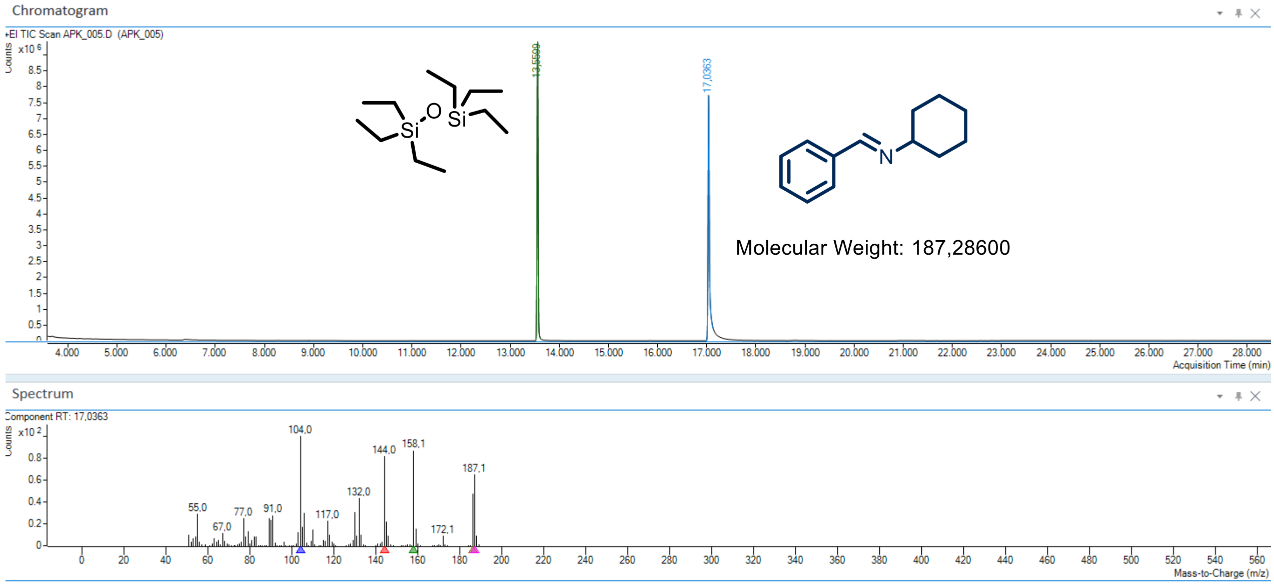

Supplementary Figure 3. Formation of imine int-A.

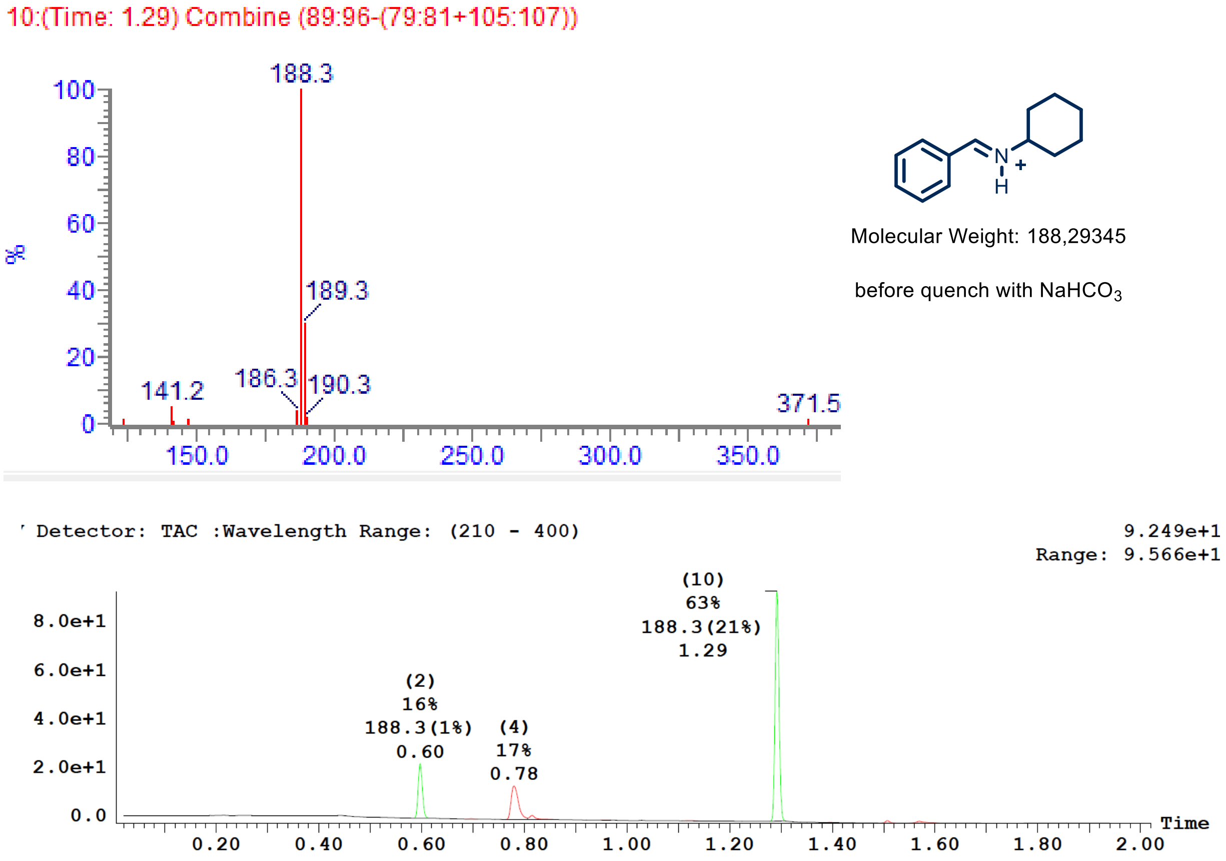

Supplementary Figure 4. Formation of iminium ion int-A.

Supplementary Table 2. Formation of imine int-B.

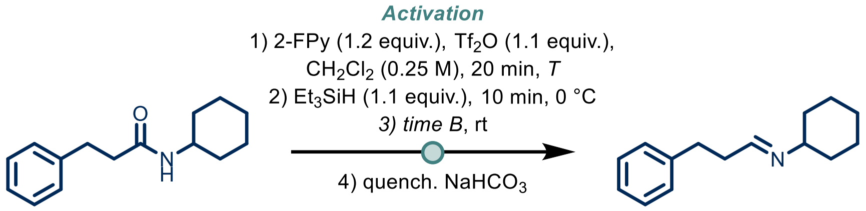

2o (0.25 mmol) **int-B**

T (°C)	time B	Yield[a]	Variation
0	2 h	no product formation	-
0	2 h	no product formation	no basic workup performed
10 min (-78) + 10 min (0)	5 h	product formed (decomposition)	-
10 min (-78) + 10 min (0)	5 h	100%	no basic workup performed

[a]Determined via ^1H NMR using trichloroethylene as external standard.

For secondary amides like **2o** derived from aliphatic carboxylic acids, we observed side-product formation when performing the activation step at 0 °C. On the contrary, when cooling the reaction mixture at -78 °C, clean formation of the desired iminium triflate was observed. For aliphatic amides it is suggested to not perform the basic work-up to avoid decomposition: direct ^1H NMR analysis of the iminium triflate can be performed after evaporation of the solvent or performing the reaction in CD_2Cl_2.

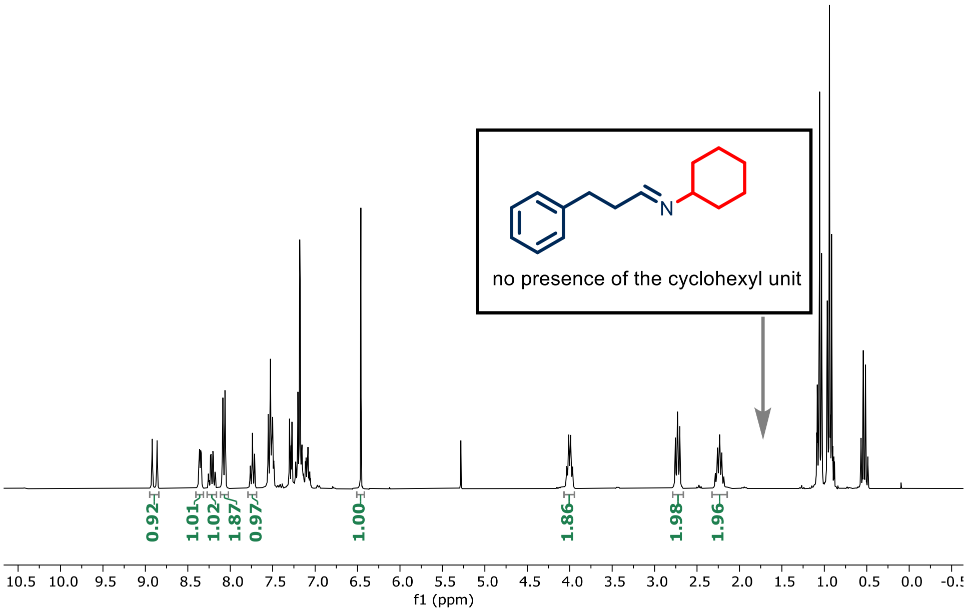

Supplementary Figure 5. Tf_2O addition at 0 °C: side-product formation derived from cyclohexyl unit extrusion.

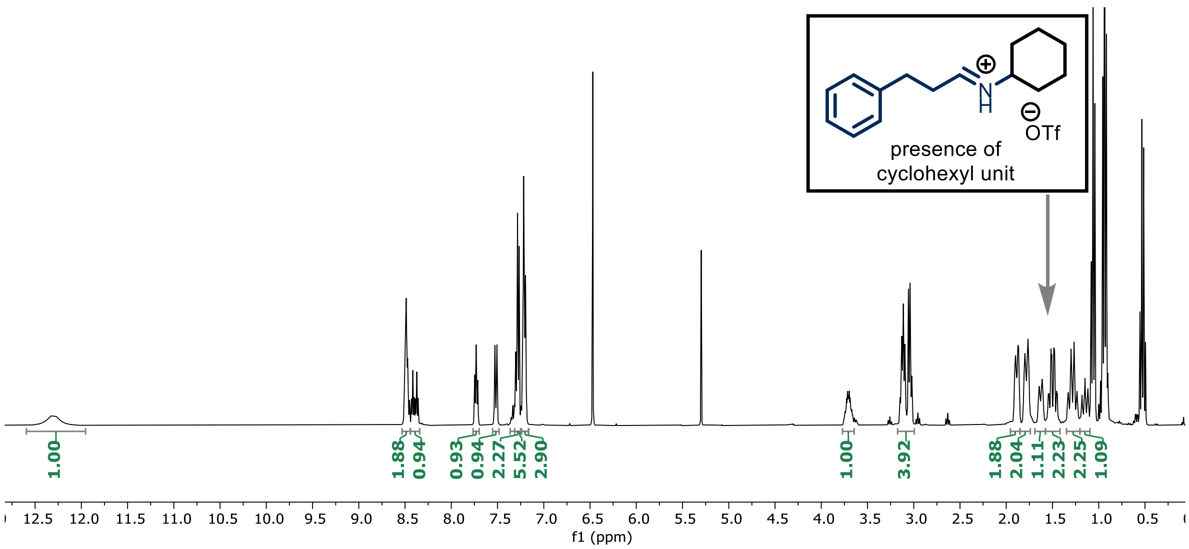

Supplementary Figure 6. Tf_2O addition at -78 °C: formation of the desired iminium ion.

5.2 Preliminary attempts of the deoxygenative alkylation of *sec*-amides

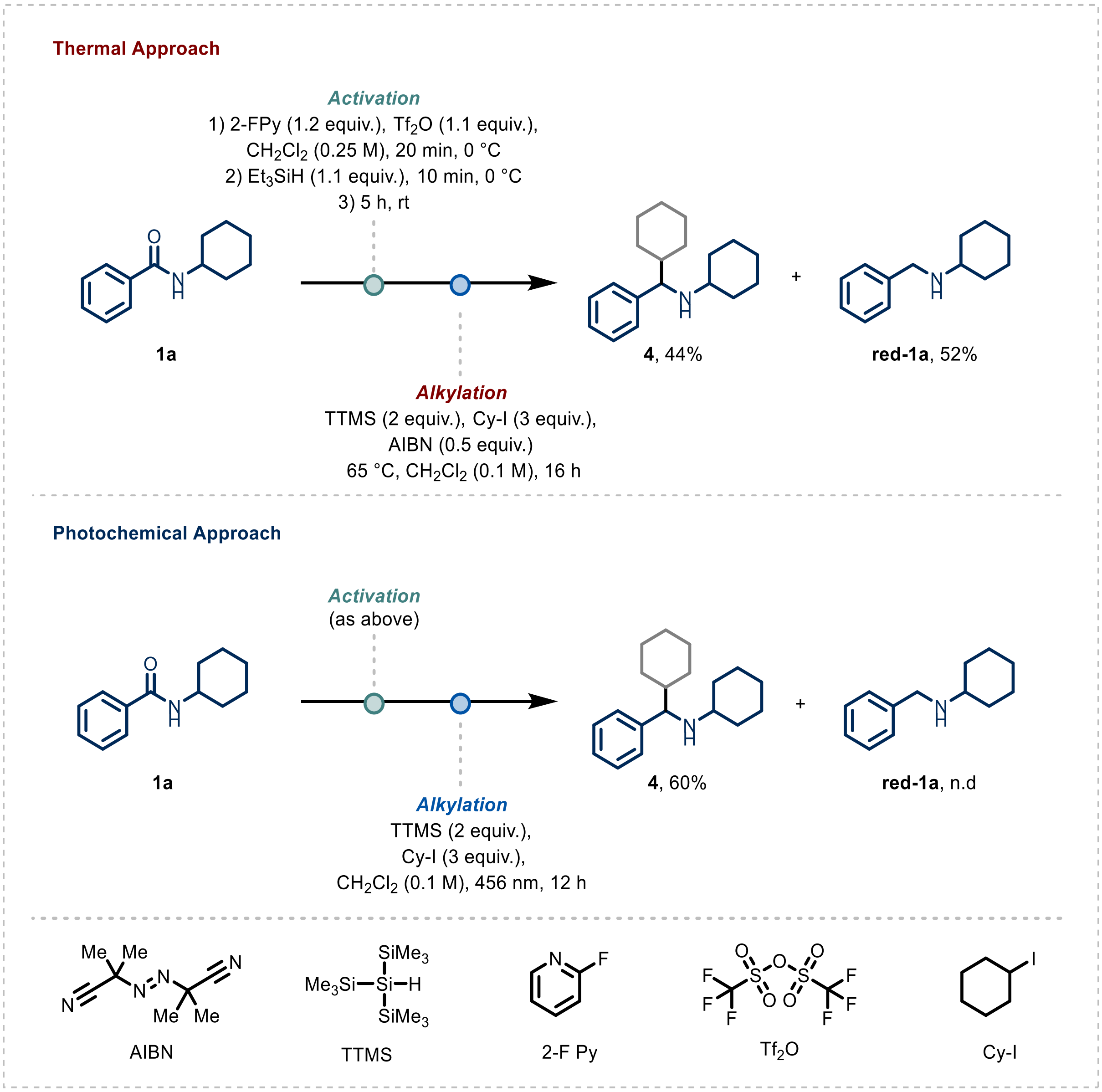

Preliminary experiments revealed that the in-situ formed iminium triflate could be intercepted by alkyl radicals, yielding the desired branched secondary amine **4**. However, when using a thermal approach, considerable amount of full reduction to the unsubstituted amine **red-1a** was observed. On the contrary, the photochemical approach afforded the target branched amine **4** in moderate yield and no over-reduction product **red-1a** was detected. Thus, we selected the photochemical approach for further investigation.

5.3 Optimization of the photochemical alkylation

Supplementary Table 3. Concentration and light source screening.

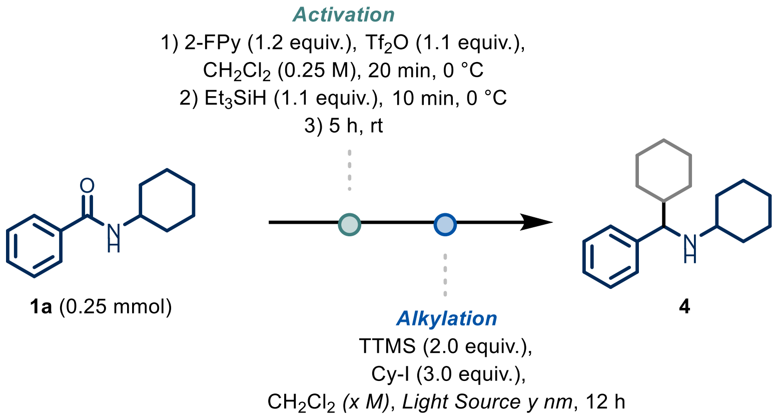

Concentration	Light Source	Yield of **4**
0.1 M	456 nm	60%
0.05 M	456 nm	65%
0.05 M	390 nm	75%
0.05 M	390 nm	76%[c]
0.05 M	none	n.d[b]

[a]Determined via ^{1}H NMR using trichloroethylene as external standard. [b]Quantitative recovery of imine **int-A**. [c]2 h instead of 5 h at rt after Et_3SiH addition.

Supplementary Table 4. Equivalents screening.

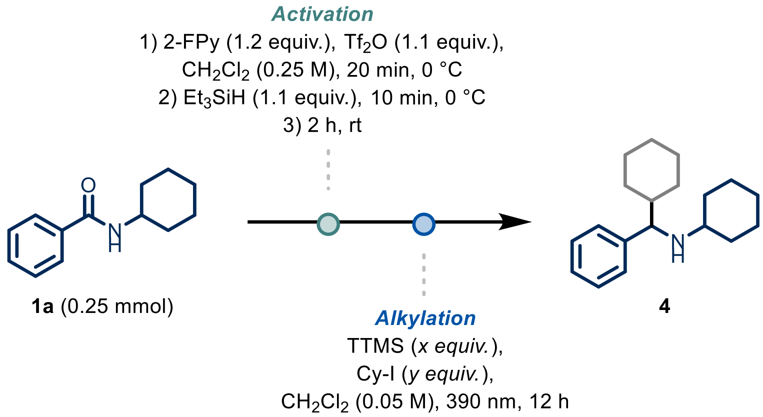

Cy-I (equiv.)	TTMS (equiv.)	Yield of **4**
none	2.0	n.d[b]
3.0	none	n.d[b]
1.5	1.0	55%
2.25	1.5	67%
3.0	2.0	76%

[a]Determined via ^{1}H NMR using trichloroethylene as external standard.
[b]Quantitative recovery of imine **int-A**.

Supplementary Table 5. Photochemical solvent screening.

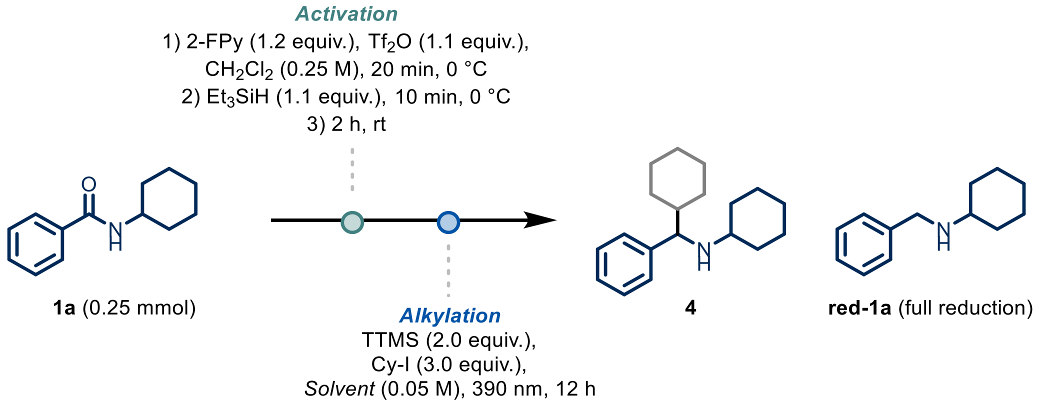

Solvent	Yield of **4**[a]	Comment
CH_2Cl_2:TFT (1:4)	74%	-
CH_2Cl_2:Acetone (1:4)	38%	messy NMR
CH_2Cl_2:MeCN (1:4)	80%	-
CH_2Cl_2:EtOAc (1:4)	32%	57% **red-1a**
CH_2Cl_2:DMSO (1:4)	15%	-
CH_2Cl_2:Cyclohexane (1:1)	62%	-
CH_2Cl_2:$CHCl_3$ (1:4)	58%	12% **red-1a**
CH_2Cl_2:PhCl (1:4)	77%	-

[a]Determined via ^{1}H NMR using trichloroethylene as external standard.

Supplementary Table 6. Additive screening.

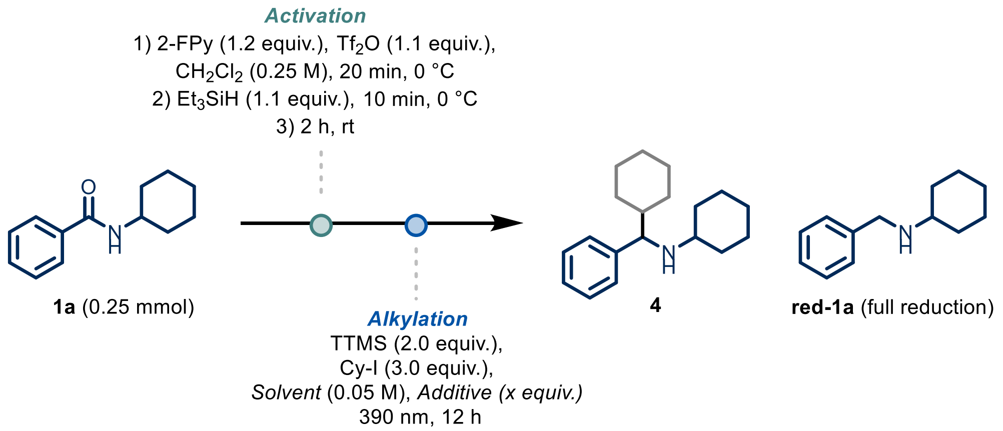

Solvent	Additive	Yield of **4**[a]	Yield of **red-1a**
CH_2Cl_2:MeCN (1:4)	-	80%	-
CH_2Cl_2	HFIP (2.5 equiv.)	60%	10%
CH_2Cl_2	iPrOH (5 equiv.)	65%	14%
CH_2Cl_2:MeCN (1:4)	iPrOH (5 equiv.)	77%	18%
CH_2Cl_2:MeCN (1:4)	aerobic, technical grade solvents	31%	-

[a]Determined via ^{1}H NMR using trichloroethylene as external standard.

Supplementary Table 7. Alkylation using alkyl bromides.

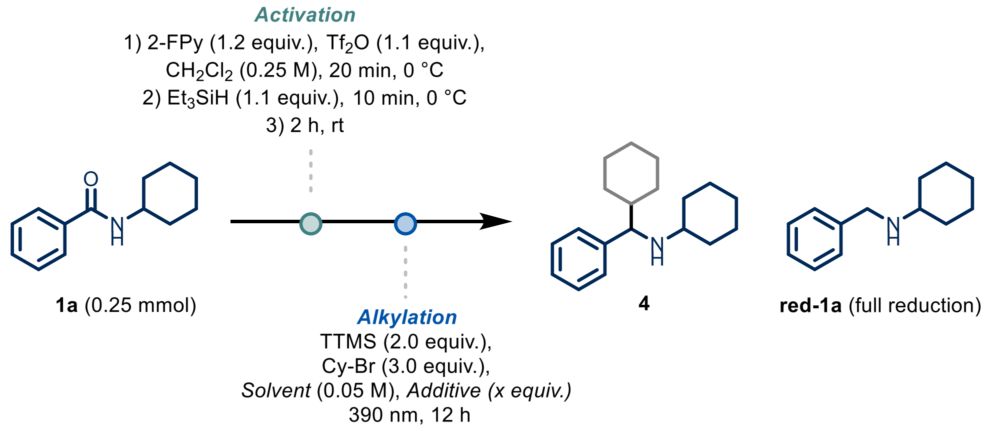

Solvent	Additive	Yield of **4**[a]	Yield of **red-1a**[a]
CH_2Cl_2	-	40%	-
CH_2Cl_2	10% ethyl 2-iodo propionate	46%	traces
CH_2Cl_2:MeCN (1:4)	-	traces	-
CH_2Cl_2:MeCN (1:4)	NaI (3.0 equiv.)	20%	-
CH_2Cl_2	NaI (3.0 equiv.)	67%	-
CH_2Cl_2	NaI (1.1 equiv.)	41%[b]	-

[a]Determined via ^{1}H NMR using trichloroethylene as external standard.
[b]TTMS (1.1 equiv.) and Cy-Br (1.1 equiv.)

Supplementary Table 8. One-pot protocol.

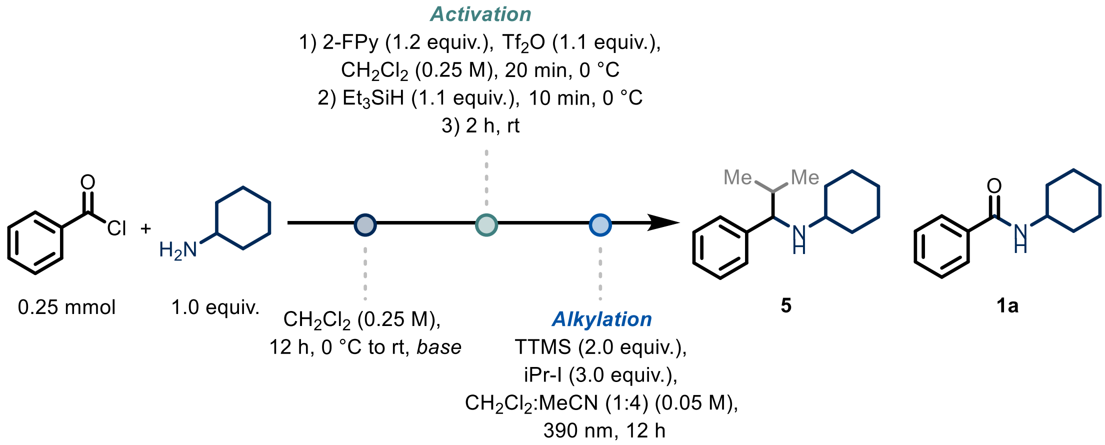

Base (1.1 equiv.)	Yield of **5**[a]	Yield of **1a**[a]	Comment
NEt_3	-	100%	activation and alkylation not performed
2-FPy	-	85%	activation and alkylation not performed
NEt_3	n.d.	100%	-
2-FPy	53%	25%	-

[a]Determined via ^{1}H NMR using trichloroethylene as external standard.

6. General procedure 5 (GP5): Deoxygenative alkylation of amides (activation at 0 ˚C)

In a typical experiment, to an oven-dried 7 mL vial equipped with a stirring bar was added the corresponding amide (0.25 mmol, 1.0 equiv.) and the vial was sealed with a septum. Subsequently, dry and degassed CH_2Cl_2 (1.0 mL) was added under N_2 atmosphere (0.25 M). The mixture was cooled at 0 ˚C with an ice-water bath and 2-fluoropyridine (29 mg, 26 µl, 0.30 mmol, 1.2 equiv.) was added. Triflic anhydride (78 mg, 47 µl, 0.28 mmol, 1.1 equiv.) was added slowly dropwise and the mixture was stirred (900 rpm) for 20 minutes at 0 ˚C. Triethylsilane (32 mg, 44 µl, 0.28 mmol, 1.1 equiv.) was added dropwise and the resulting mixture was stirred at 0 ˚C for additional 10 minutes. Then, the vial was removed from the ice-water bath and left stirring at room temperature for 2-5 h (the iminium ion formation can be monitored via UPLC-MS. See General information).
After, the solution was diluted with acetonitrile (4 mL, 0.05 M *final concentration*) and the corresponding alkyl iodide (0.75 mmol, 3.0 equiv.) and tris(trimethylsilyl)silane (124 mg, 154 µl, 0.50 mmol, 2.0 equiv.) were added. The vial was sealed with electrical tape and stirred and irradiated under 390 nm in the UFO photochemical reactor (**See section 4**) for 12 h. The temperature was maintained at 30-35 ˚C during the course of the reaction.
Finally, the vial was removed from the photochemical reactor and the solvent was evaporated under reduced pressure. The crude was suspended in *n*-pentane (10 mL) and sonicated for two minutes. The surnatant was filtered through a plug of celite to retain solid traces. This process was repeated three times. Finally the celite plug was washed with CH_2Cl_2 (3 × 5 mL). The CH_2Cl_2 phases were collected and added to the residual solid. The corresponding solution was then diluted with 15 mL of $NaHCO_3$ (~ 1:1 ratio organic:water phase) and the biphasic mixture was stirred for 15 minutes at rt. The solution was then transferred to a separatory funnel and extracted with CH_2Cl_2 (3 × 15 mL). The combined organic layers were dried over Na_2SO_4, filtered and the solvent was removed under reduced pressure. The crude reaction mixture was purified by flash column chromatography on silica gel.

*For the alkyl iodide scope (**43**-**54**) also the activation step was performed in acetonitrile.*

7. General procedure 6 (GP6): Deoxygenative alkylation of amides (activation at -78 ˚C)

In a typical experiment, to an oven-dried 7 mL vial equipped with a stirring bar was added the corresponding amide (0.25 mmol, 1.0 equiv.) and the vial was sealed with a septum. Subsequently, dry and degassed CH_2Cl_2 (1.0 mL) was added under N_2 atmosphere (0.25 M). The mixture was cooled at -78 ˚C with a liquid nitrogen-acetone bath and and 2-fluoropyridine (29 mg, 26 µl, 0.30 mmol, 1.2 equiv.) was added. Triflic anhydride (78 mg, 47 µl, 0.28 mmol, 1.1 equiv.) was added slowly dropwise and the mixture was stirred (900 rpm) for 10 minutes at -78 ˚C and then at 0 ˚C, using an ice-water bath, for additional 10 min (20 minutes total for the nitrilium ion formation).
Triethylsilane (32 mg, 44 µl, 0.28 mmol, 1.1 equiv.) was added dropwise and the resulting mixture was stirred at 0 ˚C for additional 10 minutes. Then, the vial was removed from the ice-water bath and left stirring at room temperature for 2-5 h (the iminium ion formation can be monitored via UPLC-MS. See General information).
After, the solution was diluted with acetonitrile (4 mL, 0.05 M *final concentration*) and the corresponding alkyl iodide (0.75 mmol, 3.0 equiv.) and tris(trimethylsilyl)silane (124 mg, 154 µl, 0.50 mmol, 2.0 equiv.) were added. The vial was sealed with electrical tape and stirred and irradiated under 390 nm in the UFO photochemical reactor (**See section 4**) for 12 h. The temperature was maintained at 30-35 ˚C during the course of the reaction.
Finally, the vial was removed from the photochemical reactor and the solvent was evaporated under reduced pressure. The crude was suspended in *n*-pentane (10 mL) and sonicated for two minutes. The surnatant was filtered through a plug of celite to retain solid traces. This process was repeated three times. Finally the celite plug was washed with CH_2Cl_2 (3 × 5 mL). The CH_2Cl_2 phases were collected and added to the residual solid. The corresponding solution was then diluted with 15 mL of $NaHCO_3$ (~ 1:1 ratio organic:water phase) and the biphasic mixture was stirred for 15 minutes at rt. The solution was then transferred to a separatory funnel and extracted with CH_2Cl_2 (3 × 15 mL). The combined organic layers were dried over Na_2SO_4, filtered and the solvent was removed under reduced pressure. The crude reaction mixture was purified by flash column chromatography on silica gel.

8. General procedure 7 (GP7): Deoxygenative alkylation of amides (activation at 0 ˚C) and subsequent cyclization: *N*-substituted piperidines synthesis.

Alkylation: In a typical experiment, to an oven-dried 7 mL vial equipped with a stirring bar was added the corresponding amide (0.25 mmol, 1.0 equiv.) and the vial was sealed with a septum. Subsequently, dry and degassed CH_2Cl_2 (1.0 mL) was added under N_2 atmosphere (0.25 M). The mixture was cooled at 0 ˚C with an ice-water bath and 2-fluoropyridine (29 mg, 26 µl, 0.30 mmol, 1.2 equiv.) was added. Triflic anhydride (78 mg, 47 µl, 0.28 mmol, 1.1 equiv.) was added slowly dropwise and the mixture was stirred (900 rpm) for 20 minutes at 0 ˚C. Triethylsilane (32 mg, 44 µl, 0.28 mmol, 1.1 equiv.) was added dropwise and the resulting mixture was stirred at 0 ˚C for additional 10 minutes. Then, the vial was removed from the ice-water bath and left stirring at room temperature for 5 h.
After, the solution was diluted with acetonitrile (4 mL, 0.05 M *final concentration*) and the corresponding alkyl iodide (0.75 mmol, 3.0 equiv.) and tris(trimethylsilyl)silane (124 mg, 154 µl, 0.50 mmol, 2.0 equiv.) were added. The vial was sealed with electrical tape and stirred and irradiated under 390 nm in the UFO photochemical reactor (**See section 4**) for 12 h. The temperature was maintained at 30-35 ˚C during the course of the reaction.
Finally, the vial was removed from the photochemical reactor and the solvent was evaporated under reduced pressure. The crude was suspended in *n*-pentane (10 mL) and sonicated for two minutes. The surnatant was filtered through a plug of celite to retain solid traces. This process was repeated three times. Finally, the celite plug was washed with CH_2Cl_2 (3 × 5 mL). The CH_2Cl_2 phases were collected and added to the residual solid. The corresponding solution was then diluted with 15 mL of $NaHCO_3$ (~ 1:1 ratio organic:water phase) and the biphasic mixture was stirred for 15 minutes at rt. The solution was then transferred to a separatory funnel and extracted with CH_2Cl_2 (3 × 15 mL). The combined organic layers were dried over Na_2SO_4, filtered and the solvent was removed under reduced pressure.

Cyclization: Then, anhydrous $NaHCO_3$ (42 mg, 0.50 mmol, 2.0 equiv.), NaI (75 mg, 0.125 mmol, 0.5 equiv.) and a stirring bar were added to the flask containing the crude reaction mixture and it was sealed with a rubber septum. Next, dry acetonitrile was added (2.5 mL, 0.1 M) and the flask was placed in an oil bath at 80˚C for 2 h. The reaction mixture was cooled to rt and filtered using a sintered funnel, rinsed with dichloromethane and the solvent was removed under reduced pressure. The crude reaction mixture was purified by flash column chromatography on silica gel.

9. Mechanistic investigation

9.1 UV-Vis Characterization

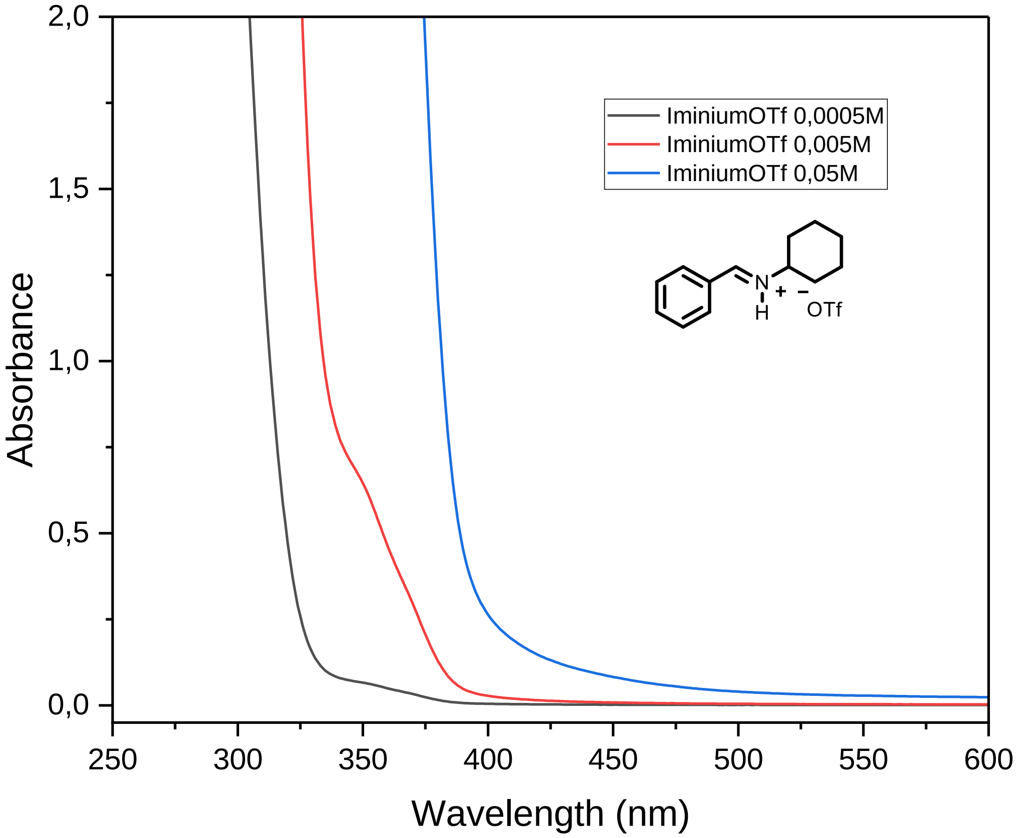

Supplementary Figure 7: Absorption spectra of prepared iminium triflate **int-A**. The iminium **int-A** was independently prepared by reacting a dichloromethane solution (0.25 M) of the corresponding imine (obtained from the semi-reduction of **1a**) with triflic acid (1.0 equiv) at 0 ˚C. The spectra were recorded in dichlomethane:acetonitrile (1:4) in quartz cuvettes (optical path: 1 cm) with a bandwidth of 5 nm and a data pitch of 1 nm. Scan rate: medium.

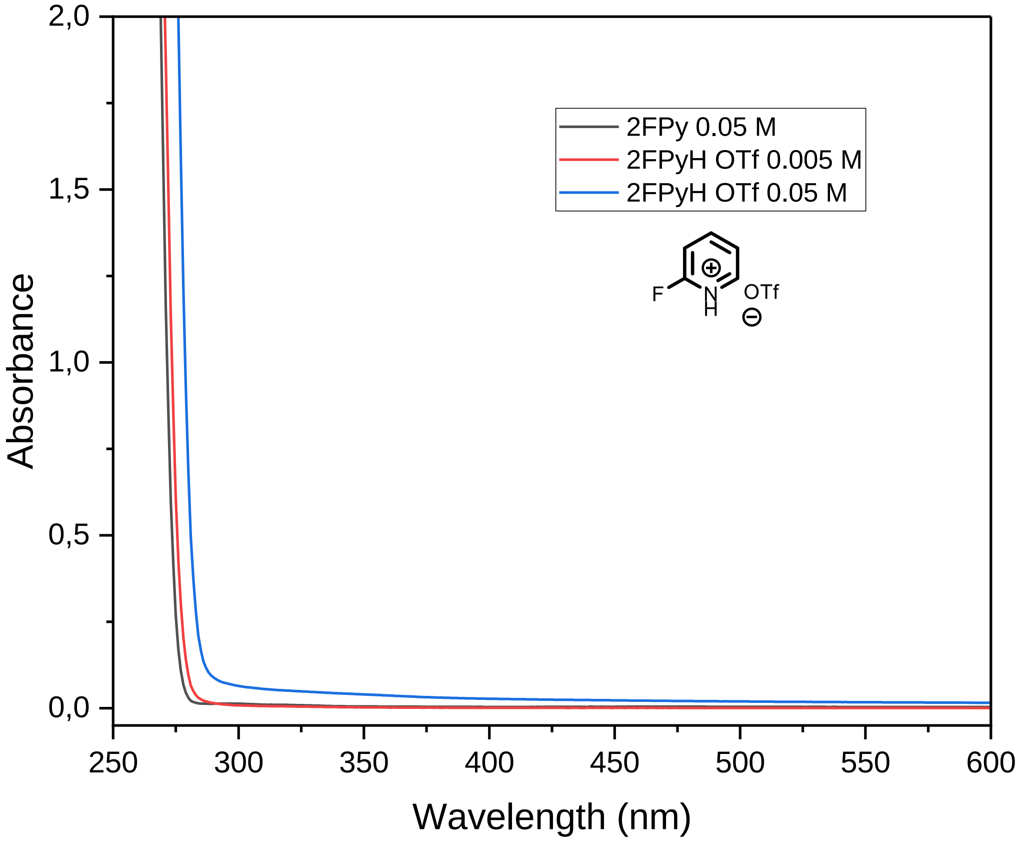

Supplementary Figure 8: Absorption spectrum of the 2-FPyH triflate formed by reacting a dichloromethane solution (0.25 M) of 2-fluoropyridine with triflic acid (1.0 equiv) at 0 ˚C The spectrum was recorded in dichlomethane:acetonitrile (1:4) in quartz cuvettes (optical path: 1 cm) with a bandwidth of 5 nm and a data pitch of 1 nm. Scan rate: medium.

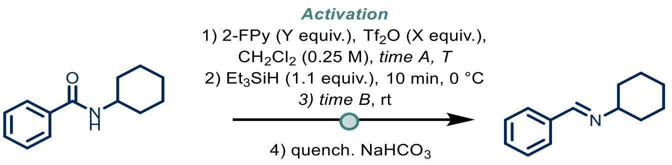

1a (0.25 mmol) **Int-A**

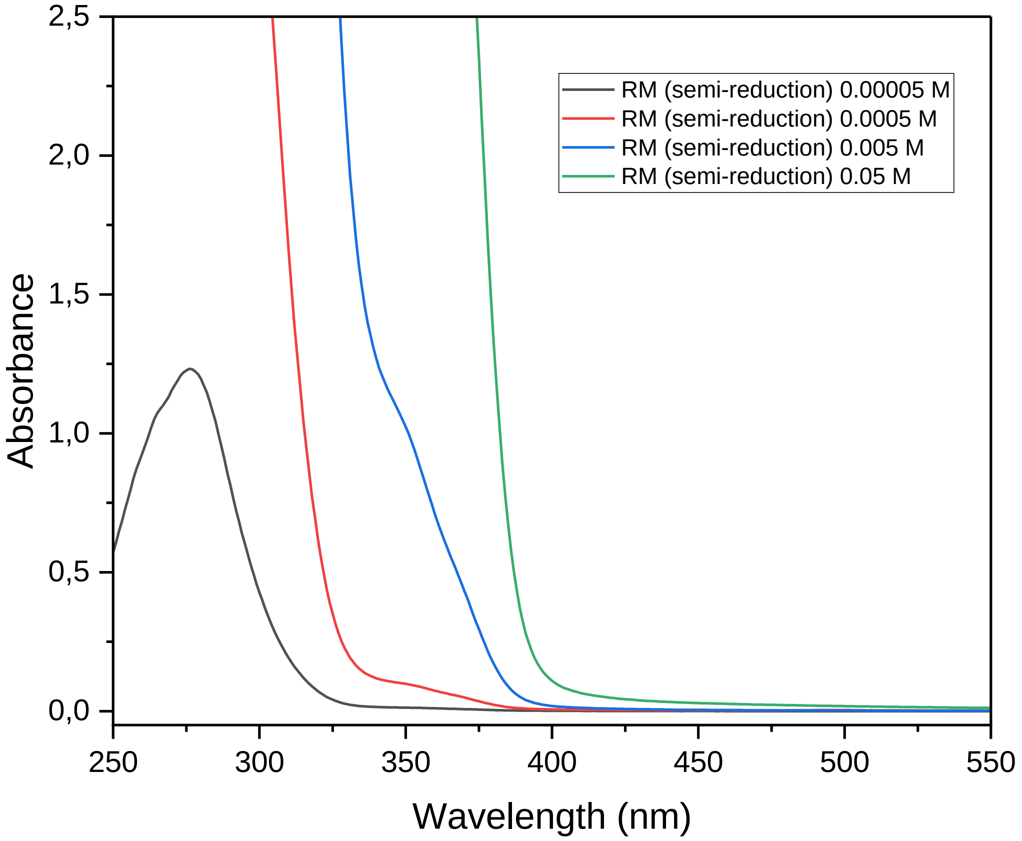

Supplementary Figure 9: Absorption spectrum of the reaction mixture obtained after semi-reduction of amide **1a**. The spectrum was recorded in dichlomethane:acetonitrile (1:4) in quartz cuvettes (optical path: 1 cm) with a bandwidth of 5 nm and a data pitch of 1 nm. Scan rate: medium (RM = reaction mixture).

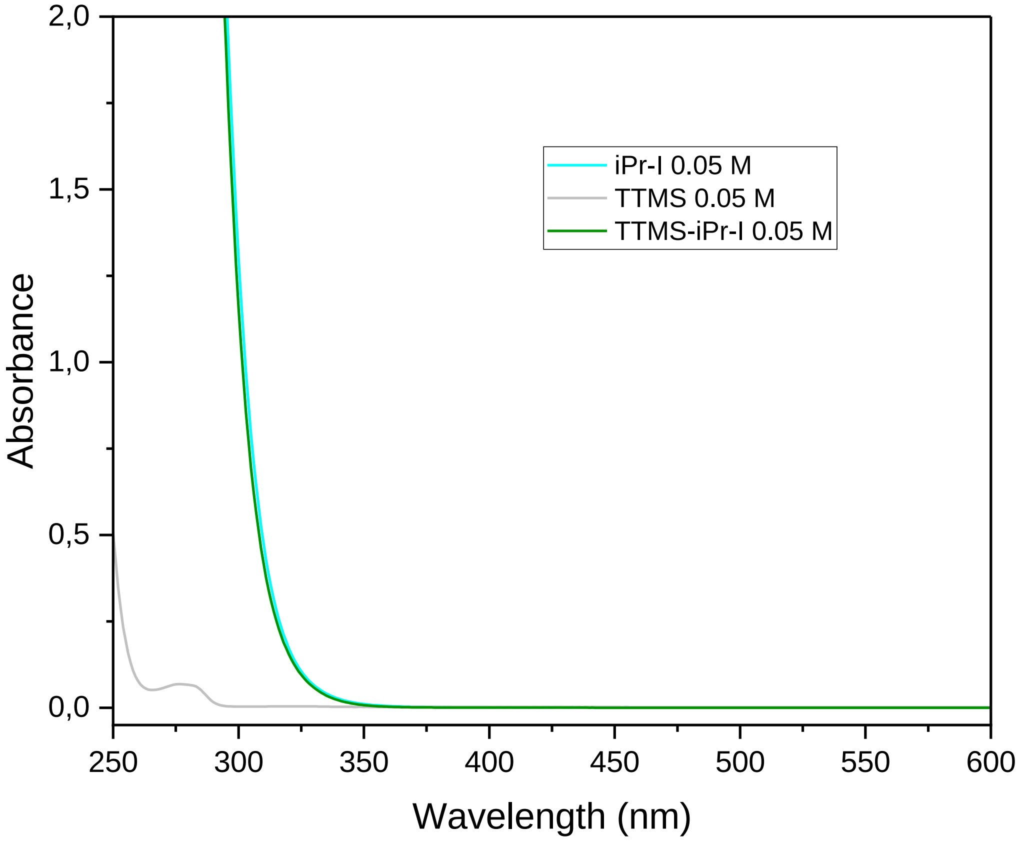

Supplementary Figure 10: Absorption spectrum of the components isopropyl-iodide, TTMS and a combination 1.5:1 *i*Pr-I:TTMS. The spectrum was recorded in dichlomethane:acetonitrile (1:4) in quartz cuvettes (optical path: 1 cm) with a bandwidth of 5 nm and a data pitch of 1 nm. Scan rate: medium.

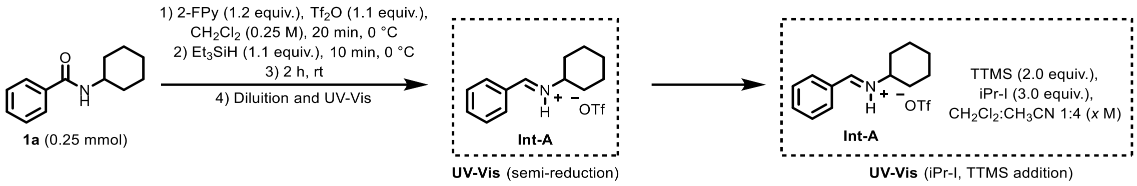

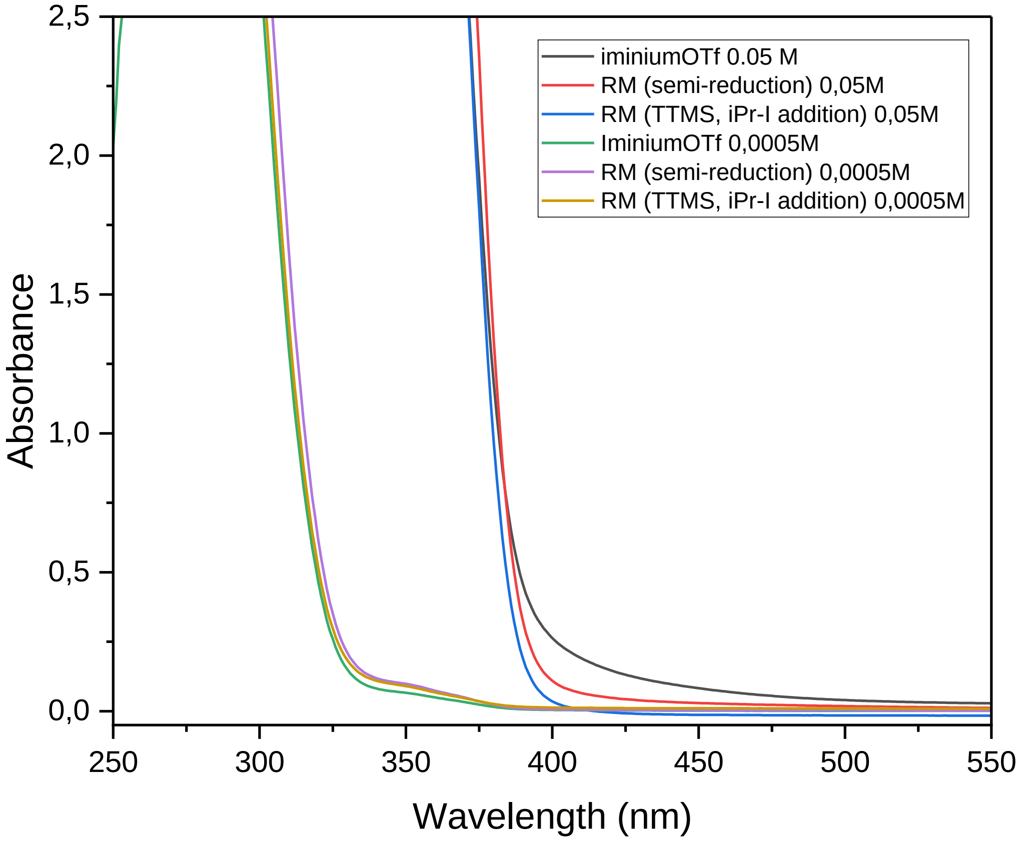

Supplementary Figure 11: Absorption spectrum of the reaction mixture obtained after semi-reduction of amide **1a** vs absorption spectrum of the final reaction mixture after addition of iPr-I and TTMS. The spectrum was recorded in dichlomethane:acetonitrile (1:4) in quartz cuvettes (optical path: 1 cm) with a bandwidth of 5 nm and a data pitch of 1 nm. Scan rate: medium (RM = reaction mixture).

According to these UV-Vis studies there is no evidence for the interaction between the iminium triflate **int-A** (obtained after the semi-reduction of **1a**) and the alkylating agents (*i*Pr-I and TTMS). According to literature precedents, we propose that the weak absorption of UV-light by alkyl iodides leads to the generation of carbon radicals, thus initiating the radical chain.[6-8]

9.2 TEMPO radical trapping

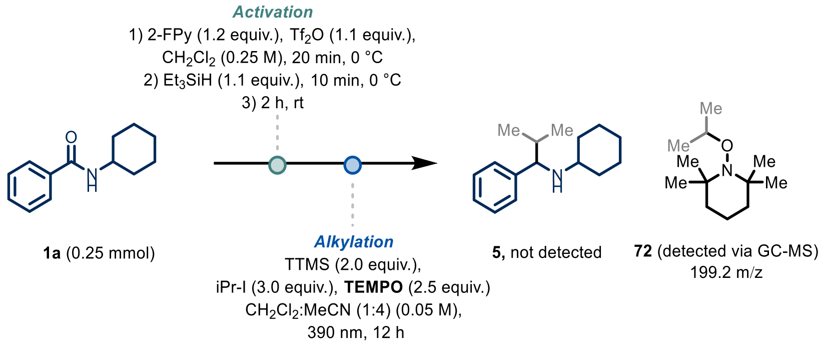

To an oven-dried 7 mL vial equipped with a stirring bar was added the amide **1a** (51 mg, 0.25 mmol, 1.0 equiv.) and the vial was sealed with a septum. Subsequently, dry and degassed CH_2Cl_2 (1.0 mL) was added under N_2 atmosphere (0.25 M). The mixture was cooled at 0 ˚C with an ice-water bath and 2-fluoropyridine (29 mg, 26 µl, 0.30 mmol, 1.2 equiv.) was added. Triflic anhydride (78 mg, 47 µl, 0.28 mmol, 1.1 equiv.) was added slowly dropwise and the mixture was stirred (900 rpm) for 20 minutes at 0 ˚C. Triethylsilane (32 mg, 44 µl, 0.28 mmol, 1.1 equiv.) was added dropwise and the resulting mixture was stirred at 0 ˚C for additional 10 minutes. Then, the vial was removed from the ice-water bath and left stirring at room temperature for 5 h.
After, the solution was diluted with acetonitrile (4 mL, 0.05 M *final concentration*) and isopropyl iodide (76 µL, 0.75 mol, 3 equiv.), tris(trimethylsilyl)silane (124 mg, 154 µl, 0.50 mmol, 2.0 equiv.) and TEMPO (117 mg, 0.75 mmol, 3.0 equiv.) were added. The vial was sealed with electrical tape and stirred and irradiated under 390 nm in the UFO photochemical reactor (**See section 4**) for 12 h. The temperature was maintained at 30-35 ˚C during the course of the reaction.
Then, the reaction mixture was transferred to a round bottom flask and diluted with CH_2Cl_2 (15 mL). The corresponding solution was then diluted with 15 mL of $NaHCO_3$ (~ 1:1 ratio organic:water phase) and the biphasic mixture was stirred for 15 minutes at rt. The solution was then transferred to a separatory funnel and extracted with CH_2Cl_2 (3 × 15 mL). The combined organic layers were dried over Na_2SO_4, filtered and the solvent was removed under reduced pressure. The final crude reaction mixture was analyzed via GC-MS.

9.3 Radical Clock Experiment

5-exo-trig experiment

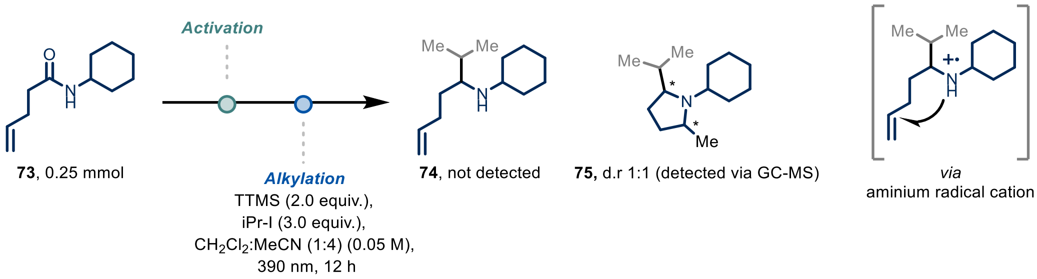

The radical clock experiment reported herein was performed according to GP6.

The result of this experiment implies that the most likely event for the C-C bond formation is the radical addition to a charged iminium triflate. Indeed, after radical addition the incipient aminium radical cation intermediate undergoes radical 5-exo-trig to afford **75** in 1:1 d.r.
Additionally, the sole formation of **75** suggests that the iminium triflate is not acting as an oxidant, otherwise product **74** should have been detected.

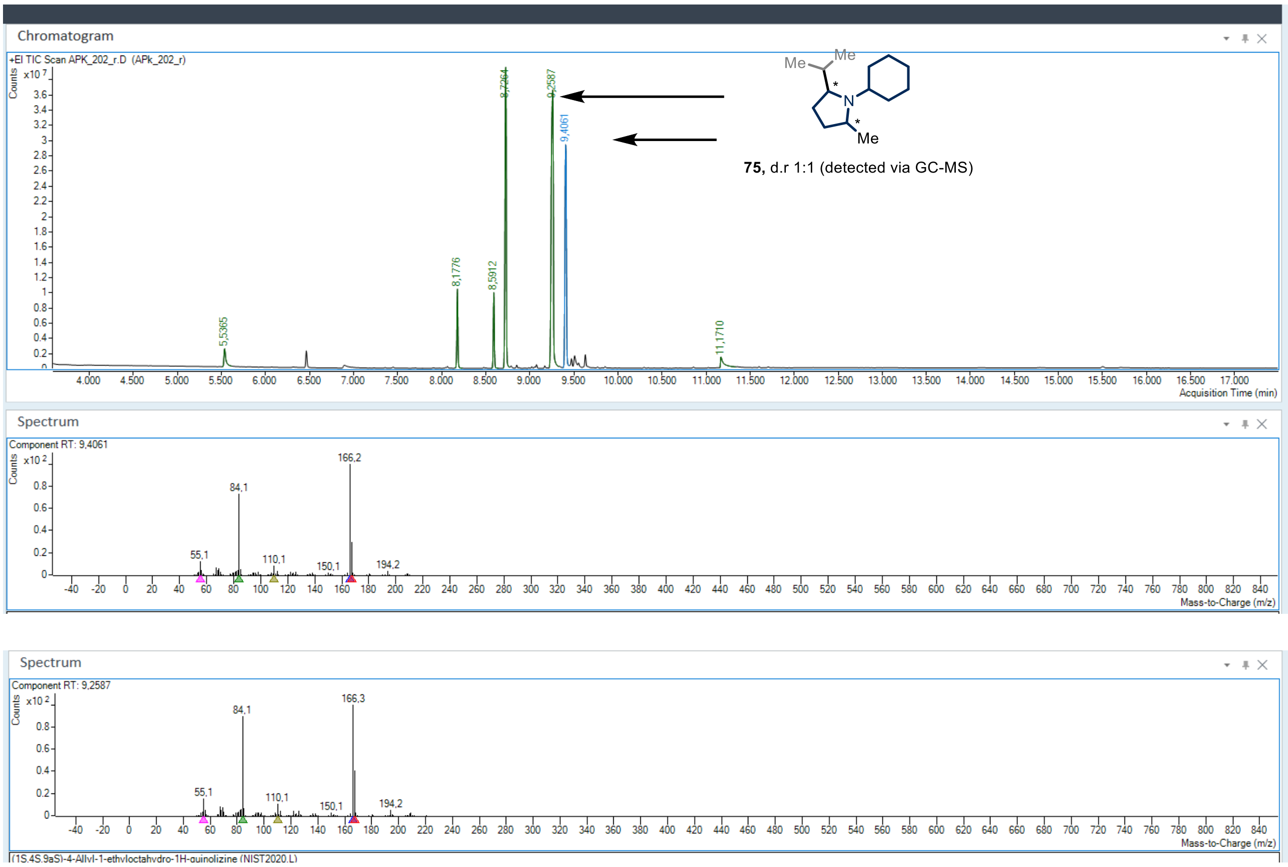

Supplementary Figure 12: GC trace of the crude reaction (formation of the two diastereoisomers of **75**).

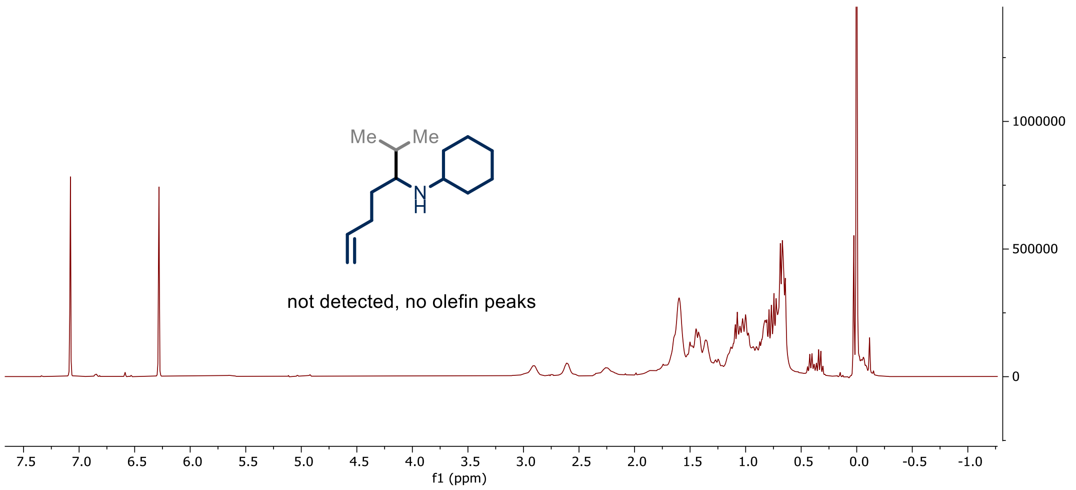

Supplementary Figure 13: ^{1}H NMR of the crude reaction.

10. Scale-up procedure for compounds 5, 79

10.1 Re-optimization of the reaction conditions in batch for compound 5

In this section is reported the re-optimization of the reaction conditions in order to obtain a more green and efficient process. The issues associated with the scalability of the developed protocol are: the use of halogenated solvent, low concentration, excess of both alkyl iodides and XAT agent TTMS and long reaction times.

Supplementary Table 9. Scalability issues.

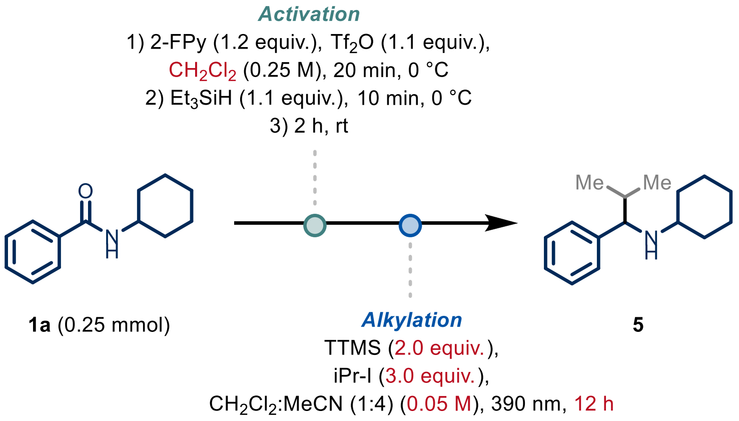

Problematic parameters when scaling-up:

- Chlorinated solvent (activation step)
- Over-stoichiometric reactants
- Low concentration
- Long reaction times

Supplementary Table 10. Solvent and concentration screening.

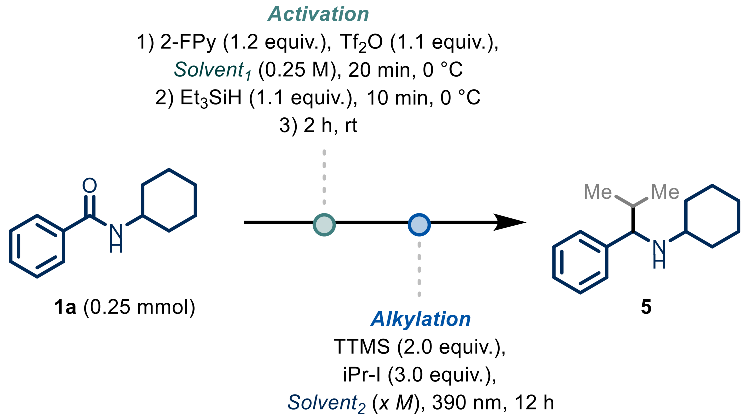

Solvent$_1$:Solvent$_2$	Concentration	Yield of **5**[a]
CH_2Cl_2:MeCN (1:4)	0.05 M	80%
MeCN:MeCN (1:4)	0.05 M	77%
MeCN:MeCN (1:2)	0.08 M	78%
MeCN:MeCN (1:1)	0.125 M	68%
MeCN: –	0.25 M	67%
MeCN:MeCN (1:1)	0.125 M	78% (1.0 mmol)
MeCN: –	0.25 M	77% (1.0 mmol)
MeCN: –	0.5 M	70%
iPrOH: –	0.25 M	n.d.
2-MeTHF: –	0.25 M	n.d.

[a]Determined via ^{1}H NMR using trichloroethylene as external standard.

Supplementary Table 11. Equivalents of TTMS.

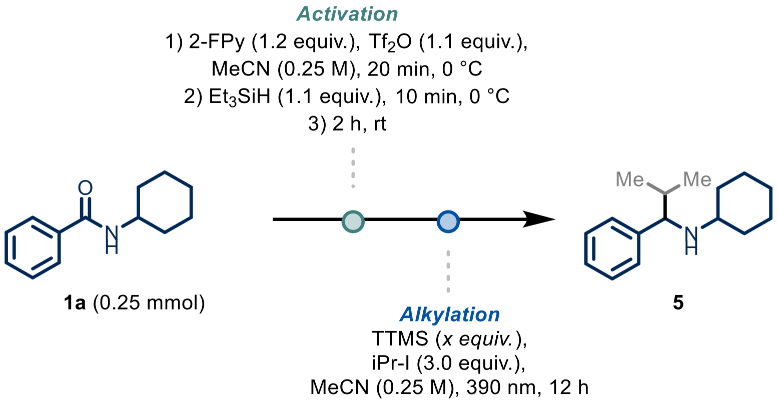

1a (0.25 mmol) → **5**

TTMS (equiv.)	Yield of **5**[a]
2.0	67%
1.5	66%
1.1	70%

[a]Determined via ^{1}H NMR using trichloroethylene as external standard.

Supplementary Table 12. Equivalents of iPr-I.

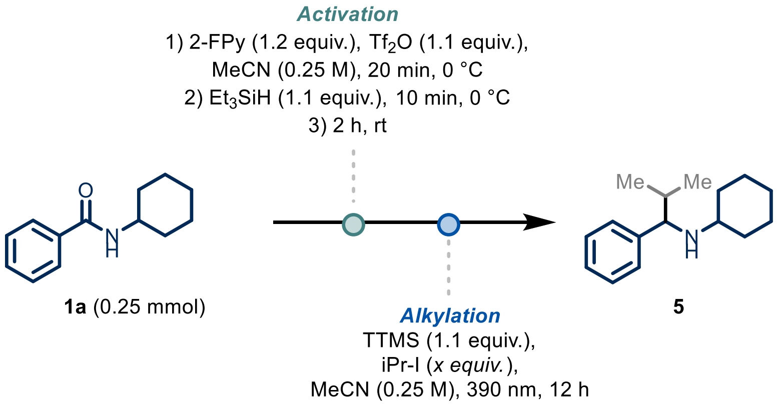

iPr-I (equiv.)	Yield of **5**[a]
3.0	70%
2.0	66%
1.5	56%
1.1	60%
1.1	54% (5.0 mmol)

[a]Determined via ^1H NMR using trichloroethylene as external standard.

5 mmol scale

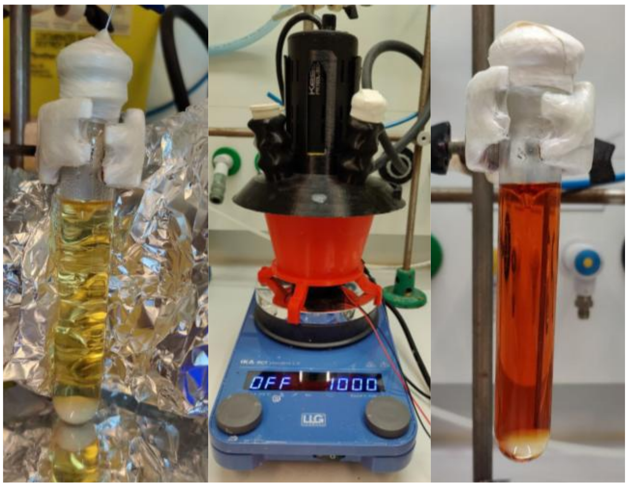

Before irradiation After irradiation

Supplementary Table 13. Effect of the light intensity.

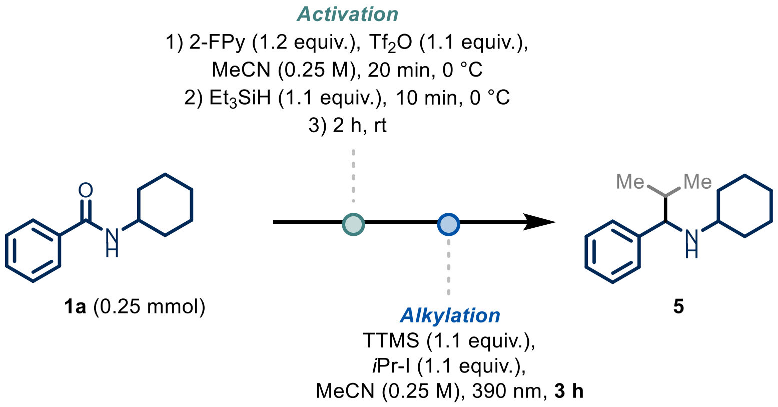

Light Intensity	Yield **5**[a]
100%	65%
50%	5%
25%	<5%

[a]Determined via ^{1}H NMR using trichloroethylene as external standard.

10.2 Optimization and scale-up in continuous flow of compound 5, 79

10.2.1 General procedure (GP7) for the deoxygenative alkylation of amides in continuous flow

In a typical experiment, to an oven-dried 7 mL vial equipped with a stirring bar was added the corresponding amide (0.50 mmol, 1 equiv.) and the vial was sealed with a septum. Subsequently, dry and degassed acetonitrile (2.0 mL) was added under N_2 atmosphere (0.25 M). The mixture was cooled at 0 ˚C with an ice-water bath and 2-fluoropyridine (52 µl, 1.2 equiv.) was added. Triflic anhydride (93 µl, 1.1 equiv.) was added slowly dropwise and the mixture was stirred (900 rpm) for 20 minutes at 0 ˚C. Triethylsilane (88 µl, 1.1 equiv.) was added dropwise and the resulting mixture was stirred at 0 ˚C for additional 10 minutes. Then, the vial was removed from the ice-water bath and left stirring at room temperature for 2 h.

For the 0.125 M screening: After, the solution was diluted with acetonitrile (2 mL, 0.125 M final concentration) and isopropyl iodide (1.1 or 1.5 equiv.) and tris(trimethylsilyl)silane (170 µl, 1.1 equiv.) were added.
For the 0.25 M screening: After, the isopropyl iodide (1.1 or 1.5 equiv.) and tris(trimethylsilyl)silane (170 µl, 1.1 equiv.) were directly added to the reaction mixture (0.25 M final concentration).

Next,the final solution was taken out with a 6 mL syringe under under N_2 atmosphere. The syringe was mounted on a syringe pump and pushed into the Signify photoreactor (V = 2.8 mL) equipped with UV LEDs (λ = 365 nm, 144 W) or blue LEDs (λ = 456 nm, 188 W) for the required residence time.[9] The outflow was collected in a 25 mL round-bottom flask. When the syringe was fully empty, acetonitrile was loaded into a new 6 mL syringe and pushed into the reactor to collect all the reaction mixture. Finally, the solvent was evaporated under reduced pressure. The crude was suspended in n-pentane (10 mL) and sonicated for two minutes. The surnatant was filtered through a plug of celite to retain solid traces. This process was repeated three times. Finally, the celite plug was washed with CH_2Cl_2 (3 × 5 mL). The CH_2Cl_2 phases were collected and added to the residual solid. The corresponding solution was then diluted with 15 mL of $NaHCO_3$ (~ 1:1 ratio organic:water phase) and the biphasic mixture was stirred for 15 minutes at rt. The solution was then transferred to a separatory funnel and extracted with CH_2Cl_2 (2 × 15 mL). The combined organic layers were dried over Na_2SO_4, filtered and the solvent was removed under reduced pressure.
The final crude was dissolved in $CDCl_3$ and analyzed via ^{1}H-NMR using trichloroethylene as external standard.

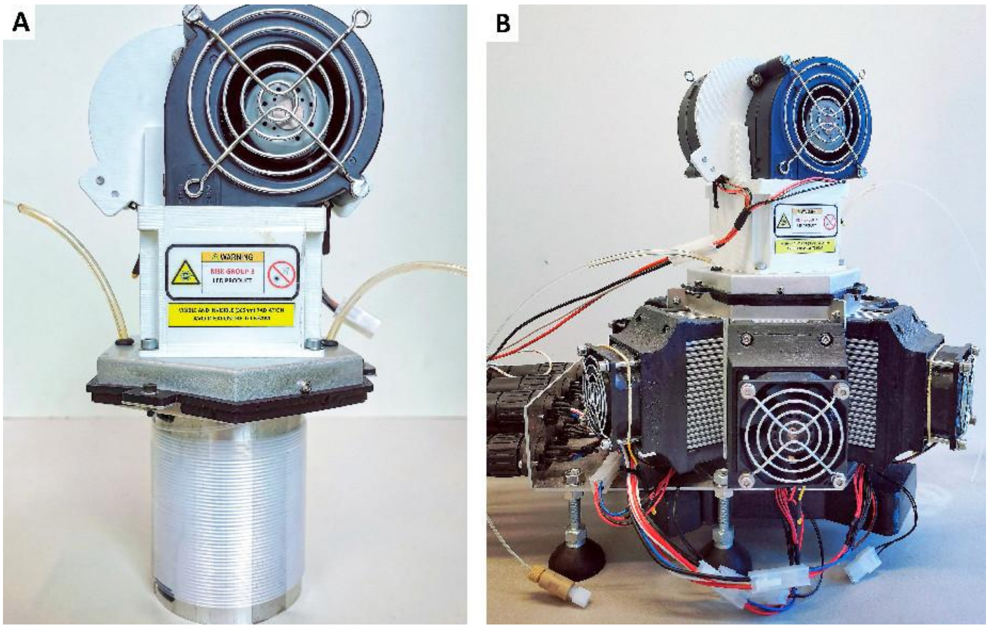

Supplementary Figure 14. Signify's (Eagle) photoreactor 365 nm (144 W) or 456 nm (188 W).

Supplementary Table 14. Screening residence time for compound **5** (0.25 M).

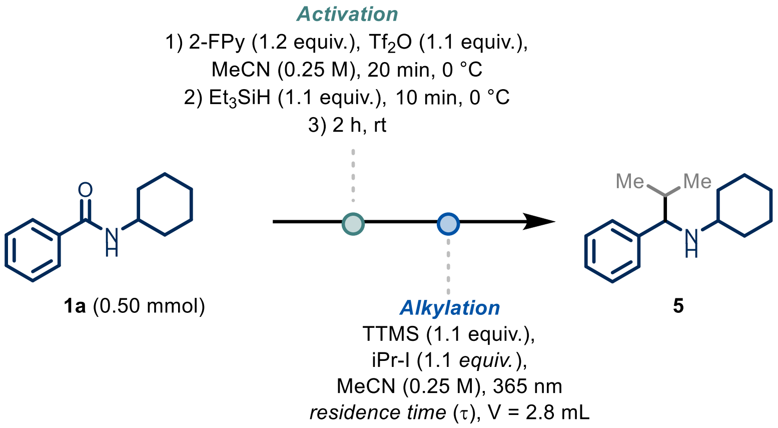

Residence time (τ)	Yield of **5**[a]	Residual SM
60 min	57%	0%
30 min	61%	0%
15 min	55%	0%
10 min	63%	0%
5 min	53%	18%
2 min	31%	54%
1 min	8%	73%

[a]Determined via ^{1}H NMR using trichloroethylene as external standard.

Supplementary Table 15. Screening residence time for compound **5** (0.125 M).

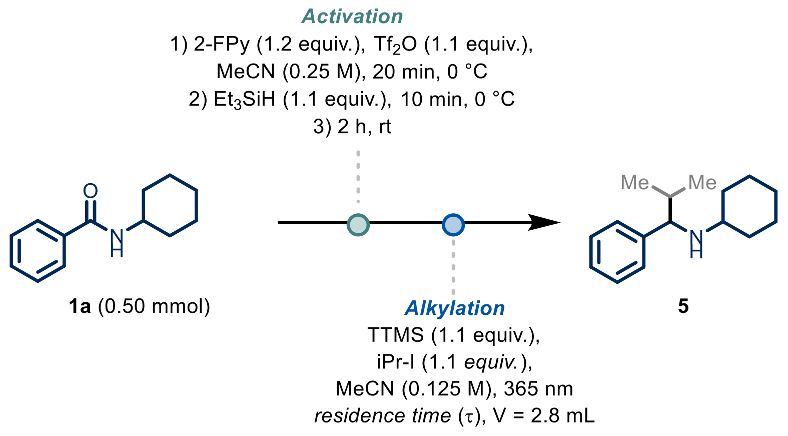

Residence time (τ)	Yield of **5**[a]	Residual SM
2 min	36%	58%
5 min	55%	18%
10 min	57%	0%
60 min[b]	51%	0%

[a]Determined via ^{1}H NMR using trichloroethylene as external standard.
[b]The reaction was performed at 456 nm.

Supplementary Table 16. Additional optimization for compound **5** (1.5 equiv. iPr-I).

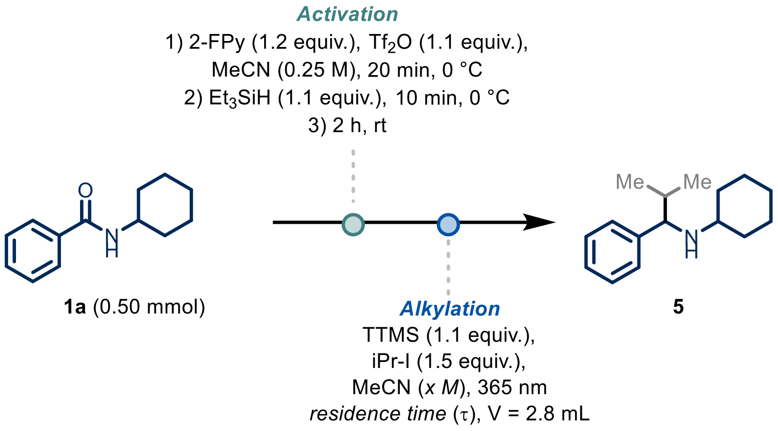

Residence time (τ)	Concentration	Yield of **5**[a]
5 min	0.25 M	71%
10 min	0.25 M	72%
10 min	0.125 M	70%

[a]Determined via ^{1}H NMR using trichloroethylene as external standard.

Supplementary Table 17. Optimization for *N*-substituted azacycle **79**.

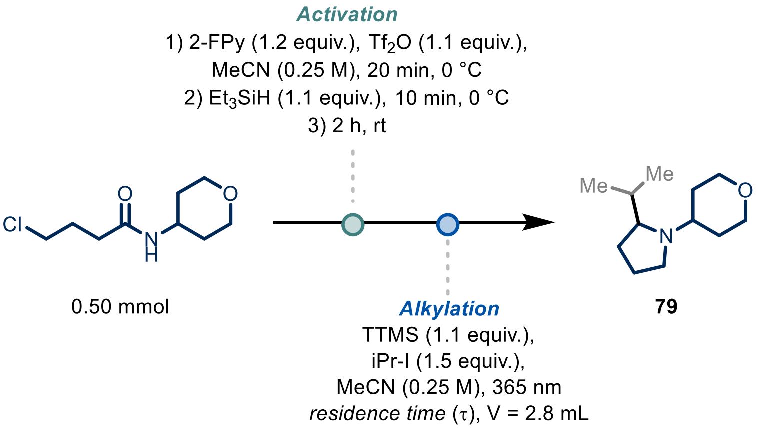

Residence time (τ)	Yield of **79**[a]
2 min	44%
5 min	72%
10 min	73%
5 min[b]	73%

[a]Determined via ^{1}H NMR using trichloroethylene as external standard.
[b]The reaction was carried out with 1.1 equiv. iPr-I.

10.2.2 *Scale-up (10 mmol) of compound **5** in flow*

To an oven dried 100 mL round bottom flask equipped with a stirring bar was added the amide **1a** (2.0 g, 10 mmol, 1.0 equiv.) and the round bottom flask was sealed with a septum. Subsequently, dry and degassed acetonitrile (40 mL) was added under N_2 atmosphere (0.25 M). The mixture was cooled at 0 ˚C with an ice-water bath and 2-fluoropyridine (1.2 g, 1.0 mL, 12 mmol, 1.2 equiv.) was added. Triflic anhydride (3.1 g, 1.9 mL, 11 mmol, 1.1 equiv.) was added slowly dropwise and the mixture was stirred (900 rpm) for 20 minutes at 0 ˚C. Triethylsilane (1.3 g, 1.8 mL, 11 mmol, 1.1 equiv.) was added dropwise and the resulting mixture was stirred at 0 ˚C for additional 10 minutes. Then, the round bottom flask was removed from the ice-water bath and left stirring at room temperature for 2 h.

For the scale-up at 0.125 M: After, the solution was diluted with acetonitrile (40 mL) and the isopropyl iodide (2.6 g, 1.5 mL, 15 mmol, 1.5 equiv.) and tris(trimethylsilyl)silane (2.7 g, 3.4 mL, 11 mmol, 1.1 equiv.) were added.
Next, the final solution was taken out with a 60 mL syringe under under N_2 atmosphere (2 × 40 mL). The syringe was mounted on a syringe pump and pushed into the Signify photoreactor (V = 2.8 mL) equipped with UV LEDs (λ= 365 nm, 144 W) with a flow rate of 0.28 mL·min^{-1} (τ = 10 min). The outflow was collected in a 250 mL round-bottom flask. When the syringe was fully empty, acetonitrile was loaded into a 6 mL syringe and pushed into the reactor to collect all the reaction mixture. Finally, the solvent was evaporated under reduced pressure. The crude was suspended in n-pentane and sonicated for two minutes. The surnatant was filtered through a plug of celite to retain solid traces. This process was repeated three times. Finally, the celite plug was washed three times with CH_2Cl_2. The CH_2Cl_2 phases were collected and added to the residual solid. The corresponding solution was then diluted with $NaHCO_3$ (~ 1:1 ratio organic:water phase) and the biphasic mixture was stirred for 15 minutes at rt. The solution was then transferred to a separatory funnel and extracted three times with CH_2Cl_2. The combined organic layers were dried over Na_2SO_4, filtered and the solvent was removed under reduced pressure. The residue was purified via flash column chromatography on silica gel (Pentane:Ethyl Acetate 20:1) to afford the product as a colorless oil (1.64 g, 71% yield).
For the scale-up at 0.25 M: After, the corresponding alkyl iodide (2.6 g, 1.5 mL, 15 mmol, 1.5 equiv.) and tris(trimethylsilyl)silane (2.7 g, 3.4 mL, 11 mmol, 1.1 equiv.) were directly added in the reaction mixture.
Next, the final emulsion (TTMS not fully soluble in acetonitrile at 0.25 M) was directly pumped, under N_2 atmosphere, from the reaction flask while stirring into the Signify photoreactor (V = 2.8 mL) equipped with UV LEDs (λ= 365 nm, 144 W) using a peristaltic pump (Masterflex ismatec) with a flow rate of 0.56 mL·min^{-1} (τ = 5 min). The outflow was collected in a 100 mL round-bottom flask. When the flask was fully empty, 6 mL of acetonitrile were loaded into the reaction flask and pushed into the reactor to collect all the product. Finally, the solvent was evaporated under reduced pressure. The crude was suspended in n-pentane and sonicated for two minutes. The surnatant was filtered through a plug of celite to retain solid traces. This process was repeated three times. Finally the celite plug was washed three times with CH_2Cl_2. The CH_2Cl_2 phases were collected and added to the residual solid. The corresponding solution was then diluted with $NaHCO_3$ (~ 1:1 ratio organic:water phase) and the biphasic mixture was stirred for 15 minutes at rt. The solution was then transferred to a separatory funnel and extracted three times with CH_2Cl_2. The combined organic layers were dried over Na_2SO_4, filtered and the solvent was removed under reduced pressure. The residue was purified via flash column chromatography on silica gel (Pentane:Ethyl Acetate 30:1) to afford the product as a colorless oil (1.48 g, 64% yield).

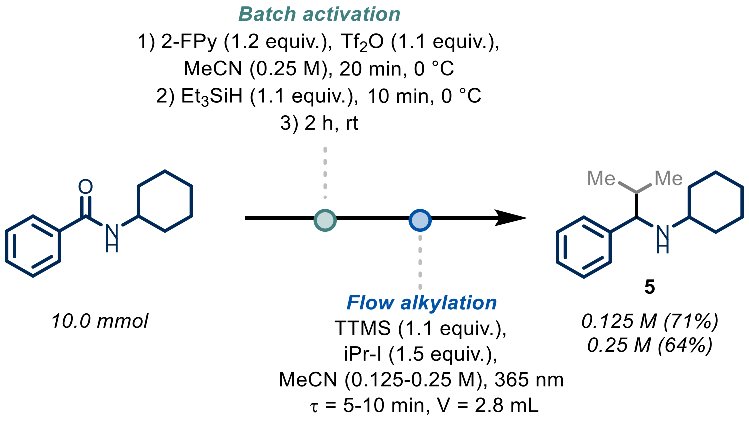

*10.2.3 Scale-up (10 mmol) of compound **79** in flow (cyclization)*

To an oven dried 100 mL round bottom flask equipped with a stirring bar was added the corresponding amide (2.0 g, 10 mmol, 1.0 equiv.) and the round bottom flask was sealed with a septum. Subsequently, dry and degassed acetonitrile (40 mL) was added under N_2 atmosphere (0.25 M). The mixture was cooled at 0 ˚C with an ice-water bath and 2-fluoropyridine (1.2 g, 1.0 mL, 12 mmol, 1.2 equiv.) was added. Triflic anhydride (3.1 g, 1.9 mL, 11 mmol, 1.1 equiv.) was added slowly dropwise and the mixture was stirred (900 rpm) for 20 minutes at 0 ˚C. Triethylsilane (1.3 g, 1.8 mL, 11 mmol, 1.1 equiv.) was added dropwise and the resulting mixture was stirred at 0 ˚C for additional 10 minutes. Then, the round bottom flask was removed from the ice-water bath and left stirring at room temperature for 2 h.

After, the isopropyl iodide (2.6 g, 1.5 mL, 15 mmol, 1.5 equiv.) and tris(trimethylsilyl)silane (2.7 g, 3.4 mL, 11 mmol, 1.1 equiv.) were directly added in the reaction flask.

Next, the final emulsion (TTMS not fully soluble in acetonitrile at 0.25 M) was directly pumped, under N_2 atmosphere, from the reaction flask while stirring into the Signify photoreactor (V = 2.8 mL) equipped with UV LEDs (λ = 365 nm, 144 W) using a peristaltic pump (Masterflex ismatec) with a flow rate of 0.56 mL·min^{-1} (τ = 5 min). The outflow was collected in a 100 mL round-bottom flask. When the flask was fully empty, 6 mL of acetonitrile were loaded into the reaction flask and pushed into the reactor to collect all the product. Finally, the solvent was evaporated under reduced pressure. The crude was suspended in n-pentane and sonicated for two minutes. The surnatant was filtered through a plug of celite to retain solid traces. This process was repeated three times. Finally, the celite plug was washed three times with CH_2Cl_2. The CH_2Cl_2 phases were collected and added to the residual solid. The corresponding solution was then diluted with $NaHCO_3$ (~ 1:1 ratio organic:water phase) and the biphasic mixture was stirred for 15 minutes at rt. The solution was then transferred to a separatory funnel and extracted three times with CH_2Cl_2. The combined organic layers were dried over Na_2SO_4, filtered and the solvent was removed under reduced pressure. The residue was purified via flash column chromatography on silica gel (Pentane:Ethyl Acetate 3:1) to afford the product as a colorless oil (1.38 g, 70% yield).

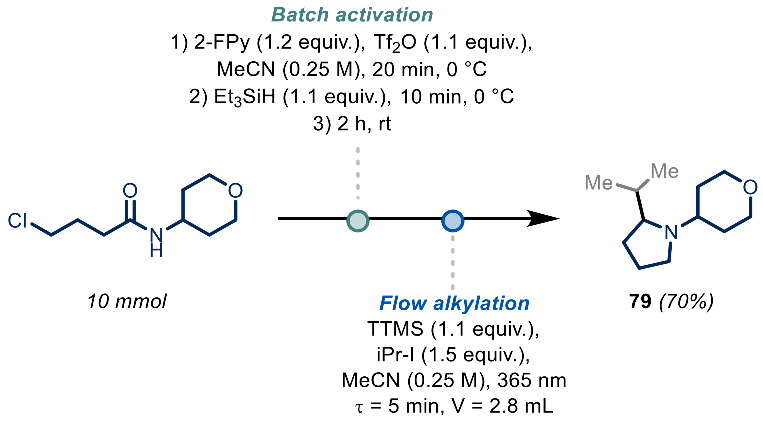

2-isopropyl-1-(tetrahydro-2H-pyran-4-yl)pyrrolidine **(79).**

^{1}H NMR (300 MHz, $CDCl_3$) δ 4.05 – 3.92 (m, 2H), 3.45 – 3.24 (m, 2H), 2.94 – 2.81 (m, 1H), 2.76 – 2.44 (m, 3H), 1.79 – 1.45 (m, 9H), 0.84 (d, *J* = 6.9 Hz, 3H), 0.80 (d, *J* = 6.8 Hz, 3H).

^{13}C NMR (75 MHz, $CDCl_3$) δ 68.3, 67.7, 64.5, 56.2, 48.0, 33.3, 30.3, 26.3, 25.4, 23.7, 20.5, 16.0.

HRMS (ESI+) (m/z): $[M+H]^+$ calcd. for $C_{12}H_{23}NO$, 198.1858; found: 198.1853.

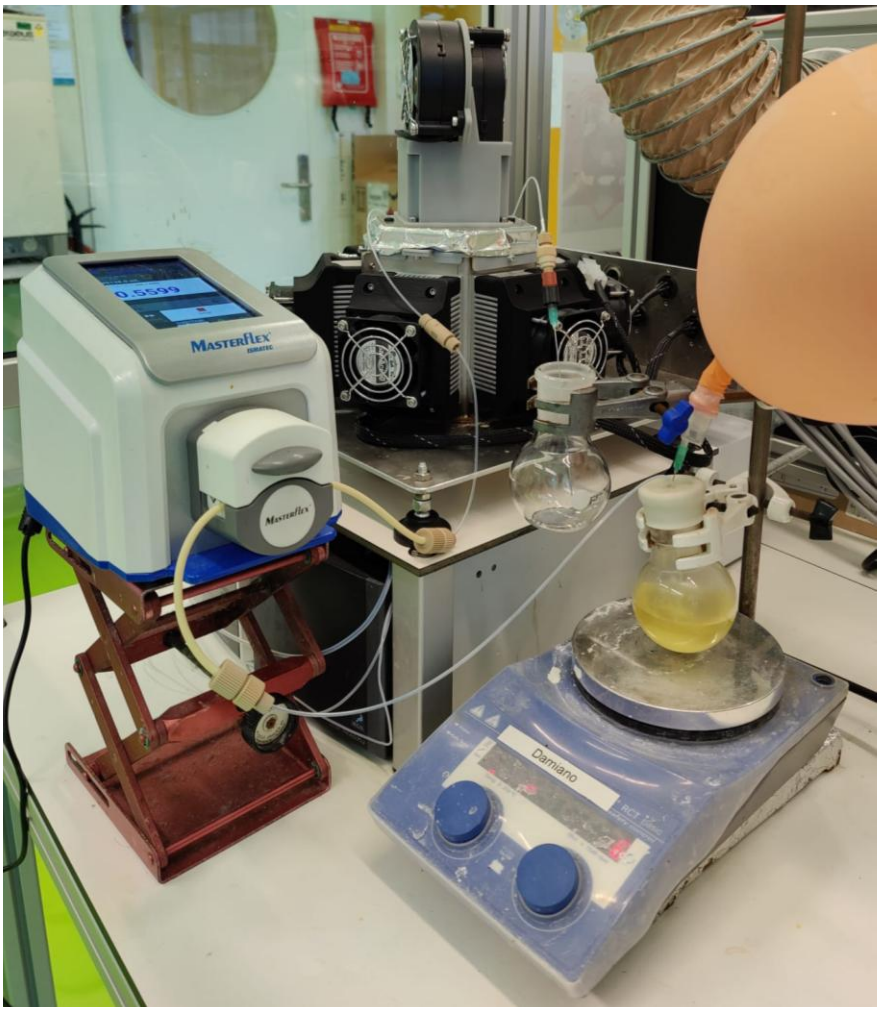

Supplementary Figure 15. Flow set-up for the 10 mmol scale of **5** and **79** at 0.25 M.

11. Characterization data of synthesized compound

11.1 Characterization of newly synthesized secondary amides

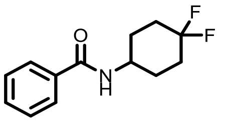

***N-(4,4-difluorocyclohexyl)benzamide* (1d).** Prepared according to GP1 from 4,4-difluorocyclohexan-1-amine (338 µL, 2.75 mmol, 1.1 equiv.) to afford the product as a white solid (478 mg, 80% yield).

^{1}H NMR (400 MHz, $CDCl_3$) δ 7.81 – 7.71 (m, 2H), 7.55 – 7.46 (m, 1H), 7.45 – 7.39 (m, 2H), 6.06 (d, *J* = 7.6 Hz, 1H), 4.13 – 4.02 (m, 1H), 2.20 – 2.01 (m, 4H), 1.97 – 1.79 (m, 2H), 1.69 – 1.60 (m, 2H).
^{13}C NMR (101 MHz, $CDCl_3$) δ 167.2, 134.6, 131.7, 128.8, 126.7, 46.9, 32.4 (t, *J* = 24.8 Hz), 28.9, 28.8.
^{19}F NMR (282 MHz, $CDCl_3$) δ -94.76 (d, *J* = 237.5 Hz), -101.48 (d, *J* = 237.8 Hz).
HRMS (GC-FI+) (m/z): $[M+H]^+$ calcd. for $C_{13}H_{15}F_2NO$, 240.1200; found: 240.1207.

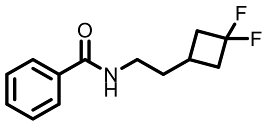

N-(2-(3,3-difluorocyclobutyl)ethyl)benzamide (**1o**). Prepared according to GP1 from 2-(3,3-difluorocyclobutyl)ethan-1-amine hydrochloride (283 mg, 1.65 mmol, 1.1 equiv.) and triethylamine (437 µl, 3.15 mmol, 2.1 equiv.). Isolated as a white solid (264 mg, 74% yield).

^{1}H NMR (400 MHz, $CDCl_3$) δ 7.77 – 7.71 (m, 2H), 7.53 – 7.47 (m, 1H), 7.46 – 7.40 (m, 2H), 6.16 (s, 1H), 3.44 (ddd, *J* = 8.0, 7.2, 6.0 Hz, 2H), 2.80 – 2.63 (m, 2H), 2.32 – 2.13 (m, 3H), 1.88 – 1.77 (m, 2H).
^{13}C NMR (101 MHz, $CDCl_3$) δ 167.8, 134.6, 131.7, 128.7, 126.9, 120.3 (dd, *J* = 283.9, 274.6 Hz), 40.7 (dd, *J* = 22.9, 21.4 Hz), 38.4, 36.0 (dd, *J* = 3.1 Hz), 21.2 (dd, *J* = 12.4, 6.3 Hz).
^{19}F NMR (377 MHz, $CDCl_3$) δ -82.39 (d, *J* = 193.3 Hz), -95.05 (d, *J* = 193.3 Hz).
HRMS (ESI+) (m/z): $[M+H]^+$ calcd. for $C_{13}H_{15}F_2NO$, 240.1200; found: 240.1195.

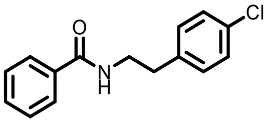

***N-(4-chlorophenethyl)benzamide* (1p).** Prepared according to GP1 from 2-(4-chlorophenyl)ethan-1-amine (384 µL, 2.75 mmol, 1.1 equiv.) to afford the product as a white solid (519 mg, 80%).

^{1}H NMR (400 MHz, $CDCl_3$) δ 7.73 – 7.64 (m, 2H), 7.55 – 7.46 (m, 1H), 7.44 – 7.37 (m, 2H), 7.31 – 7.22 (m, 2H), 7.17 (d, *J* = 8.4 Hz, 2H), 6.15 (s, 1H), 3.72 – 3.65 (m, 2H), 2.91 (t, *J* = 6.9 Hz, 2H).
^{13}C NMR (101 MHz, $CDCl_3$) δ 167.6, 137.5, 134.6, 132.6, 131.7, 130.3, 129.0, 128.8, 126.9, 41.2, 35.2.
HRMS (ESI+) (m/z): $[M+H]^+$ calcd. for $C_{15}H_{14}ClNO$, 260.0842; found: 260.0847.

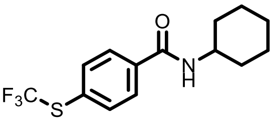

***N-cyclohexyl-4-((trifluoromethyl)thio)benzamide* (2c).** Prepared according to GP2 from 4-((trifluoromethyl)thio)benzoic acid (555 mg, 2.5 mmol, 1.0 equiv.) to afford the product as a white solid (531 mg, 70% yield).

^{1}H NMR (400 MHz, $CDCl_3$) δ 7.78 (d, *J* = 8.5 Hz, 2H), 7.69 (d, *J* = 8.5 Hz, 2H), 6.05 (d, *J* = 8.1 Hz, 1H), 4.12 – 3.80 (m, 1H), 2.07 – 1.94 (m, 2H), 1.76 (dt, *J* = 13.5, 3.9 Hz, 2H), 1.66 (dt, *J* = 12.8, 3.8 Hz, 1H), 1.48 – 1.35 (m, 2H), 1.29 – 1.12 (m, 3H).

^{13}**C NMR** (101 MHz, $CDCl_3$) δ 165.6, 137.5, 136.2, 132.6 (q, *J* = 308.3 Hz), 128.0, 127.9 (q, *J* = 2.3 Hz), 49.1, 33.3, 25.6, 25.0.
^{19}**F NMR** (282 MHz, $CDCl_3$) δ -42.2.
HRMS (ESI+) (m/z): $[M+H]^+$ calcd. for $C_{14}H_{16}F_3NOS$, 304.0983; found: 304.0987

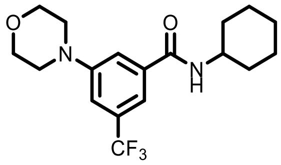

***N*-cyclohexyl-3-morpholino-5-(trifluoromethyl)benzamide** (**2h**). Prepared according to GP2 from 3-morpholino-5-(trifluoromethyl)benzoic acid (550 mg, 2.0 mmol, 1.0 equiv.). *Alternative work-up:* The reaction was diluted with CH_2Cl_2 and washed with water (2x) and a saturated aqueous Na_2CO_3 solution. The organic layers were dried over Na_2SO_4, filtered and the solvent was removed under reduced pressure to afford the product as a white solid (372 mg, 52% yield).

^{1}**H NMR** (400 MHz, $CDCl_3$) δ 7.52 (t, *J* = 1.9 Hz, 1H), 7.30 – 7.26 (m, 1H), 7.17 (t, *J* = 1.9 Hz, 1H), 5.97 (d, *J* = 8.1 Hz, 1H), 3.96 (dddd, *J* = 14.8, 10.7, 8.0, 4.0 Hz, 1H), 3.89 – 3.83 (m, 4H), 3.29 – 3.22 (m, 4H), 2.03 (dt, *J* = 12.2, 3.9 Hz, 2H), 1.83 – 1.72 (m, 2H), 1.72 – 1.62 (m, 1H), 1.51 – 1.34 (m, 2H), 1.34 – 1.13 (m, 3H).
^{13}**C NMR** (101 MHz, $CDCl_3$) δ 165.8, 151.8, 137.1, 131.8 (d, *J* = 32.1 Hz), 124.0 (q, *J* = 272.7 Hz), 117.5, 114.1 (q, *J* = 3.8 Hz), 113.3 (q, *J* = 4.0 Hz), 66.7, 49.2, 48.6, 33.3, 25.7, 25.1.
^{19}**F NMR** (377 MHz, $CDCl_3$) δ -62.7.
HRMS (ESI+) (m/z): $[M+H]^+$ calcd. for $C_{18}H_{23}F_3N_2O_2$, 357.1790; found: 357.1793.

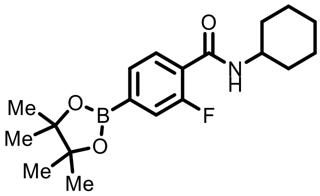

***N*-cyclohexyl-2-fluoro-4-(4,4,5,5-tetramethyl-1,3,2-dioxaborolan-2-yl)benzamide** (**2i**). Prepared according to GP2 from 2-fluoro-4-(4,4,5,5-tetramethyl-1,3,2-dioxaborolan-2-yl)benzoic acid (532 mg, 2.0 mmol, 1.0 equiv.). *Alternative work-up:* The reaction was diluted with CH_2Cl_2 and washed with water (3x). The organic layers were dried over Na_2SO_4, filtered and the solvent was removed under reduced pressure to afford the product as a pale yellow solid (470 mg, 67% yield).

^{1}**H NMR** (400 MHz, $CDCl_3$) δ 8.06 (t, *J* = 7.7 Hz, 1H), 7.68 – 7.62 (m, 1H), 7.54 – 7.46 (m, 1H), 6.72 – 6.58 (m, 1H), 4.13 – 3.92 (m, 1H), 2.07 – 1.97 (m, 2H), 1.79 – 1.69 (m, 2H), 1.68 – 1.58 (m, 1H), 1.51 – 1.37 (m, 2H), 1.35 (s, 12H), 1.33 – 1.17 (m, 3H).
^{13}**C NMR** (101 MHz, $CDCl_3$) δ 162.3 (d, *J* = 3.3 Hz), 160.3 (d, *J* = 247.3 Hz), 131.5 (d, *J* = 1.7 Hz), 130.9 (d, *J* = 3.1 Hz), 123.7 (d, *J* = 11.6 Hz), 121.8 (d, *J* = 23.3 Hz), 84.5, 48.8, 33.1, 25.7, 25.0, 24.9.
^{11}**B NMR** (128 MHz, $CDCl_3$) δ 30.41.
HRMS (ESI+) (m/z): $[M+H]^+$ calcd. for $C_{19}H_{27}BFNO_3$, 348.2150; found: 348.2158.

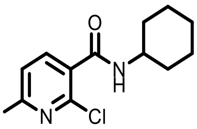

2,6-dichloro-N-cyclohexylnicotinamide (**2j**)**.** Prepared according to GP2 from 2,6-dichloronicotinic acid (480 mg, 2.50 mmol, 1.0 equiv.) to afford the product as a white solid (440 mg, 64% yield).

^{1}**H NMR** (400 MHz, $CDCl_3$) δ 8.08 (d, *J* = 8.1 Hz, 1H), 7.36 (d, *J* = 8.0 Hz, 1H), 6.34 (s, 1H), 4.08 – 3.94 (m, 1H), 2.07 – 1.98 (m, 2H), 1.80 – 1.70 (m, 2H), 1.68 – 1.60 (m, 1H), 1.50 – 1.38 (m, 2H), 1.35 – 1.19 (m, 3H).
^{13}**C NMR** (101 MHz, $CDCl_3$) δ 162.8, 151.6, 146.3, 142.2, 130.4, 123.6, 49.4, 32.8, 25.6, 24.8.
HRMS (ESI+) (m/z): $[M+H]^+$ calcd. for $C_{12}H_{14}Cl_2N_2O$, 273.0565; found: 273.0561.

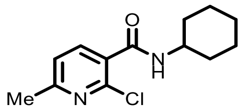

***2-chloro-N-cyclohexyl-6-methylnicotinamide* (2k).** Prepared according to GP2 from 2-chloro-6-methylnicotinic acid (429 mg, 2.5 mmol, 1.0 equiv.) to afford the product as a white solid (493 mg, 78% yield).

^{1}H NMR (400 MHz, $CDCl_3$) δ 8.02 (d, *J* = 7.8 Hz, 1H), 7.23 (d, *J* = 7.8 Hz, 1H), 6.43 (s, 1H), 4.02 (d, *J* = 10.5 Hz, 1H), 2.54 (s, 3H), 2.05 (dd, *J* = 12.8, 4.4 Hz, 2H), 1.7 (dt, *J* = 13.3, 4.2 Hz, 2H), 1.66 – 1.57 (m, 1H), 1.49 – 1.34 (m, 2H), 1.33 – 1.15 (m, 3H).
^{13}C NMR (101 MHz, $CDCl_3$) δ 163.9, 161.1, 146.1, 140.3, 128.5, 122.5, 49.2, 32.9, 25.6, 24.8, 24.2.
HRMS (ESI+) (m/z): $[M+H]^+$ calcd. for $C_{13}H_{17}ClN_2O$, 253.1108; found: 253.1101.

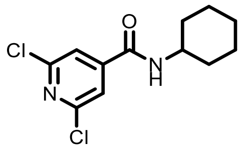

***2,6-dichloro-N-cyclohexylisonicotinamide* (2l).** Prepared according to GP2 from 2,6-dichloroisonicotinic acid (480 mg, 2.5 mmol, 1.0 equiv.) to afford the product as a white solid (567 mg, 83% yield).

^{1}H NMR (400 MHz, $CDCl_3$) δ 7.55 (s, 2H), 6.06 (s, 1H), 3.99 – 3.82 (m, 1H), 2.04 – 1.96 (m, 2H), 1.77 (dt, *J* = 13.5, 3.8 Hz, 2H), 1.68 – 1.60 (m, 1H), 1.47 – 1.34 (m, 2H), 1.33 – 1.13 (m, 3H).
^{13}C NMR (101 MHz, $CDCl_3$) δ 162.3, 151.5, 147.6, 120.8, 49.6, 33.1, 25.5, 24.9.
HRMS (ESI+) (m/z): $[M+H]^+$ calcd. for $C_{12}H_{14}Cl_2N_2O$, 273.0565; found: 273.0562.

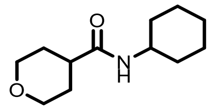

***N-cyclohexyltetrahydro-2H-pyran-4-carboxamide* (2n).** Prepared according to GP2 from tetrahydro-2H-pyran-4-carboxylic acid (325 mg, 2.5 mmol, 1.0 equiv.) to afford the product as a white solid (453 mg, 86% yield).

^{1}H NMR (400 MHz, $CDCl_3$) δ 5.56 (d, *J* = 8.2 Hz, 1H), 4.01 – 3.9 (m, 2H), 3.81 – 3.63 (m, 1H), 3.35 (td, *J* = 11.5, 2.6 Hz, 2H), 2.26 (tt, *J* = 11.1, 4.3 Hz, 1H), 1.89 – 1.82 (m, 2H), 1.81 – 1.62 (m, 6H), 1.59 – 1.54 (m, 1H), 1.37 – 1.23 (m, 2H), 1.18 – 1.01 (m, 3H).
^{13}C NMR (101 MHz, $CDCl_3$) δ 173.4, 67.3, 48.0, 42.3, 33.2, 29.4, 25.5, 24.9.
HRMS (ESI+) (m/z): $[M+H]^+$ calcd. for $C_{12}H_{21}NO_2$, 212.1651; found: 212.1653.

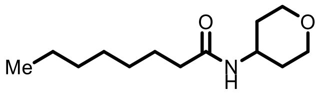

***N-(tetrahydro-2H-pyran-4-yl)octanamide* (2p).** Prepared according to GP2 from octanoic acid (721 mg, 792 mL, 5.00 mmol, 1.0 equiv.) to afford the product as a white solid (910 mg, 80% yield).

^{1}H NMR (400 MHz, $CDCl_3$) δ 5.44 – 5.31 (m, 1H), 4.09 – 3.88 (m, 3H), 3.46 (td, *J* = 11.7, 2.2 Hz, 2H), 2.14 (t, *J* = 7.8 Hz, 2H), 1.92 – 1.83 (m, 2H), 1.66 – 1.56 (m, 2H), 1.44 (qd, *J* = 12.6, 12.0, 4.4 Hz, 2H), 1.35 – 1.21 (m, 8H), 0.87 (t, *J* = 6.5 Hz, 3H).
^{13}C NMR (101 MHz, $CDCl_3$) δ 172.6, 66.9, 45.6, 37.1, 33.4, 31.8, 29.3, 29.1, 25.9, 22.7, 14.2.
HRMS (GC-FI+) (m/z): $[M+H]^+$ calcd. for $C_{13}H_{25}NO_2$, 288.1964; found: 288.1961.

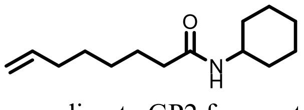

***N-cyclohexyloct-7-enamide* (2q).** Prepared according to GP2 from oct-7-enoic acid (711 mg, 769 mL, 5.00 mmol, 1.0 equiv.) to afford the product as a white solid (890 mg, 80% yield).
^{1}H NMR (400 MHz, $CDCl_3$) δ 5.78 (ddt, *J* = 16.9, 10.1, 6.6 Hz, 1H), 5.31 (s, 1H), 5.02 – 4.95 (m, 1H), 4.95 – 4.89 (m, 1H), 3.83 – 3.69 (m, 1H), 2.13 (t, *J* = 7.6 Hz, 2H), 2.08 – 1.99 (m, 2H), 1.95 – 1.86 (m, 2H), 1.74 – 1.65 (m, 2H), 1.65 – 1.56 (m, 3H), 1.43 – 1.28 (m, 6H), 1.20 – 1.06 (m, 3H).

^{13}C NMR (101 MHz, $CDCl_3$) δ 172.2, 139.0, 114.5, 48.2, 37.1, 33.7, 33.4, 28.8, 28.7, 25.8, 25.7, 25.0.
HRMS (GC-FI+) (m/z): $[M+H]^+$ calcd. for $C_{14}H_{25}NO$, 224.2014; found: 224.2016.

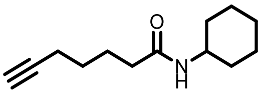

N-cyclohexylhept-6-ynamide **(2r).** Prepared according to GP2 from hept-6-ynoic acid (701 mg, 717 mL, 5.00 mmol, 90% Wt, 1.0 equiv.) to afford the product as a pale brown solid (1.04 g, quantitative).

^{1}H NMR (400 MHz, $CDCl_3$ δ 5.33 (br s, 1H), 3.83 – 3.67 (m, 1H), 2.26 – 2.11 (m, 4H), 1.96 – 1.88 (m, 3H), 1.80 – 1.66 (m, 4H), 1.64 – 1.51 (m, 3H), 1.42 – 1.30 (m, 2H), 1.21 – 1.03 (m, 3H).
^{13}C NMR (101 MHz, $CDCl_3$) δ 171.7, 84.3, 68.7, 48.3, 36.5, 33.4, 28.1, 25.7, 25.1, 25.0, 18.4.
HRMS (GC-FI+) (m/z): $[M+H]^+$ calcd. for $C_{13}H_{21}NO$, 208.1701; found: 208.1711.

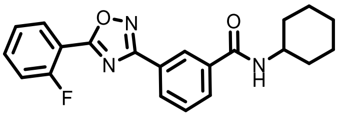

N-cyclohexyl-3-(5-(2-fluorophenyl)-1,2,4-oxadiazol-3-yl)benzamide **(2t).** Prepared according to GP2 from 3-[5-(2-fluorophenyl)-1,2,4-oxadiazol-3-yl]-benzoic acid (284 mg, 1.00 mmol) to afford the product as a pale brown solid (256 mg, 70%).

^{1}H NMR (400 MHz, $CDCl_3$) δ 8.45 (s, 1H), 8.29 (d, *J* = 7.8 Hz, 1H), 8.22 (t, *J* = 7.6 Hz, 1H), 8.00 (d, *J* = 7.8 Hz, 1H), 7.65 – 7.52 (m, 2H), 7.39 – 7.28 (m, 2H), 6.14 (d, *J* = 8.1 Hz, 1H), 4.06 – 3.91 (m, 1H), 2.07 (d, *J* = 12.1 Hz, 2H), 1.83 – 1.75 (m, 2H), 1.74 – 1.63 (m, 1H), 1.50 – 1.36 (m, 2H), 1.34 – 1.16 (m, 3H).
^{13}C NMR (101 MHz, $CDCl_3$) δ 173.2 (d, *J* = 4.4 Hz), 168.3, 165.9, 160.9 (d, *J* = 260.8 Hz), 136.1, 134.9 (d, *J* = 8.7 Hz), 131.1, 130.5, 130.4, 129.5, 127.1, 125.4, 124.9 (d, *J* = 3.9 Hz), 117.3 (d, *J* = 20.9 Hz), 112.8 (d, *J* = 11.5 Hz), 49.1, 33.4, 25.7, 25.1.
^{19}F NMR (282 MHz, $CDCl_3$) δ -108.18.
HRMS (ESI+) (m/z): $[M+H]^+$ calcd. for $C_{21}H_{20}FN_3O_2$, 366.1618; found: 366.1618.

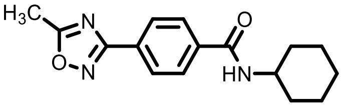

N-cyclohexyl-4-(5-methyl-1,2,4-oxadiazol-3-yl)benzamide (**2u**). Prepared according to GP2 from 4-(5-methyl-1,2,4-oxadiazol-3-yl)benzoic acid (408 mg, 2.0 mmol, 1.0 equiv.).
N.B. For the work-up HCl 0.2 M instead of 1.0 M was used. Isolated as a pale brown solid (413 mg, 72% yield).

^{1}H NMR (400 MHz, $CDCl_3$) δ 8.15 – 8.08 (m, 2H), 7.92 – 7.82 (m, 2H), 6.03 (d, *J* = 8.0 Hz, 1H), 3.99 (ddt, *J* = 14.8, 10.8, 5.6 Hz, 1H), 2.67 (s, 3H), 2.08 – 2.00 (m, 2H), 1.81 – 1.72 (m, 2H), 1.72 – 1.65 (m, 1H), 1.51 – 1.36 (m, 2H), 1.32 – 1.14 (m, 3H).
^{13}C NMR (101 MHz, $CDCl_3$) δ 177.0, 167.9, 165.9, 137.5, 129.5, 127.6, 127.5, 48.9, 33.3, 25.7, 25.1, 12.5.
HRMS (ESI+) (m/z): $[M+H]^+$ calcd. for $C_{16}H_{19}N_3O_2$, 286.1556; found: 286.1567.

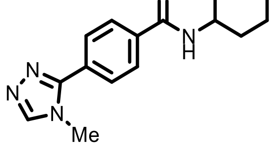

N-cyclohexyl-4-(4-methyl-4H-1,2,4-triazol-3-yl)benzamide (**2v**). Prepared according to GP2 from 4-(4-methyl-4H-1,2,4-triazol-3-yl)benzoic acid (305 mg, 1.50 mmol, 1.0 equiv.).
N.B. For the work-up HCl 0.2 M instead of 1.0 M was used. Isolated as a pale yellow solid (211 mg, 50% yield).

^{1}H NMR (400 MHz, $CDCl_3$) δ 8.18 (s, 1H), 7.89 – 7.81 (m, 2H), 7.70 – 7.63 (m, 2H), 6.45 (d, *J* = 8.1 Hz, 1H), 4.04 – 3.91 (m, 1H), 3.75 (s, 3H), 2.08 – 1.97 (m, 2H), 1.76 (dt, *J* = 13.2, 3.6 Hz, 2H), 1.69 – 1.61 (m, 1H), 1.47 – 1.35 (m, 2H), 1.34 – 1.19 (m, 3H).
^{13}C NMR (101 MHz, $CDCl_3$) δ 165.9, 153.6, 145.6, 136.8, 129.1, 128.7, 127.7, 49.1, 33.2, 32.4, 25.6, 25.1.

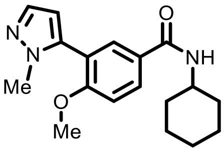

***N*-cyclohexyl-4-methoxy-3-(1-methyl-1H-pyrazol-5-yl)benzamide** (**2z**). Prepared according to GP2 from 4-methoxy-3-(1-methyl-1H-pyrazol-5-yl)benzoic acid (348 mg, 1.50 mmol, 1.0 equiv.).
N.B. For the work-up HCl 0.2 M instead of 1.0 M was used. The crude solid was triturated in a 20:1 heptane:ethyl acetate mixture and filtered to isolate the product as a white solid (302 mg, 64% yield).

^{1}H NMR (400 MHz, $CDCl_3$) δ 7.91 – 7.84 (m, 1H), 7.65 – 7.60 (m, 1H), 7.60 – 7.50 (m, 1H), 7.01 (d, *J* = 8.7 Hz, 1H), 6.29 – 6.24 (m, 1H), 5.93 (d, *J* = 7.9 Hz, 1H), 4.02 – 3.93 (m, 1H), 3.87 (s, 3H), 3.70 (s, 3H), 2.07 – 1.98 (m, 2H), 1.80 – 1.70 (m, 3H), 1.70 – 1.60 (m, 1H), 1.42 (q, *J* = 12.5 Hz, 2H), 1.23 – 1.12 (m, 2H).
^{13}C NMR (101 MHz, $CDCl_3$) δ 165.6, 159.4, 139.5, 138.5, 130.4, 130.0, 127.6, 119.9, 110.9, 107.1, 55.9, 48.9, 37.3, 33.4, 25.7, 25.1.
HRMS (ESI+) (m/z): $[M+H]^+$ calcd. for $C_{18}H_{23}N_3O_2$, 314.1869; found: 314.1868.

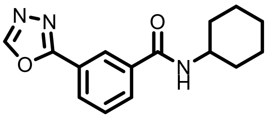

***N*-cyclohexyl-3-(1,3,4-oxadiazol-2-yl)benzamide** (**2aa**). Prepared according to GP2 from 3-(1,3,4-oxadiazol-2-yl)benzoic acid (285 mg, 1.50 mmol, 1.0 equiv.).
N.B. For the work-up HCl 0.2 M instead of 1.0 M was used. Isolated as a white solid (346 mg, 85% yield).

^{1}H NMR (400 MHz, $CDCl_3$) δ 8.50 (s, 1H), 8.43 (td, *J* = 1.8, 0.6 Hz, 1H), 8.19 (ddd, *J* = 7.8, 1.7, 1.2 Hz, 1H), 8.00 (ddd, *J* = 7.8, 1.8, 1.2 Hz, 1H), 7.61 (td, *J* = 7.8, 0.6 Hz, 1H), 6.07 (d, *J* = 7.8 Hz, 1H), 4.01 (dddd, *J* = 14.7, 10.7, 7.9, 3.9 Hz, 1H), 2.10 – 2.02 (m, 2H), 1.83 – 1.74 (m, 2H), 1.73 – 1.63 (m, 1H), 1.54 – 1.37 (m, 2H), 1.34 – 1.15 (m, 3H).
^{13}C NMR (101 MHz, $CDCl_3$) δ 165.3, 164.3, 153.0, 136.3, 130.9, 129.7, 129.7, 125.3, 123.9, 49.2, 33.3, 25.7, 25.1.
HRMS (ESI+) (m/z): $[M+H]^+$ calcd. for $C_{15}H_{17}N_3O_2$, 272.1399; found: 272.1400.

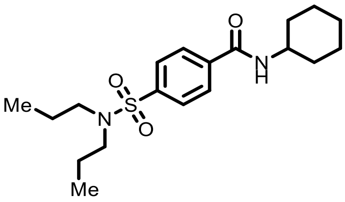

***N*-cyclohexyl-4-(*N,N*-dipropylsulfamoyl)benzamide (2ad).** Prepared according to GP2 from probenecid acid (428 mg, 1.50 mmol, 1.0 equiv.) to afford the product as white solid (363 mg, 66% yield).

^{1}H NMR (400 MHz, $CDCl_3$) δ 7.84 – 7.79 (m, 2H), 7.73 (d, *J* = 8.5 Hz, 2H), 6.42 (d, *J* = 8.0 Hz, 1H), 3.99 – 3.85 (m, 1H), 3.09 – 2.97 (m, 4H), 2.06 – 1.92 (m, 2H), 1.74 (dt, *J* = 13.3, 3.8 Hz, 2H), 1.66 – 1.58 (m, 1H), 1.55 – 1.44 (m, 4H), 1.41 – 1.09 (m, 5H), 0.83 (t, *J* = 7.4 Hz, 6H).
^{13}C NMR (101 MHz, $CDCl_3$) δ 165.5, 142.4, 138.8, 127.8, 127.1, 50.0, 49.2, 33.1, 25.6, 25.0, 22.0, 11.2.
HRMS (ESI+) (m/z): $[M+H]^+$ calcd. for $C_{19}H_{30}N_2O_3S$, 367.2055; found: 367.2050.

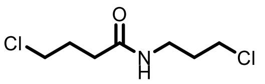

4-chloro-N-(3-chloropropyl)butanamide **(3b).** Prepared according to GP3 from 3-chloropropylamine hydrochloride (357 mg, 2.75 mmol, 1.1 equiv.) and 4-chlorobutanoyl chloride (280 µL, 2.5 mmol, 1.0 equiv.) to afford the product as a pale brown solid (436 mg, 88%).

^{1}H NMR (400 MHz, $CDCl_3$) (400 MHz, $CDCl_3$) δ 5.89 (s, 1H), 3.67 – 3.50 (m, 4H), 3.49 – 3.34 (m, 2H), 2.36 (t, J = 7.0 Hz, 2H), 2.11 (q, J = 5.8 Hz, 2H), 2.05 – 1.93 (m, 2H).
^{13}C NMR (101 MHz, $CDCl_3$) δ 172.2, 44.6, 42.61, 37.1, 33.2, 32.2, 28.2.

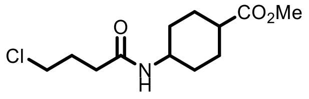

methyl 4-(4-chlorobutanamido)cyclohexane-1-carboxylate **(3d).** Prepared according to GP3 from methyl trans-4-aminocyclohexanecarboxylate hydrochloride (533 mg, 2.75 mmol, 1.1 equiv.) and 4-chlorobutanoyl chloride (280 µL, 2.5 mmol, 1.0 equiv.) to afford the product as a white solid (510 mg, 78%).

^{1}H NMR (400 MHz, $CDCl_3$) δ 5.38 – 5.31 (m, 1H), 3.78 – 3.70 (m, 1H), 3.66 (s, 3H), 3.60 (t, J = 6.1 Hz, 2H), 2.32 (t, J = 7.1 Hz, 2H), 2.28 – 2.16 (m, 1H), 2.15 – 1.96 (m, 6H), 1.62 – 1.47 (m, 2H), 1.25 – 0.94 (m, 2H).
^{13}C NMR (101 MHz, $CDCl_3$) δ 175.8, 171.1, 51.8, 47.9, 44.7, 42.5, 33.5, 32.3, 28.3, 27.8.
HRMS (ESI+) (m/z): $[M+H]^+$ calcd. for $C_{12}H_{20}ClNO_3$, 262.1210; found: 262.1216.

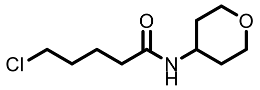

5-chloro-N-(tetrahydro-2H-pyran-4-yl)pentanamide **(3g).** Prepared according to GP3 from 4-aminotetrahydropyran (285 µL, 2.75 mmol, 1.1 equiv.) and 5-chlorovaleroyl chloride (323 µL, 2.5 mmol, 1.0 equiv.) to afford the product as a white solid (401 mg, 74%).

^{1}H NMR (400 MHz, $CDCl_3$) δ 5.58 – 5.47 (m, 1H), 4.04 – 3.88 (m, 3H), 3.56 – 3.49 (m, 2H), 3.44 (td, J = 11.7, 2.1 Hz, 2H), 2.27 – 2.13 (m, 2H), 1.86 (ddd, J = 12.6, 4.4, 2.2 Hz, 2H), 1.79 (s, 4H), 1.51 – 1.33 (m, 2H).
^{13}C NMR (101 MHz, $CDCl_3$) δ 171.7, 66.9, 45.7, 44.7, 35.9, 33.3, 32.0, 23.1.
HRMS (ESI+) (m/z): $[M+H]^+$ calcd. for $C_{10}H_{18}ClNO_2$, 220.1104; found: 220.1103.

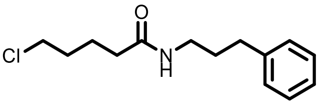

5-chloro-N-(3-phenylpropyl)pentanamide **(3h).** Prepared according to GP3 from 3-phenylpropan-1-amine (285 µL, 2.75 mmol, 1.1 equiv.) and 5-chlorovaleroyl chloride (391 µL, 2.5 mmol, 1.0 equiv.) to afford the product as a colorless oil (495 mg, 78%).

^{1}H NMR (300 MHz, $CDCl_3$) δ 7.36 – 7.23 (m, 2H), 7.23 – 7.14 (m, 3H), 5.47 – 5.29 (m, 1H), 3.54 (t, J = 6.2 Hz, 2H), 3.34 – 3.25 (m, 2H), 2.69 – 2.62 (m, 2H), 2.20 – 2.11 (m, 2H), 1.92 – 1.65 (m, 6H).
^{13}C NMR (101 MHz, $CDCl_3$) δ 172.5, 141.5, 128.5, 128.4, 126.1, 44.7, 39.3, 35.7, 33.4, 32.0, 31.2, 23.1.
HRMS (ESI+) (m/z): $[M+H]^+$ calcd. for $C_{14}H_{20}ClN$, 254.1312; found: 254.1316.

11.2 Characterization of compounds 5-42 (*sec*-amides varying amine or carboxylic acid)

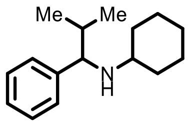

***N-(2-methyl-1-phenylpropyl)cyclohexanamine* (5).** Prepared according to GP5 from **1a** (51 mg, 0.25 mmol, 1.0 equiv.). Purified via flash column chromatography on silica gel (Pentane:Ethyl Acetate 20:1) to afford the product as a colorless oil (45 mg, 78% yield).

Characterization data are in accordance with literature.[10]
^{1}H NMR (400 MHz, $CDCl_3$) δ 7.34 – 7.28 (m, 2H), 7.28 – 7.20 (m, 3H), 3.47 (d, *J* = 6.9 Hz, 1H), 2.20 (tt, *J* = 9.8, 3.5 Hz, 1H), 2.01 – 1.93 (m, 1H), 1.88 – 1.75 (m, *J* = 6.8 Hz, 1H), 1.73 – 1.59 (m, 3H), 1.58 – 1.49 (m, 1H), 1.31 – 1.21 (m, 1H), 1.19 – 0.99 (m, 5H), 0.96 (d, *J* = 6.7 Hz, 3H), 0.74 (d, *J* = 6.8 Hz, 3H).
^{13}C NMR (101 MHz, $CDCl_3$) δ 144.1, 128.0, 128.0, 126.6, 66.0, 53.7, 35.1, 34.8, 33.0, 26.4, 25.3, 24.9, 19.9, 19.7.

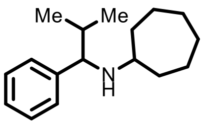

***N-(2-methyl-1-phenylpropyl)cycloheptanamine* (6).** Prepared according to GP5 from **1b** (54 mg, 0.25 mmol, 1.0 equiv.). Purified via flash column chromatography on silica gel (Pentane:Ethyl Acetate 20:1, 0.5 vol% triethylamine) to afford the product as a colorless oil (34 mg, 55% yield).

^{1}H NMR (400 MHz, $CDCl_3$) 7.35 – 7.27 (m, 2H), 7.24 – 7.17 (m, 3H), 3.38 (d, *J* = 7.0 Hz, 1H), 2.56 – 2.18 (m, 1H), 2.00 – 1.73 (m, 2H), 1.72 – 1.13 (m, 12H), 0.96 (d, *J* = 6.7 Hz, 3H), 0.73 (d, *J* = 6.7 Hz, 3H).
^{13}C NMR (101 MHz, $CDCl_3$) δ 143.8, 128.0, 128.0, 126.7, 66.5, 55.7, 36.8, 34.8, 33.2, 28.8, 28.4, 24.5, 23.9, 19.9, 19.8.
HRMS (GC-FI+) (m/z): $[M+H]^+$ calcd. for $C_{17}H_{27}N$, 246.2222; found: 246.2230.

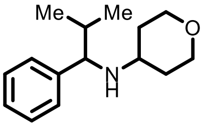

***N-(2-methyl-1-phenylpropyl)tetrahydro-2H-pyran-4-amine* (7).** Prepared according to GP5 from **1c** (51 mg, 0.25 mmol, 1.0 equiv.). Purified via flash column chromatography on silica gel (Pentane:Ethyl Acetate 10:1, 0.5 vol% triethylamine) to afford the product as a colorless oil (37 mg, 64% yield).

^{1}H NMR (400 MHz, $CDCl_3$) δ 7.36 – 7.27 (m, 2H), 7.26 – 7.20 (m, 3H), 4.03 – 3.79 (m, 2H), 3.47 (d, *J* = 6.9 Hz, 1H), 3.25 (qd, *J* = 11.6, 2.4 Hz, 2H), 2.55 – 2.27 (m, 1H), 1.99 – 1.85 (m, 1H), 1.85 – 1.71 (m, 1H), 1.69 – 1.51 (m, 1H), 1.45 – 1.21 (m, 3H), 0.96 (d, *J* = 6.6 Hz, 3H), 0.73 (d, *J* = 6.7 Hz, 3H).
^{13}C NMR (101 MHz, $CDCl_3$) δ 143.6, 128.1, 128.0, 126.8, 67.1, 66.8, 65.7, 51.0, 34.9, 34.8, 33.4, 19.8, 19.7.
HRMS (GC-FI+) (m/z): $[M+H]^+$ calcd. for $C_{15}H_{23}NO$, 234.1858; found: 234.1856.

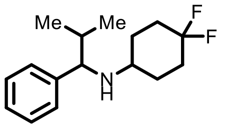

***4,4-difluoro-N-(2-methyl-1-phenylpropyl)cyclohexan-1-amine* (8).** Prepared according to GP5 from **1d** (60 mg, 0.25 mmol, 1.0 equiv.). Purified via flash column chromatography on silica gel (Pentane:Ethyl Acetate 20:1, 0.5 vol% triethylamine) to afford the product as a colorless oil (47 mg, 70% yield).

^{1}H NMR (400 MHz, $CDCl_3$) δ 7.35 – 7.28 (m, 2H), 7.27 – 7.20 (m, 3H), 3.37 (d, *J* = 7.1 Hz, 1H), 2.45 – 2.34 (m, 1H), 2.16 – 1.95 (m, 2H), 1.94 – 1.84 (m, 1H), 1.83 – 1.75 (m, 1H), 1.74 – 1.56 (m, 3H), 1.51 – 1.37 (m, 2H), 1.33 – 1.12 (m, 1H), 0.98 (d, *J* = 6.7 Hz, 3H), 0.74 (d, *J* = 6.7 Hz, 3H).

^{13}**C NMR** (101 MHz, $CDCl_3$) δ 143.5, 128.2, 127.8, 126.9, 123.7 (t, *J* = 240.8 Hz), 66.5, 50.8, 34.9, 31.7 (t, *J* = 24.3 Hz), 31.3 (t, *J* = 24.2 Hz). 30.3 (t, *J* = 4.9 Hz), 27.7 (t, *J* = 4.9 Hz), 19.9, 19.8.
^{19}**F NMR** (282 MHz, $CDCl_3$) δ -95.98 (d, *J* = 233.9 Hz), -98.07 (d, *J* = 235.0 Hz).
HRMS (GC-FI+) (m/z): $[M+H]^+$ calcd. for $C_{16}H_{23}F_2N$, 268.1877; found: 268.1878.

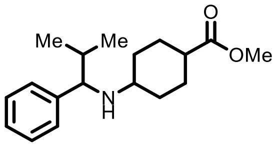

***methyl 4-((2-methyl-1-phenylpropyl)amino)cyclohexane-1-carboxylate* (9).** Prepared according to GP5 from **1e** (65 mg, 0.25 mmol, 1.0 equiv.). Purified via flash column chromatography on silica gel (Pentane:Ethyl Acetate 4:1, 0.5 vol% triethylamine) to afford the product as a colorless oil (59 mg, 82% yield).

^{1}**H NMR** (400 MHz, $CDCl_3$) δ 7.35 – 7.27 (m, 2H), 7.24 – 7.18 (m, 3H), 3.61 (s, 3H), 3.44 (d, *J* = 7.0 Hz, 1H), 2.29 – 2.14 (m, 2H), 2.14 – 2.05 (m, 1H), 1.99 – 1.67 (m, 4H), 1.45 – 1.21 (m, 3H), 1.17 – 0.96 (m, 2H), 0.94 (d, *J* = 6.7 Hz, 3H), 0.72 (d, *J* = 6.8 Hz, 3H).
^{13}**C NMR** (101 MHz, $CDCl_3$) δ 176. 5, 143.7, 128.1, 127.9, 126.8, 66.2, 53.4, 51.6, 43.1, 34.7, 33. 9, 32.2, 28.2, 27.8, 19.9, 19.6.
HRMS (GC-FI+) (m/z): $[M+H]^+$ calcd. for $C_{18}H_{27}NO_2$, 290.2120; found: 290.2122.

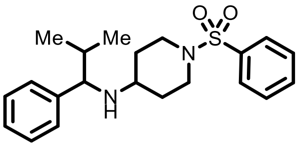

***N-(2-methyl-1-phenylpropyl)-1-(phenylsulfonyl)piperidin-4-amineN-(3-chloropropyl)-2-methyl-1-henylpropan-1-amine* (10).** Prepared according to GP5 from **1f** (86 mg, 0.25 mmol, 1.0 equiv.). Purified via flash column chromatography on silica gel (from Pentane:Ethyl Acetate 5:1 to 1:1) to afford the product as a white solid (62 mg, 67% yield).

^{1}**H NMR** (400 MHz, $CDCl_3$) δ 7.74 – 7.67 (m, 2H), 7.59 – 7.51 (m, 1H), 7.49 (dd, *J* = 8.2, 6.6 Hz, 2H), 7.25 (td, *J* = 6.5, 2.4 Hz, 2H), 7.23 – 7.12 (m, 3H), 3.64 – 3.50 (m, 2H), 3.34 (d, *J* = 7.0 Hz, 1H), 2.31 (qd, *J* = 10.9, 3.0 Hz, 2H), 2.16 (ddd, *J* = 9.8, 6.0, 3.9 Hz, 1H), 1.95 (dd, *J* = 12.8, 3.8 Hz, 1H), 1.70 (ddd, *J* = 16.2, 11.4, 5.8 Hz, 2H), 1.46 – 1.31 (m, 2H), 1.27 – 1.19 (m, 1H), 0.89 (d, *J* = 6.6 Hz, 3H), 0.68 (d, *J* = 6.7 Hz, 3H).
^{13}**C NMR** (101 MHz, $CDCl_3$) δ 143.3, 136.2, 132.7, 129.0, 128.1, 127.8, 127.7, 126.9, 65.9, 50.6, 45.2, 44.9, 34.8, 33.1, 31.1, 19.7, 19.7.
HRMS (ESI+) (m/z): $[M+H]^+$ calcd. for $C_{21}H_{28}N_2O_2S$, 373.1950; found: 373.1947.

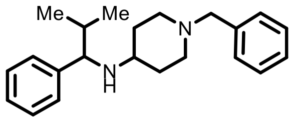

***1-benzyl-N-(2-methyl-1-phenylpropyl)piperidin-4-amine* (11).** Prepared according to GP6 from **1g** (74 mg, 0.25 mmol, 1.0 equiv.). Purified via flash column chromatography on silica gel (CH_2Cl_2:Acetone 20:1) to afford the product as a colorless oil (48 mg, 60% yield).

^{1}**H NMR** (400 MHz, $CDCl_3$) δ 7.32 – 7.16 (m, 10H), 3.47 (s, 2H), 3.38 (d, *J* = 7.0 Hz, 1H), 2.86 – 2.69 (m, 2H), 2.26 – 2.16 (m, 1H), 1.93 (t, *J* = 12.5 Hz, 3H), 1.76 (h, 1H), 1.67 – 1.59 (m, 1H), 1.47 – 1.28 (m, 2H), 0.91 (d, *J* = 6.6 Hz, 3H), 0.68 (d, *J* = 6.7 Hz, 3H). *NH not visible*.
^{13}**C NMR** (101 MHz, $CDCl_3$) δ 143.6, 137.5, 129.5, 128.4, 128.1, 128.0, 127.4, 126.8, 66.0, 63.0, 52.3, 52.0, 51.3, 34.8, 33.5, 31.6, 19.8, 19.8.
HRMS (ESI+) (m/z): $[M+H]^+$ calcd. for $C_{22}H_{30}N_2$, 323.2487; found: 323.2490.

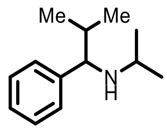

N-isopropyl-2-methyl-1-phenylpropan-1-amine **(12).** Prepared according to GP5 from **1h** (41 mg, 0.25 mmol, 1.0 equiv.). Purified via flash column chromatography on silica gel (Pentane:Ethyl Acetate 20:1, 0.5 vol% triethylamine) to afford the product as a colorless oil (29 mg, 60% yield).

^{1}H NMR (400 MHz, $CDCl_3$) δ 7.44 – 7.27 (m, 2H), 7.25 – 7.15 (m, 3H), 3.40 (d, *J* = 7.0 Hz, 1H), 2.52 (h, *J* = 6.2 Hz, 1H), 1.81 (h, *J* = 6.7 Hz, 1H), 1.45 – 1.13 (m, 1H), 0.98 (d, *J* = 6.1 Hz, 3H), 0.95 (d, *J* = 6.5 Hz, 6H), 0.73 (d, *J* = 6.7 Hz, 3H).
^{13}C NMR (101 MHz, $CDCl_3$) δ 143.8, 128.0, 128.0, 126.7, 66.7, 45.8, 34.7, 24.6, 22.1, 20.0, 19.6.
HRMS (GC-FI+) (m/z): $[M+H]^+$ calcd. for $C_{13}H_{21}N$, 192.1752; found: 192.1747.

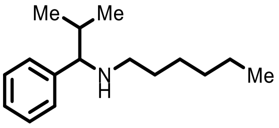

N-(2-methyl-1-phenylpropyl)hexan-1-amine **(13).** Prepared according to GP5 from **1i** (51 mg, 0.25 mmol, 1.0 equiv.). Purified via flash column chromatography on silica gel (Pentane:Ethyl Acetate 20:1, 0.5 vol% triethylamine) to afford the product as a colorless oil (37 mg, 64% yield).

^{1}H NMR (400 MHz, $CDCl_3$) δ 7.36 – 7.27 (m, 2H), 7.27 – 7.19 (m, 3H), 3.29 (d, *J* = 6.9 Hz, 1H), 2.36 (t, *J* = 7.2 Hz, 2H), 1.91 – 1.80 (m, 1H), 1.55 (br s, 1H), 1.56 – 1.33 (m, 2H), 1.29 – 1.07 (m, 6H), 0.97 (d, *J* = 6.6 Hz, 3H), 0.86 (t, *J* = 6.8 Hz, 3H), 0.74 (d, *J* = 6.7 Hz, 3H).
^{13}C NMR (101 MHz, $CDCl_3$) δ 143.3, 128.2, 128.0, 126.8, 69.9, 48.2, 34.5, 31.9, 30.3, 27.2, 22.8, 19.9, 19.6, 14.2.
HRMS (GC-FI+) (m/z): $[M+H]^+$ calcd. for $C_{16}H_{27}N$, 234.2222; found: 234.2220.

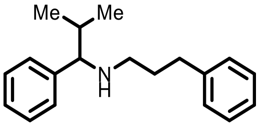

2-methyl-1-phenyl-N-(3-phenylpropyl)propan-1-amine **(14).** Prepared according to GP5 from **1j** (60 mg, 0.25 mmol, 1.0 equiv.). Purified via flash column chromatography on silica gel (Pentane:Ethyl Acetate 20:1, 0.5 vol% triethylamine) to afford the product as a colorless oil (32 mg, 48% yield).

^{1}H NMR (400 MHz, $CDCl_3$) δ 7.42 – 7.22 (m, 7H), 7.16 (m, 3H), 3.32 (d, *J* = 6.9 Hz, 1H), 2.70 – 2.52 (m, 2H), 2.47 (m, 2H), 1.96 – 1.85 (m, 1H), 1.84 – 1.64 (m, 2H), 1.39 – 1.18 (m, 1H), 0.98 (d, *J* = 6.7 Hz, 3H), 0.76 (d, *J* = 6.7 Hz, 3H).
^{13}C NMR (101 MHz, $CDCl_3$) δ 142.8, 142.4, 128.5, 128.4, 128.2, 128.1, 126.9, 125.8, 69.7, 47.5, 34.4, 33.7, 31.8, 19.9, 19.5.
HRMS (GC-FI+) (m/z): $[M+H]^+$ calcd. for $C_{19}H_{25}N$, 268.2065; found: 268.2063.

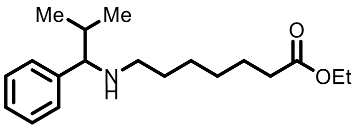

ethyl 7-((2-methyl-1-phenylpropyl)amino)heptanoate **(15).** Prepared according to GP5 from **1k** (69 mg, 0.25 mmol, 1.0 equiv.). Purified via flash column chromatography on silica gel (Pentane:Ethyl Acetate 10:1, 0.5 vol% triethylamine) to afford the product as a colorless oil (53 mg, 69% yield).

^{1}H NMR (400 MHz, $CDCl_3$) δ 7.32 – 7.27 (m, 2H), 7.25 – 7.19 (m, 3H), 4.11 (q, *J* = 7.1 Hz, 2H), 3.28 (d, *J* = 6.9 Hz, 1H), 2.35 (t, *J* = 7.1 Hz, 2H), 2.25 (t, *J* = 7.5 Hz, 2H), 1.96 – 1.76 (m, 1H), 1.75 – 1.52 (m, 2H), 1.50 – 1.36 (m, 3H), 1.32 – 1.14 (m, 7H), 0.96 (d, *J* = 6.7 Hz, 3H), 0.73 (d, *J* = 6.8 Hz, 3H).
^{13}C NMR (101 MHz, $CDCl_3$) δ 174.0, 143.3, 128.1, 128.0, 126.8, 69.9, 60.3, 48.0, 34.5, 34.4, 30.2, 29.2, 27.1, 25.1, 19.9, 19.6, 14.4.
HRMS (GC-FI+) (m/z): $[M+H]^+$ calcd. for $C_{19}H_{31}NO_2$, 306.2433; found: 306.2422.

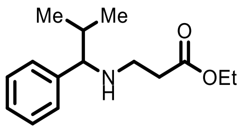

***ethyl 3-((2-methyl-1-phenylpropyl)amino)propanoate* (16).** Prepared according to GP5 from **1j** (55 mg, 0.25 mmol, 1.0 equiv.). Purified via flash column chromatography on silica gel (Pentane:Ethyl Acetate 10:1, 0.5 vol% triethylamine) to afford the product as a colorless oil (33 mg, 53% yield).

^{1}H NMR (400 MHz, $CDCl_3$) δ 7.33 – 7.27 (m, 2H), 7.27 – 7.19 (m, 3H), 4.13 (q, *J* = 7.2 Hz, 2H), 3.31 (d, *J* = 6.9 Hz, 1H), 2.65 (t, *J* = 6.3 Hz, 2H), 2.51 – 2.34 (m, 2H), 1.90 – 1.71 (m, 2H), 1.29 – 1.21 (m, 3H), 0.96 (d, *J* = 6.6 Hz, 3H), 0.73 (d, *J* = 6.6 Hz, 3H).
^{13}C NMR (101 MHz, $CDCl_3$) δ 173.2, 143.0, 128.1, 128.1, 126.9, 69.6, 60.5, 43.3, 35.0, 34.6, 19.7, 19.6, 14.4.
HRMS (GC-FI+) (m/z): $[M+H]^+$ calcd. for $C_{15}H_{23}NO_2$, 250.1807; found: 250.1800.

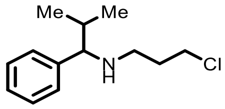

***N-(3-chloropropyl)-2-methyl-1-phenylpropan-1-amine* (17).** Prepared according to GP5 from **1m** (49 mg, 0.25 mmol, 1.0 equiv.). Purified via flash column chromatography on silica gel (from Pentane:Ethyl Acetate 20:1 to 5:1) to afford the product as a colorless oil (37 mg, 65% yield).

^{1}H NMR (400 MHz, $CDCl_3$) δ 7.38 – 7.20 (m, 5H), 3.69 – 3.51 (m, 2H), 3.31 (d, *J* = 6.9 Hz, 1H), 2.61 – 2.48 (m, 2H), 1.92 – 1.80 (m, 3H), 1.46 (s, 1H), 0.98 (d, *J* = 6.6 Hz, 3H), 0.76 (d, *J* = 6.7 Hz, 3H).
^{13}C NMR (101 MHz, $CDCl_3$) δ 143.0, 128.1, 128.0, 126.9, 69.7, 44.9, 43.3, 34.5, 33.2, 19.8, 19.6.
HRMS (ESI+) (m/z): $[M+H]^+$ calcd. for $C_{13}H_{20}ClN$, 226.1363; found: 226.1359.

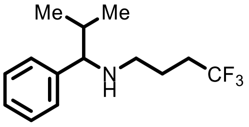

***4,4,4-trifluoro-N-(2-methyl-1-phenylpropyl)butan-1-amine* (18).** Prepared according to GP5 from **1n** (64 mg, 0.25 mmol, 1.0 equiv.). Purified via flash column chromatography on silica gel (gradient from Heptane:Ethyl Acetate 95:5 to 50:50) to afford the product as a pale yellow oil (33 mg, 51% yield).

^{1}H NMR (400 MHz, $CDCl_3$) δ 7.33 – 7.24 (m, 2H), 7.24 – 7.15 (m, 3H), 3.24 (d, *J* = 7.0 Hz, 1H), 2.50 – 2.32 (m, 2H), 2.23 – 1.94 (m, 2H), 1.88 – 1.72 (m, *J* = 6.8 Hz, 1H), 1.67 – 1.56 (m, 2H), 1.25 (br s, 1H), 0.94 (d, *J* = 6.7 Hz, 3H), 0.71 (d, *J* = 6.8 Hz, 3H).
^{13}C NMR (101 MHz, $CDCl_3$) δ 143.1, 128.2, 128.0, 127.5 (q, *J* = 275.0 Hz), 126.9, 69.6, 46.5, 34.6, 31.8 (q, *J* = 28.7 Hz), 22.8 (q, *J* = 2.8 Hz), 19.8, 19.6.
^{19}F NMR (376 MHz, $CDCl_3$) δ -66.4.
HRMS (ESI+) (m/z): $[M+H]^+$ calcd. for $C_{14}H_{20}F_3N$, 260.1626; found: 260.1627.

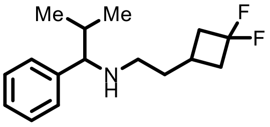

***N-(2-(3,3-difluorocyclobutyl)ethyl)-2-methyl-1-phenylpropan-1-amine* (19).** Prepared according to GP5 from **1o** (60 mg, 0.25 mmol, 1.0 equiv.). Purified via flash column chromatography on silica gel (gradient from Heptane:Ethyl Acetate 95:5 to 50:50) to afford the product as a yellow oil (43 mg, 64% yield).

^{1}H NMR (400 MHz, $CDCl_3$) δ 7.31 – 7.25 (m, 2H), 7.24 – 7.17 (m, 3H), 3.25 (d, *J* = 6.9 Hz, 1H), 2.65 – 2.46 (m, 2H), 2.40 – 2.25 (m, 2H), 2.18 – 1.97 (m, 3H), 1.87 – 1.73 (m, *J* = 6.8 Hz, 1H), 1.65 – 1.51 (m, 2H), 1.32 (br s, 1H), 0.93 (d, *J* = 6.7 Hz, 3H), 0.72 (d, *J* = 6.8 Hz, 3H).
^{13}C NMR (101 MHz, $CDCl_3$) δ 143.1, 128.1, 128.0, 126.9, 120.6 (dd, *J* = 284.5, 274.3 Hz), 69.9, 46.0, 40.9 (dd, *J* = 21.8, 8.8 Hz), 40.7 (dd, *J* = 21.0, 9.6 Hz), 36.8 – 36.6 (m), 34.5, 21.5 (dd, *J* = 12.5, 6.0 Hz), 19.8, 19.5.
^{19}F NMR (376 MHz, $CDCl_3$) δ -82.09 (d, *J* = 192.1 Hz), -95.39 (d, *J* = 192.4 Hz).

HRMS (ESI+) (m/z): $[M+H]^+$ calcd. for $C_{16}H_{23}F_2N$, 268.1877; found: 268.1881.

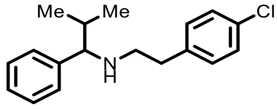

***N-(4-chlorophenethyl)-2-methyl-1-phenylpropan-1-amine* (20).** Prepared according to GP6 from **1p** (65 mg, 0.25 mmol, 1.0 equiv.). Purified via flash column chromatography on silica gel (from Pentane:Ethyl Acetate 20:1 to 5:1) to afford the product as a colorless oil (38 mg, 53% yield).

^{1}H NMR (300 MHz, $CDCl_3$) δ 7.39 – 7.19 (m, 7H), 7.18 – 7.04 (m, 2H), 3.35 (d, *J* = 7.0 Hz, 1H), 2.80 – 2.61 (m, 4H), 1.86 (h, *J* = 6.8 Hz, 1H), 1.31 (s, 1H), 0.96 (d, *J* = 6.7 Hz, 3H), 0.75 (d, *J* = 6.8 Hz, 3H).
^{13}C NMR (75 MHz, $CDCl_3$) δ 143.0, 138.9, 131.9, 130.2, 128.5, 128.1, 128.1, 126.9, 69.7, 48.9, 35.9, 34.5, 19.8, 19.5.
HRMS (ESI+) (m/z): $[M+H]^+$ calcd. for $C_{18}H_{22}ClN$, 288.1502; found: 288.1506.

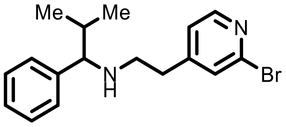

***N-(2-(2-bromopyridin-4-yl)ethyl)-2-methyl-1-phenylpropan-1-amine* (21).** Prepared according to GP6 from **1q** (76 mg, 0.25 mmol, 1.0 equiv.). Purified via flash column chromatography on silica gel (gradient from Heptane:Ethyl Acetate 90:10 to 50:50) to afford the product as a yellow oil (14 mg, 16% yield).

^{1}H NMR (400 MHz, $CDCl_3$) δ 8.19 (d, *J* = 5.0 Hz, 1H), 7.31 – 7.25 (m, 3H), 7.23 – 7.18 (m, 1H), 7.18 – 7.13 (m, 2H), 7.00 (dd, *J* = 5.1, 1.5 Hz, 1H), 3.26 (d, *J* = 7.0 Hz, 1H), 2.71 – 2.59 (m, 4H), 1.85 – 1.72 (m, *J* = 6.8 Hz, 1H), 1.30 – 1.18 (m, 1H), 0.91 (d, *J* = 6.7 Hz, 3H), 0.69 (d, *J* = 6.8 Hz, 3H).
^{13}C NMR (101 MHz, $CDCl_3$) δ 152.9, 149.9, 142.7, 142.5, 128.4, 128.2, 128.0, 127.1, 123.5, 69.7, 47.6, 35.7, 34.6, 19.8, 19.6.
HRMS (ESI+) (m/z): $[M+H]^+$ calcd. for $C_{17}H_{21}BrN_2$, 333.0966; found: 333.0971.

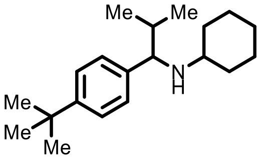

***N-(1-(4-(tert-butyl)phenyl)-2-methylpropyl)cyclohexanamine* (23).** Prepared according to GP5 from **2a** (65 mg, 0.25 mmol, 1.0 equiv.). Purified via flash column chromatography on silica gel (Pentane:Ethyl Acetate 30:1) to afford the product as a colorless oil (39 mg, 54% yield).

^{1}H NMR (400 MHz, $CDCl_3$) δ 7.29 (d, *J* = 8.3 Hz, 2H), 7.13 (d, *J* = 8.3 Hz, 2H), 3.43 (d, *J* = 6.8 Hz, 1H), 2.20 (tt, *J* = 9.9, 3.6 Hz, 1H), 1.99 – 1.90 (m, 1H), 1.86 – 1.73 (m, *J* = 6.8 Hz, 1H), 1.70 – 1.59 (m, 3H), 1.55 – 1.45 (m, 1H), 1.32 (s, 9H), 1.19 – 0.97 (m, 6H), 0.93 (d, *J* = 6.7 Hz, 3H), 0.73 (d, *J* = 6.8 Hz, 3H).
^{13}C NMR (101 MHz, $CDCl_3$) δ 149.2, 140.9, 127.5, 124.8, 65.5, 53.5, 35.0, 34.8, 34.5, 33.1, 31.6, 26.4, 25.4, 24.9, 19.8, 19.8.
HRMS (ESI+) (m/z): $[M+H]^+$ calcd. for $C_{20}H_{33}N$, 288.2691; found: 288.2691.

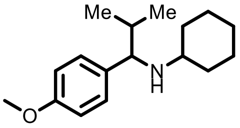

***N-(1-(4-methoxyphenyl)-2-methylpropyl)cyclohexanamine* (24).** Prepared according to GP5 from **2b** (58 mg, 0.25 mmol, 1.0 equiv.). Purified via flash column chromatography on silica gel (Pentane:Ethyl Acetate 10:1, 0.5 vol% triethylamine) to afford the product as a colorless oil (21 mg, 32% yield).

^{1}H NMR (400 MHz, $CDCl_3$) δ 7.19 – 7.11 (m, 2H), 6.92 – 6.77 (m, 2H), 3.80 (s, 3H), 3.41 (d, *J* = 6.9 Hz, 1H), 2.26 – 2.07 (m, 1H), 1.99 – 1.88 (m, 1H), 1.85 – 1.68 (m, 1H), 1.69 – 1.58 (m, 3H), 1.51 (s, 1H), 1.37-1.21 (m, 1H), 1.15 – 1.01 (m, 5H), 0.93 (d, *J* = 6.7 Hz, 3H), 0.71 (d, *J* = 6.6 Hz, 3H).
^{13}C NMR (101 MHz, $CDCl_3$) δ 158.4, 136.0, 128.9, 113.3, 65.4, 55.3, 53.6, 35.1, 34.9, 33.0, 26.4, 25.4, 25.0, 20.0, 19.6.
HRMS (GC-FI+) (m/z): $[M+H]^+$ calcd. for $C_{17}H_{27}NO$, 262.2171; found: 262.2179.

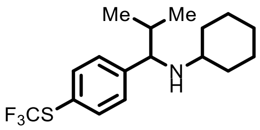

N-(2-methyl-1-(4-((trifluoromethyl)thio)phenyl)propyl)cyclohexanamine **(25).** Prepared according to GP5 from **2c** (76 mg, 0.25 mmol, 1.0 equiv.). Purified via flash column chromatography on silica gel Pentane:Ethyl Acetate 30:1) to afford the product as a colorless oil (50 mg, 60% yield).

^{1}H NMR (400 MHz, $CDCl_3$) δ 7.57 (d, *J* = 8.0 Hz, 2H), 7.32 (d, *J* = 8.2 Hz, 2H), 3.52 (d, *J* = 6.7 Hz, 1H), 2.14 (ddd, *J* = 9.9, 6.4, 3.5 Hz, 1H), 1.93 (d, *J* = 12.7 Hz, 1H), 1.83 – 1.74 (m, 1H), 1.67 – 1.61 (m, 3H), 1.55 – 1.49 (m, 1H), 1.30 (s, 1H), 1.12 – 0.95 (m, 5H), 0.92 (d, *J* = 6.7 Hz, 3H), 0.74 (d, *J* = 6.8 Hz, 3H).
^{13}C NMR (101 MHz, $CDCl_3$) δ 147.8, 136.1, 129.8 (q, *J* = 307.3 Hz), 129.2, 122.0 (q, *J* = 2.3 Hz), 65.7, 53.9, 35.1, 34.8, 33.0, 26.3, 25.3, 24.9, 19.6, 19.5.
^{19}F NMR (282 MHz, $CDCl_3$) δ -42.94.
HRMS (ESI+) (m/z): $[M+H]^+$ calcd. for $C_{17}H_{24}F_3NS$, 332.1660; found: 332.1659.

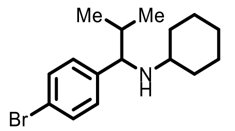

N-(1-(4-bromophenyl)-2-methylpropyl)cyclohexanamine **(26).** Prepared according to GP5 from **2d** (72 mg, 0.25 mmol, 1.0 equiv.). Purified via flash column chromatography on silica gel (from Pentane:Ethyl Acetate 30:1 to 10:1) to afford the product as a colorless oil (40 mg, 52% yield).

^{1}H NMR (400 MHz, $CDCl_3$) δ 7.57 (d, *J* = 8.0 Hz, 2H), 7.34 – 7.29 (m, 2H), 3.52 (d, *J* = 6.7 Hz, 1H), 2.20 – 2.08 (m, 1H), 1.93 (d, *J* = 12.7 Hz, 1H), 1.87 – 1.72 (m, *J* = 6.8 Hz, 1H), 1.70 – 1.57 (m, 3H), 1.57 – 1.46 (m, 1H), 1.35 – 1.27 (m, 1H), 1.19 – 0.95 (m, 5H), 0.92 (d, *J* = 6.7 Hz, 3H), 0.74 (d, *J* = 6.8 Hz, 3H).
^{13}C NMR (101 MHz, $CDCl_3$) δ 143.3, 131.1, 129.8, 120.2, 65.5, 53.8, 35.1, 34.7, 33.0, 26.3, 25.3, 24.9, 19.7, 19.5.
HRMS (ESI+) (m/z): $[M+H]^+$ calcd. for $C_{16}H_{24}N$, 310.1170; found: 310.1165.

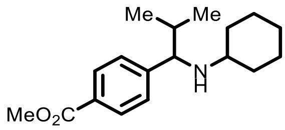

methyl 4-(1-(cyclohexylamino)-2-methylpropyl)benzoate **(27).** Prepared according to GP5 from **2e** (65 mg, 0.25 mmol, 1.0 equiv.). Purified via flash column chromatography on silica gel (Pentane:Ethyl Acetate 10:1, 0.5 vol% triethylamine) to afford the product as a colorless oil (56 mg, 79% yield).

^{1}H NMR (300 MHz, $CDCl_3$) δ 7.97 (d, *J* = 8.3 Hz, 2H), 7.32 (d, *J* = 8.3 Hz, 2H), 3.90 (s, 3H), 3.52 (d, *J* = 6.8 Hz, 1H), 2.17 – 2.07 (m, 1H), 1.92 (d, *J* = 11.0 Hz, 1H), 1.80 (h, *J* = 6.7 Hz, 1H), 1.68 – 1.47 (m, 4H), 1.27 (s, 1H), 1.10 (d, *J* = 9.9 Hz, 5H), 0.93 (d, *J* = 6.7 Hz, 3H), 0.72 (d, *J* = 6.8 Hz, 3H).
^{13}C NMR (101 MHz, $CDCl_3$) δ 167.4, 150.0, 129.4, 128.6, 128.1, 66.0, 54.0, 52.1, 35.0, 34.7, 33.0, 26.3, 25.3, 24.9, 19.7, 19.6.
HRMS (GC-FI+) (m/z): $[M+H]^+$ calcd. for $C_{18}H_{27}NO_2$, 290.2120; found: 290.2119.

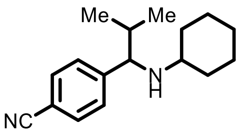

***4-(1-(cyclohexylamino)-2-methylpropyl)benzonitrile* (28).** Prepared according to GP5 from **2f** (57 mg, 0.25 mmol, 1.0 equiv.). Purified via flash column chromatography on silica gel (gradient from Pentane:Ethyl Acetate 95:5 to 80:20) to afford the product as a pale yellow oil (37 mg, 58% yield).

^{1}H NMR (400 MHz, $CDCl_3$) δ 7.61 – 7.54 (m, 2H), 7.41 – 7.35 (m, 2H), 3.54 (d, *J* = 6.5 Hz, 1H), 2.09 (tt, *J* = 9.8, 3.6 Hz, 1H), 1.91 (d, *J* = 12.6 Hz, 1H), 1.86 – 1.70 (m, *J* = 6.8 Hz, 1H), 1.70 – 1.54 (m, 3H), 1.56 – 1.46 (m, 1H), 1.27 – 1.18 (m, 1H), 1.15 – 0.93 (m, 5H), 0.91 (d, *J* = 6.7 Hz, 3H), 0.73 (d, *J* = 6.8 Hz, 3H).
^{13}C NMR (101 MHz, $CDCl_3$) δ 150.4, 131.9, 128.8, 119.3, 110.4, 65.9, 54.2, 35.0, 34.8, 33.0, 26.2, 25.2, 24.9, 19.5, 19.4.
HRMS (ESI+) (m/z): $[M+H]^+$ calcd. for $C_{17}H_{24}N_2$, 257.2018; found: 257.2016.

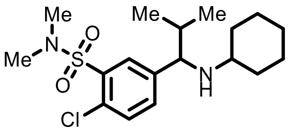

***2-chloro-5-(1-(cyclohexylamino)-2-methylpropyl)-N,N-dimethylbenzenesulfonamide* (29).** Prepared according to GP5 from **2g** (86 mg, 0.25 mmol, 1.0 equiv.). Purified via flash column chromatography on silica gel (gradient from Heptane:Ethyl Acetate 95:5 to 60:40) to afford the product as a pale yellow oil (66 mg, 70% yield).

^{1}H NMR (400 MHz, $CDCl_3$) δ 7.92 (d, *J* = 1.8 Hz, 1H), 7.47 – 7.38 (m, 2H), 3.55 (d, *J* = 6.4 Hz, 1H), 2.86 (s, 6H), 2.08 (tt, *J* = 9.8, 3.5 Hz, 1H), 1.93 – 1.85 (m, 1H), 1.82 – 1.70 (m, *J* = 6.7 Hz, 1H), 1.69 – 1.54 (m, 3H), 1.50 (d, *J* = 5.0 Hz, 1H), 1.28 – 1.19 (m, 1H), 1.14 – 0.92 (m, 5H), 0.89 (d, *J* = 6.7 Hz, 3H), 0.73 (d, *J* = 6.8 Hz, 3H).
^{13}C NMR (101 MHz, $CDCl_3$) δ 144.1, 135.1, 133.0, 131.8, 131.7, 130.0, 65.2, 54.2, 37.5, 35.0, 34.7, 33.0, 26.2, 25.2, 24.9, 19.3, 19.3.
HRMS (ESI+) (m/z): $[M+H]^+$ calcd. for $C_{18}H_{29}ClN_2O_2S$, 373.1717; found: 373.1717.

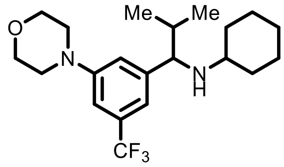

***N-(2-methyl-1-(3-morpholino-5-(trifluoromethyl)phenyl)propyl)cyclohexanamine* (30).** Prepared according to GP5 from **2h** (89 mg, 0.25 mmol, 1.0 equiv.). Purified via flash column chromatography on silica gel (gradient from Heptane:Ethyl Acetate 90:10 to 0:100) to afford the product as colorless oil (50 mg, 52% yield).

^{1}H NMR (400 MHz, $CDCl_3$) δ 7.03 (dd, *J* = 5.1, 3.0 Hz, 2H), 6.97 (d, *J* = 2.0 Hz, 1H), 3.92 – 3.82 (m, 4H), 3.49 (d, *J* = 6.6 Hz, 1H), 3.25 – 3.13 (m, 4H), 2.16 (tt, *J* = 9.9, 3.5 Hz, 1H), 1.98 – 1.87 (m, 1H), 1.84 – 1.72 (m, *J* = 6.7 Hz, 1H), 1.72 – 1.57 (m, 3H), 1.57 – 1.47 (m, 1H), 1.34 – 1.25 (m, 1H), 1.21 – 0.95 (m, 5H), 0.92 (d, *J* = 6.7 Hz, 3H), 0.76 (d, *J* = 6.8 Hz, 3H).
^{13}C NMR (101 MHz, $CDCl_3$) δ 151.3, 146.6, 131.0 (q, *J* = 31.4 Hz), 124.6 (q, *J* = 272.5 Hz), 118.3 (q, *J* = 1.5 Hz), 116.4 (q, *J* = 3.9 Hz), 110.4 (q, *J* = 3.8 Hz), 66.9, 66.2, 54.0, 49.2, 35.1, 34.8, 33.1, 26.3, 25.3, 24.9, 19.8, 19.4.
^{19}F NMR (377 MHz, $CDCl_3$) δ -62.5.
HRMS (ESI+) (m/z): $[M+H]^+$ calcd. for $C_{21}H_{31}F_3N_2O$, 385.2467; found: 385.2474.

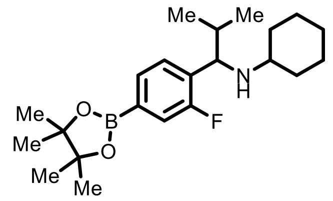

N-(1-(2-fluoro-4-(4,4,5,5-tetramethyl-1,3,2-dioxaborolan-2-yl)phenyl)-2-methylpropyl)cyclohexanamine **(31).** Prepared according to GP5 from **2i** (87 mg, 0.25 mmol, 1.0 equiv.). Purified via flash column chromatography on silica gel (gradient from Heptane:Ethyl Acetate 95:5 to 20:80) to afford the product as a colorless oil (31 mg, 33% yield).

^{1}H NMR (400 MHz, $CDCl_3$) δ 7.54 (dd, *J* = 7.5, 1.2 Hz, 1H), 7.42 (dd, *J* = 10.7, 1.1 Hz, 1H), 7.32 (t, *J* = 7.3 Hz, 1H), 3.83 (d, *J* = 7.2 Hz, 1H), 2.14 (ddt, *J* = 9.7, 7.1, 3.6 Hz, 1H), 1.97 – 1.80 (m, 2H), 1.70 – 1.55 (m, 2H), 1.54 – 1.46 (m, 2H), 1.34 (s, 12H), 1.31 – 1.22 (m, 1H), 1.18 – 0.99 (m, 5H), 0.99 – 0.94 (m, 3H), 0.79 – 0.72 (m, 3H).
^{13}C NMR (101 MHz, $CDCl_3$) δ 161.2 (d, *J* = 244.8 Hz), 134.5 (d, *J* = 13.6 Hz), 130.2 (d, *J* = 3.1 Hz), 128.8 (d, *J* = 4.7 Hz), 121.0 (d, *J* = 21.4 Hz), 84.1, 59.1, 54.1, 34.9, 34.2, 32.9, 26.3, 25.2, 25.0, 24.8, 19.8, 19.6.
^{11}B NMR (128 MHz, $CDCl_3$) δ 30.93.
^{19}F NMR (377 MHz, $CDCl_3$) δ -120.2.
HRMS (ESI+) (m/z): $[M+H]^+$ calcd. for $C_{22}H_{35}BFNO_2$, 376.2827; found: 376.2825.

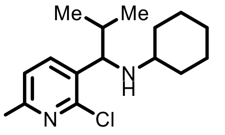

N-(1-(2,6-dichloropyridin-3-yl)-2-methylpropyl)cyclohexanamine **(32).** Prepared according to GP6 from **2j** (68 mg, 0.25 mmol, 1.0 equiv.). Purified via flash column chromatography on silica gel (Pentane:Ethyl Acetate 95:5) to afford the product as a pale yellow oil (34 mg, 45% yield).

^{1}H NMR (400 MHz, $CDCl_3$) δ 7.86 (d, *J* = 8.0 Hz, 1H), 7.26 (d, *J* = 8.1 Hz, 1H), 4.00 (d, *J* = 5.9 Hz, 1H), 2.07 (tt, *J* = 10.0, 3.7 Hz, 1H), 1.94 (d, *J* = 12.8 Hz, 1H), 1.91 – 1.80 (m, 1H), 1.73 – 1.48 (m, 4H), 1.30 – 1.07 (m, 4H), 1.05 – 0.91 (m, 2H), 0.89 (d, *J* = 6.8 Hz, 6H).
^{13}C NMR (101 MHz, $CDCl_3$) δ 149.6, 148.0, 140.8, 138.1, 123.1, 60.7, 54.7, 35.0, 33.7, 33.2, 26.2, 25.2, 24.9, 19.8, 18.1.
HRMS (ESI+) (m/z): $[M+H]^+$ calcd. for $C_{15}H_{22}Cl_2N_2$, 301.1238; found: 301.1241.

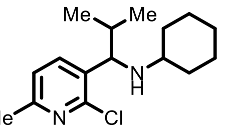

N-(1-(2-chloro-6-methylpyridin-3-yl)-2-methylpropyl)cyclohexanamine **(33).** Prepared according to GP6 from **2k** (63 mg, 0.25 mmol, 1.0 equiv.). Purified via flash column chromatography on silica gel (from Pentane:Ethyl Acetate 30:1to 10:1) to afford the product as a colorless oil (37 mg, 52% yield).

^{1}H NMR (300 MHz, $CDCl_3$) δ 7.70 (d, *J* = 7.7 Hz, 1H), 7.07 (d, *J* = 7.8 Hz, 1H), 3.98 (d, *J* = 6.2 Hz, 1H), 2.51 (s, 3H), 2.12 – 2.03 (m, 1H), 2.01 – 1.91 (m, 1H), 1.85 (h, *J* = 6.7 Hz, 1H), 1.70 – 1.47 (m, 4H), 1.29 – 0.97 (m, 6H), 0.90 (d, *J* = 6.7 Hz, 3H), 0.86 (d, *J* = 6.8 Hz, 3H).
^{13}C NMR (75 MHz, $CDCl_3$) δ 157.0, 150.1, 138.5, 135.5, 122.2, 60.9, 54.3, 35.0, 33.8, 33.1, 26.3, 25.2, 24.9, 23.8, 19.9, 18.3.
HRMS (ESI+) (m/z): $[M+H]^+$ calcd. for $C_{16}H_{25}N_2Cl$, 281.1785; found: 281.1789.

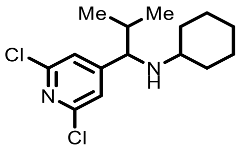

N-(1-(2,6-dichloropyridin-4-yl)-2-methylpropyl)cyclohexanamine **(34).** Prepared according to GP6 from **2l** (68 mg, 0.25 mmol, 1.0 equiv.). Purified via flash column chromatography on silica gel (Pentane:Ethyl Acetate 10:1, 0.5 vol% triethylamine) to afford the product as a colorless oil (23 mg, 31% yield).

^{1}H NMR (400 MHz, $CDCl_3$) δ 7.21 (s, 2H), 3.50 (d, *J* = 6.1 Hz, 1H), 2.12 (ddt, *J* = 9.9, 7.1, 3.6 Hz, 1H), 1.92 – 1.85 (m, 1H), 1.78 (h, *J* = 6.7 Hz, 1H), 1.70 – 1.46 (m, 4H), 1.19 – 1.09 (m, 4H), 1.05 – 0.94 (m, 2H), 0.89 (d, *J* = 6.7 Hz, 3H), 0.81 (d, *J* = 6.8 Hz, 3H).
^{13}C NMR (101 MHz, $CDCl_3$) δ 160.9, 150.3, 122.5, 64.9, 54.7, 35.0, 34.6, 33.1, 26.2, 25.2, 24.9, 19.5, 18.9.
HRMS (ESI+) (m/z): $[M+H]^+$ calcd. for $C_{15}H_{22}N_2Cl_2$, 301.1238; found: 301.1237.

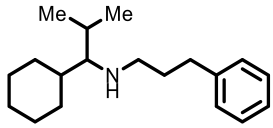

1-cyclohexyl-2-methyl-N-(3-phenylpropyl)propan-1-amine **(35).** Prepared according to GP5 from **2m** (61 mg, 0.25 mmol, 1.0 equiv.). Purified via flash column chromatography on silica gel (gradient from Pentane:Ethyl Acetate 95:5 to 80:20) to afford the product as a yellow oil (29 mg, 42% yield).

^{1}H NMR (400 MHz, $CDCl_3$) δ 7.32 – 7.24 (m, 2H), 7.23 – 7.13 (m, 3H), 2.72 – 2.62 (m, 4H), 1.87 (t, *J* = 5.6 Hz, 1H), 1.83 – 1.70 (m, 6H), 1.69 – 1.57 (m, 2H), 1.37 (tdt, *J* = 11.9, 6.2, 3.1 Hz, 1H), 1.30 – 0.96 (m, 6H), 0.89 (dd, *J* = 16.0, 6.7 Hz, 6H).
^{13}C NMR (101 MHz, $CDCl_3$) δ 142.6, 128.5, 128.4, 125.8, 68.9, 51.7, 41.5, 33.9, 32.8, 31.3, 30.2, 29.1, 26.9, 26.9, 26.8, 21.0, 18.0.
HRMS (ESI+) (m/z): $[M+H]^+$ calcd. for $C_{19}H_{31}N$, 274.2535; found: 274.2536.

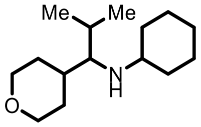

N-(2-methyl-1-(tetrahydro-2H-pyran-4-yl)propyl)cyclohexanamine **(36).** Prepared according to GP5 from **2n** (53 mg, 0.25 mmol, 1.0 equiv.). Purified via flash column chromatography on silica gel (gradient from Pentane:Ethyl Acetate 60:40 to 40:60) to afford the product as a colorless oil (28 mg, 47% yield).

^{1}H NMR (400 MHz, $CDCl_3$) δ 4.04 – 3.92 (m, 2H), 3.34 (td, *J* = 11.4, 2.4 Hz, 2H), 2.34 (t, *J* = 9.9 Hz, 1H), 2.05 (d, *J* = 5.7 Hz, 1H), 1.91 – 1.76 (m, 3H), 1.76 – 1.65 (m, 3H), 1.62 – 1.30 (m, 6H), 1.29 – 1.07 (m, 4H), 1.04 – 0.97 (m, 1H), 0.93 (d, *J* = 6.8 Hz, 3H), 0.84 (d, *J* = 6.9 Hz, 3H).
^{13}C NMR (101 MHz, $CDCl_3$) δ 68.7, 68.3, 64.3, 57.4, 39.5, 34.9, 34.7, 31.3, 29.9, 29.7, 26.3, 25.5, 25.5, 21.1, 17.3.
HRMS (ESI+) (m/z): $[M+H]^+$ calcd. for $C_{15}H_{29}NO$, 240.2327; found: 240.2323.

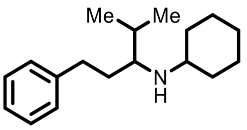

N-(4-methyl-1-phenylpentan-3-yl)cyclohexanamine **(37).** Prepared according to GP6 from **2o** (58 mg, 0.25 mmol, 1.0 equiv.). Purified via flash column chromatography on silica gel (gradient from Pentane:Ethyl Acetate 90:10 to 70:30) to afford the product as a colorless oil (52 mg, 80% yield).

^{1}H NMR (400 MHz, $CDCl_3$) δ 7.32 – 7.24 (m, 2H), 7.23 – 7.13 (m, 3H), 2.72 (ddd, *J* = 13.8, 10.6, 5.5 Hz, 1H), 2.59 (ddd, *J* = 13.8, 10.5, 5.9 Hz, 1H), 2.48 – 2.37 (m, 2H), 1.89 – 1.76 (m, 3H), 1.77 – 1.64 (m, 3H), 1.64 – 1.46 (m, 2H), 1.32 – 0.98 (m, 6H), 0.88 (dd, *J* = 6.9, 1.2 Hz, 6H).
^{13}C NMR (101 MHz, $CDCl_3$) δ 143.1, 128.5, 128.4, 125.7, 59.4, 54.6, 34.6, 34.5, 33.6, 33.1, 30.5, 26.4, 25.4, 25.4, 18.8, 18.1.
HRMS (ESI+) (m/z): $[M+H]^+$ calcd. for $C_{18}H_{29}N$, 260.2378; found: 260.2376.

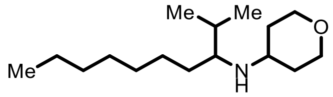

N-(2-methyldecan-3-yl)tetrahydro-2H-pyran-4-amine **(38).** Prepared according to GP5 from **2p** (57 mg, 0.25 mmol, 1.0 equiv.). Purified via flash column chromatography on silica gel (gradient from Pentane:Ethyl Acetate 70:30 to 50:50) to afford the product as a colorless oil (48 mg, 75% yield).

^{1}H NMR (400 MHz, $CDCl_3$) δ 3.95 (dd, *J* = 11.6, 3.6 Hz, 2H), 3.39 (td, *J* = 11.3, 2.4 Hz, 2H), 2.68 (ddt, *J* = 14.4, 9.8, 4.0 Hz, 1H), 2.40 – 2.32 (m, 1H), 1.83 – 1.68 (m, 3H), 1.43 – 1.16 (m, 14H), 0.96 – 0.74 (m, 10H).
^{13}C NMR (101 MHz, $CDCl_3$) δ 67.1, 67.1, 59.5, 52.0, 34.9, 34.8, 32.1, 31.8, 30.8, 30.1, 29.5, 26.8, 22.8, 18.5, 18.4, 14.2.
HRMS (ESI+) (m/z): $[M+H]^+$ calcd. for $C_{16}H_{33}NO$, 256.2640; found: 256.2639.

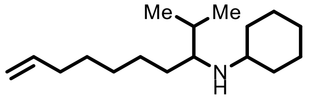

N-(2-methyldec-9-en-3-yl)cyclohexanamine **(39).** Prepared according to GP6 from **2q** (56 mg, 0.25 mmol, 1.0 equiv.). Purified via flash column chromatography on silica gel (Pentane:Ethyl Acetate 90:10 + 0.5% NEt_3) to afford the product as a colorless oil (27 mg, 42% yield).

^{1}H NMR (400 MHz, $CDCl_3$) δ 5.81 (ddt, *J* = 16.9, 10.3, 6.7 Hz, 1H), 4.99 (dq, *J* = 17.1, 1.7 Hz, 1H), 4.93 (ddt, *J* = 10.2, 2.3, 1.2 Hz, 1H), 2.45 – 2.29 (m, 2H), 2.10 – 1.99 (m, 2H), 1.89 – 1.79 (m, 2H), 1.78 – 1.66 (m, 3H), 1.63 – 1.54 (m, 1H), 1.46 – 1.09 (m, 12H), 1.08 – 0.94 (m, 2H), 0.85 (d, *J* = 6.8 Hz, 6H).
^{13}C NMR (101 MHz, $CDCl_3$) δ 139.3, 114.3, 59.8, 54.9, 34.7, 34.5, 34.0, 31.7, 30.6, 29.7, 29.1, 26.7, 26.4, 25.5 (2C), 18.7, 18.2.
HRMS (ESI+) (m/z): $[M+H]^+$ calcd. for $C_{17}H_{33}N$, 252.2691; found: 252.2687.

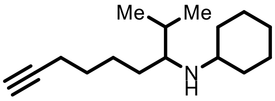

N-(2-methylnon-8-yn-3-yl)cyclohexanamine **(40).** Prepared according to GP6 from **2r** (52 mg, 0.25 mmol, 1.0 equiv.). Purified via flash column chromatography on silica gel (gradient from Pentane:Ethyl Acetate 95:5 + 0.5% NEt_3 to 80:20 + 0.5% NEt_3) to afford the product as a pale yellow oil with a 15% of an inseparable minor olefinic impurity (35 mg, 59% yield, 85% purity).

^{1}H NMR (400 MHz, $CDCl_3$) δ 2.45 – 2.30 (m, 2H), 2.19 (td, *J* = 7.0, 2.7 Hz, 2H), 1.93 (t, *J* = 2.7 Hz, 1H), 1.89 – 1.78 (m, 2H), 1.77 – 1.65 (m, 4H), 1.63 – 1.30 (m, 6H), 1.30 – 1.13 (m, 4H), 1.10 – 0.96 (m, 2H), 0.85 (d, *J* = 6.9 Hz, 6H).
^{13}C NMR (101 MHz, $CDCl_3$) δ 84.8, 68.3, 59.7, 54.9, 34.6, 34.4, 31.2, 30.6, 28.9, 26.3, 25.9, 25.4, 25.4, 18.6, 18.5, 18.2.
HRMS (ESI+) (m/z): $[M+H]^+$ calcd. for $C_{16}H_{29}N$, 236.2378; found: 236.2373.

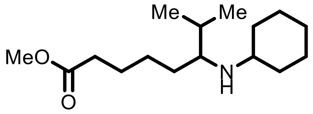

methyl 6-(cyclohexylamino)-7-methyloctanoate **(41).** Prepared according to GP5 from **2s** (60 mg, 0.25 mmol, 1.0 equiv.). Purified via flash column chromatography on silica gel (gradient from Pentane:Ethyl Acetate 80:20 to 50:50) to afford the product as a pale yellow oil (34 mg, 50% yield).

^{1}H NMR (400 MHz, $CDCl_3$) δ 3.66 (dt, *J* = 3.3, 1.6 Hz, 3H), 2.44 – 2.26 (m, 4H), 1.89 – 1.52 (m, 8H), 1.45 – 1.32 (m, 2H), 1.31 – 1.09 (m, 6H), 1.08 – 0.94 (m, 2H), 0.89 – 0.81 (m, 6H).
^{13}C NMR (101 MHz, $CDCl_3$) δ 174.4, 59.6, 54.8, 51.6, 34.6, 34.5, 34.2, 31.4, 30.5, 26.3, 26.3, 25.5, 25.4, 25.4, 18.7, 18.1.
HRMS (ESI+) (m/z): $[M+H]^+$ calcd. for $C_{16}H_{31}NO_2$, 270.2433; found: 270.2438.

11.3 Characterization of compounds 43-54 (alkyl iodides scope)

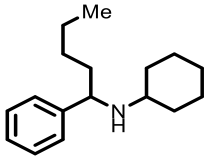

***N-(1-phenylpentyl)cyclohexanamine* (43).** Prepared according to GP5 from **1a** (51 mg, 0.25 mmol, 1.0 equiv.) and 1-iodobutane (138 mg, 0.75 mmol, 3.0 equiv.). Purified via flash column chromatography on silica gel (Pentane:Ethyl Acetate 10:1, 0.5 vol% triethylamine) to afford the product as a colorless oil (32 mg, 53% yield).

^{1}H NMR (400 MHz, $CDCl_3$) δ 7.36 – 7.28 (m, 2H), 7.28 – 7.19 (m, 3H), 3.72 (t, *J* = 6.9 Hz, 1H), 2.36 – 2.18 (m, 1H), 2.06 – 1.90 (m, 1H), 1.74 – 1.47 (m, 6H), 1.34 – 0.93 (m, 10H), 0.83 (t, *J* = 7.0 Hz, 3H).
^{13}C NMR (101 MHz, $CDCl_3$) δ 145.5, 128.4, 127.3, 126.7, 59.8, 53.6, 38.8, 34.9, 33.2, 28.8, 26.3, 25.4, 25.0, 22.6, 14.1.
HRMS (GC-FI+) (m/z): $[M+H]^+$ calcd. for $C_{17}H_{27}N$, 246.2222; found: 246.2220.

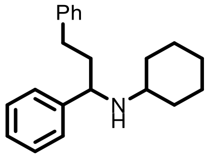

***N-(1,3-diphenylpropyl)cyclohexanamine* (44).** Prepared according to GP5 from **1a** (51 mg, 0.25 mmol, 1.0 equiv.) and (2-iodoethyl)benzene (174 mg, 0.75 mmol, 3.0 equiv.). Purified via flash column chromatography on silica gel (Pentane:Ethyl Acetate 10:1, 0.5 vol% triethylamine) to afford the product as a colorless oil (36 mg, 49% yield).

^{1}H NMR (400 MHz, $CDCl_3$) δ 7.37 – 7.31 (m, 1H), 7.31 – 7.22 (m, 3H), 7.19 – 7.11 (m, 1H), 3.78 (t, *J* = 6.9 Hz, 0H), 2.64 – 2.44 (m, 1H), 2.30 – 2.19 (m, 1H), 2.10 – 1.86 (m, 1H), 1.73 – 1.49 (m, 2H), 1.28 (s, 1H), 1.18 – 0.97 (m, 2H).
^{13}C NMR (101 MHz, $CDCl_3$) δ 145.0, 142.3, 128.5 (2C), 128.4, 127.3, 127.0, 125.8, 59.3, 53.6, 40.3, 34.8, 33.2, 32.8, 26.3, 25.3, 25.0.
HRMS (GC-FI+) (m/z): $[M+H]^+$ calcd. for $C_{21}H_{27}N$, 294.2222; found: 294.2225.

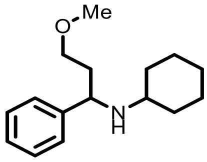

***N-(3-methoxy-1-phenylpropyl)cyclohexanamine* (45).** Prepared according to GP5 from **1a** (51 mg, 0.25 mmol, 1.0 equiv.) and 1-iodo-2-methoxyethane (140 mg, 0.75 mmol, 3.0 equiv.). Purified via flash column chromatography on silica gel (Pentane:Ethyl Acetate 10:1, 0.5 vol% triethylamine) to afford the product as a colorless oil (24 mg, 39% yield).

^{1}H NMR (400 MHz, $CDCl_3$) δ 7.32 – 7.26 (m, 3H), 7.25 – 7.18 (m, 2H), 3.89 (t, *J* = 7.0 Hz, 1H), 3.36 – 3.27 (m, 1H), 3.25 (s, 3H), 3.23 – 3.16 (m, 1H), 2.27 – 2.16 (m, 1H), 2.02 – 1.87 (m, 2H), 1.81 – 1.69 (m, 1H), 1.67 – 1.55 (m, 3H), 1.54 – 1.41 (m, 1H), 1.22 (s, 1H), 1.16 – 0.90 (m, 5H).
^{13}C NMR (101 MHz, $CDCl_3$) δ 144.8, 128.5, 127.2, 126.9, 70.4, 58.7, 57.2, 53.6, 38.7, 34.9, 33.1, 26.3, 25.3, 24.9.
HRMS (GC-FI+) (m/z): $[M+H]^+$ calcd. for $C_{16}H_{25}NO$, 248.2014; found: 248.2016.

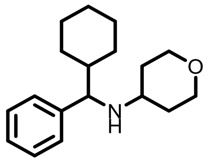

***N-(cyclohexyl(phenyl)methyl)tetrahydro-2H-pyran-4-amine* (46).** Prepared according to GP5 from **1c** (51 mg, 0.25 mmol, 1.0 equiv.) and iodocyclohexane (97 µL, 0.75 mmol, 3 equiv.). Purified via flash column chromatography on silica gel (Pentane:Ethyl Acetate 3:1) to afford the product as a colorless oil (46 mg, 67% yield).

^{1}H NMR (400 MHz, $CDCl_3$) δ 7.38 – 7.23 (m, 5H), 3.99 – 3.83 (m, 2H), 3.52 (d, J = 7.2 Hz, 1H), 3.28 (qd, J = 11.6, 2.5 Hz, 2H), 2.44 (ddd, J = 10.3, 6.2, 4.1 Hz, 1H), 2.04 – 1.84 (m, 2H), 1.82 – 1.72 (m, 1H), 1.70 – 1.60 (m, 2H), 1.53 – 1.44 (m, 1H), 1.43 – 0.67 (m, 10H).
^{13}C NMR (101 MHz, $CDCl_3$) δ 143.7, 128.1, 128.0, 126.8, 67.1, 66.8, 64.9, 50.9, 44.6, 35.0, 33.4, 30.5, 30.1, 26.7, 26.4, 26.4.
HRMS (GC-FI+) (m/z): $[M+H]^+$ calcd. for $C_{18}H_{27}NO$, 274.2171; found: 274.2176.

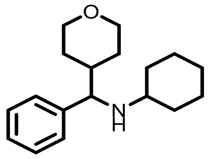

***N-(phenyl(tetrahydro-2H-pyran-4-yl)methyl)cyclohexanamine* (47).** Prepared according to GP5 from **1a** (51 mg, 0.25 mmol, 1.0 equiv.) and 4-iodotetrahydro-2H-pyran (159 mg, 0.25 mmol, 1.0 equiv.). Purified via flash column chromatography on silica gel (Pentane:Ethyl Acetate 4:1, 0.5 vol% triethylamine) to afford the product as a colorless oil (40 mg, 58% yield).

^{1}H NMR (400 MHz, $CDCl_3$) δ 7.41 – 7.27 (m, 2H), 7.25 – 7.18 (m, 3H), 4.04 – 3.93 (m, 1H), 3.90 – 3.79 (m, 1H), 3.45 (d, J = 7.9 Hz, 1H), 3.34 (td, J = 11.8, 2.2 Hz, 1H), 3.23 (td, J = 11.6, 2.4 Hz, 1H), 2.23 – 2.09 (m, 1H), 2.02 – 1.82 (m, 2H), 1.74 – 1.57 (m, 4H), 1.52 – 1.45 (m, 1H), 1.43 – 0.89 (m, 9H).
^{13}C NMR (101 MHz, $CDCl_3$) δ 143.4, 128.3, 127.8, 126.9, 68.2, 68.1, 65.0, 53.4, 42.2, 35.0, 32.9, 30.9, 30.4, 26.3, 25.2, 24.8.
HRMS (GC-FI+) (m/z): $[M+H]^+$ calcd. for $C_{18}H_{27}NO$, 274.2171; found: 274.2175.

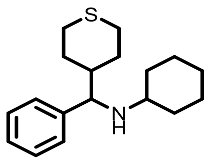

***N-(phenyl(tetrahydro-2H-thiopyran-4-yl)methyl)cyclohexanamine* (48).** Prepared according to GP5 from **1a** (51 mg, 0.25 mmol, 1.0 equiv.) and 4-iodotetrahydro-2H-thiopyran (171 mg, 0.75 mmol, 3.0 equiv.). Purified via flash column chromatography on silica gel (Pentane:Ethyl Acetate 10:1, 0.5 vol% triethylamine) to afford the product as a colorless oil (42 mg, 58% yield).

^{1}H NMR (^{1}H NMR (400 MHz, $CDCl_3$) δ 7.35 – 7.27 (m, 2H), 7.25 – 7.16 (m, 3H), 3.51 (d, J = 6.7 Hz, 1H), 2.68 – 2.45 (m, 4H), 2.38 – 2.23 (m, 1H), 2.22 – 2.11 (m, 1H), 1.99 – 1.85 (m, 1H), 1.80 – 1.68 (m, 1H), 1.68 – 1.56 (m, 3H), 1.57 – 1.46 (m, 2H), 1.45 – 1.21 (m, 3H), 1.17 – 0.91 (m, 6H).
^{13}C NMR (101 MHz, $CDCl_3$) δ 143.4, 128.3, 127.9, 126.9, 64.8, 53.6, 44.3, 34.9, 33.0, 31.5, 31.4, 29.1, 29.0, 26.3, 25.2, 24.8.
HRMS (GC-FI+) (m/z): $[M+H]^+$ calcd. for $C_{18}H_{27}NS$, 290.1942; found: 290.1949.

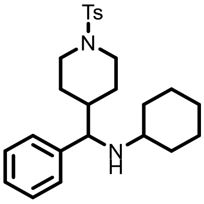

***N-(phenyl(1-tosylpiperidin-4-yl)methyl)cyclohexanamine* (49).** Prepared according to GP5 from **1a** (51 mg, 0.25 mmol, 1.0 equiv.) and 4-iodo-1-tosylpiperidine (273 mg, 0.75 mmol, 3.0 equiv.). Purified via flash column chromatography on silica gel (Pentane:Ethyl Acetate 4:1, 0.5 vol% triethylamine) to afford the product as a colorless oil (48 mg, 45% yield).

^{1}H NMR (400 MHz, $CDCl_3$) δ 7.62 – 7.55 (m, 2H), 7.31 – 7.19 (m, 6H), 7.17 – 7.11 (m, 2H), 3.80 (d, *J* = 11.5 Hz, 1H), 3.67 (d, *J* = 11.6 Hz, 1H), 3.43 (d, *J* = 5.8 Hz, 1H), 2.41 (s, 3H), 2.22 – 2.12 (m, 2H), 2.12 – 2.00 (m, 2H), 1.87 (d, *J* = 12.3 Hz, 1H), 1.68 – 1.54 (m, 3H), 1.49 (s, 1H), 1.40 – 1.17 (m, 5H), 1.16 – 0.91 (m, 5H). *One aromatic carbon not visible.*
^{13}C NMR (101 MHz, $CDCl_3$) δ 143.5, 133.2, 129.6, 128.4, 127.8, 127.7, 127.2, 64.2, 53.6, 46.7, 46.5, 42.3, 34.8, 32.7, 29.1, 28.8, 26.2, 25.1, 24.7, 21.6.
HRMS (GC-FI+) (m/z): $[M+H]^+$ calcd. for $C_{25}H_{34}N_2O_2S$, 427.2419; found: 427.2411.

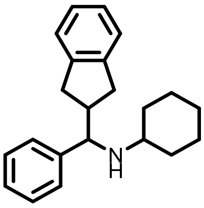

***N-((2,3-dihydro-1H-inden-2-yl)(phenyl)methyl)cyclohexanamine* (50).** Prepared according to GP5 from **1a** (51 mg, 0.25 mmol, 1.0 equiv.) and 2-iodo-2,3-dihydro-1H-indene (183 mg, 0.75 mmol, 3.0 equiv.). Purified via flash column chromatography on silica gel (Pentane:Ethyl Acetate 10:1, 0.5 vol% triethylamine) to afford the product as a colorless oil (40 mg, 53% yield).

^{1}H NMR (400 MHz, $CDCl_3$) δ 7.41 – 7.30 (m, 4H), 7.29 – 7.23 (m, 1H), 7.22 – 7.16 (m, 1H), 7.16 – 7.04 (m, 3H), 3.72 (d, *J* = 9.1 Hz, 1H), 3.19 (dd, *J* = 15.6, 7.9 Hz, 1H), 2.89 (dd, *J* = 15.6, 8.7 Hz, 1H), 2.69 (h, *J* = 8.6 Hz, 1H), 2.60 – 2.49 (m, 2H), 2.35 – 2.14 (m, 1H), 2.07 – 1.94 (m, 1H), 1.77 – 1.35 (m, 5H), 1.22 – 0.99 (m, 5H).
^{13}C NMR (101 MHz, $CDCl_3$) δ 144.5, 143.2, 128.4, 127.7, 127.0, 126.3, 124.6, 124.4, 65.0, 53.4, 47.4, 37.6, 37.2, 35.1, 32.9, 26.3, 25.3, 24.8.
HRMS (GC-FI+) (m/z): $[M+H]^+$ calcd. for $C_{22}H_{27}N$, 306.2222; found: 306.2226.

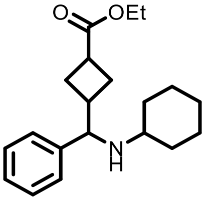

***ethyl 3-((cyclohexylamino)(phenyl)methyl)cyclobutane-1-carboxylate* (51).** Prepared according to GP5 from **1a** (51 mg, 0.25 mmol, 1.0 equiv.) and ethyl 3-iodocyclobutane-1-carboxylate (191 mg, 0.75 mmol, 3.0 equiv.). Purified via flash column chromatography on silica gel (Pentane:Ethyl Acetate 10:1, 0.5 vol% triethylamine) to afford the two diastereomers as an inseparable mixture (*d.r.* 2:1) as a colorless oil (40 mg, 51% yield).

^{1}H NMR (400 MHz, $CDCl_3$) δ 7.31 – 7.17 (m, 10H, *major* + *minor*), 4.09 (q, *J* = 2.2 Hz, 4H, *major* + *minor*), 3.64 (d, *J* = 3.9 Hz, 2H, *major* + *minor*), 3.01 – 2.91 (m, 1H, *minor*), 2.91 – 2.78 (m, 1H, *major*), 2.49 – 1.77 (m, 14H, *major* + *minor*), 1.69 – 1.54 (m, 6H, *major* + *minor*), 1.54 – 1.45 (m, 2H, *major* + *minor*), 1.27 – 1.19 (m, 8H, *major* + *minor*), 1.16 – 0.89 (m, 10H, *major* + *minor*).
^{13}C NMR (101 MHz, $CDCl_3$) δ 176.1, 175.4, 128.3, 128.3, 127.6 (2C), 127.4 (2C), 127.0 (2C), 65.8, 65.0, 60.5 (2C), 53.5, 53.5, 39.0, 38.3, 35.0, 34.4, 33.7, 33.1, 33.0, 30.2, 29.0, 28.8, 27.7, 26.3, 25.3, 25.3, 24.9, 24.9, 14.4 (2C).
HRMS (ESI+) (m/z): $[M+H]^+$ calcd. for $C_{20}H_{29}NO_2$, 316.2277; found: 316.2272.

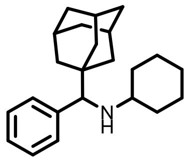

***N-(((3r,5r,7r)-adamantan-1-yl)(phenyl)methyl)cyclohexanamine* (52).** Prepared according to GP5 from **1a** (51 mg, 0.25 mmol, 1.0 equiv.) and 2-iodoadamantane (197 mg, 0.75 mmol, 3.0 equiv.). Purified via flash column chromatography on silica gel (Pentane:Ethyl Acetate 30:1) to afford the product as a colorless oil (60 mg, 74% yield).

^{1}H NMR (400 MHz, $CDCl_3$) δ 7.37 – 7.15 (m, 5H), 3.33 – 3.19 (m, 1H), 2.13 – 2.05 (m, 1H), 1.95 – 1.86 (m, 4H), 1.70 – 1.50 (m, 13H), 1.45 – 1.34 (m, 3H), 1.23 – 0.92 (m, 6H).
^{13}C NMR (101 MHz, $CDCl_3$) δ 142.2, 129.3, 127.3, 126.4, 70.1, 53.9, 39.6, 37.3, 36.4, 35.1, 32.7, 28.8, 26.4, 25.2, 24.8.
HRMS (GC-FI+) (m/z): $[M+H]^+$ calcd. for $C_{23}H_{33}N$, 324.2686; found: 324.2682.

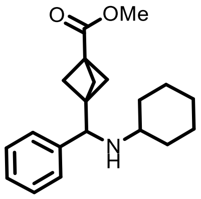

***methyl 3-((cyclohexylamino)(phenyl)methyl)bicyclo[1.1.1]pentane-1-carboxylate* (53).** Prepared according to GP5 from **1a** (51 mg, 0.25 mmol, 1.0 equiv.) and methyl 3-iodobicyclo[1.1.1]pentane-1-carboxylate (189 mg, 0.25 mmol, 3.0 equiv.). Purified via flash column chromatography on silica gel (gradient from Heptane:Ethyl Acetate 90:10 to 40:60) to afford the product as a pale yellow solid (60 mg, 75% yield).

^{1}H NMR (400 MHz, $CDCl_3$) δ 7.32 – 7.24 (m, 2H), 7.23 – 7.15 (m, 3H), 3.85 (s, 1H), 3.60 (s, 3H), 2.25 (tt, J = 10.0, 3.8 Hz, 1H), 1.95 – 1.87 (m, 1H), 1.82 (dd, J = 9.4, 1.7 Hz, 3H), 1.76 (dd, J = 9.5, 1.7 Hz, 3H), 1.72 – 1.57 (m, 3H), 1.55 – 1.45 (m, 1H), 1.40 – 1.19 (m, 1H), 1.17 – 0.90 (m, 5H).
^{13}C NMR (101 MHz, $CDCl_3$) δ 170.8, 141.7, 128.2, 127.3, 127.0, 59.9, 54.1, 51.6, 49.7, 43.5, 38.0, 34.8, 33.0, 26.2, 25.3, 25.0.
HRMS (ESI+) (m/z): $[M+H]^+$ calcd. for $C_{20}H_{27}NO_2$, 314.2120; found: 314.2118.

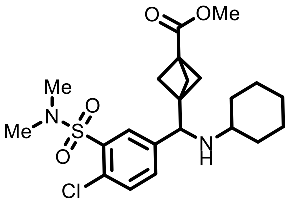

***methyl 3-((4-chloro-3-(N,N-dimethylsulfamoyl)phenyl)(cyclohexylamino)methyl)bicyclo[1.1.1]pentane-1-carboxylate* (54).** Prepared according to GP5 from **2g** (86 mg, 0.25 mmol, 1.0 equiv.) and methyl 3-iodobicyclo[1.1.1]pentane-1-carboxylate (189 mg, 0.25 mmol, 3.0 equiv.). Purified via flash column chromatography on silica gel (gradient from Heptane:Ethyl Acetate 80:20 to 30:70) to afford the product as a pale yellow oil (40 mg, 35% yield).

^{1}H NMR (400 MHz, $CDCl_3$) δ 7.89 (d, J = 2.1 Hz, 1H), 7.46 (d, J = 8.1 Hz, 1H), 7.39 (dd, J = 8.2, 2.1 Hz, 1H), 3.92 (s, 1H), 3.62 (s, 3H), 2.88 (s, 6H), 2.18 (tt, J = 10.1, 3.6 Hz, 1H), 1.88 (d, J = 12.6 Hz, 1H), 1.81 (dd, J = 9.4, 1.7 Hz, 3H), 1.76 (dd, J = 9.5, 1.7 Hz, 3H), 1.69 – 1.58 (m, 3H), 1.56 – 1.50 (m, 1H), 1.36 – 1.19 (m, 1H), 1.17 – 0.91 (m, 5H).
^{13}C NMR (101 MHz, $CDCl_3$) δ 170.4, 141.9, 135.7, 132.3, 132.1, 131.1, 130.5, 59.3, 54.6, 51.8, 49.6, 43.1, 38.1, 37.6, 34.9, 33.2, 26.1, 25.3, 25.0.
HRMS (ESI+) (m/z): $[M+H]^+$ calcd. for $C_{22}H_{31}ClN_2O_4S$, 455.1771; found: 455.1766.

11.4 Characterization of compounds 55-63 (drug-like sec-amides)

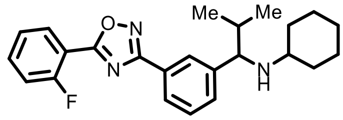

***N-(1-(3-(5-(2-fluorophenyl)-1,2,4-oxadiazol-3-yl)phenyl)-2-methylpropyl)cyclohexanamine* (55).** Prepared according to GP5 from **2t** (91 mg, 0.25 mmol, 1.0 equiv.). Purified via flash column chromatography on silica gel (Pentane:Ethyl Acetate 30:1) to afford the product as a colorless oil (46 mg, 47% yield).

^{1}H NMR (300 MHz, $CDCl_3$) δ 8.24 (td, J = 7.4, 1.8 Hz, 1H), 8.11 – 8.01 (m, 2H), 7.66 – 7.55 (m, 1H), 7.54 – 7.42 (m, 2H), 7.39 – 7.27 (m, 2H), 3.61 (d, J = 6.8 Hz, 1H), 2.35 – 2.17 (m, 1H), 2.07 – 1.84 (m, 2H), 1.77 – 1.58 (m, 3H), 1.57 – 1.46 (m, 1H), 1.36 – 1.03 (m, 6H), 0.99 (d, J = 6.7 Hz, 3H), 0.79 (d, J = 6.8 Hz, 3H).
^{13}C NMR (101 MHz, $CDCl_3$) δ 172.8 (d, J = 4.3 Hz), 169.1, 160.9 (d, J = 260.4 Hz), 134.6 (d, J = 8.6 Hz), 131.1 (2C), 131.0, 128.7, 127.3, 126.5, 126.2, 124.8 (d, J = 3.8 Hz), 117.3 (d, J = 20.9 Hz), 113.1 (d, J = 11.4 Hz), 66.0, 54.1, 34.8, 34.6, 32.8, 26.2, 25.3, 24.9, 19.8, 19.7.
^{19}F NMR (282 MHz, $CDCl_3$) δ -108.38.

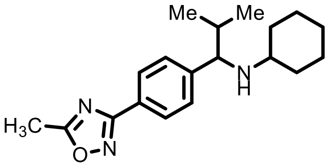

***N-(2-methyl-1-(4-(5-methyl-1,2,4-oxadiazol-3-yl)phenyl)propyl)cyclohexanamine* (56).** Prepared according to GP5 from **2u** (71 mg, 0.25 mmol, 1.0 equiv.). Purified via flash column chromatography on silica gel (gradient from Heptane:Ethyl Acetate 95:5 to 60:40) to afford the product as a pale yellow oil (42 mg, 53% yield).

^{1}H NMR (400 MHz, $CDCl_3$) δ 8.02 – 7.94 (m, 2H), 7.40 – 7.32 (m, 2H), 3.52 (d, J = 6.8 Hz, 1H), 2.64 (s, 3H), 2.16 (tt, J = 9.8, 3.5 Hz, 1H), 1.93 (d, J = 12.7 Hz, 1H), 1.88 – 1.75 (m, J = 6.8 Hz, 1H), 1.72 – 1.56 (m, 3H), 1.55 – 1.45 (m, 1H), 1.38 – 1.30 (m, 1H), 1.18 – 0.97 (m, 5H), 0.94 (d, J = 6.7 Hz, 3H), 0.74 (d, J = 6.8 Hz, 3H).
^{13}C NMR (101 MHz, $CDCl_3$) δ 176.5, 168.6, 147.9, 128.6, 127.1, 125.2, 66.0, 53.9, 35.0, 34.8, 33.0, 26.3, 25.3, 24.9, 19.7, 19.6, 12.5.
HRMS (ESI+) (m/z): $[M+H]^+$ calcd. for $C_{19}H_{27}N_3O$, 314.2232; found: 314.2238.

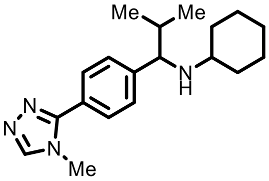

***N-(2-methyl-1-(4-(4-methyl-4H-1,2,4-triazol-3-yl)phenyl)propyl)cyclohexanamine* (57).** Prepared according to GP5 from **2v** (71 mg, 0.25 mmol, 1.0 equiv.). Purified via flash column chromatography on silica gel [gradient from Dichloromethane:(Dichloromethane:Methanol 90:10) 95:5 to 0:100] to afford the product as a pale yellow solid (39 mg, 50% yield).

^{1}H NMR (400 MHz, $CDCl_3$) δ 8.21 (s, 1H), 7.65 – 7.58 (m, 2H), 7.45 – 7.38 (m, 2H), 3.78 (s, 3H), 3.58 (d, J = 6.8 Hz, 1H), 2.21 (ddt, J = 9.8, 6.0, 3.7 Hz, 1H), 1.99 – 1.91 (m, 1H), 1.91 – 1.81 (m, 1H), 1.70 – 1.58 (m, 3H), 1.57 – 1.48 (m, 1H), 1.30 – 1.21 (m, 1H), 1.17 – 0.99 (m, 5H), 0.96 (d, J = 6.7 Hz, 3H), 0.76 (d, J = 6.8 Hz, 3H).
^{13}C NMR (101 MHz, $CDCl_3$) δ 154.5, 146.0, 145.3, 128.7, 128.4, 125.0, 65.9, 54.1, 34.7, 34.6, 32.7, 32.4, 26.2, 25.3, 24.9, 19.7, 19.6.
HRMS (ESI+) (m/z): $[M+H]^+$ calcd. for $C_{19}H_{28}N_4$, 313.2392; found: 313.2396.

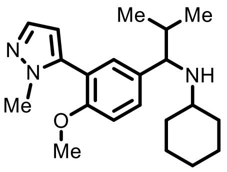

N-(1-(4-methoxy-3-(1-methyl-1H-pyrazol-5-yl)phenyl)-2-methylpropyl)cyclohexanamine **(58).** Prepared according to GP5 from **2z** (78 mg, 0.25 mmol, 1.0 equiv.). Purified via flash column chromatography on silica gel [gradient from Dichloromethane:(Dichloromethane:Methanol 90:10) 95:5 to 0:100] to afford the product as a yellow oil (52 mg, 61% yield).

^{1}H NMR (400 MHz, $CDCl_3$) δ 7.52 (d, *J* = 1.9 Hz, 1H), 7.29 (dd, *J* = 8.5, 2.3 Hz, 1H), 7.13 (d, *J* = 2.2 Hz, 1H), 6.92 (d, *J* = 8.5 Hz, 1H), 6.25 (d, *J* = 1.9 Hz, 1H), 3.81 (s, 3H), 3.73 (s, 3H), 3.47 (d, *J* = 6.7 Hz, 1H), 2.21 (tt, *J* = 9.9, 3.5 Hz, 1H), 1.98 – 1.90 (m, 1H), 1.86 – 1.73 (m, *J* = 6.8 Hz, 1H), 1.73 – 1.40 (m, 5H), 1.20 – 0.96 (m, 5H), 0.94 (d, *J* = 6.7 Hz, 3H), 0.74 (d, *J* = 6.8 Hz, 3H).
^{13}C NMR (101 MHz, $CDCl_3$) δ 155.7, 140.6, 138.3, 135.9, 131.2, 129.8, 119.1, 110.7, 106.8, 65.2, 55.6, 53.8, 37.3, 34.9, 34.7, 32.9, 26.3, 25.3, 24.9, 19.8, 19.5.
HRMS (ESI+) (m/z): $[M+H]^+$ calcd. for $C_{21}H_{31}N_3O$, 342.2545; found: 342.2546.

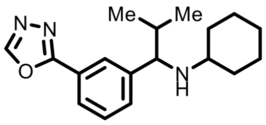

N-(1-(3-(1,3,4-oxadiazol-2-yl)phenyl)-2-methylpropyl)cyclohexanamine **(59).** Prepared according to GP5 from **2aa** (68 mg, 0.25 mmol, 1.0 equiv.). Purified via flash column chromatography on silica gel (gradient from Heptane:Ethyl Acetate 90:10 to 50:50) to afford the product as a pale yellow oil (30 mg, 40% yield).

^{1}H NMR (400 MHz, $CDCl_3$) δ 8.46 (s, 1H), 7.98 (d, *J* = 1.8 Hz, 1H), 7.93 (dt, *J* = 6.9, 1.8 Hz, 1H), 7.50 – 7.41 (m, 2H), 2.16 (tt, *J* = 9.7, 3.5 Hz, 1H), 1.99 – 1.89 (m, 1H), 1.89 – 1.74 (m, *J* = 6.7 Hz, 1H), 1.71 – 1.55 (m, 4H), 1.55 – 1.46 (m, 1H), 1.29 – 1.22 (m, 1H), 1.17 – 0.97 (m, 5H), 0.94 (d, *J* = 6.7 Hz, 3H), 0.75 (d, *J* = 6.8 Hz, 3H).
^{13}C NMR (101 MHz, $CDCl_3$) δ 165.2, 152.6, 145.8, 131.8, 128.8, 126.7, 125.5, 123.2, 65.9, 54.0, 35.0, 34.8, 33.0, 26.3, 25.3, 24.9, 19.6, 19.6.
HRMS (ESI+) (m/z): $[M+H]^+$ calcd. for $C_{18}H_{25}N_3O$, 300.2076; found: 300.2080.

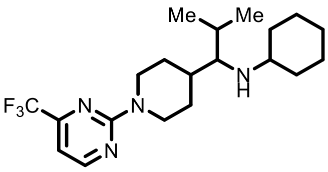

N-(2-methyl-1-(1-(4-(trifluoromethyl)pyrimidin-2-yl)piperidin-4-yl)propyl)cyclohexanamine **(60).** Prepared according to GP6 from **2ab** (89 mg, 0.25 mmol, 1.0 equiv.). Purified via flash column chromatography on silica gel (gradient from Pentane:Ethyl Acetate 90:10 to 70:30) to afford the product as a yellow oil (34 mg, 35% yield).

^{1}H NMR (400 MHz, $CDCl_3$) δ 8.45 (d, *J* = 4.8 Hz, 1H), 6.67 (d, *J* = 4.8 Hz, 1H), 4.91 – 4.78 (m, 2H), 2.89 – 2.76 (m, 2H), 2.35 (ddt, *J* = 10.6, 7.4, 3.8 Hz, 1H), 2.11 (t, *J* = 5.6 Hz, 1H), 1.95 – 1.80 (m, 3H), 1.75 – 1.53 (m, 5H), 1.42 – 0.98 (m, 9H), 0.95 (d, *J* = 6.8 Hz, 3H), 0.89 (d, *J* = 6.8 Hz, 3H).
^{13}C NMR (101 MHz, $CDCl_3$) δ 161.4, 160.1, 156.36 (q, *J* = 35.2 Hz), 120.81 (q, *J* = 275.2 Hz), 103.85 (q, *J* = 2.8 Hz), 64.3, 57.5, 44.5, 44.4, 40.6, 34.8, 34.6, 30.3, 30.1, 28.6, 26.2, 25.6, 25.5, 21.2, 17.6.
^{19}F NMR (282 MHz, $CDCl_3$) δ -70.9.
HRMS (ESI+) (m/z): $[M+H]^+$ calcd. for $C_{20}H_{31}F_3N_4$, 385.2579; found: 385.2580.

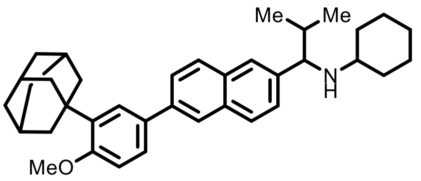

N-(1-(6-(3-(adamantan-1-yl)-4-methoxyphenyl)naphthalen-2-yl)-2-methylpropyl)cyclohexanamine **(61).** Prepared according to GP6 from **2ac** (123 mg, 0.25 mmol, 1.0 equiv.). Purified via flash column chromatography on silica gel (gradient from Pentane:Ethyl Acetate 95:5 to 70:30) to afford the product as a white solid (46 mg, 35% yield).

^{1}H NMR (400 MHz, $CDCl_3$) δ 7.99 (s, 1H), 7.89 (dd, J = 8.6, 3.8 Hz, 2H), 7.79 – 7.72 (m, 2H), 7.59 (s, 1H), 7.56 – 7.49 (m, 2H), 7.00 (d, J = 8.4 Hz, 1H), 3.91 (s, 3H), 3.77 (d, J = 7.1 Hz, 1H), 2.43 – 2.31 (m, 1H), 2.20 (s, 6H), 2.16 – 1.99 (m, 5H), 1.88 – 1.75 (m, 7H), 1.74 – 1.59 (m, 2H), 1.56 – 1.48 (m, 1H), 1.36 – 1.10 (m, 6H), 1.07 (d, J = 6.6 Hz, 3H), 0.79 (d, J = 6.7 Hz, 3H).
^{13}C NMR (101 MHz, $CDCl_3$) δ 158.7, 139.1, 139.0, 133.4, 133.4, 132.1, 128.4, 128.3, 127.1, 127.0, 126.4, 126.1, 126.1, 125.7, 125.0, 112.2, 66.4, 55.3, 54.4, 40.8, 37.3, 37.3, 34.3, 34.1, 29.3, 26.0, 25.2, 24.8, 22.5, 20.2, 19.6.
HRMS (ESI+) (m/z): $[M+H]^+$ calcd. for $C_{37}H_{47}NO$, 522.3736; found: 522.3738.

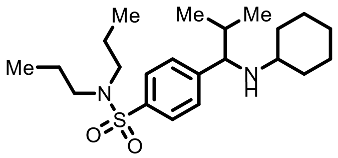

4-(1-(cyclohexylamino)-2-methylpropyl)-N,N-dipropylbenzenesulfonamide **(62).** Prepared according to GP5 from **2ad** (93 mg, 0.25 mmol, 1.0 equiv.). Purified via flash column chromatography on silica gel (gradient from Pentane:Ethyl Acetate 90:10 to 80:20) to afford the product as a colorless oil (62 mg, 63% yield).

^{1}H NMR (400 MHz, $CDCl_3$) δ 7.72 (dd, J = 8.4, 1.6 Hz, 2H), 7.41 – 7.34 (m, 2H), 3.53 (d, J = 6.7 Hz, 1H), 3.14 – 3.05 (m, 4H), 2.10 (tt, J = 9.8, 3.6 Hz, 1H), 1.91 (d, J = 12.6 Hz, 1H), 1.85 – 1.74 (m, 1H), 1.71 – 1.46 (m, 8H), 1.39 – 1.22 (m, 1H), 1.16 – 0.95 (m, 5H), 0.92 (d, J = 6.6 Hz, 3H), 0.84 (t, J = 7.4 Hz, 6H), 0.71 (d, J = 6.7 Hz, 3H).
^{13}C NMR (101 MHz, $CDCl_3$) δ 149.4, 138.5, 128.6, 126.8, 65.9, 54.1, 49.9, 35.0, 34.7, 33.0, 26.2, 25.3, 24.9, 22.0, 19.6, 19.6, 11.3.
HRMS (ESI+) (m/z): $[M+H]^+$ calcd. for $C_{22}H_{38}N_2O_2S$, 395.2732; found: 395.2737.

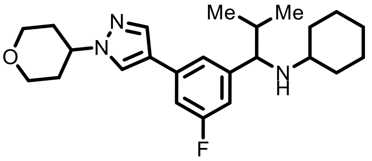

N-(1-(3-fluoro-5-(1-(tetrahydro-2H-pyran-4-yl)-1H-pyrazol-4-yl)phenyl)-2-methylpropyl)cyclohexanamine **(63).** Prepared according to GP5 from **2ae** (93 mg, 0.25 mmol, 1.0 equiv.). Purified via flash column chromatography on silica gel (gradient from Heptane:Ethyl Acetate 80:20 to 20:80) to afford the product as a colorless oil (60 mg, 60% yield).

^{1}H NMR (400 MHz, $CDCl_3$) δ 7.78 (s, 1H), 7.69 (s, 1H), 7.14 (d, J = 1.5 Hz, 1H), 7.02 (ddd, J = 9.7, 2.5, 1.5 Hz, 1H), 6.85 (ddd, J = 9.8, 2.5, 1.3 Hz, 1H), 4.37 (tt, J = 10.5, 5.4 Hz, 1H), 4.12 (dt, J = 12.0, 2.7 Hz, 2H), 3.61 – 3.50 (m, 2H), 3.47 (d, J = 6.8 Hz, 1H), 2.26 – 2.03 (m, 5H), 1.95 (d, J = 12.4 Hz, 1H), 1.87 – 1.73 (m, 1H), 1.70 – 1.57 (m, 3H), 1.57 – 1.48 (m, 1H), 1.40 – 1.25 (m, 1H), 1.21 – 0.97 (m, 5H), 0.95 (d, J = 6.7 Hz, 3H), 0.77 (d, J = 6.8 Hz, 3H).
^{13}C NMR (101 MHz, $CDCl_3$) δ 163.3 (d, J = 244.2 Hz), 147.9 (d, J = 7.0 Hz), 136.7, 134.1 (d, J = 8.7 Hz), 123.8, 122.3 (d, J = 2.6 Hz), 120.6 (d, J = 2.3 Hz), 112.7 (d, J = 21.2 Hz), 110.5 (d, J = 22.5 Hz), 67.0, 66.0 (d, J = 1.7 Hz), 58.5, 53.9, 35.1, 34.8, 33.5, 33.0, 26.3, 25.3, 24.9, 19.8, 19.6.
^{19}F NMR (376 MHz, $CDCl_3$) δ -114.2.
HRMS (ESI+) (m/z): $[M+H]^+$ calcd. for $C_{24}H_{34}FN_3O$, 400.2764; found: 400.2762.

11.5 Characterization of compounds 64-71 (cyclization strategy)

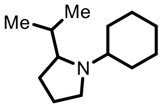

***1-cyclohexyl-2-isopropylpyrrolidine* (64).** Prepared according to GP5 from **3a** (51 mg, 0.25 mmol, 1.0 equiv.). Purified via flash column chromatography on silica gel (Pentane:Ethyl Acetate 20:1, 0.5 vol% triethylamine) to afford the product as a colorless oil (37 mg, 75% yield).

^{1}H NMR (400 MHz, MeOD) δ 2.90 – 2.83 (m, 1H), 2.65 (dd, *J* = 6.8, 4.7 Hz, 1H), 2.60 – 2.43 (m, 2H), 1.87 – 1.71 (m, 5H), 1.67 – 1.52 (m, 4H), 1.38 – 1.06 (m, 6H), 0.89 (d, *J* = 6.9 Hz, 3H), 0.84 (d, *J* = 6.8 Hz, 3H).
^{13}C NMR (101 MHz, MeOD) δ 66.4, 60.7, 33.9, 31.6, 27.5, 26.9, 26.8, 26.4, 24.4, 21.1, 16.6.
HRMS (ESI+) (m/z): $[M+H]^+$ calcd. for $C_{13}H_{25}N$, 196.2065; found: 196.2068

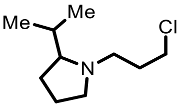

***1-(3-chloropropyl)-2-isopropylpyrrolidine* (65).** Prepared according to GP5 from **3b** (50 mg, 0.25 mmol, 1.0 equiv.). Purified via flash column chromatography on silica gel (from Pentane:Ethyl Acetate 5:1 to 1:1, 0.5 vol% triethylamine) to afford the product as a colorless oil (23 mg, 48% yield).

^{1}H NMR (300 MHz, MeOD) δ 3.63 (td, *J* = 6.7, 3.2 Hz, 2H), 3.14 – 3.06 (m, 1H), 2.96 (dt, *J* = 12.0, 8.0 Hz, 1H), 2.28 – 2.05 (m, 3H), 2.02 – 1.79 (m, 3H), 1.72 – 1.50 (m, 4H), 0.90 (d, *J* = 6.9 Hz, 3H), 0.85 (d, *J* = 6.7 Hz, 3H).
^{13}C NMR (101 MHz, MeOD) δ 71.4, 55.4, 53.0, 43.9, 32.7, 30.2, 25.5, 23.6, 20.9, 16.0.
HRMS (ESI+) (m/z): $[M+H]^+$ calcd. for $C_{10}H_{20}ClN$, 190.1363; found: 190.1367.

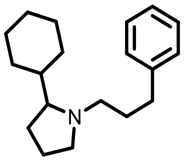

***2-cyclohexyl-1-(3-phenylpropyl)pyrrolidine* (66).** Prepared according to GP5 from **3c** (60 mg, 0.25 mmol, 1.0 equiv.) and iodocyclohexane (97 µL, 0.75 mmol, 3 equiv.). Purified via flash column chromatography on silica gel (Pentane:Ethyl Acetate 5:1, 0.5 vol% triethylamine) to afford the product as a colorless oil (35 mg, 61% yield).

^{1}H NMR (400 MHz, $CDCl_3$) δ 7.39 – 7.12 (m, 5H), 3.21 (s, 1H), 2.91 – 2.72 (m, 2H), 2.70 – 2.55 (m, 1H), 2.28 – 2.11 (m, 3H), 1.89 (d, *J* = 7.2 Hz, 2H), 1.81 (d, *J* = 10.8 Hz, 3H), 1.75 – 1.60 (m, 6H), 1.55 – 1.44 (m, 1H), 1.35 – 1.14 (m, 4H), 1.08 – 0.96 (m, 2H).
^{13}C NMR (101 MHz, $CDCl_3$) δ 142.7, 128.6, 128.4, 125.8, 69.9, 55.0, 54.5, 40.5, 33.9, 31.4, 30.4, 27.2, 27.1, 27.0, 26.6, 26.4, 23.2.
HRMS (ESI+) (m/z): $[M+H]^+$ calcd. for $C_{19}H_{29}N$, 272.2378; found: 272.2373.

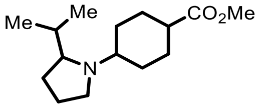

***methyl 4-(2-isopropylpyrrolidin-1-yl)cyclohexane-1-carboxylate* (67).** Prepared according to GP5 from **3d** (63 mg, 0.25 mmol, 1.0 equiv.). Purified via flash column chromatography on silica gel (Pentane:(CH_2Cl_2: MeOH 1:1),1:1) to afford the product as a colorless oil (39 mg, 62% yield).

^{1}H NMR (400 MHz, $CDCl_3$) δ 3.65 (s, 3H), 2.97 (s, 1H), 2.76 – 2.42 (m, 3H), 2.32 – 2.17 (m, 1H), 2.09 – 1.13 (m, 13H), 0.91 – 0.75 (m, 6H).
^{13}C NMR (101 MHz, $CDCl_3$) δ 176.2, 65.5, 58.7, 51.6, 48.2, 43.3, 31.8, 30.4, 29.1, 28.5, 25.8, 24.8, 23.9, 20.6, 16.4.
HRMS (ESI+) (m/z): $[M+H]^+$ calcd. for $C_{15}H_{27}NO_2$, 254.2120; found: 254.2116.

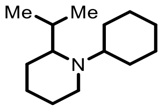

***1-cyclohexyl-2-isopropylpiperidine* (68).** Prepared according to GP7 from **3e** (54 mg, 0.25 mmol, 1.0 equiv.). Purified via flash column chromatography on silica gel (Pentane:Ethyl Acetate 6:1, 0.5 vol% triethylamine) to afford the product as a colorless oil (44 mg, 84% yield).

^{1}H NMR (400 MHz, MeOD) δ 3.09 – 3.01 (m, 1H), 2.92 – 2.82 (m, 1H), 2.39 (d, *J* = 8.8 Hz, 1H), 2.31 (td, *J* = 11.9, 2.8 Hz, 1H), 2.16 (ddq, *J* = 10.7, 6.9, 3.5 Hz, 1H), 1.86 – 1.71 (m, 4H), 1.71 – 1.58 (m, 3H), 1.58 – 1.47 (m, 2H), 1.38 – 1.00 (m, 7H), 0.92 (d, *J* = 6.9 Hz, 3H), 0.86 (d, *J* = 6.9 Hz, 3H).
^{13}C NMR (101 MHz, MeOD) δ 65.5, 58.2, 47.8, 32.2, 27.8, 27.5, 26.9, 26.7, 25.6, 24.9, 24.8, 20.7, 15.6. *One carbon not visible.*
HRMS (ESI+) (m/z): $[M+H]^+$ calcd. for $C_{14}H_{27}N$, 210.2222; found: 210.2222.

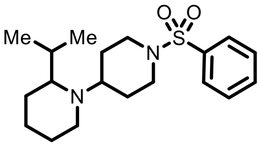

***2-isopropyl-1'-(phenylsulfonyl)-1,4'-bipiperidine* (69).** Prepared according to GP7 from **3f** (91 mg, 0.25 mmol, 1.0 equiv.). Purified via flash column chromatography on silica gel (Pentane:Ethyl Acetate 3:1, 0.5 vol% triethylamine) to afford the product as a colorless oil (62 mg, 71% yield).

^{1}H NMR (400 MHz, $CDCl_3$) δ 7.79 – 7.72 (m, 2H), 7.64 – 7.56 (m, 1H), 7.53 (dd, *J* = 8.3, 6.7 Hz, 2H), 3.86 (ddt, *J* = 13.2, 6.3, 2.9 Hz, 2H), 2.86 (dt, *J* = 11.8, 4.0 Hz, 1H), 2.66 (tt, *J* = 11.5, 3.7 Hz, 1H), 2.34 – 2.24 (m, 1H), 2.22 – 2.02 (m, 3H), 1.99 – 1.80 (m, 2H), 1.62 – 1.46 (m, 6H), 1.24 (m, 3H), 0.80 (d, *J* = 6.9 Hz, 3H), 0.73 (d, *J* = 6.8 Hz, 3H).
^{13}C NMR (101 MHz, $CDCl_3$) δ 136.5, 132.8, 129.1, 127.8, 62.9, 53.9, 47.1, 46.4, 46.2, 30.4, 26.7, 26.2, 24.6, 24.2, 23.2, 20.4, 15.6.
HRMS (ESI+) (m/z): $[M+H]^+$ calcd. for $C_{19}H_{30}N_2O_2S$, 351.2106; found: 351.2103.

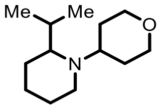

***2-isopropyl-1-(tetrahydro-2H-pyran-4-yl)piperidine* (70).** Prepared according to GP7 from **3g** (55 mg, 0.25 mmol, 1.0 equiv.). Purified via flash column chromatography on silica gel (Pentane:(CH_2Cl_2: MeOH 1:1), 4:1) to afford the product as a colorless oil (40 mg, 75% yield).

^{1}H NMR (400 MHz, MeOD) δ 4.04 – 3.90 (m, 2H), 3.56 – 3.34 (m, 2H), 3.25 – 3.01 (m, 2H), 2.43 (s, 1H), 2.32 – 2.21 (m, 1H), 1.94 – 1.43 (m, 9H), 1.35 – 1.26 (m, 2H), 0.94 (d, *J* = 6.8 Hz, 3H), 0.90 (d, *J* = 6.8 Hz, 3H).
^{13}C NMR ^{13}C NMR (101 MHz, MeOD) δ 69.0, 68.3, 65.2, 55.7, 47.7, 32.0, 27.4, 26.3, 25.9, 25.1, 24.6, 20.6, 15.8.
HRMS (ESI+) (m/z): $[M+H]^+$ calcd. for $C_{13}H_{25}NO$, 212.2014; found: 212.2018.

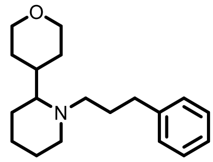

***1-(3-phenylpropyl)-2-(tetrahydro-2H-pyran-4-yl)piperidine* (71).** Prepared according to GP7 from **3h** (63 mg, 0.25 mmol, 1.0 equiv.) and 4-iodotetrahydro-2H-pyran (90 µL, 0.75 mmol, 3.0 equiv.). Purified via flash column chromatography on silica gel (Pentane:(CH_2Cl_2: MeOH 1:1), 1:2) to afford the product as a colorless oil (36 mg, 50% yield).

^{1}H NMR (400 MHz, MeOD) δ 7.30 – 7.24 (m, 2H), 7.22 – 7.12 (m, 3H), 3.92 – 3.80 (m, 2H), 3.26 (td, *J* = 11.5, 2.5 Hz, 1H), 3.12 (td, *J* = 11.6, 2.4 Hz, 1H), 2.99 – 2.91 (m, 1H), 2.75 – 2.62 (m, 2H), 2.61 – 2.48 (m, 2H), 2.42 (td, *J* = 11.6, 3.3 Hz, 1H), 2.19 – 2.07 (m, 1H), 1.90 – 1.68 (m, 4H), 1.63 – 1.16 (m, 9H).

^{13}C NMR (101 MHz, MeOD) δ 143.2, 129.7, 129.5, 127.0, 69.4, 69.0, 65.7, 53.8, 51.9, 36.8, 34.4, 31.4, 28.4, 26.5, 25.7, 25.3. *One carbon not visible.*
HRMS (ESI+) (m/z): $[M+H]^+$ calcd. for $C_{19}H_{29}NO$, 288.2327; found: 288.2322.

12. Limitation of the scope

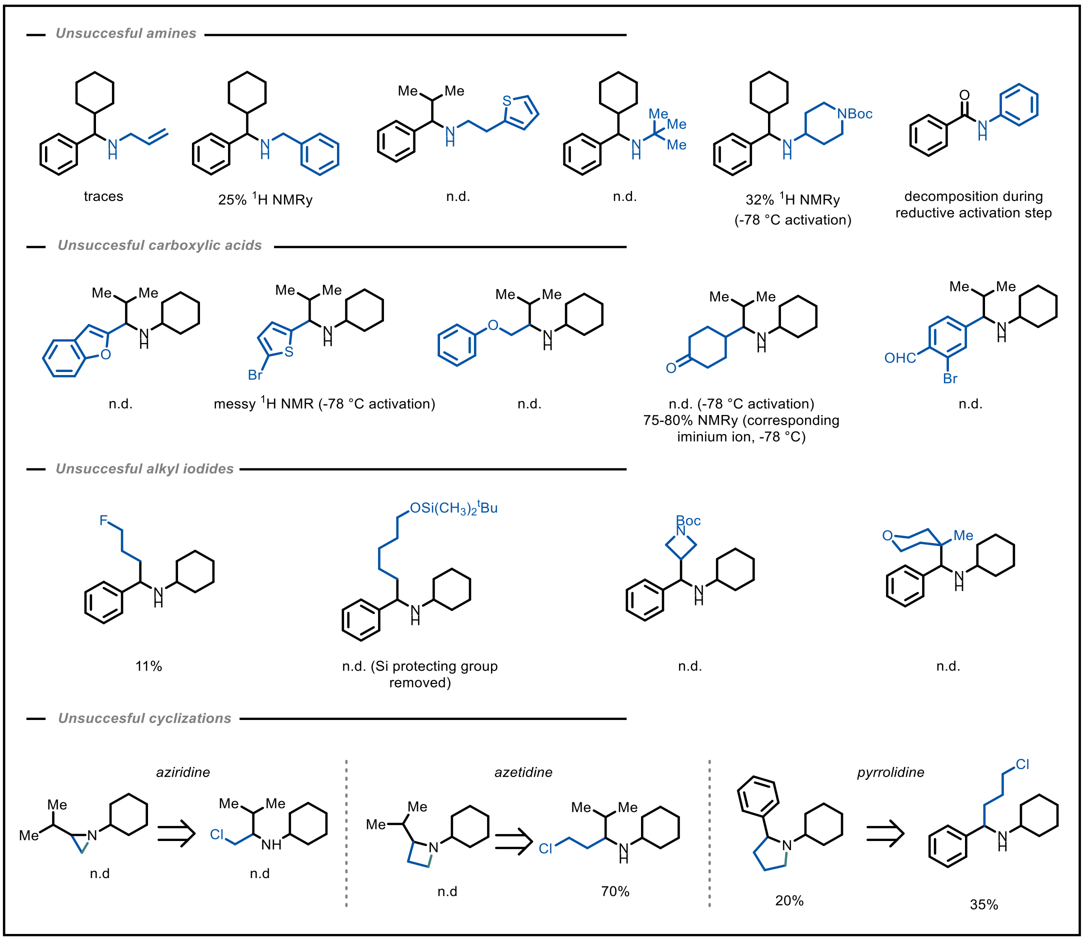

13. NMR spectra secondary amides

^{1}H NMR (400 MHz, $CDCl_3$) of **S4**

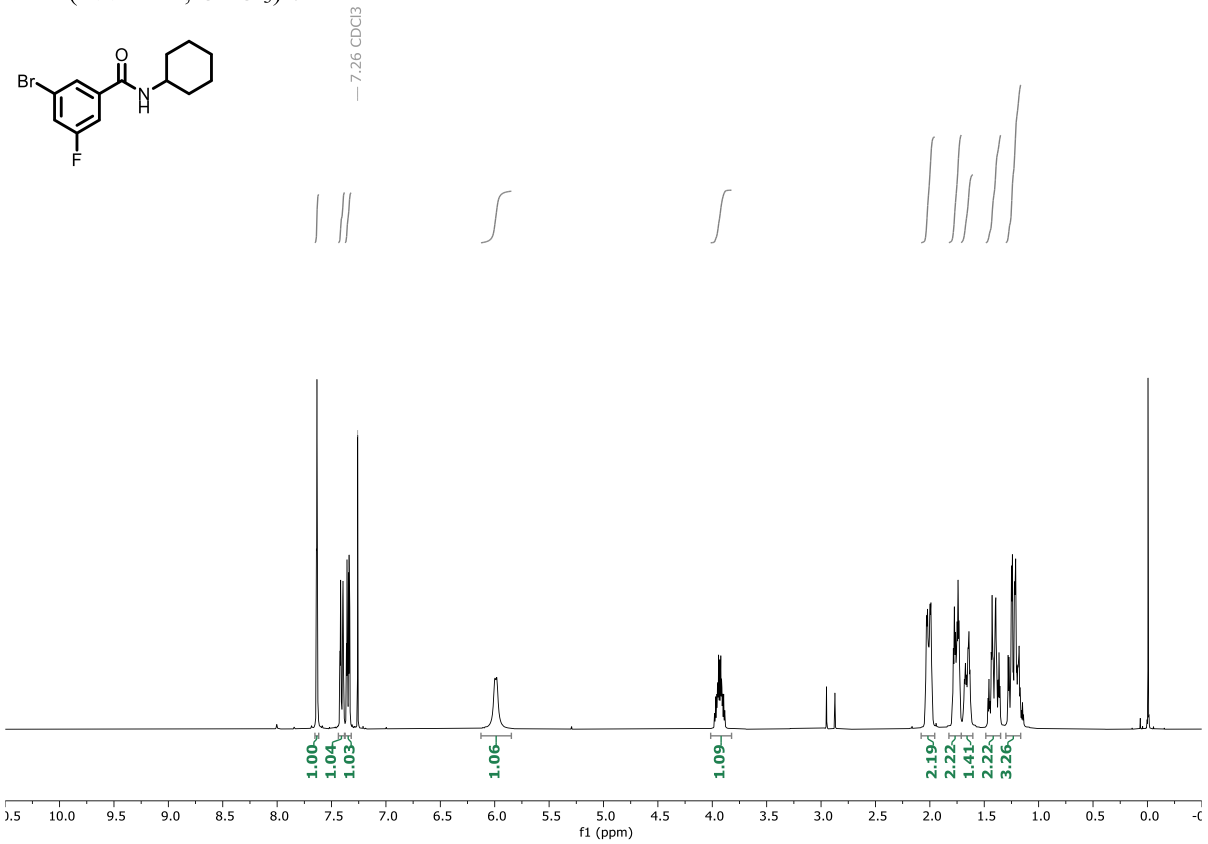

^{13}C NMR (101 MHz, $CDCl_3$) of **S4**

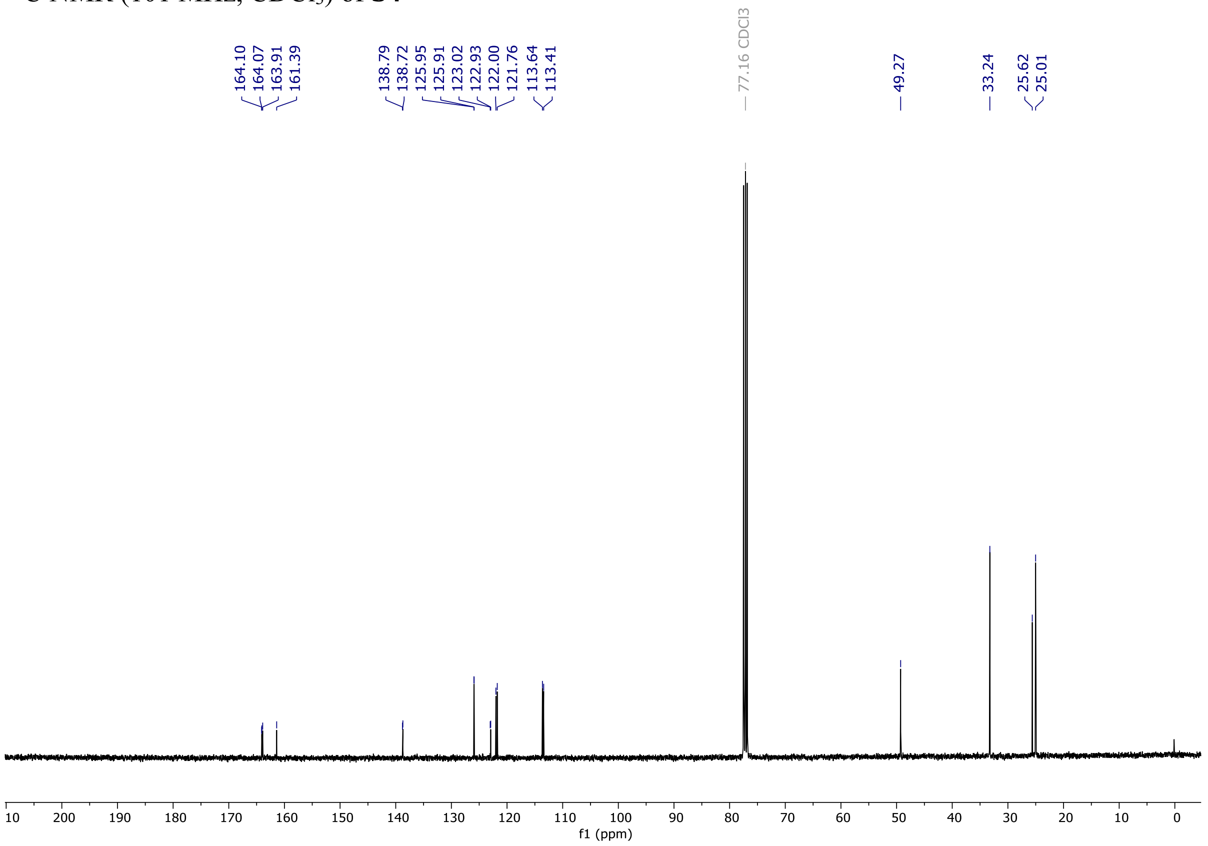

^{19}F NMR (377 MHz, $CDCl_3$) of **S4**

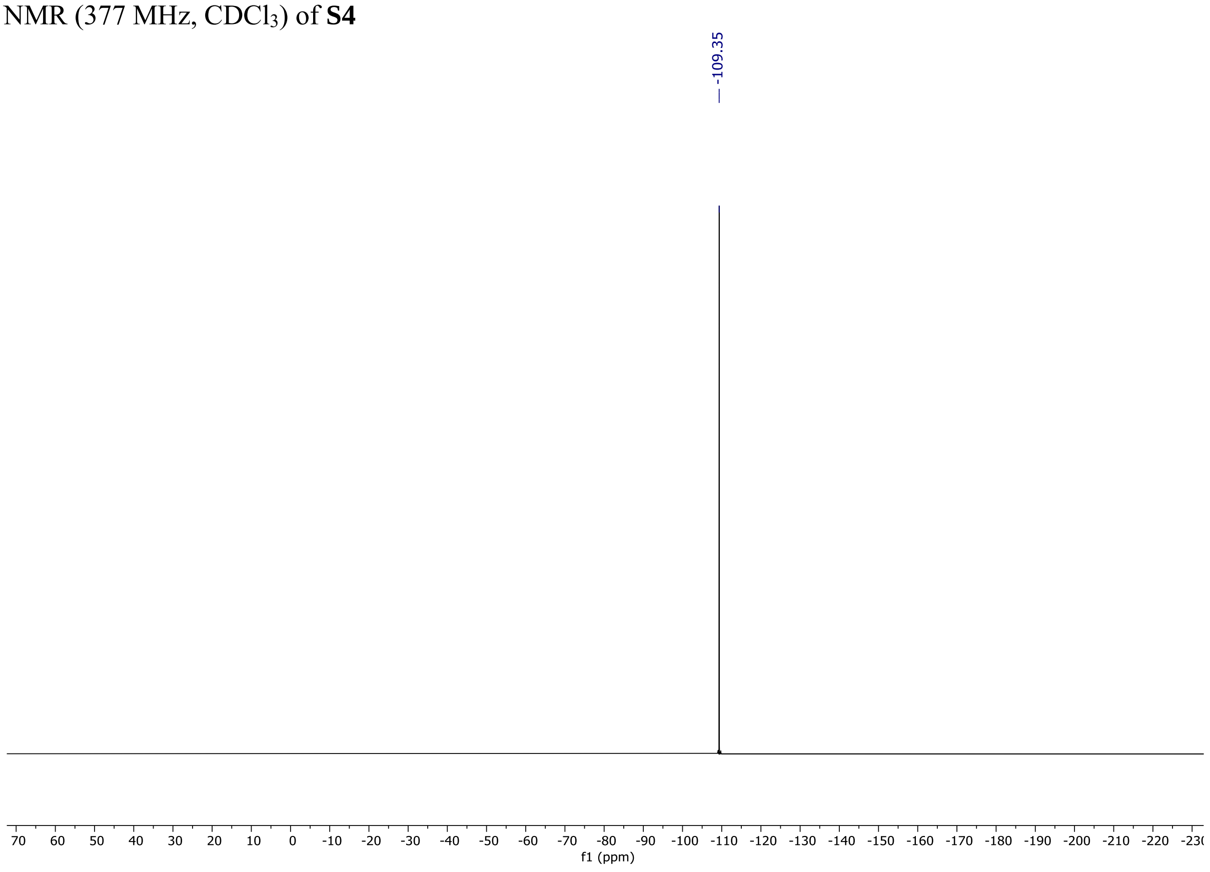

^{1}H NMR (400 MHz, $CDCl_3$) of **1d**

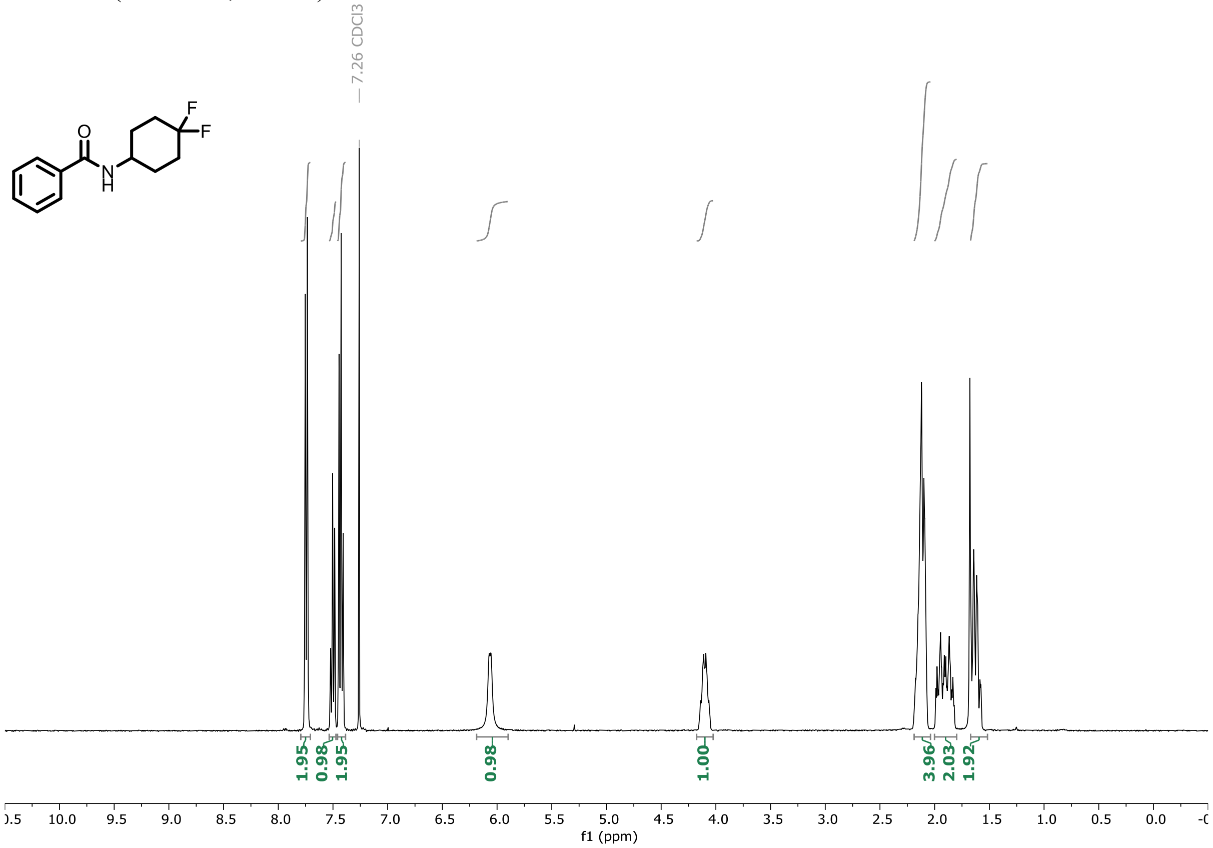

^{13}C NMR (101 MHz, $CDCl_3$) of **1d**

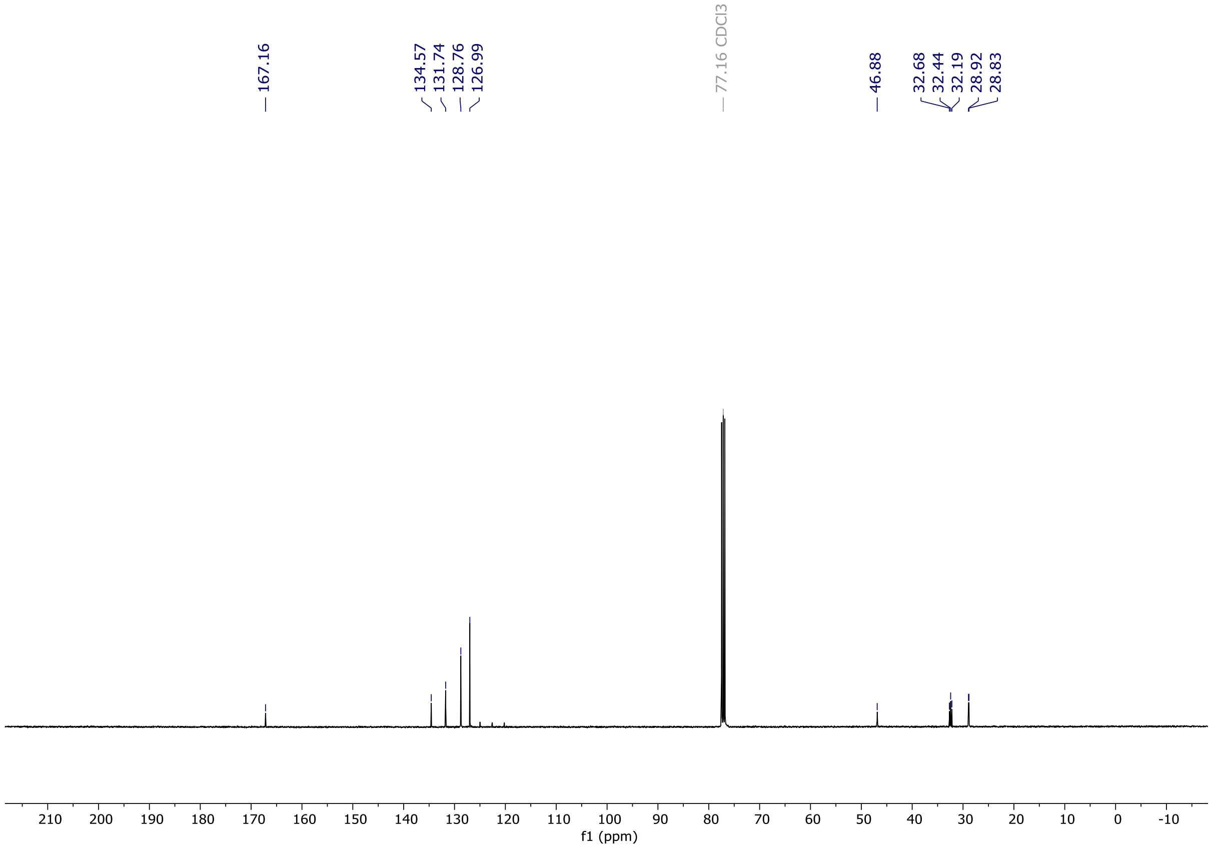

^{19}F NMR (282 MHz, $CDCl_3$) of **1d**

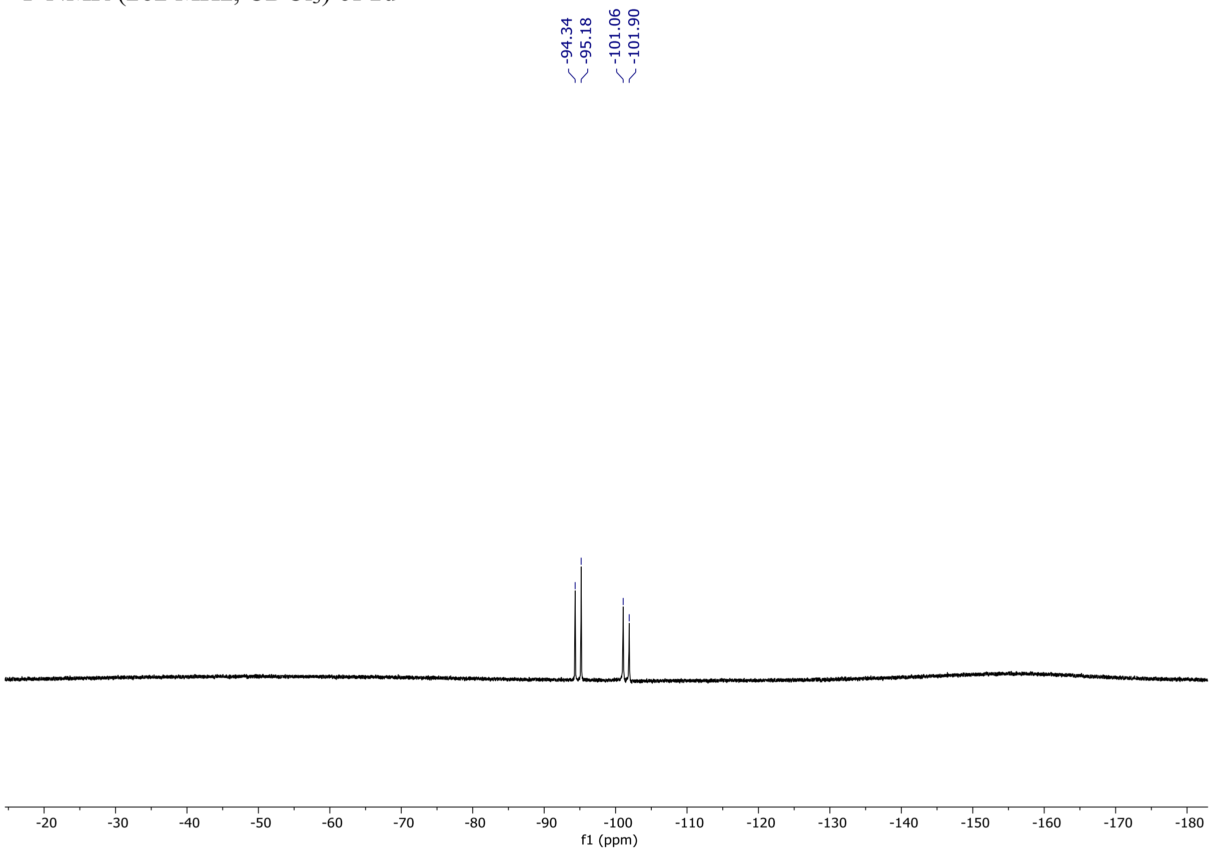

^{1}H NMR (400 MHz, $CDCl_3$) of **1f**

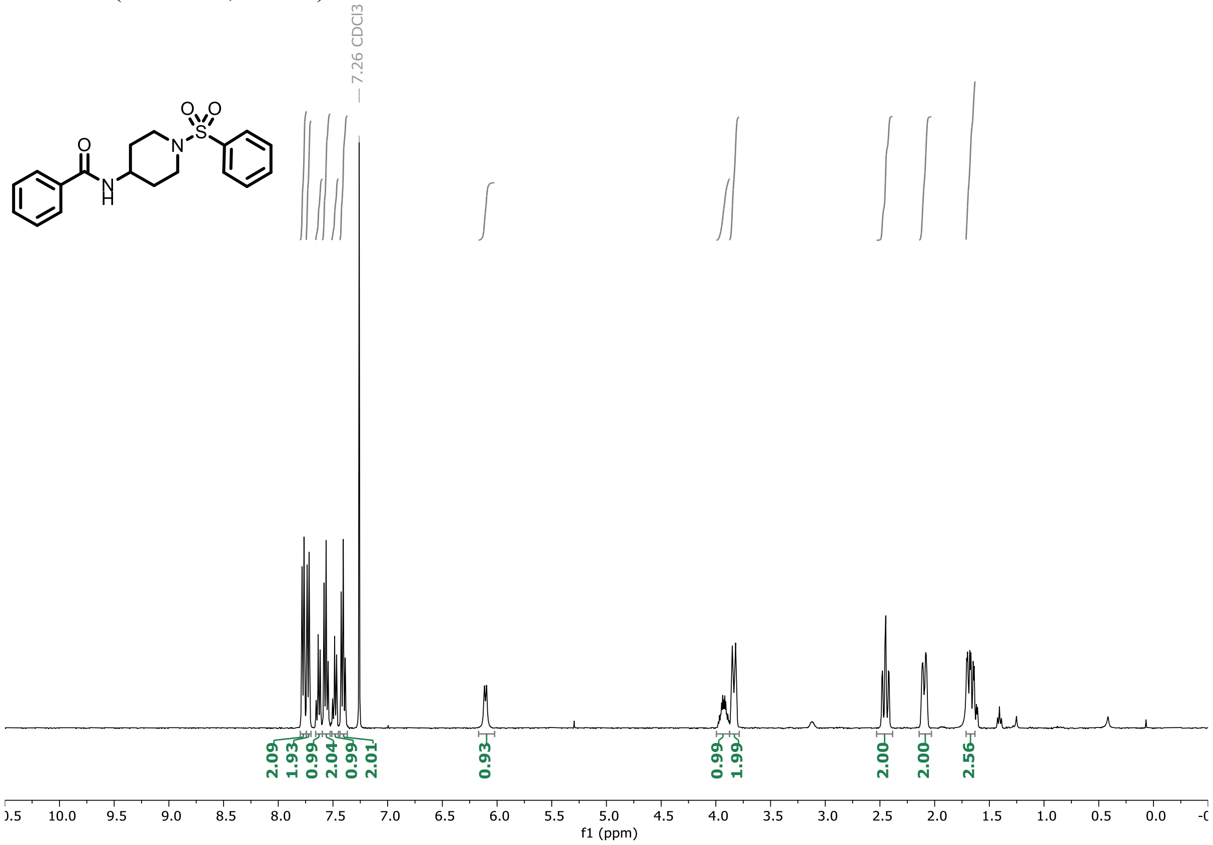

^{13}C NMR (101 MHz, $CDCl_3$) of **1f**

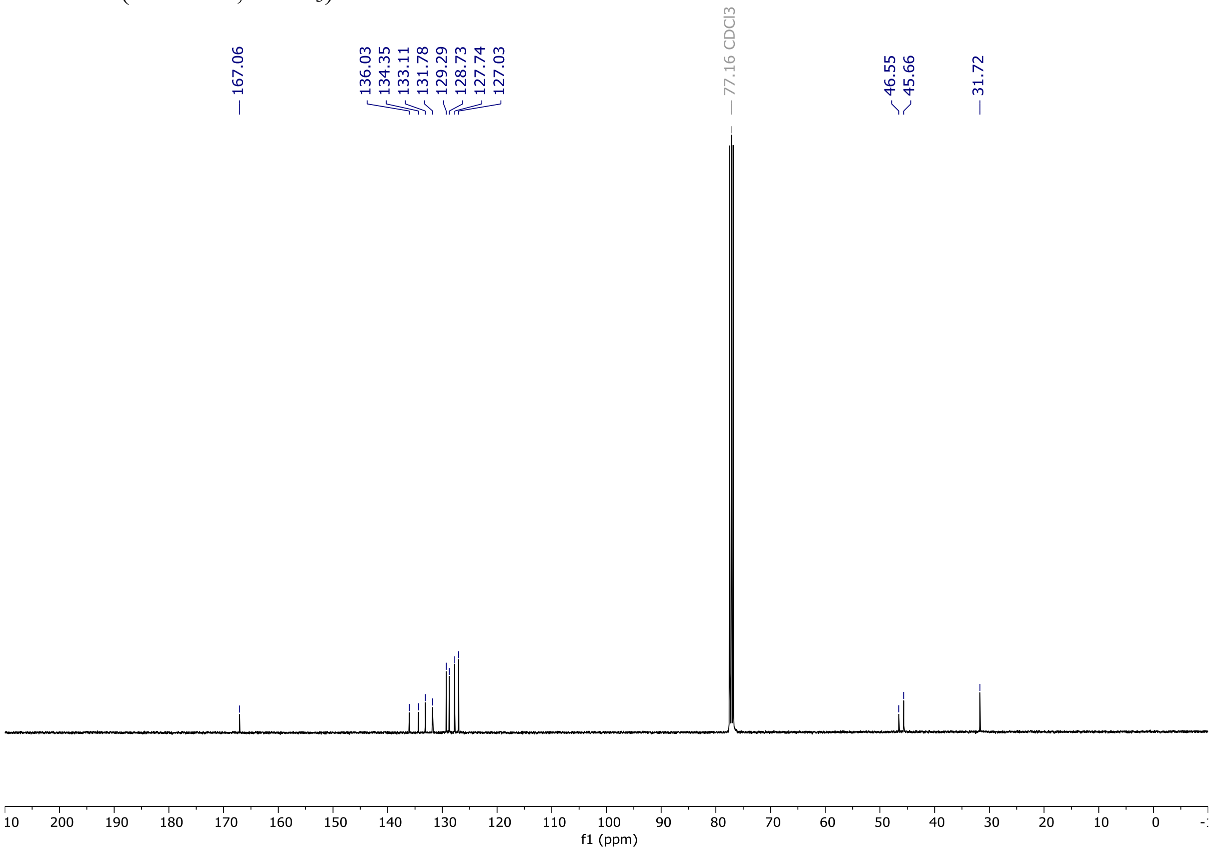

^{1}H NMR (400 MHz, $CDCl_3$) of **1g**

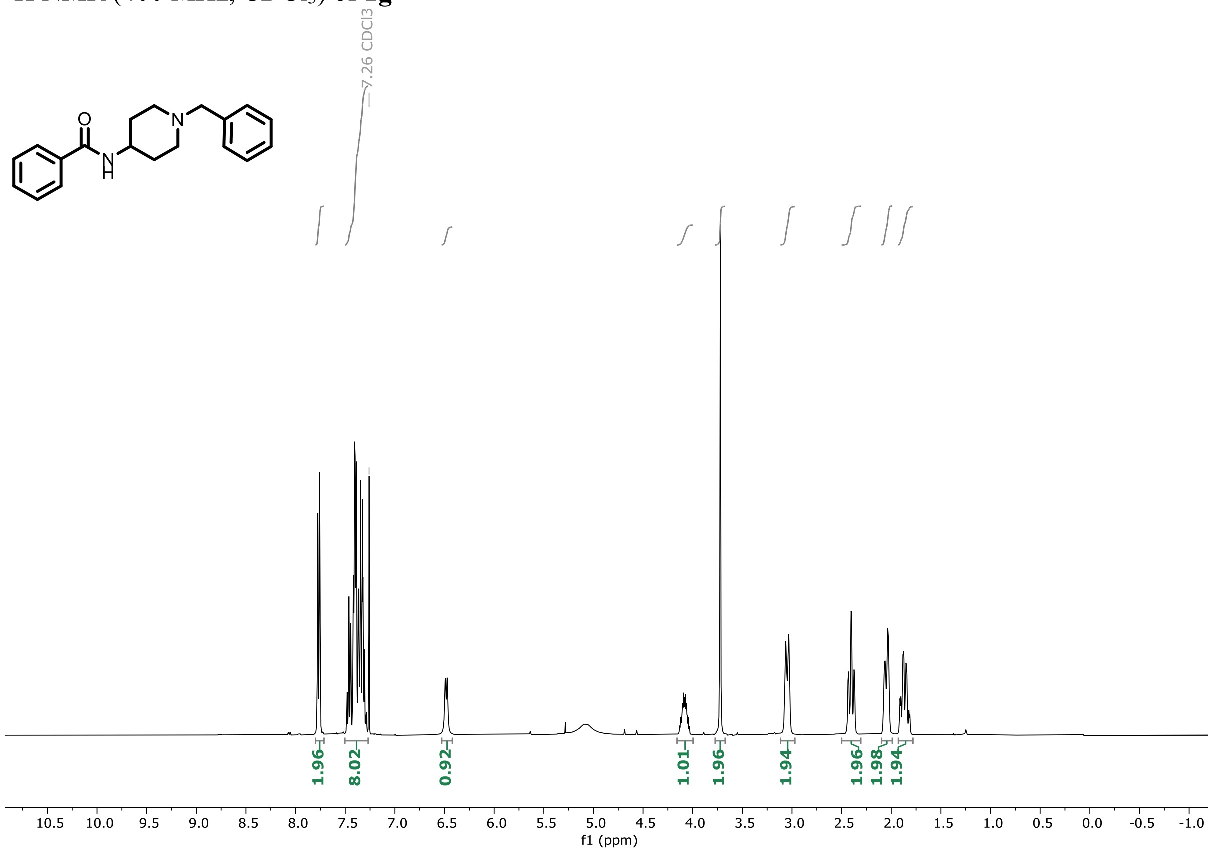

^{13}C NMR (101 MHz, $CDCl_3$) of **1g**

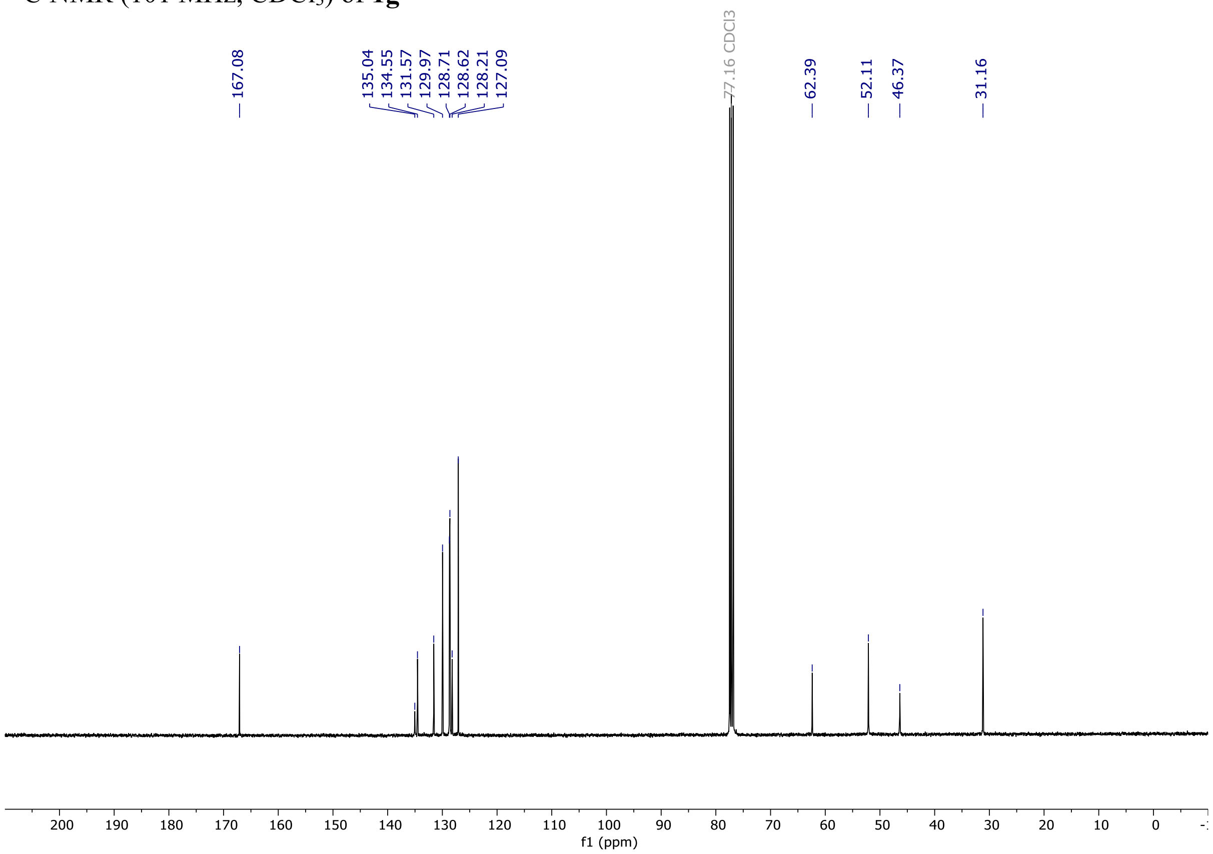

^{1}H NMR (400 MHz, $CDCl_3$) of **1o**

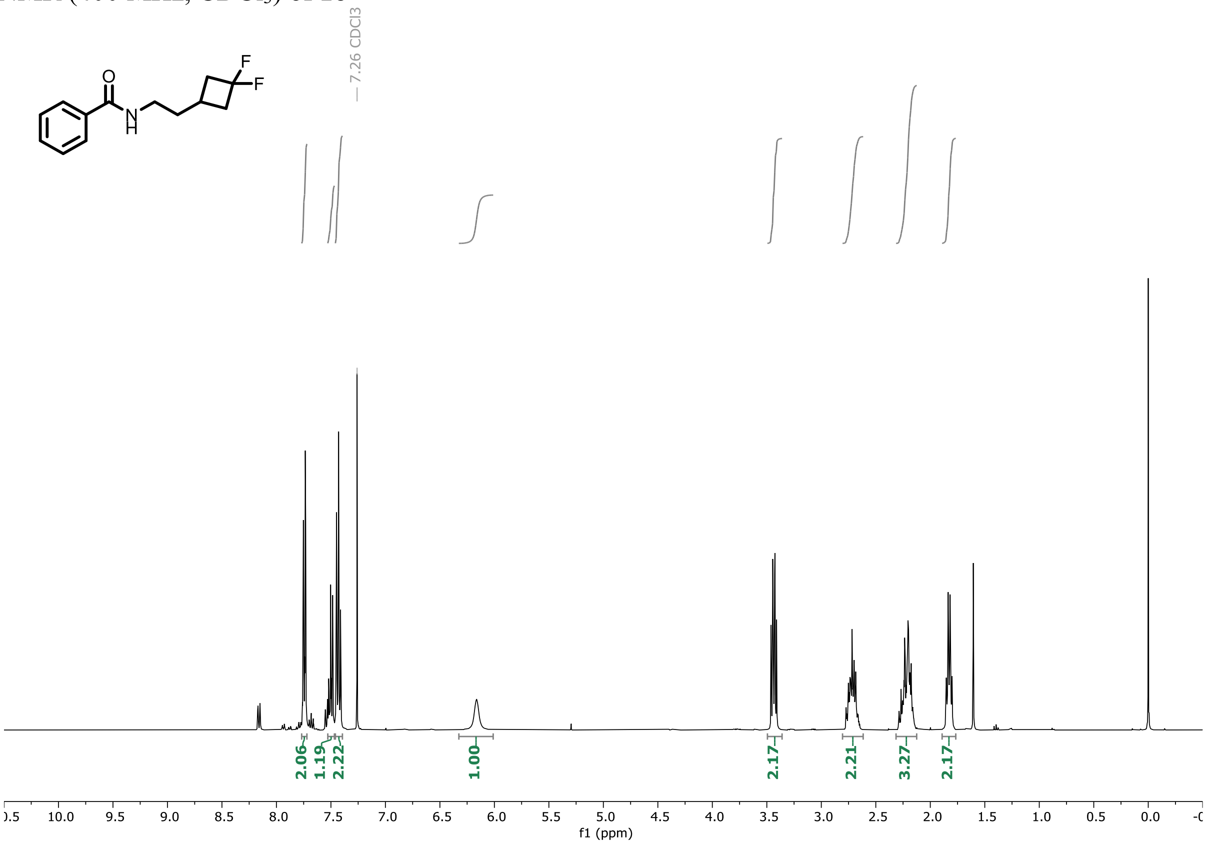

^{13}C NMR (101 MHz, $CDCl_3$) of **1o**

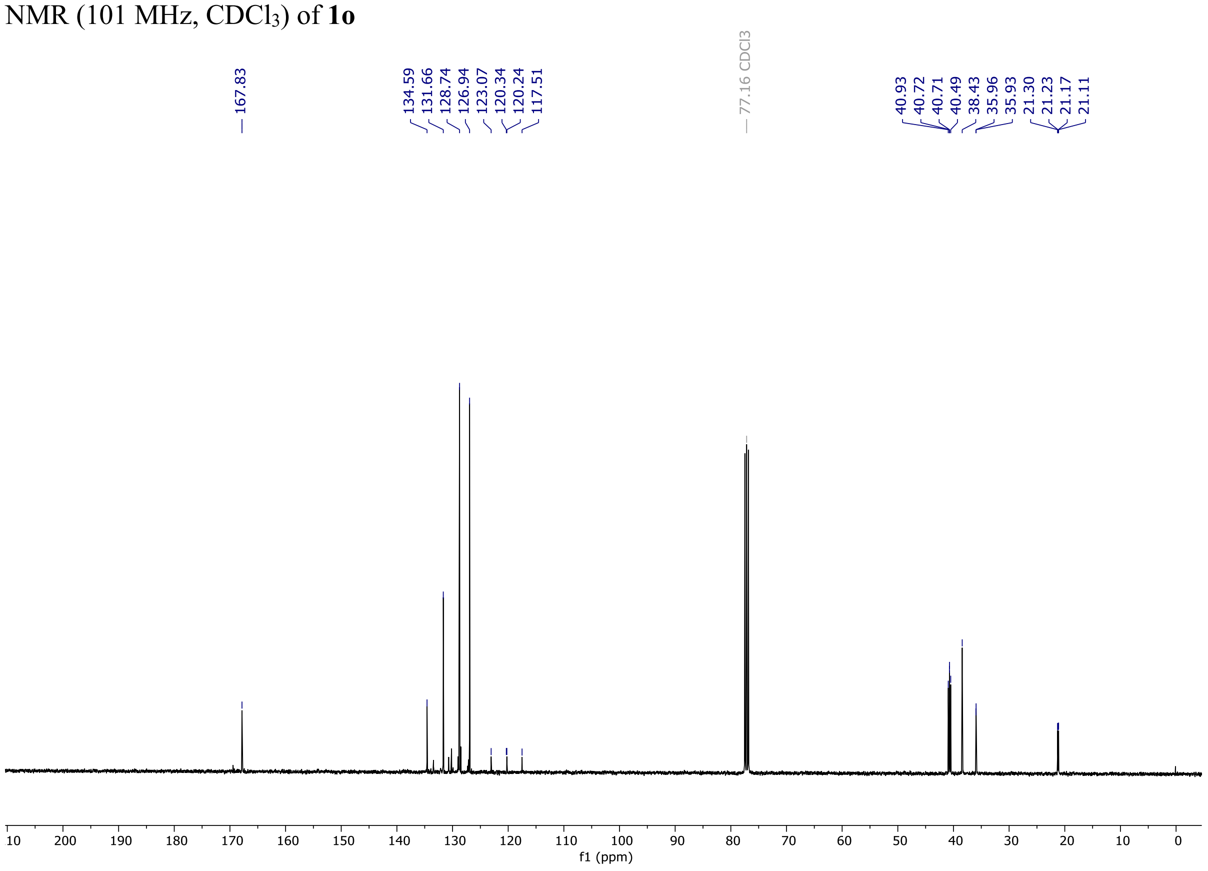

^{19}F NMR (377 MHz, $CDCl_3$)of **1o**

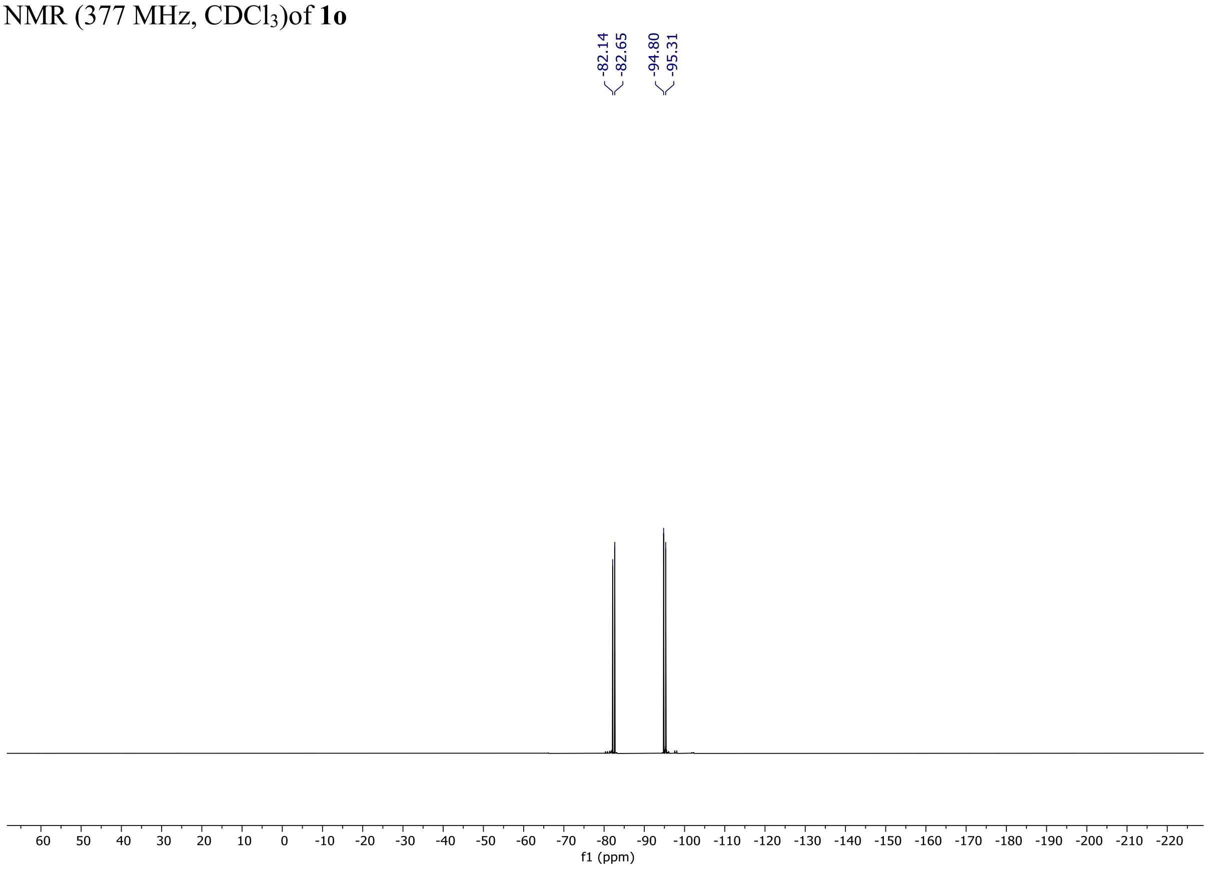

^1H NMR (400 MHz, $CDCl_3$) of **1p**

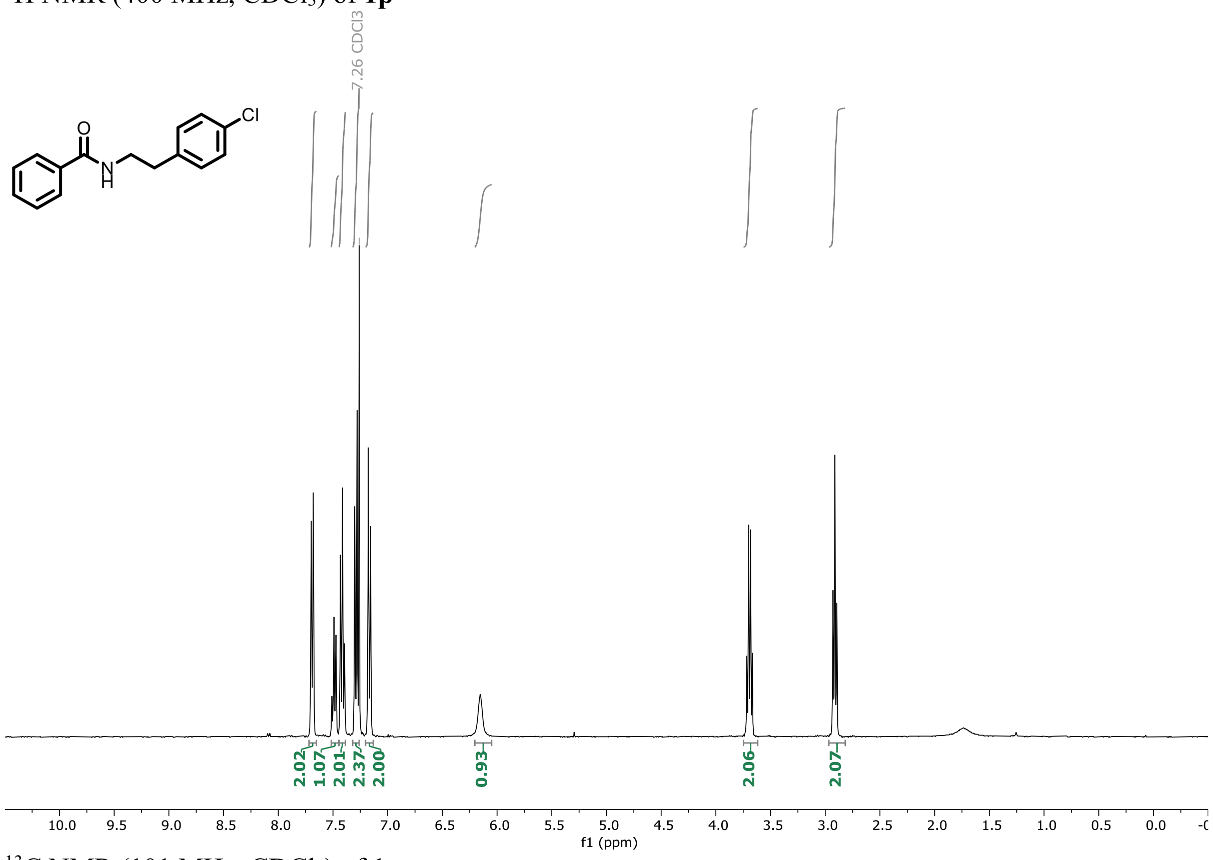

^{13}C NMR (101 MHz, $CDCl_3$) of **1p**

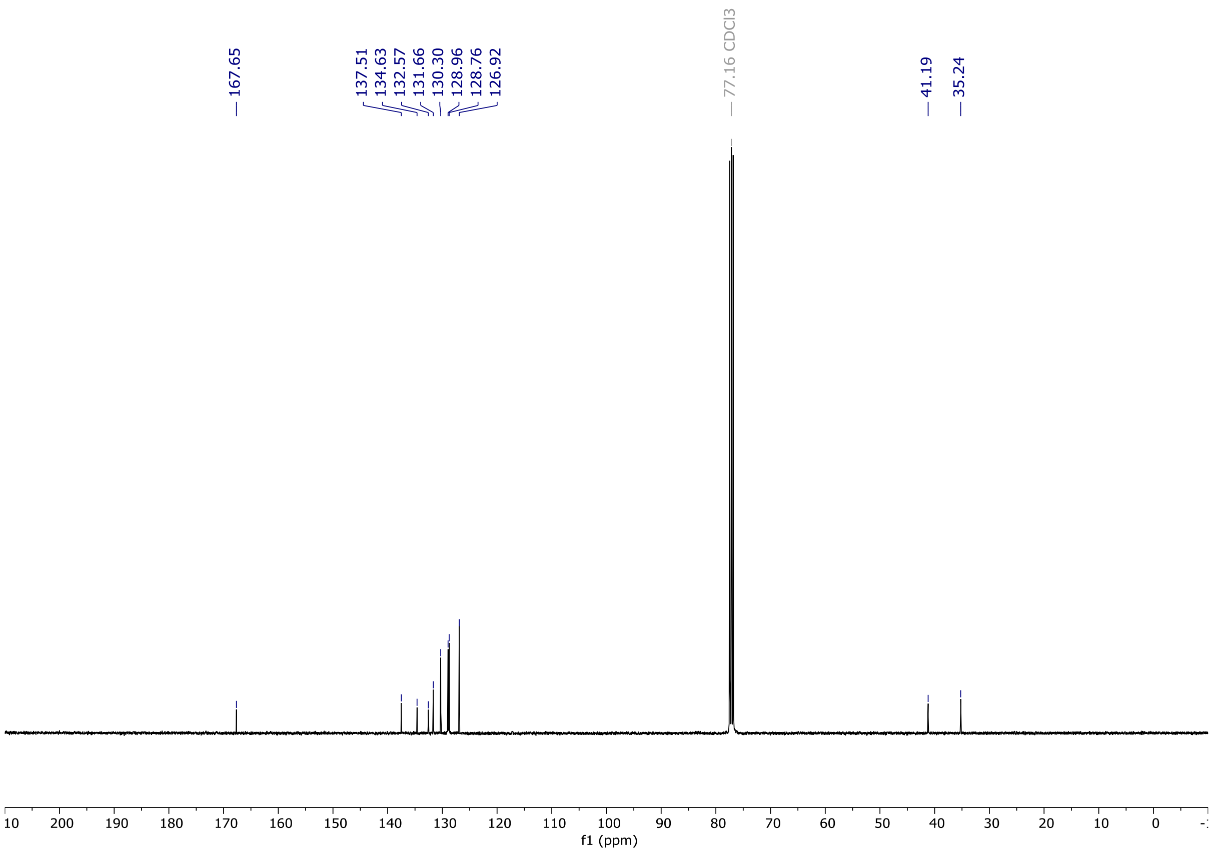

^1H NMR (400 MHz, $CDCl_3$) of **2c**

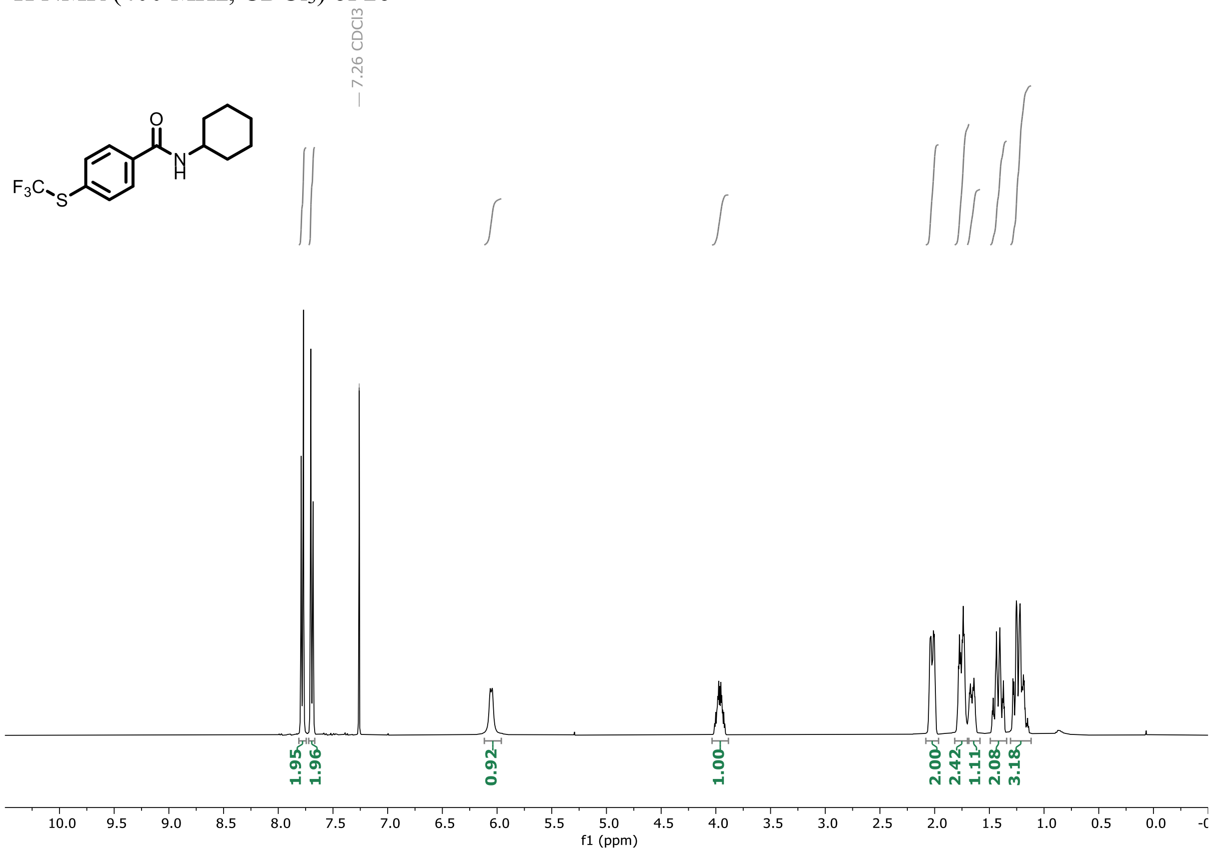

^{13}C NMR (101 MHz, $CDCl_3$) of **2c**

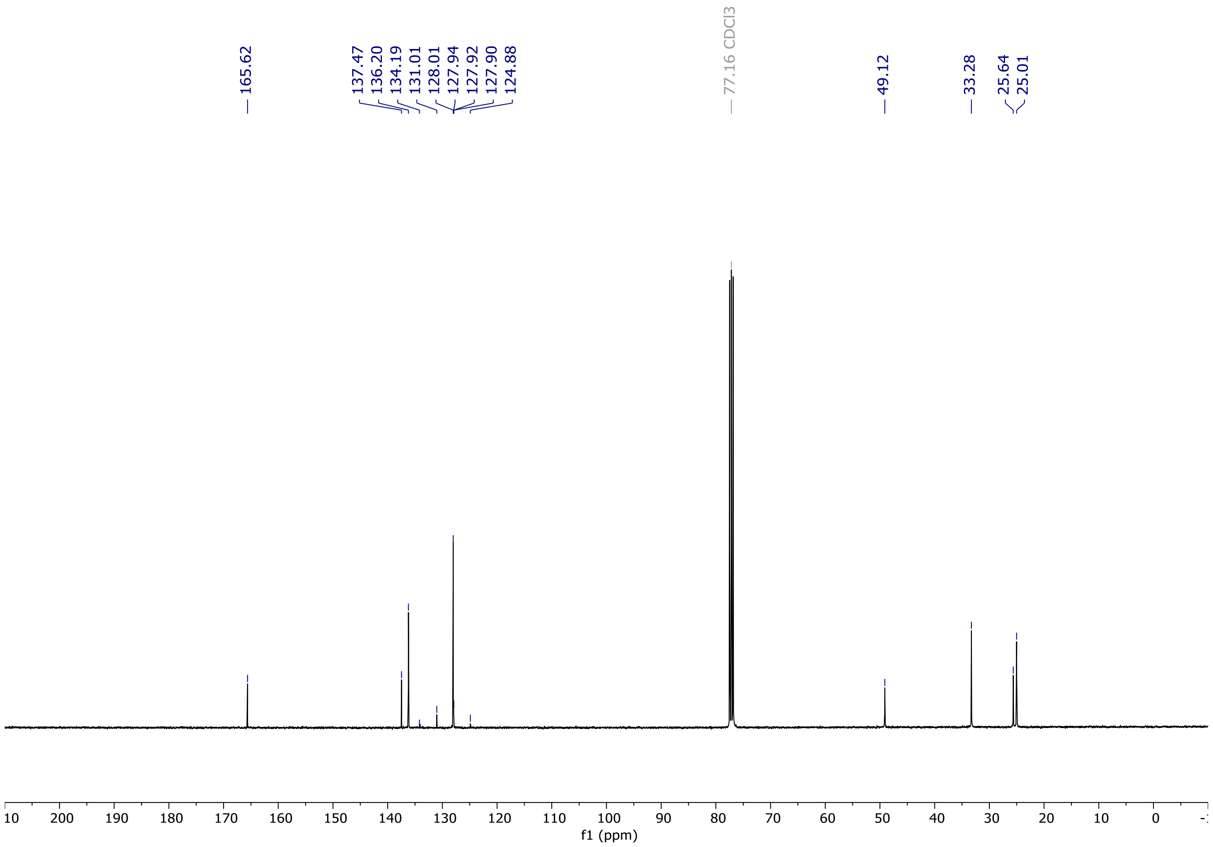

^{19}F NMR (282 MHz, $CDCl_3$) of **2c**

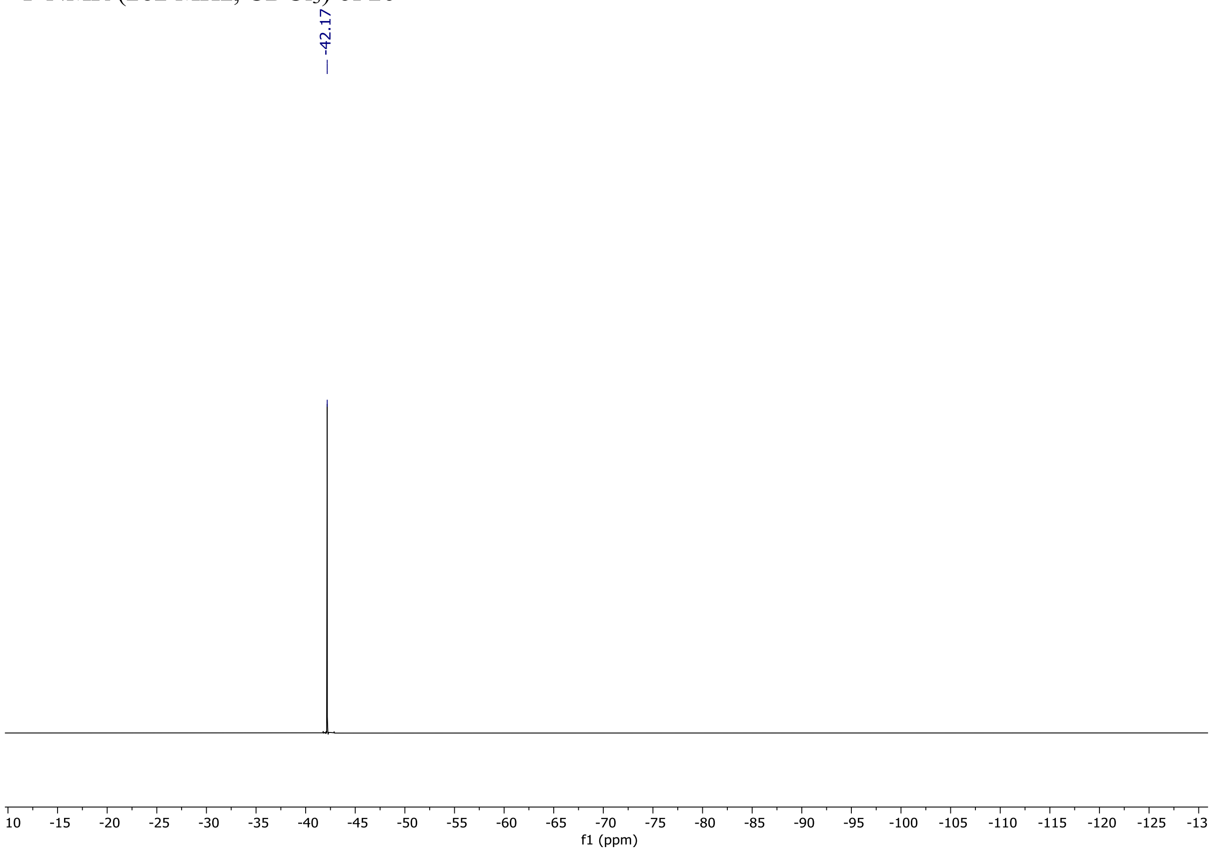

^{1}H NMR (400 MHz, $CDCl_3$) of **2h**

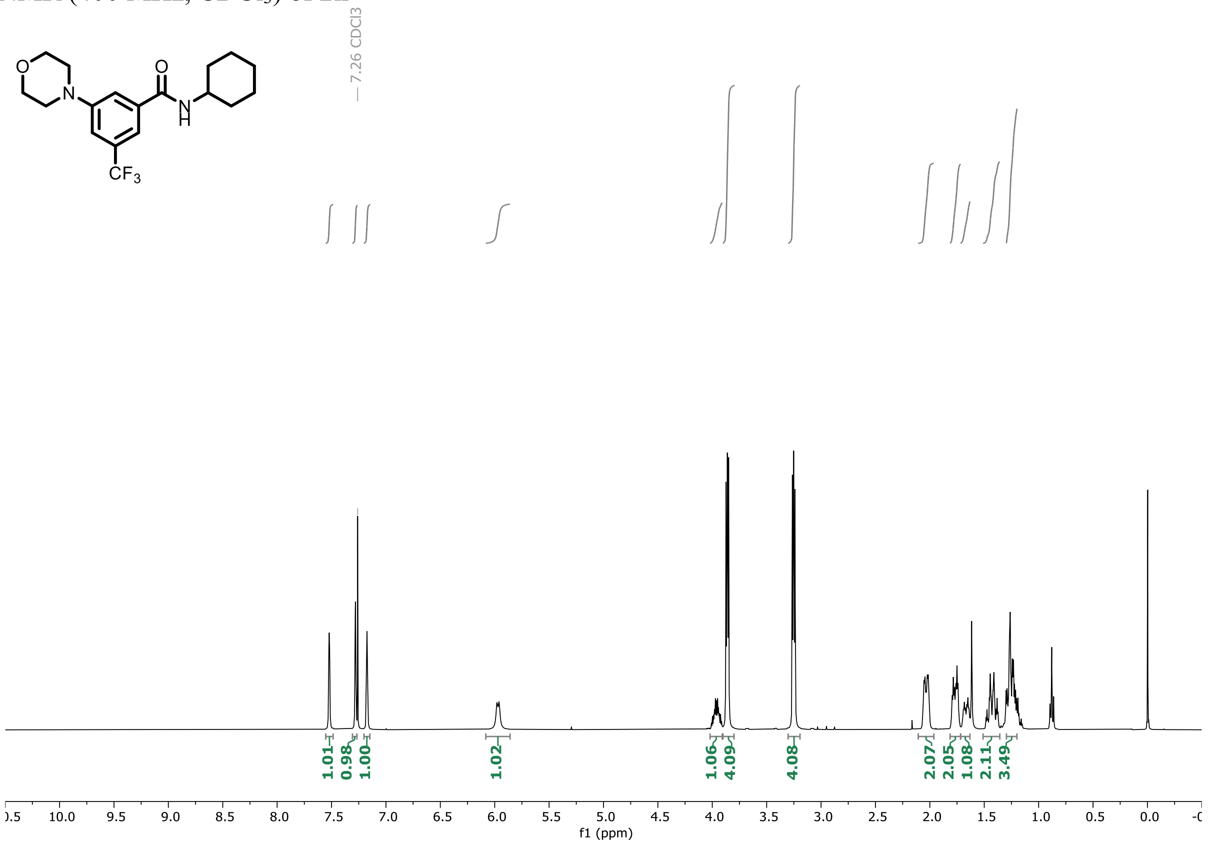

^{13}C NMR (101 MHz, $CDCl_3$) of **2h**

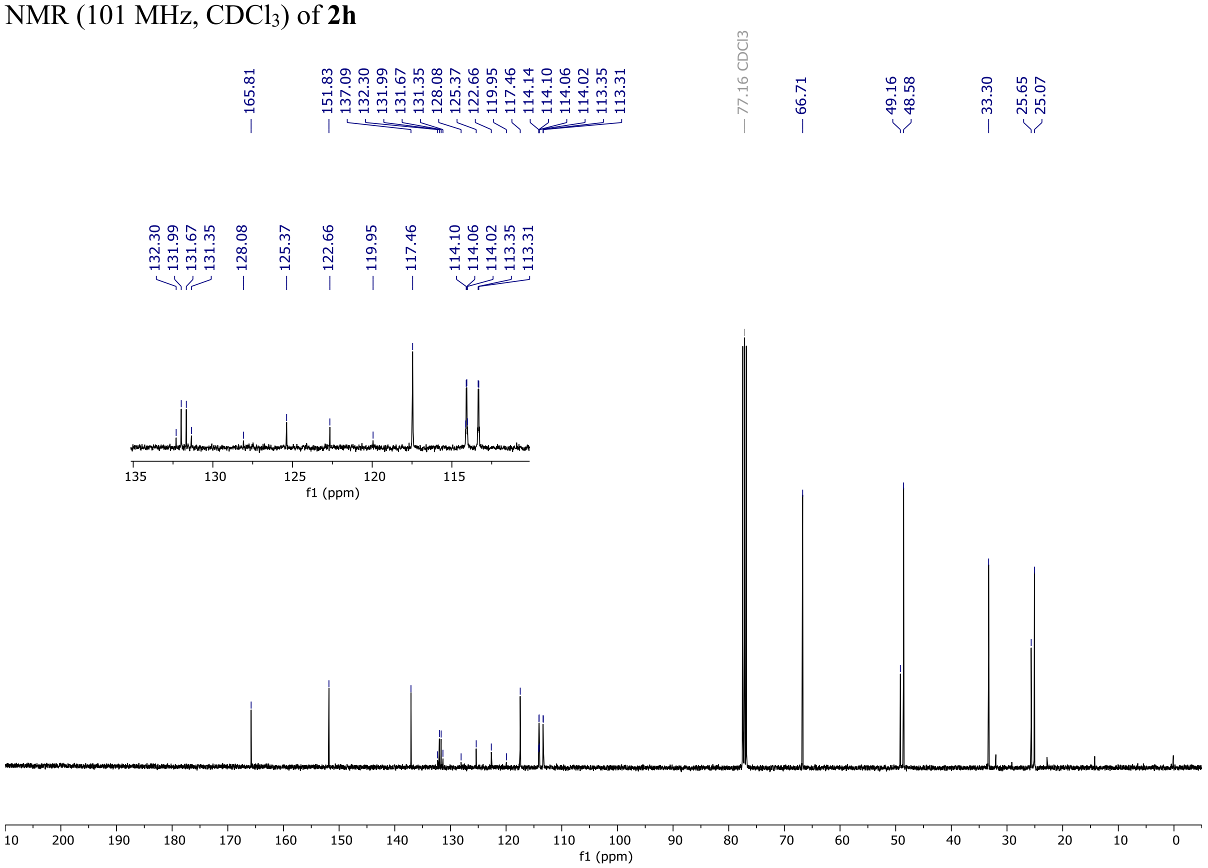

^{19}F NMR (377 MHz, $CDCl_3$)of **2h**

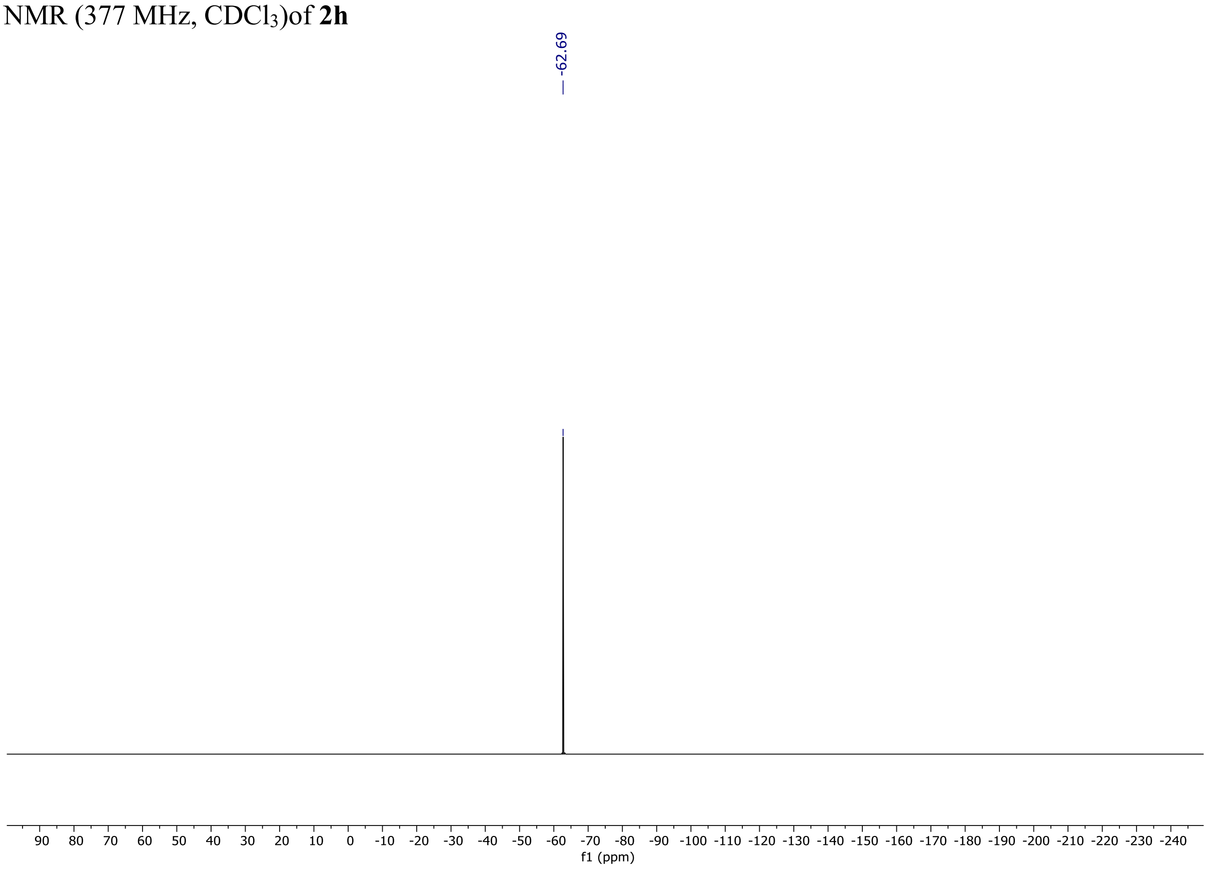

^{1}H NMR (400 MHz, $CDCl_3$) of **2i**

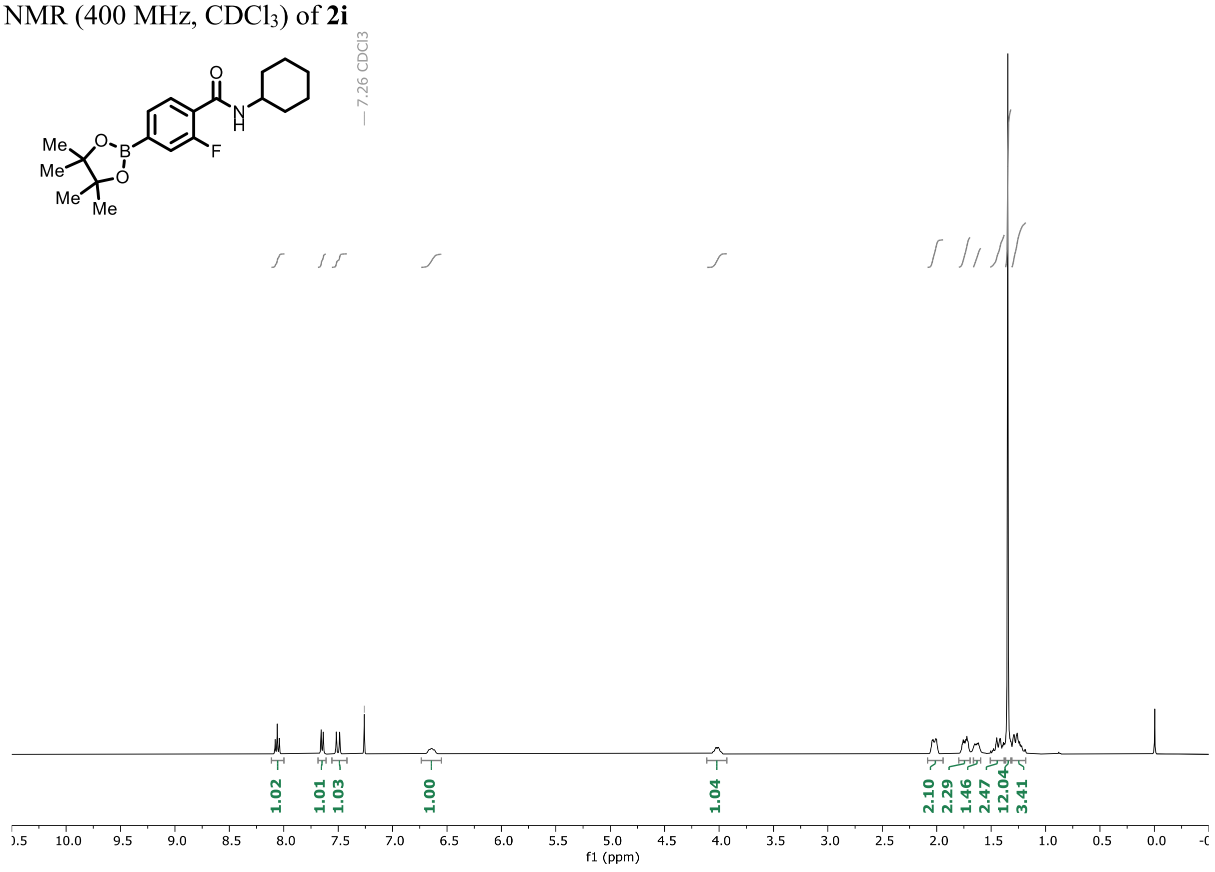

^{13}C NMR (101 MHz, $CDCl_3$) of **2i**

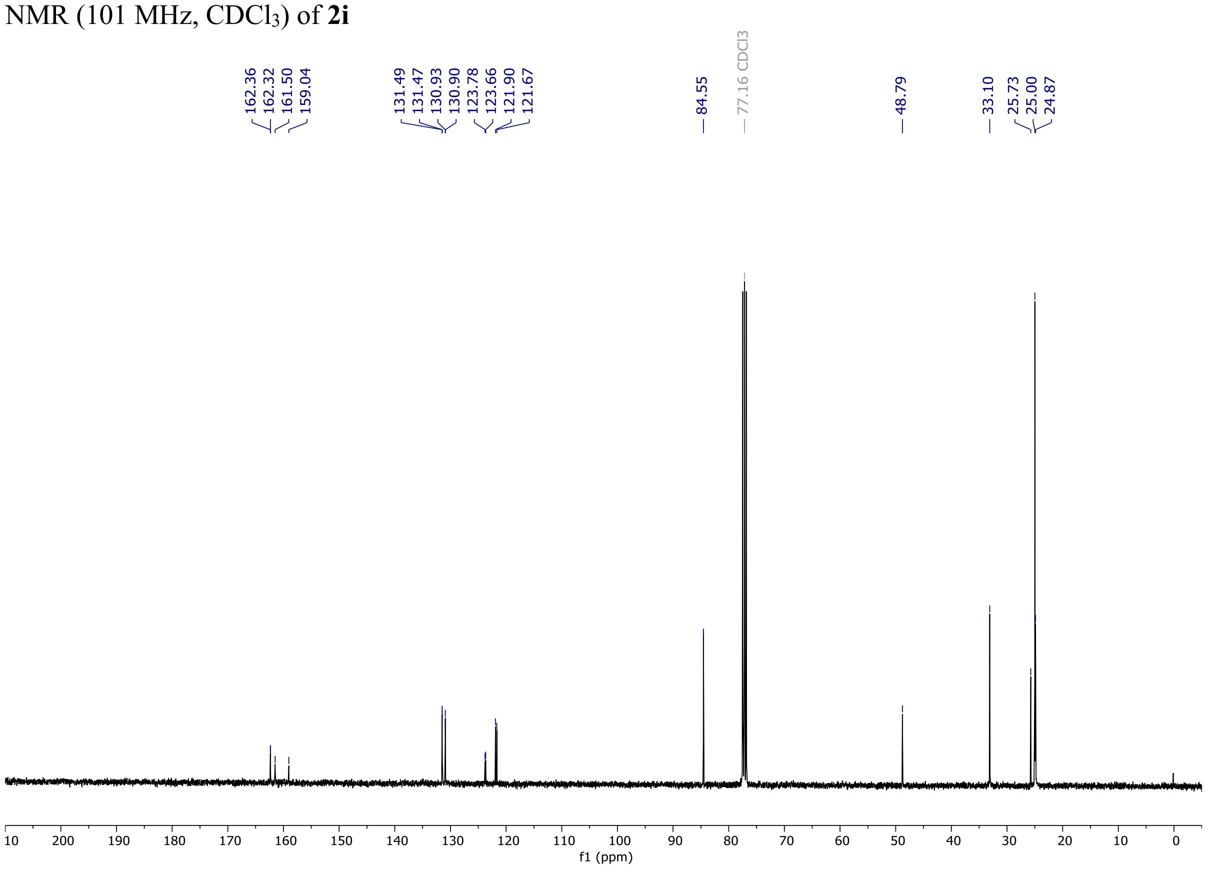

^{11}B NMR (128 MHz, $CDCl_3$) of **2i**

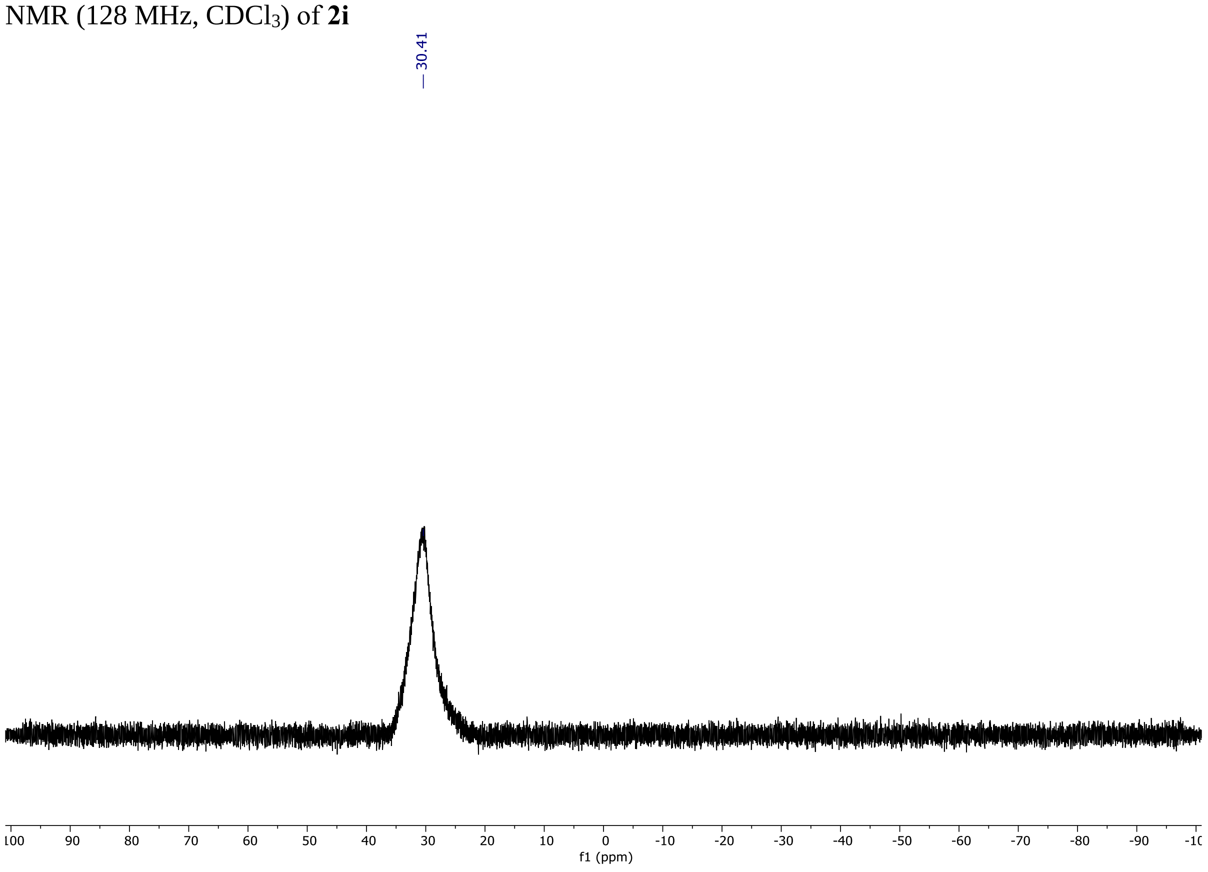

^{1}H NMR (400 MHz, $CDCl_3$) of **2j**

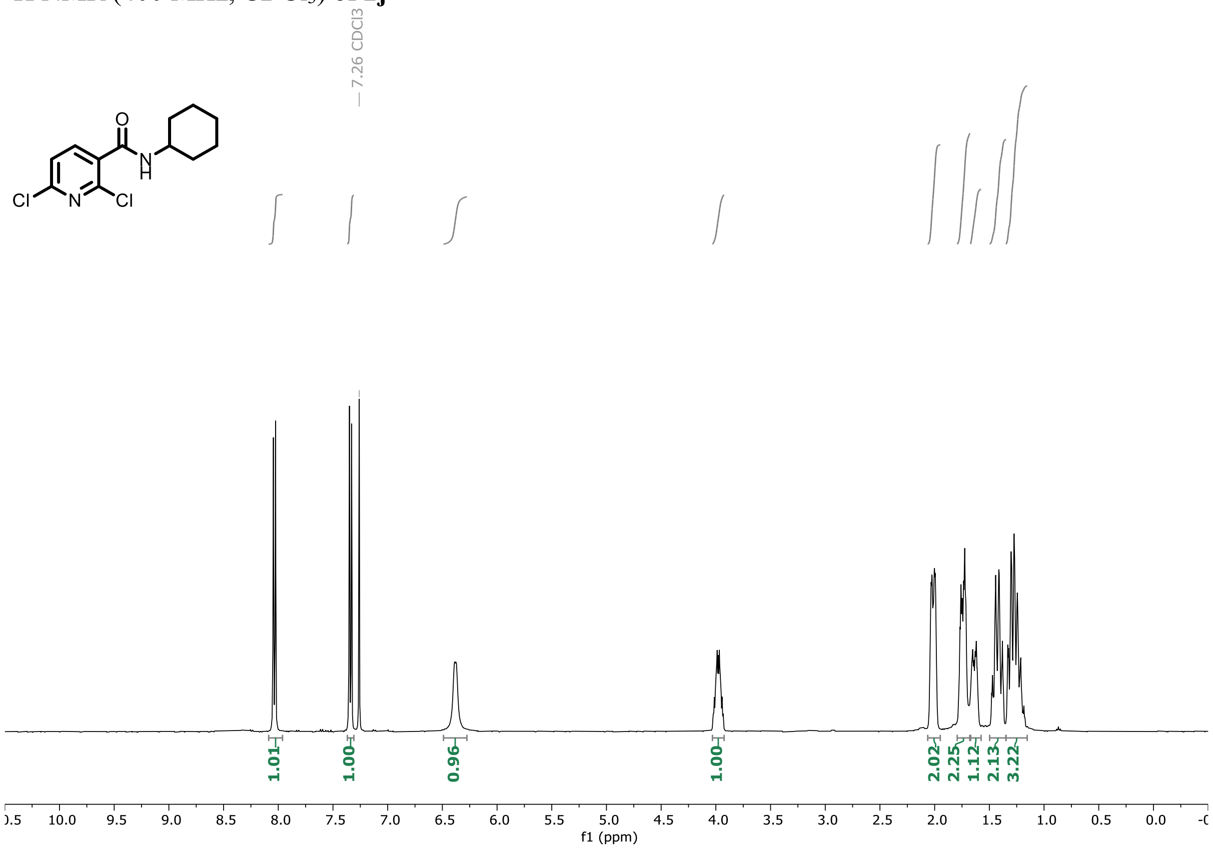

^{13}C NMR (101 MHz, $CDCl_3$) of **2j**

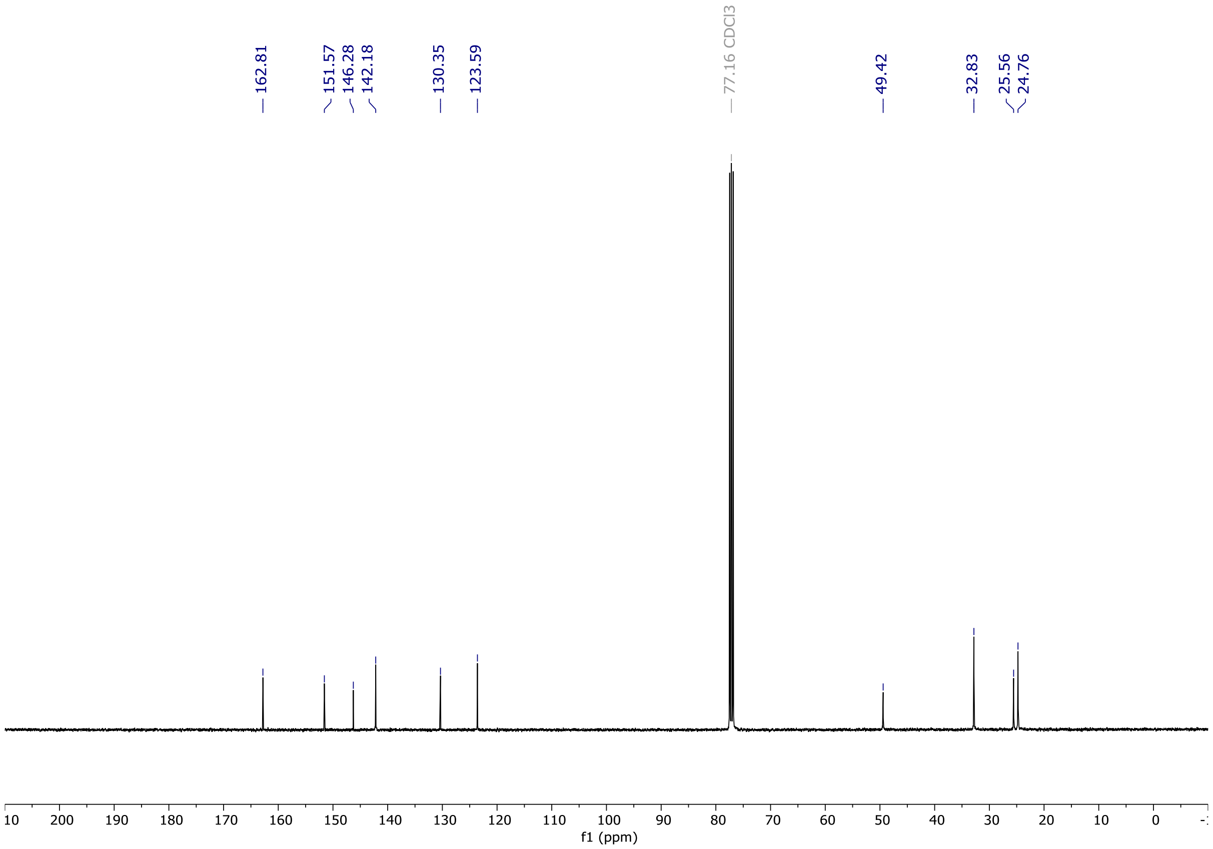

^{1}H NMR (400 MHz, $CDCl_3$) of **2k**

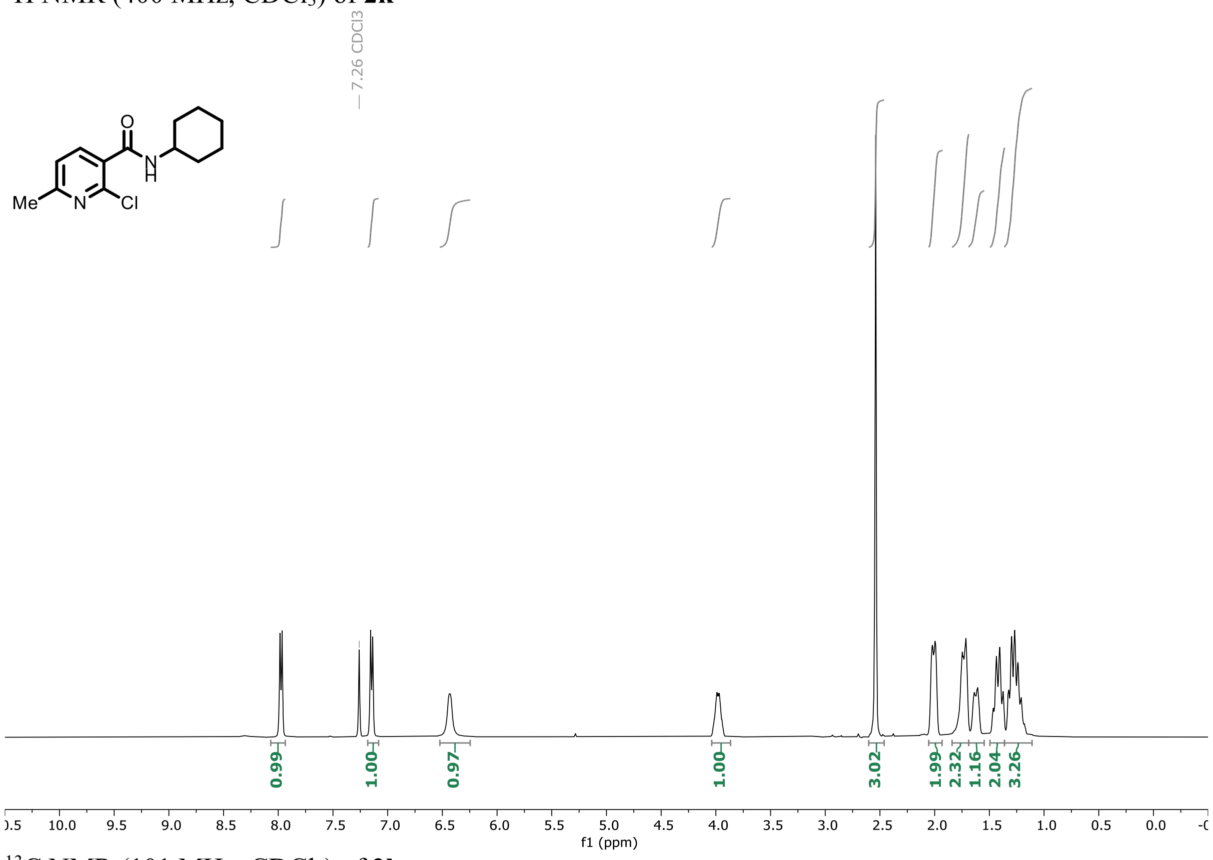

^{13}C NMR (101 MHz, $CDCl_3$) of **2k**

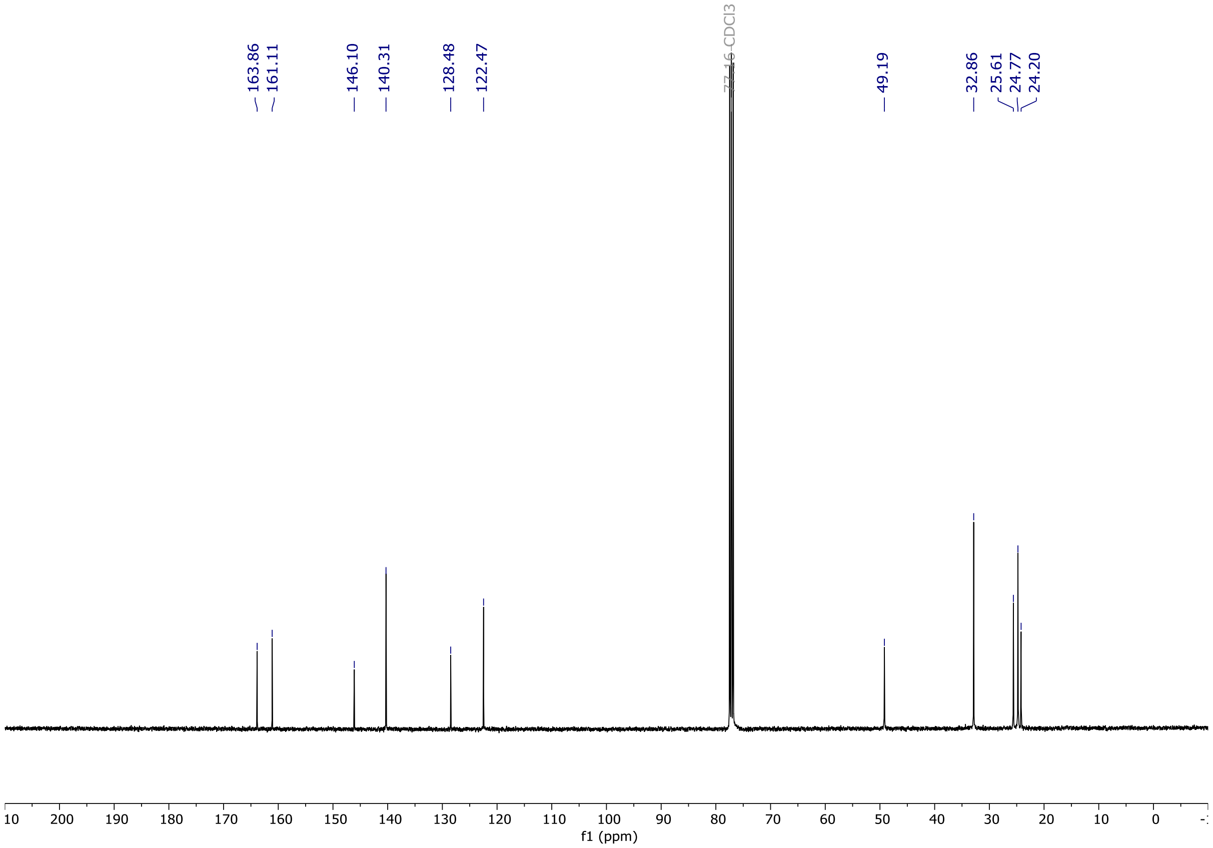

^1H NMR (400 MHz, $CDCl_3$) of **2l**

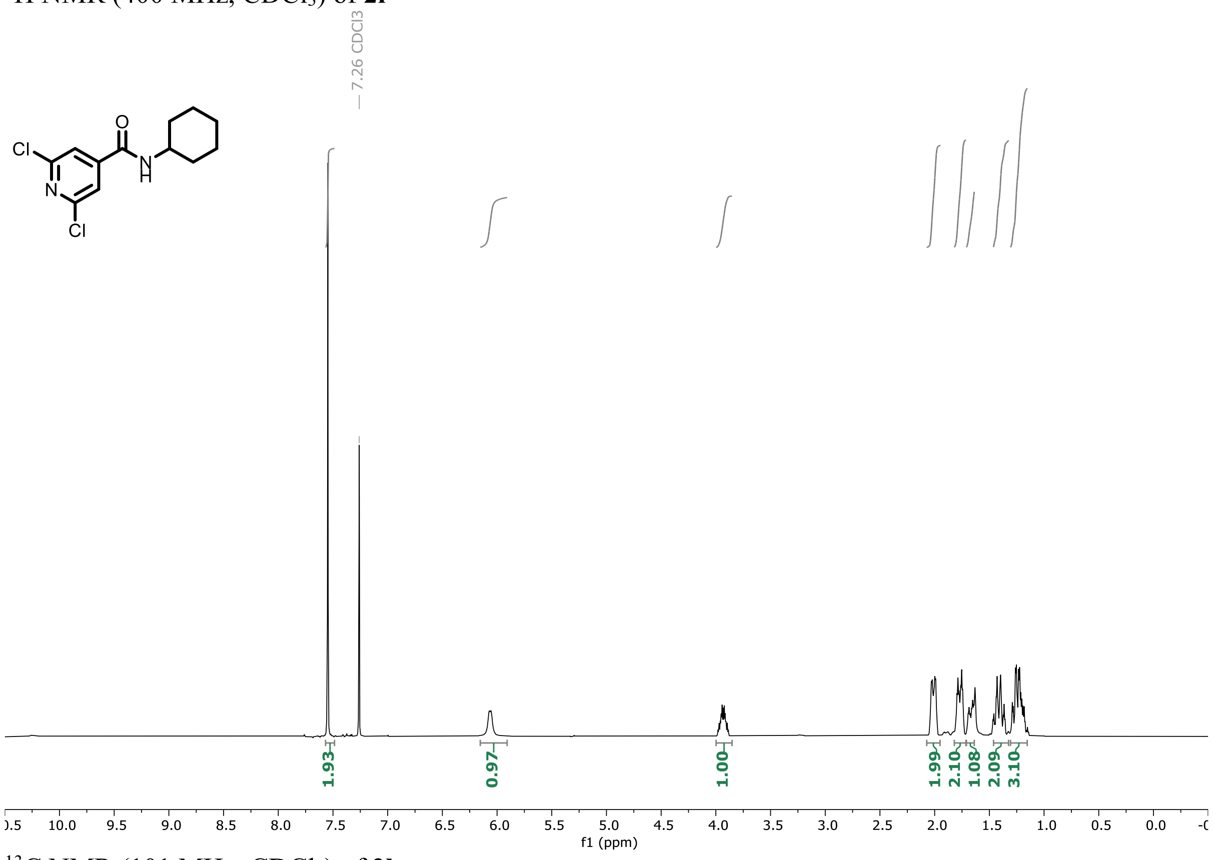

^{13}C NMR (101 MHz, $CDCl_3$) of **2l**

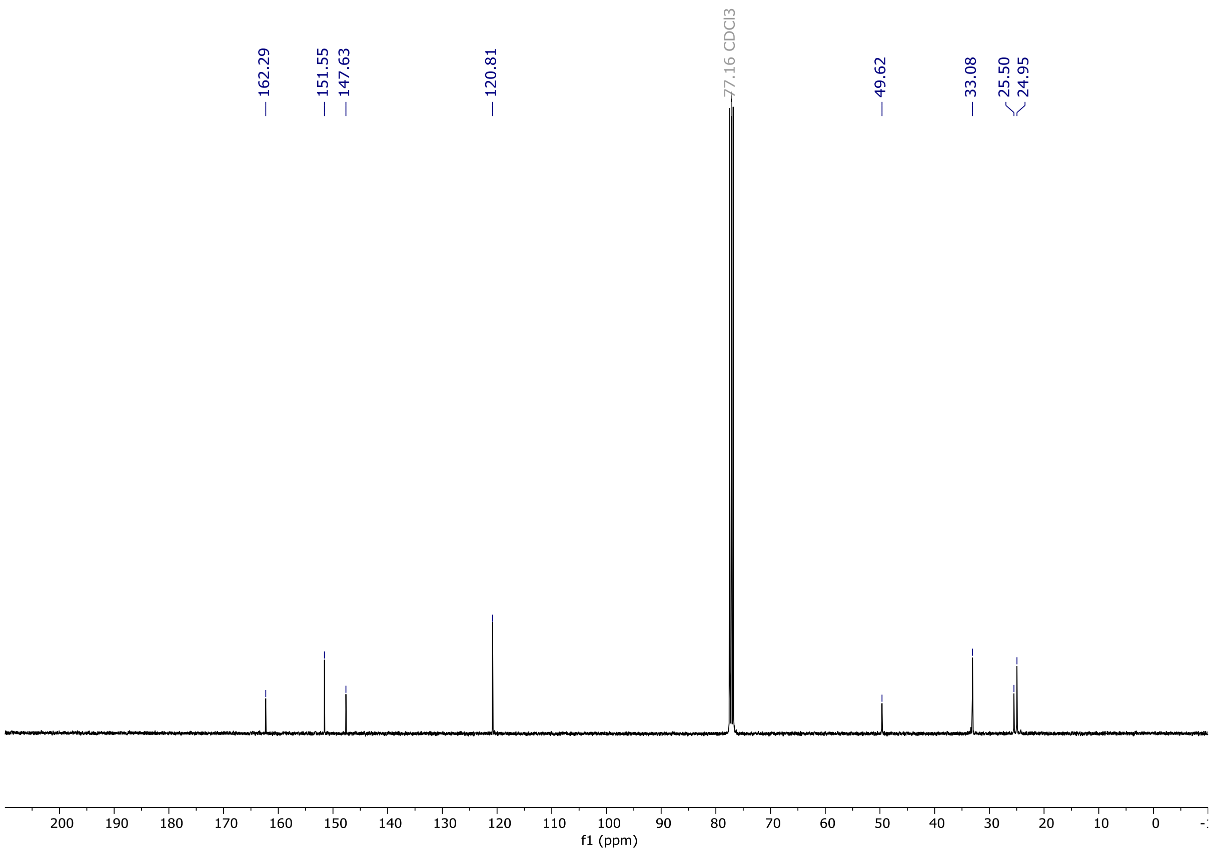

^{1}H NMR (400 MHz, $CDCl_3$) of **2n**

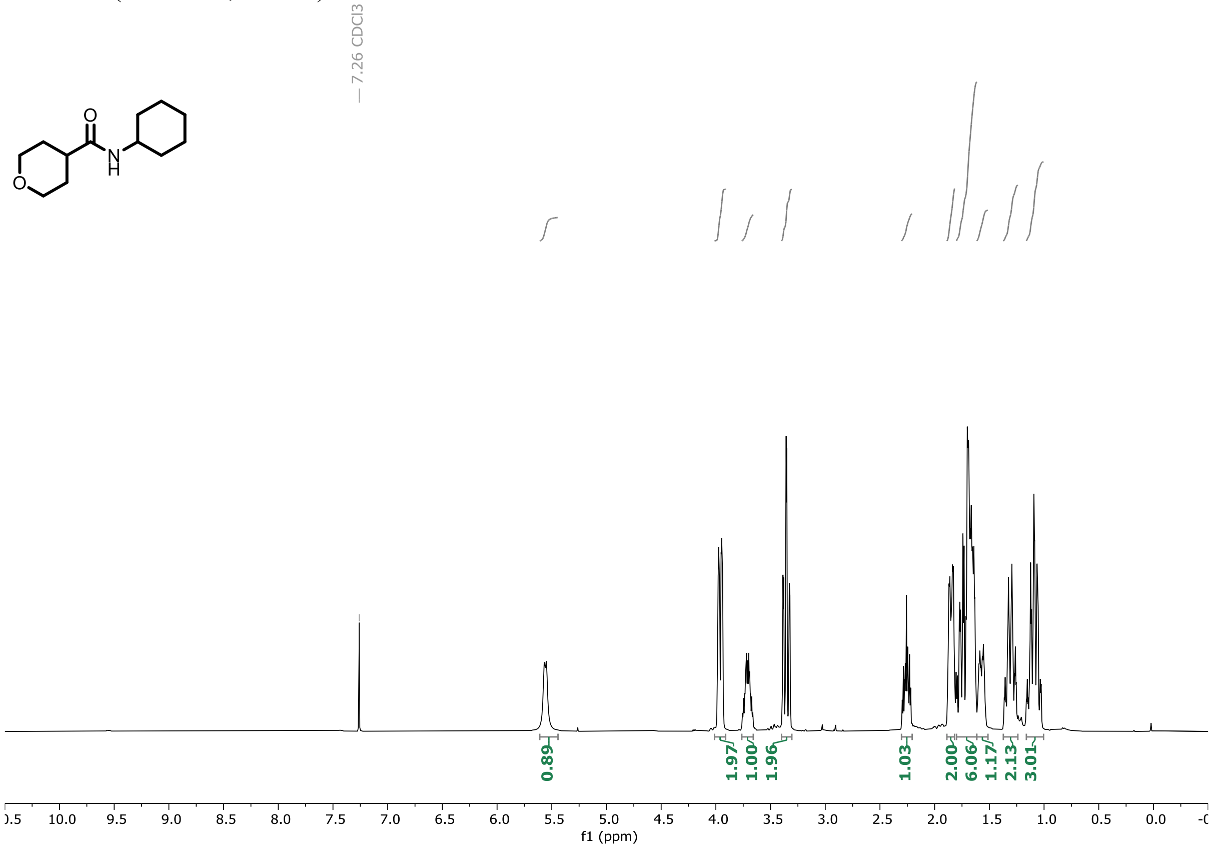

^{13}C NMR (101 MHz, $CDCl_3$) of **2n**

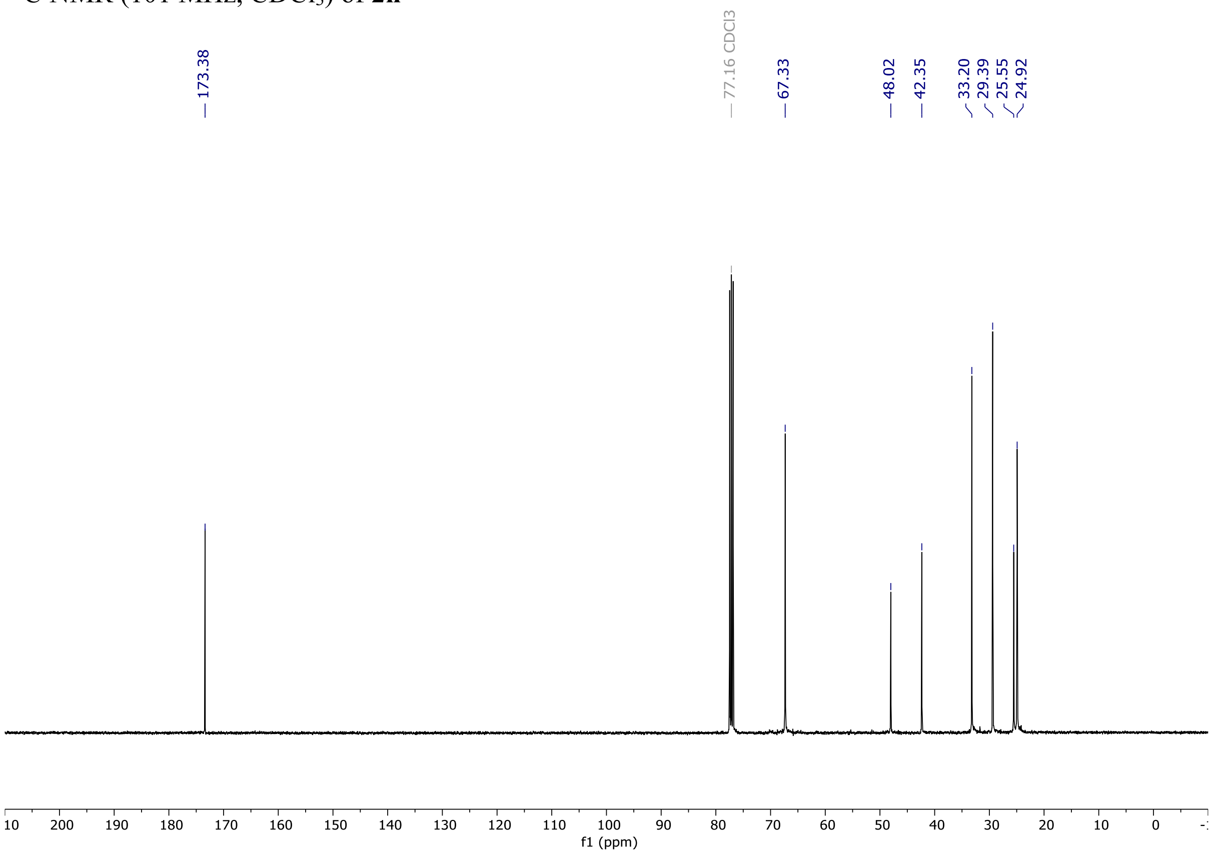

^{1}H NMR (400 MHz, $CDCl_3$) of **2p**

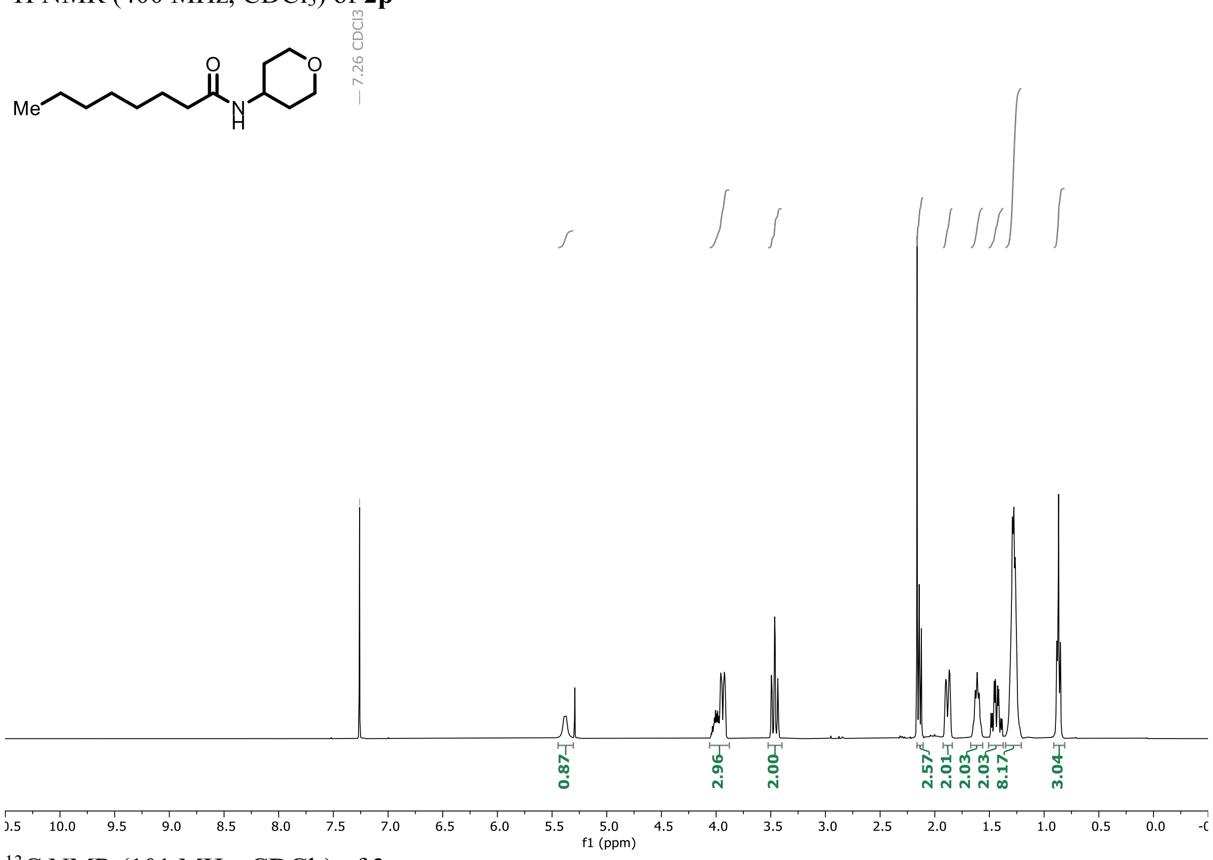

^{13}C NMR (101 MHz, $CDCl_3$) of **2p**

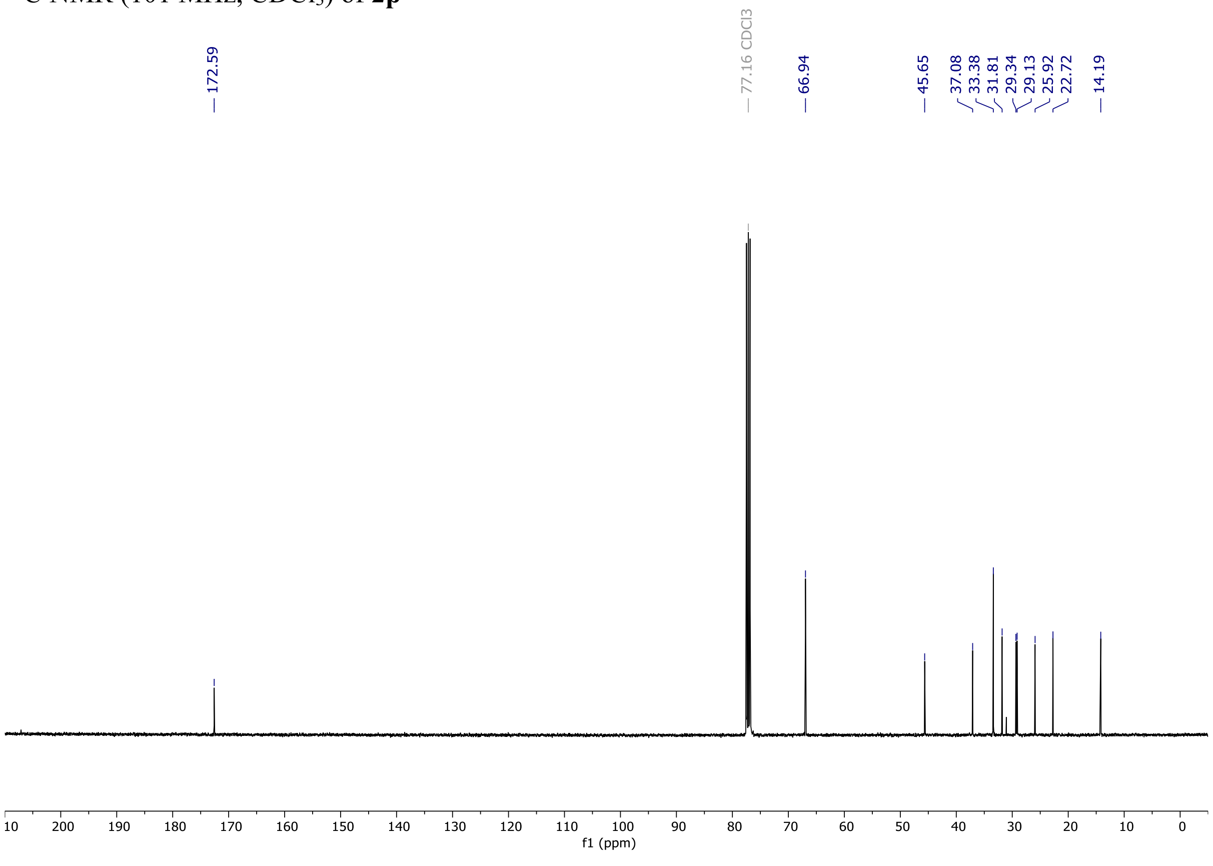

^{1}H NMR (400 MHz, $CDCl_3$) of **2q**

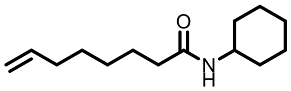

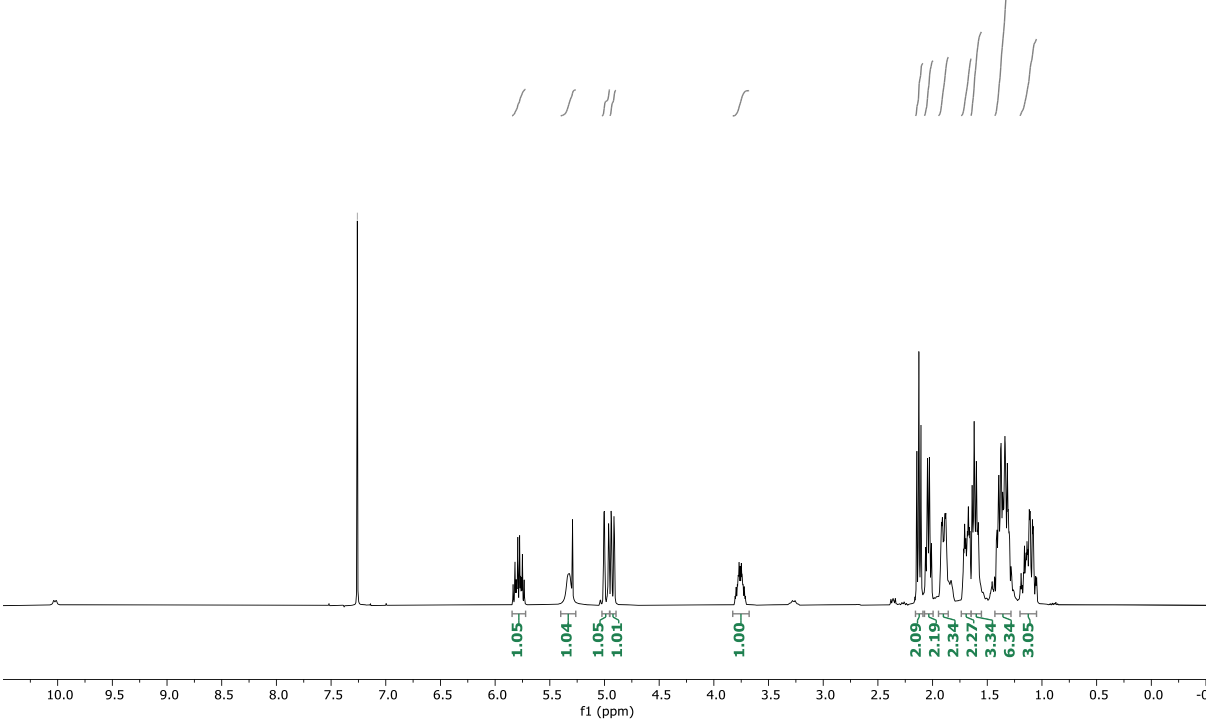

^{13}C NMR (101 MHz, $CDCl_3$) of **2q**

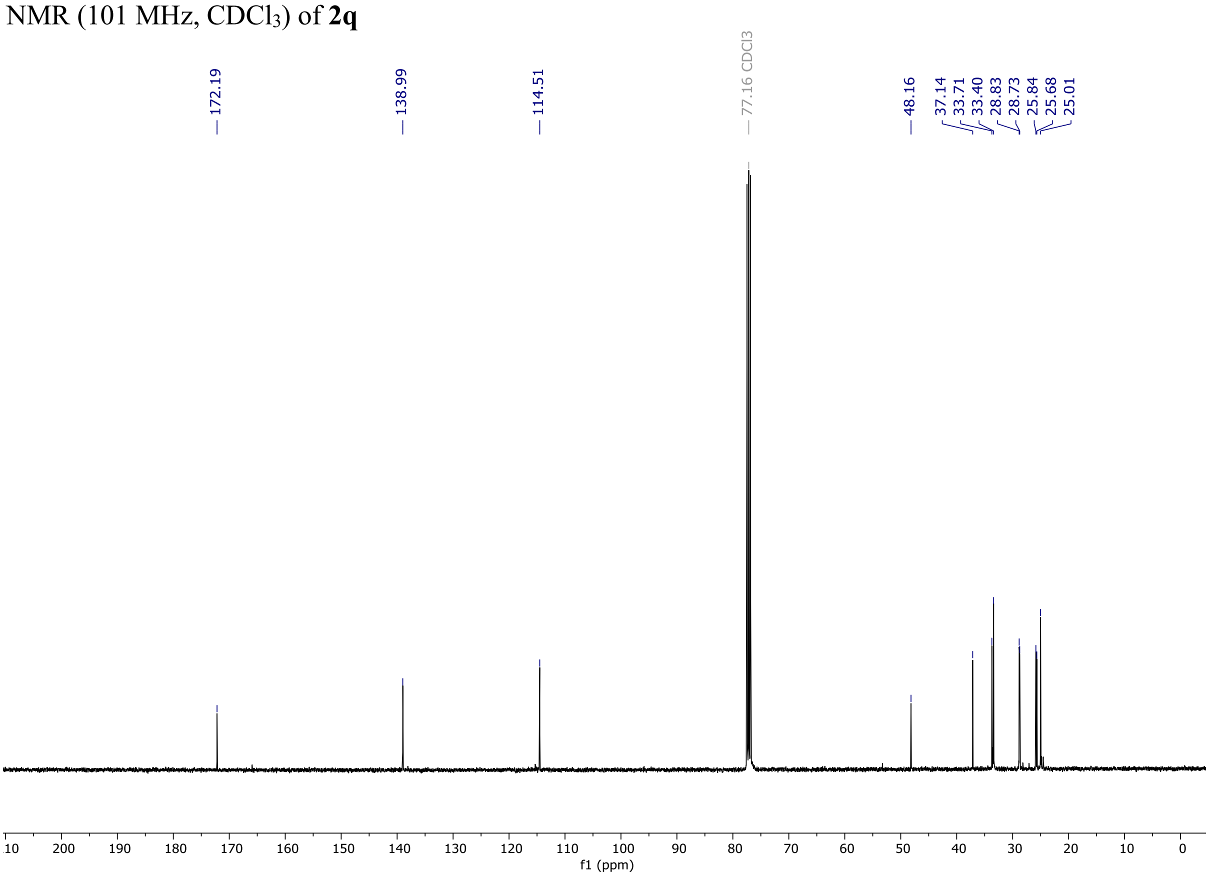

^1H NMR (400 MHz, $CDCl_3$) of **2r**

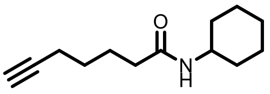

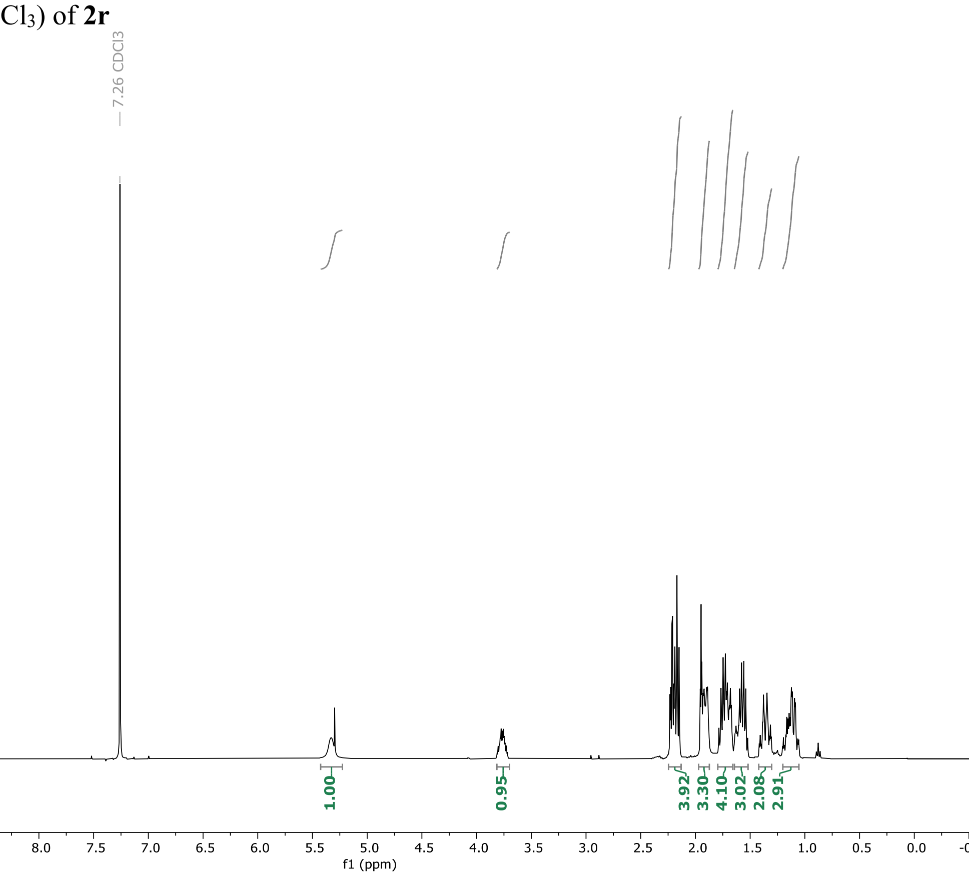

^{13}C NMR (101 MHz, $CDCl_3$) of **2r**

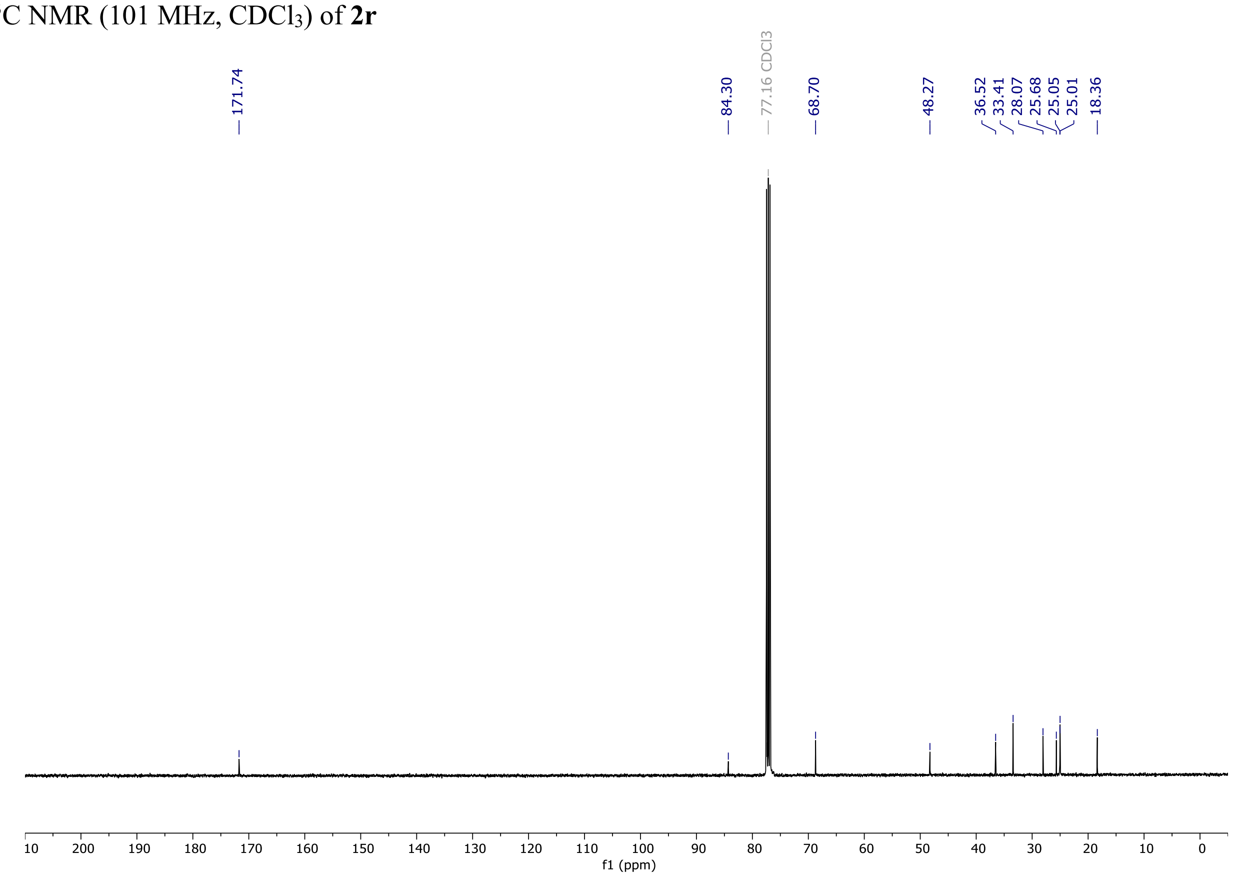

^{1}H NMR (400 MHz, $CDCl_3$) of **2t**

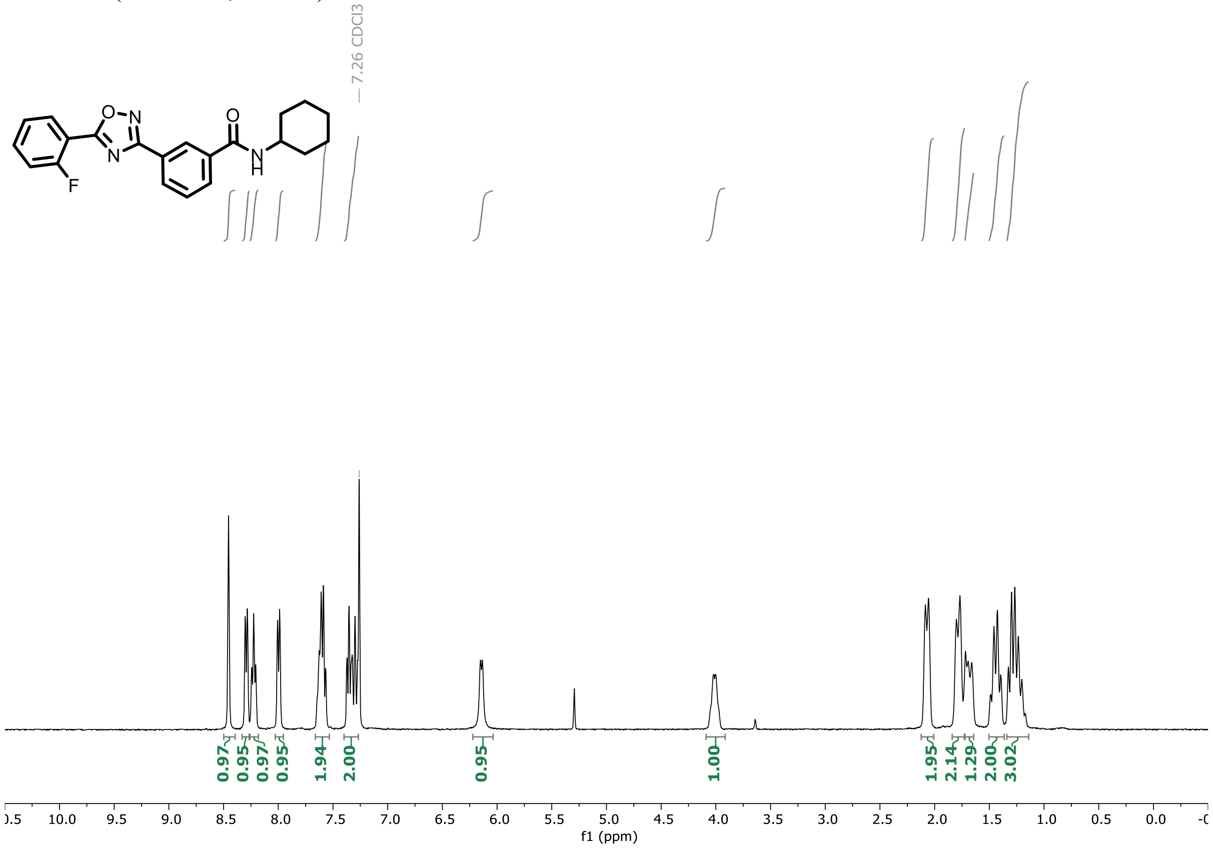

^{13}C NMR (101 MHz, $CDCl_3$) of **2t**

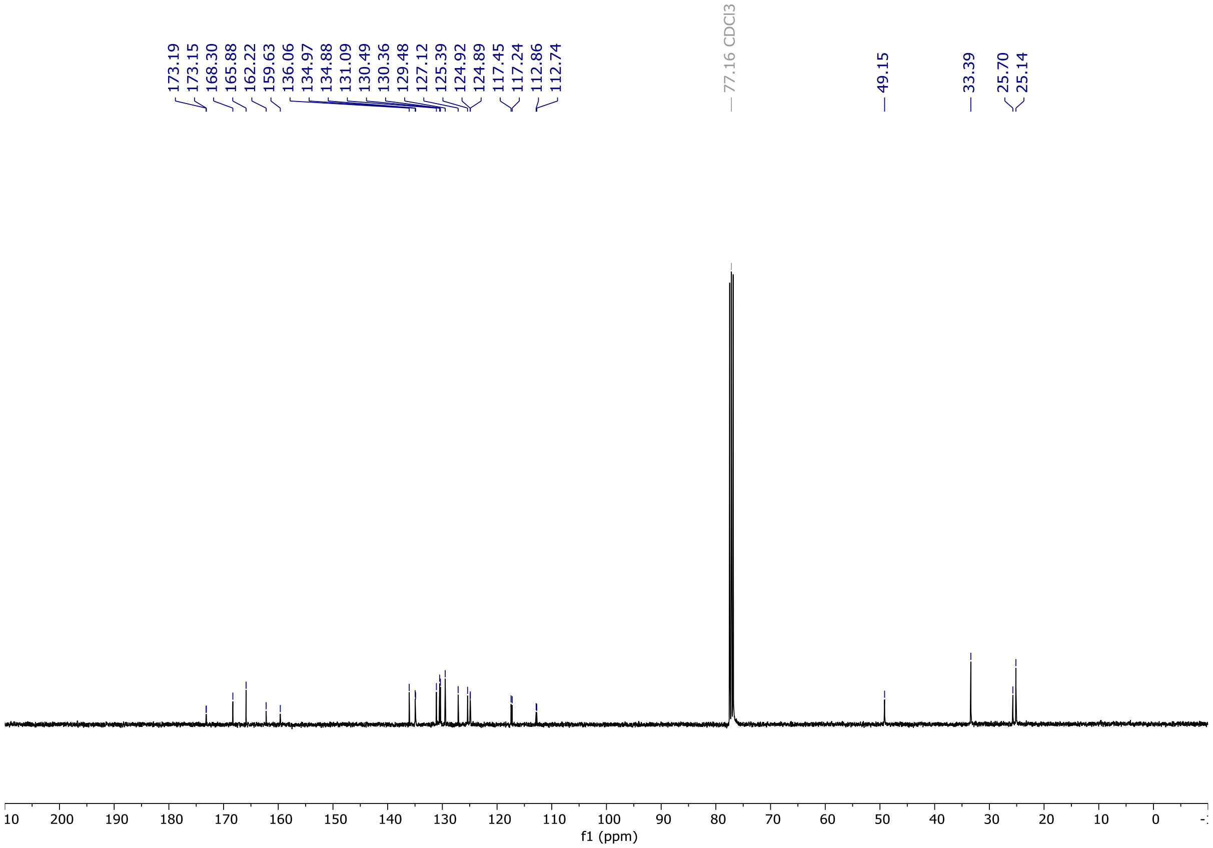

^{19}F NMR (282 MHz, $CDCl_3$) of **2t**

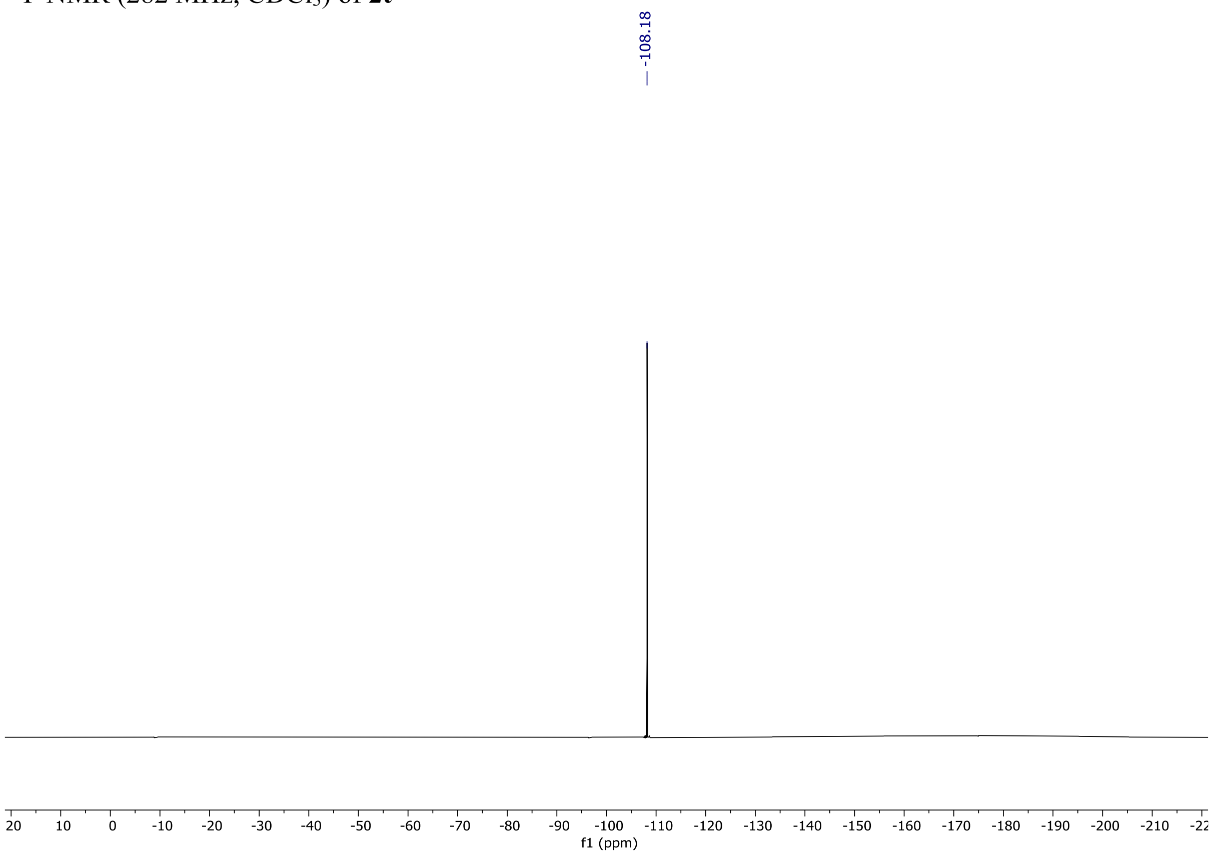

^{1}H NMR (400 MHz, $CDCl_3$) of **2u**

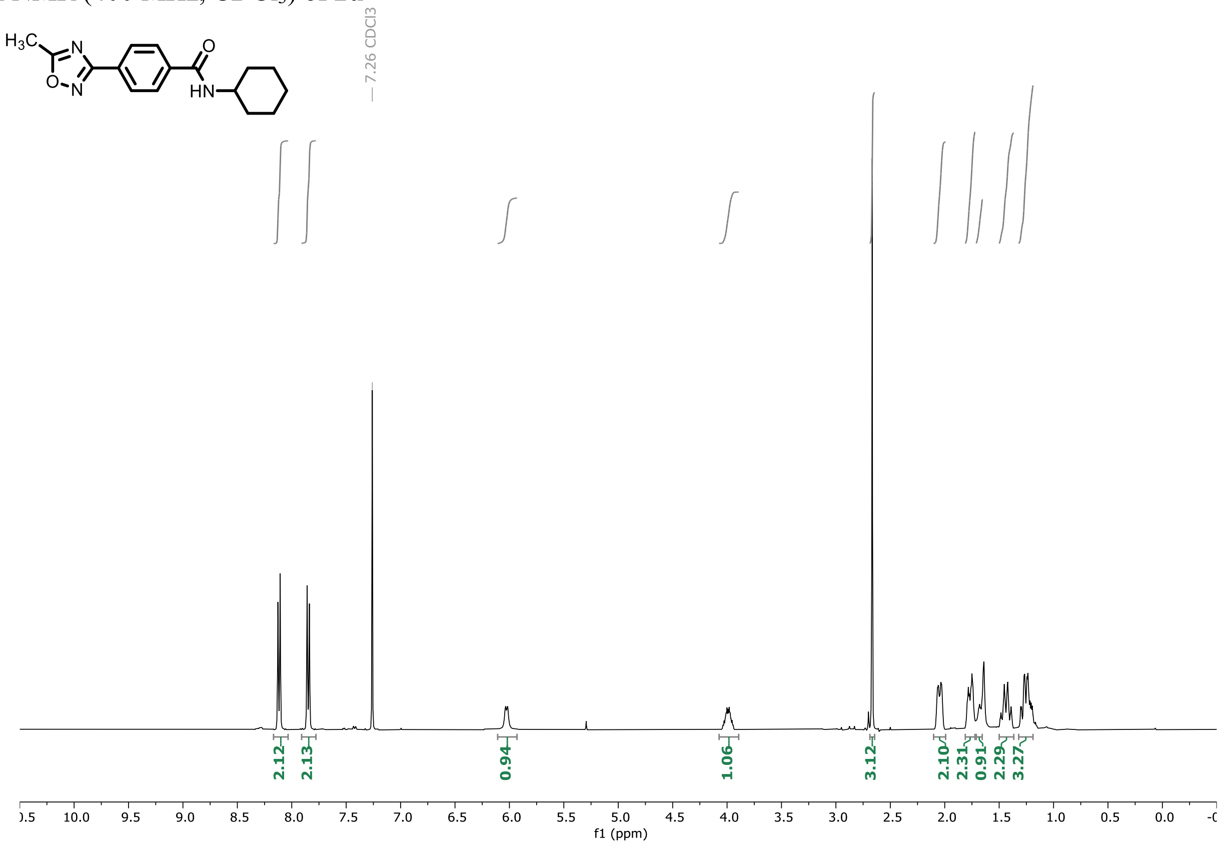

^{13}C NMR (101 MHz, $CDCl_3$) of **2u**

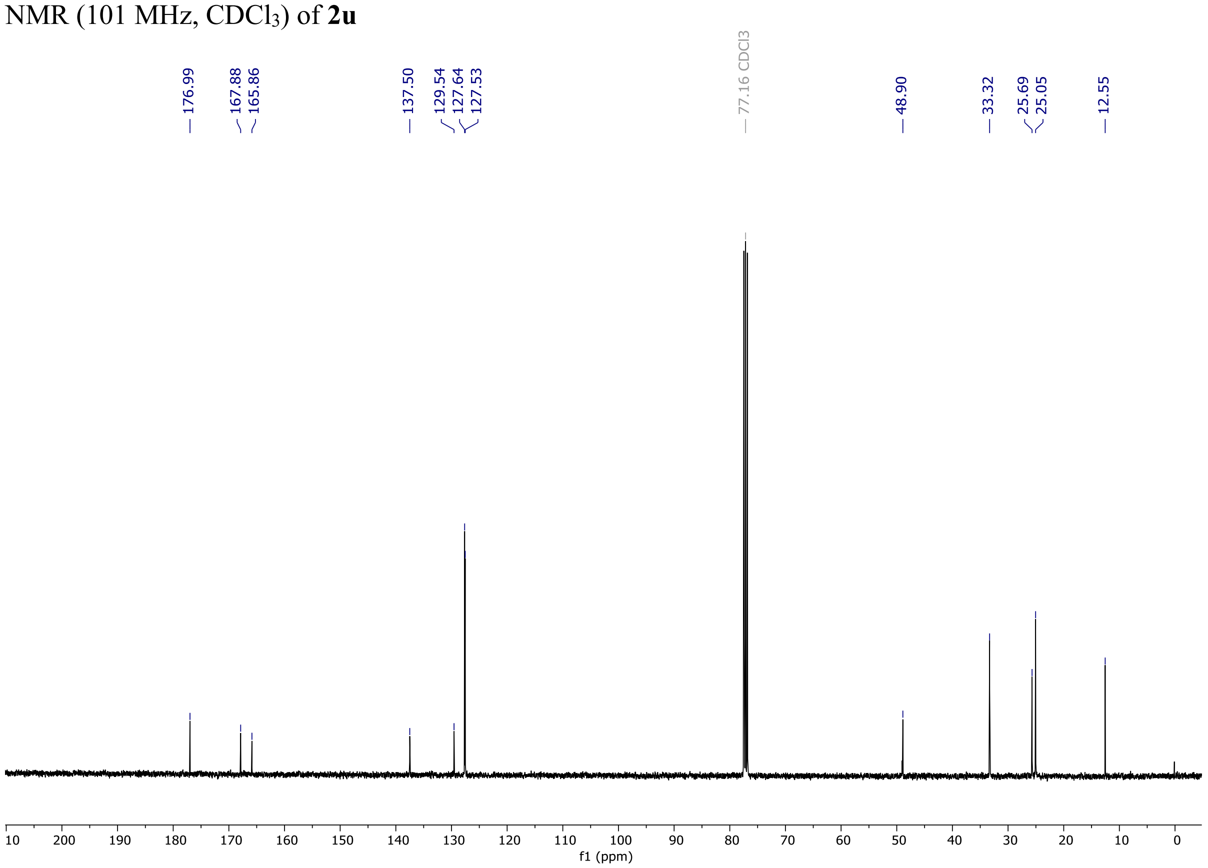

^{1}H NMR (400 MHz, $CDCl_3$) of **2v**

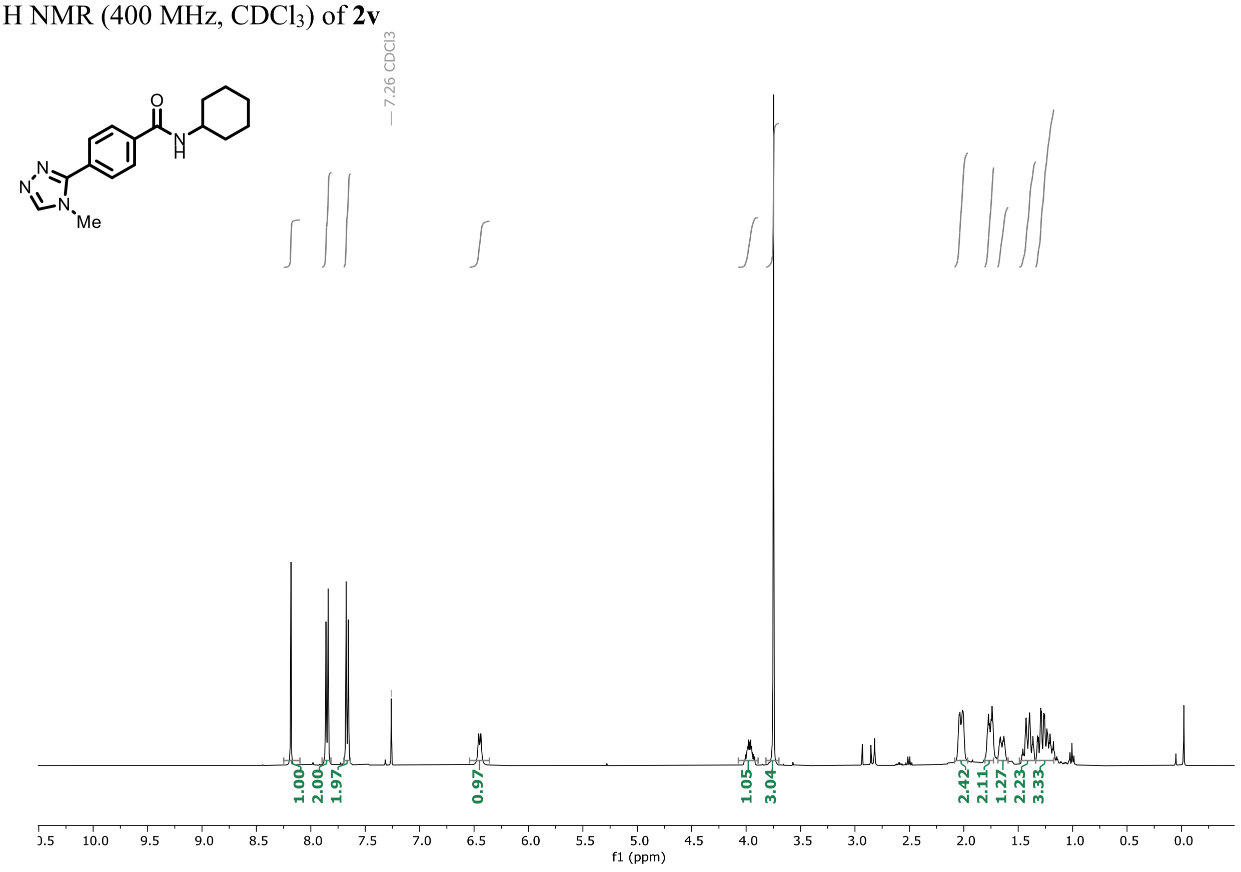

^{13}C NMR (101 MHz, $CDCl_3$) of **2v**

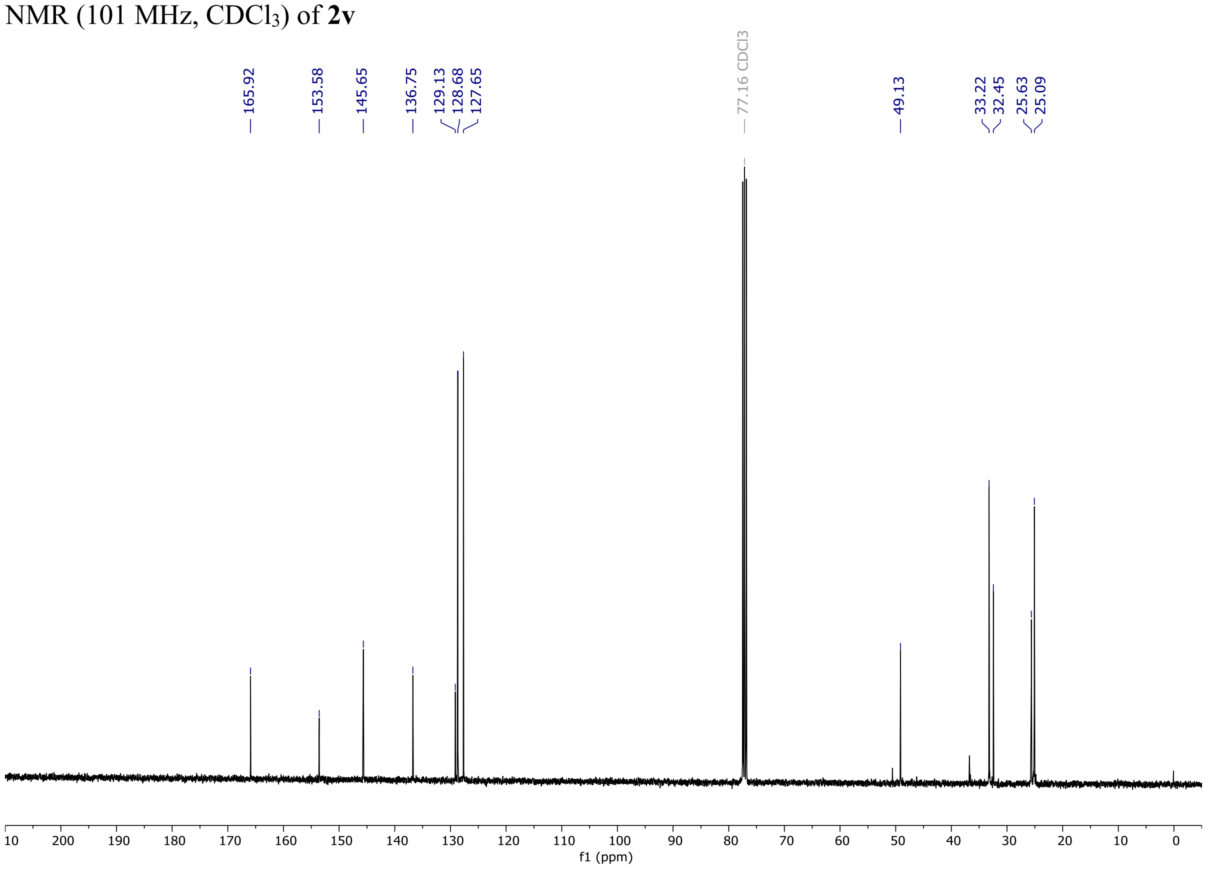

^{1}H NMR (400 MHz, $CDCl_3$) of **2z**

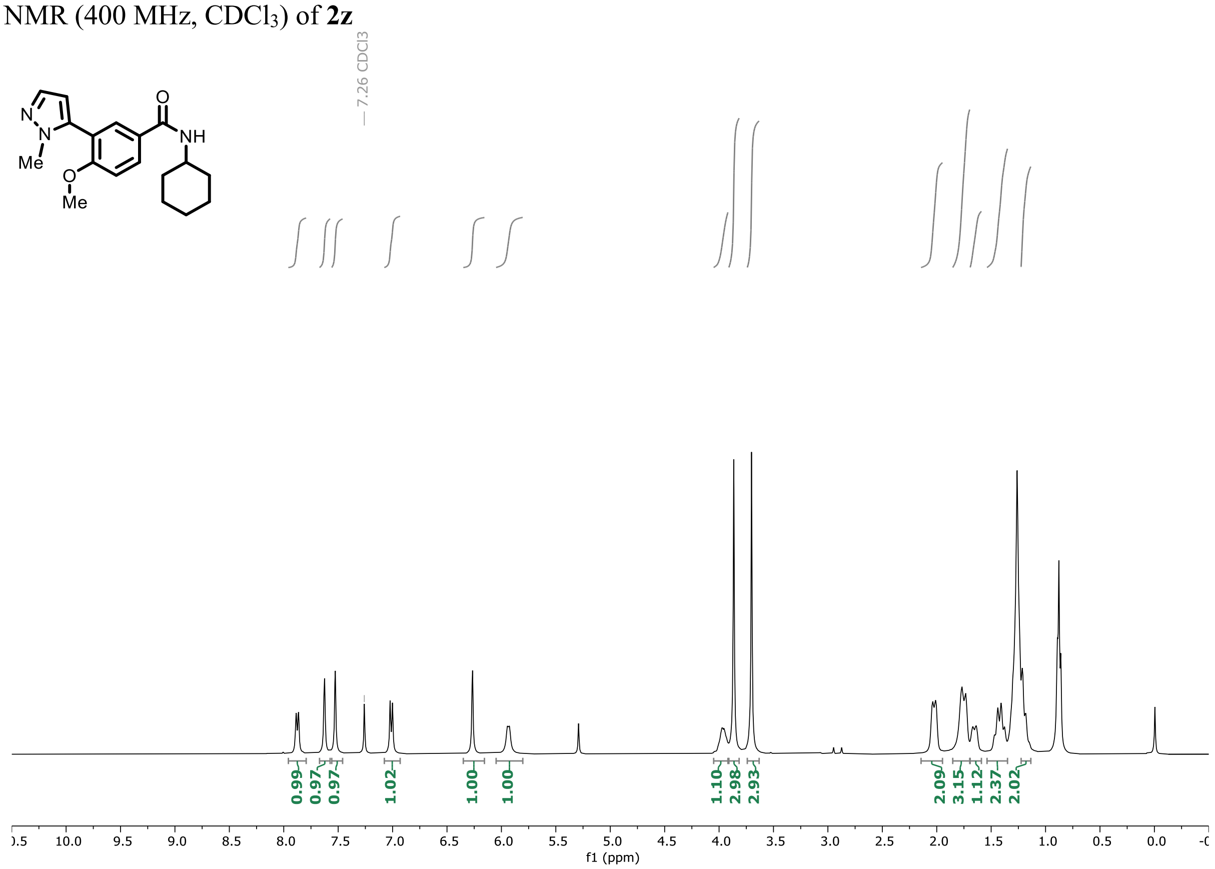

^{13}C NMR (101 MHz, $CDCl_3$) of **2z**

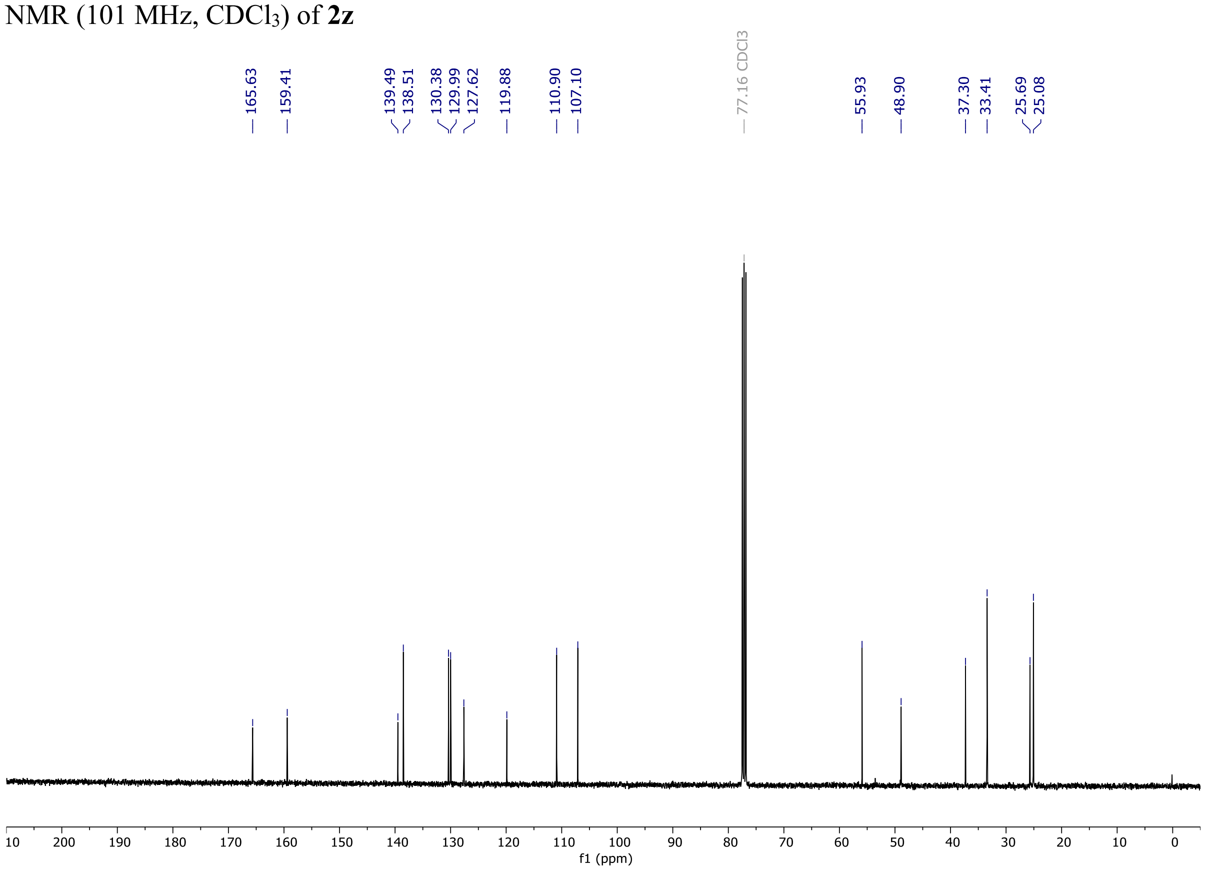

^{1}H NMR (400 MHz, $CDCl_3$) of **2aa**

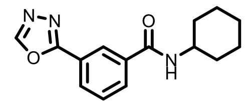

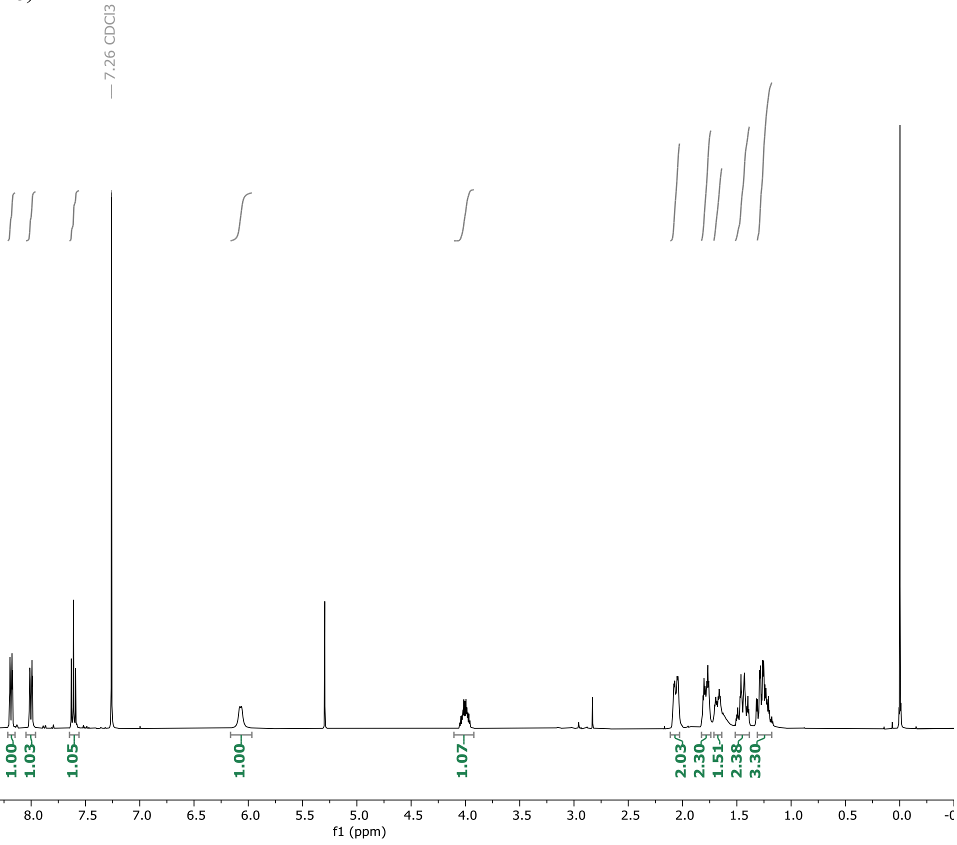

^{13}C NMR (101 MHz, $CDCl_3$) of **2aa**

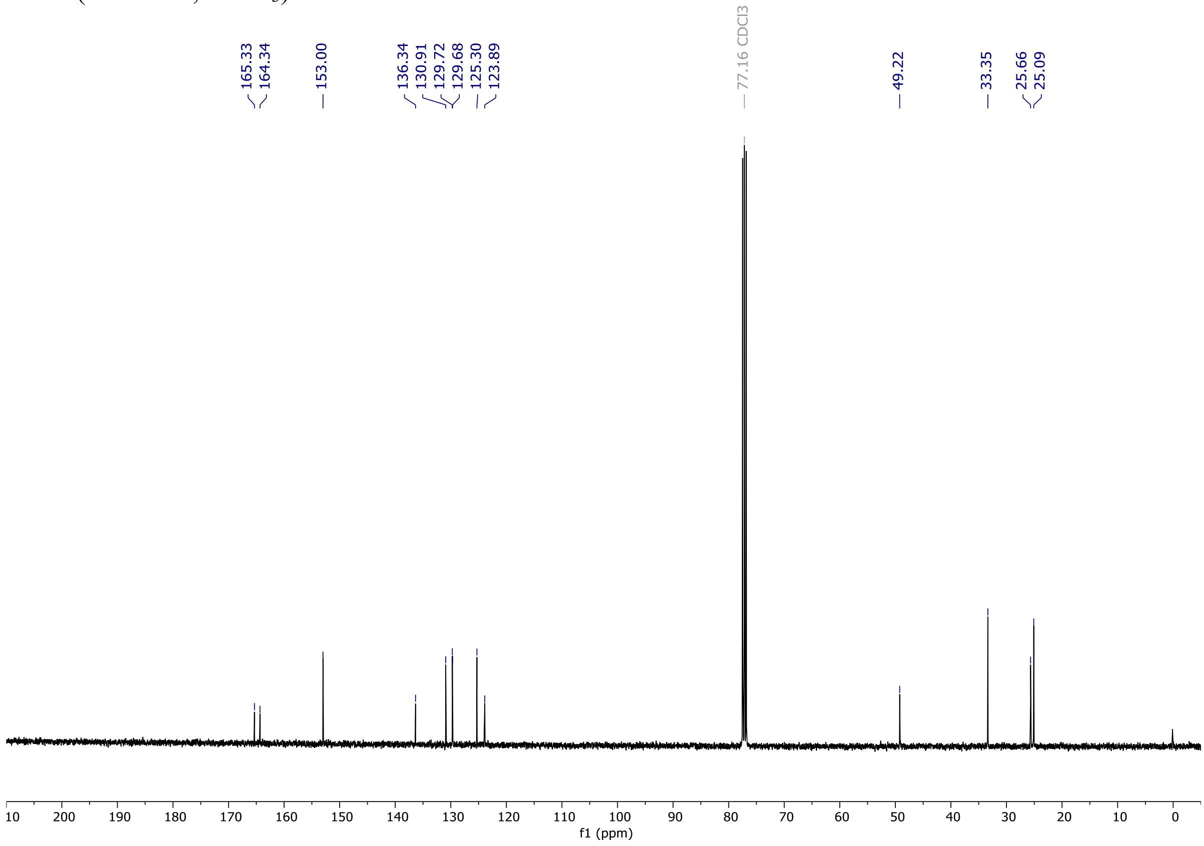

^{1}H NMR (400 MHz, $CDCl_3$) of **2ad**

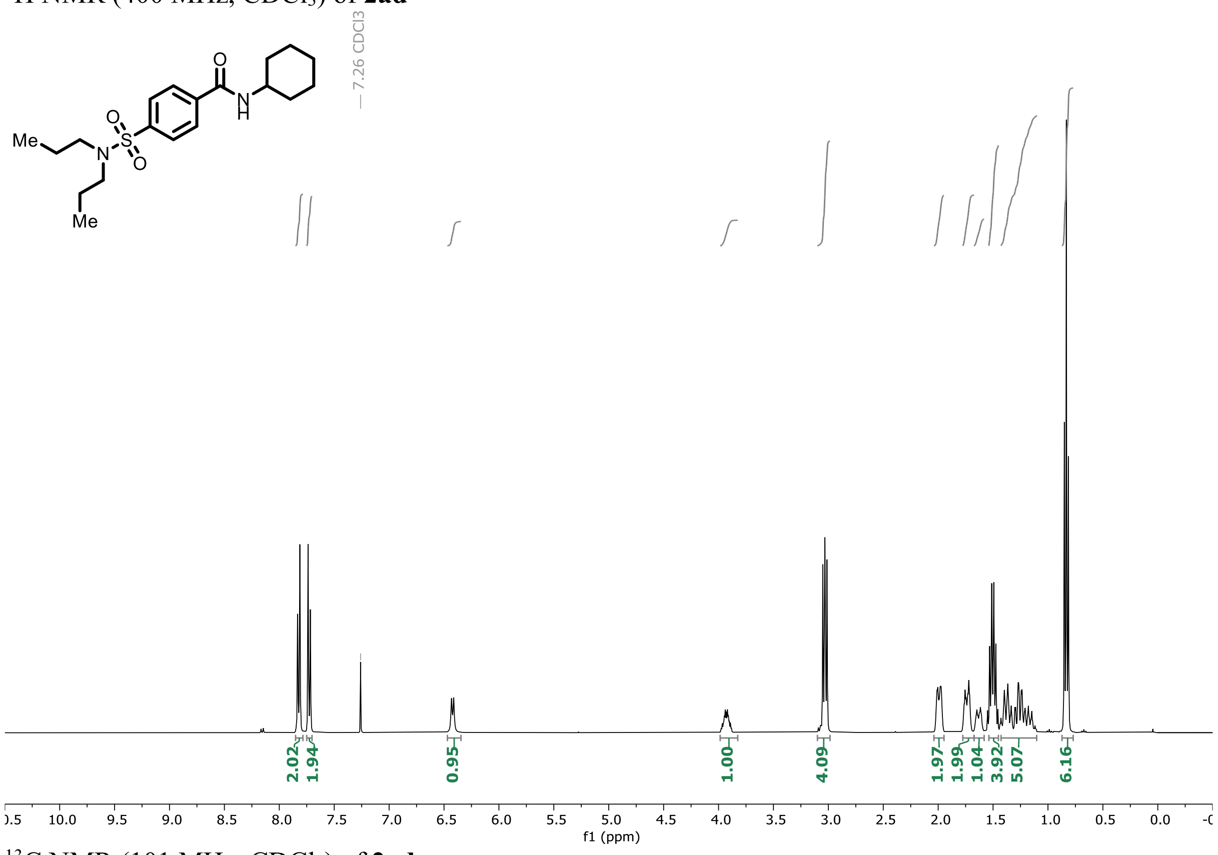

^{13}C NMR (101 MHz, $CDCl_3$) of **2ad**

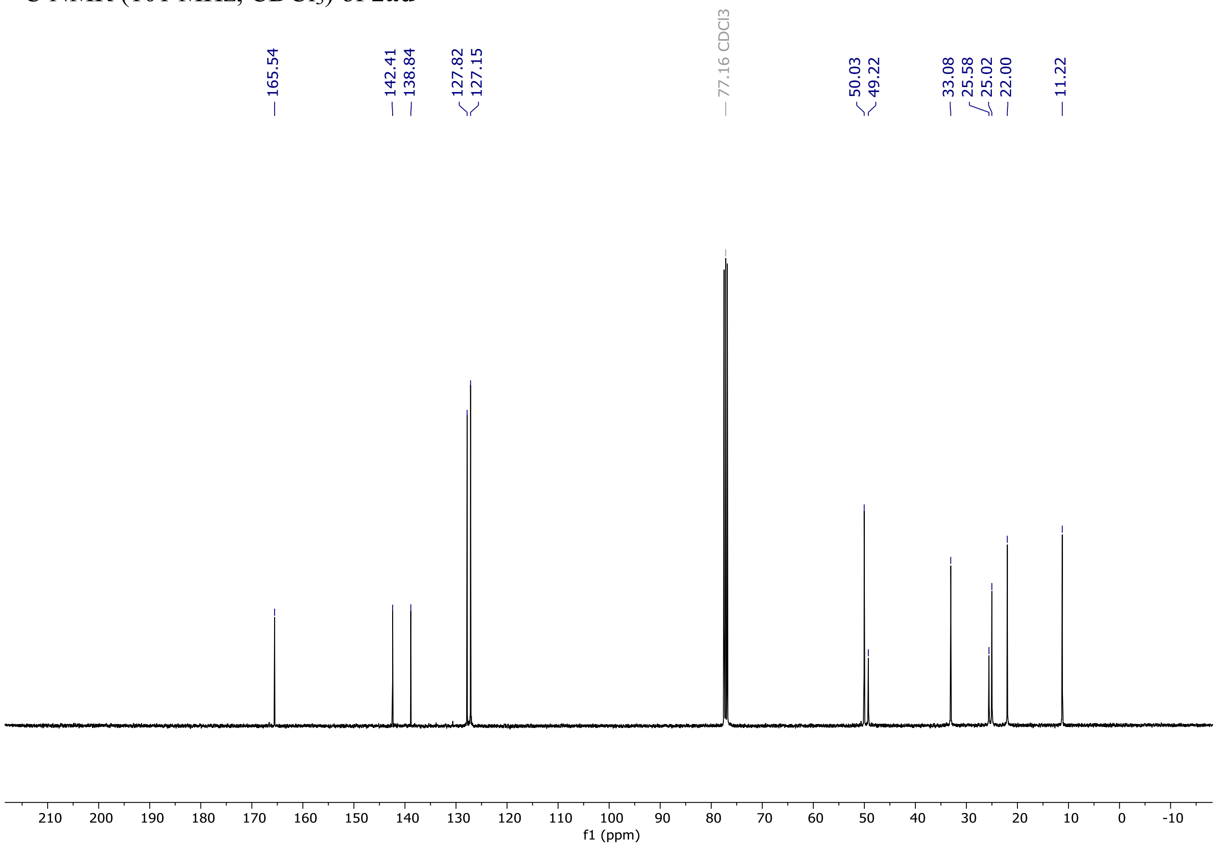

^{1}H NMR (400 MHz, $CDCl_3$) of **2ae** (* = impurity)

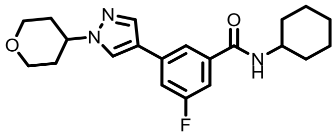

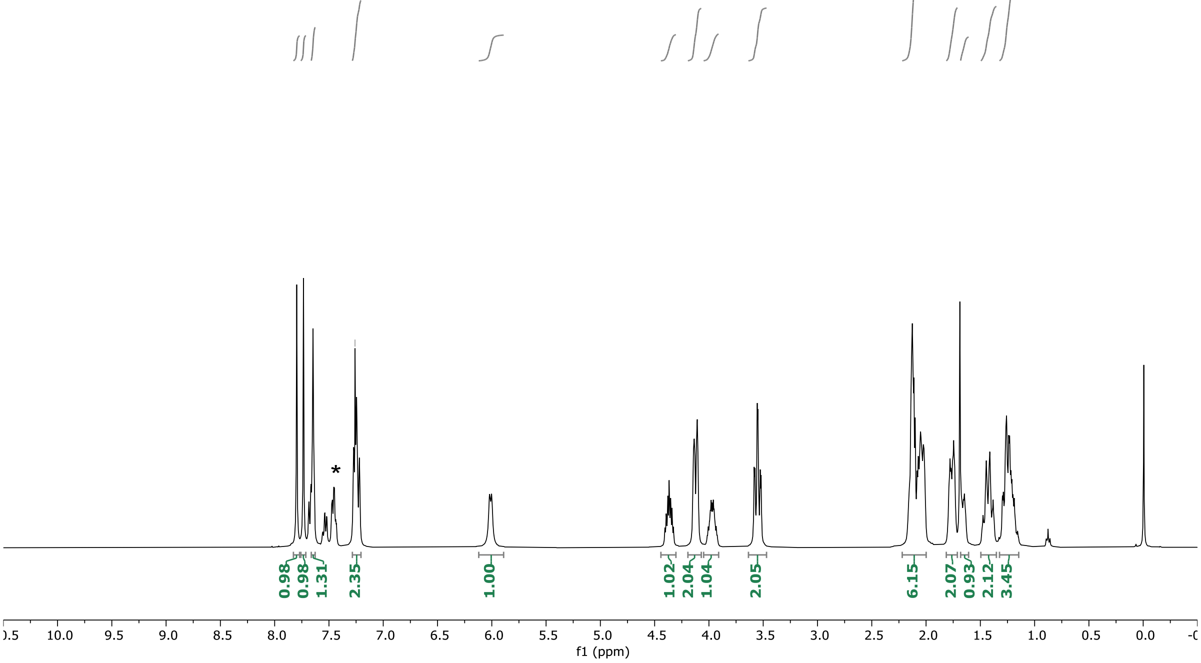

^{13}C NMR (101 MHz, $CDCl_3$) of **2ae**

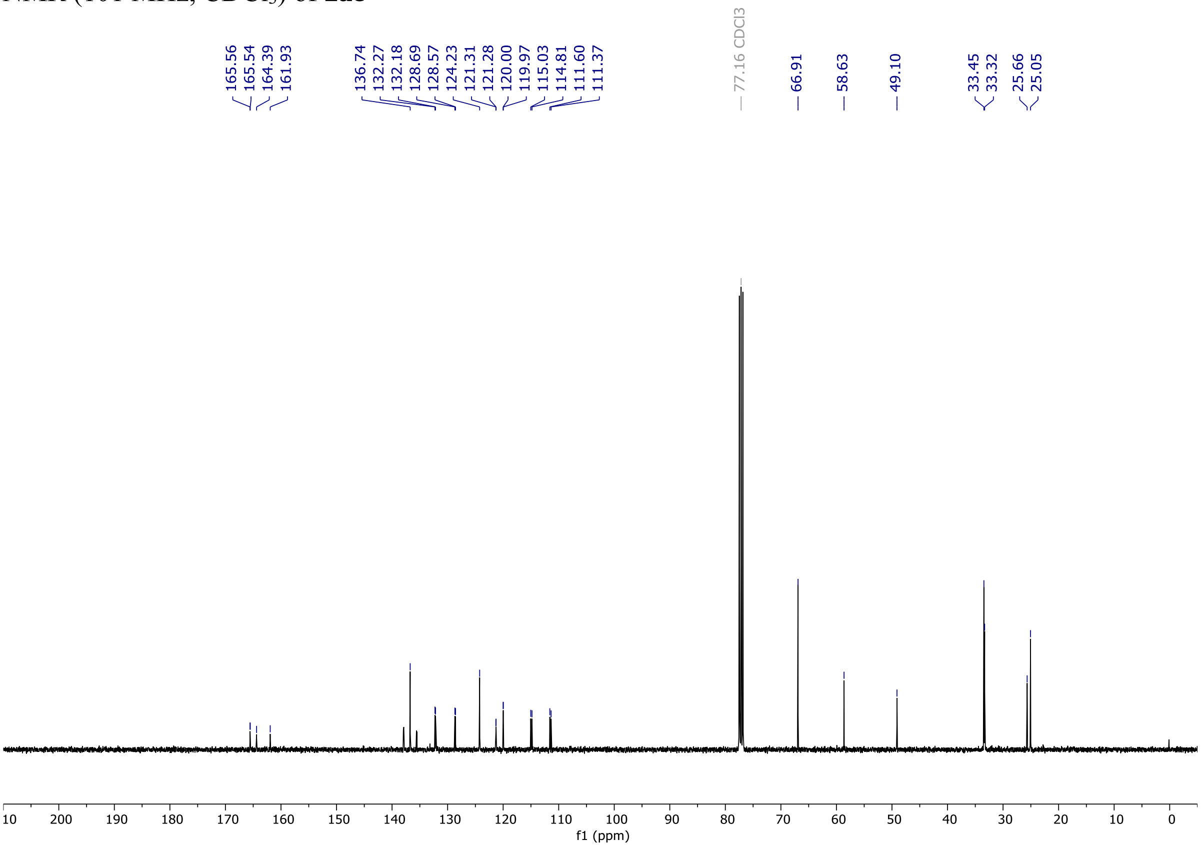

^{19}F NMR (377 MHz, $CDCl_3$)of **2ae**

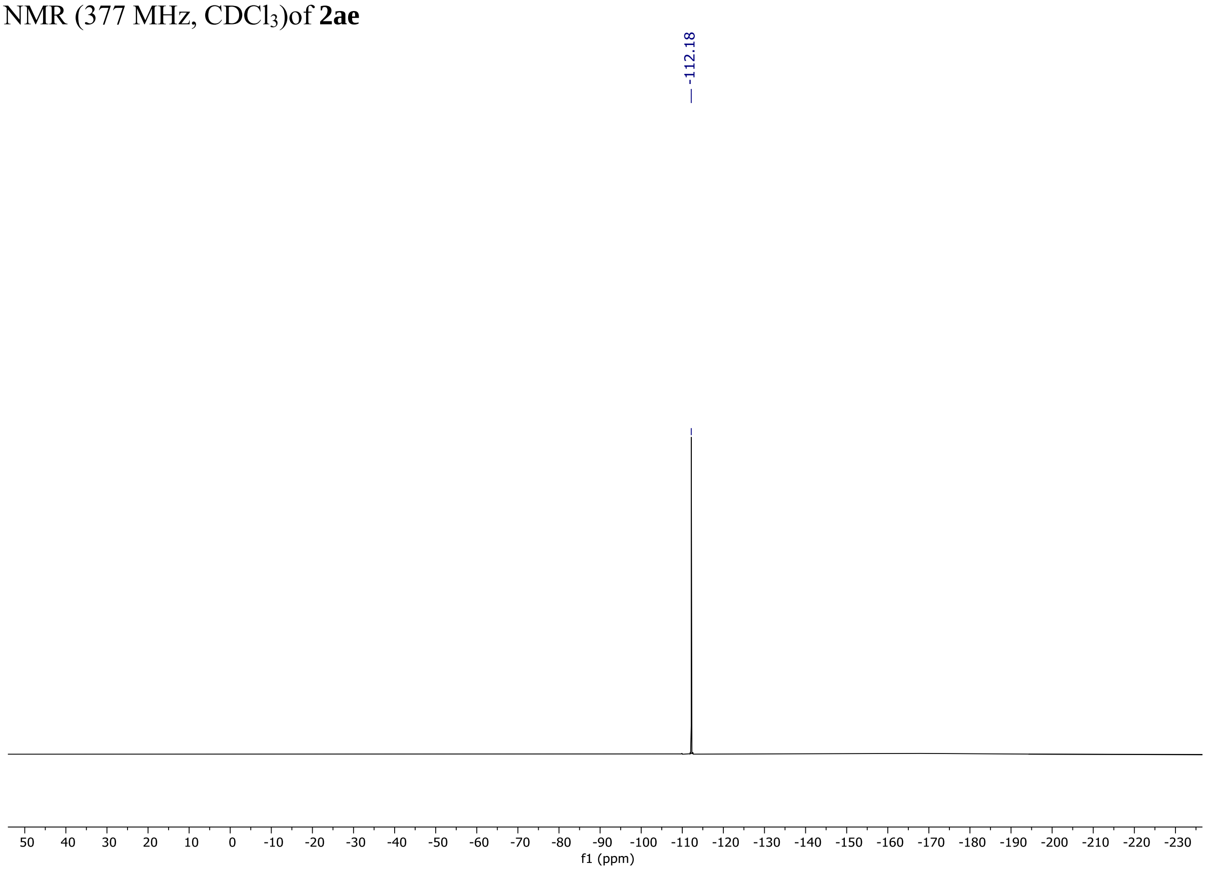

^{1}H NMR (400 MHz, $CDCl_3$) of **3b**

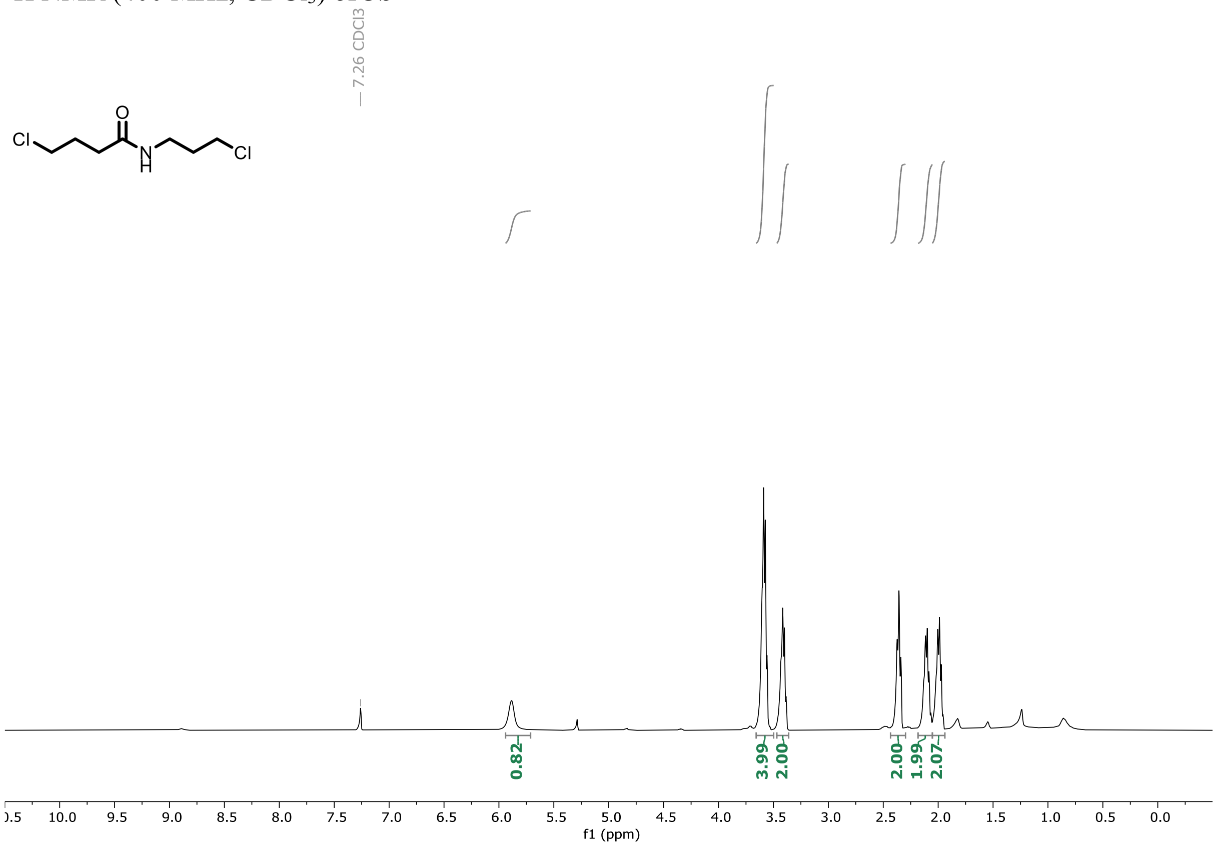

^{13}C NMR (101 MHz, $CDCl_3$) of **3b**

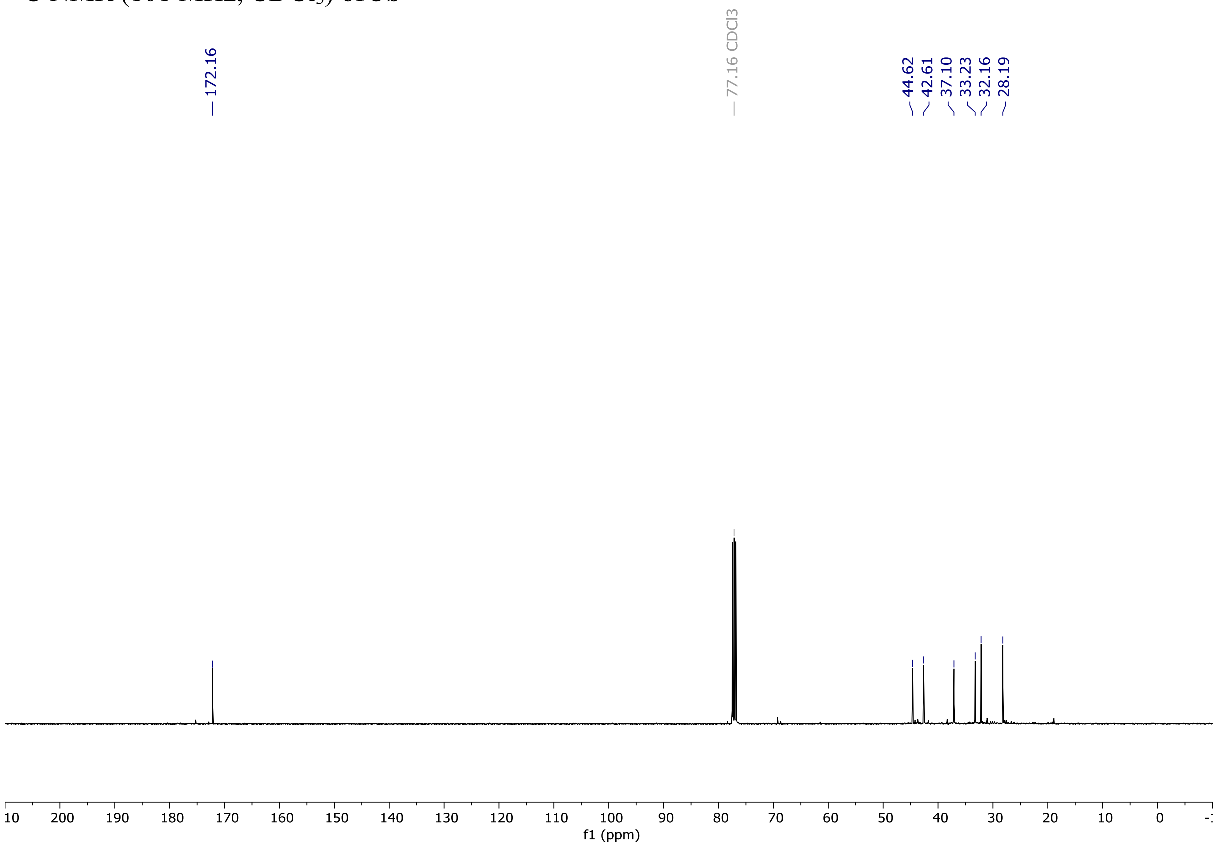

^{1}H NMR (400 MHz, $CDCl_3$) of **3d**

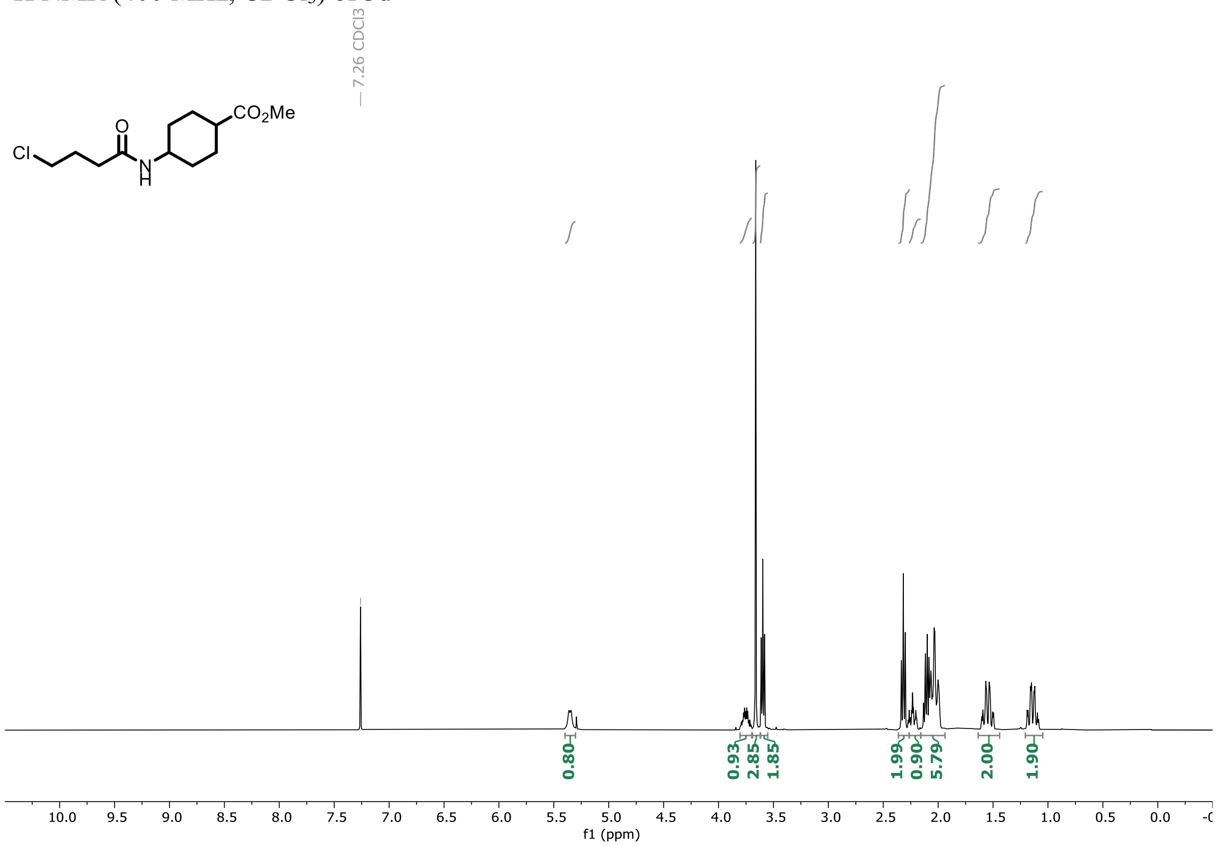

^{13}C NMR (101 MHz, $CDCl_3$) of **3d**

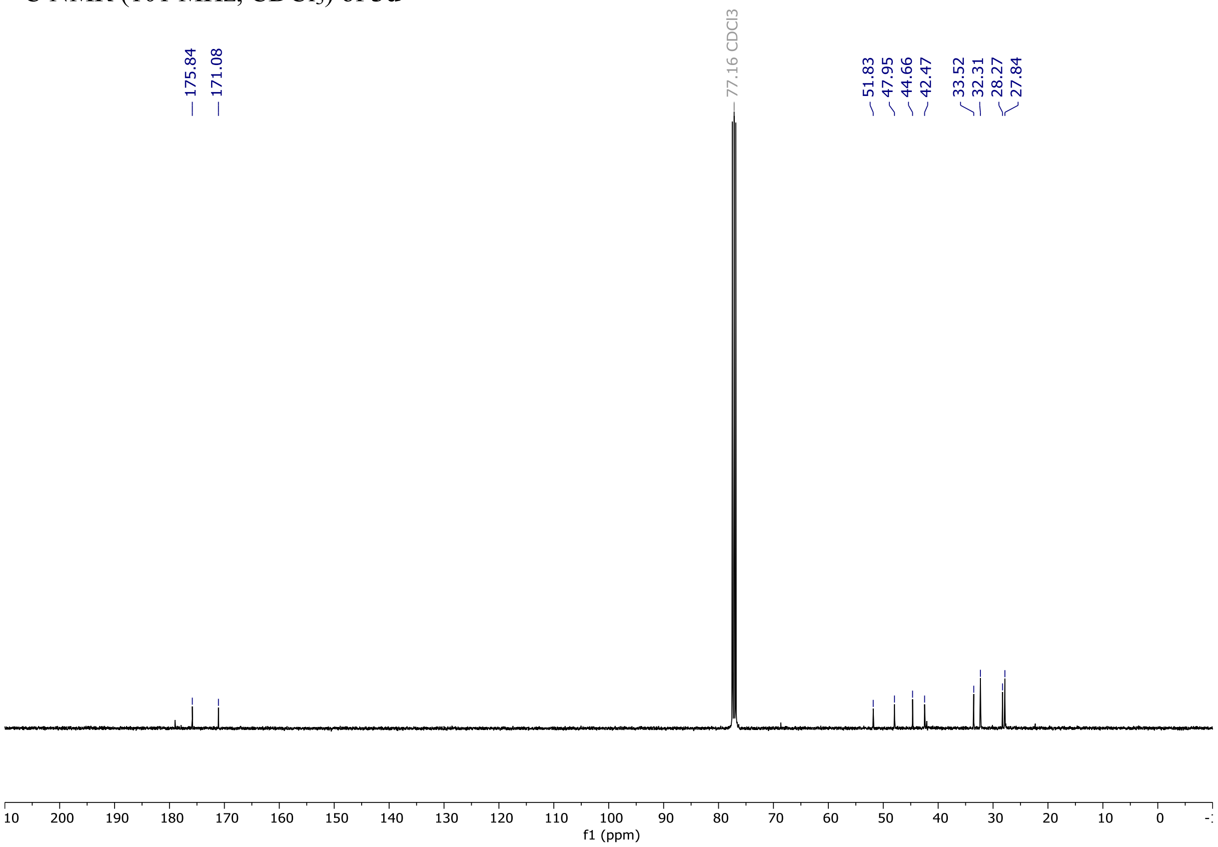

^{1}H NMR (400 MHz, $CDCl_3$) of **3f**

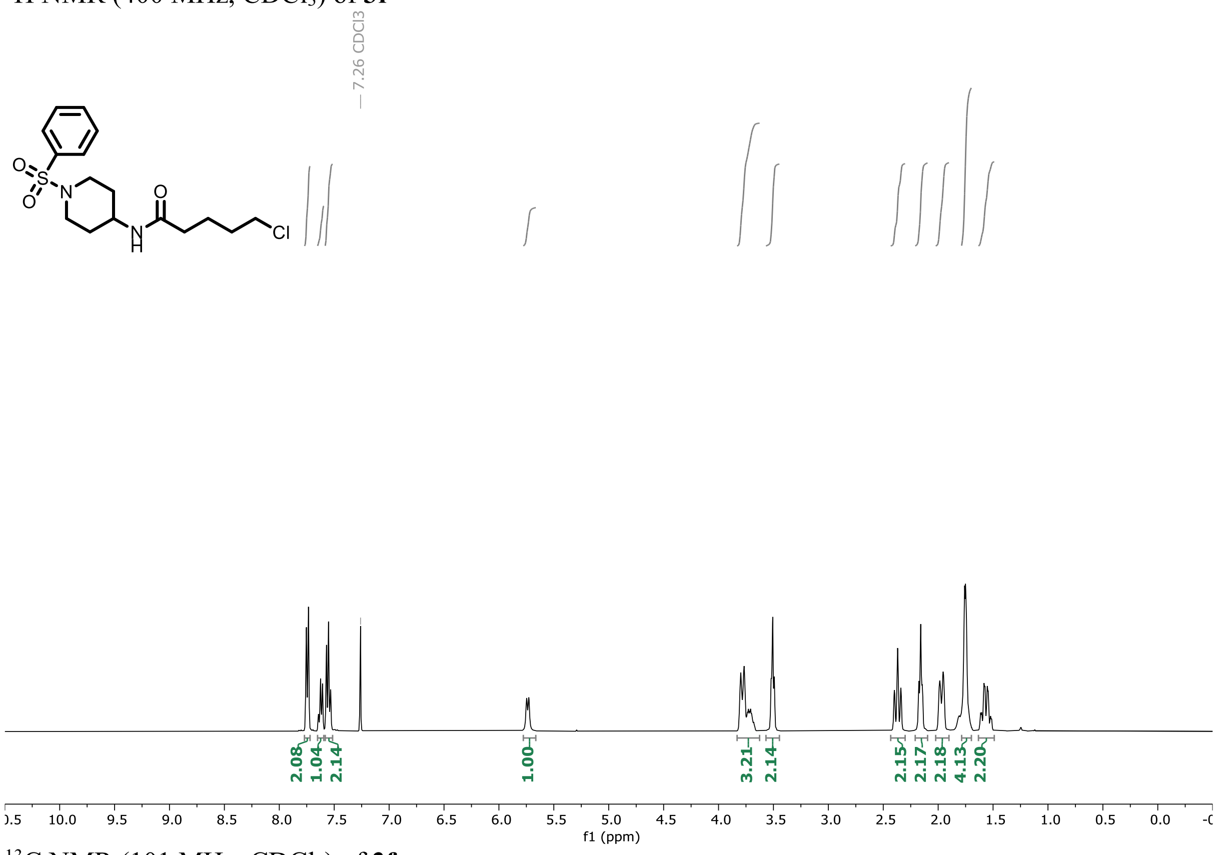

^{13}C NMR (101 MHz, $CDCl_3$) of **3f**

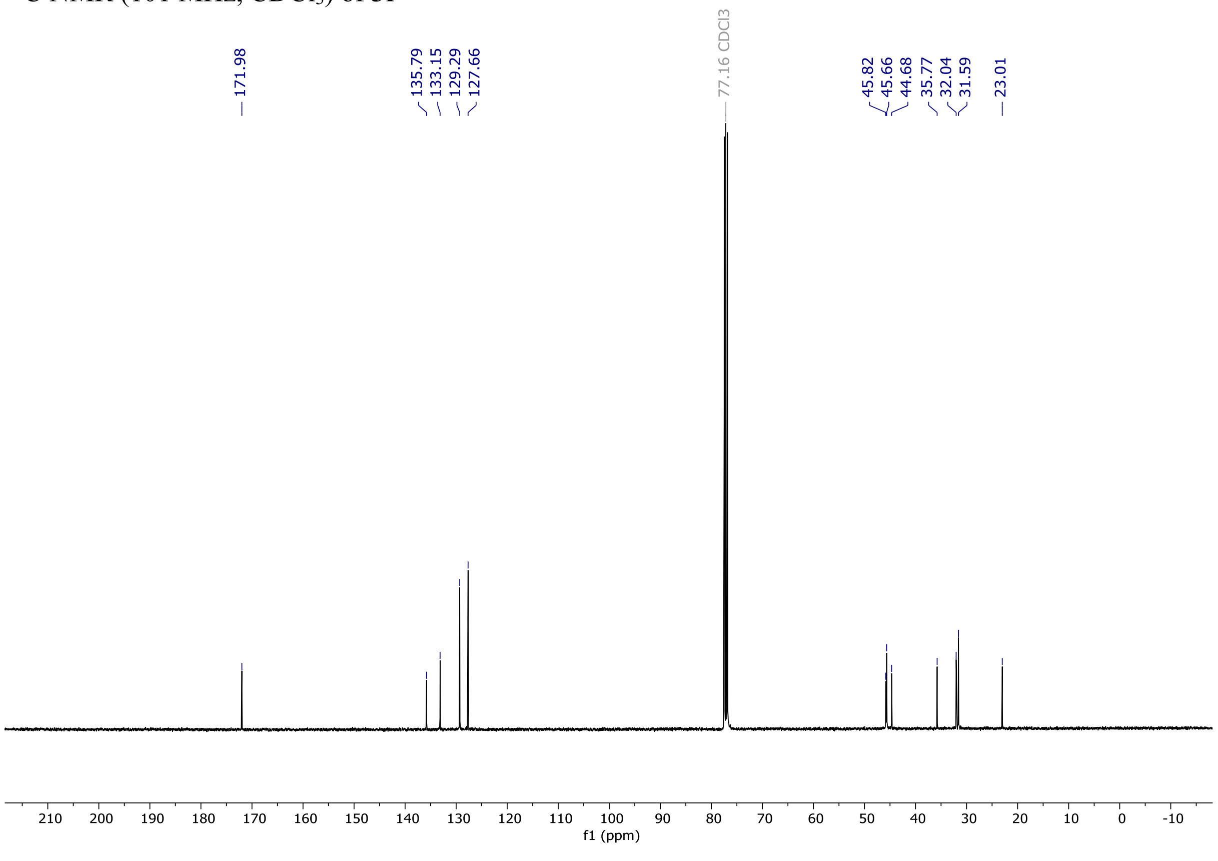

^{1}H NMR (400 MHz, $CDCl_3$) of **3g**

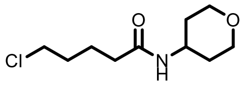

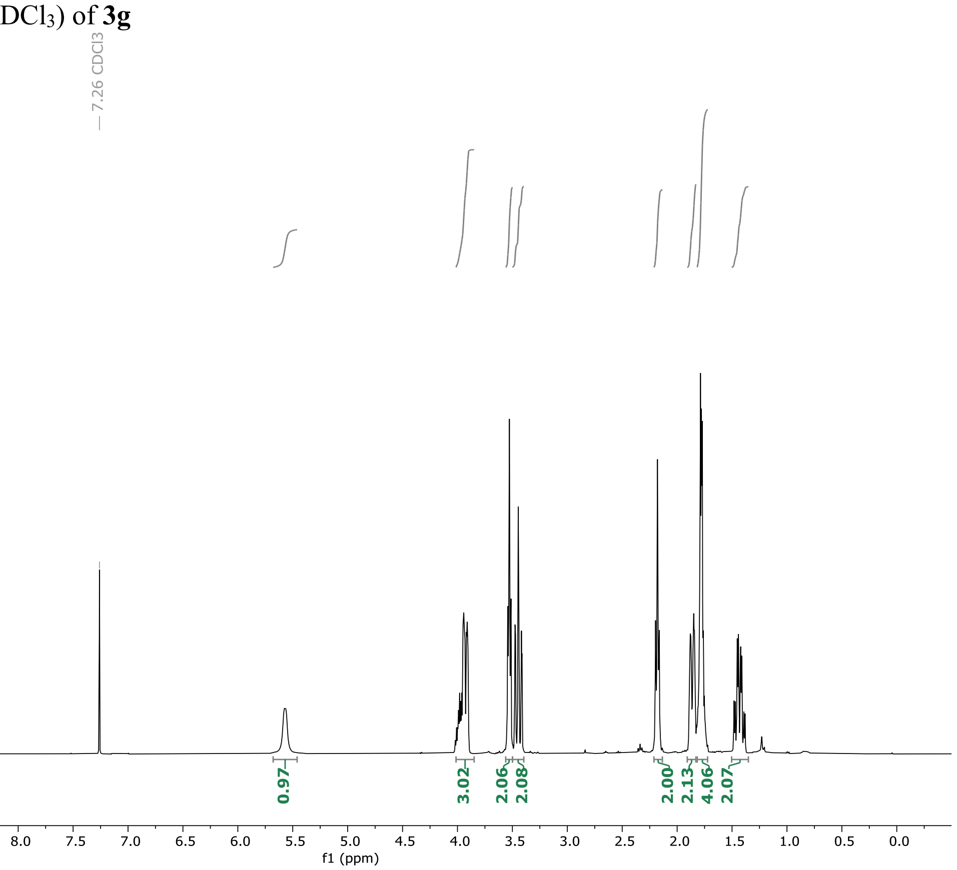

^{13}C NMR (101 MHz, $CDCl_3$) of **3g**

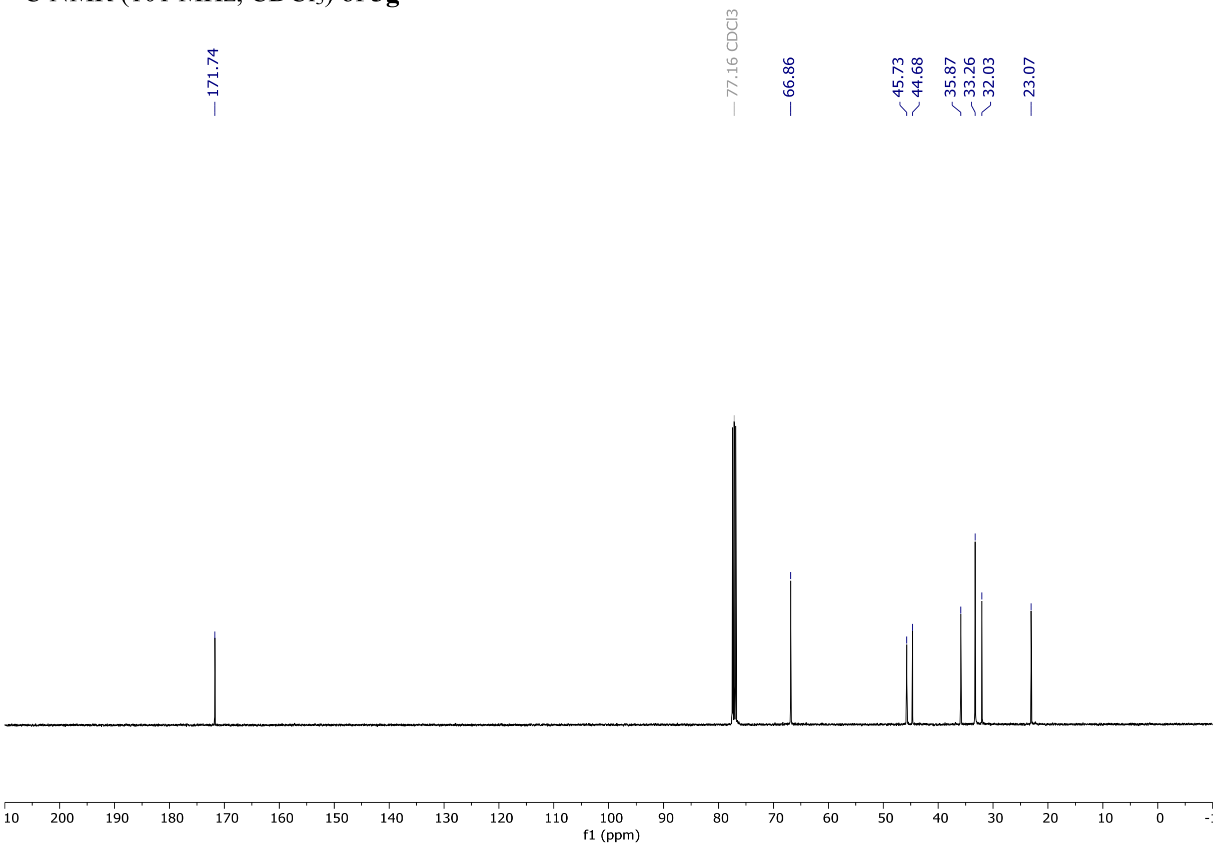

^{1}H NMR (400 MHz, $CDCl_3$) of **3h**

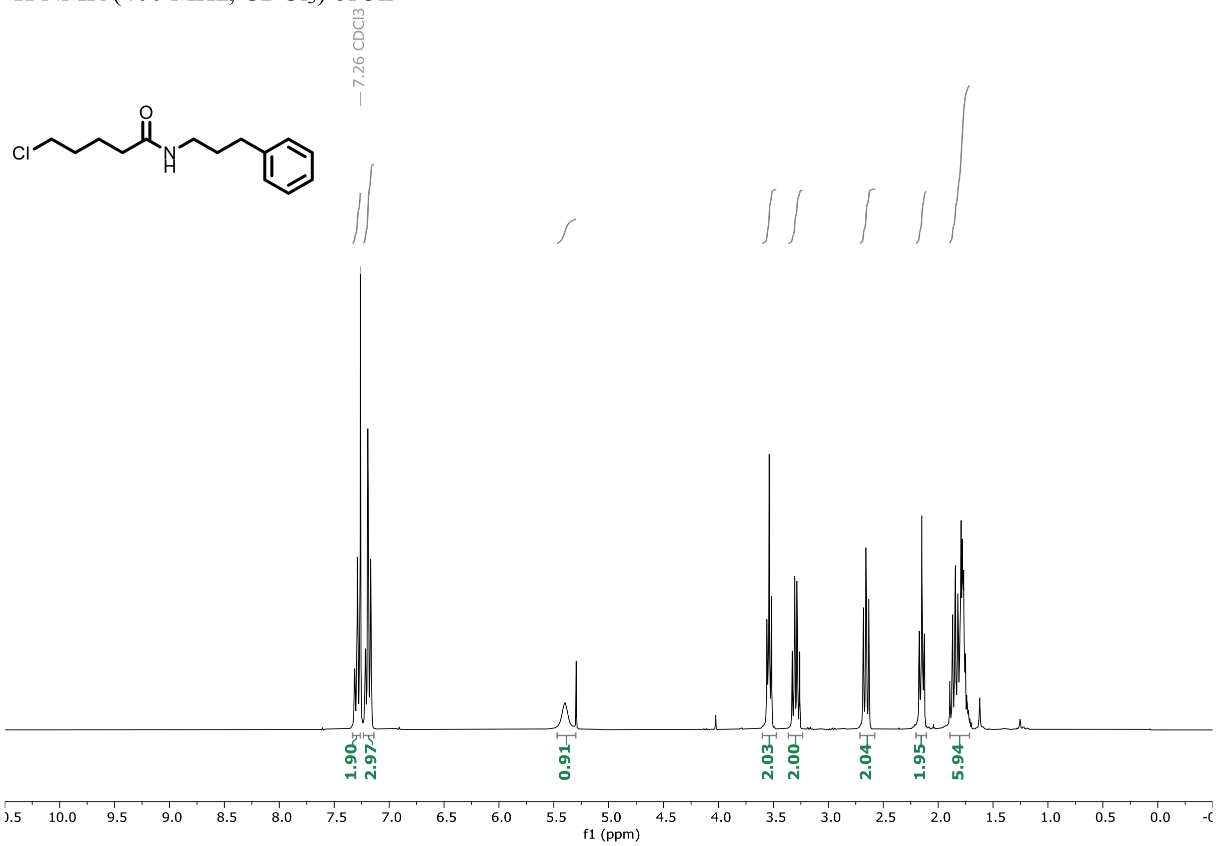

^{13}C NMR (101 MHz, $CDCl_3$) of **3h**

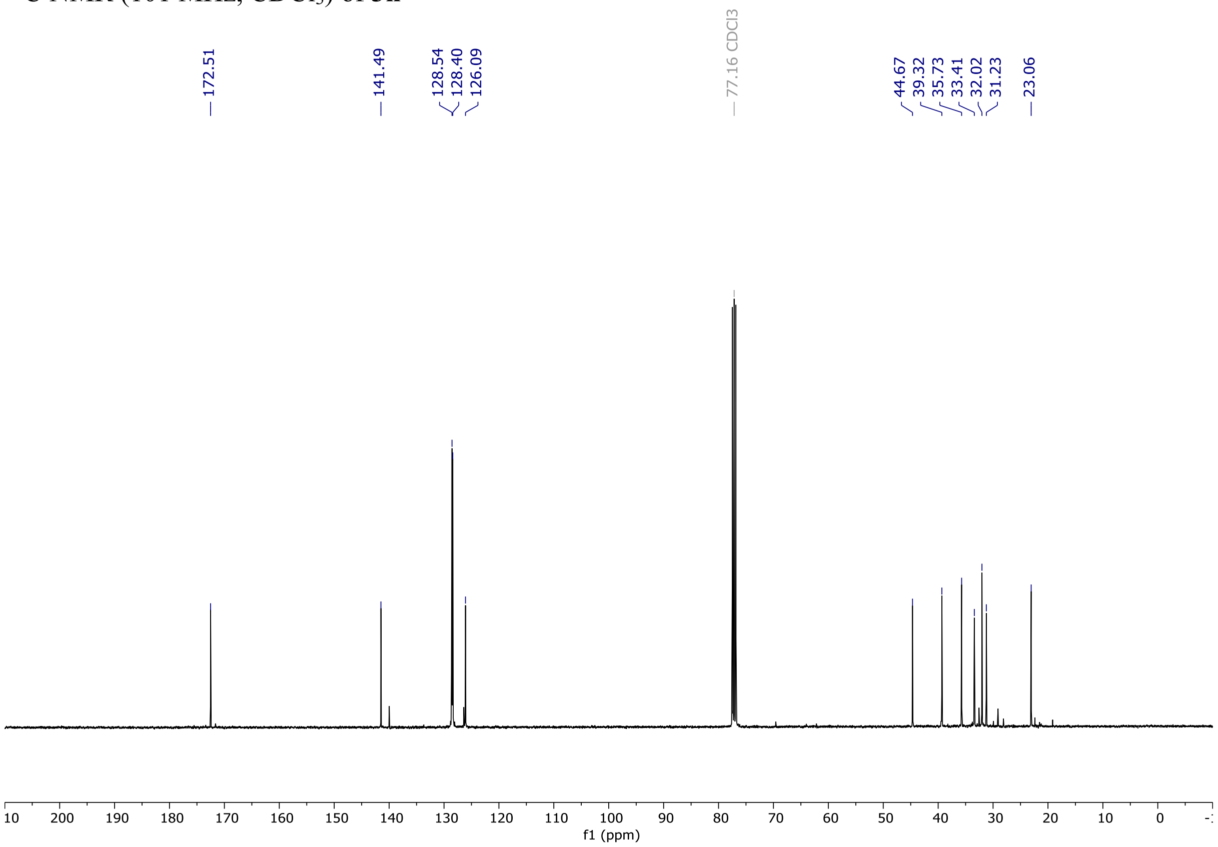

14. NMR spectra of products (5-63)

^{1}H NMR (400 MHz, $CDCl_3$) of **5**

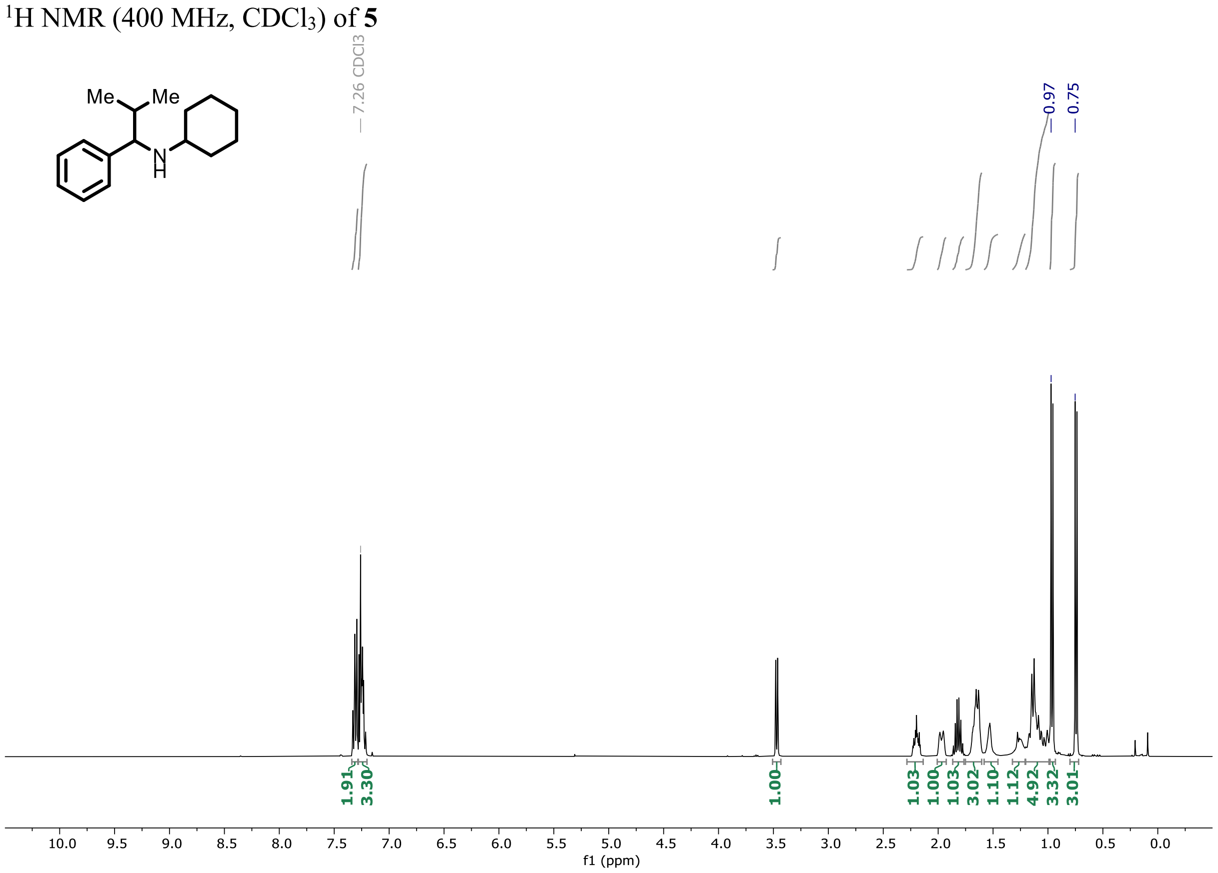

^{13}C NMR (101 MHz, $CDCl_3$) of **5**

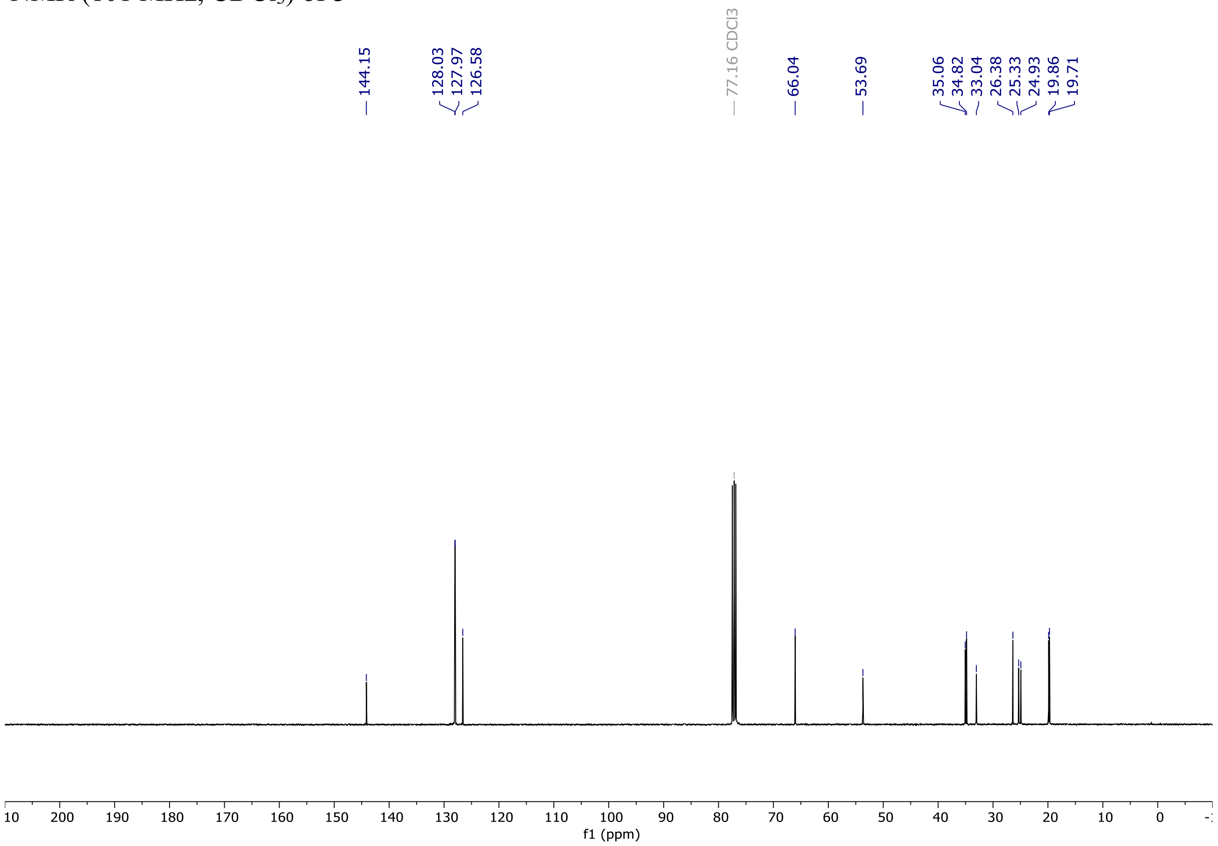

^{1}H NMR (400 MHz, $CDCl_3$) of **6**

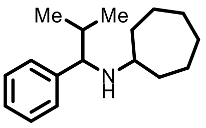

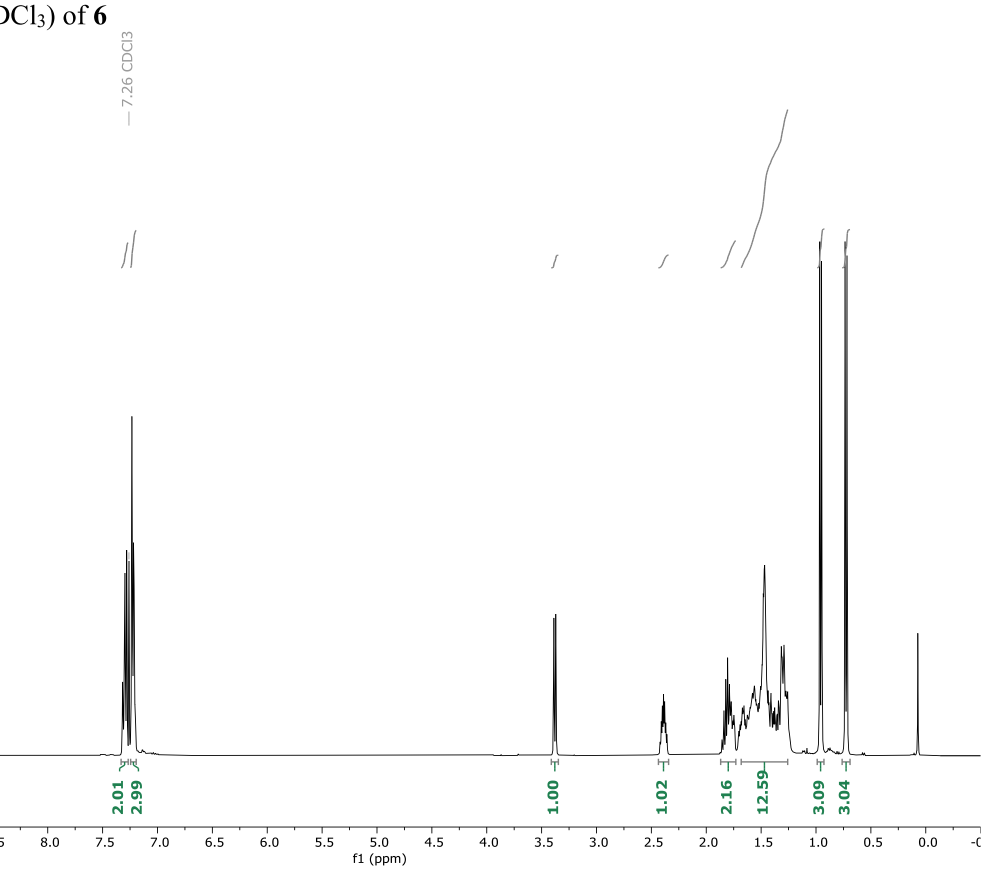

^{13}C NMR (101 MHz, $CDCl_3$) of **6**

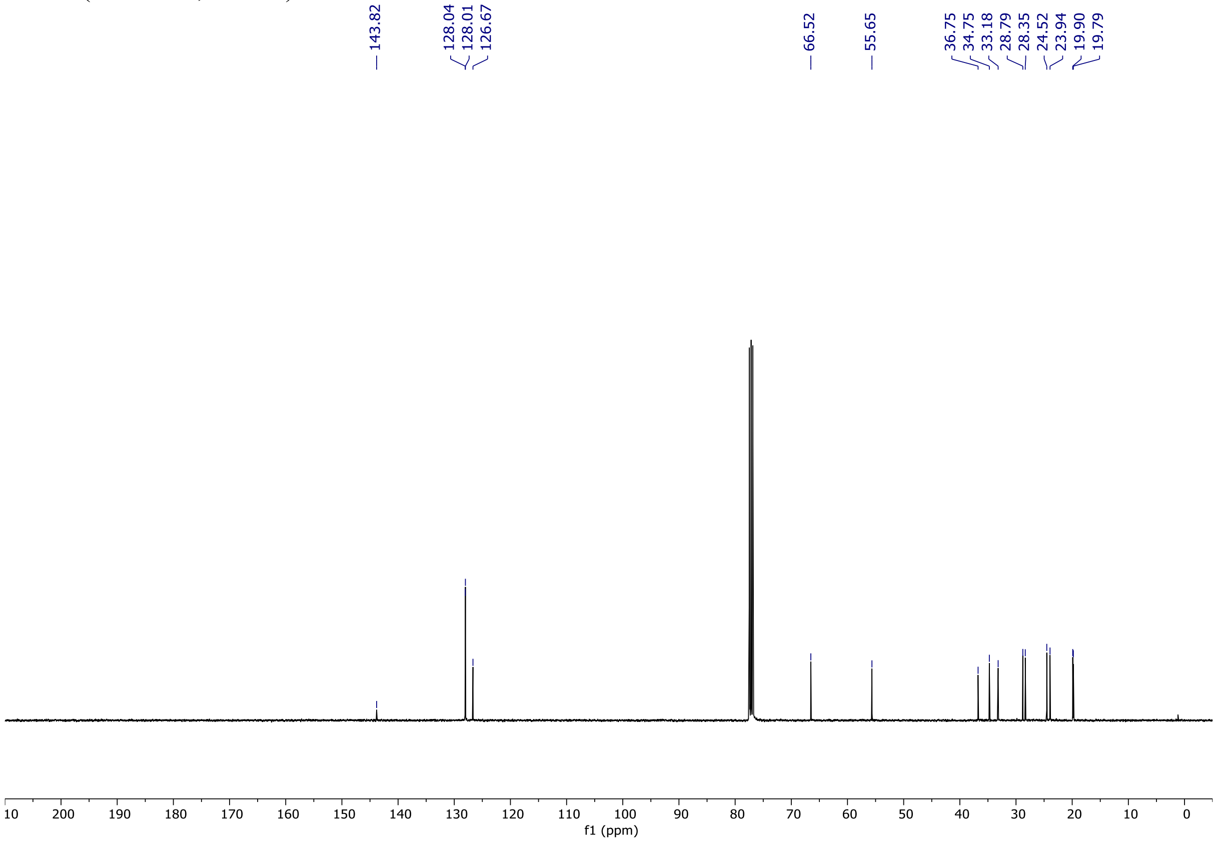

^{1}H NMR (400 MHz, $CDCl_3$) of **7**

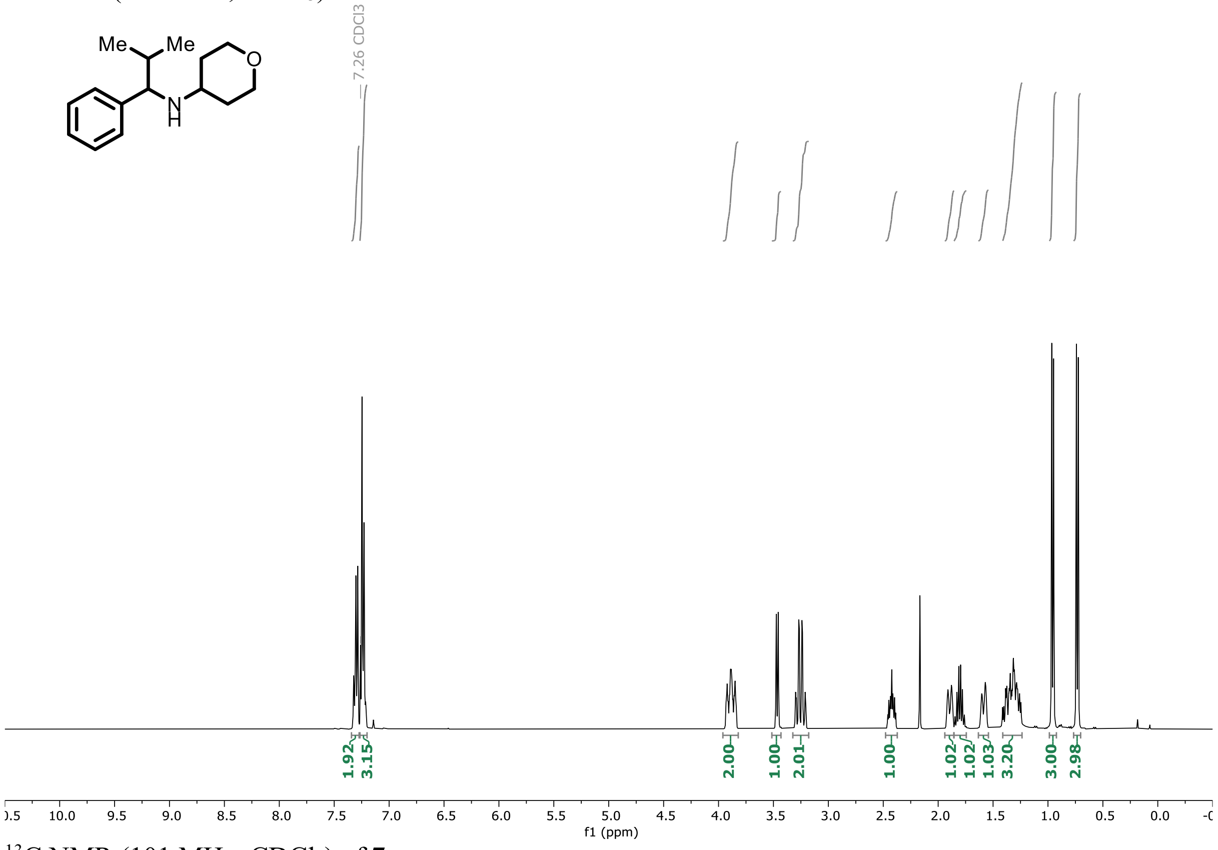

^{13}C NMR (101 MHz, $CDCl_3$) of **7**

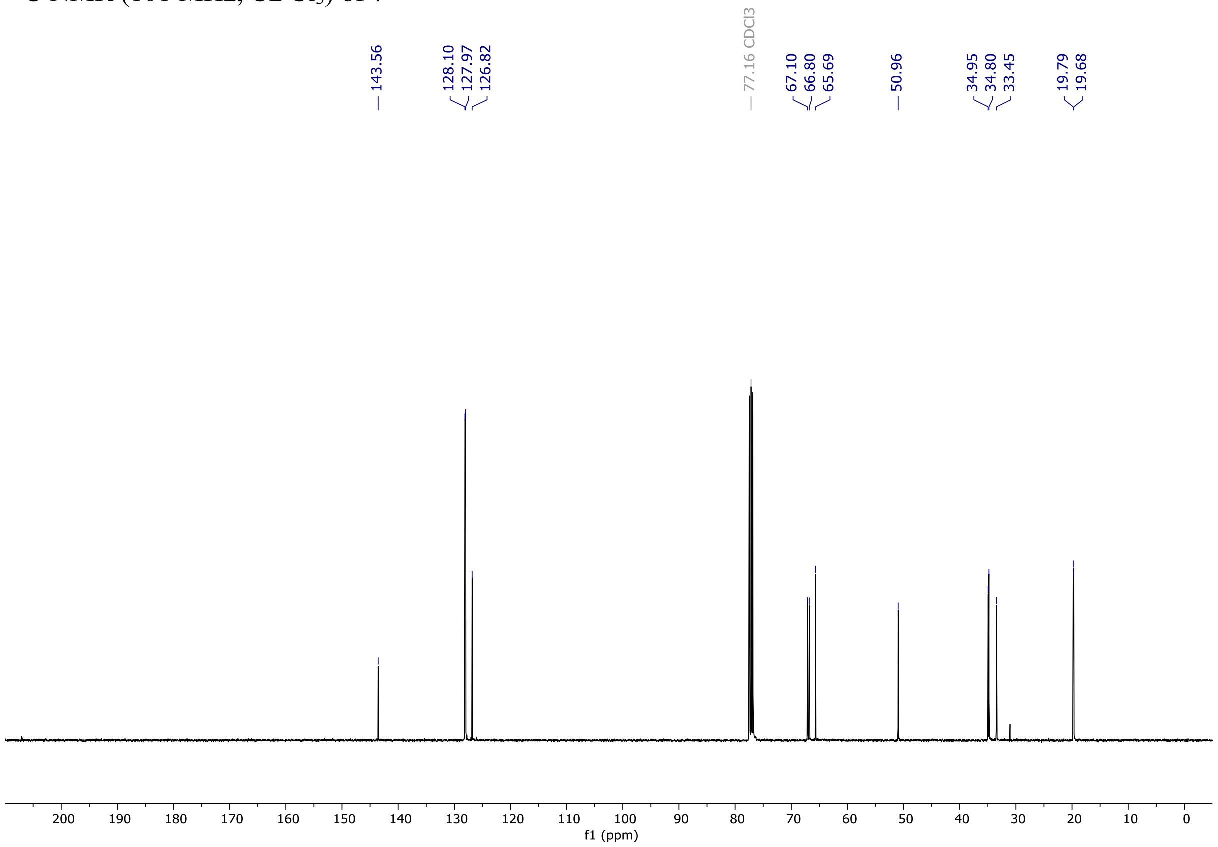

^{1}H NMR (400 MHz, $CDCl_3$) of **8**

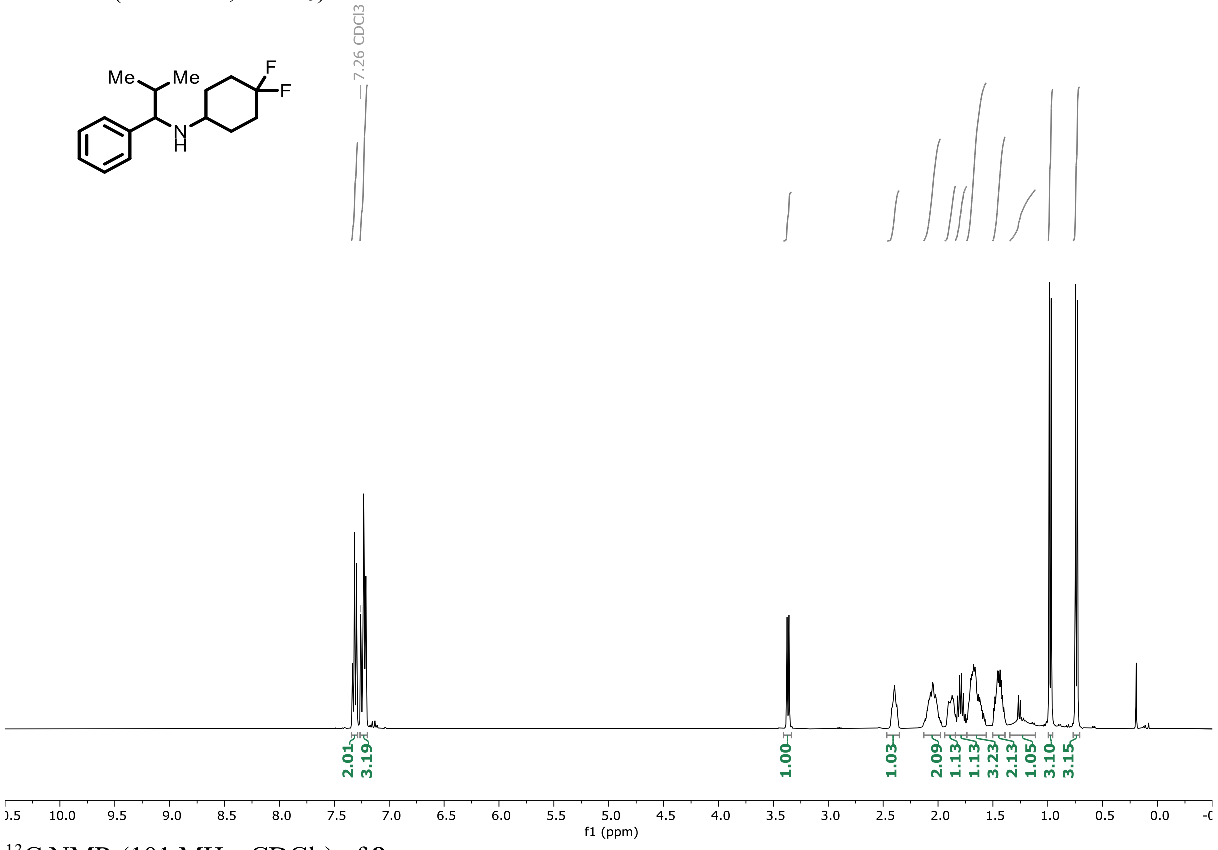

^{13}C NMR (101 MHz, $CDCl_3$) of **8**

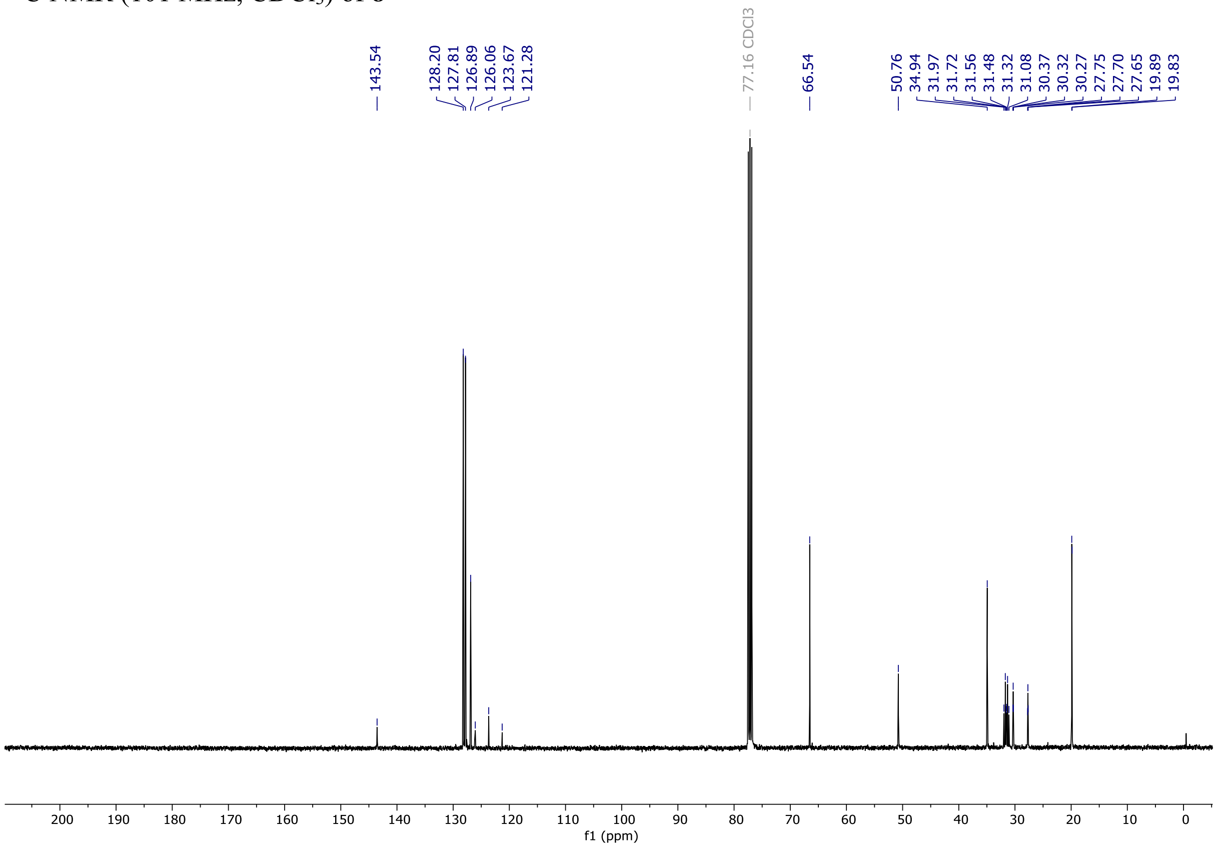

^{19}F NMR (282 MHz, $CDCl_3$) of **8**

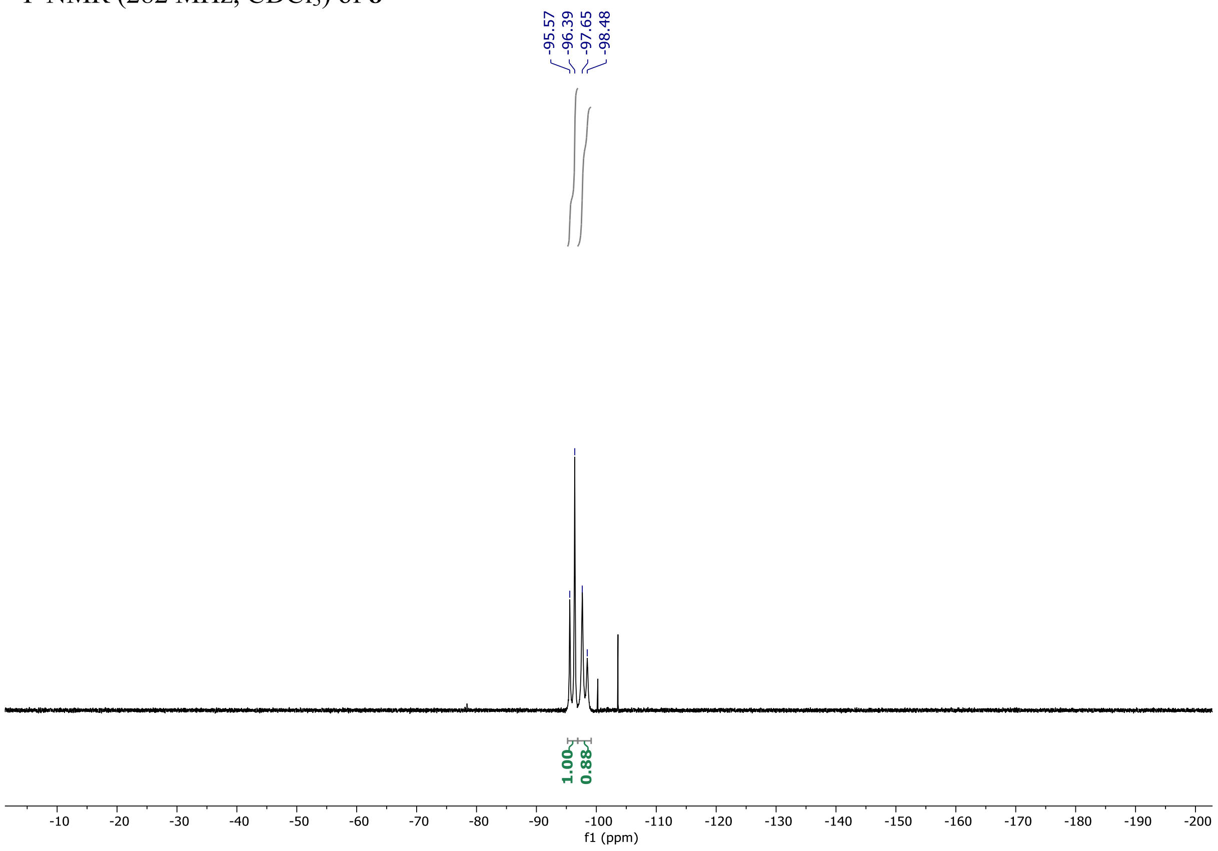

^{1}H NMR (400 MHz, $CDCl_3$) of **9**

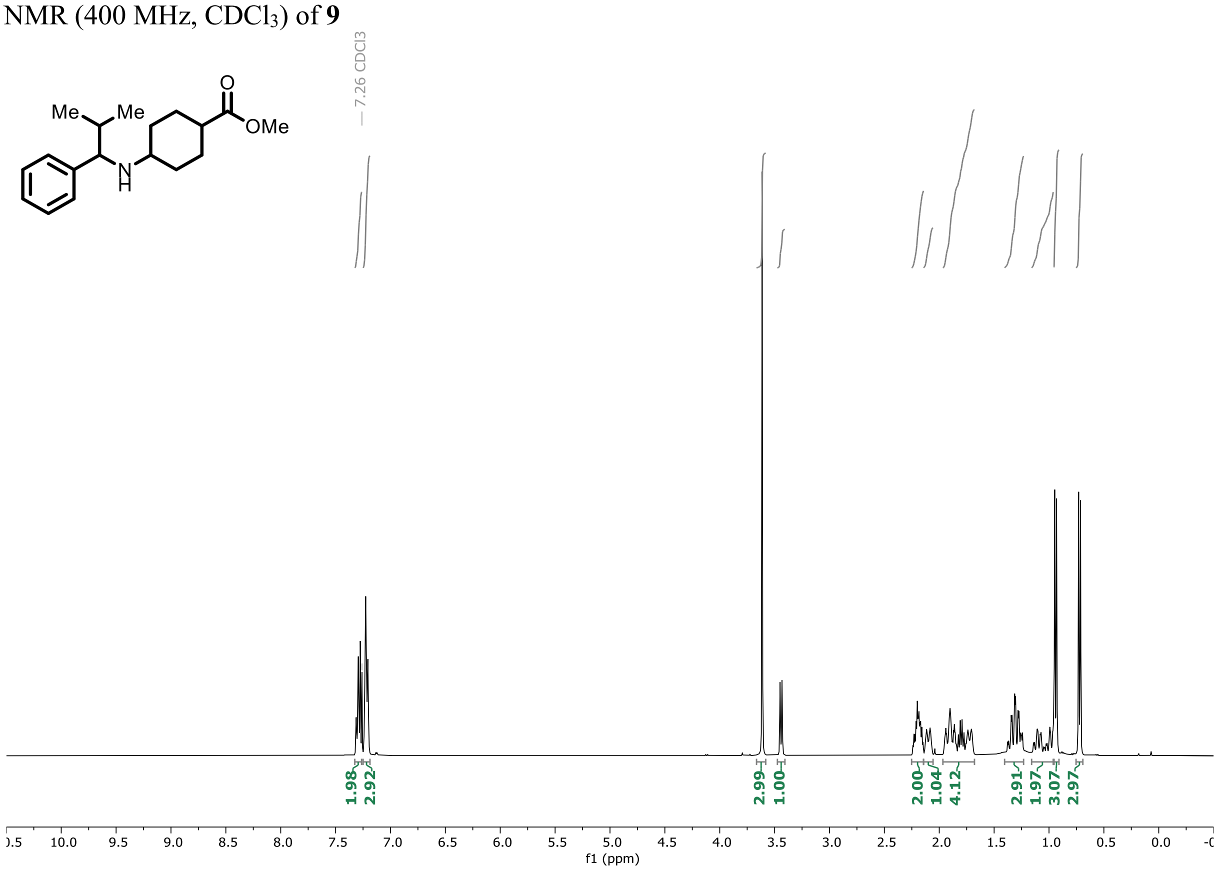

^{13}C NMR (101 MHz, $CDCl_3$) of **9**

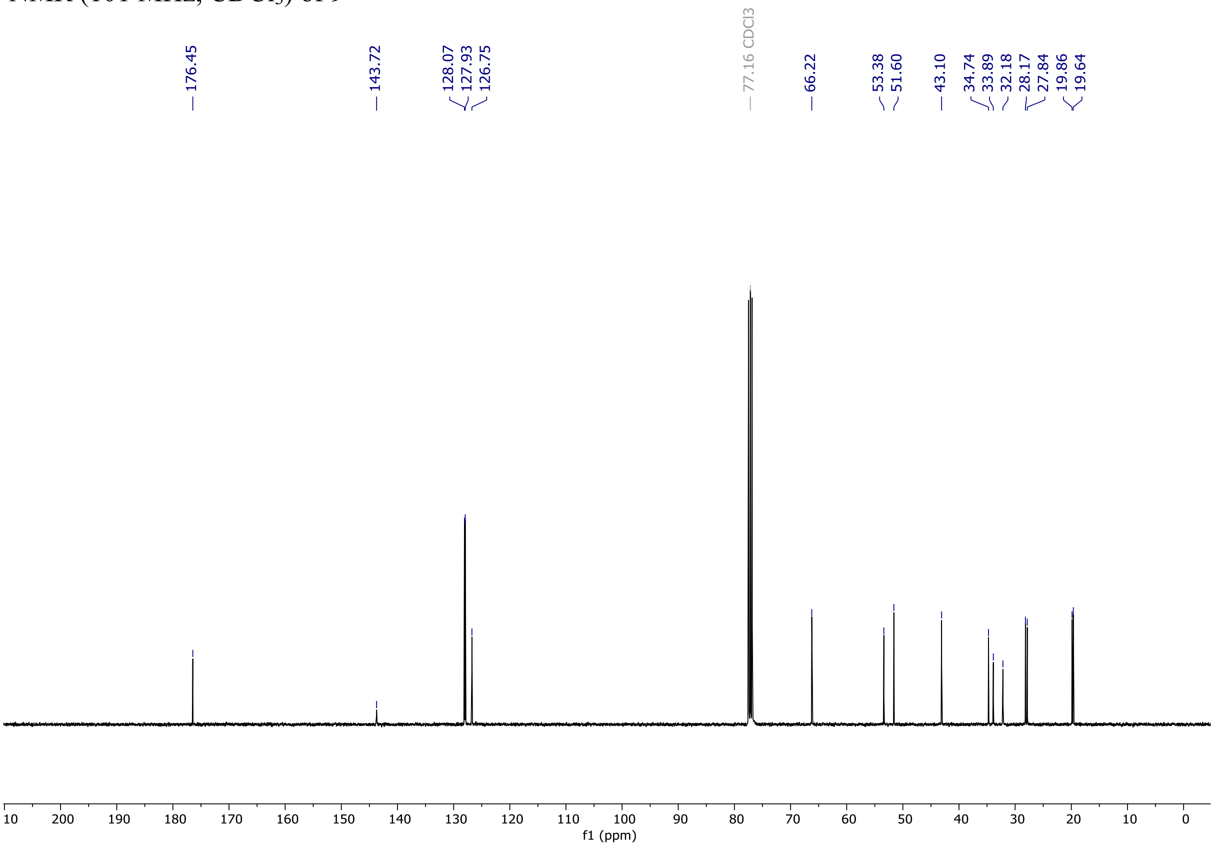

^{1}H NMR (400 MHz, $CDCl_3$) of **10**

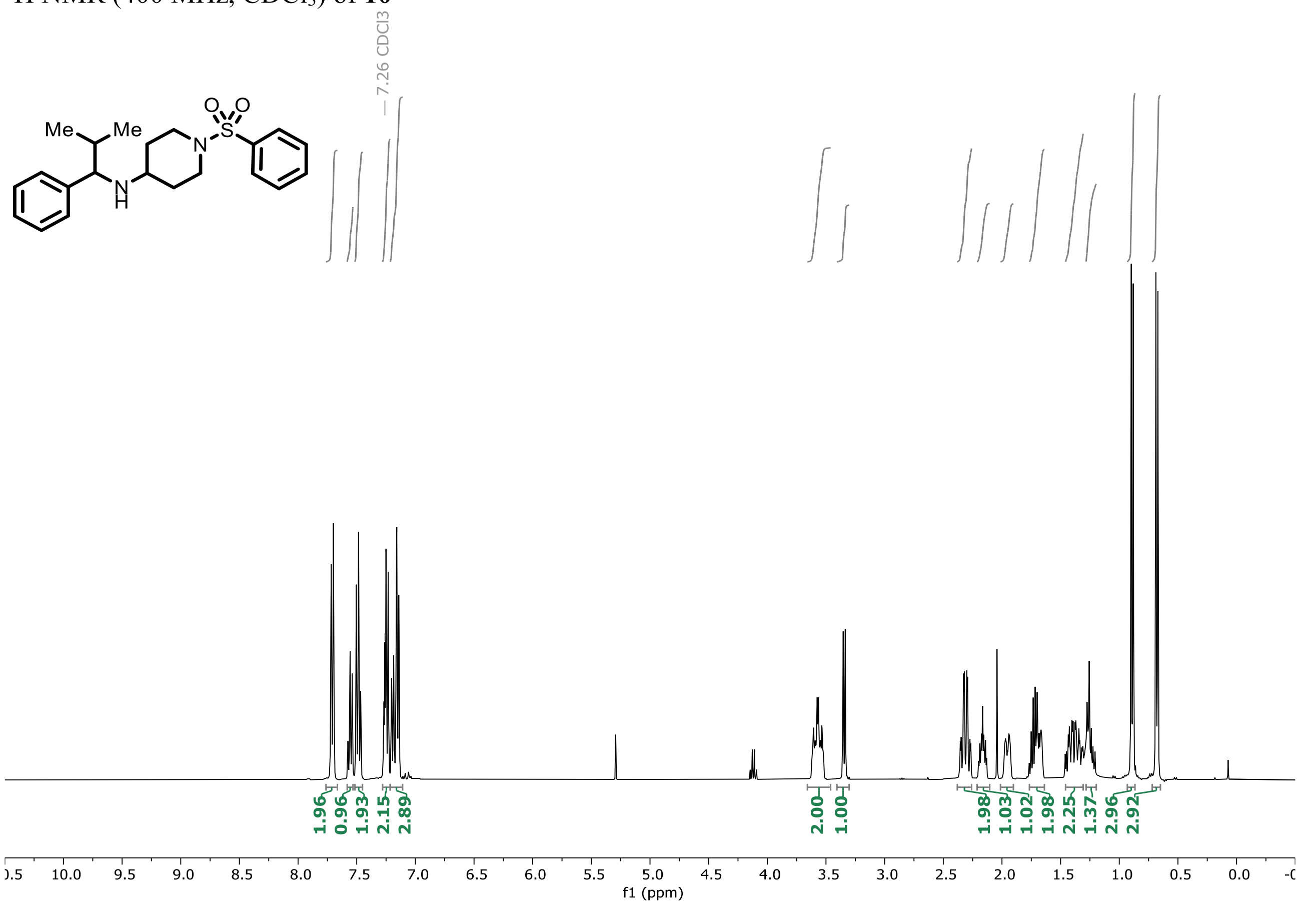

^{13}C NMR (101 MHz, $CDCl_3$) of **10**

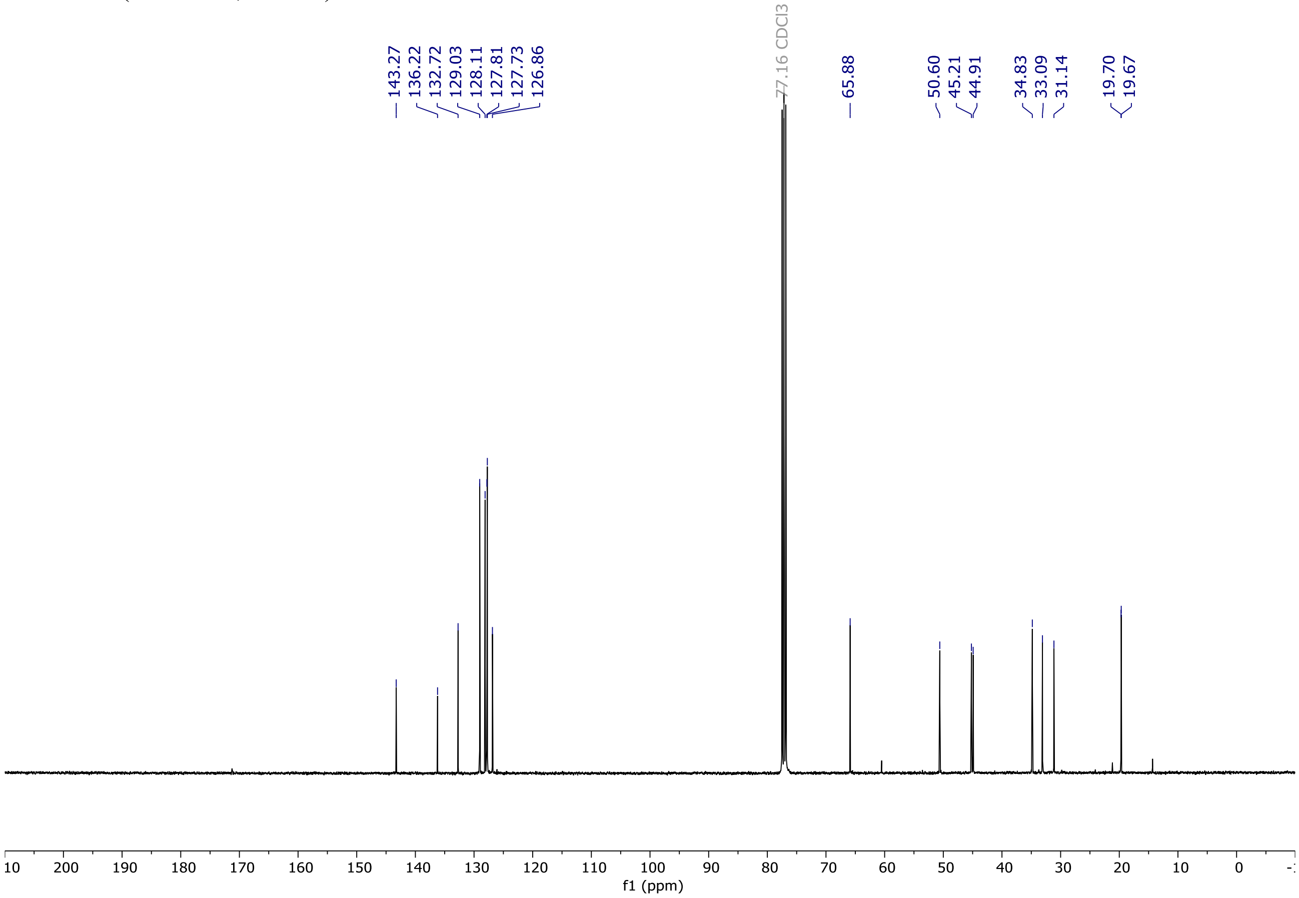

^{1}H NMR (400 MHz, $CDCl_3$) of **11**

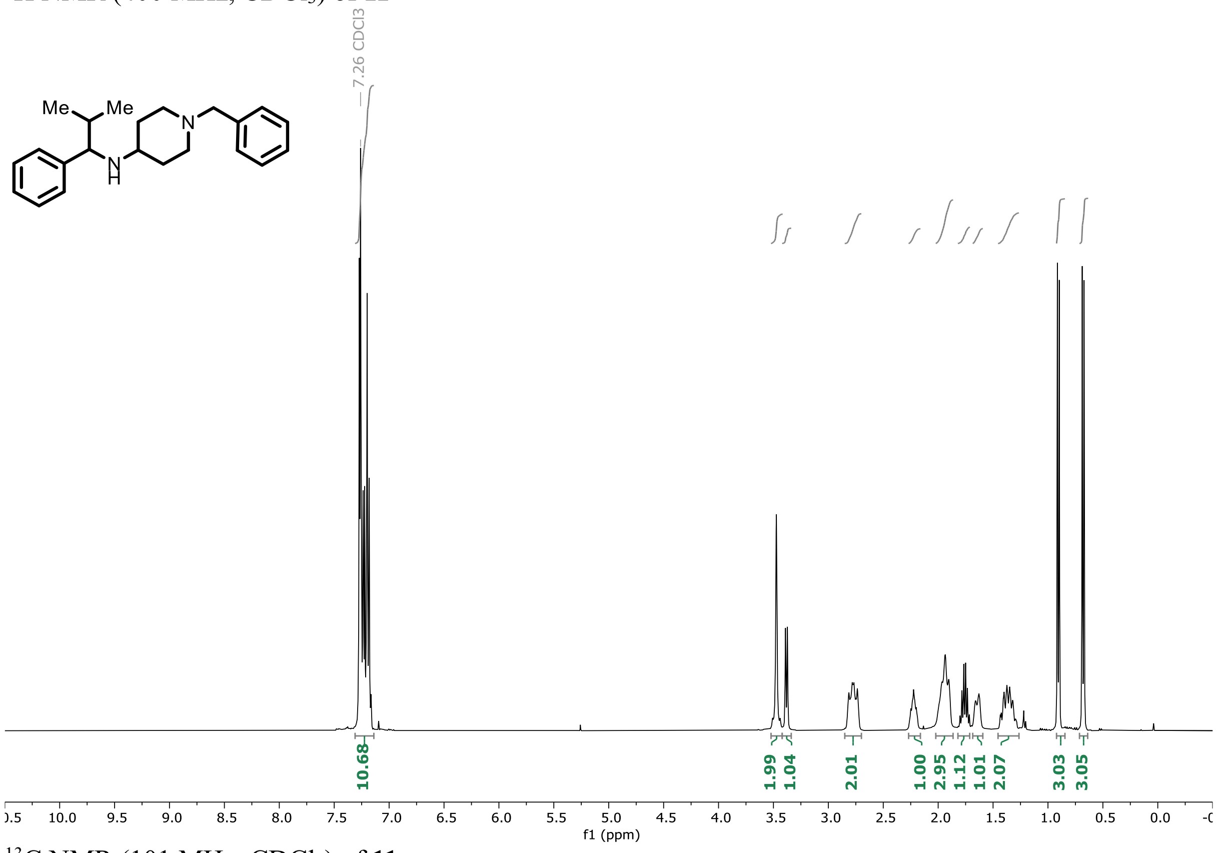

^{13}C NMR (101 MHz, $CDCl_3$) of **11**

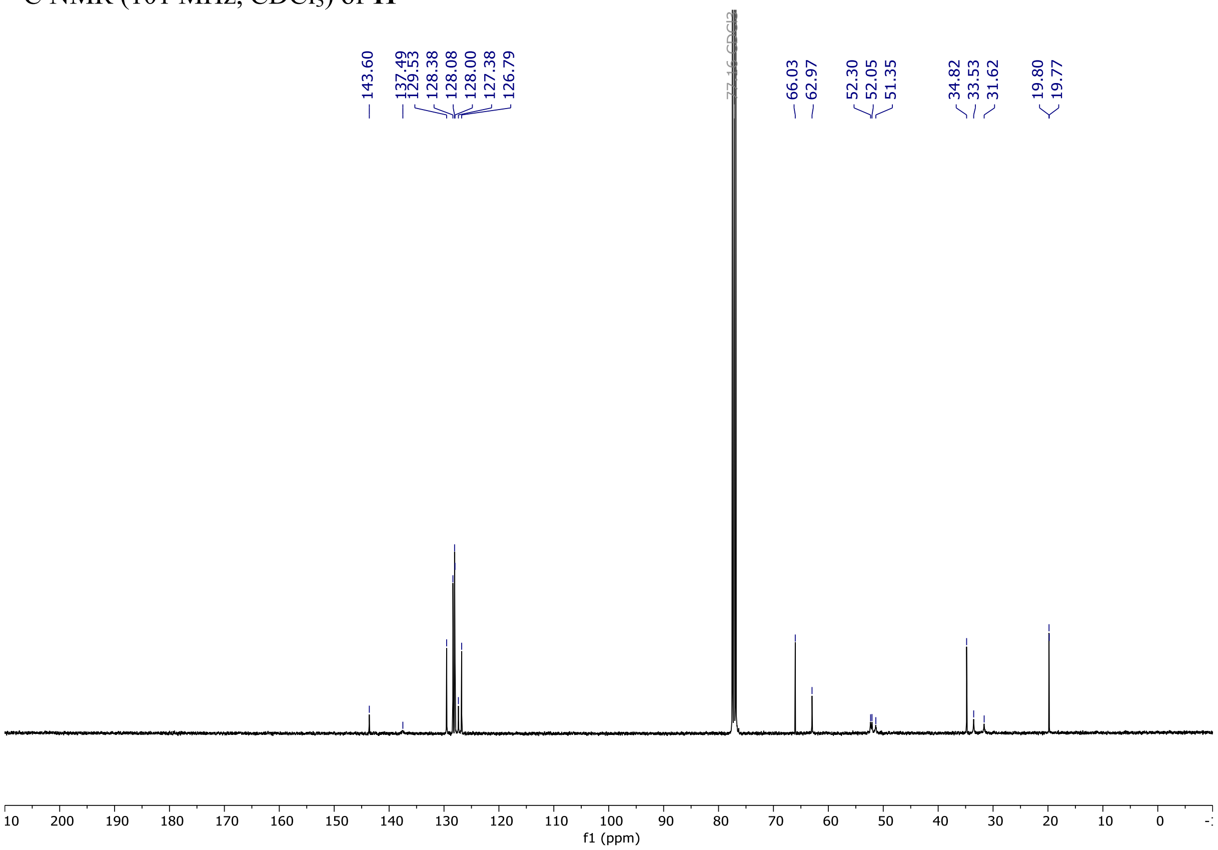

^{1}H NMR (400 MHz, $CDCl_3$) of **12**

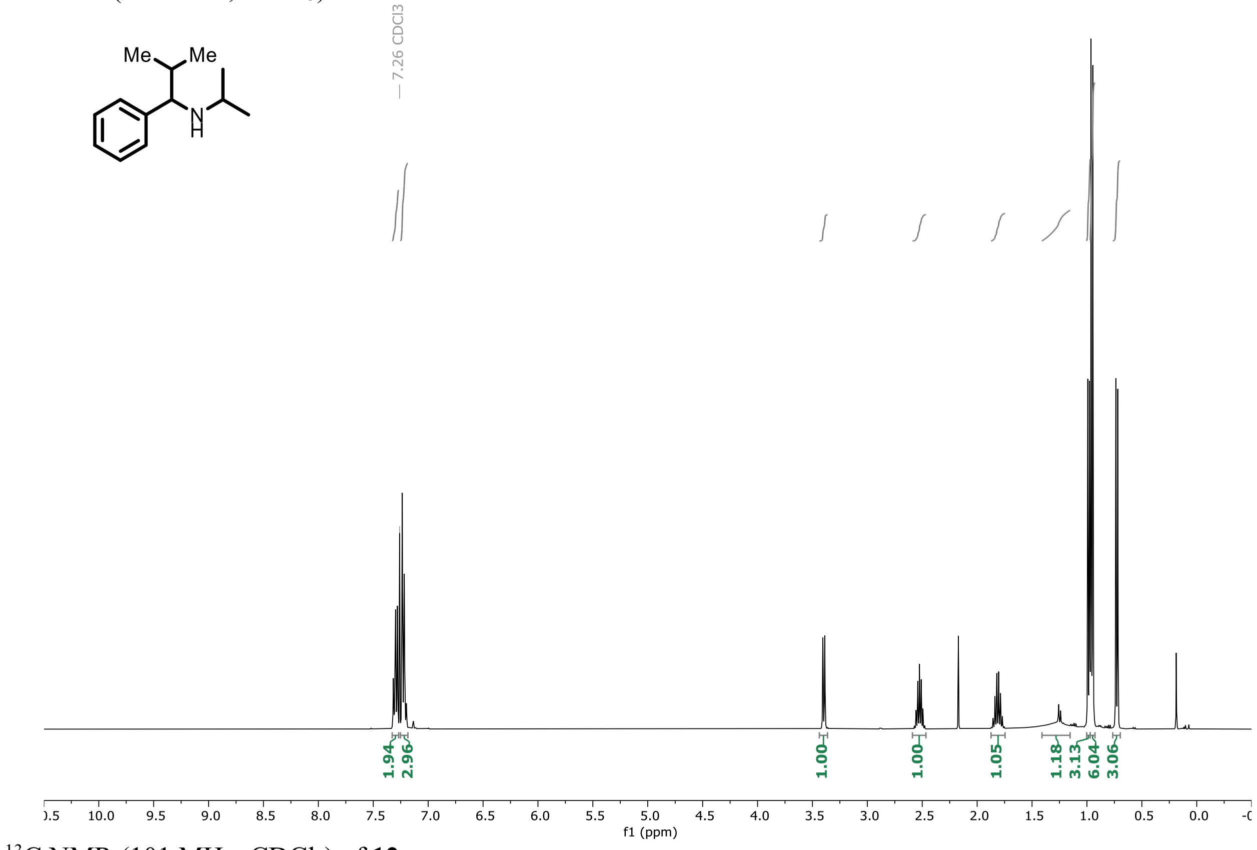

^{13}C NMR (101 MHz, $CDCl_3$) of **12**

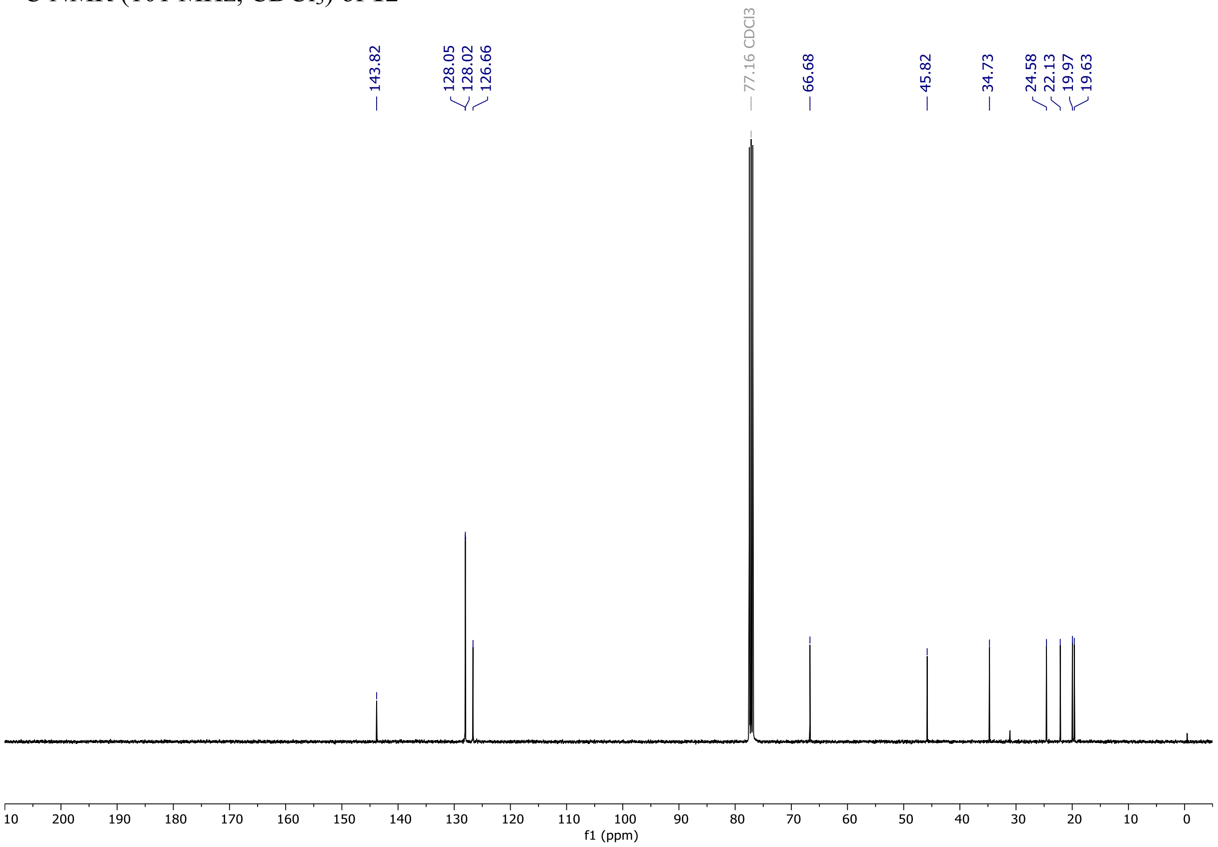

^{1}H NMR (400 MHz, $CDCl_3$) of **13**

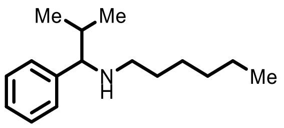

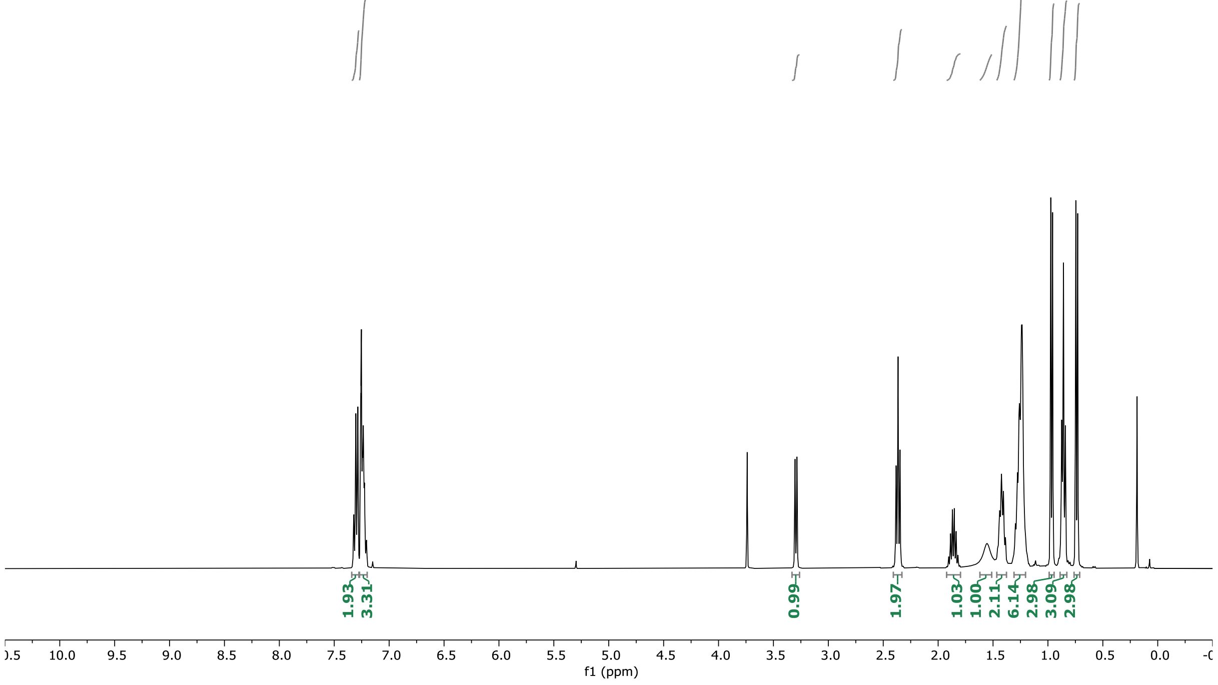

^{13}C NMR (101 MHz, $CDCl_3$) of **13**

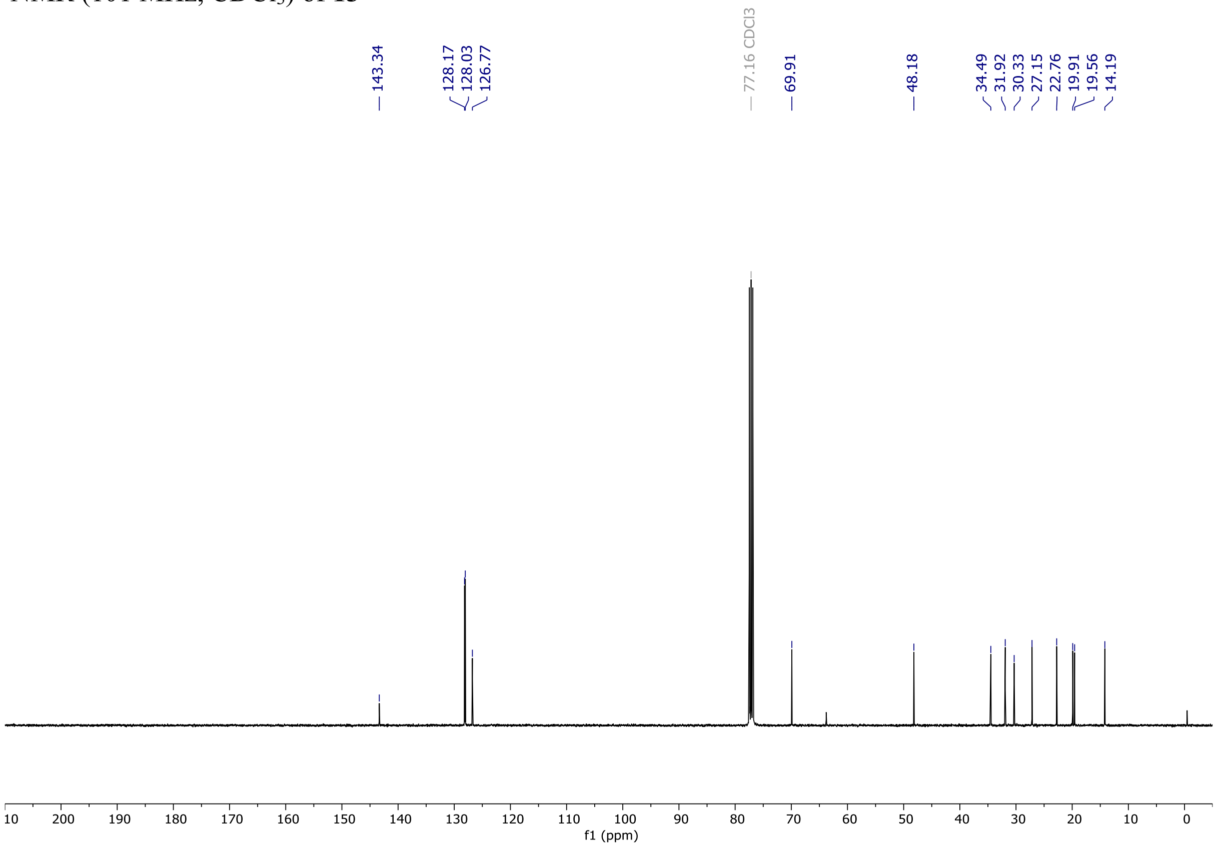

^{1}H NMR (400 MHz, $CDCl_3$) of **14**

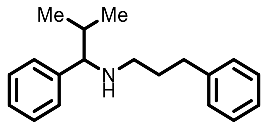

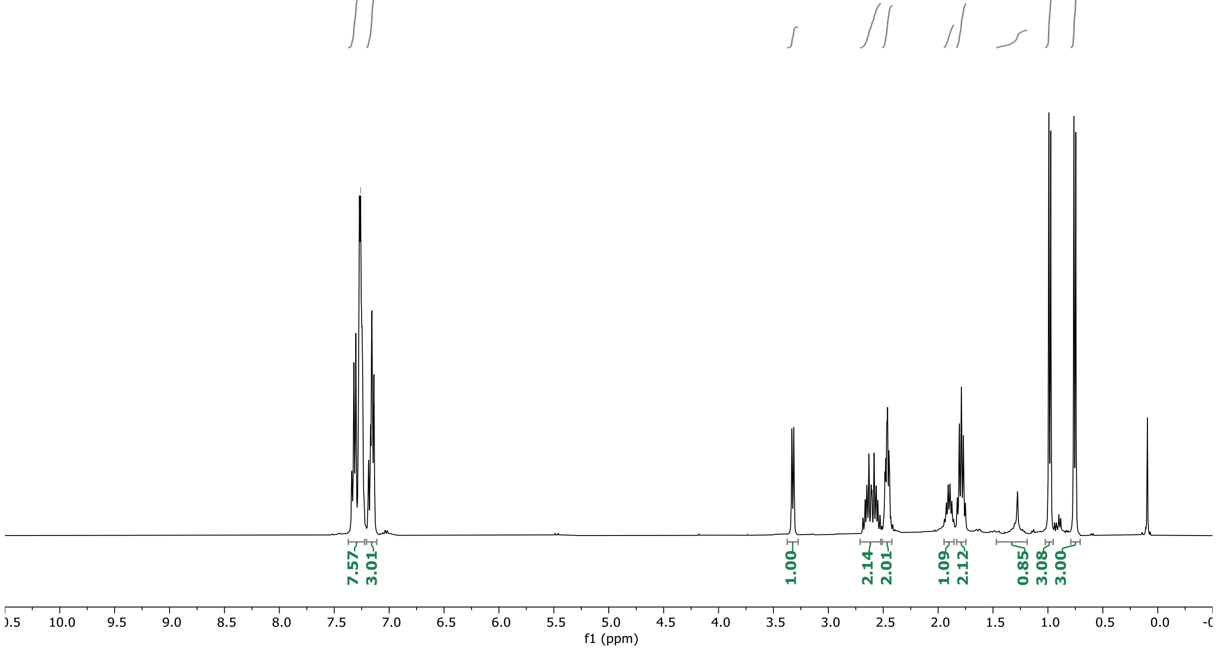

^{13}C NMR (101 MHz, $CDCl_3$) of **14**

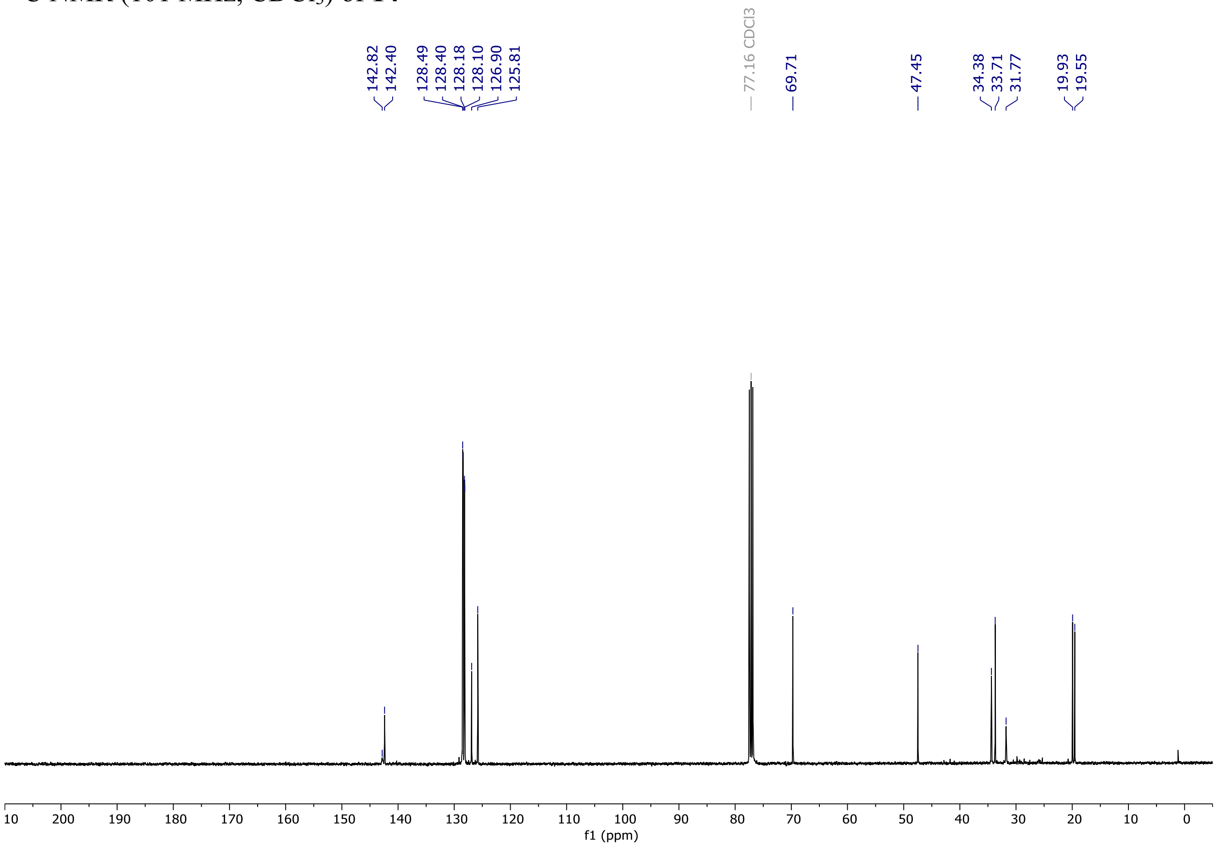

^{1}H NMR (400 MHz, $CDCl_3$) of **15**

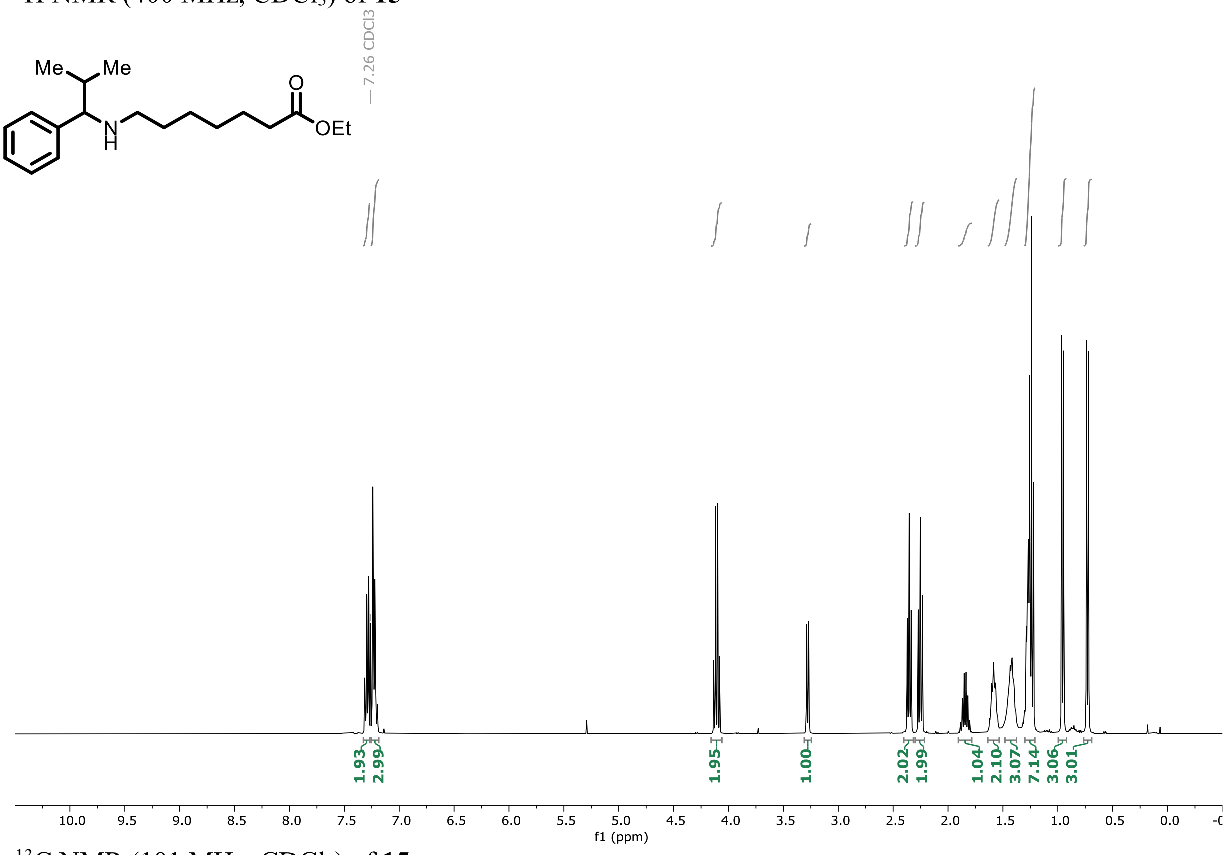

^{13}C NMR (101 MHz, $CDCl_3$) of **15**

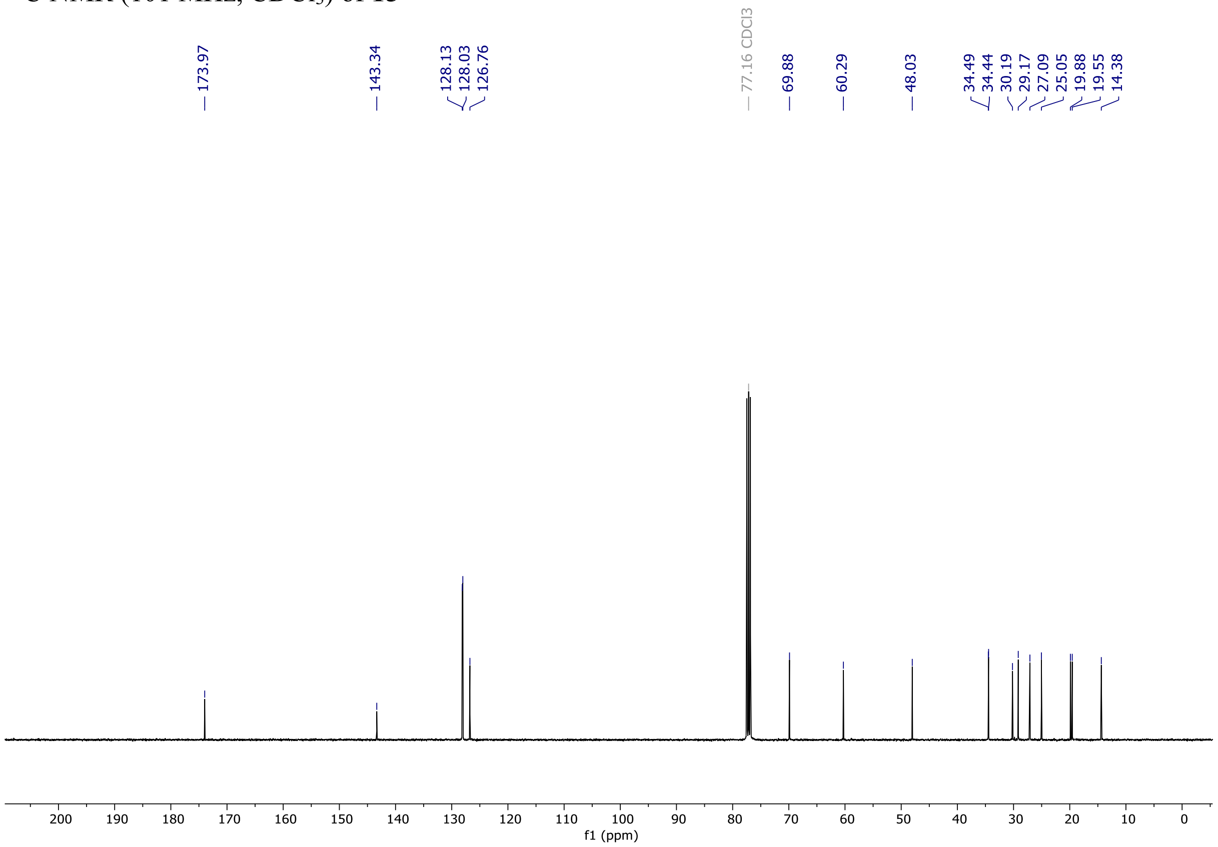

^{1}H NMR (400 MHz, $CDCl_3$) of **16**

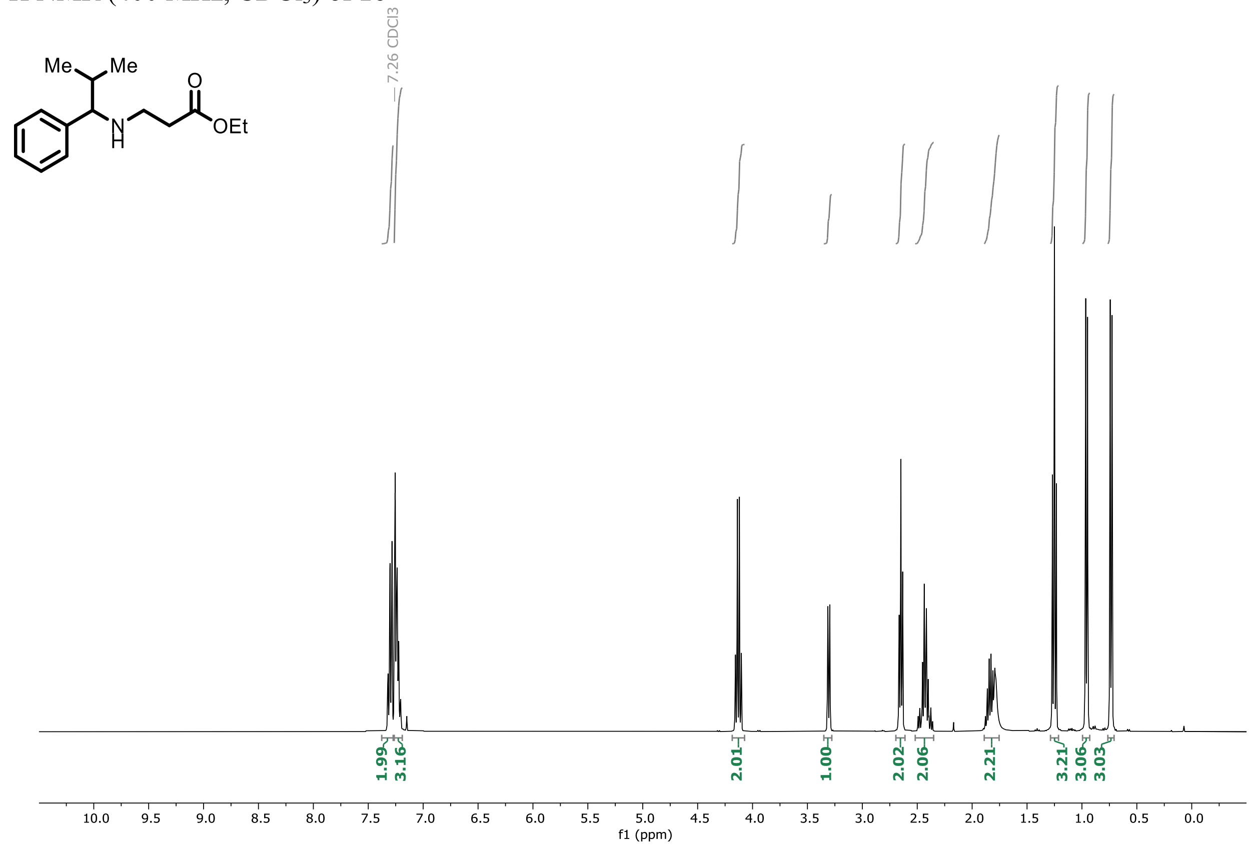

^{13}C NMR (101 MHz, $CDCl_3$) of **16**

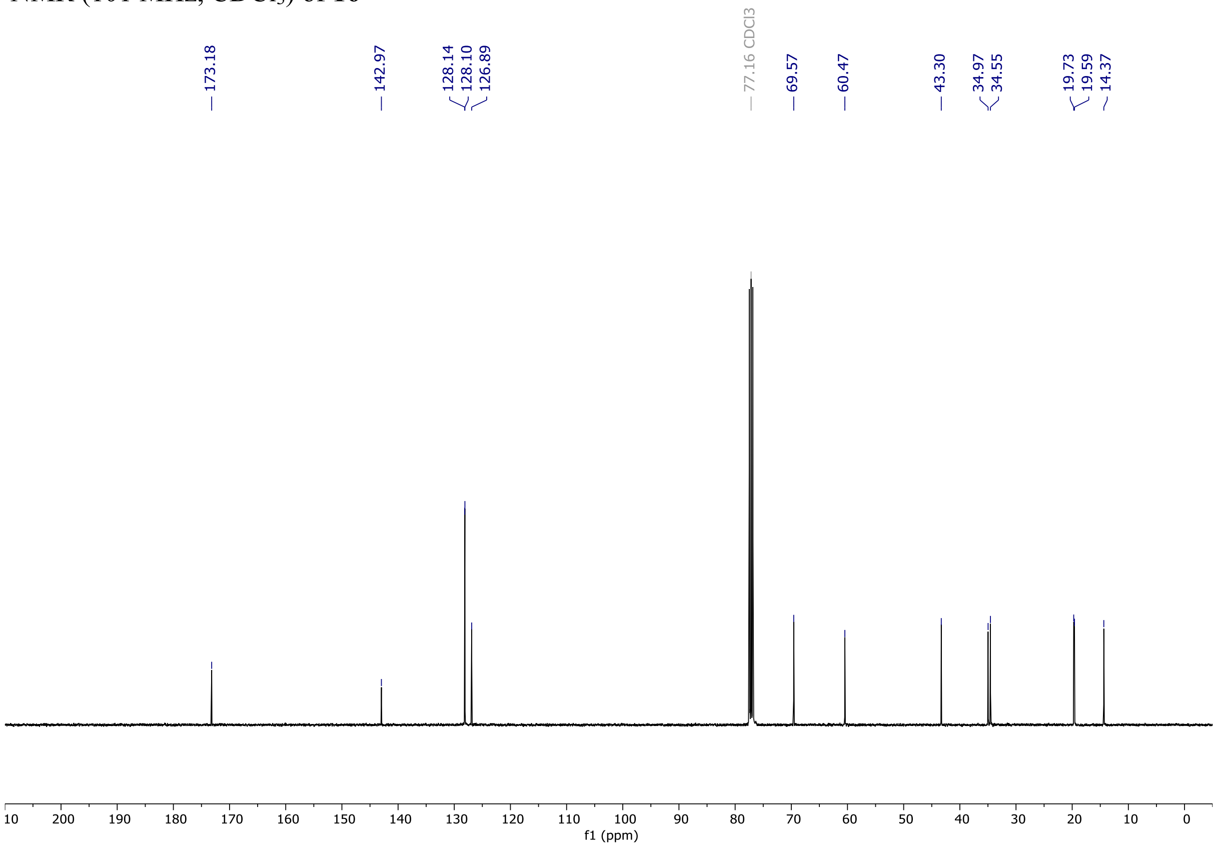

^{1}H NMR (400 MHz, $CDCl_3$) of **17**

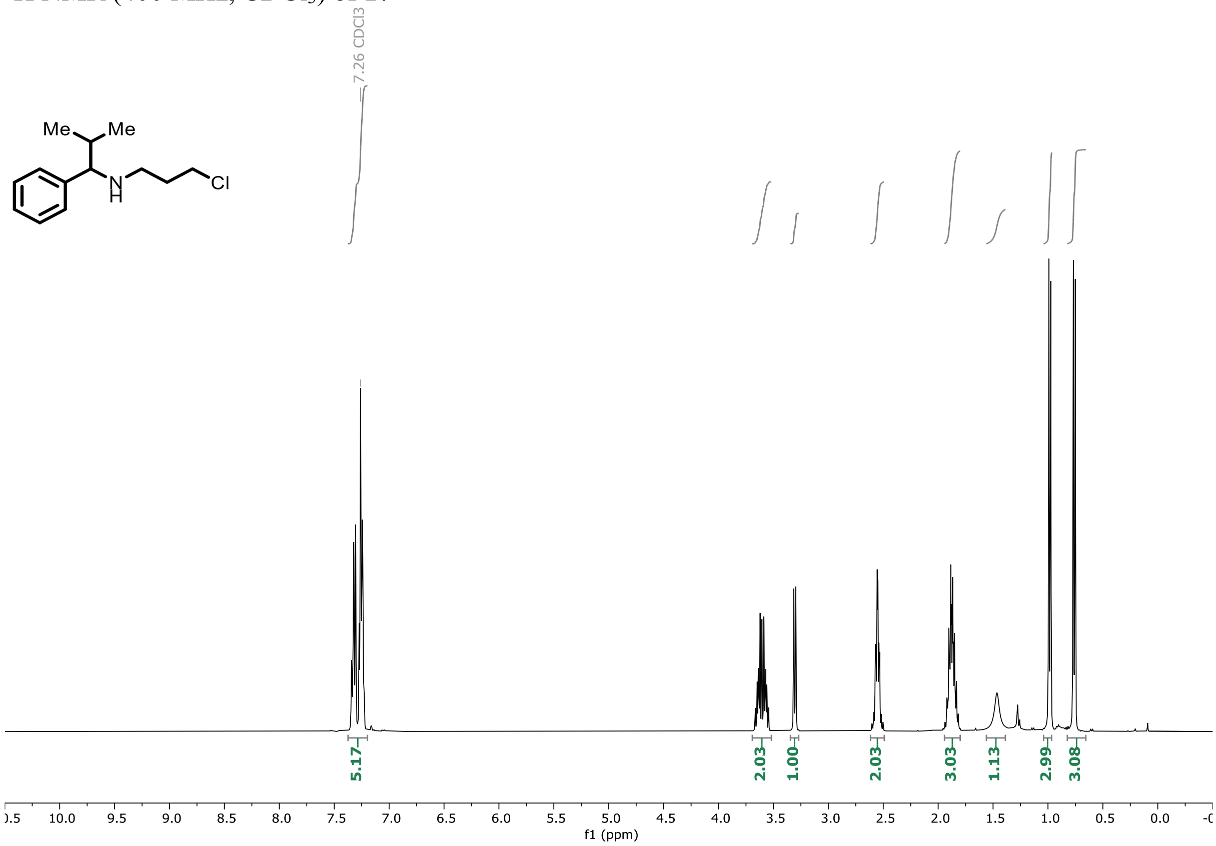

^{13}C NMR (101 MHz, $CDCl_3$) of **17**

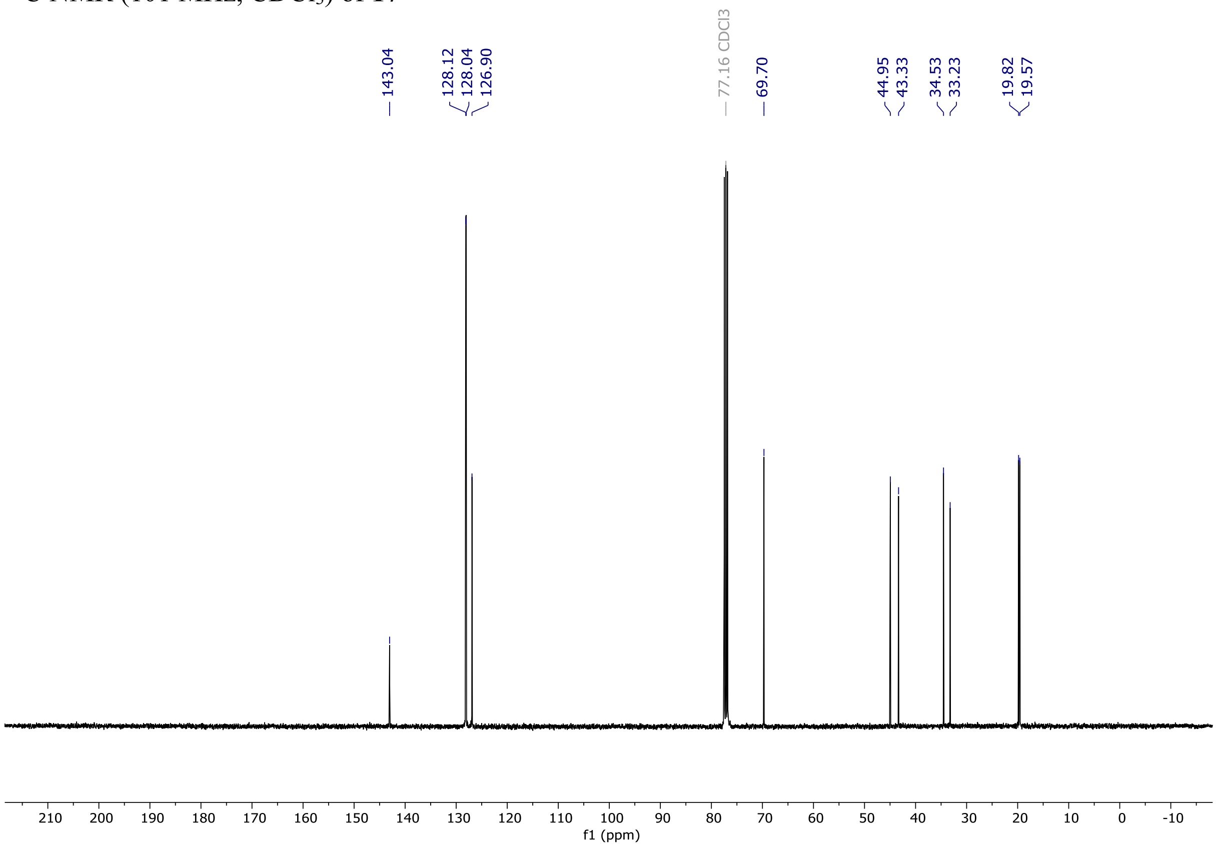

^1H NMR (400 MHz, $CDCl_3$) of **18**

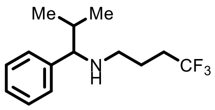

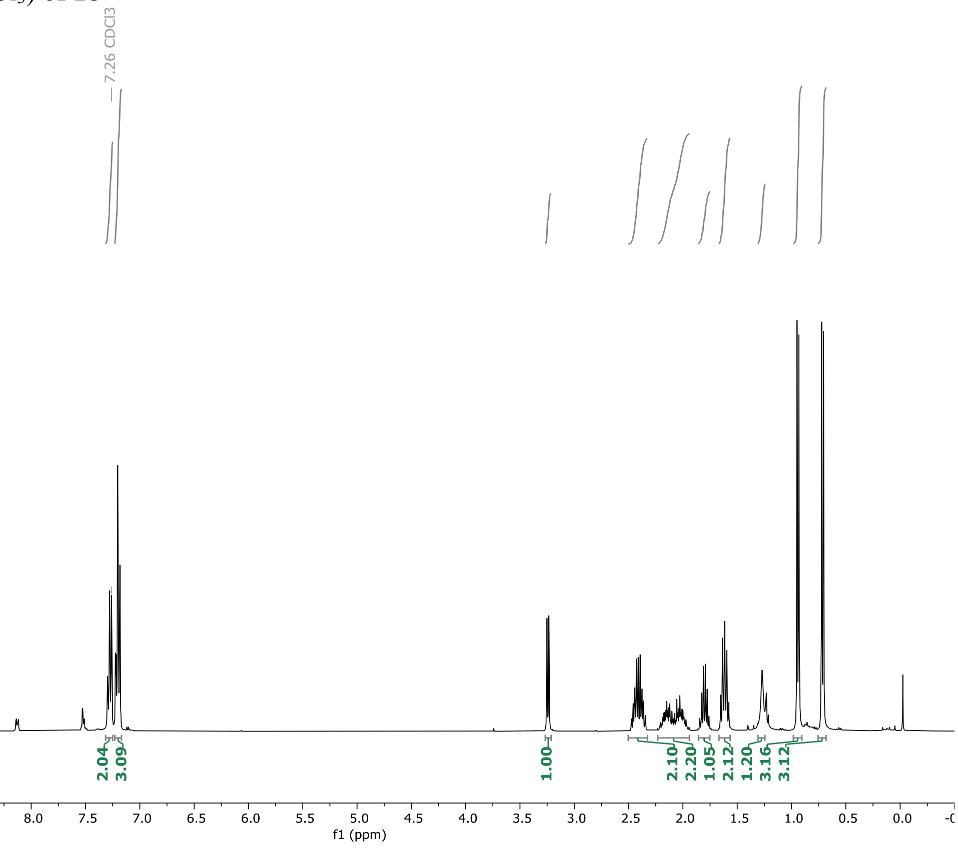

^{13}C NMR (101 MHz, $CDCl_3$) of **18**

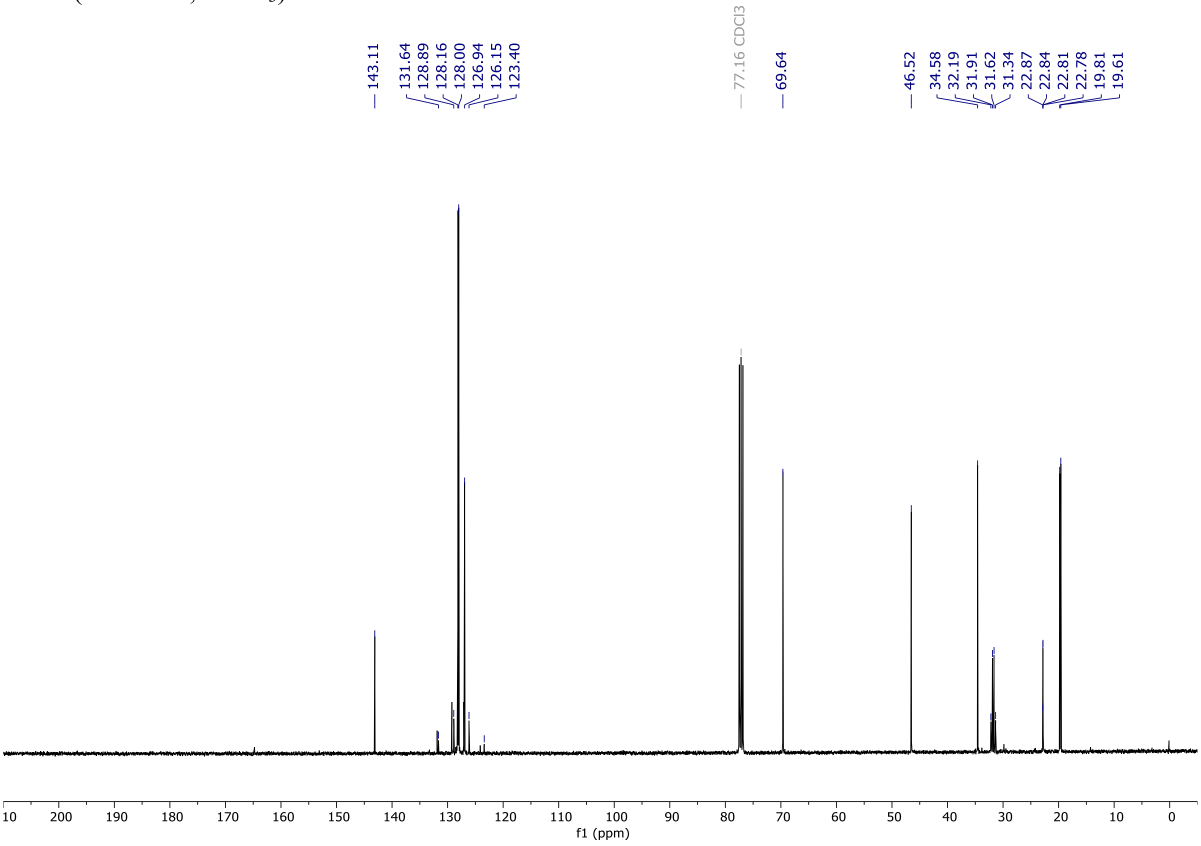

^{19}F NMR (376 MHz, $CDCl_3$) of **18**

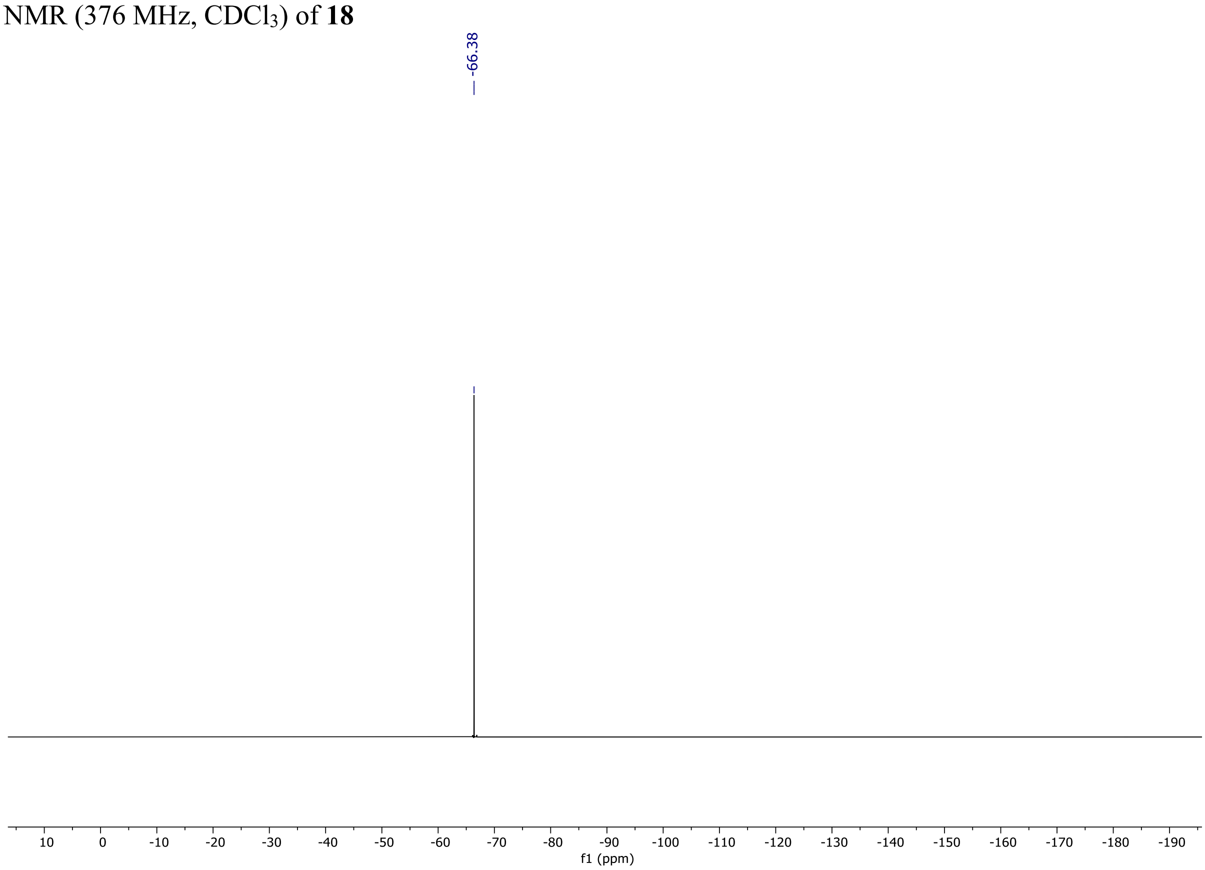

^{1}H NMR (400 MHz, $CDCl_3$) of **19**

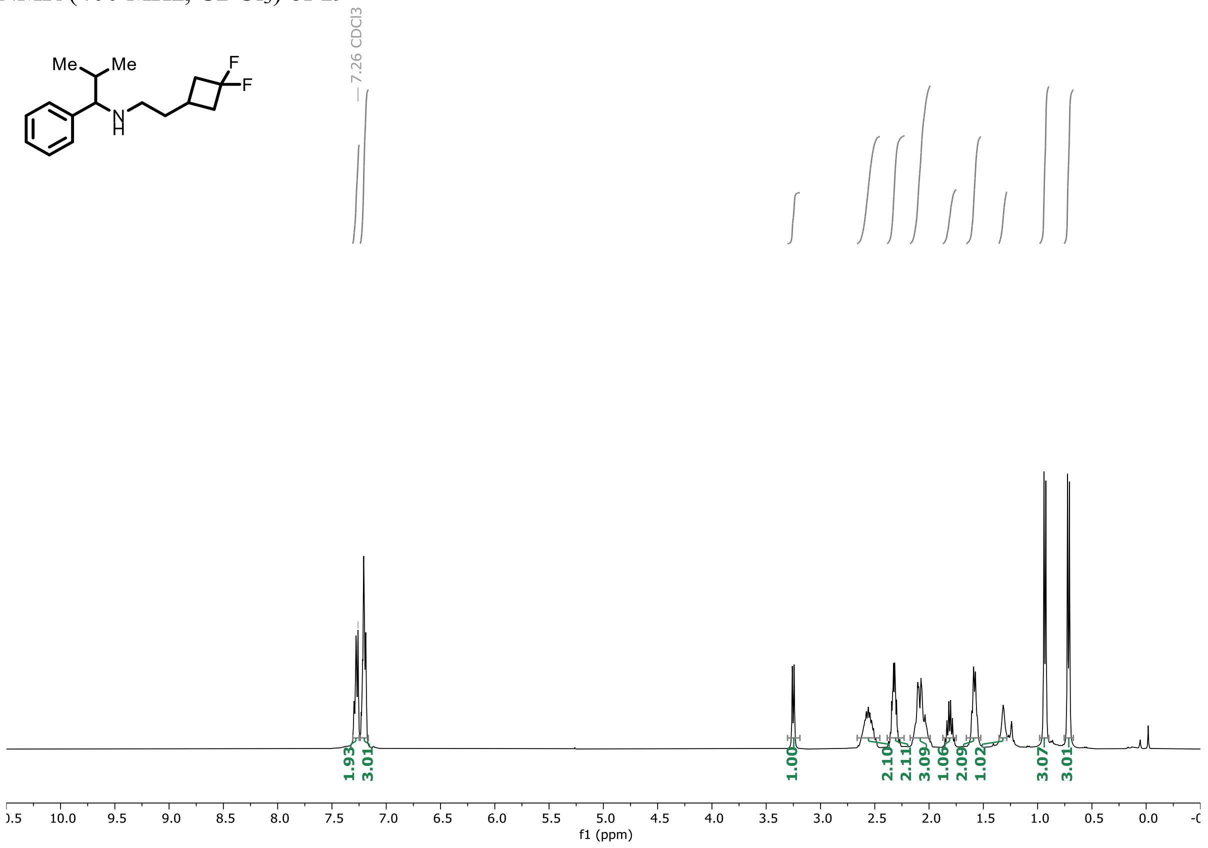

^{13}C NMR (101 MHz, $CDCl_3$) of **19**

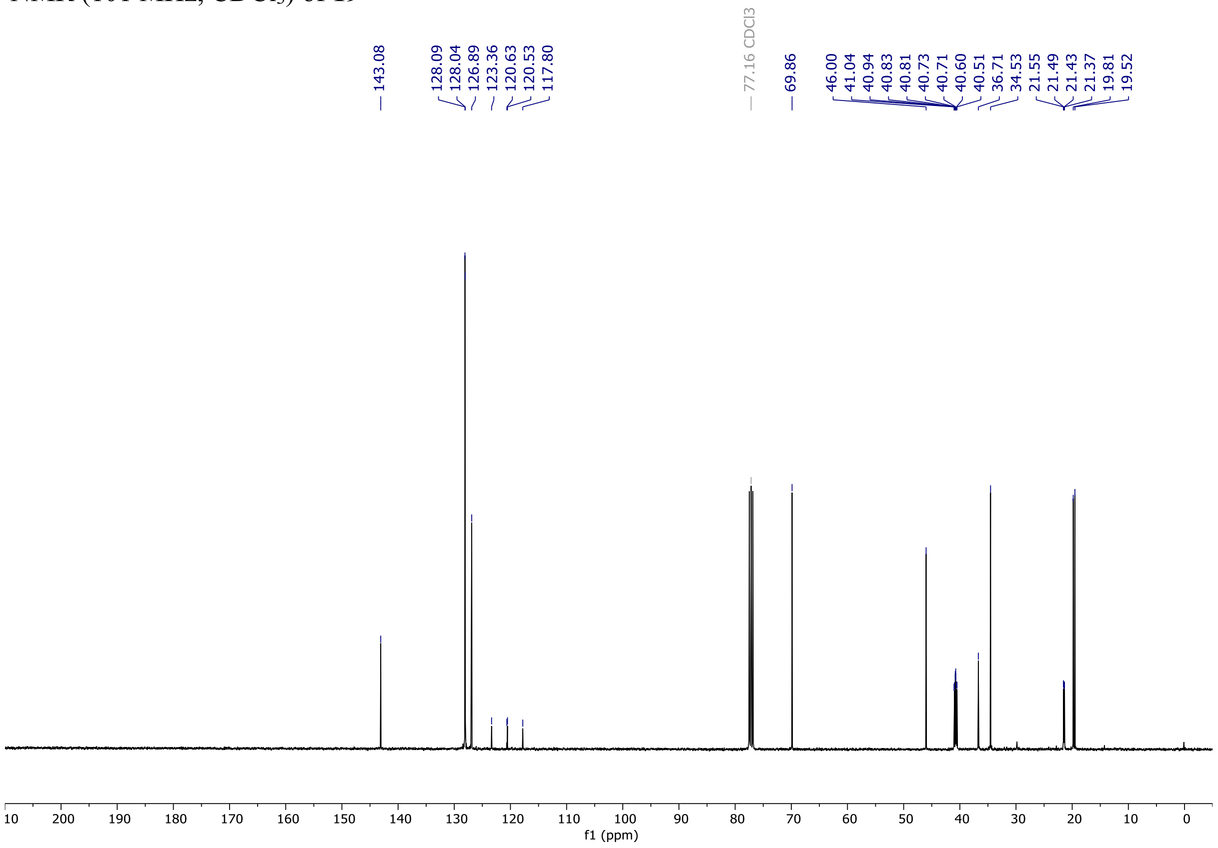

^{19}F NMR (377 MHz, $CDCl_3$) of **19**

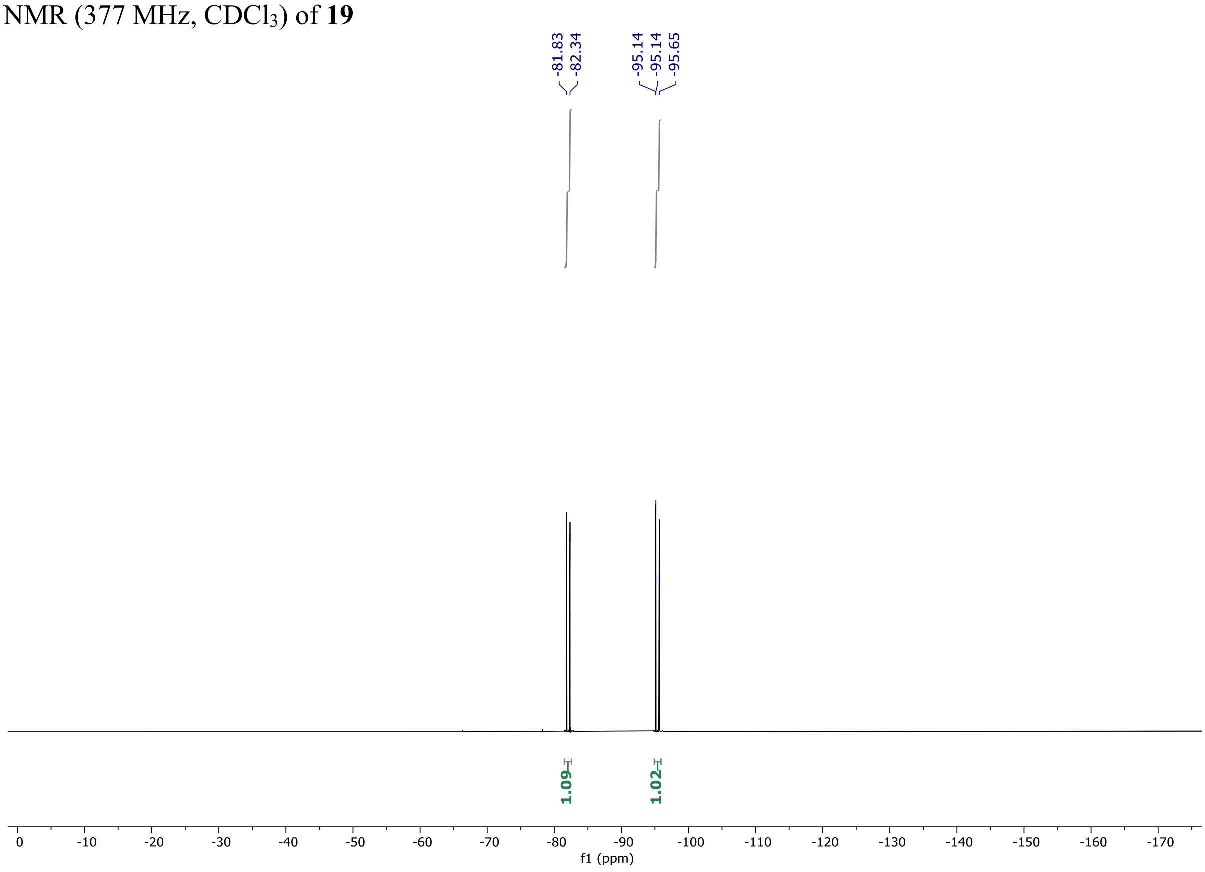

^{1}H NMR (400 MHz, $CDCl_3$) of **20**

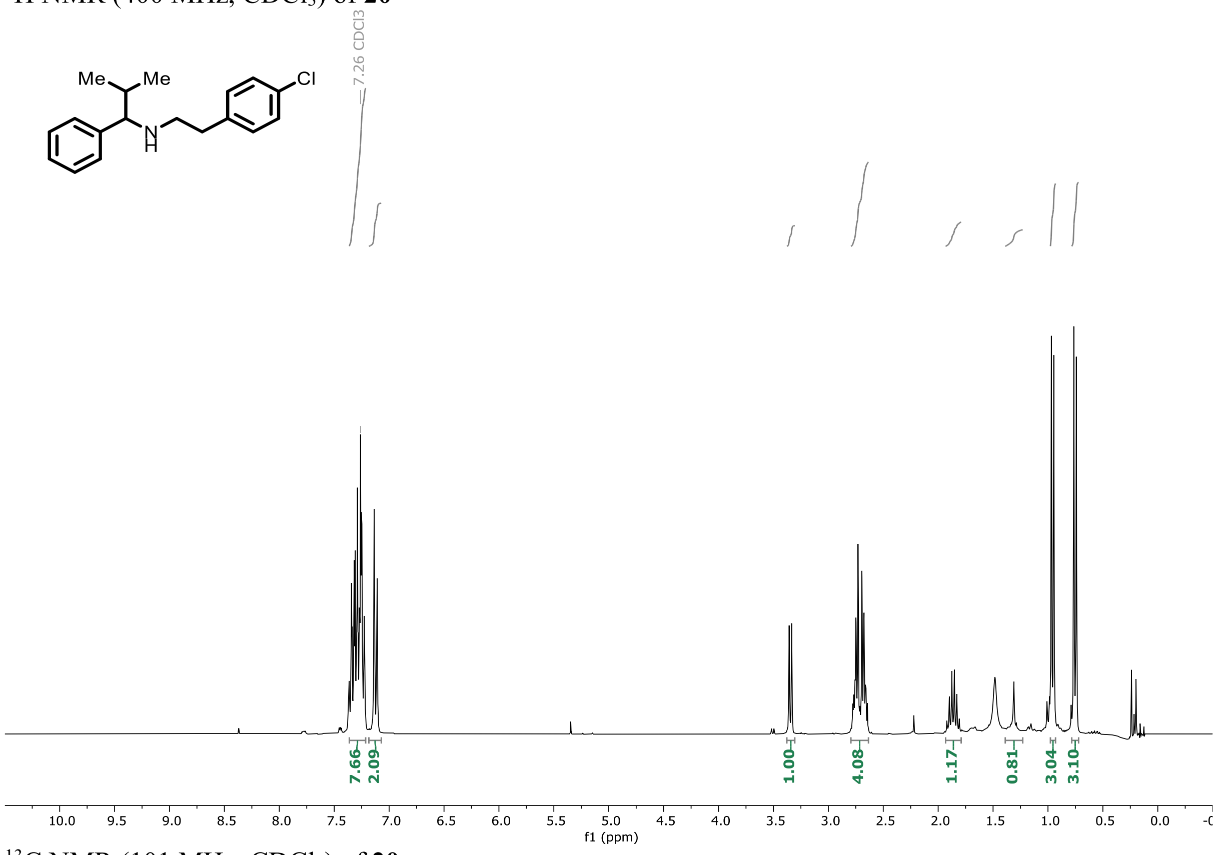

^{13}C NMR (101 MHz, $CDCl_3$) of **20**

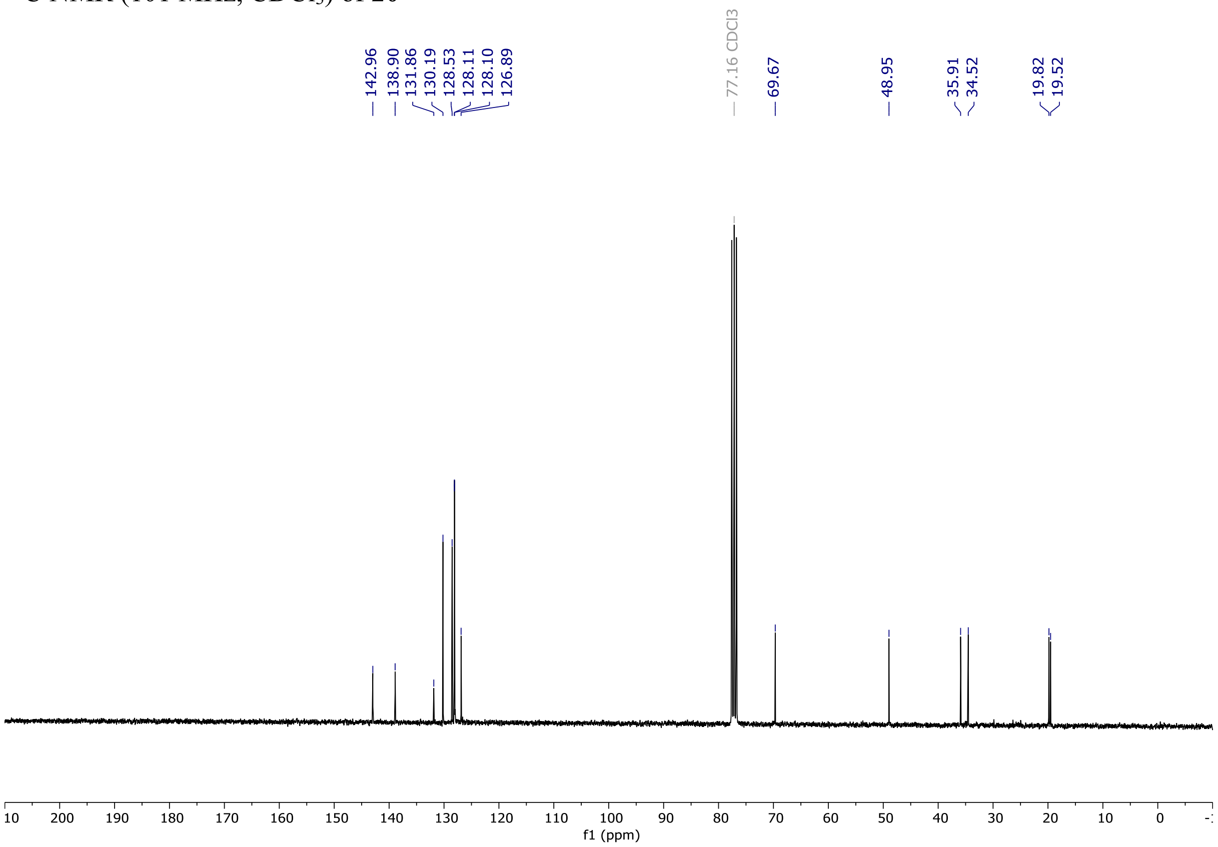

^{1}H NMR (400 MHz, $CDCl_3$) of **21**

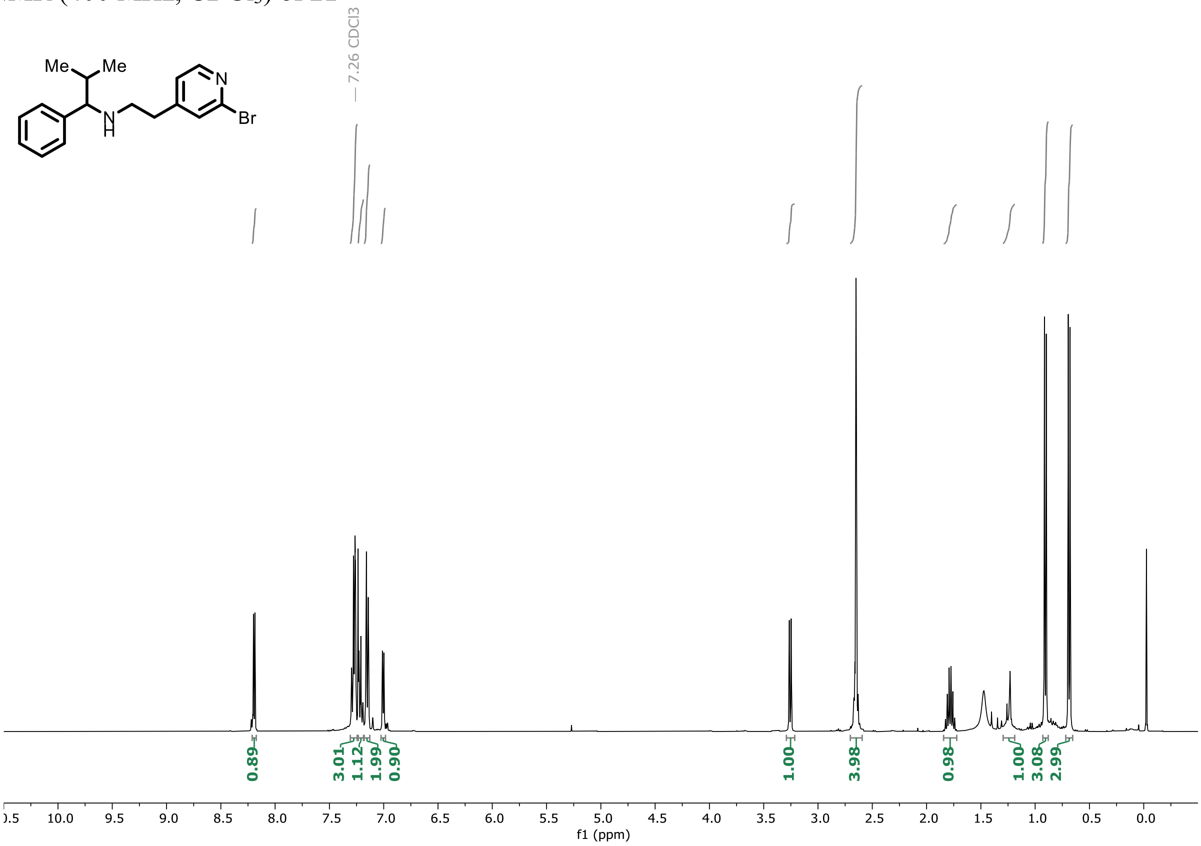

^{13}C NMR (101 MHz, $CDCl_3$) of **21**

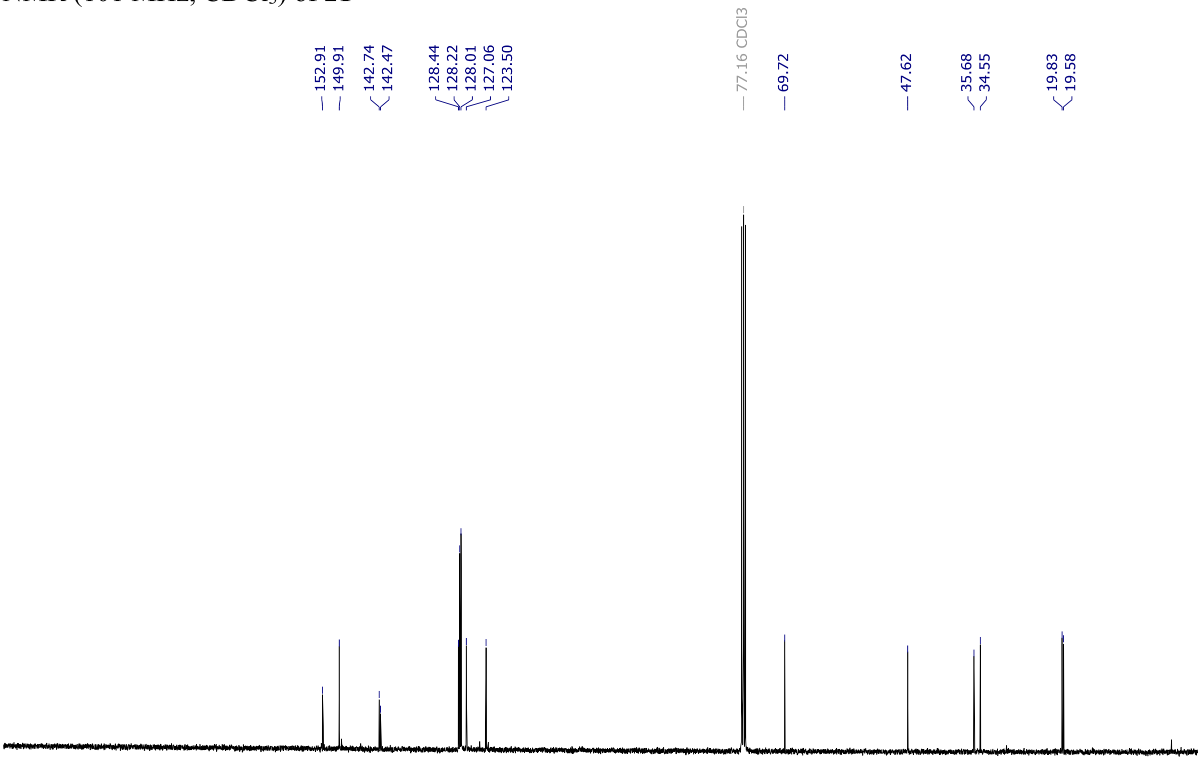

^{1}H NMR (400 MHz, $CDCl_3$) of **23**

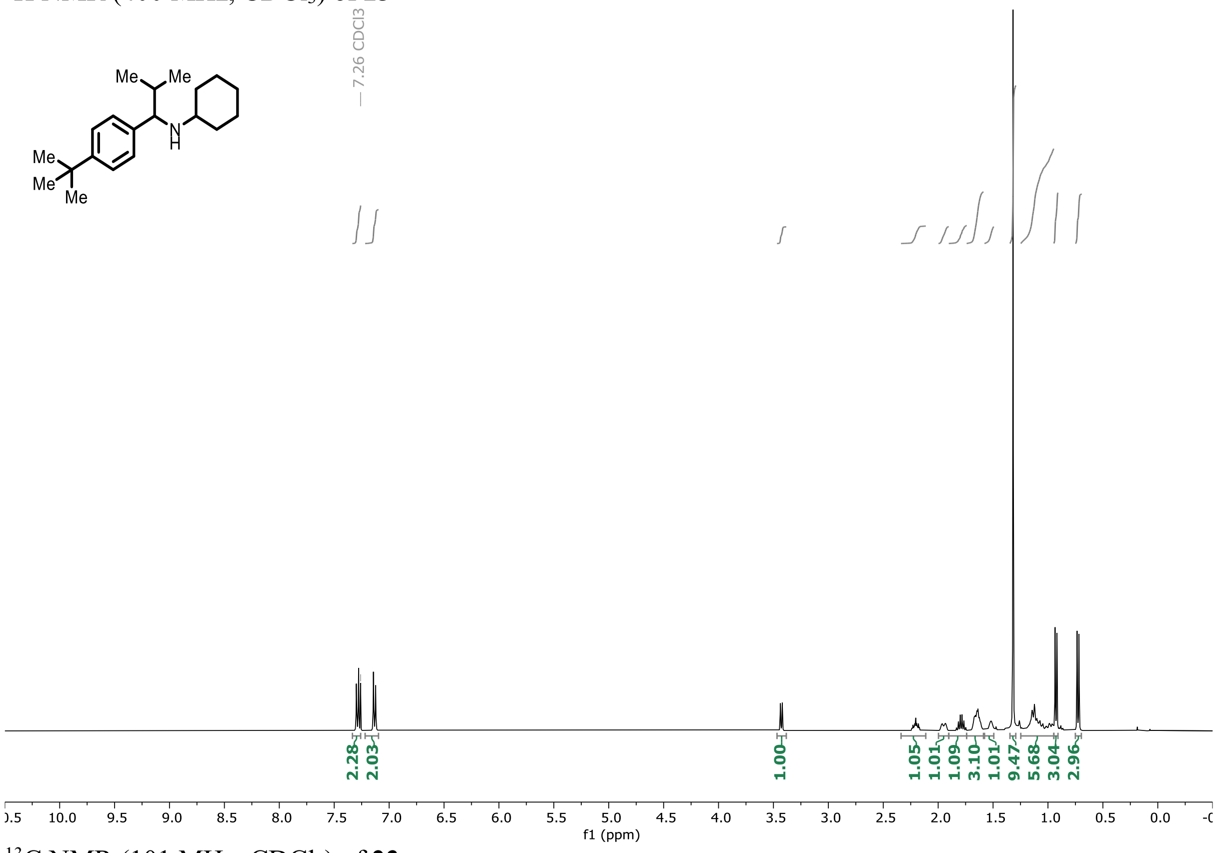

^{13}C NMR (101 MHz, $CDCl_3$) of **23**

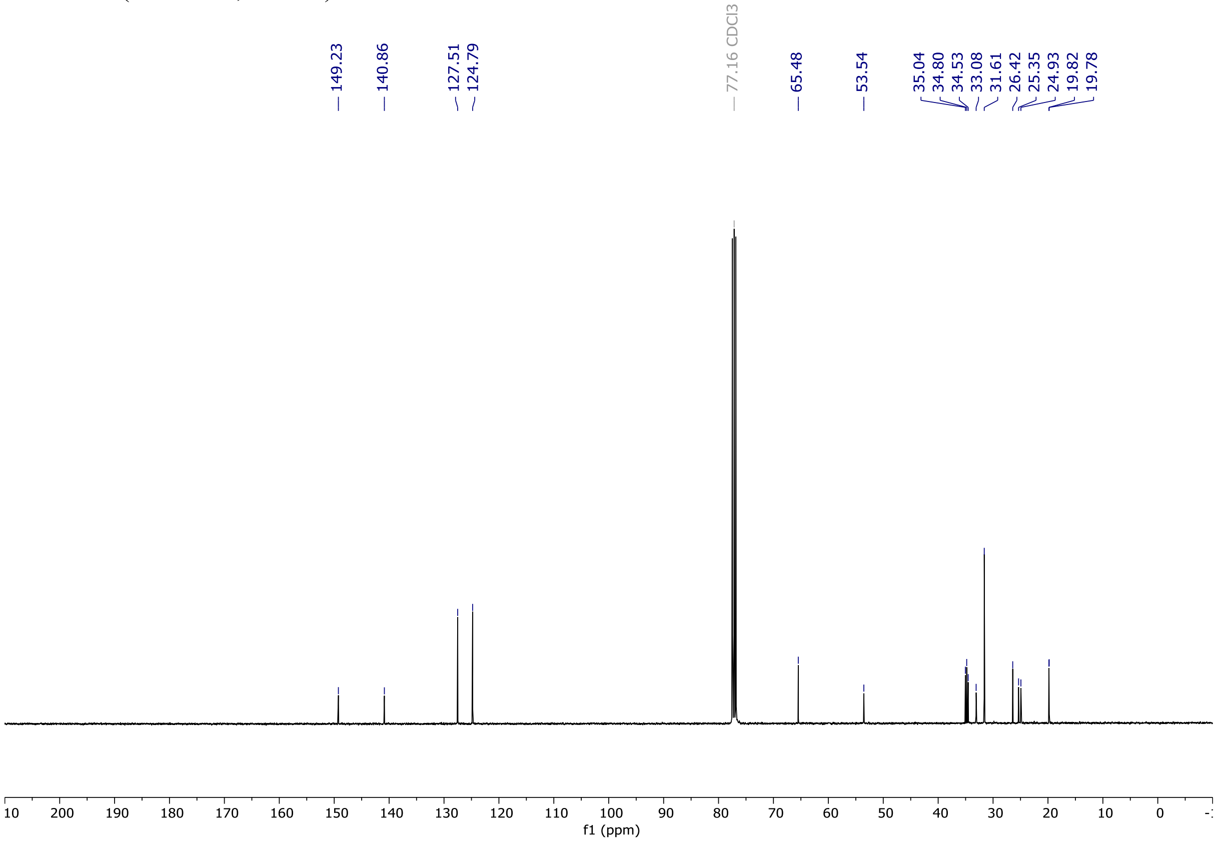

^{1}H NMR (400 MHz, $CDCl_3$) of **24**

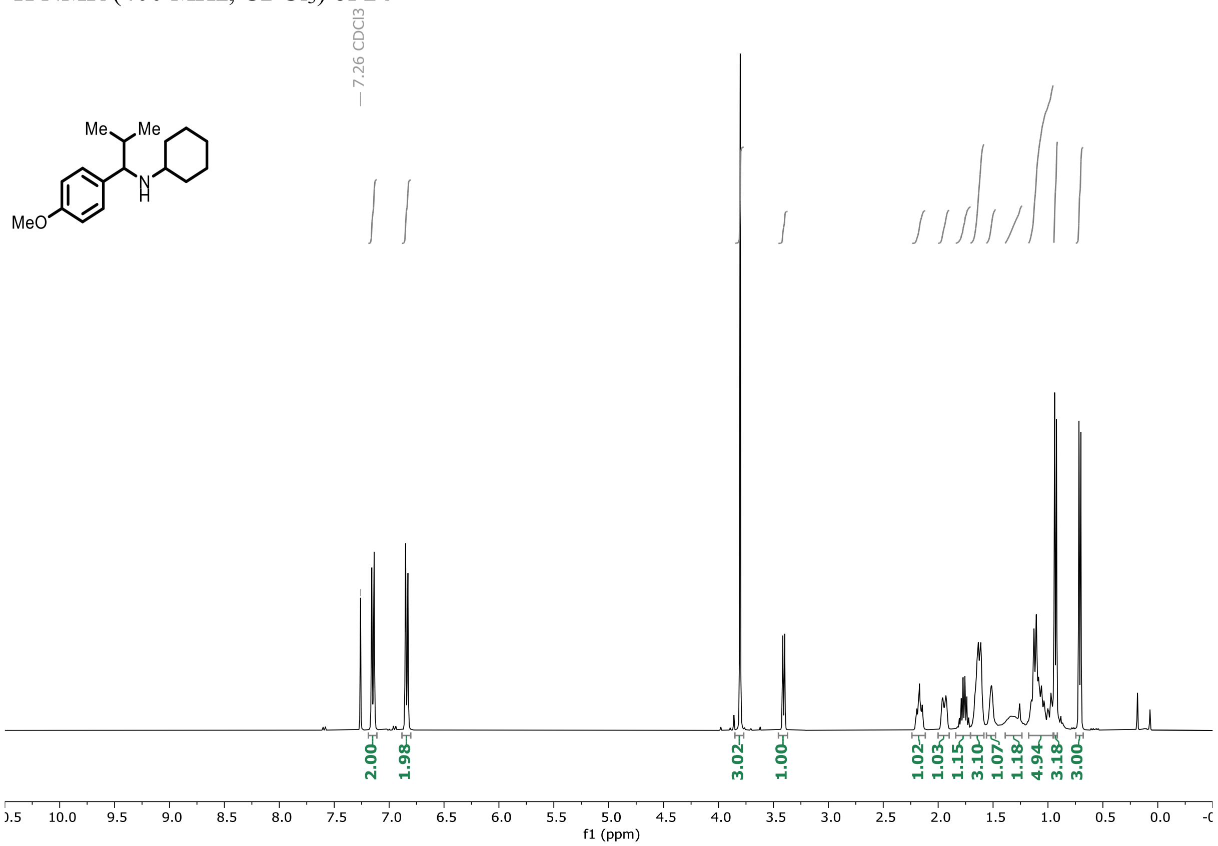

^{13}C NMR (101 MHz, $CDCl_3$) of **24**

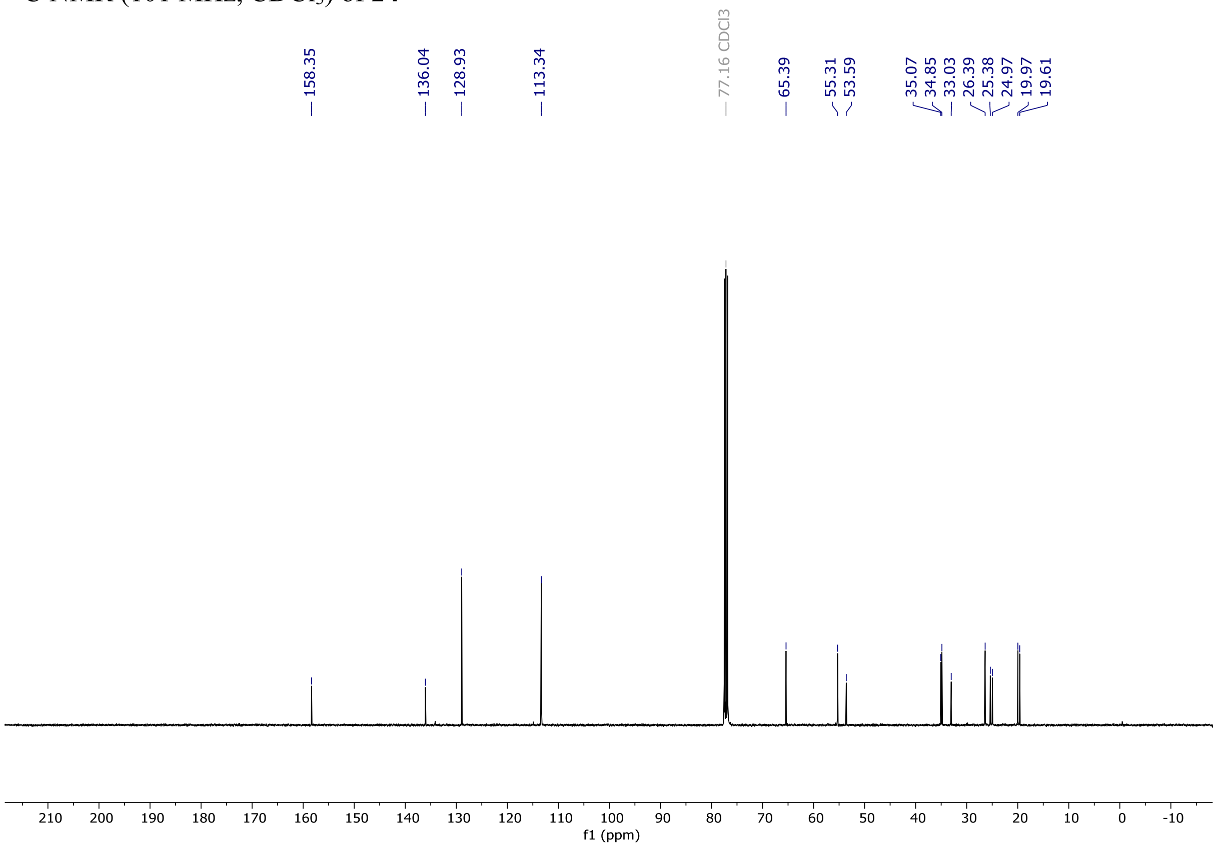

^{1}H NMR (400 MHz, $CDCl_3$) of **25**

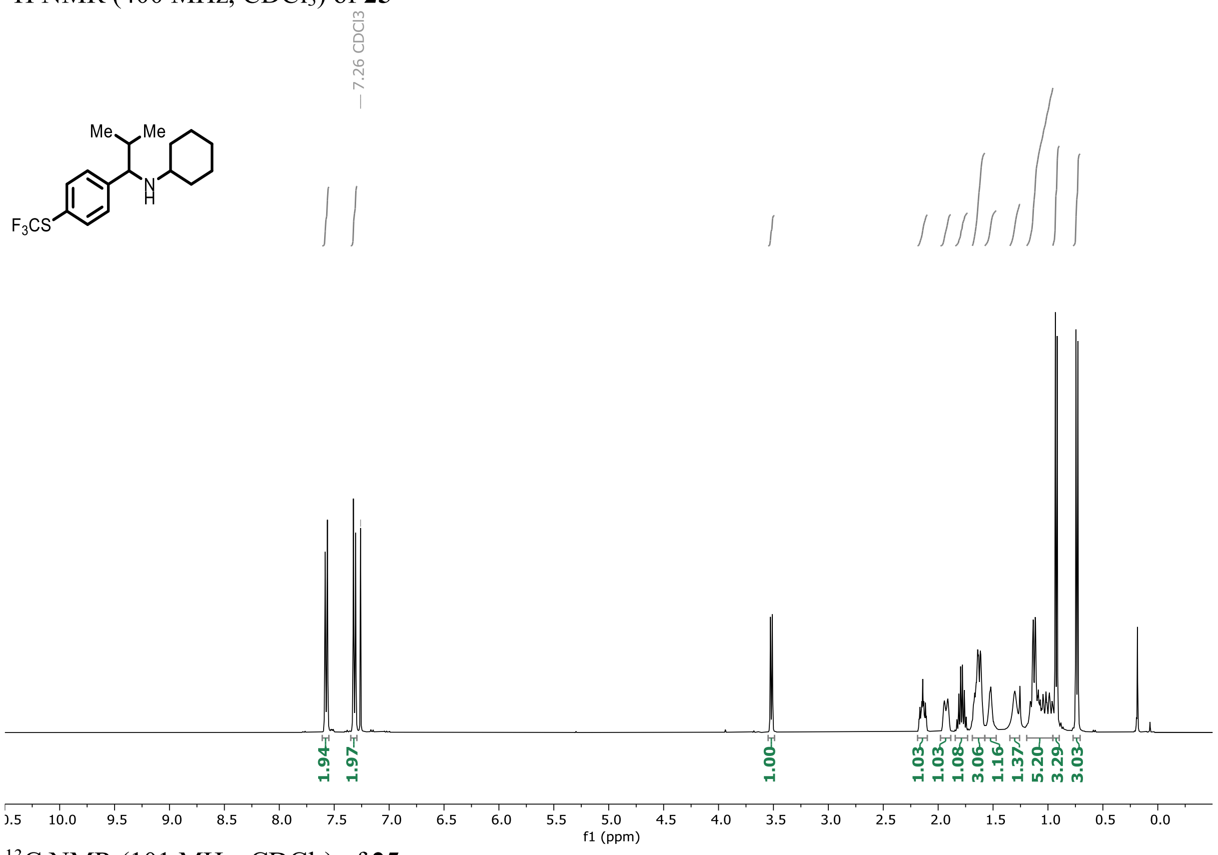

^{13}C NMR (101 MHz, $CDCl_3$) of **25**

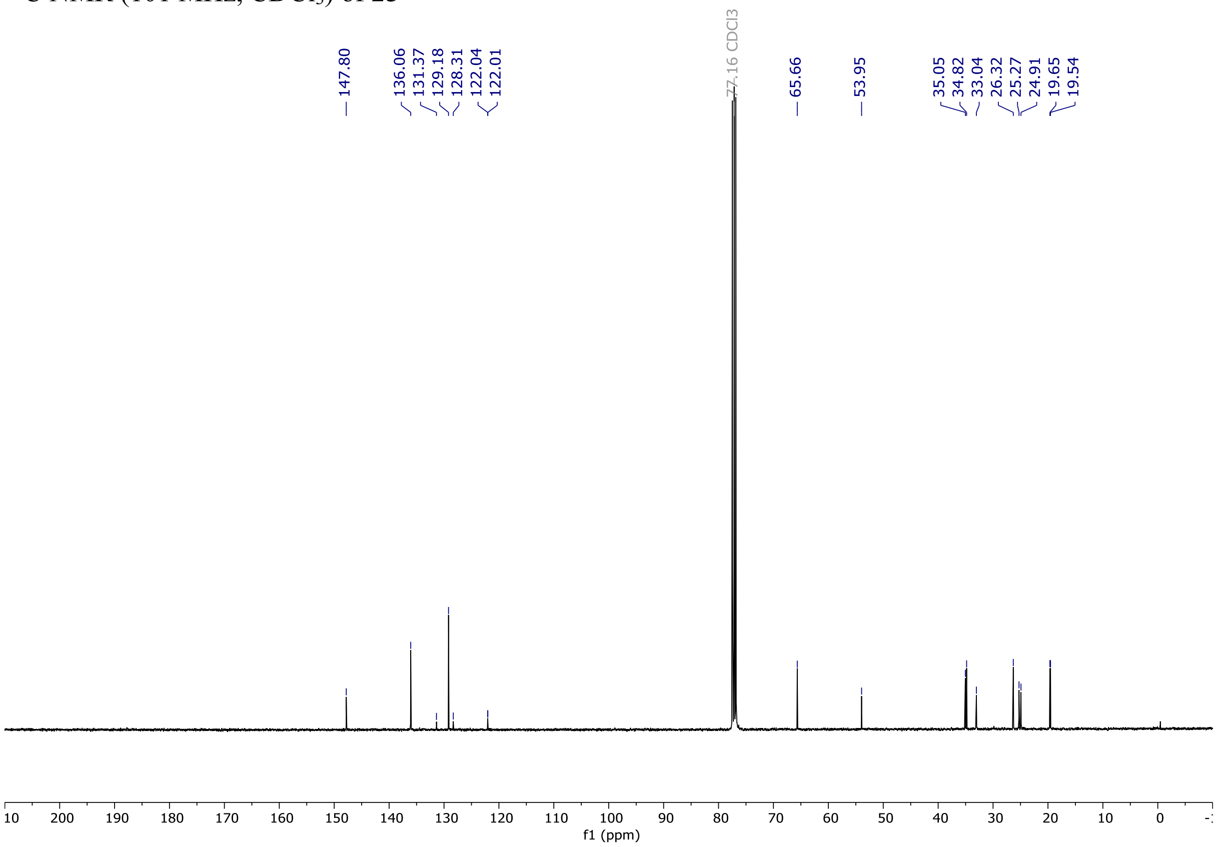

^{19}F NMR (282 MHz, $CDCl_3$) of **25**

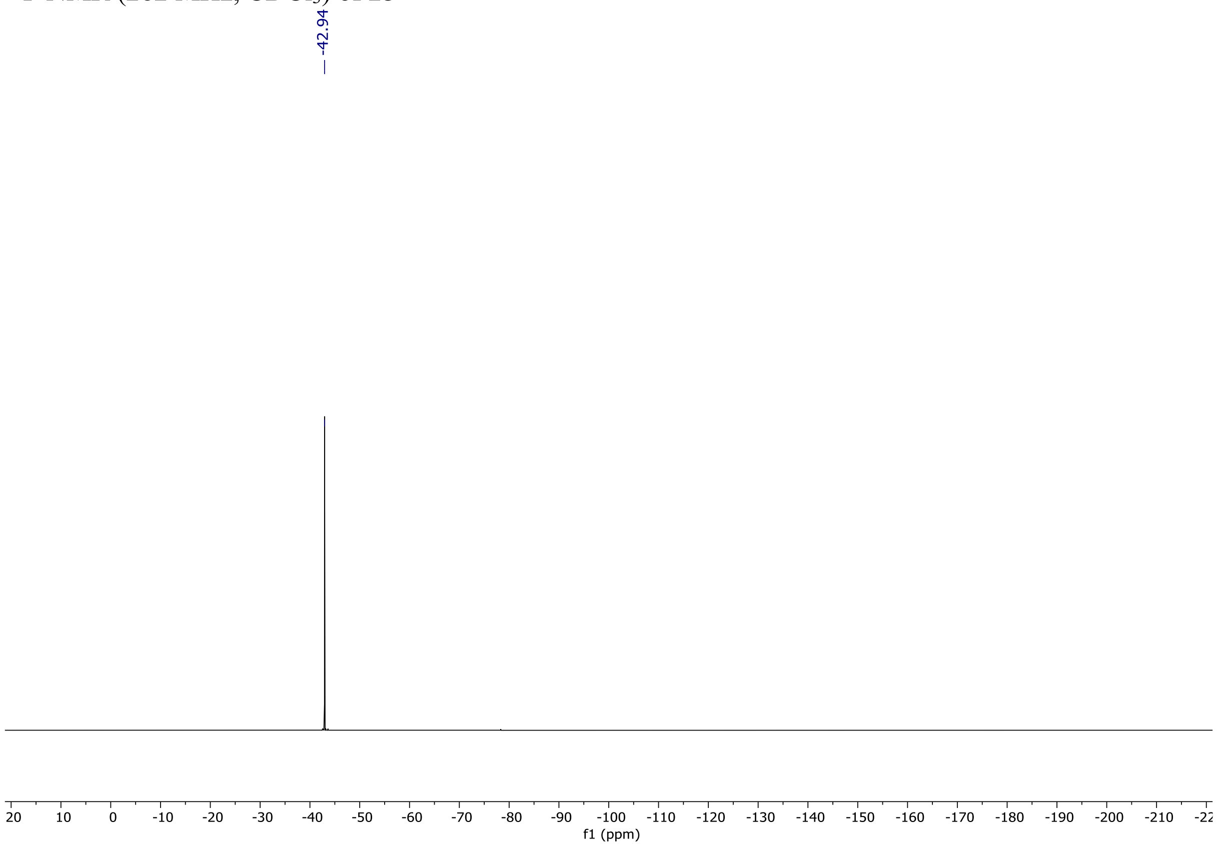

^{1}H NMR (400 MHz, $CDCl_3$) of **26**

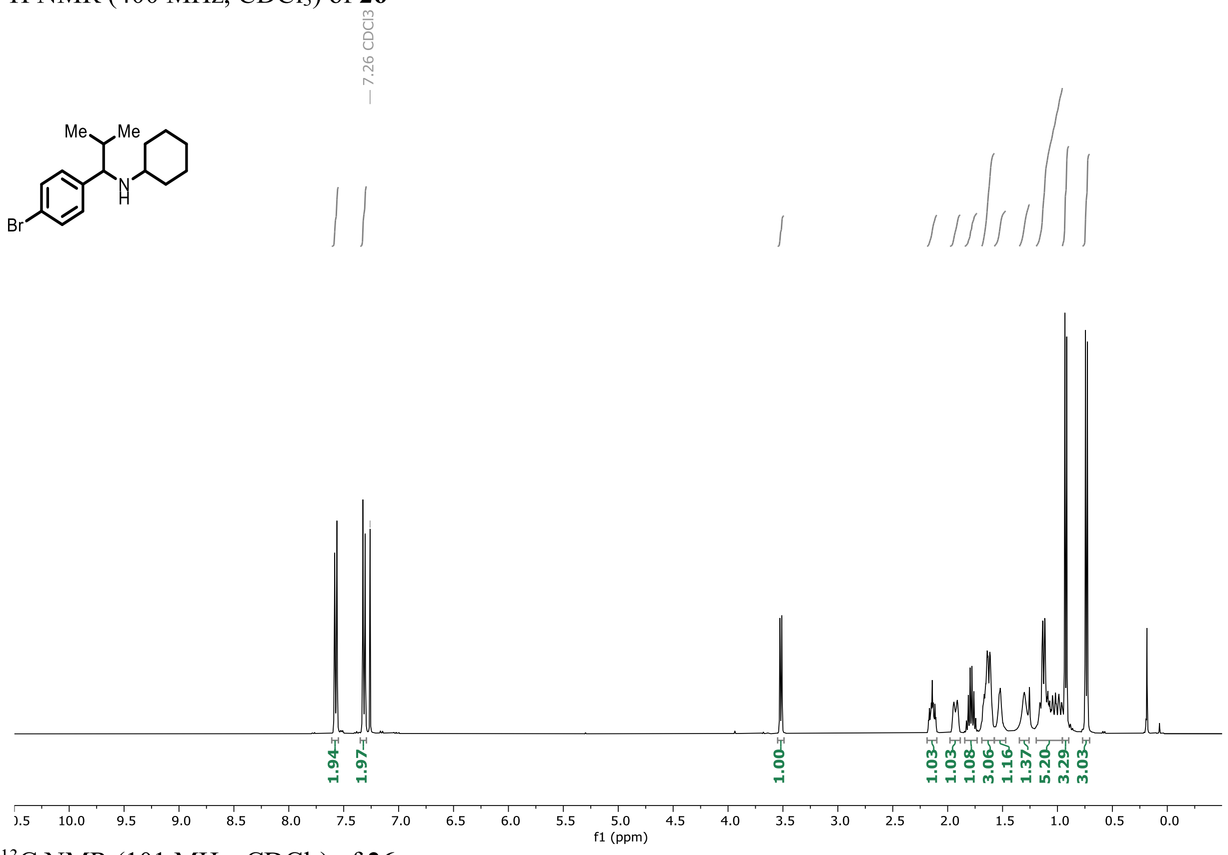

^{13}C NMR (101 MHz, $CDCl_3$) of **26**

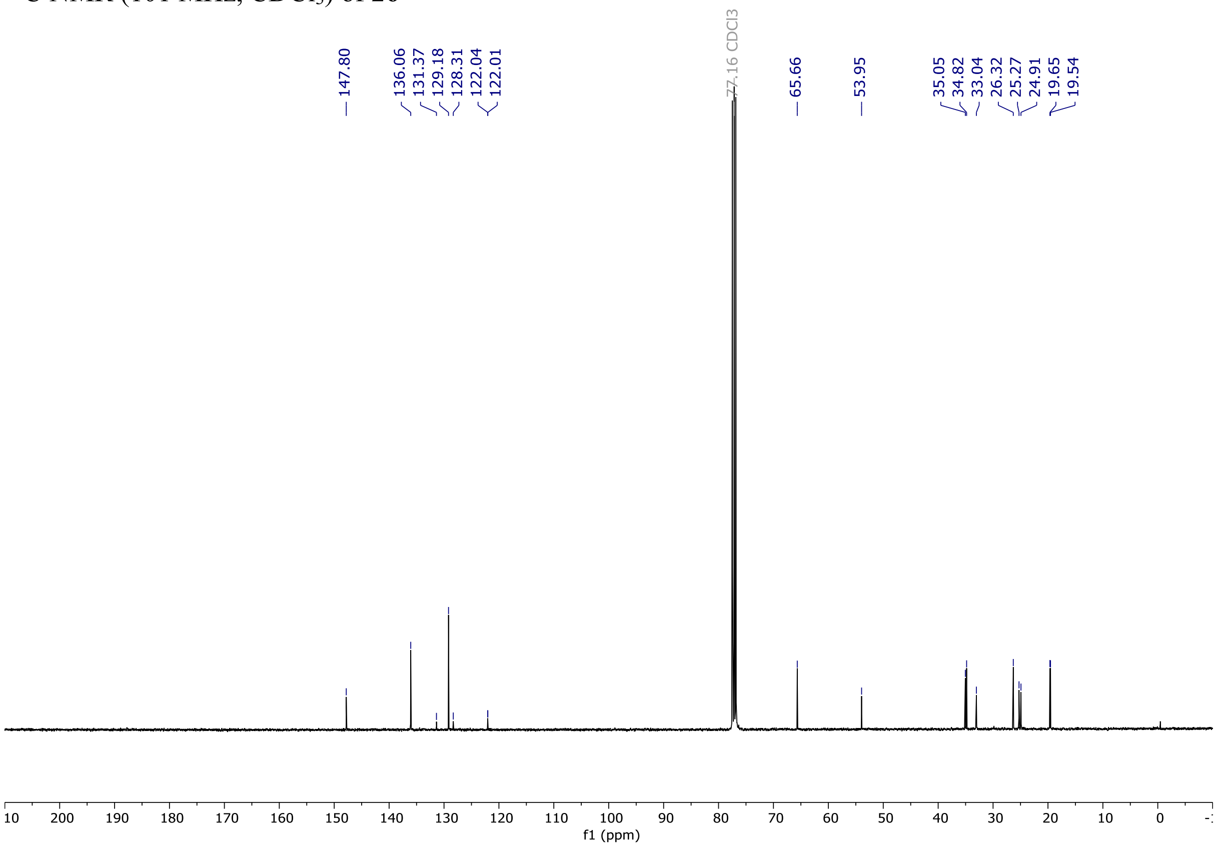

^{1}H NMR (300 MHz, $CDCl_3$) of **27**

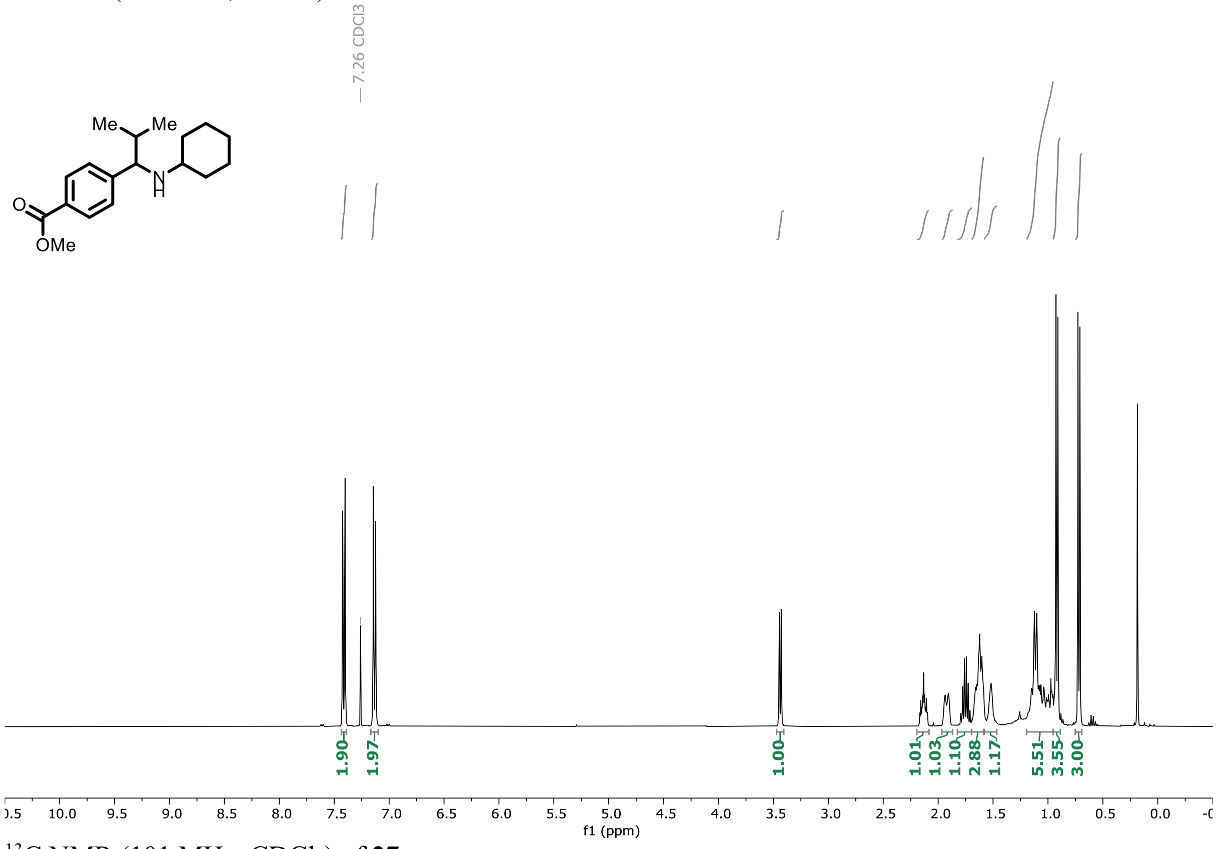

^{13}C NMR (101 MHz, $CDCl_3$) of **27**

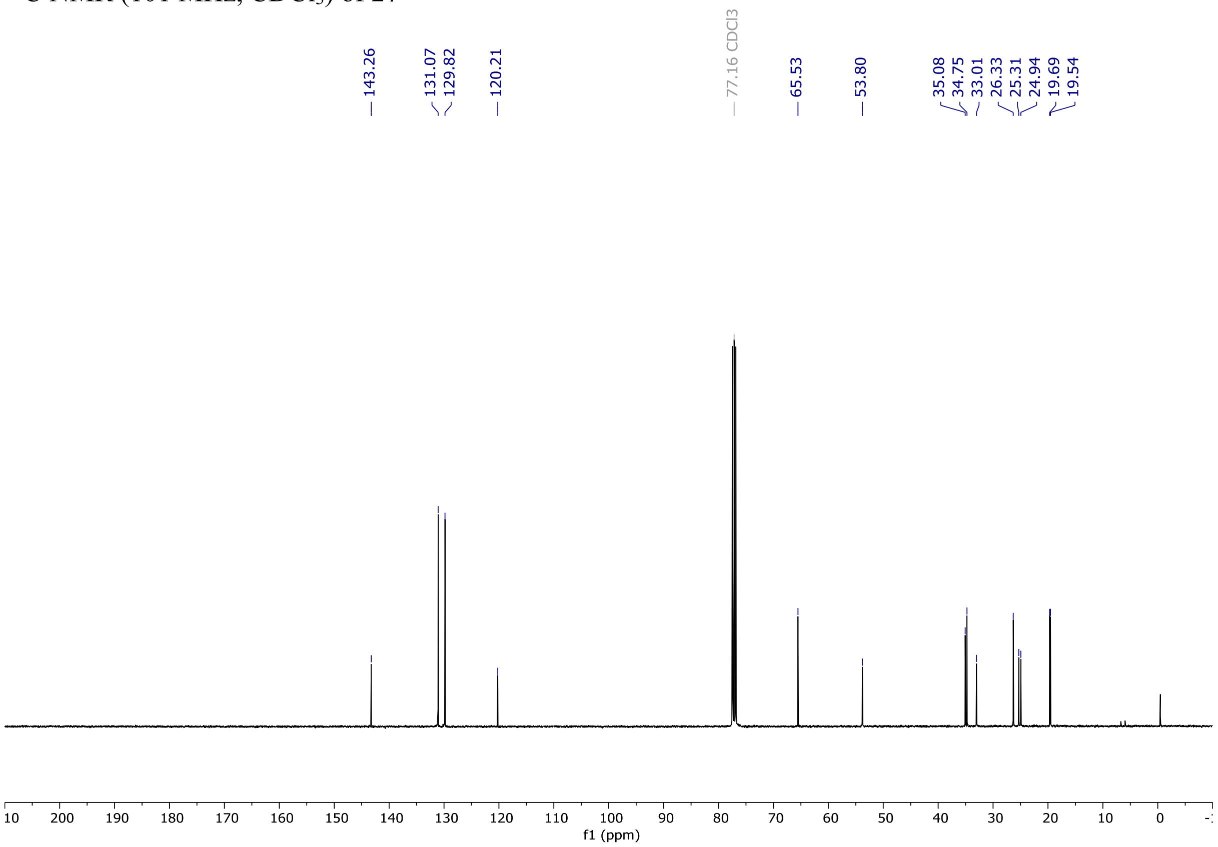

^{1}H NMR (400 MHz, $CDCl_3$) of **28**

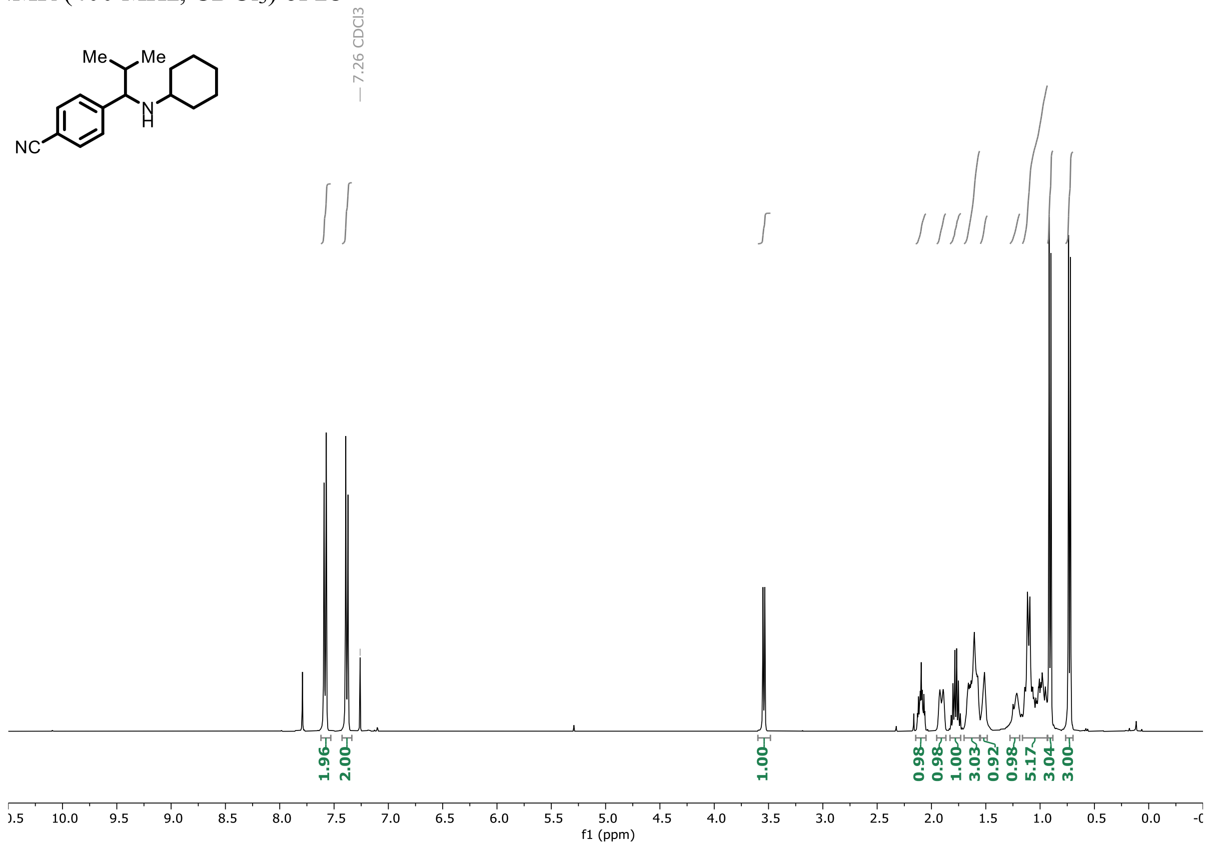

^{13}C NMR (101 MHz, $CDCl_3$) of **28**

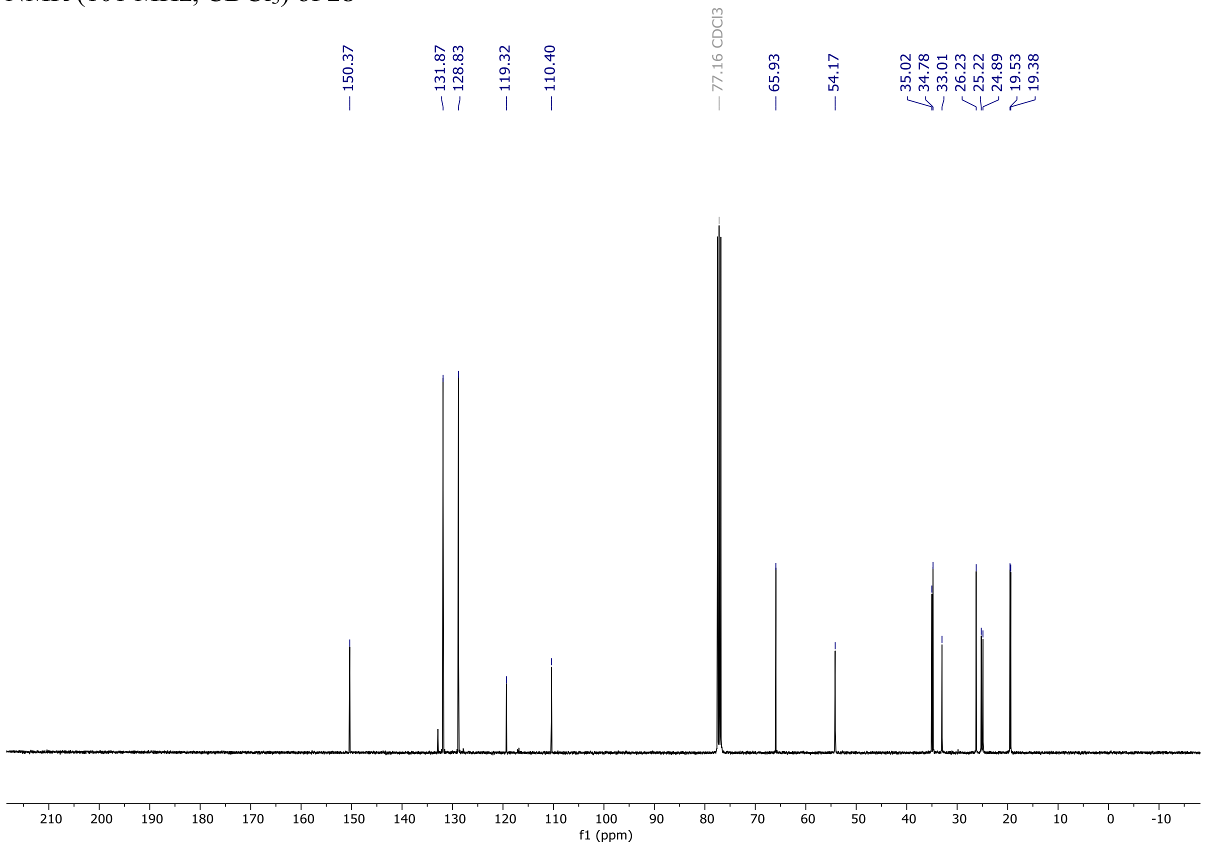

^{1}H NMR (400 MHz, $CDCl_3$) of **29**

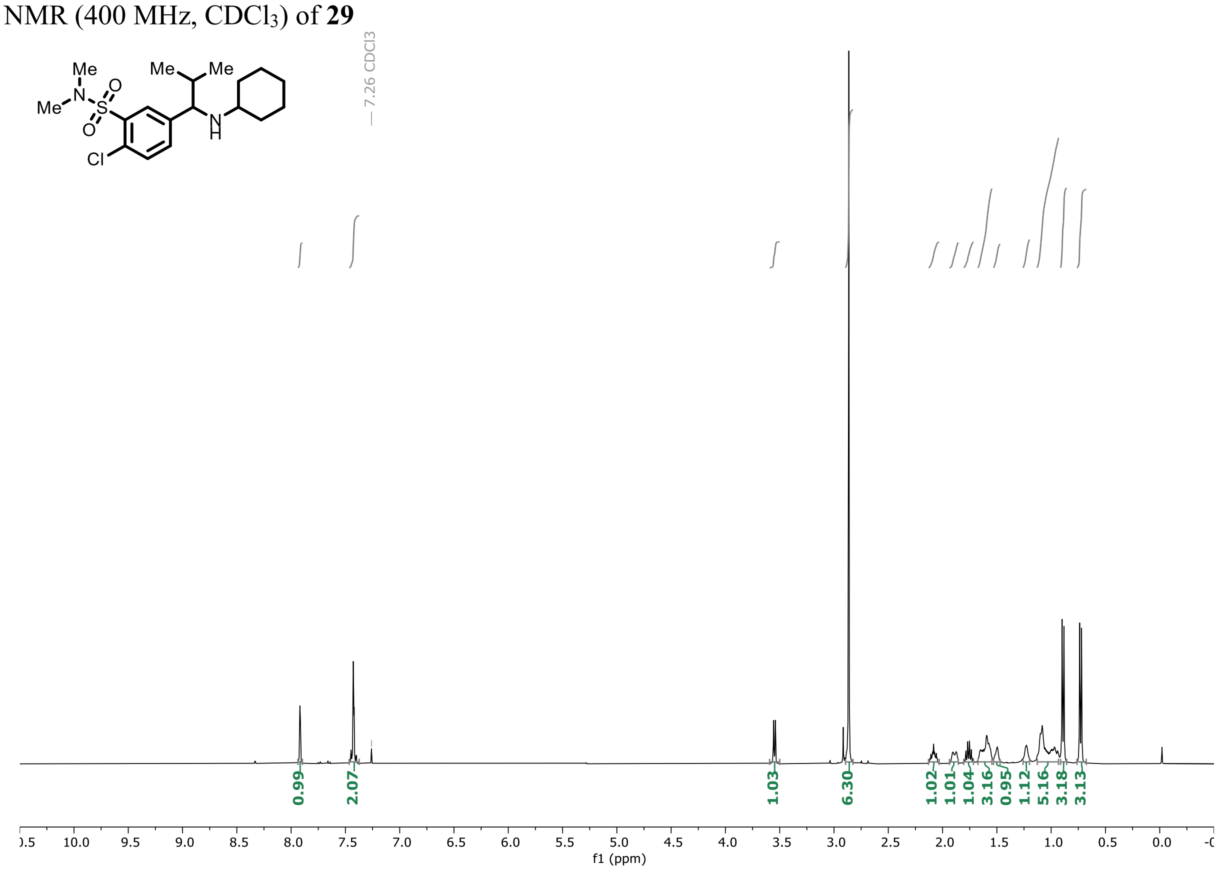

^{13}C NMR (101 MHz, $CDCl_3$) of **29**

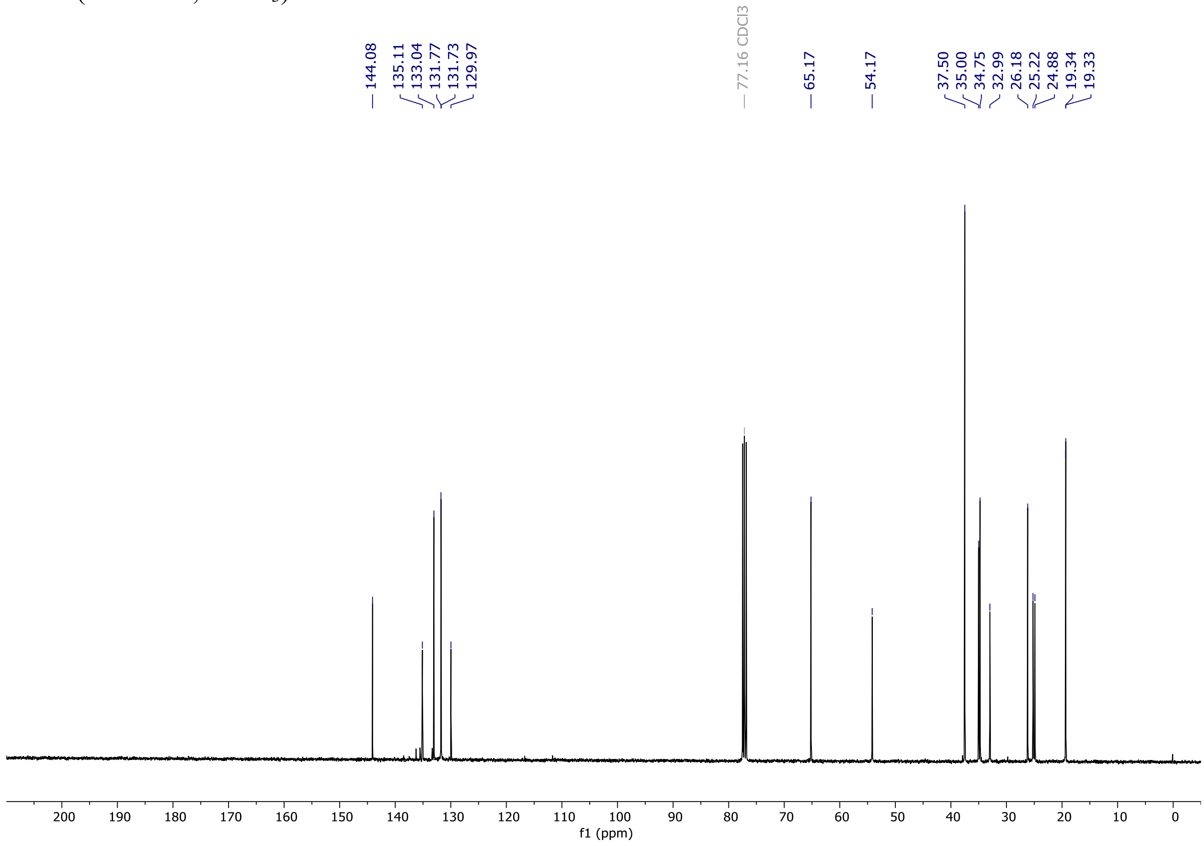

^{1}H NMR (400 MHz, $CDCl_3$) of **30**

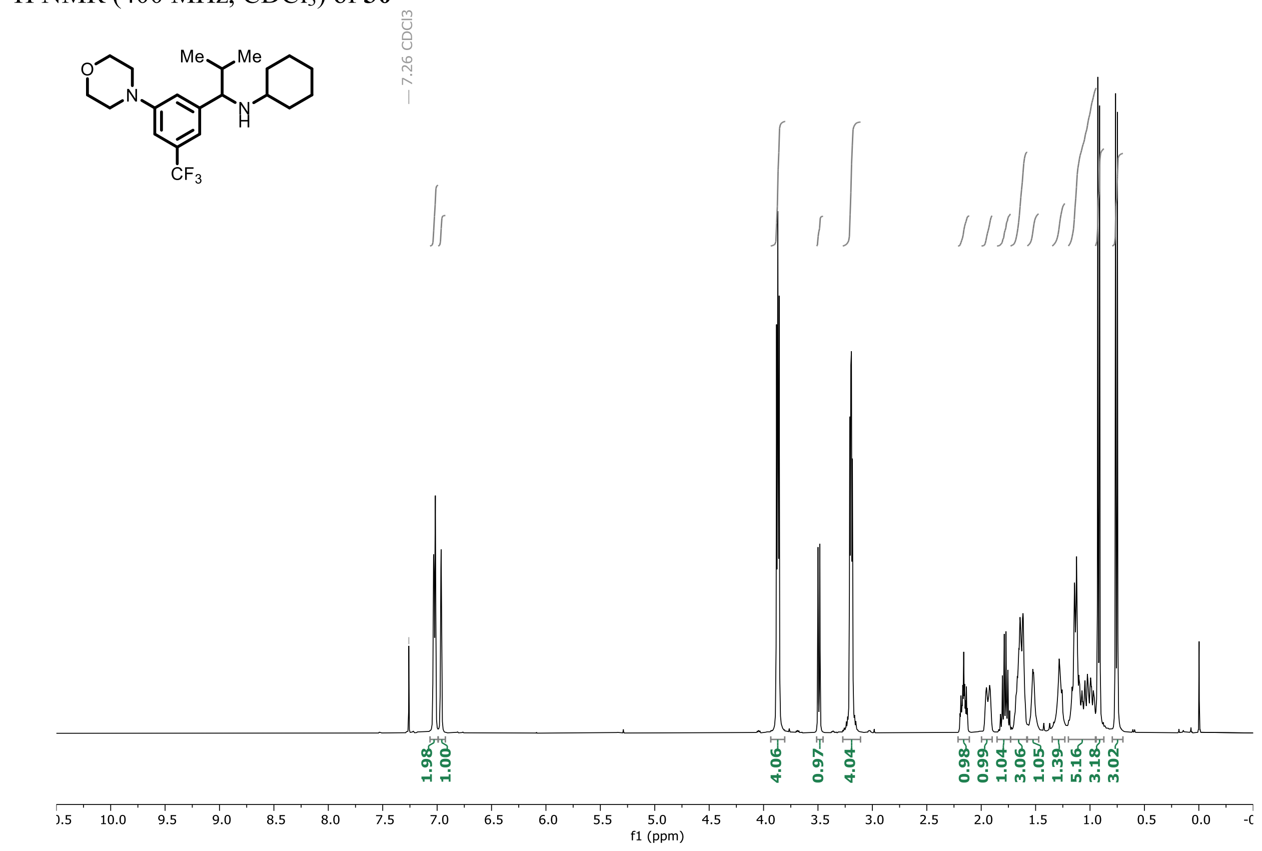

^{13}C NMR (101 MHz, $CDCl_3$) of **30**

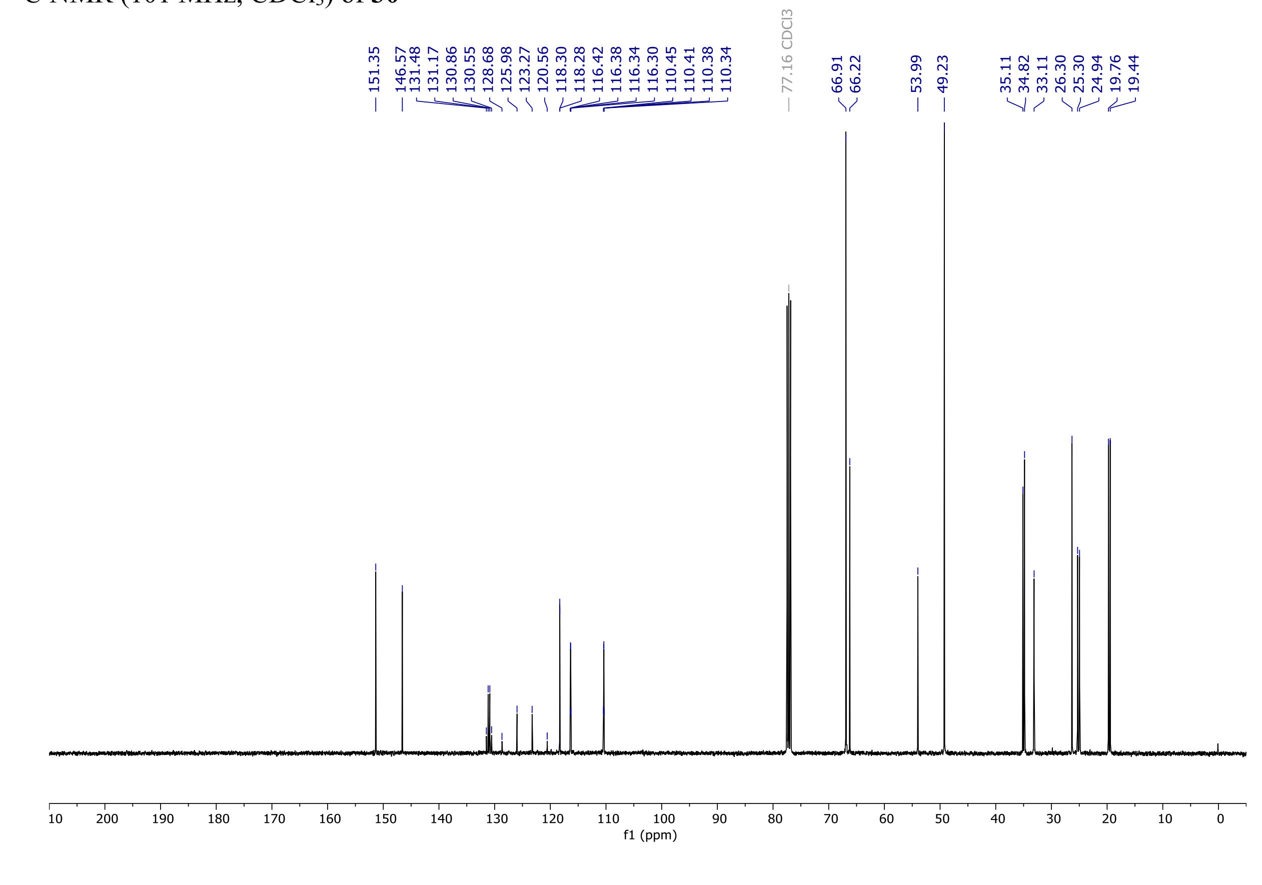

^{19}F NMR (377 MHz, $CDCl_3$) of **30**

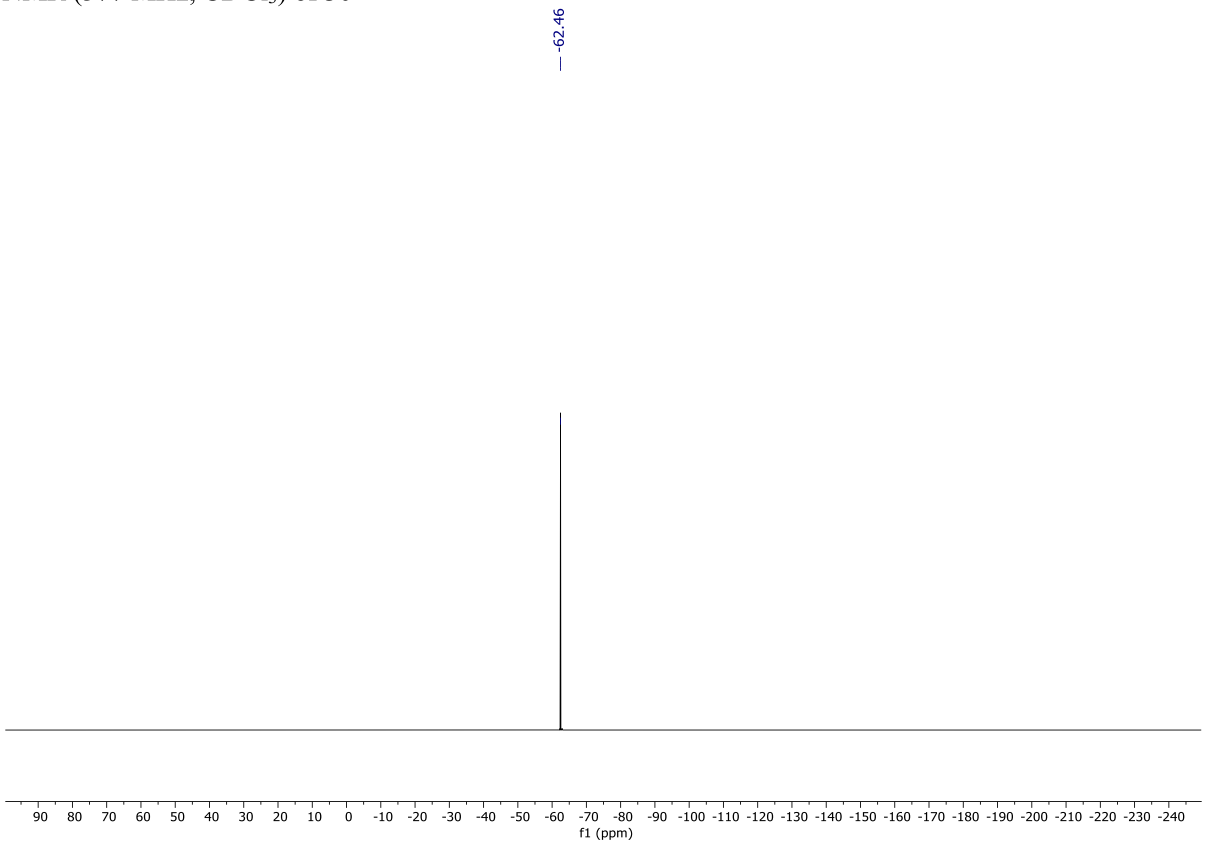

^{1}H NMR (400 MHz, $CDCl_3$) of **31**

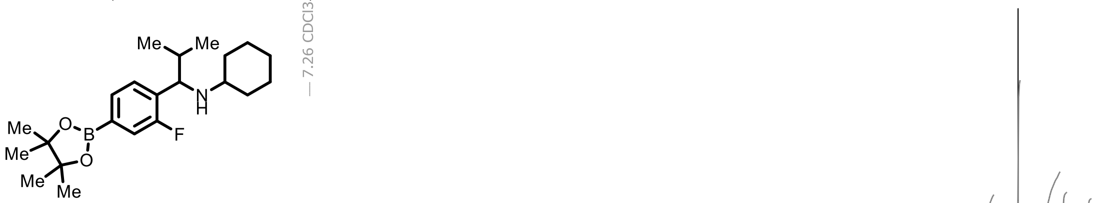

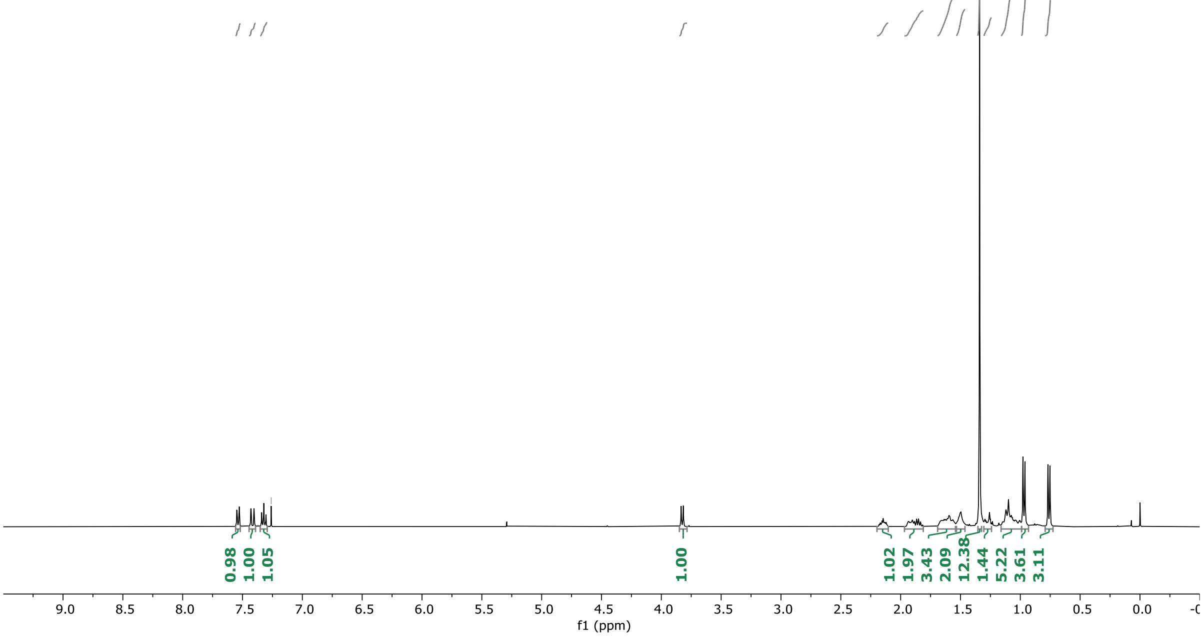

^{13}C NMR (101 MHz, $CDCl_3$) of **31**

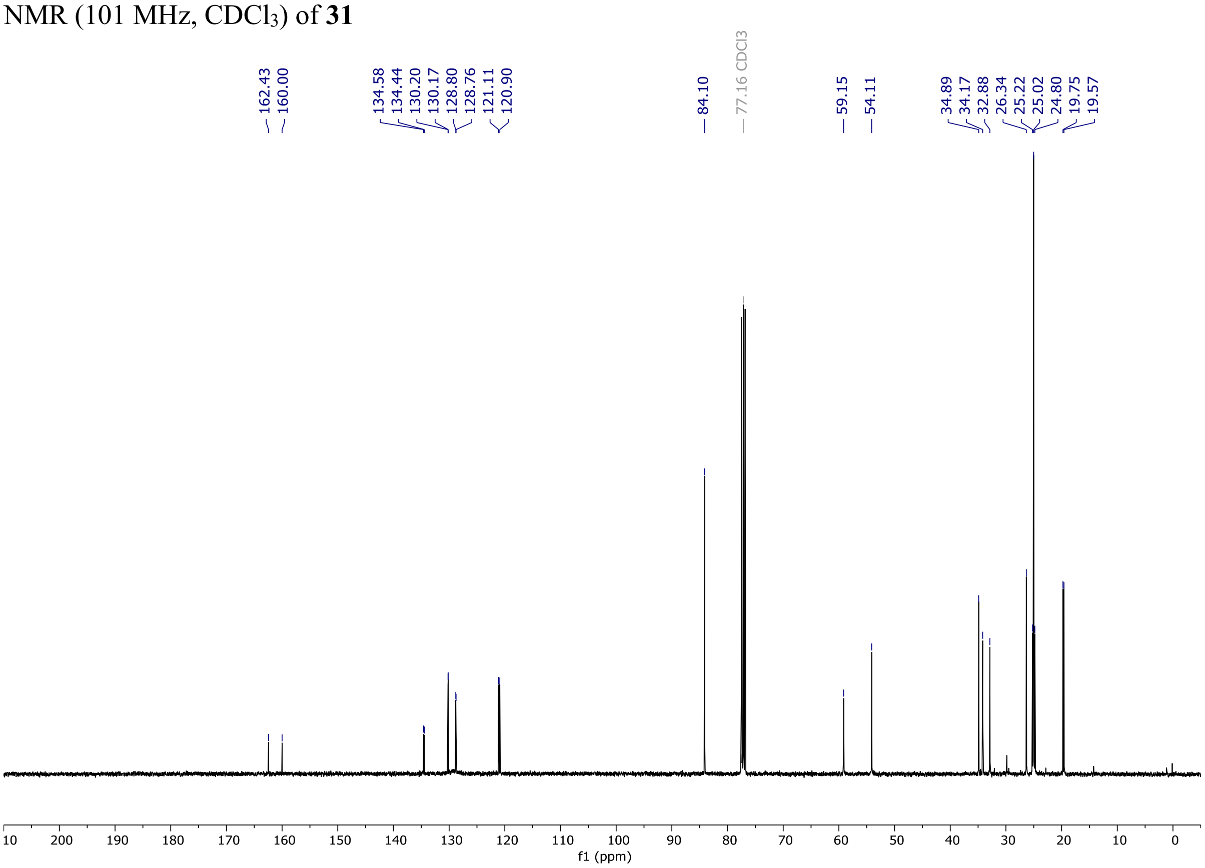

^{11}B NMR (128 MHz, $CDCl_3$) of **31**

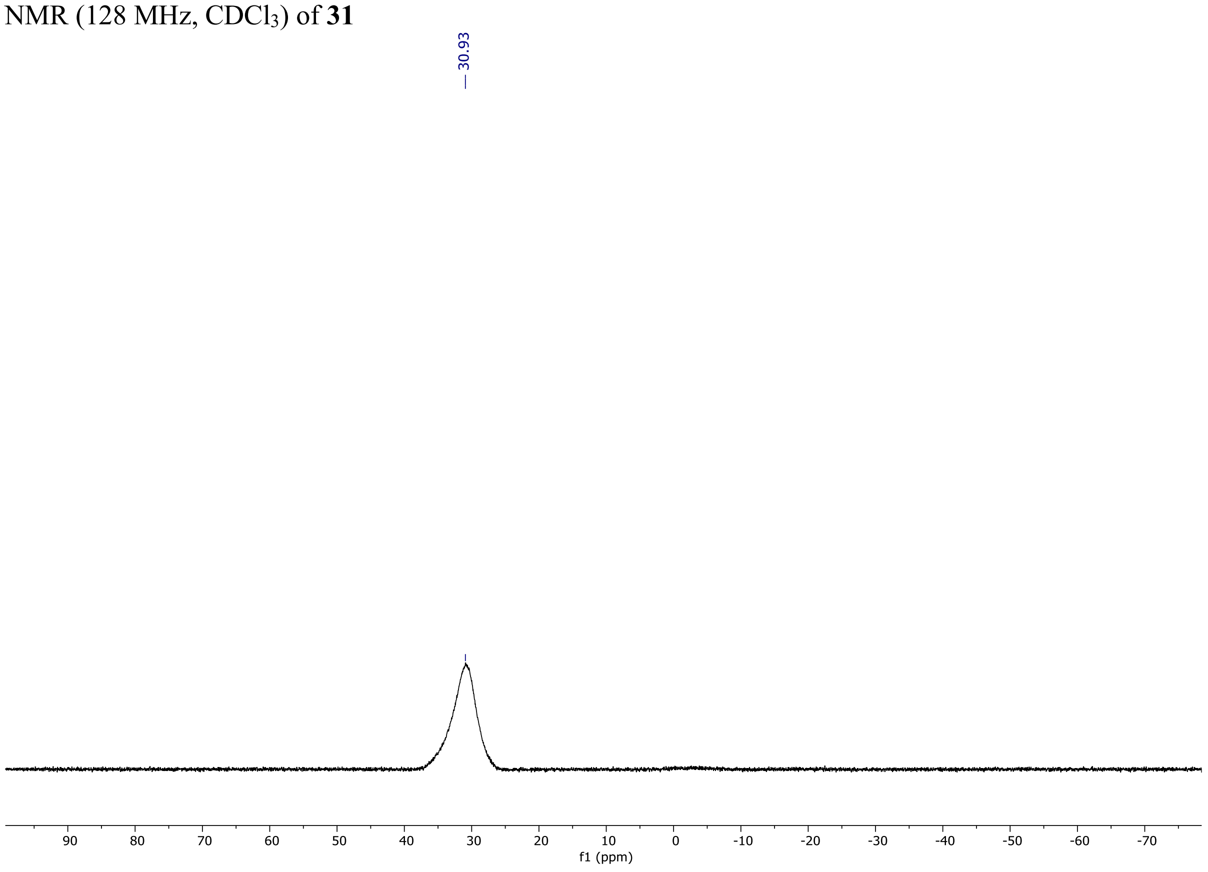

^{19}F NMR (377 MHz, $CDCl_3$) of **31**

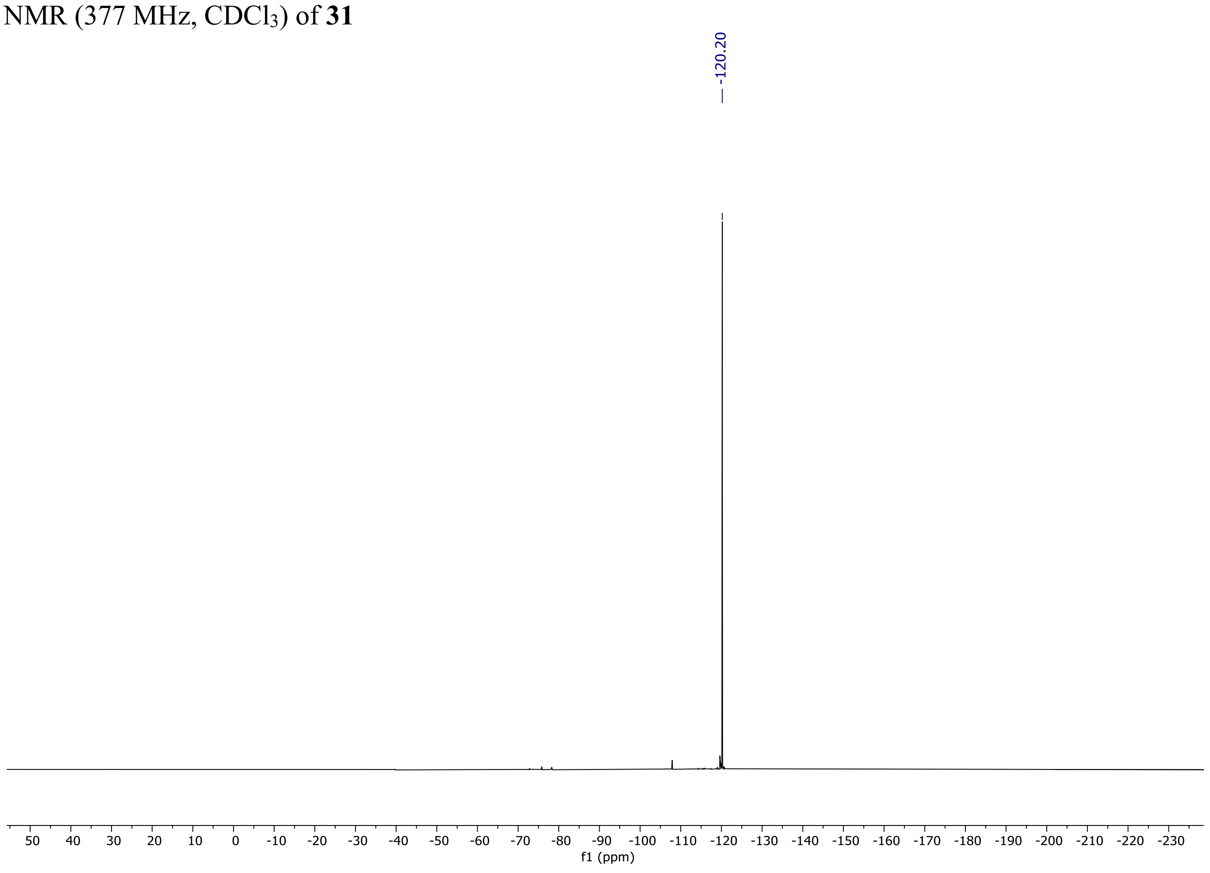

^{1}H NMR (400 MHz, $CDCl_3$) of **32**

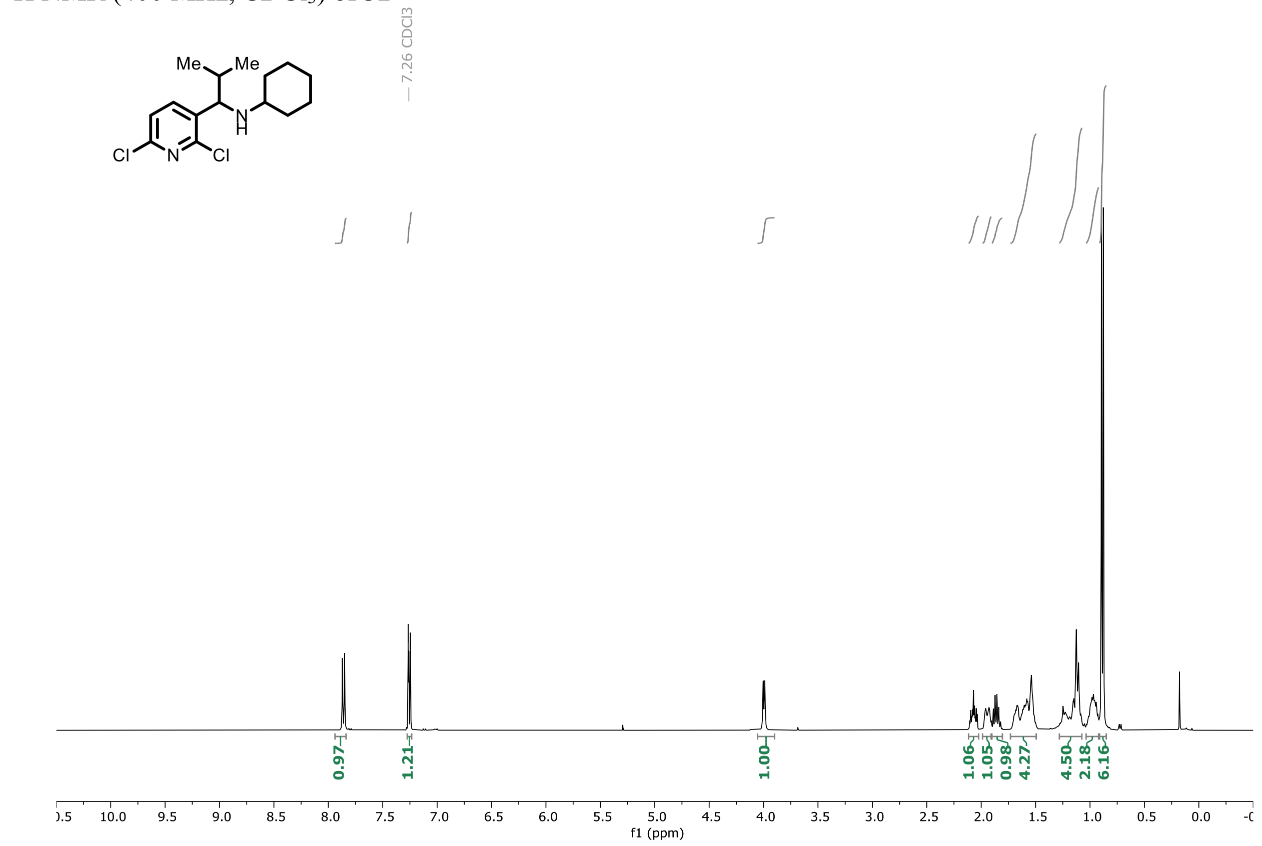

^{13}C NMR (101 MHz, $CDCl_3$) of **32**

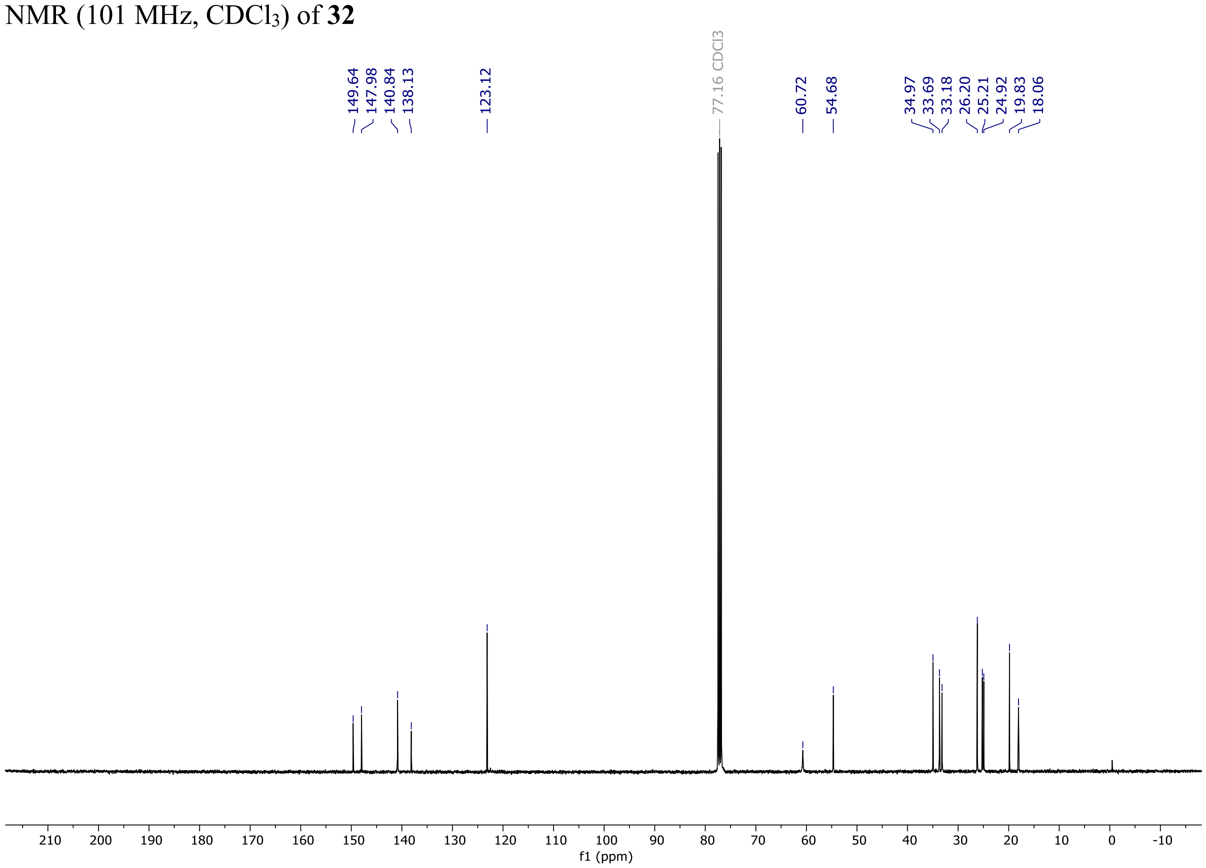

^{1}H NMR (400 MHz, $CDCl_3$) of **33**

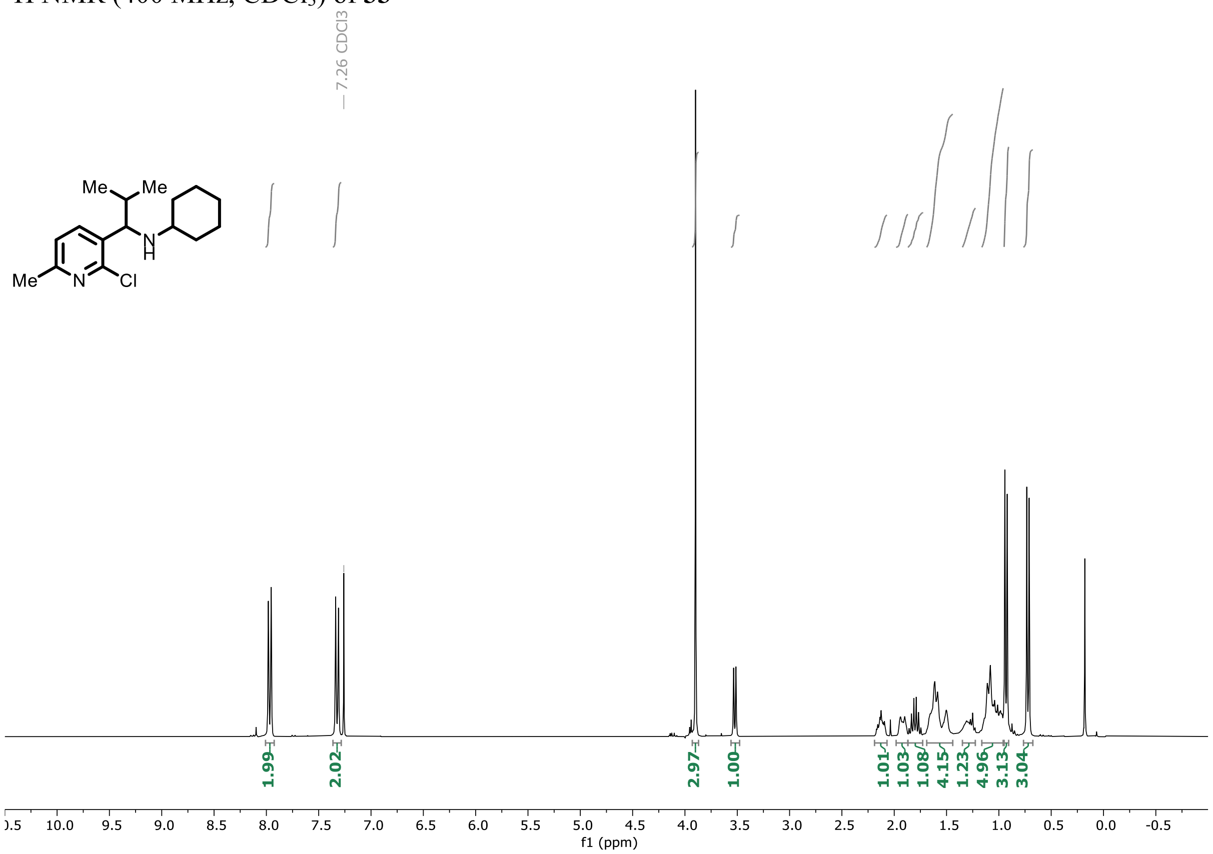

^{13}C NMR (101 MHz, $CDCl_3$) of **33**

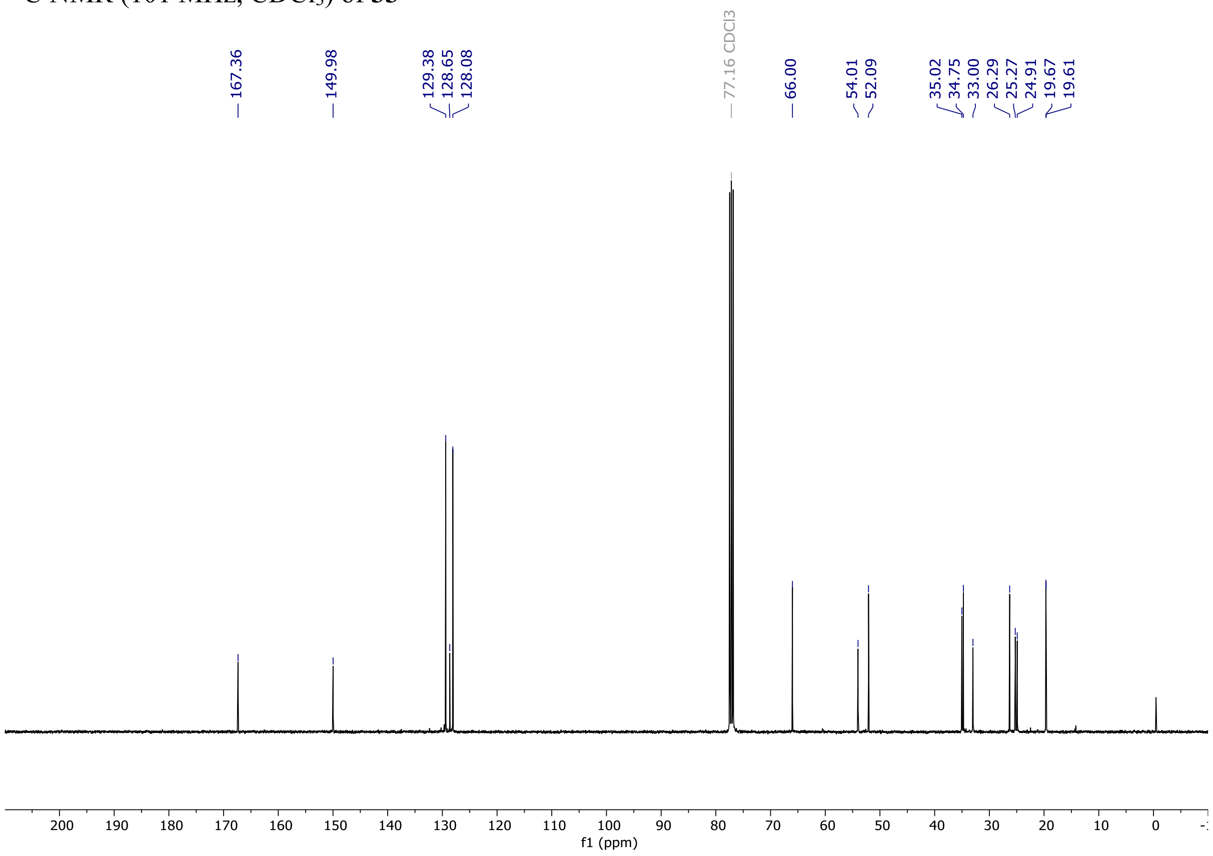

^{1}H NMR (400 MHz, $CDCl_3$) of **34**

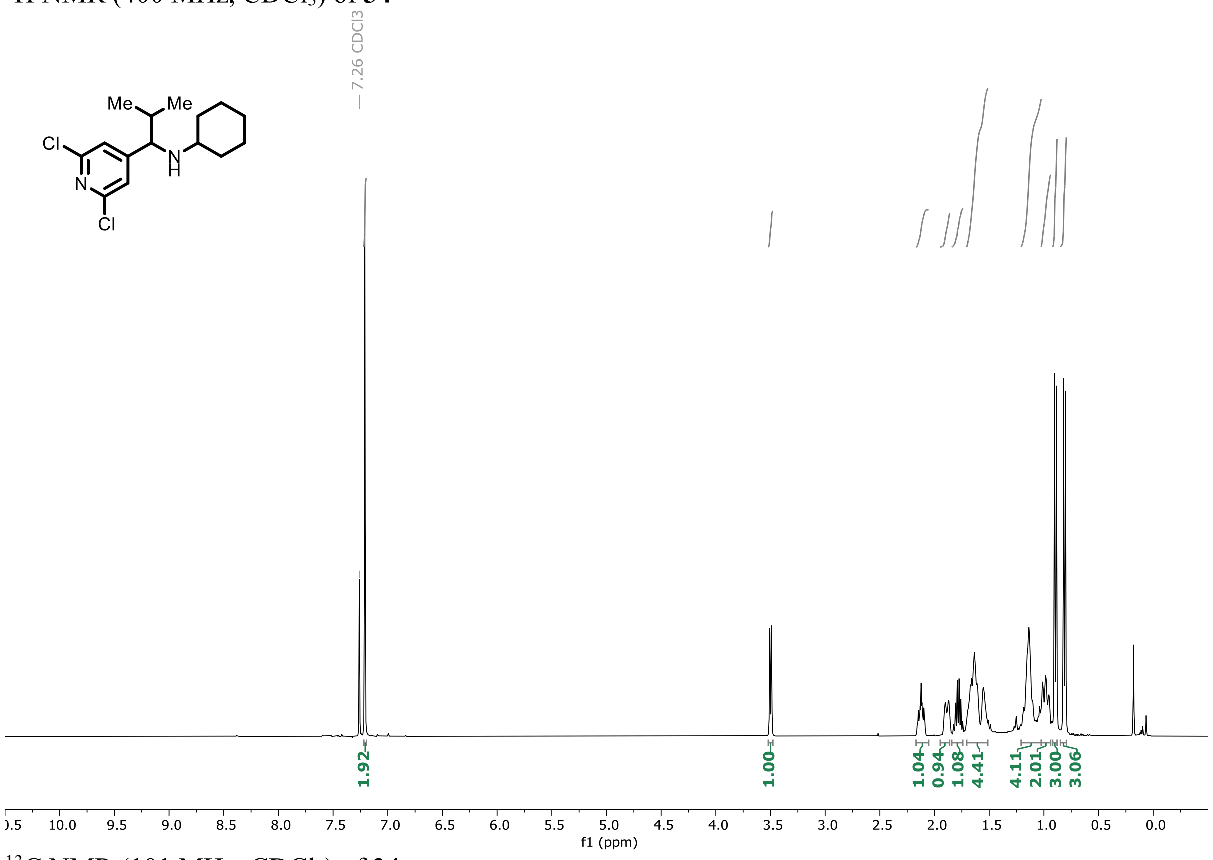

^{13}C NMR (101 MHz, $CDCl_3$) of **34**

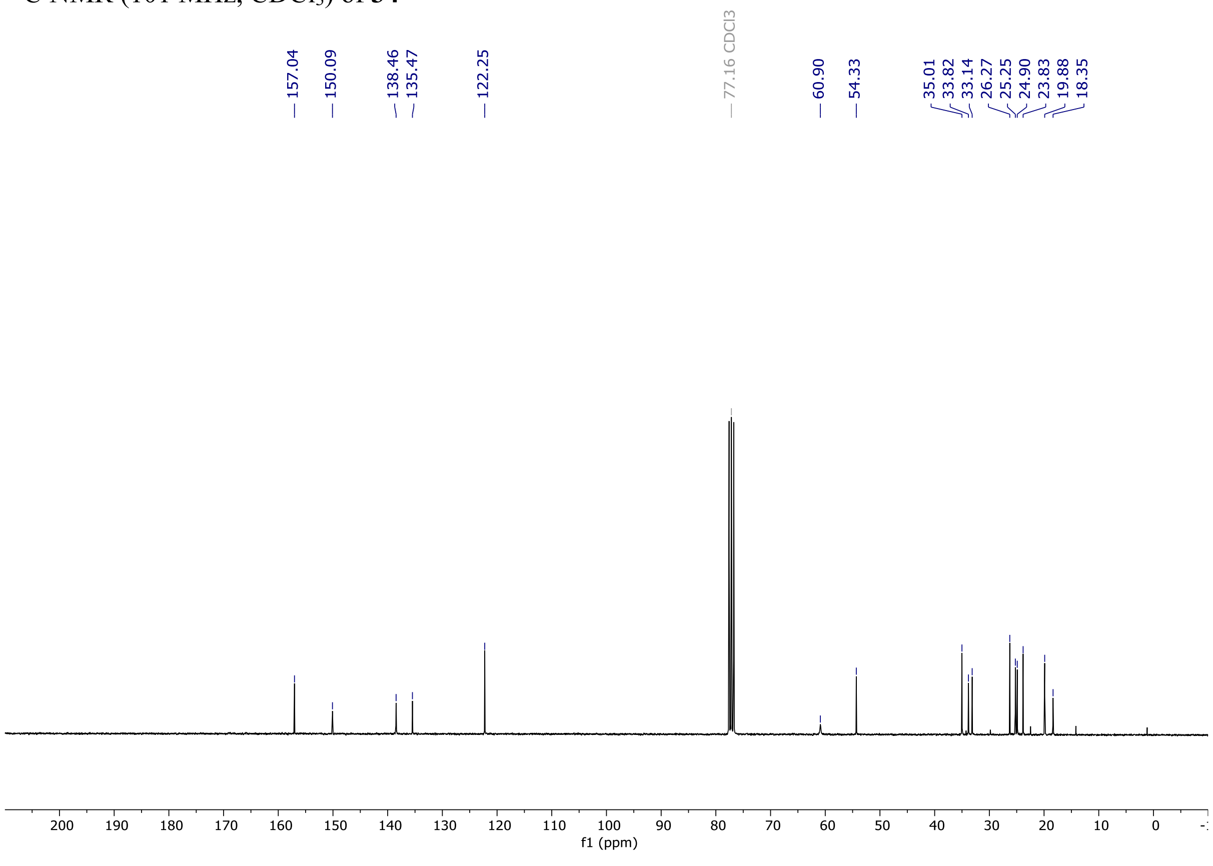

^{1}H NMR (400 MHz, $CDCl_3$) of **35**

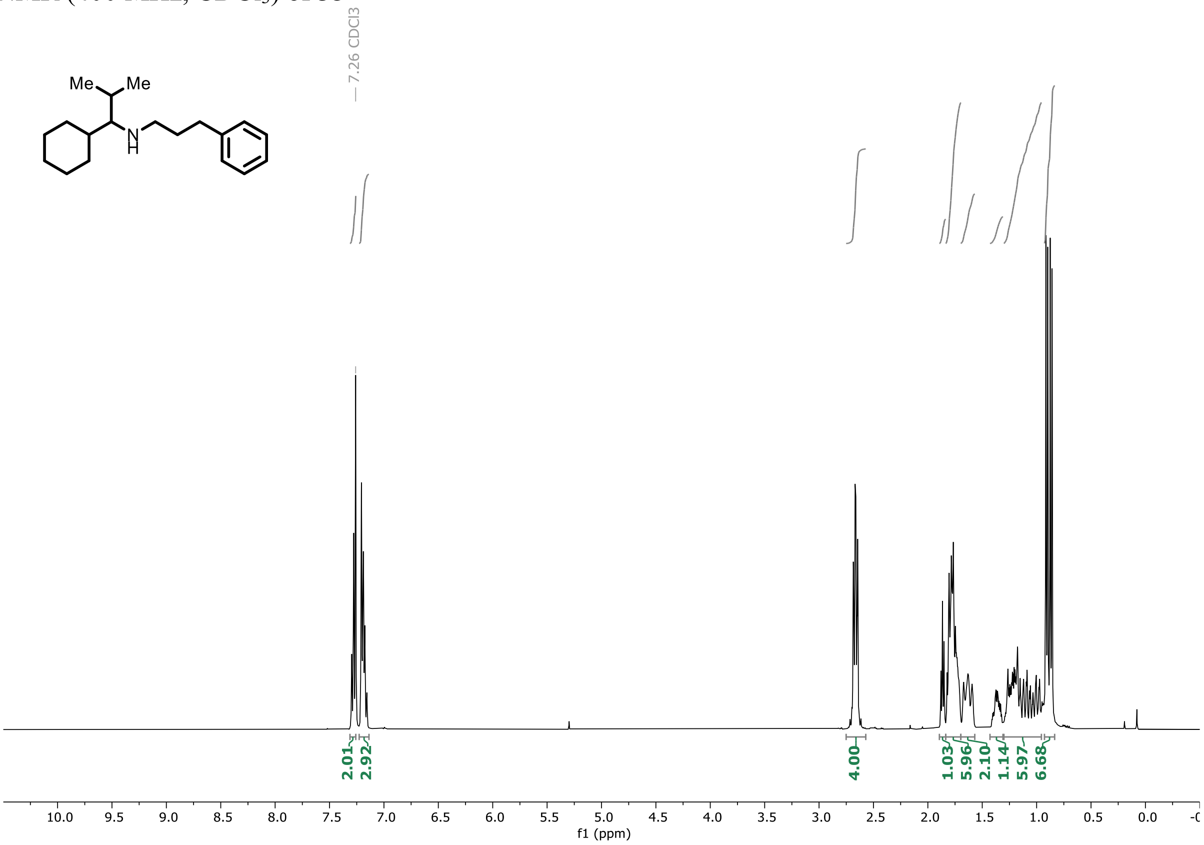

^{13}C NMR (101 MHz, $CDCl_3$) of **35**

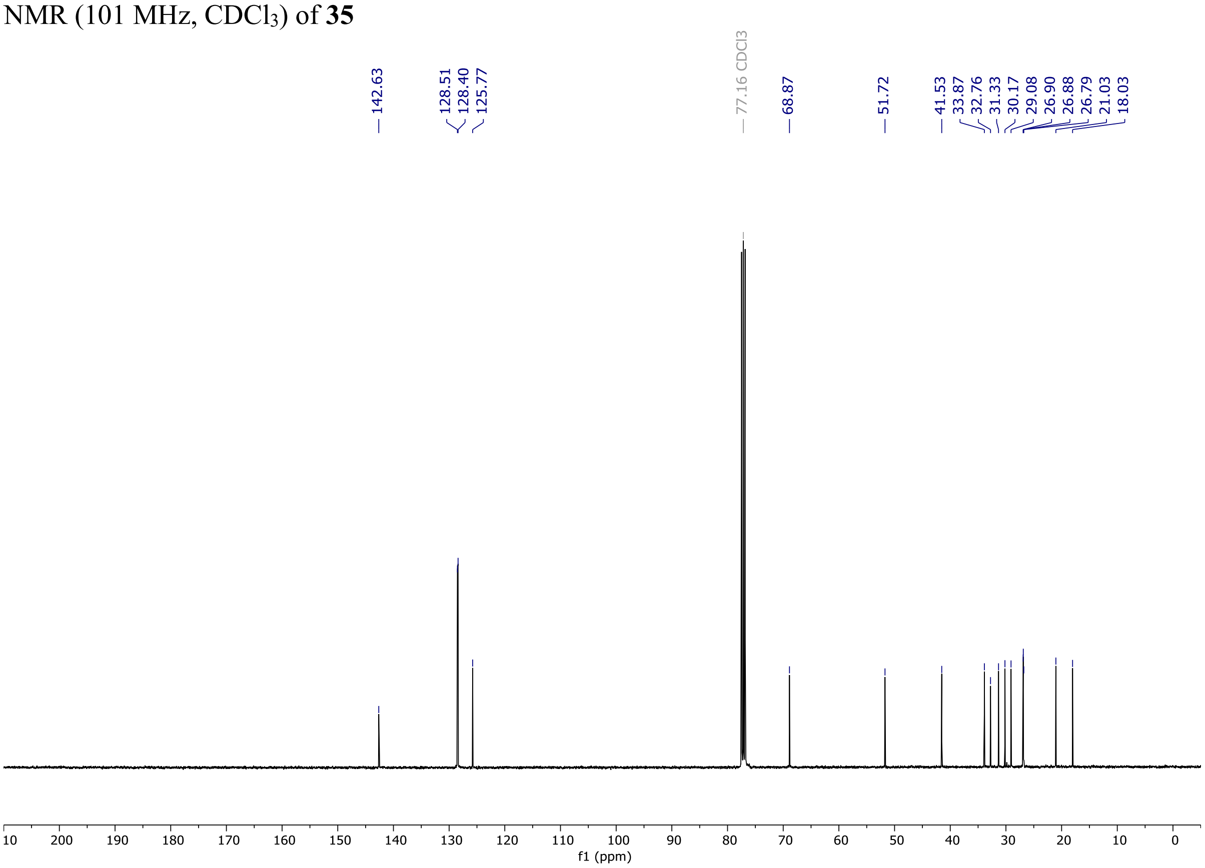

^{1}H NMR (400 MHz, $CDCl_3$) of **36**

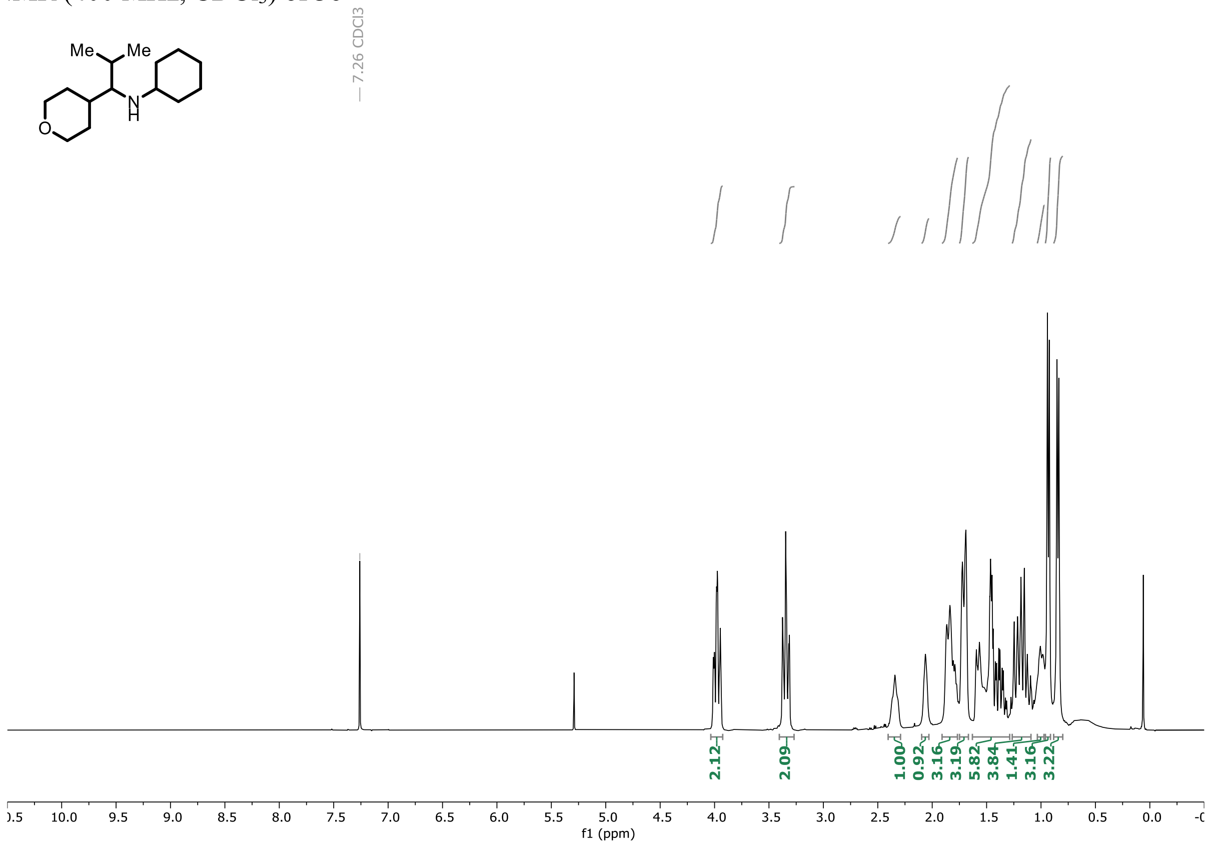

^{13}C NMR (101 MHz, $CDCl_3$) of **36**

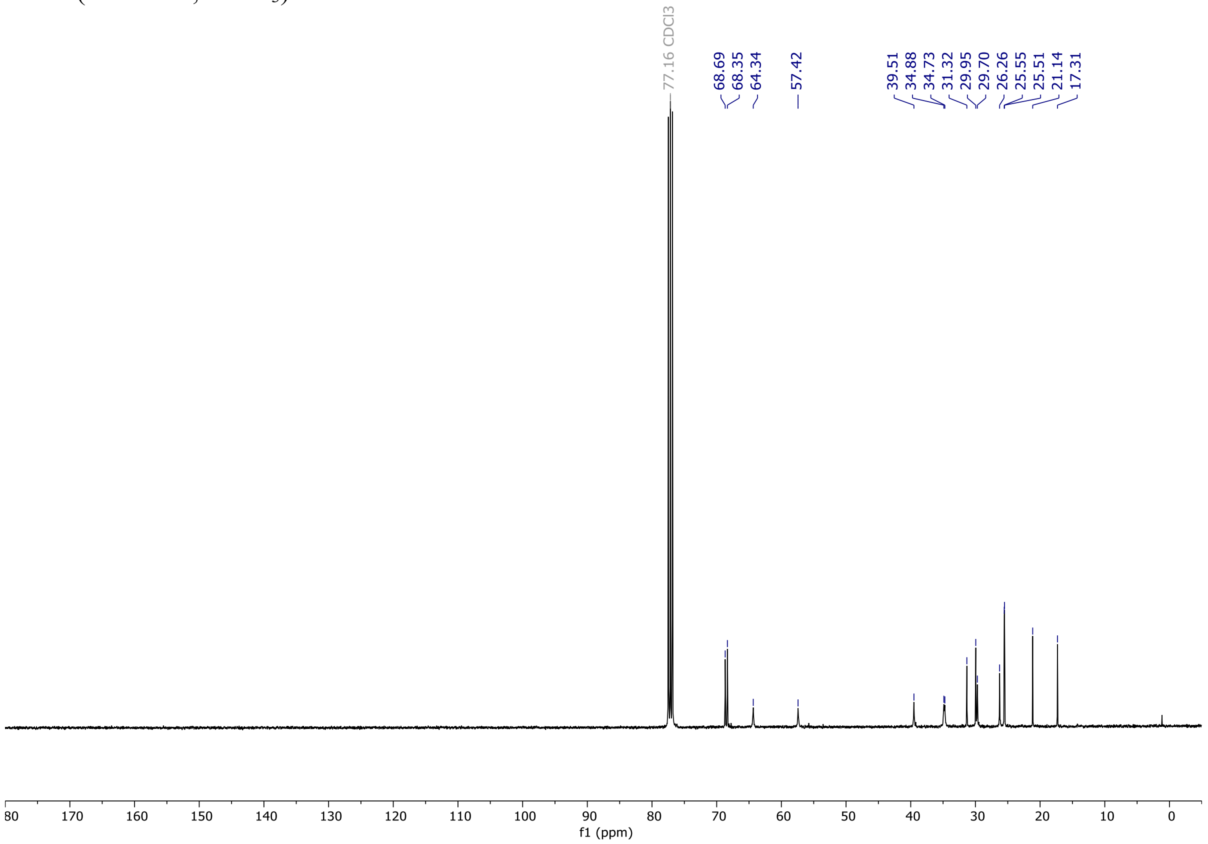

^{1}H NMR (400 MHz, $CDCl_3$) of **37**

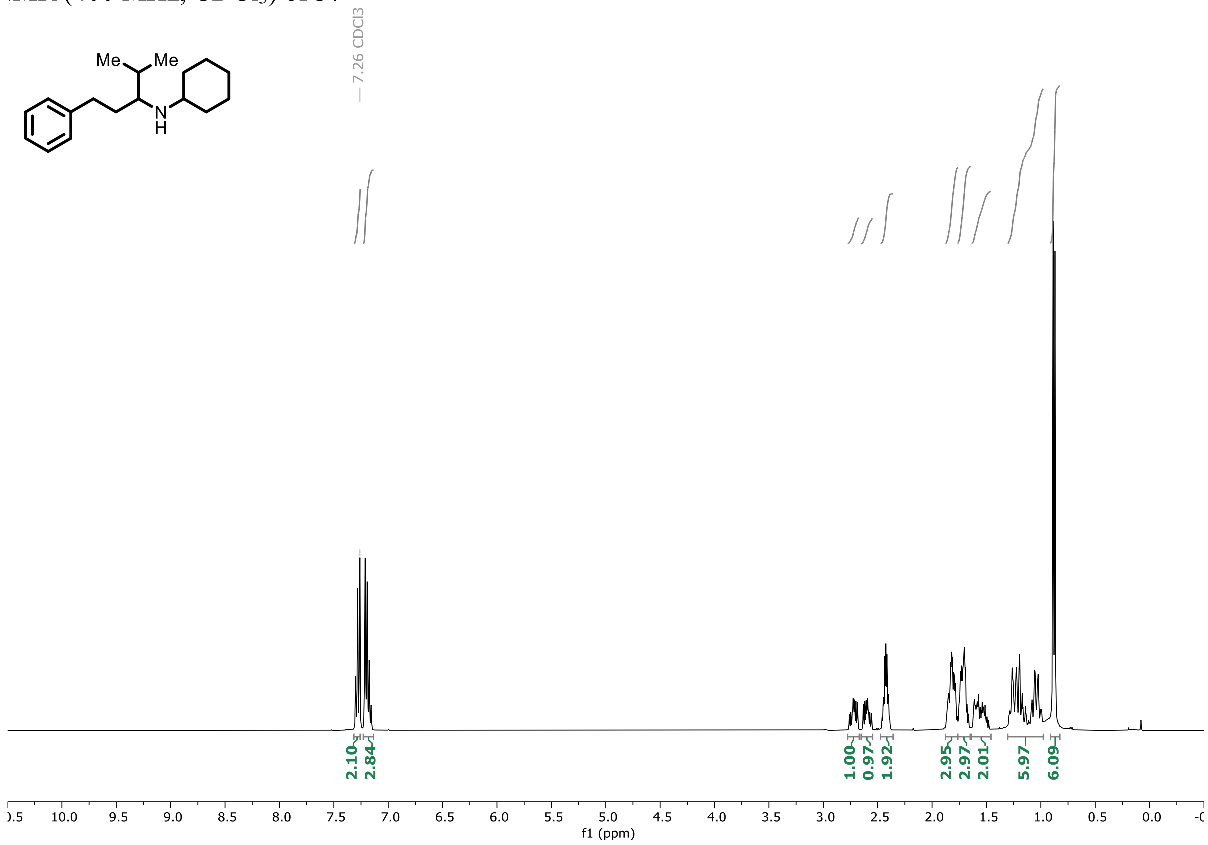

^{13}C NMR (101 MHz, $CDCl_3$) of **37**

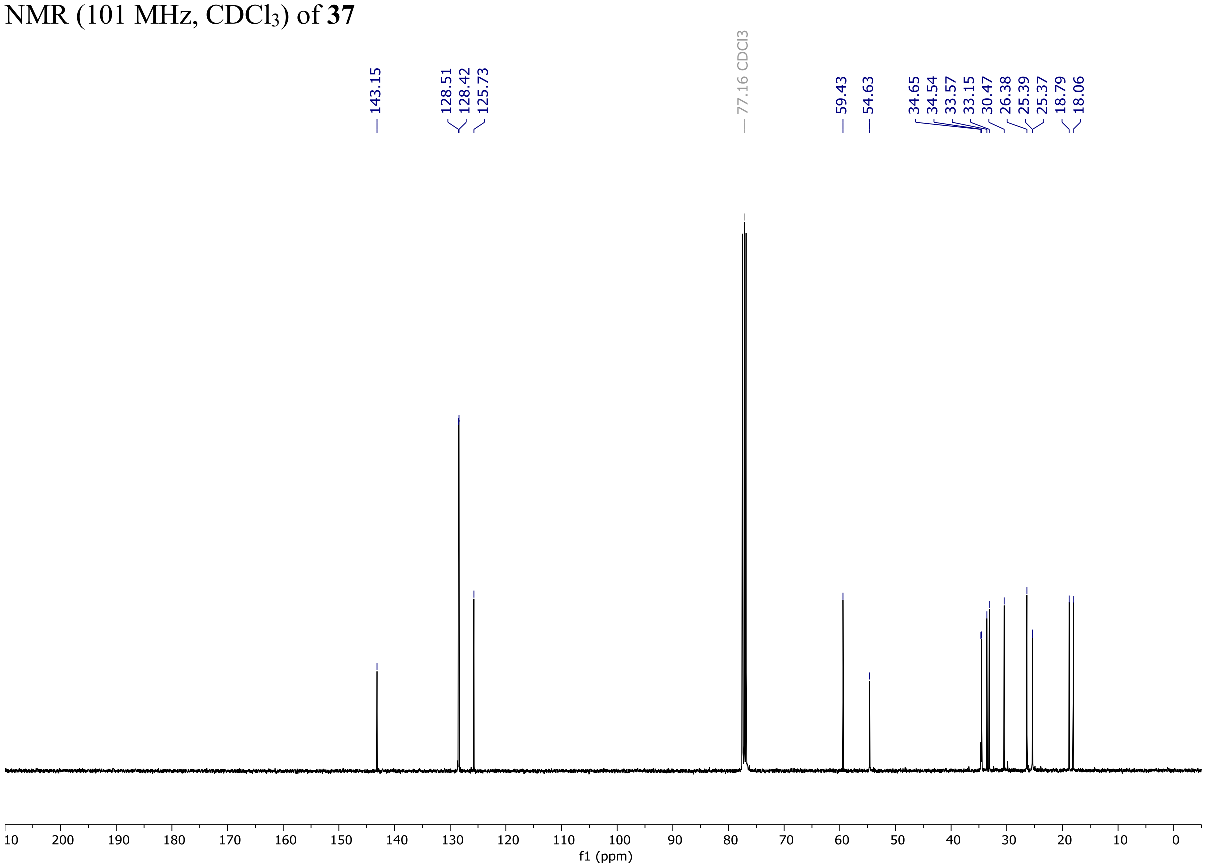

^{1}H NMR (400 MHz, $CDCl_3$) of **38**

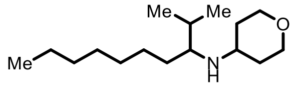

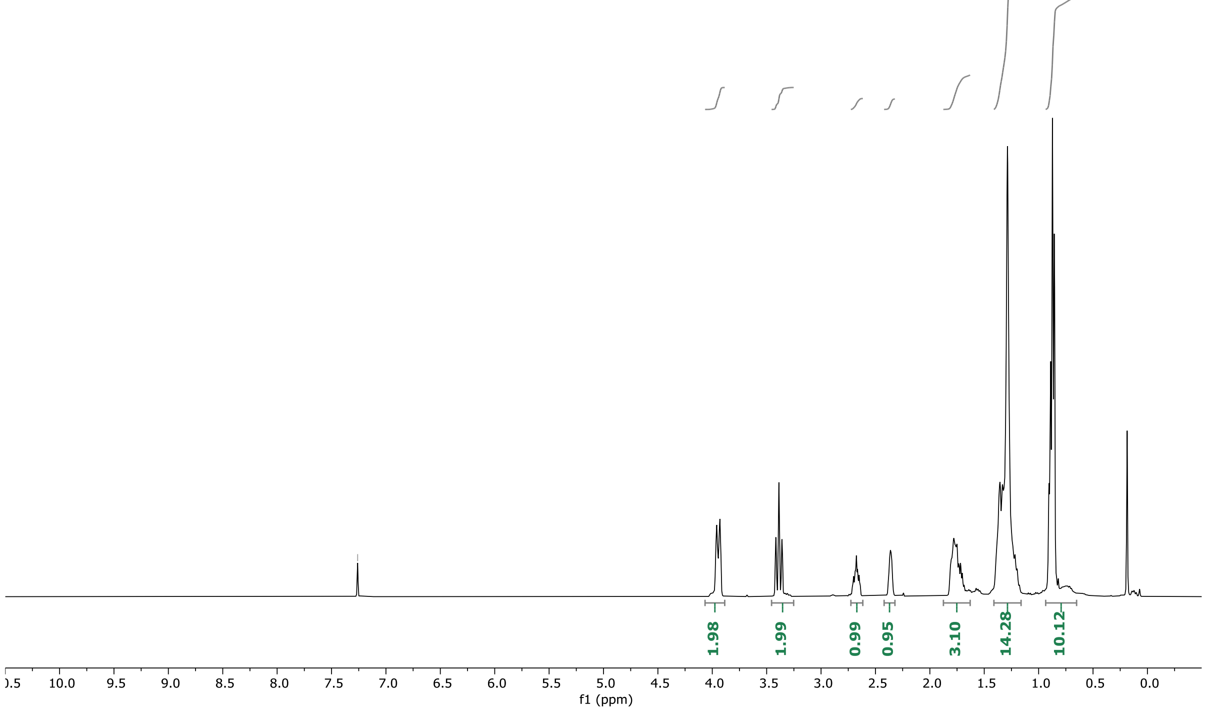

^{13}C NMR (101 MHz, $CDCl_3$) of **38**

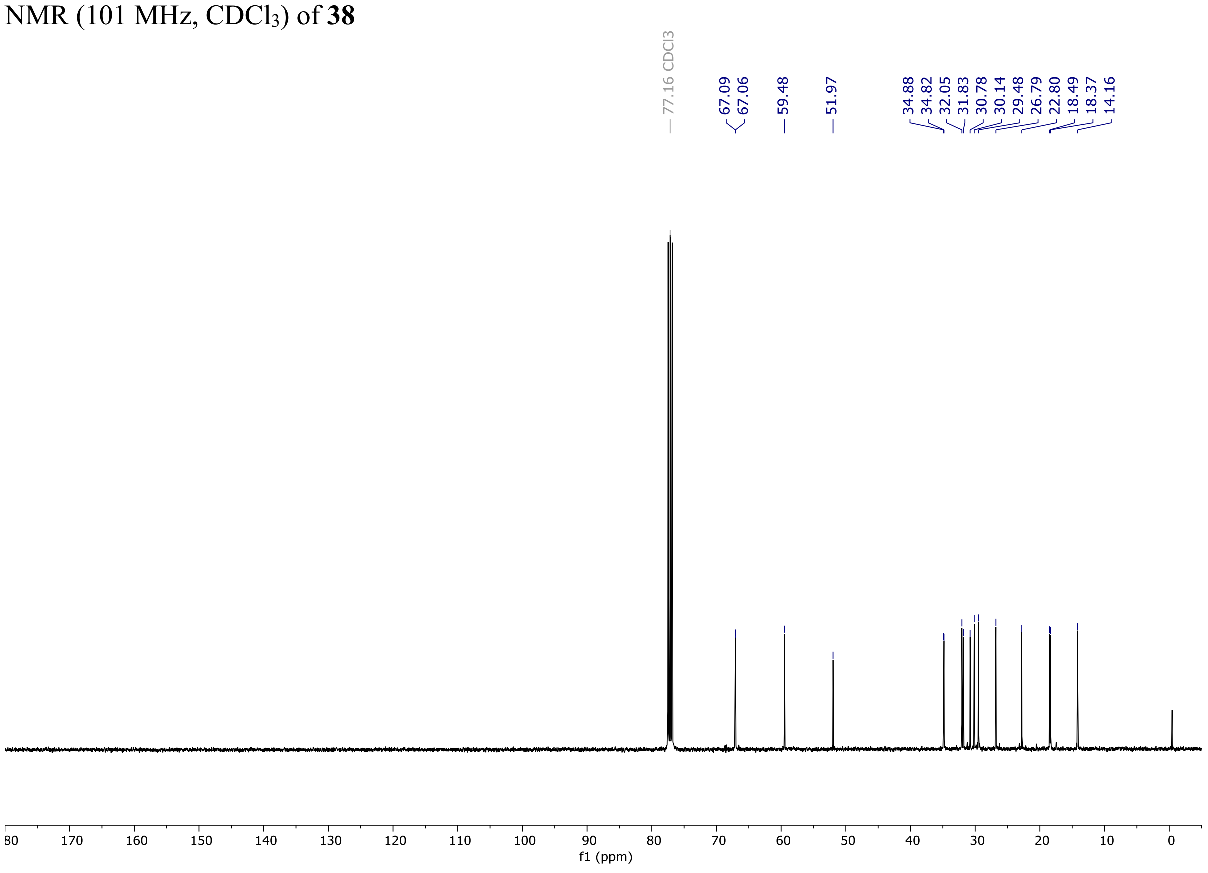

^{1}H NMR (400 MHz, $CDCl_3$) of **39**

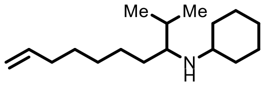

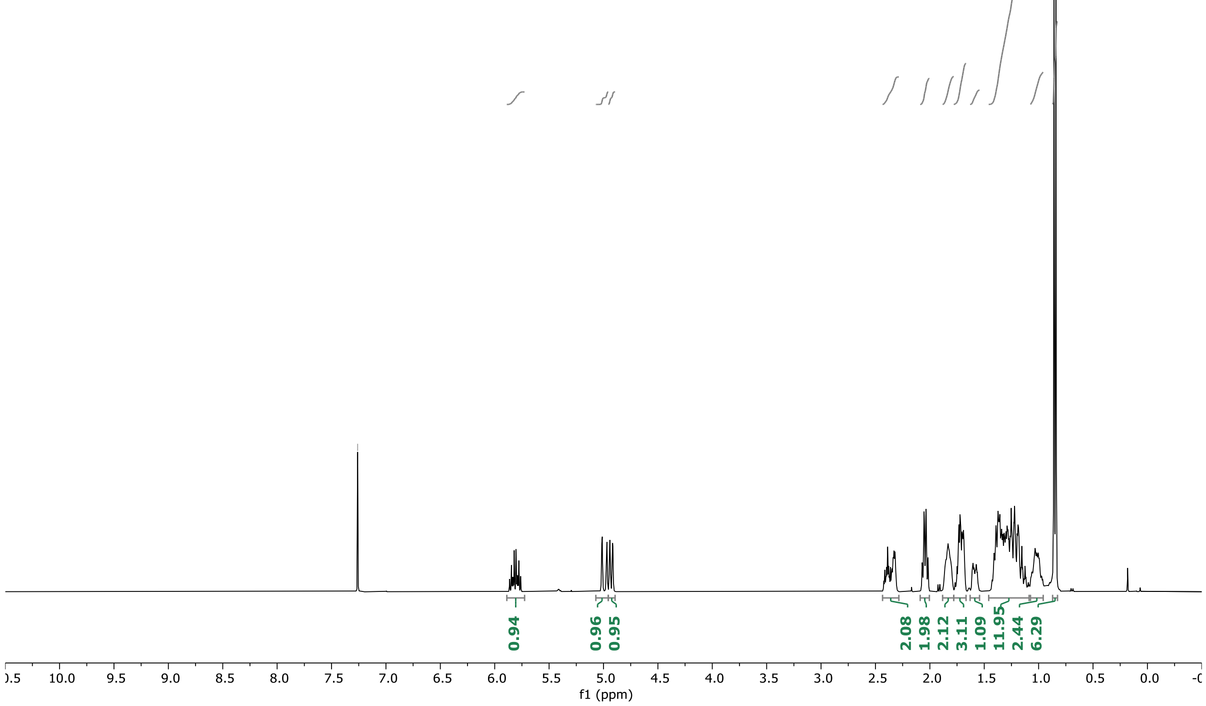

^{13}C NMR (101 MHz, $CDCl_3$) of **39**

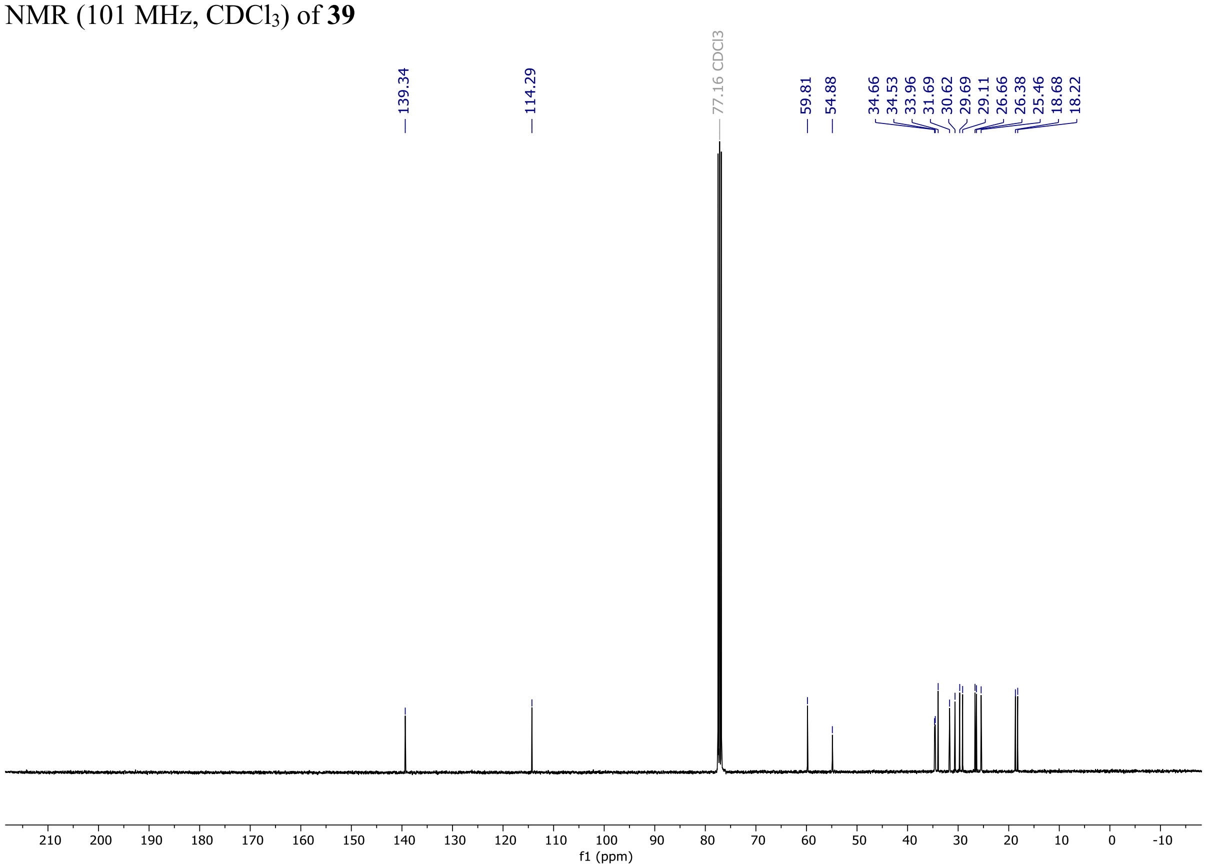

^{1}H NMR (400 MHz, $CDCl_3$) of **40** (* = 15% impurity)

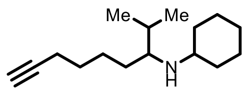

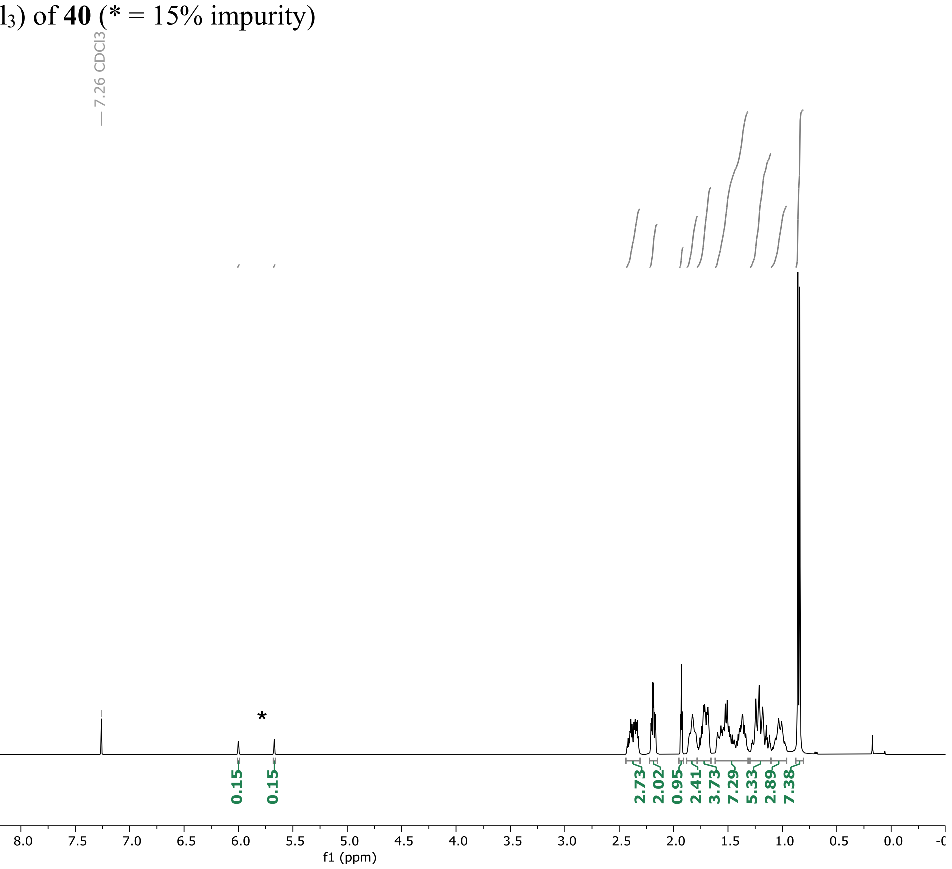

^{13}C NMR (101 MHz, $CDCl_3$) of **40**

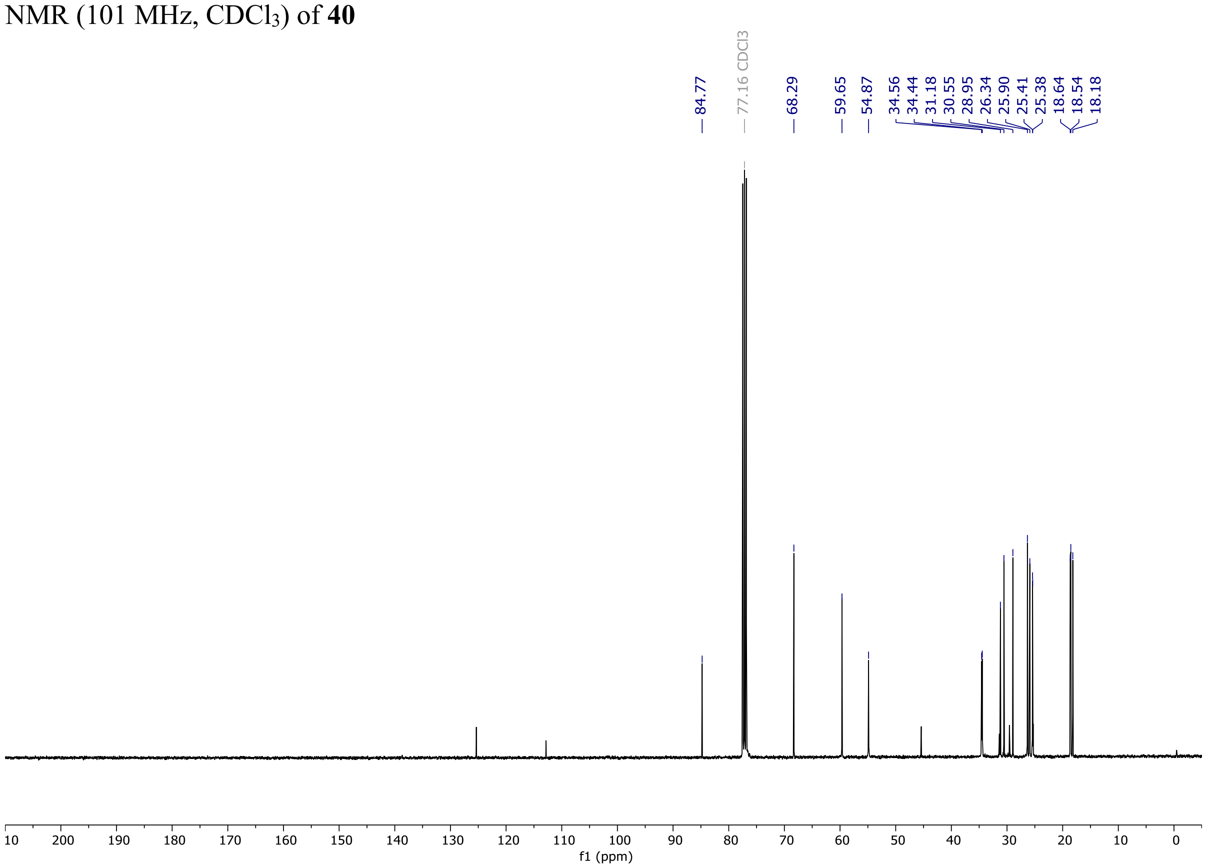

^{1}H NMR (400 MHz, $CDCl_3$) of **41**

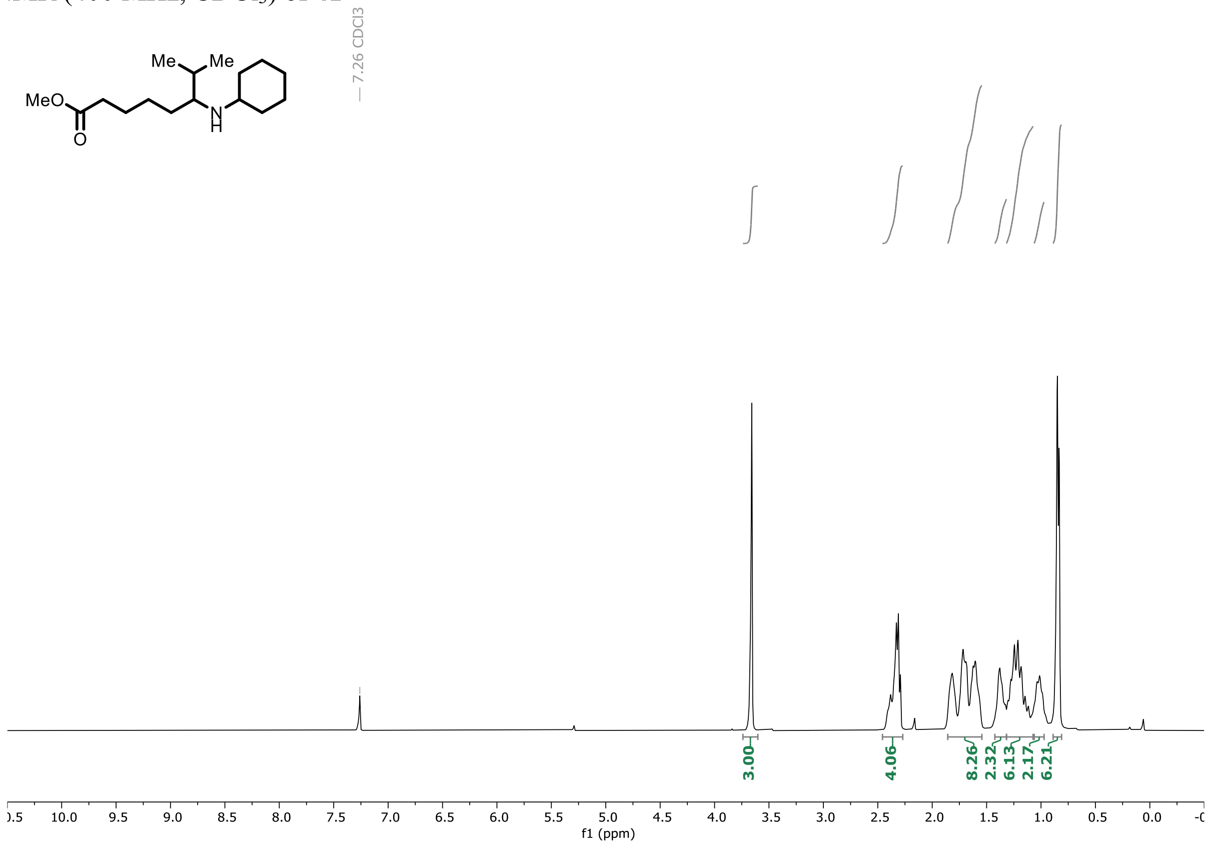

^{13}C NMR (101 MHz, $CDCl_3$) of **41**

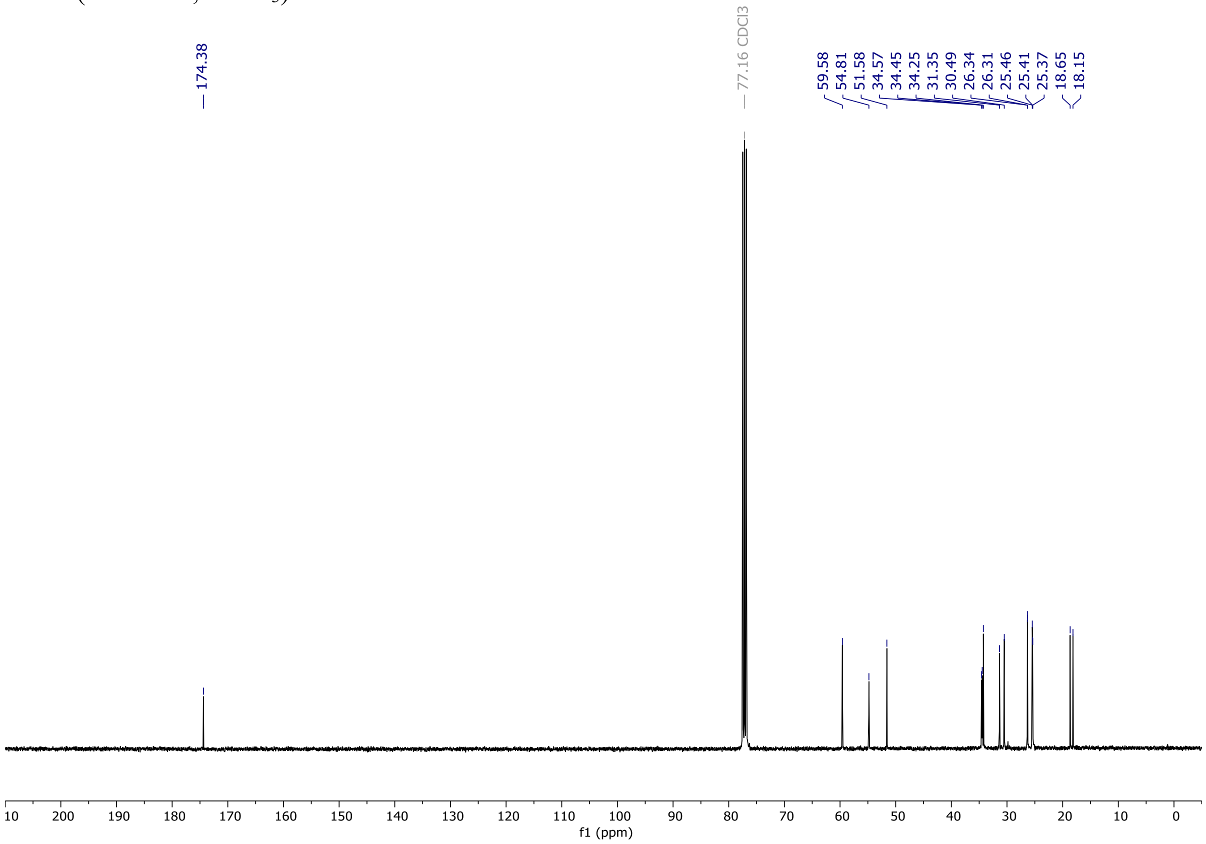

^{1}H NMR (400 MHz, $CDCl_3$) of **43**

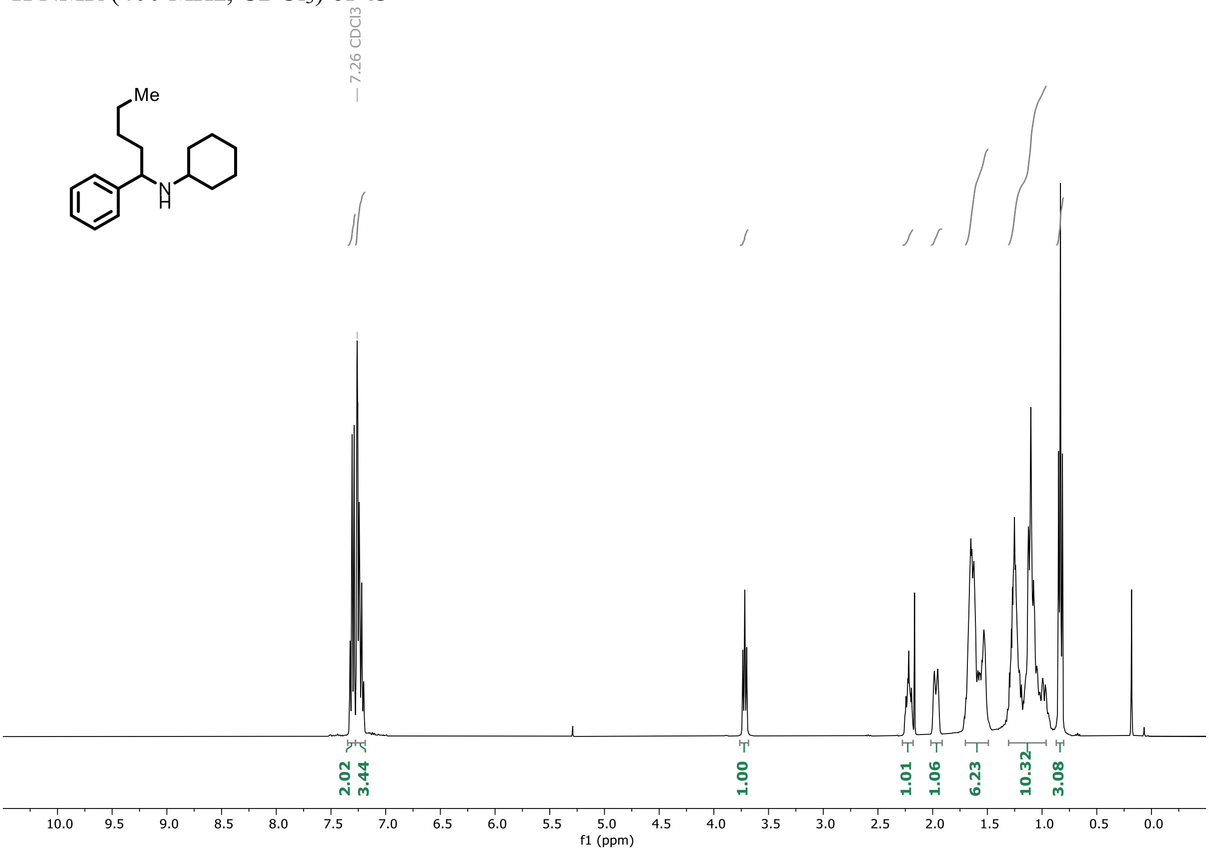

^{13}C NMR (101 MHz, $CDCl_3$) of **43**

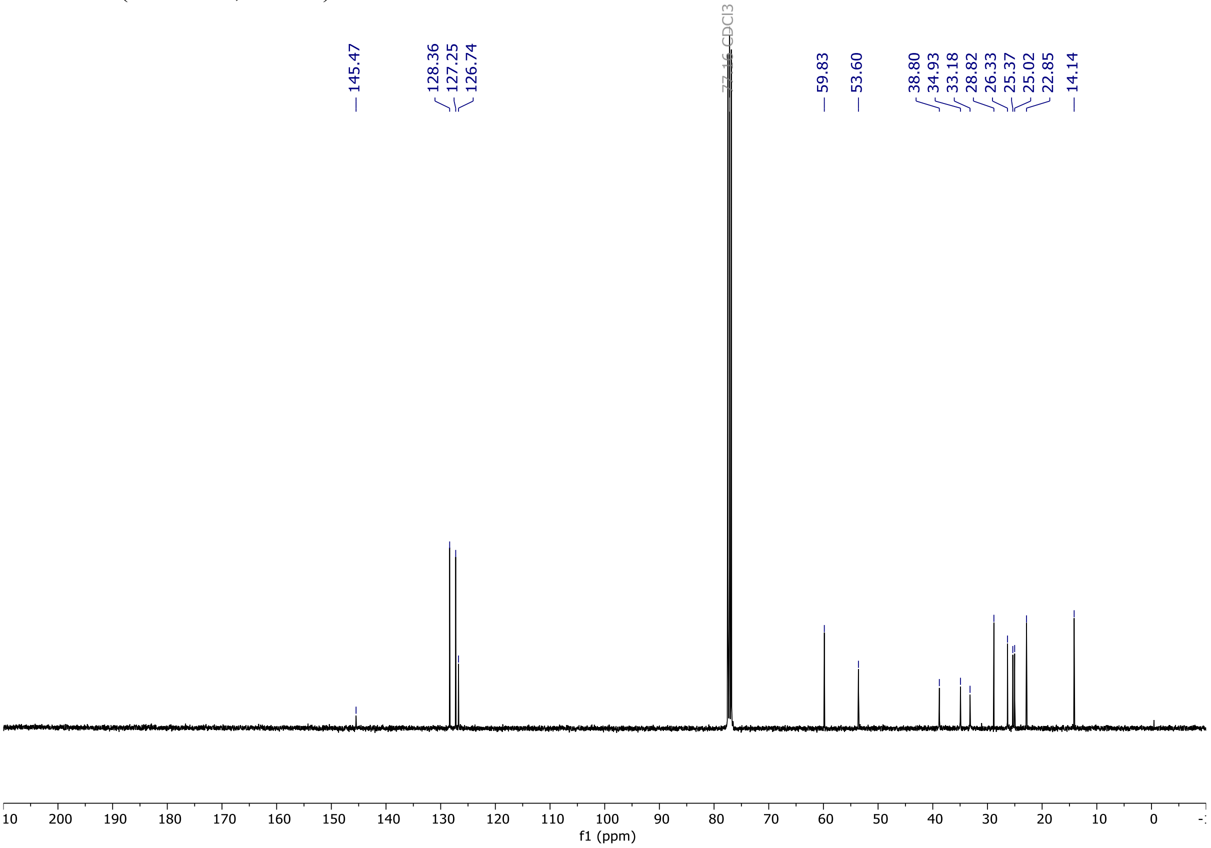

^{1}H NMR (400 MHz, $CDCl_3$) of **44**

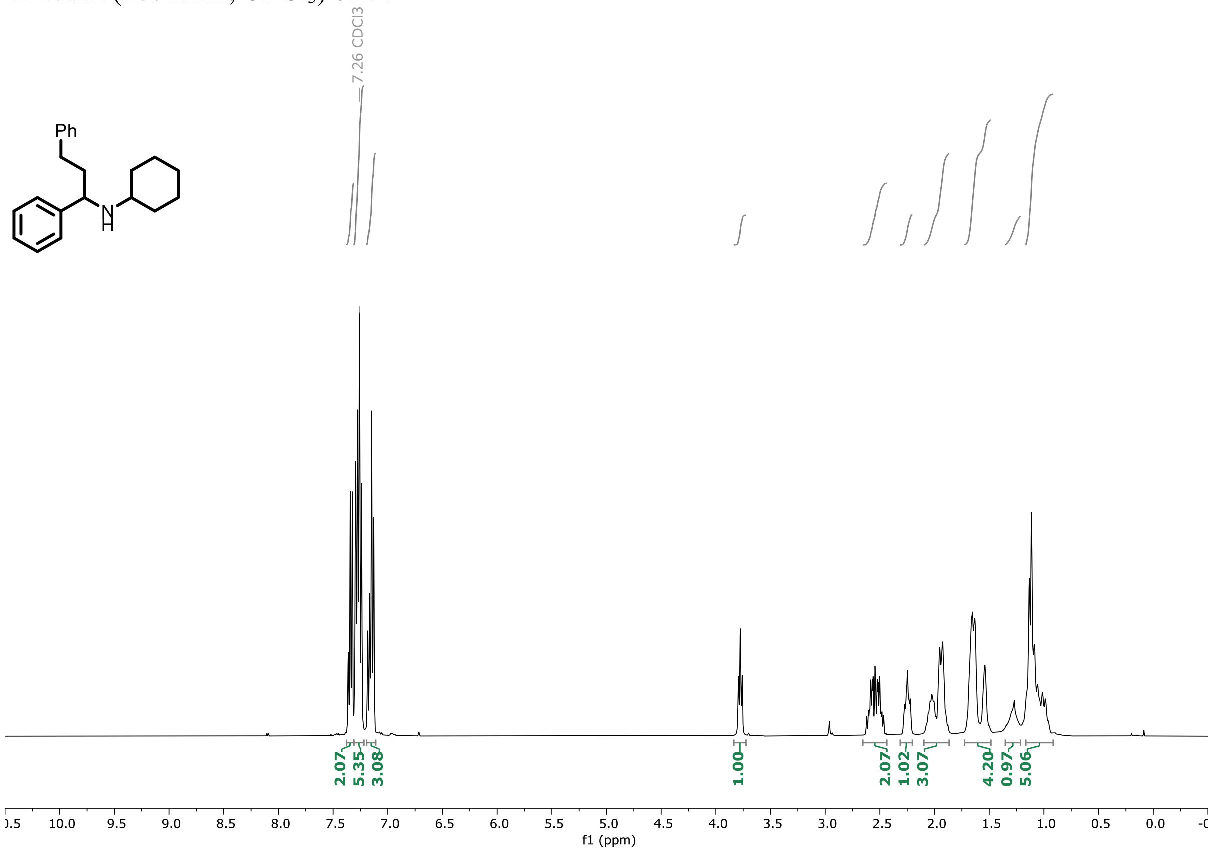

^{13}C NMR (101 MHz, $CDCl_3$) of **44**

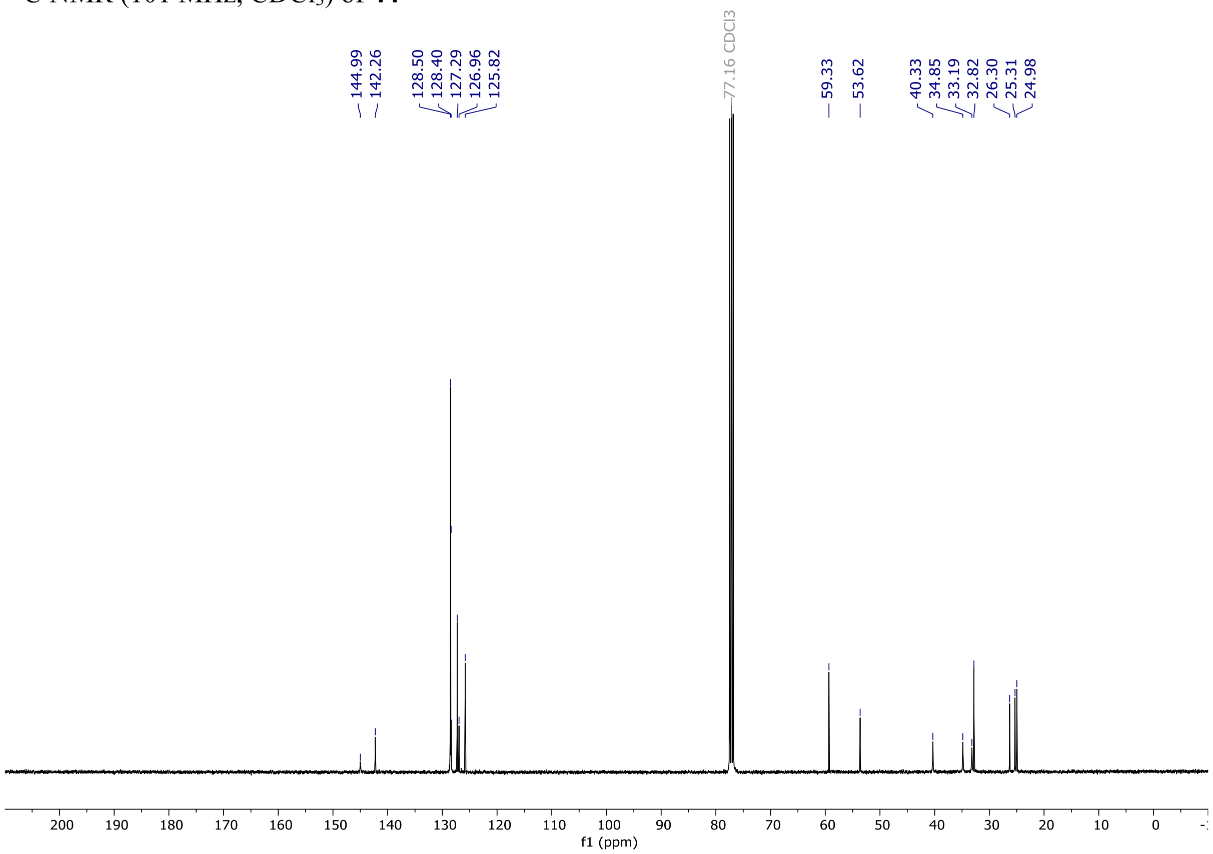

^{1}H NMR (400 MHz, $CDCl_3$) of **45**

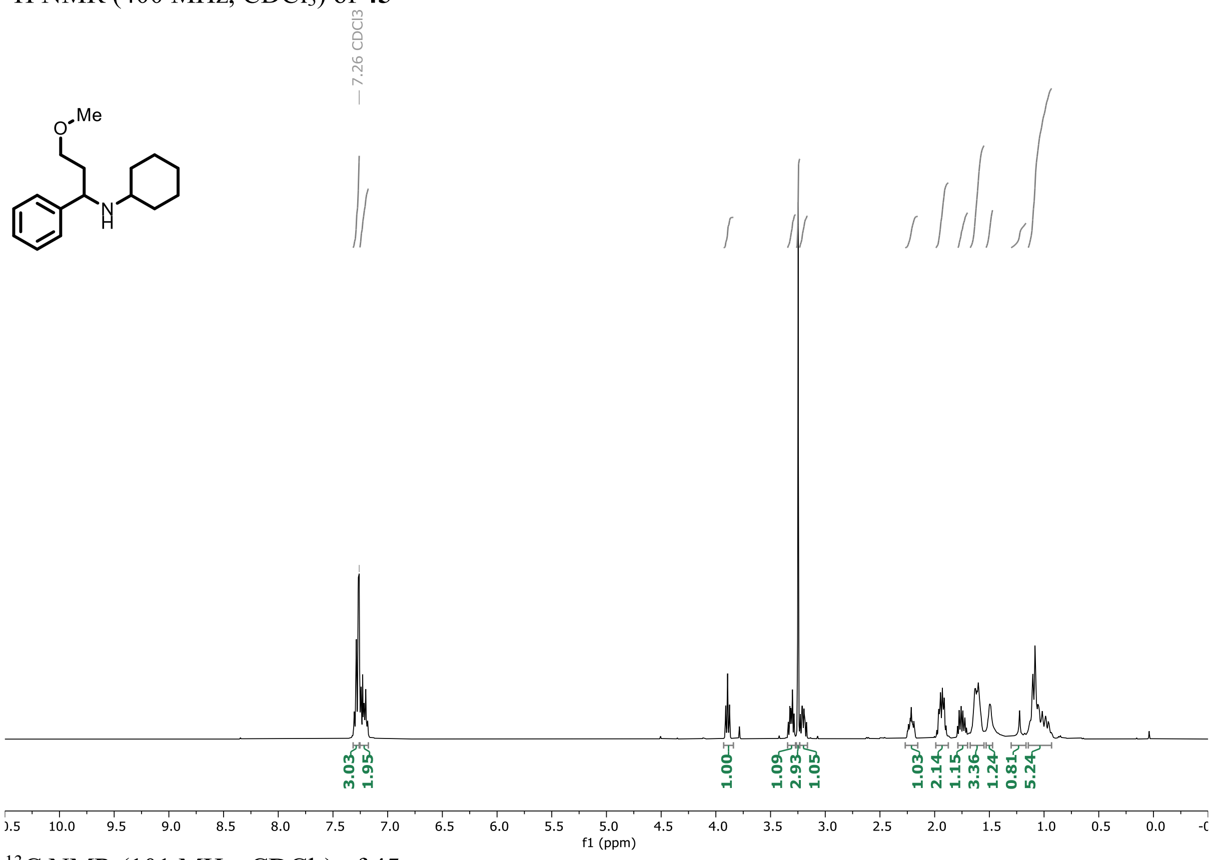

^{13}C NMR (101 MHz, $CDCl_3$) of **45**

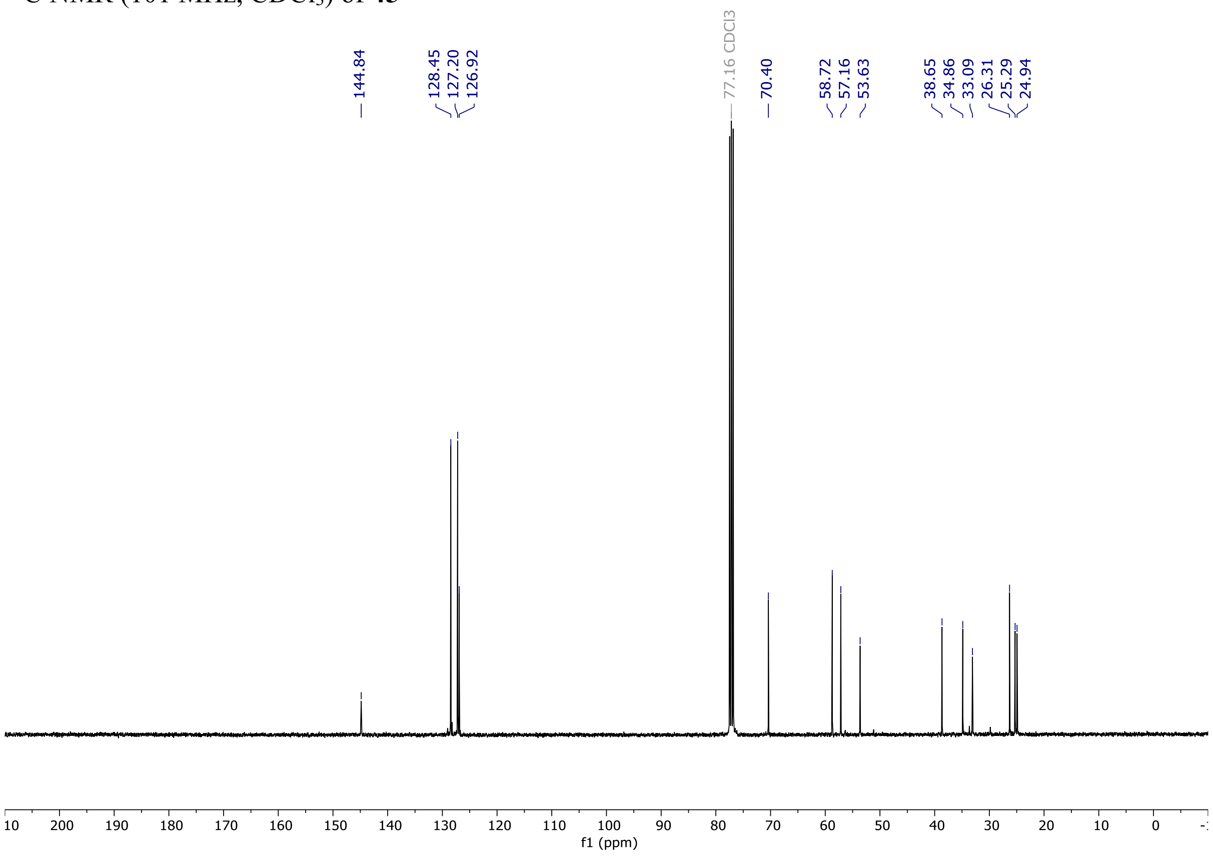

^{1}H NMR (400 MHz, $CDCl_3$) of **46**

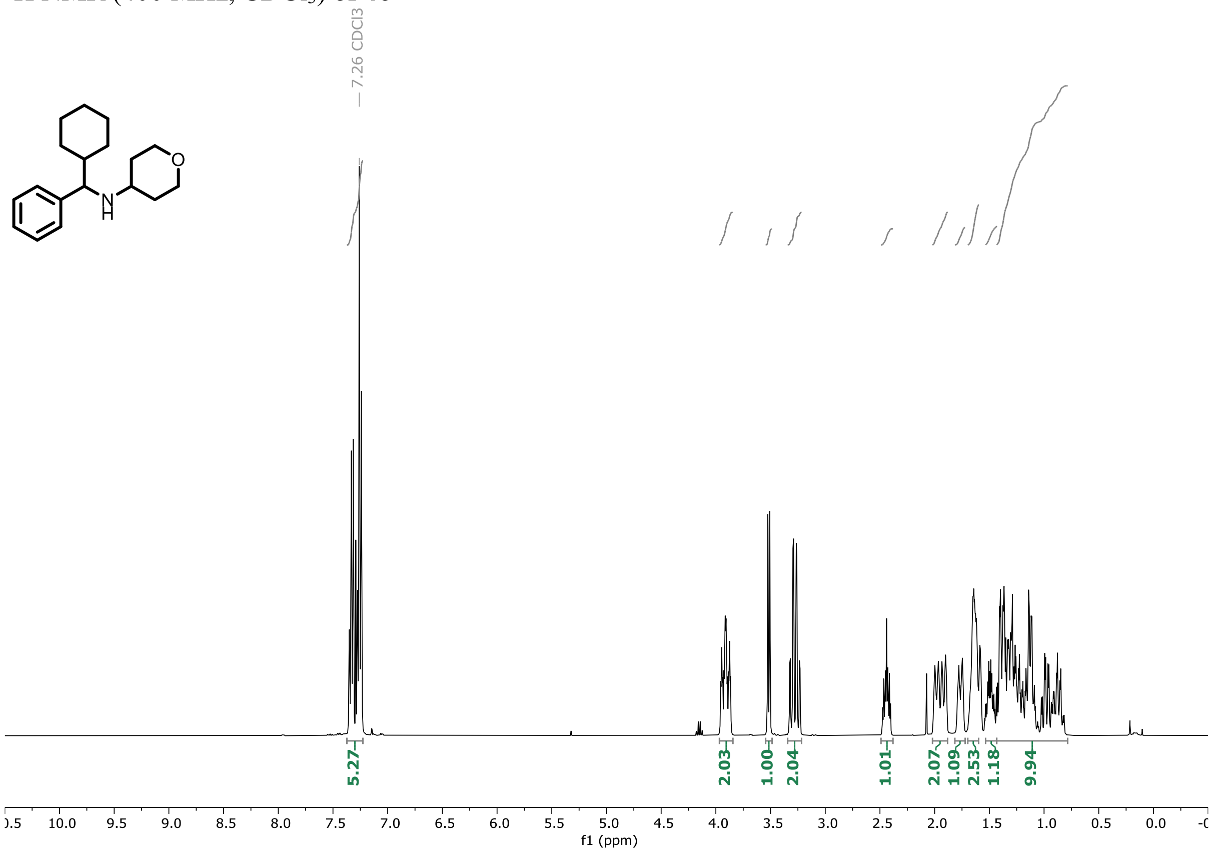

^{13}C NMR (101 MHz, $CDCl_3$) of **46**

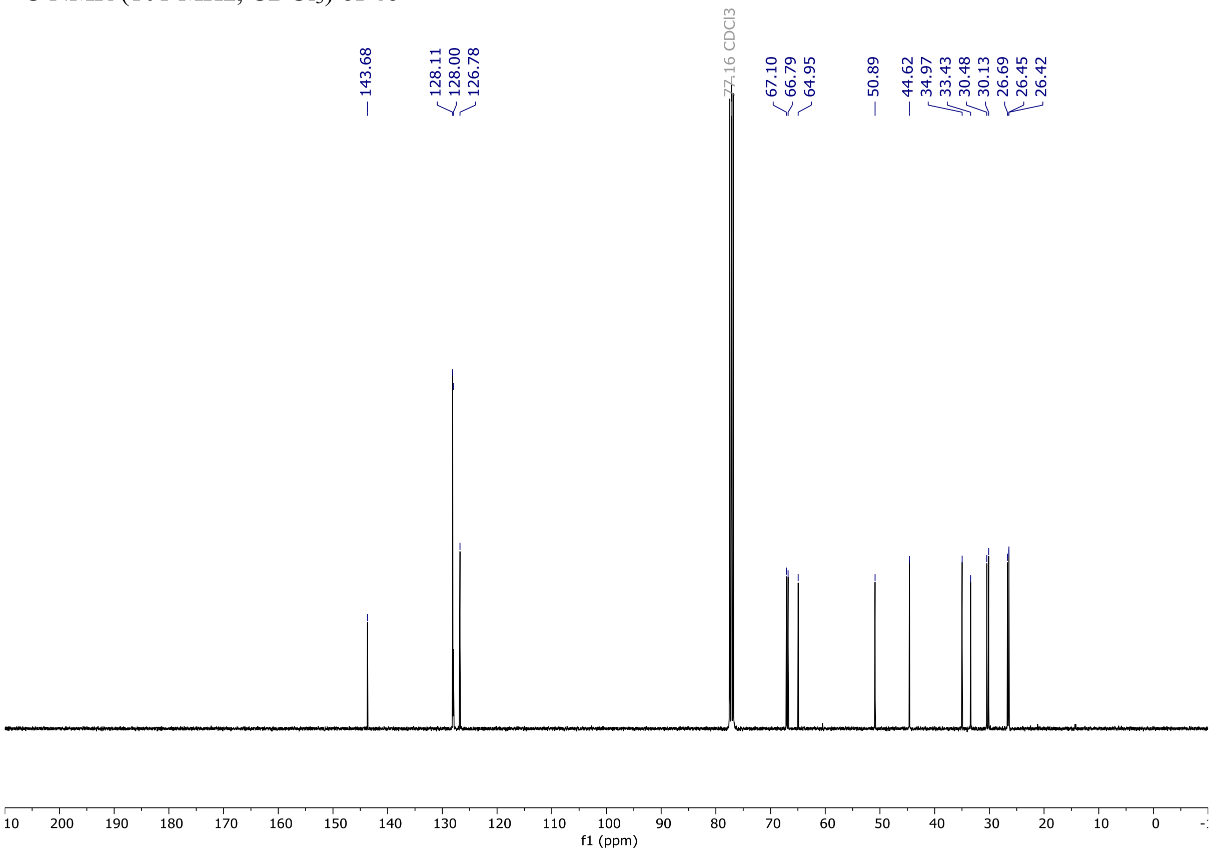

^{1}H NMR (400 MHz, $CDCl_3$) of **47**

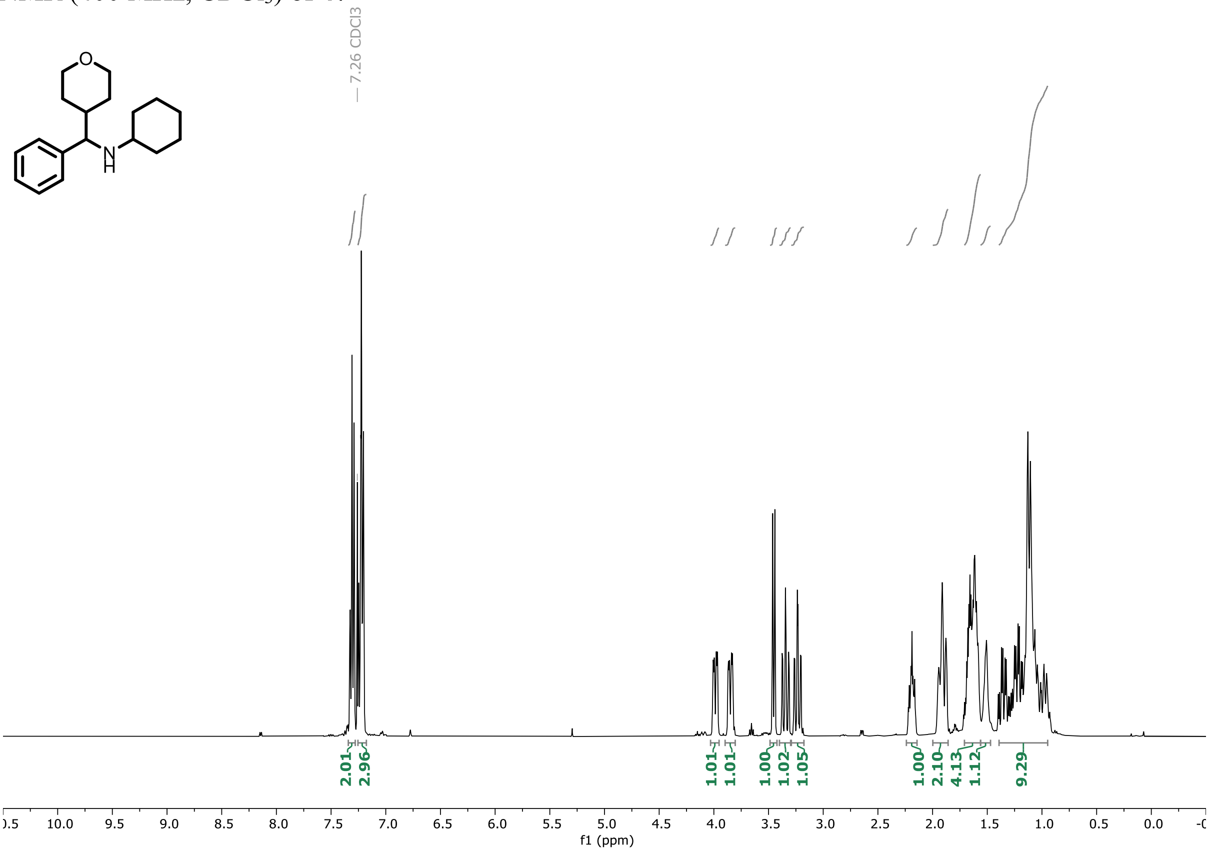

^{13}C NMR (101 MHz, $CDCl_3$) of **47**

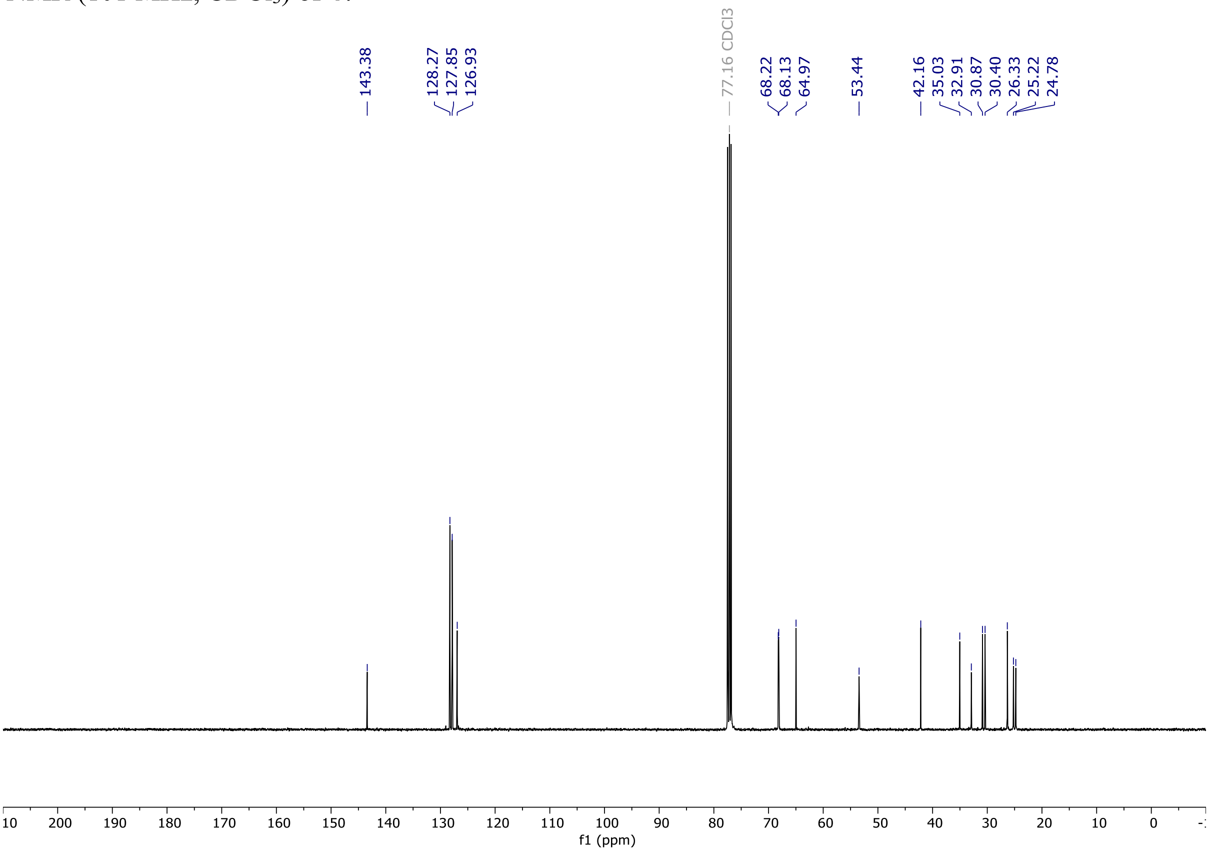

^{1}H NMR (400 MHz, $CDCl_3$) of **48**

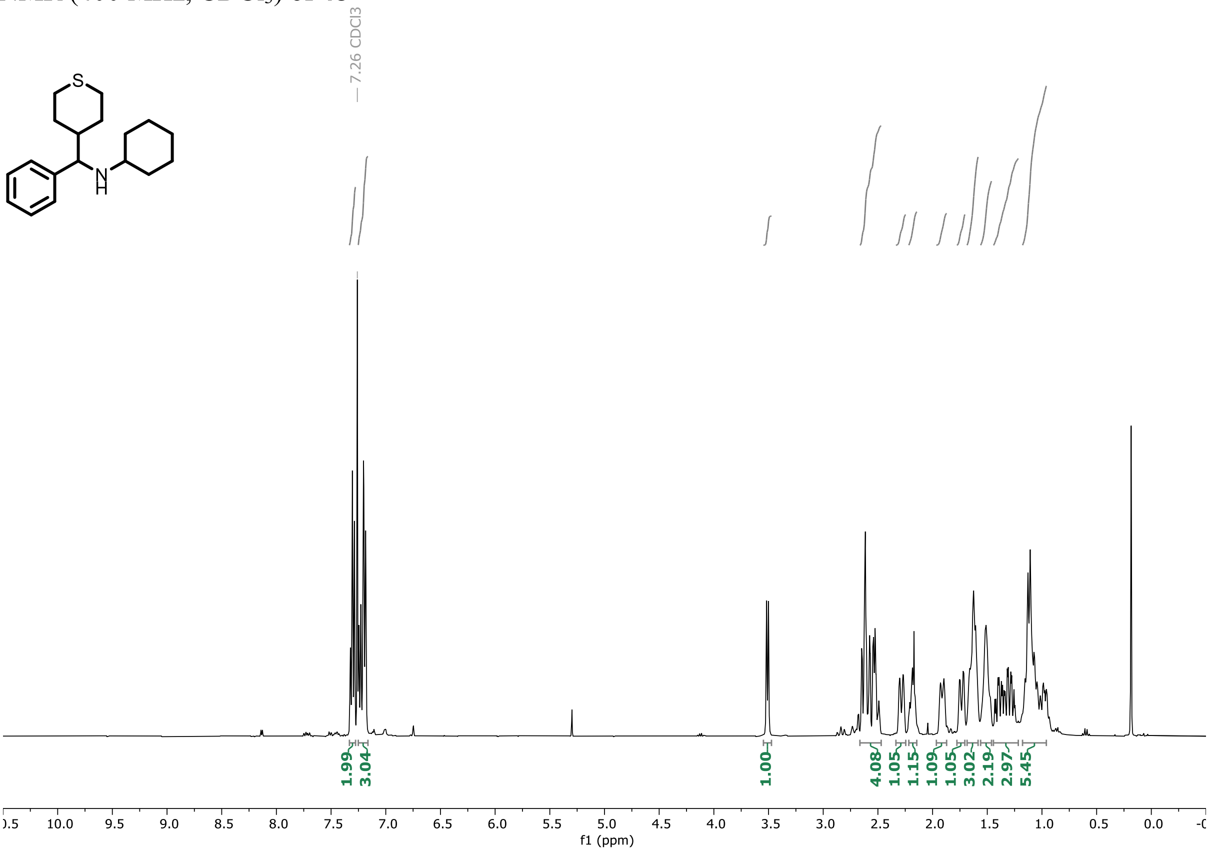

^{13}C NMR (101 MHz, $CDCl_3$) of **48**

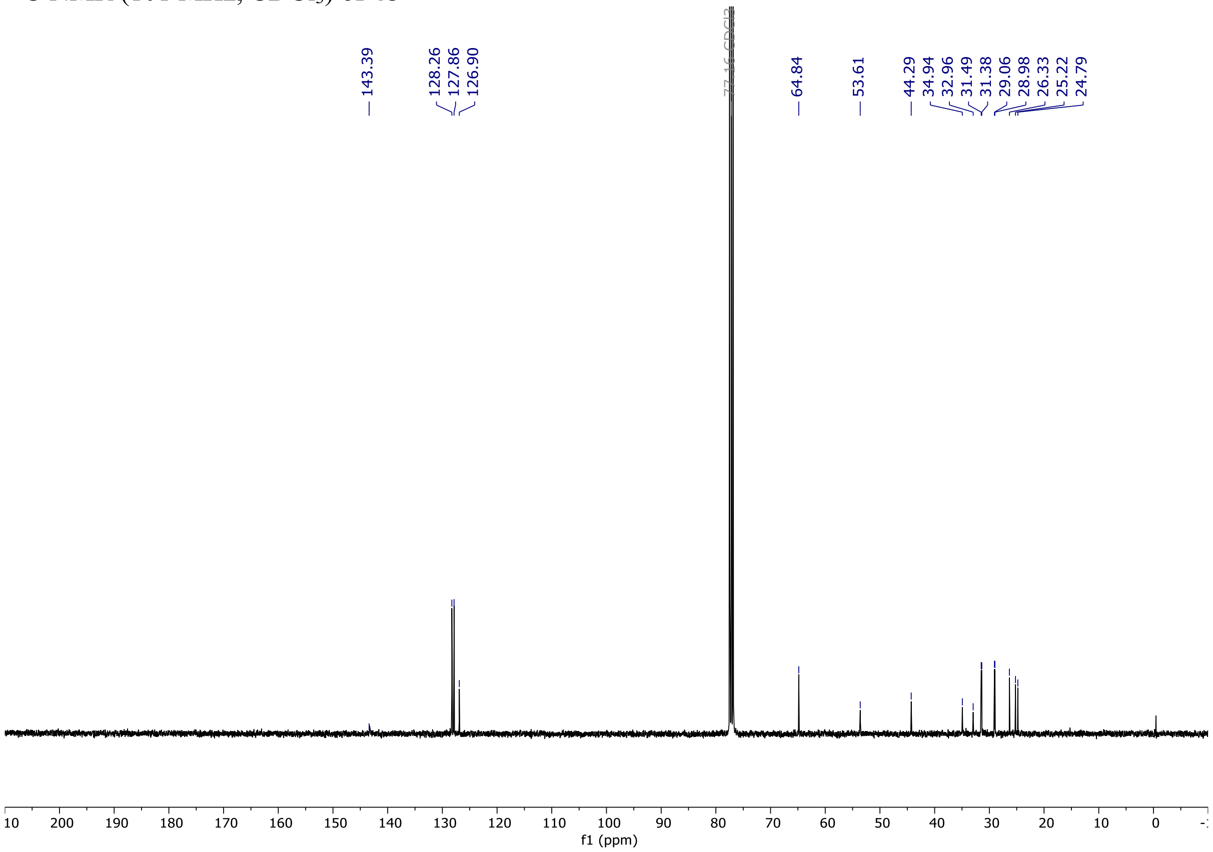

^{1}H NMR (400 MHz, $CDCl_3$) of **49**

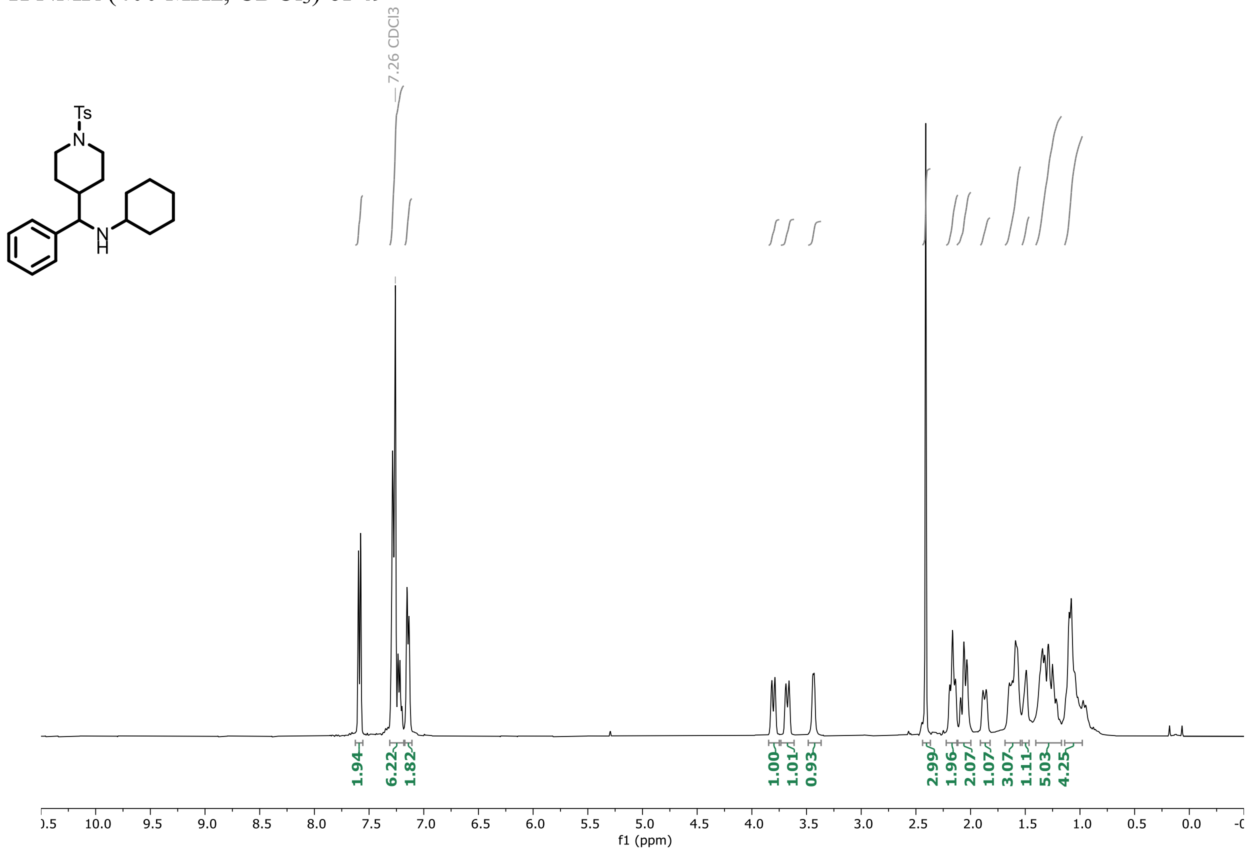

^{13}C NMR (101 MHz, $CDCl_3$) of **49**

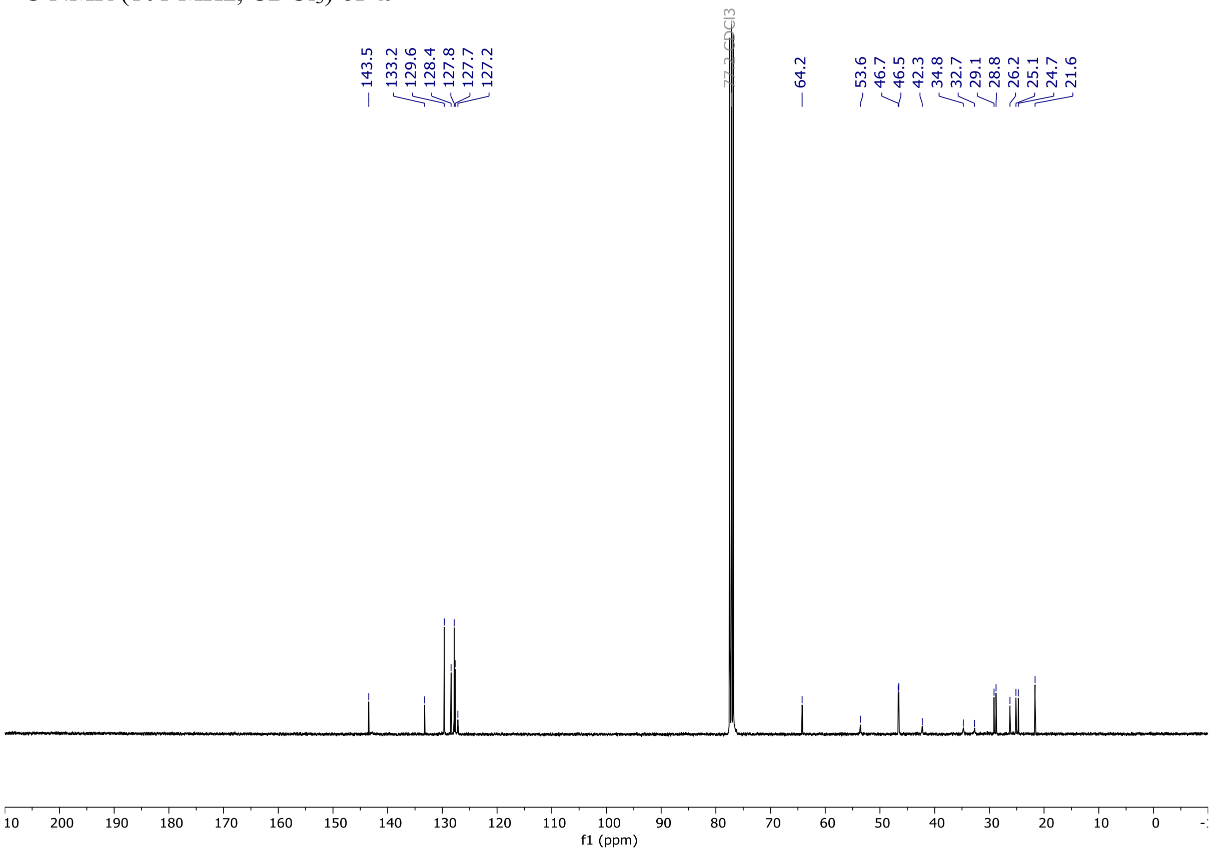

^{1}H NMR (400 MHz, $CDCl_3$) of **50**

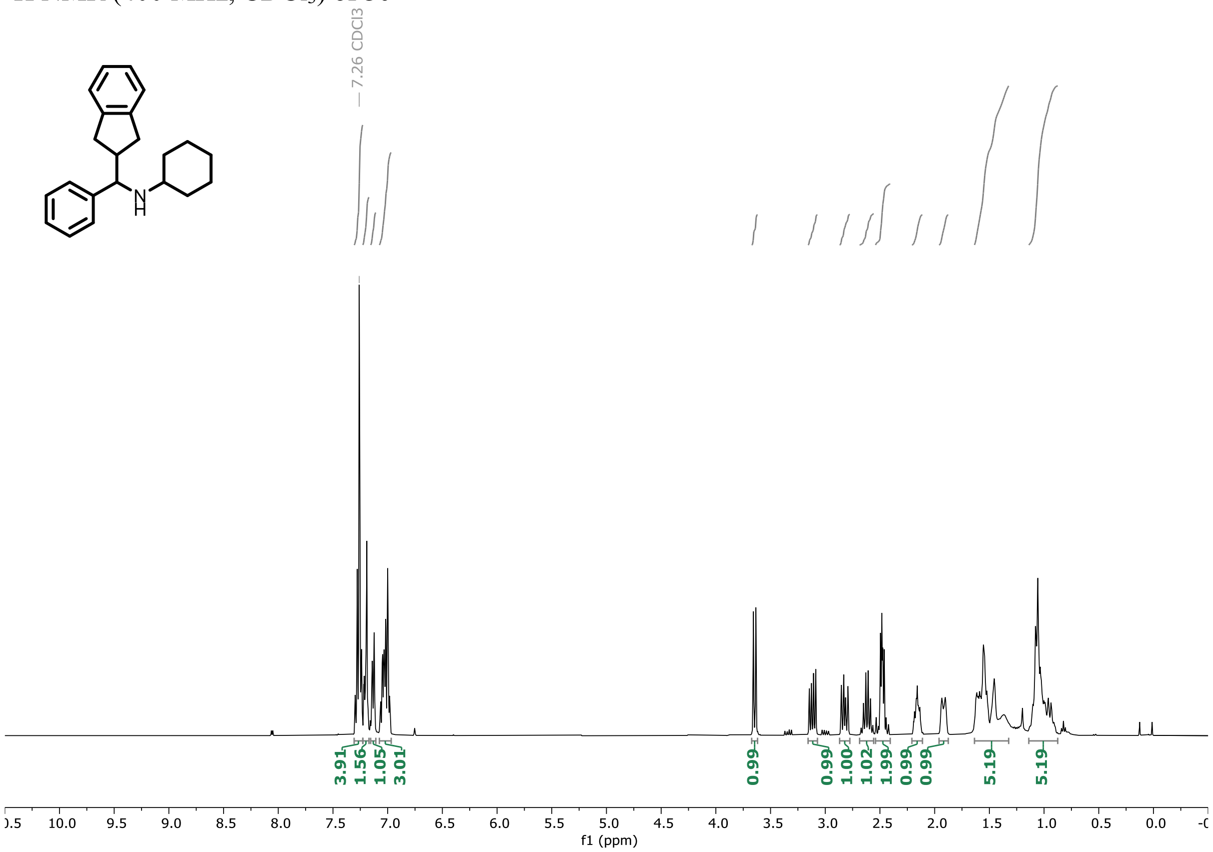

^{13}C NMR (101 MHz, $CDCl_3$) of **50**

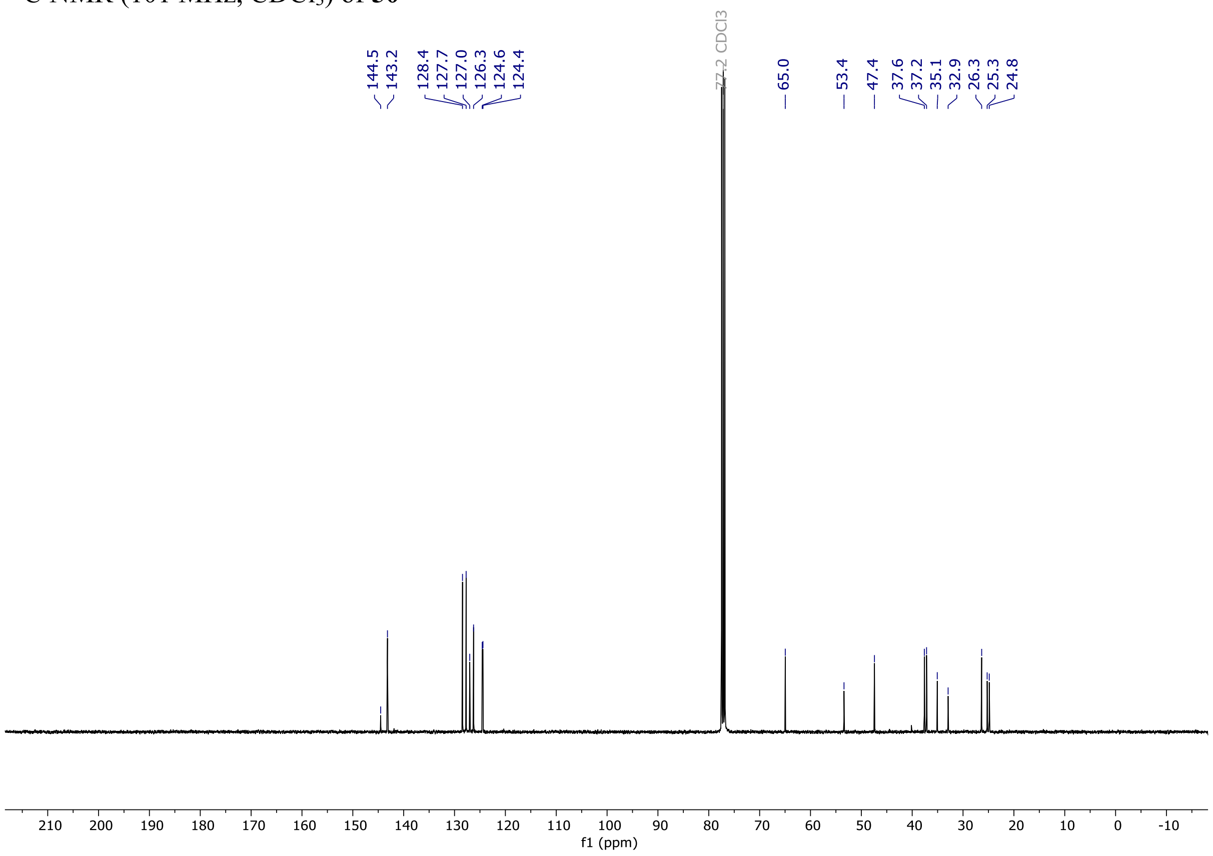

^{1}H NMR (400 MHz, $CDCl_3$) of **51**

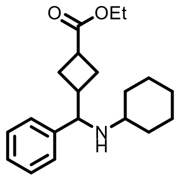

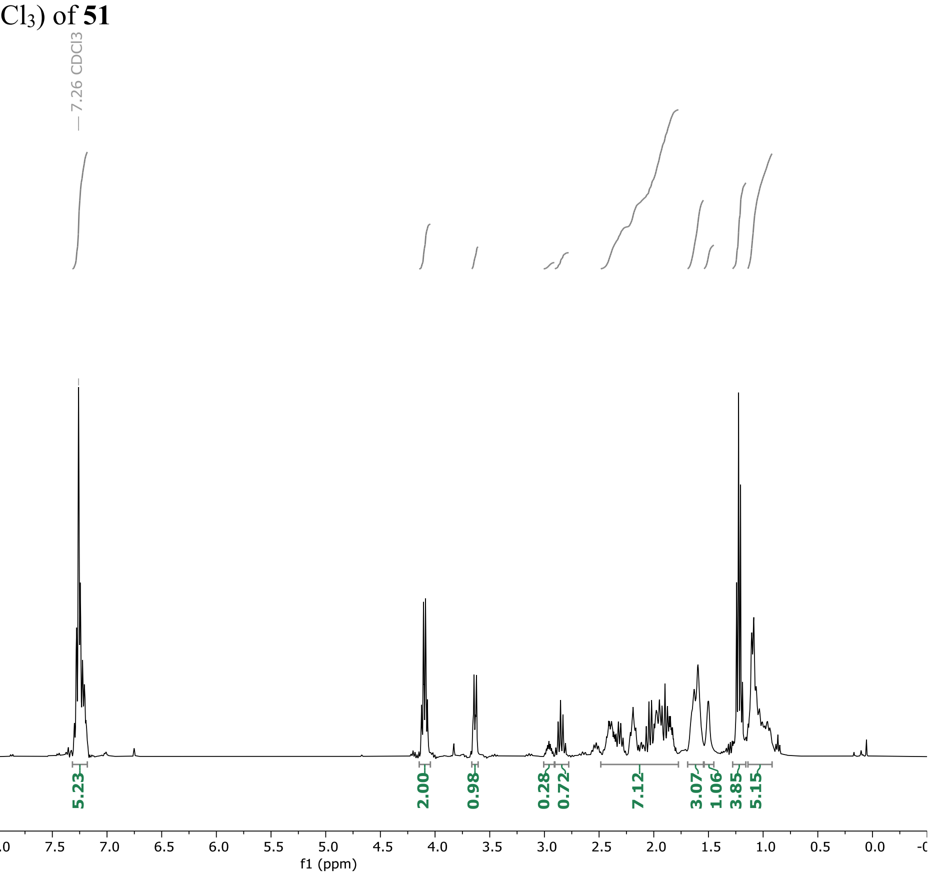

^{13}C NMR (101 MHz, $CDCl_3$) of **51**

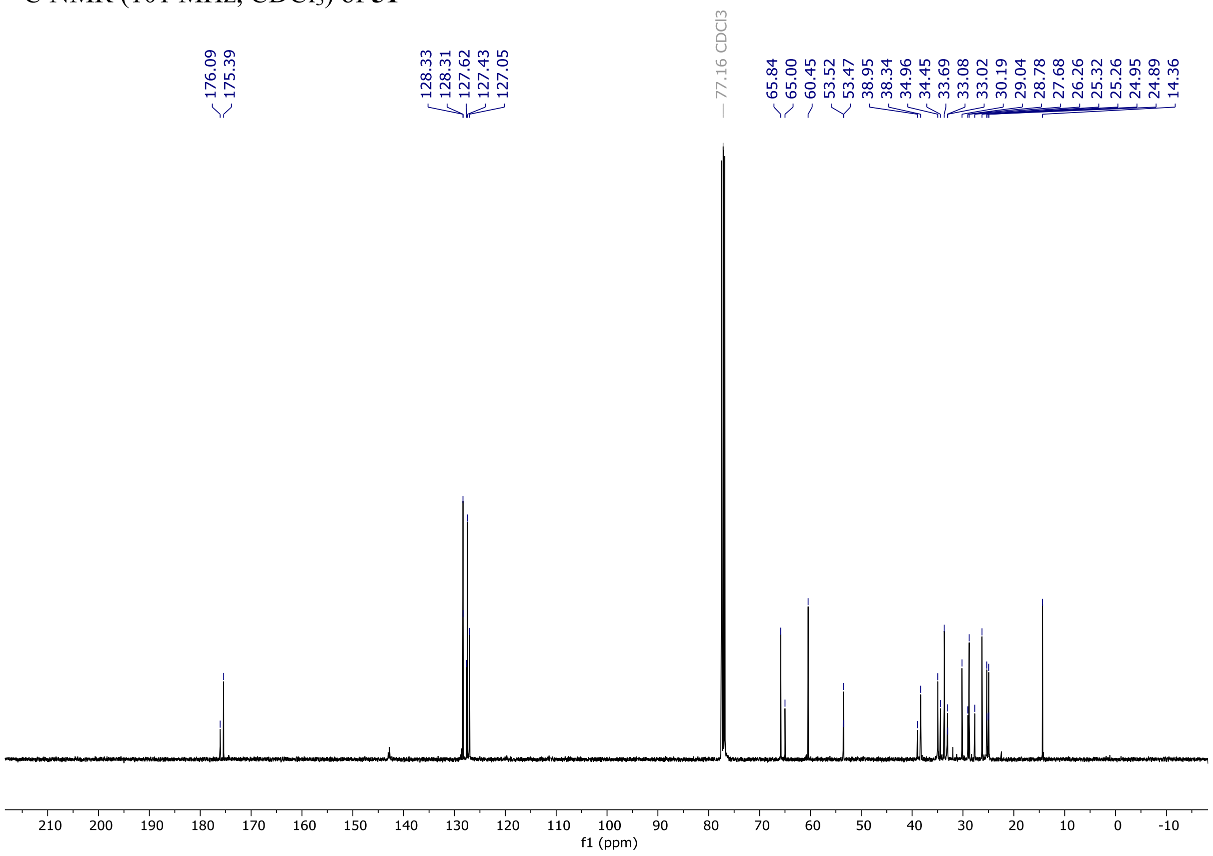

^{1}H NMR (400 MHz, $CDCl_3$) of **52**

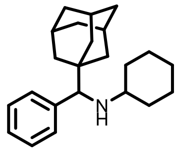

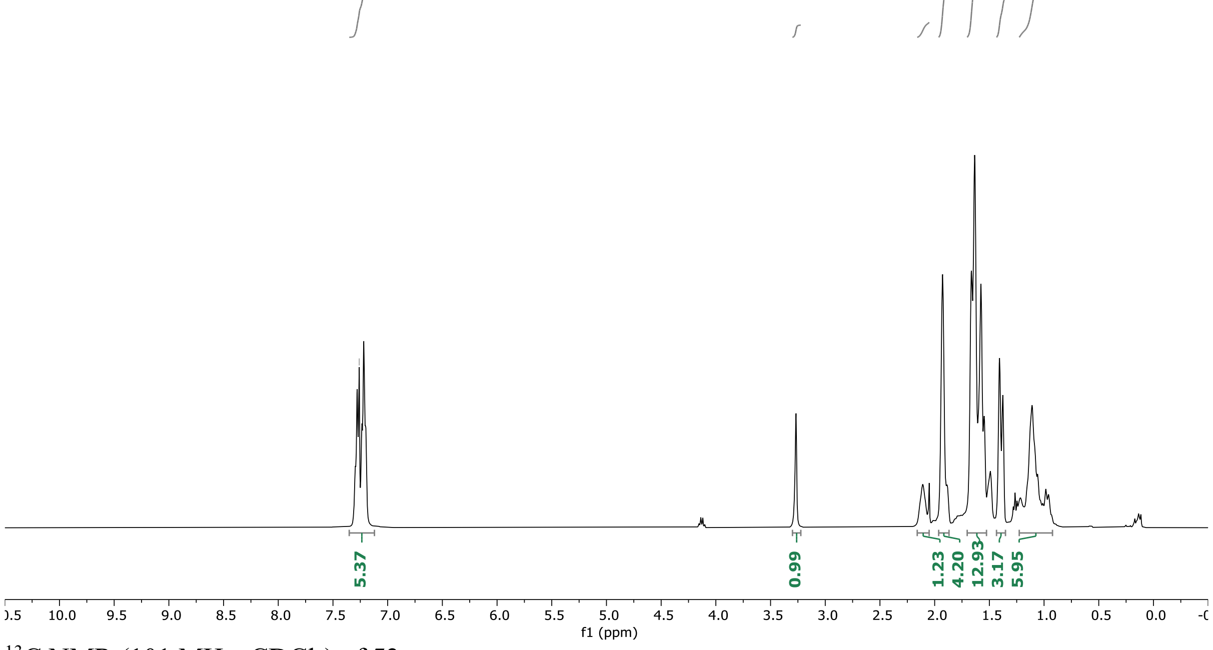

^{13}C NMR (101 MHz, $CDCl_3$) of **52**

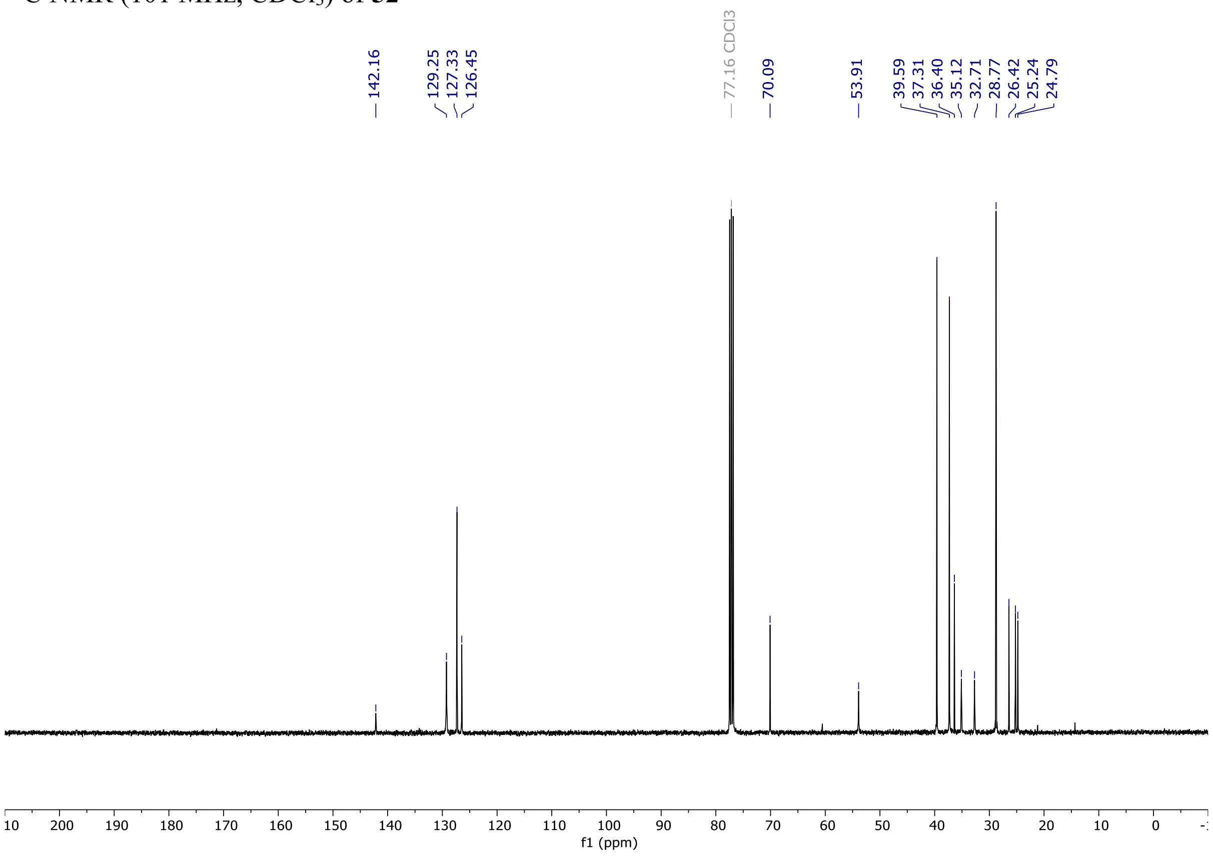

^{1}H NMR (400 MHz, $CDCl_3$) of **53**

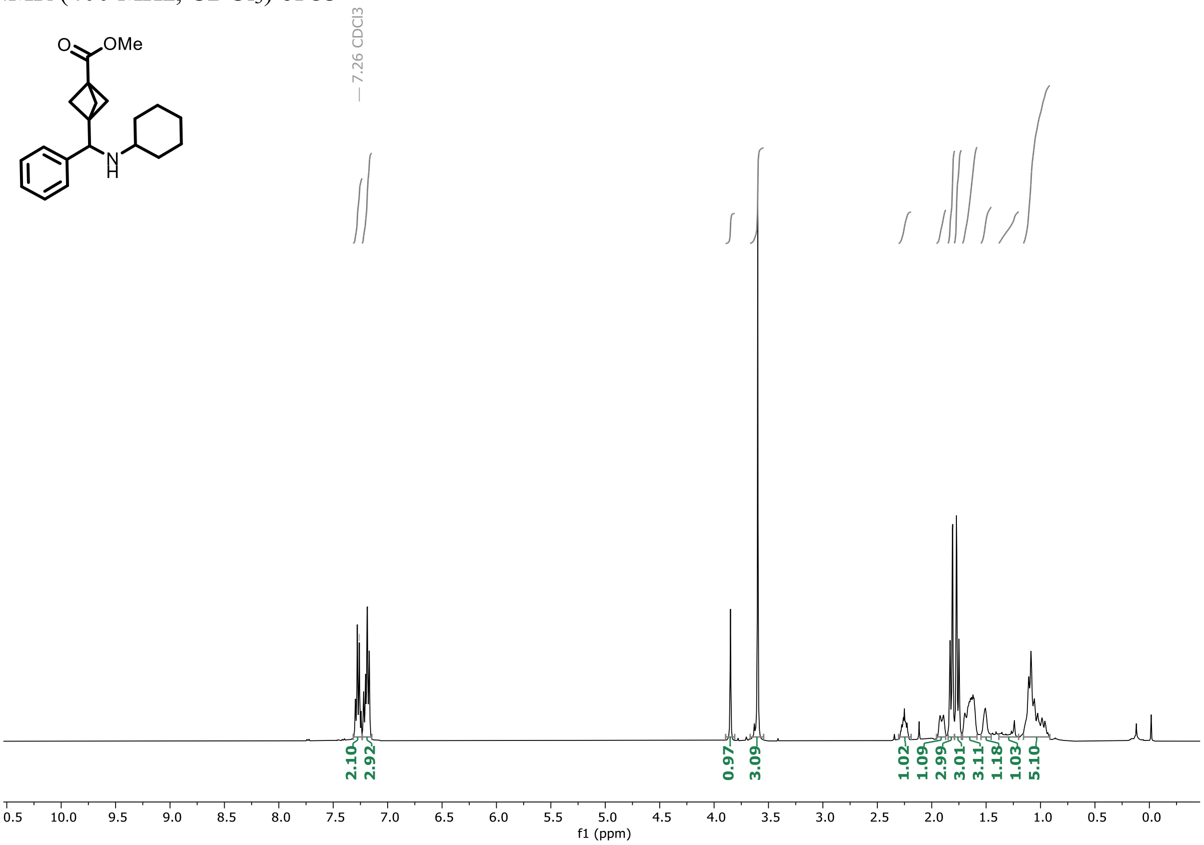

^{13}C NMR (101 MHz, $CDCl_3$) of **53**

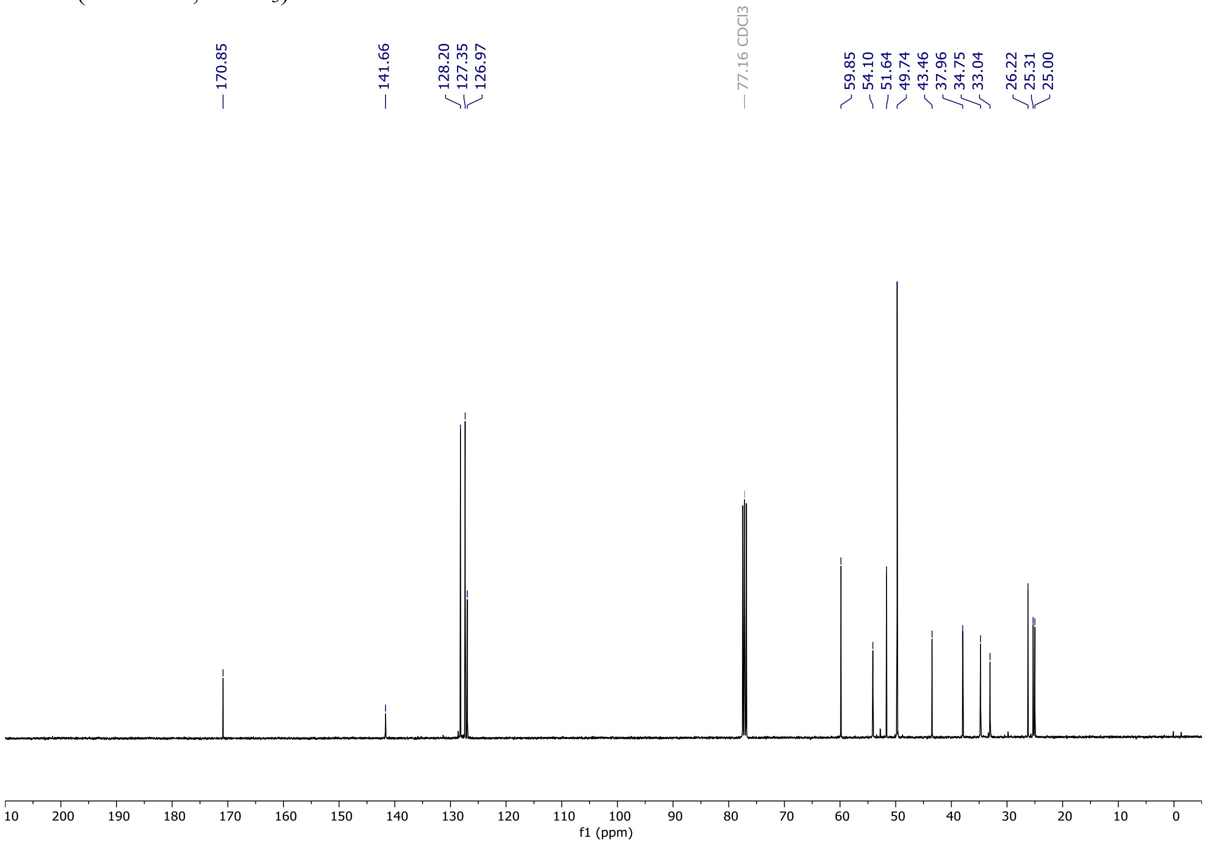

^{1}H NMR (400 MHz, $CDCl_3$) of **54**

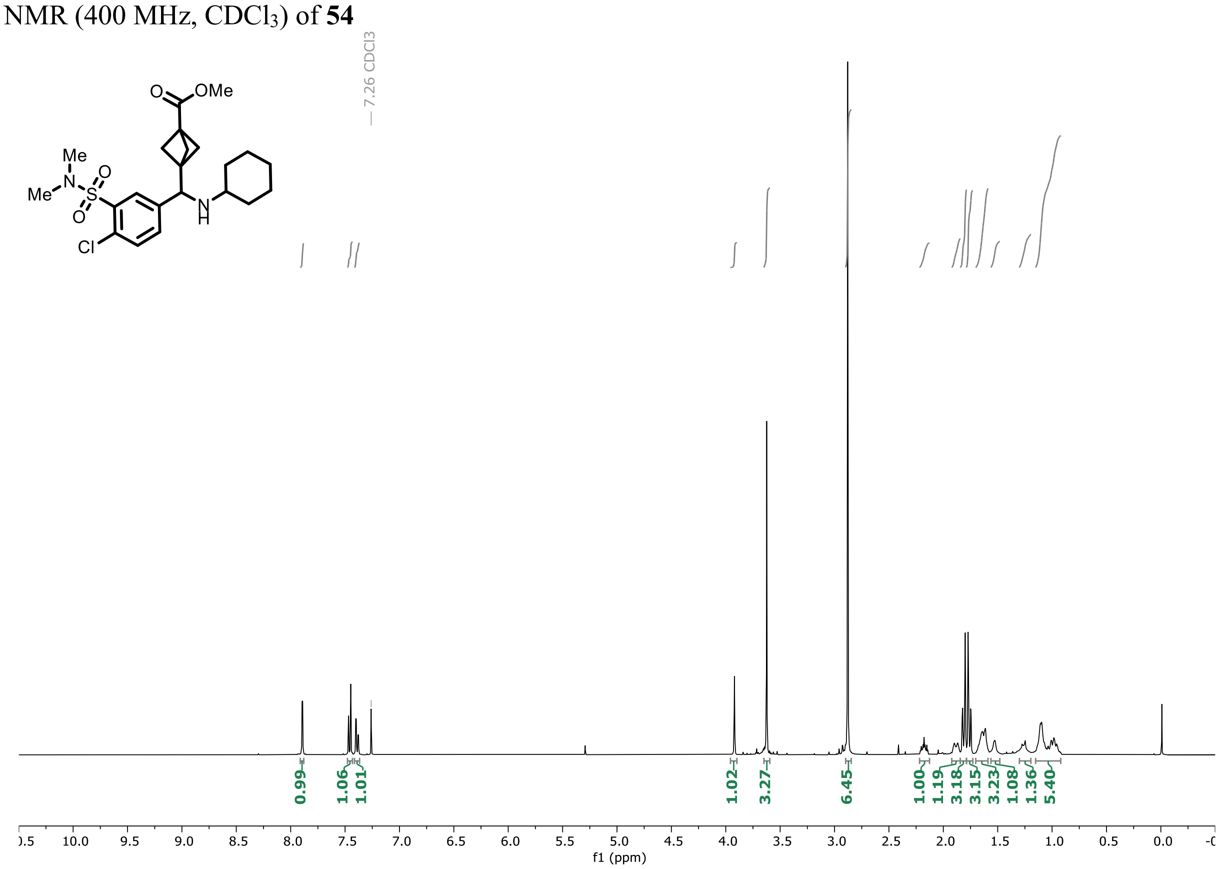

^{13}C NMR (101 MHz, $CDCl_3$) of **54**

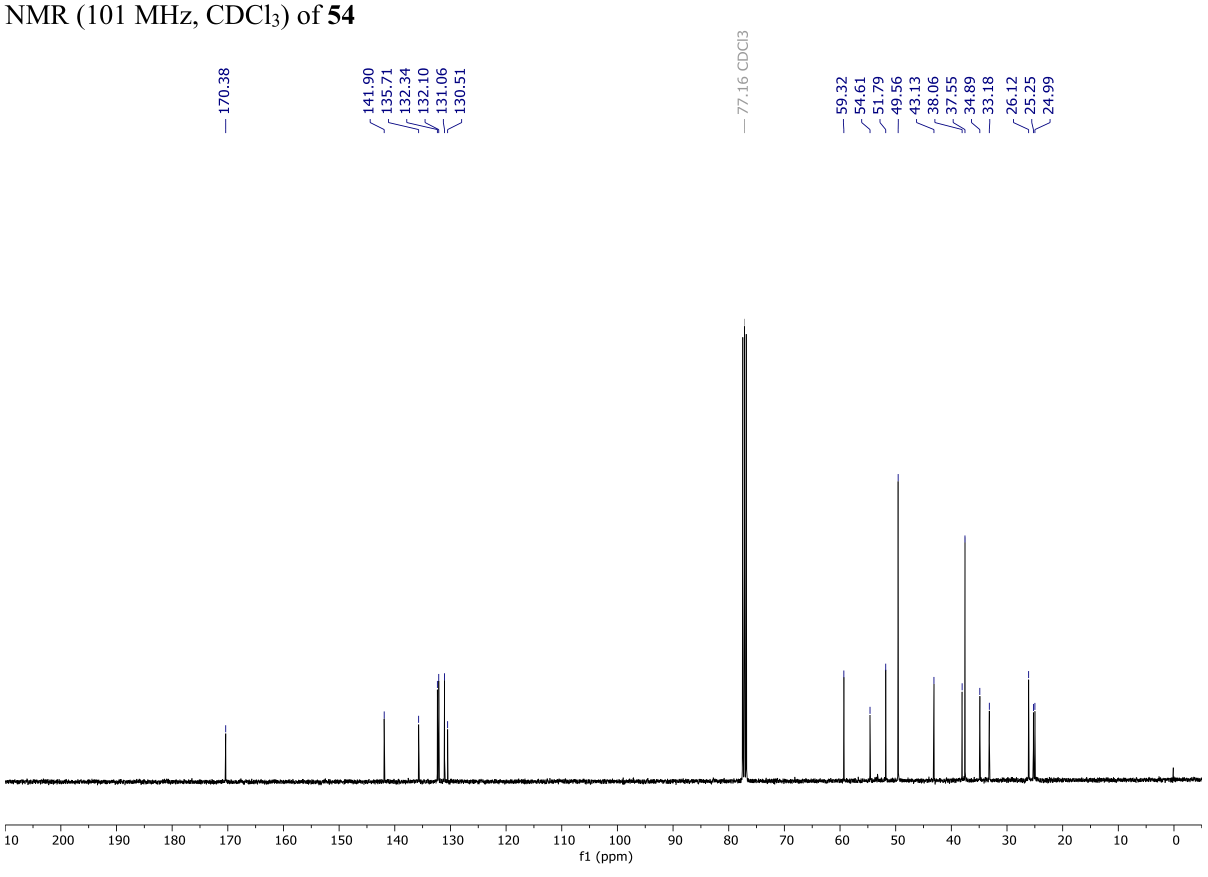

^{1}H NMR (400 MHz, $CDCl_3$) of **55**

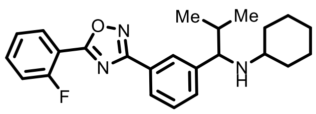

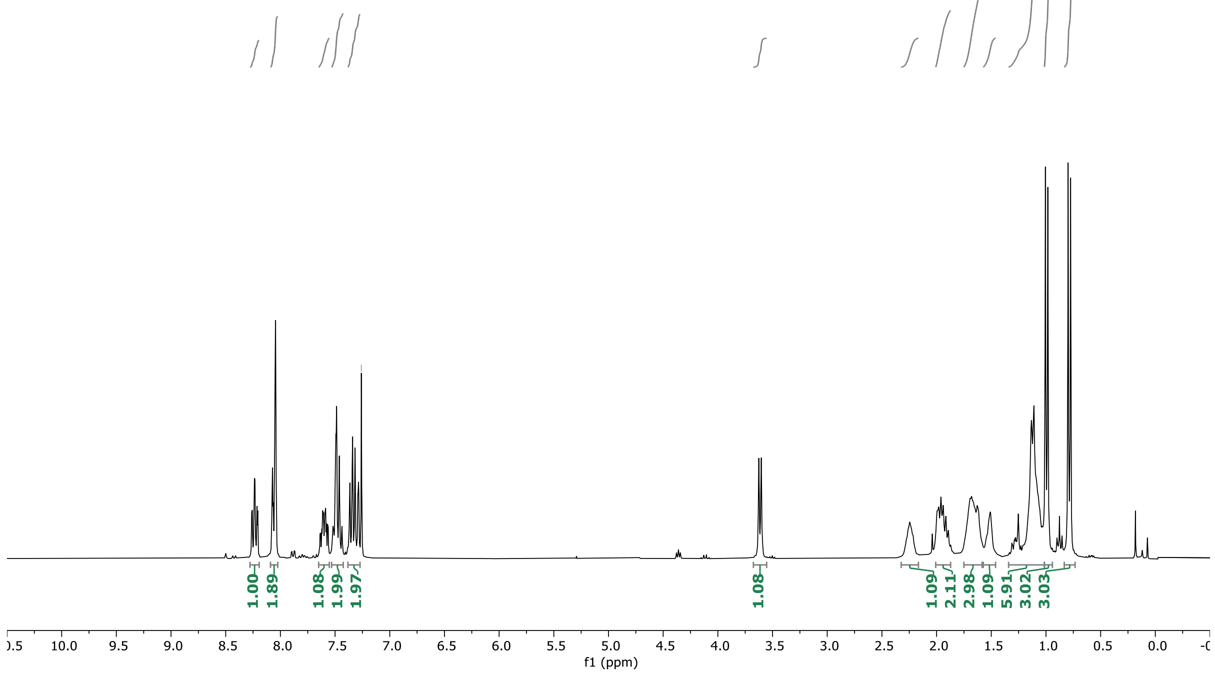

^{13}C NMR (101 MHz, $CDCl_3$) of **55**

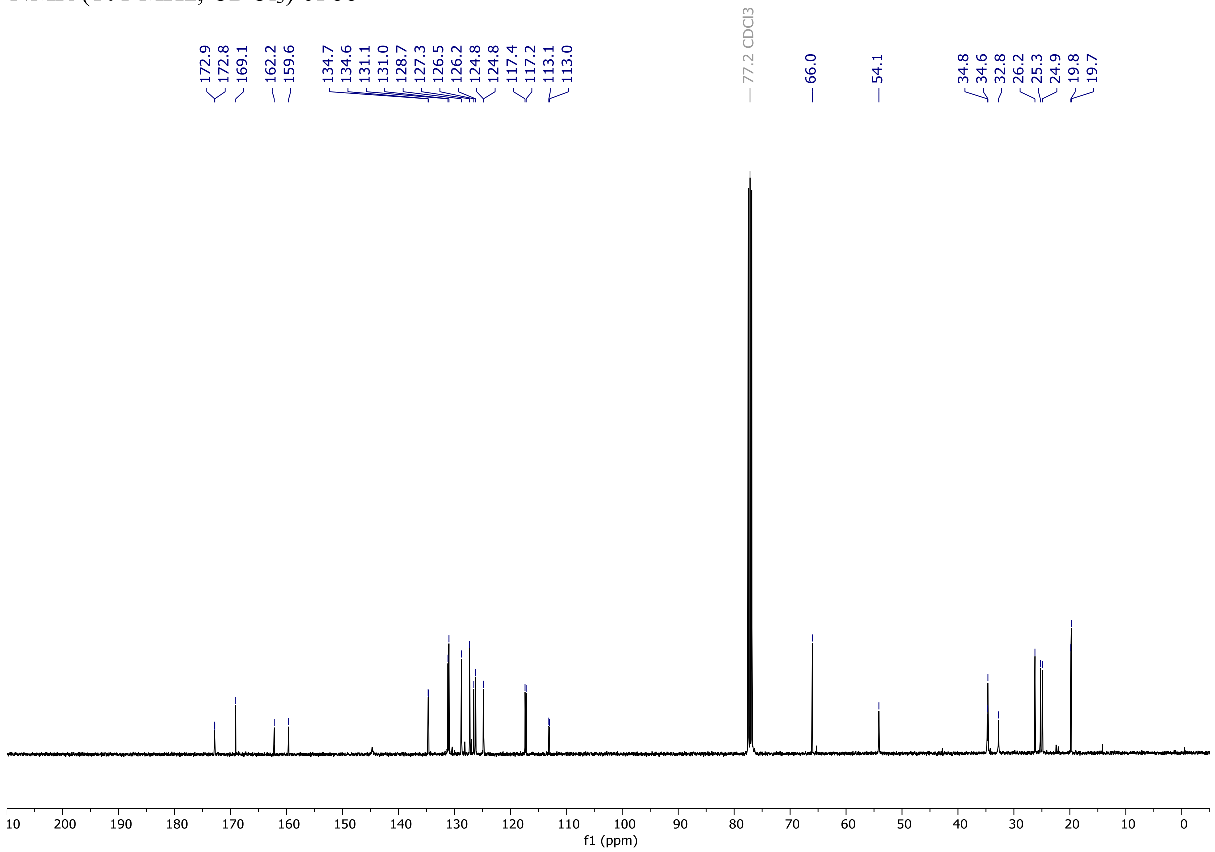

^{19}F NMR (282 MHz, $CDCl_3$) of **55**

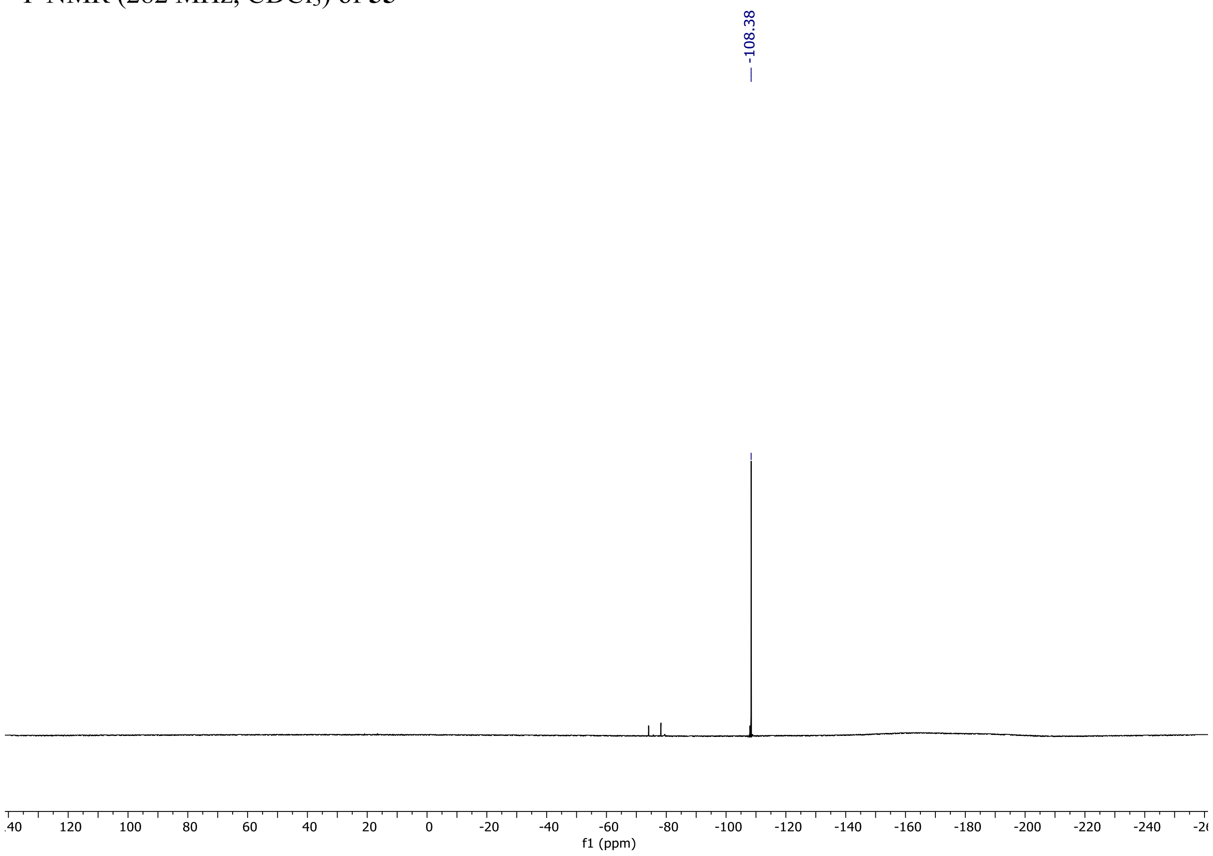

^{1}H NMR (400 MHz, $CDCl_3$) of **56**

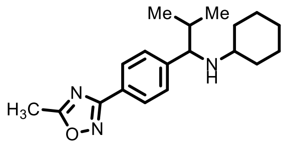

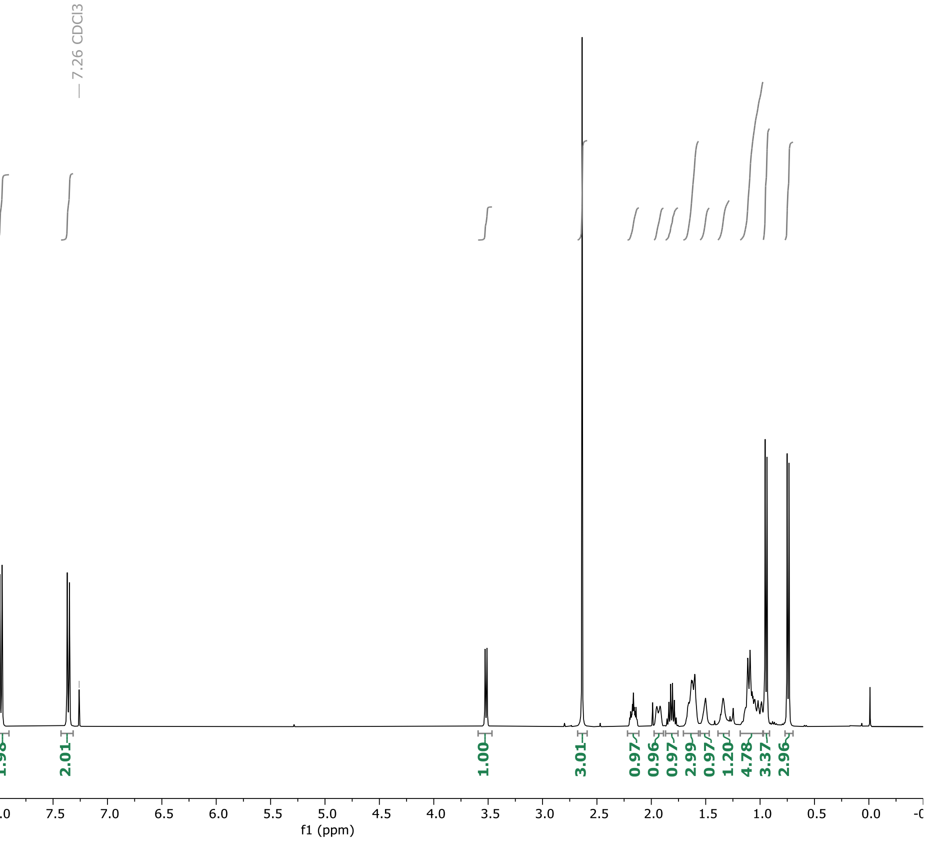

^{13}C NMR (101 MHz, $CDCl_3$) of **56**

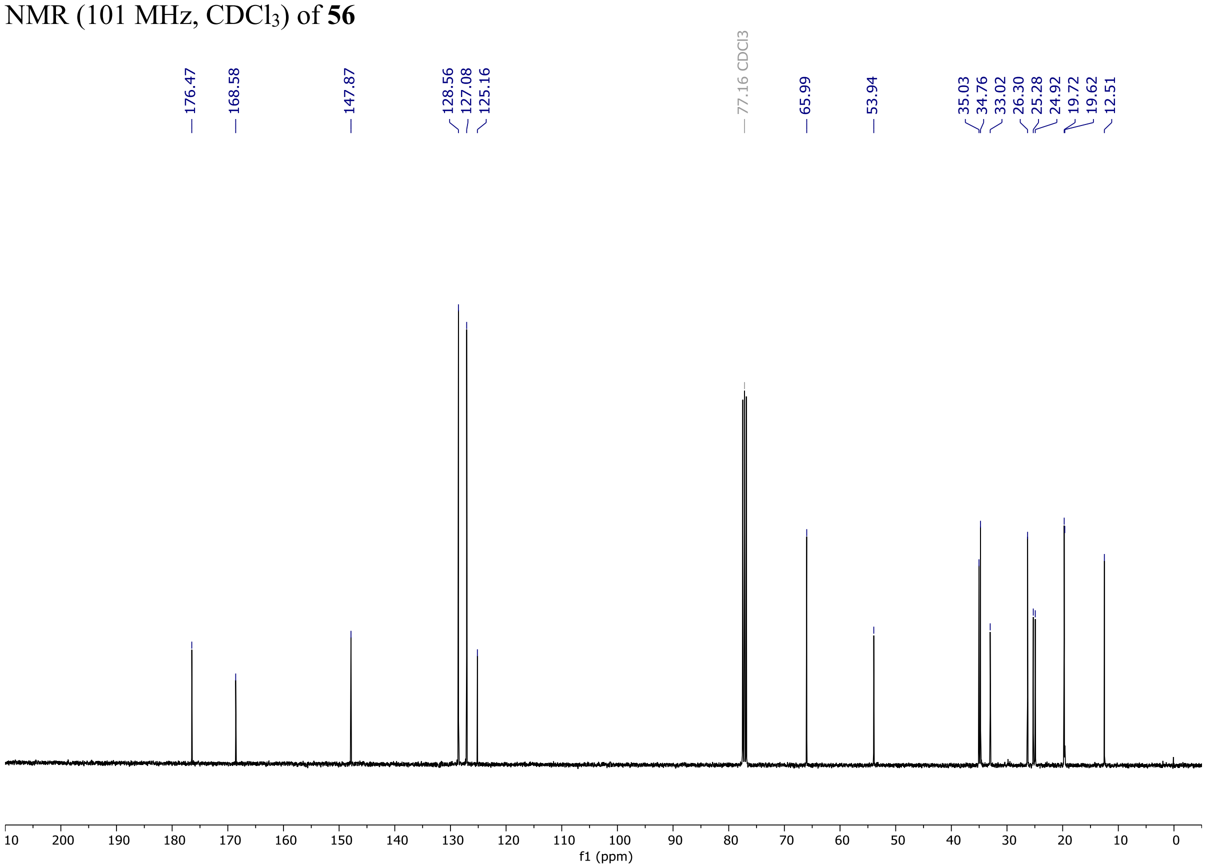

^{1}H NMR (400 MHz, $CDCl_3$) of **57**

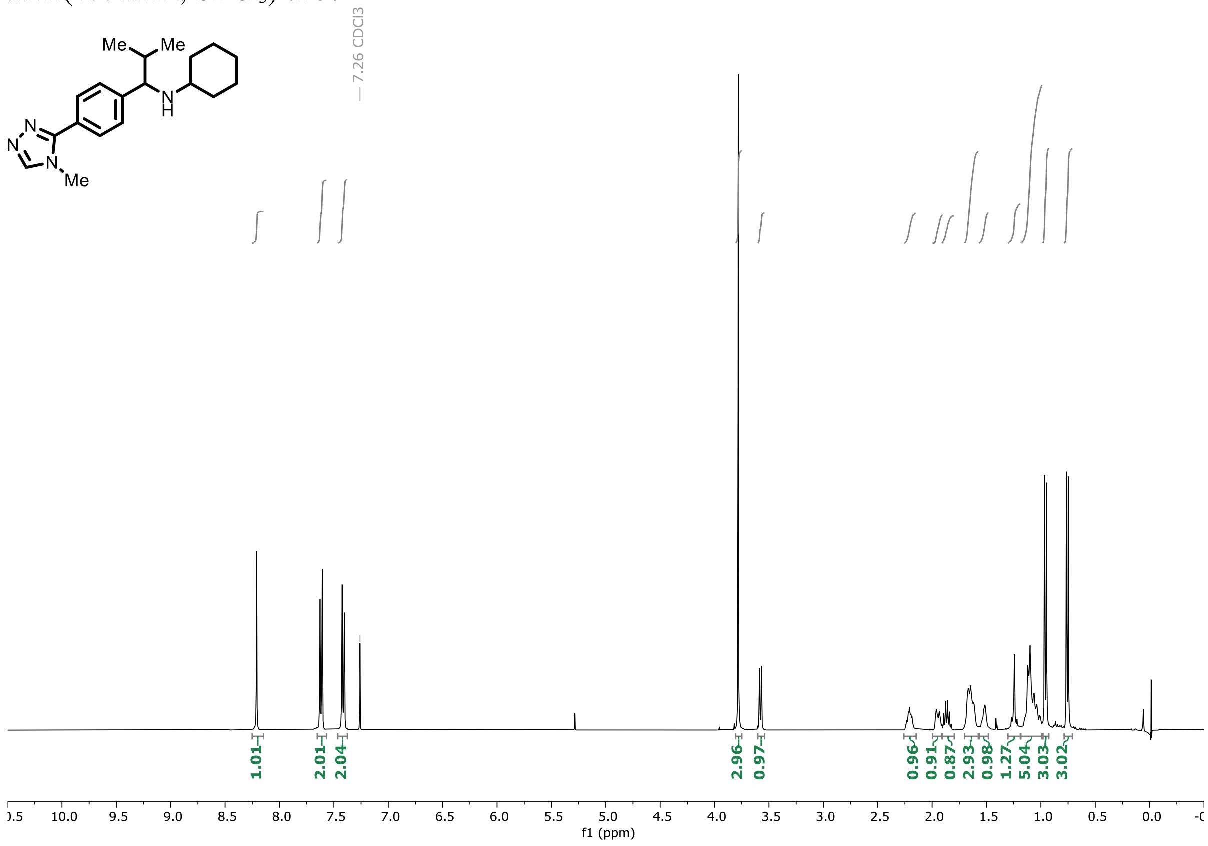

^{13}C NMR (101 MHz, $CDCl_3$) of **57**

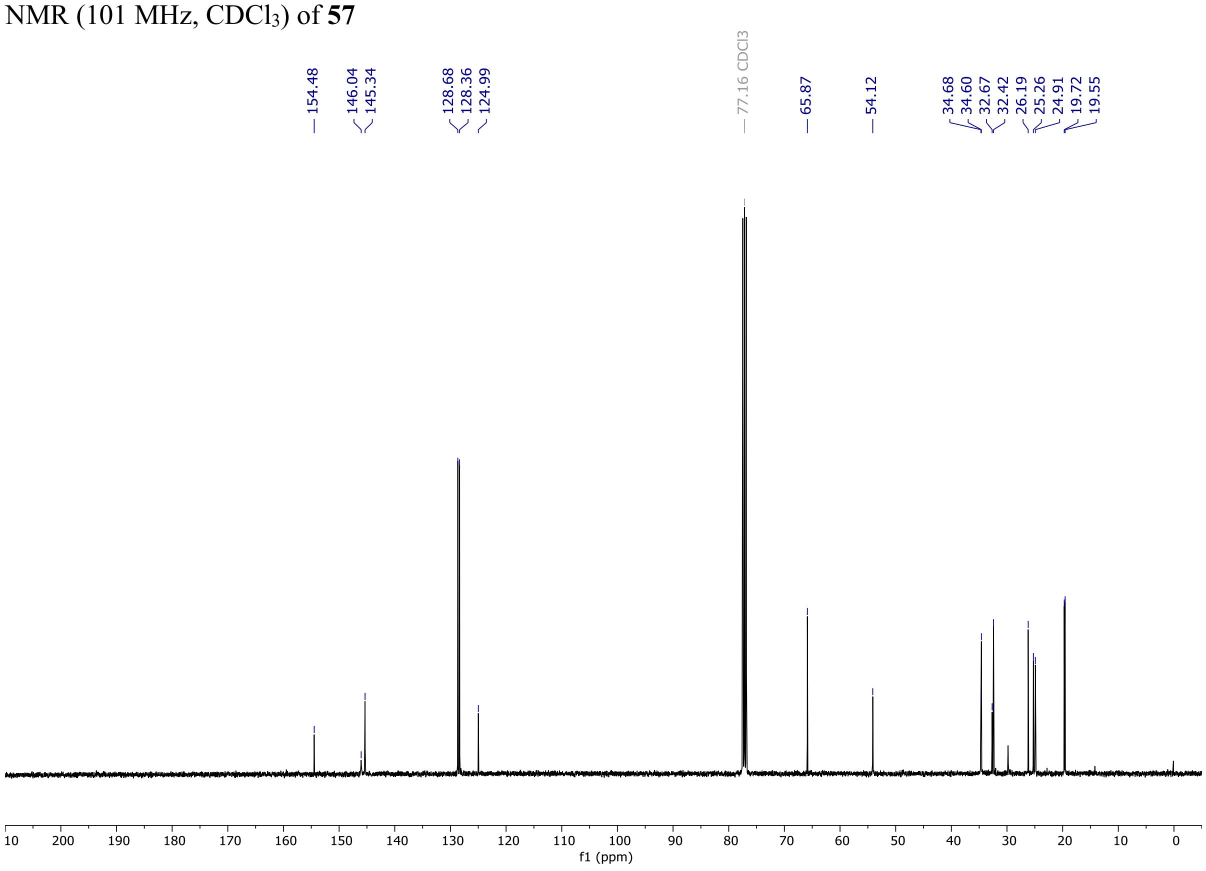

^{1}H NMR (400 MHz, $CDCl_3$) of **58**

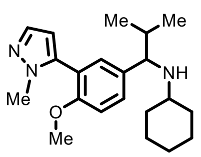

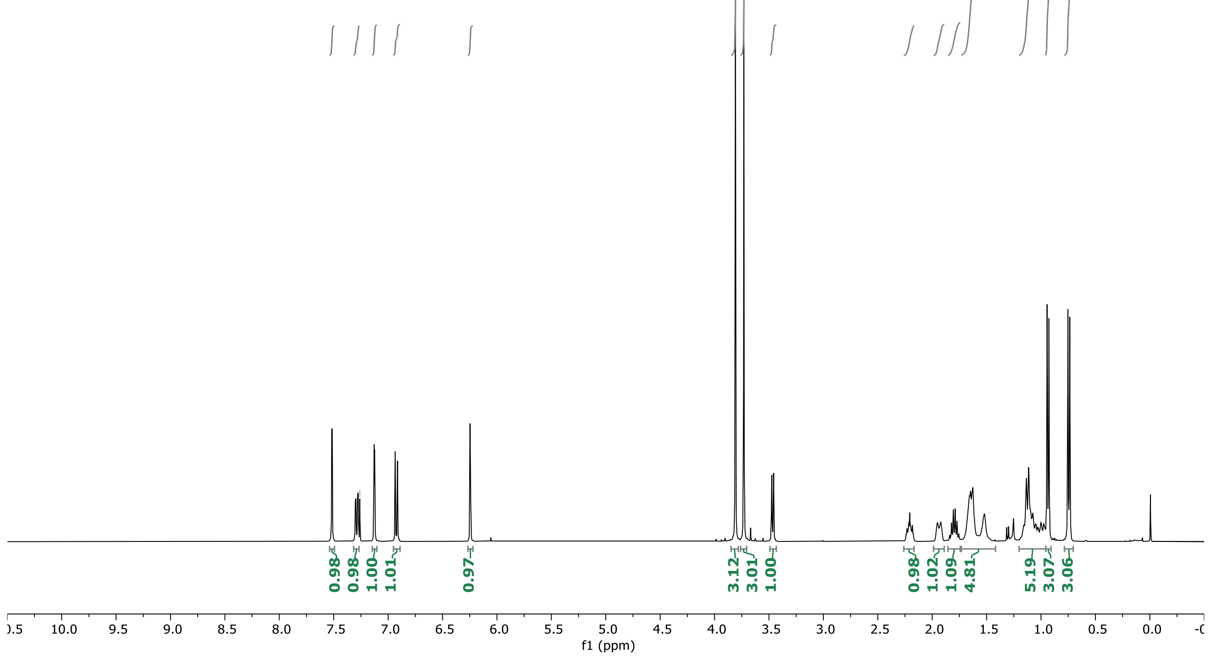

^{13}C NMR (101 MHz, $CDCl_3$) of **58**

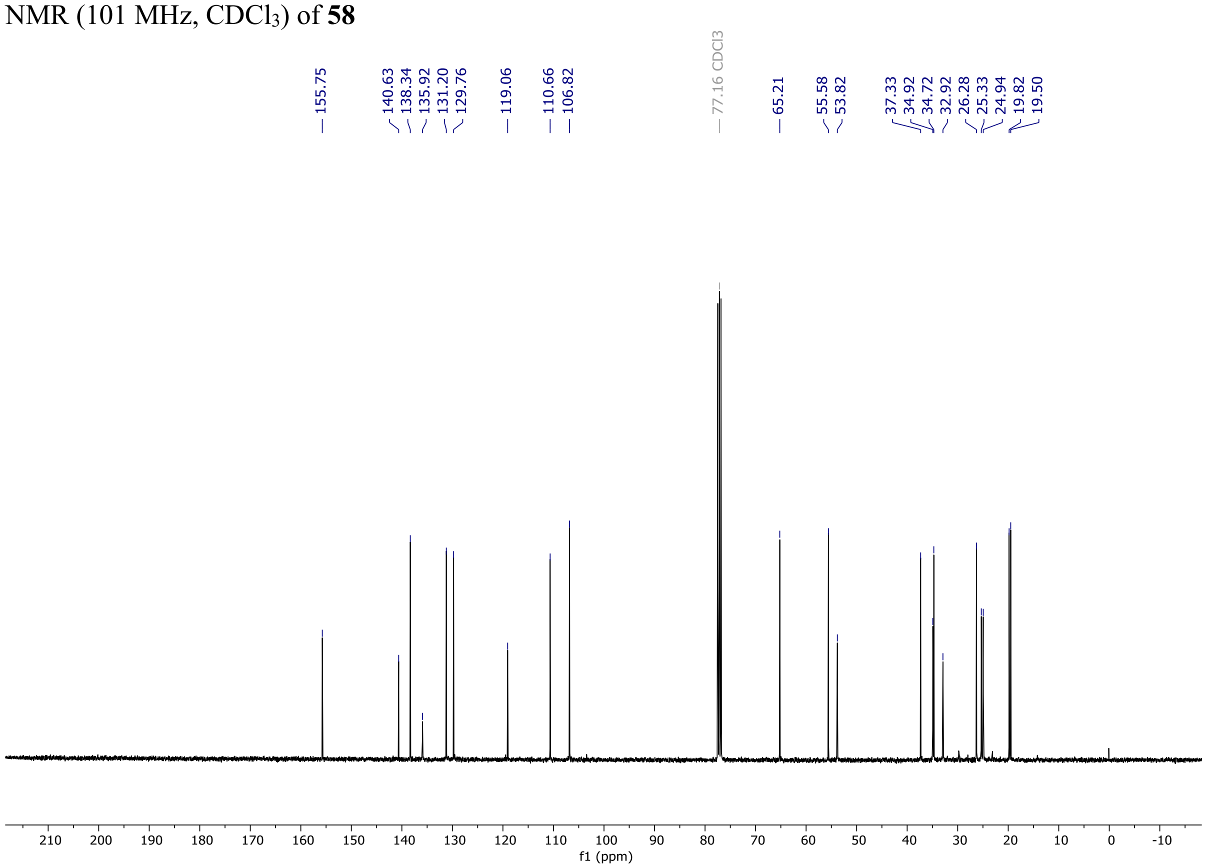

^{1}H NMR (400 MHz, $CDCl_3$) of **59**

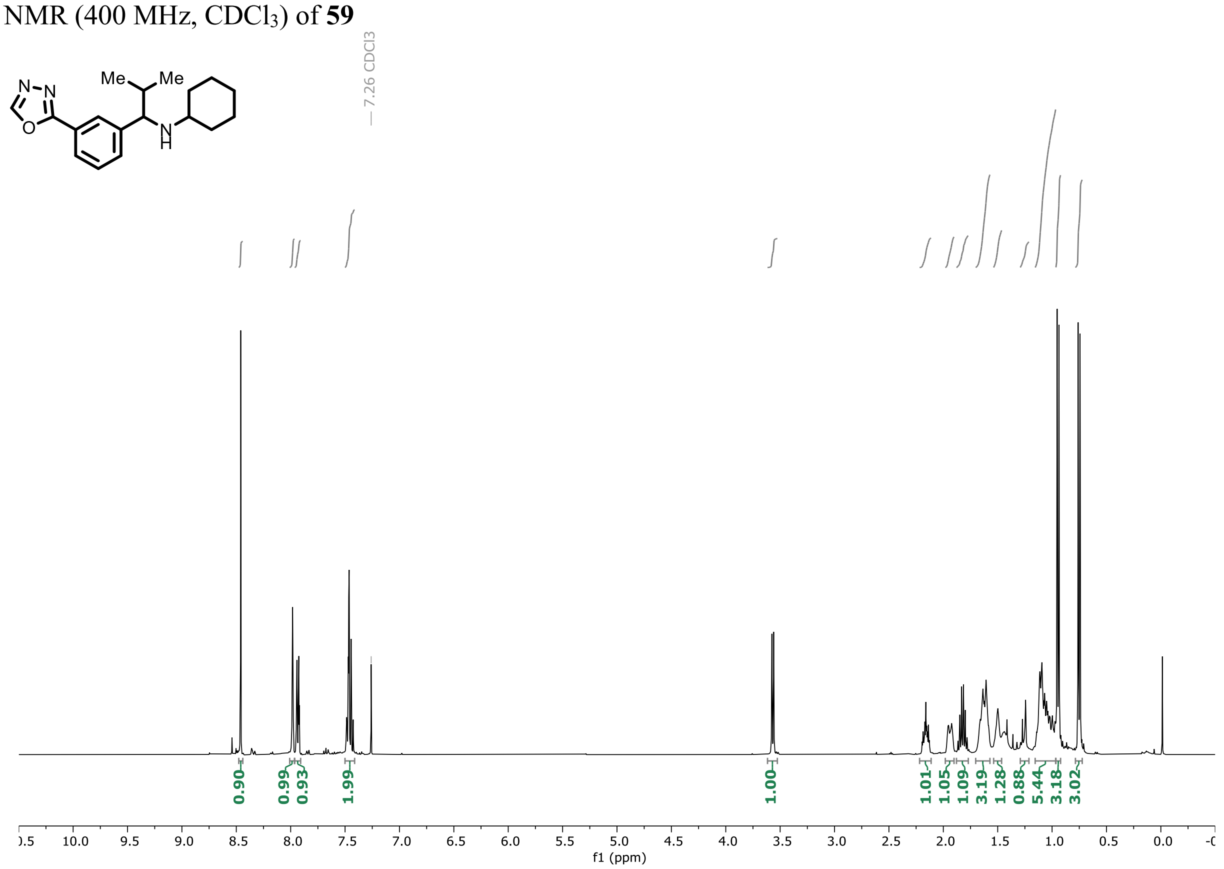

^{13}C NMR (101 MHz, $CDCl_3$) of **59**

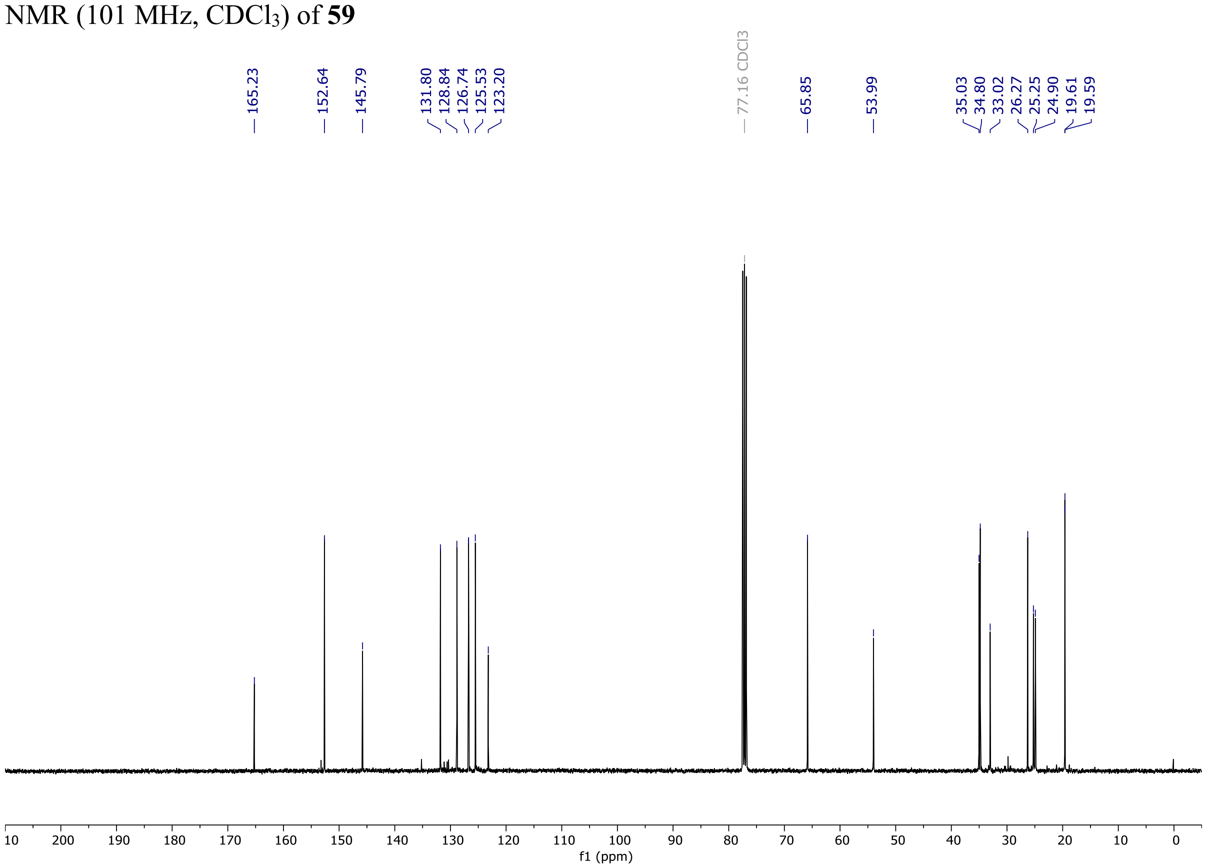

^{1}H NMR (400 MHz, $CDCl_3$) of **60**

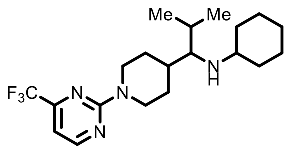

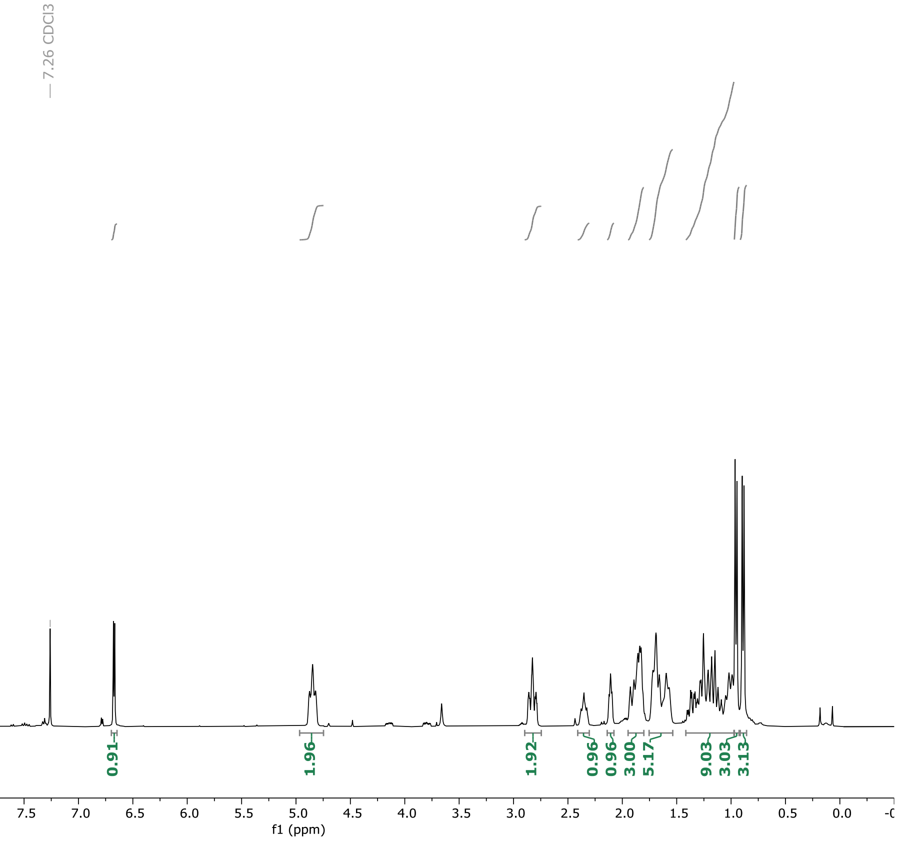

^{13}C NMR (101 MHz, $CDCl_3$) of **60**

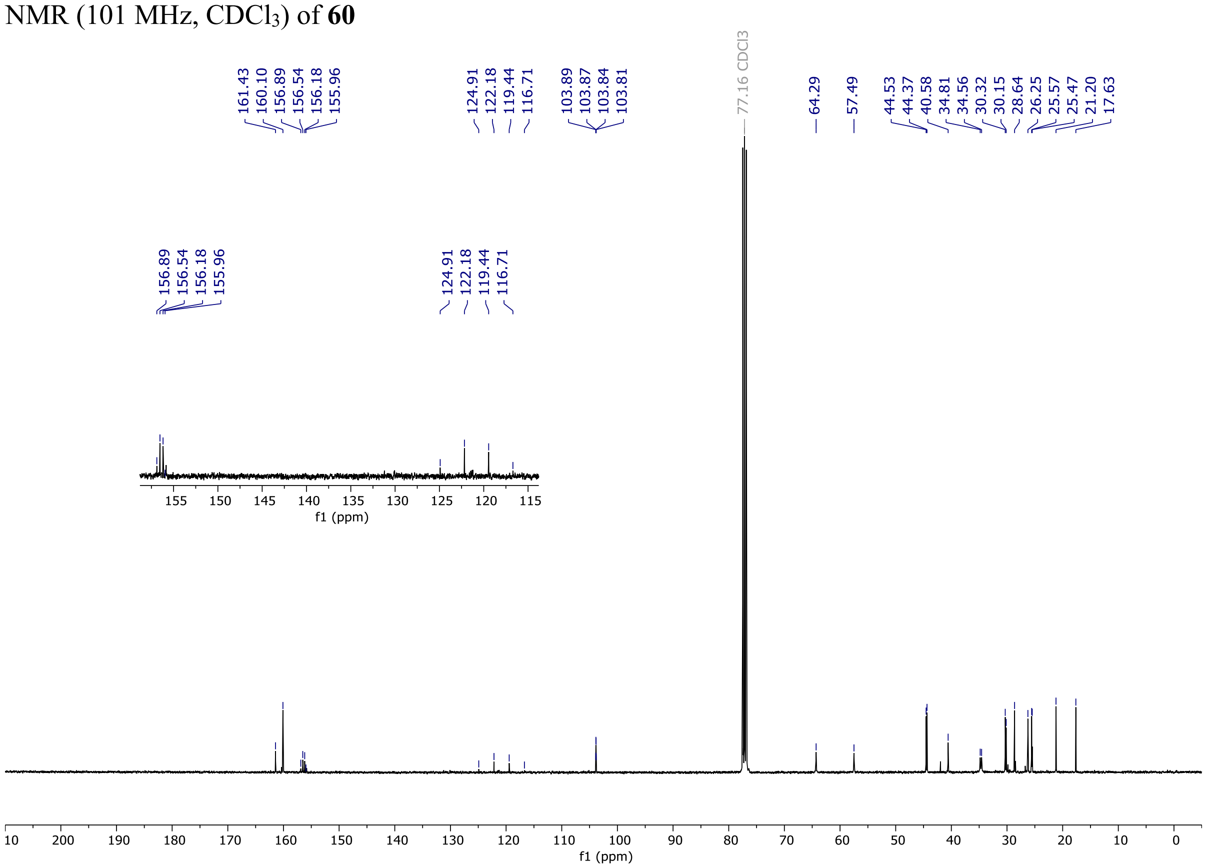

^{19}F NMR (282 MHz, $CDCl_3$) of **60**

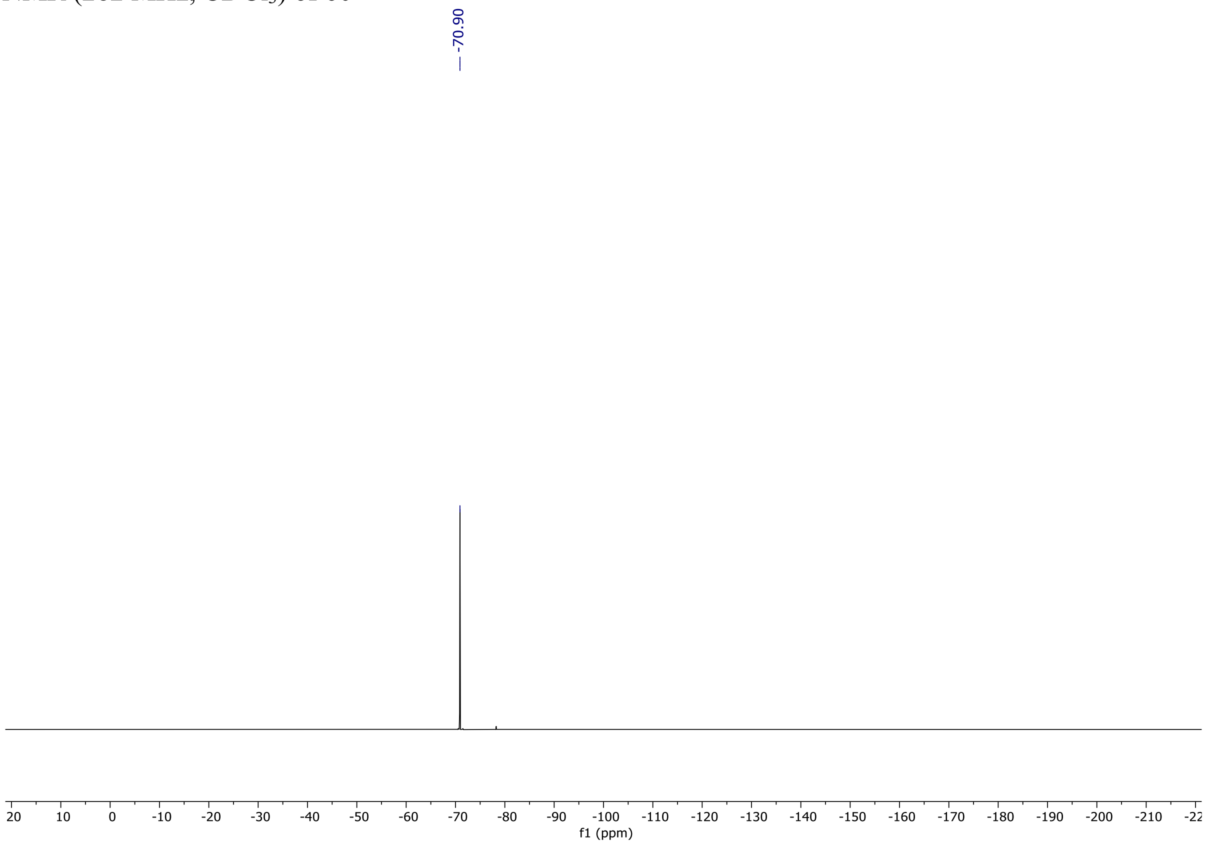

^{1}H NMR (400 MHz, $CDCl_3$) of **61**

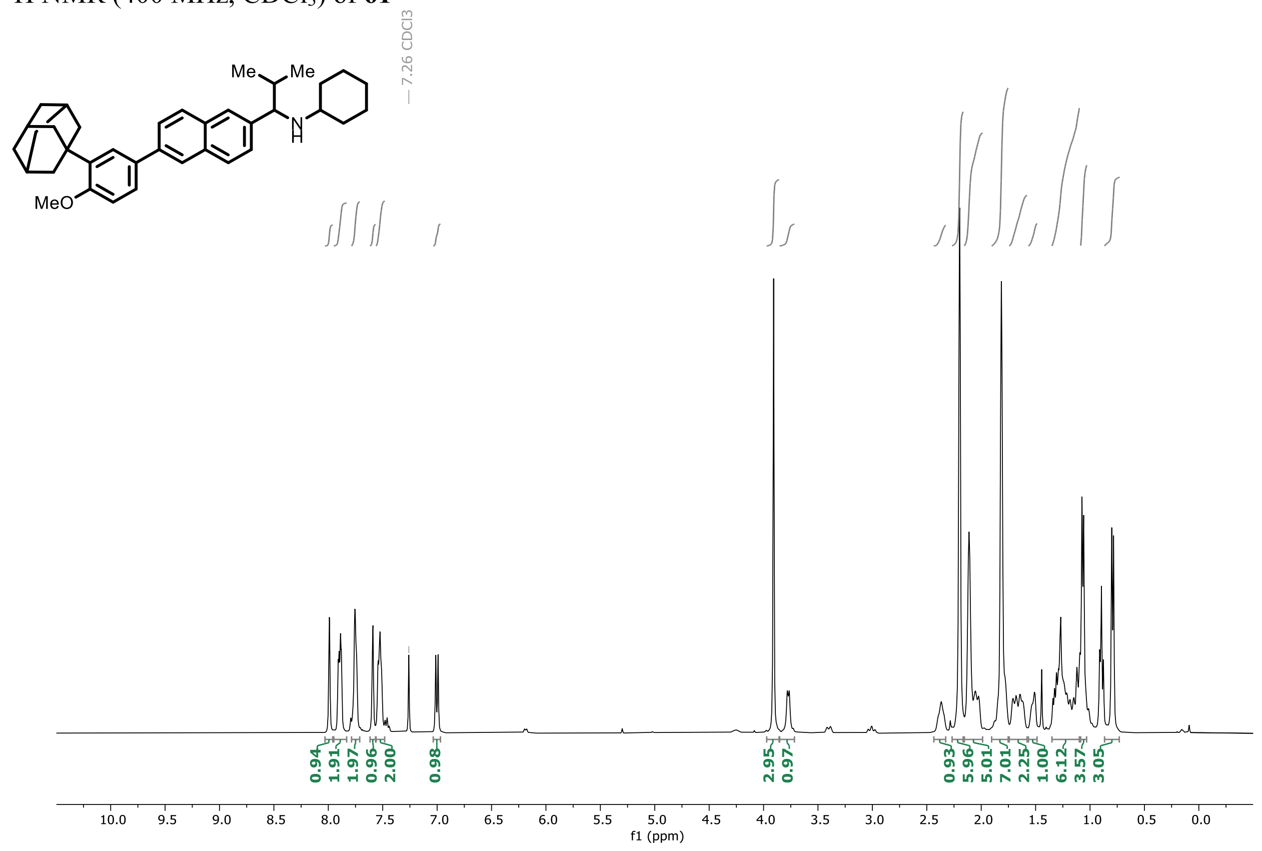

^{13}C NMR (101 MHz, $CDCl_3$) of **61**

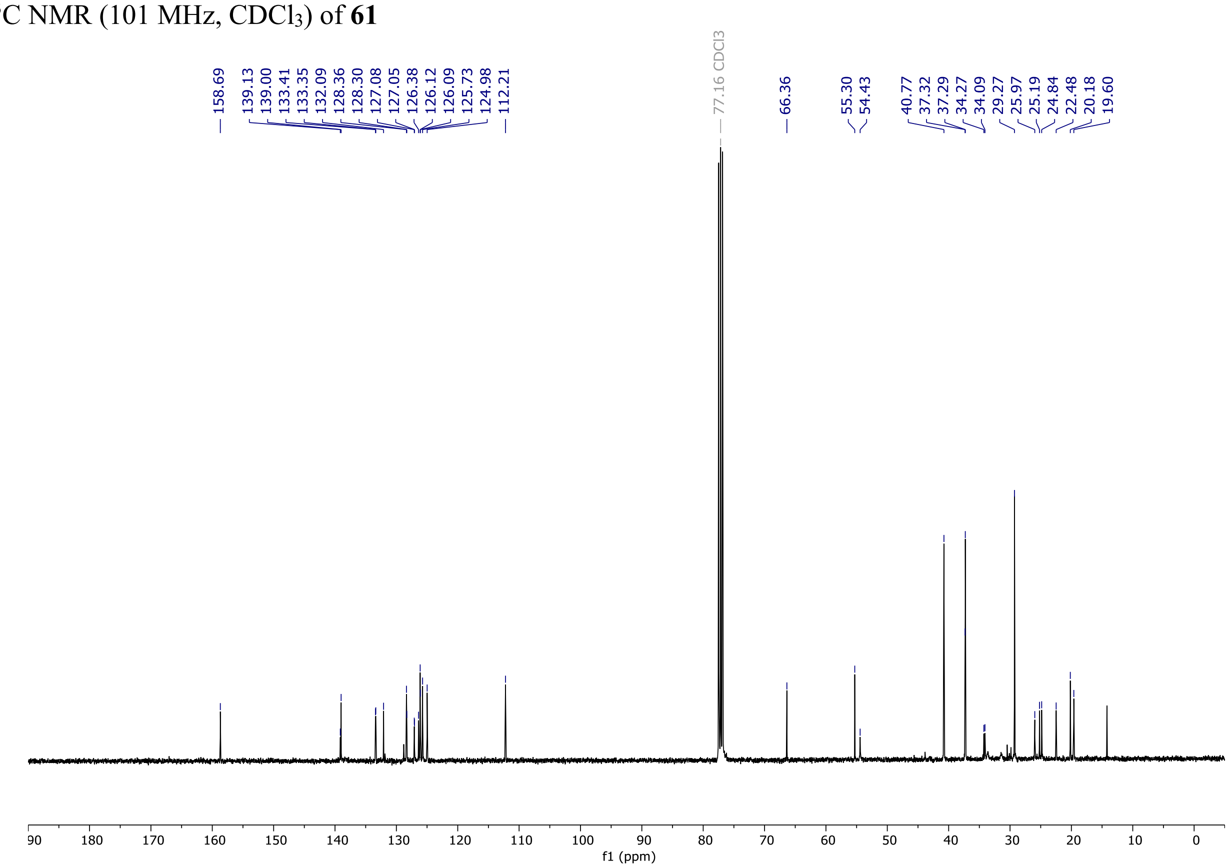

^{1}H NMR (400 MHz, $CDCl_3$) of **62**

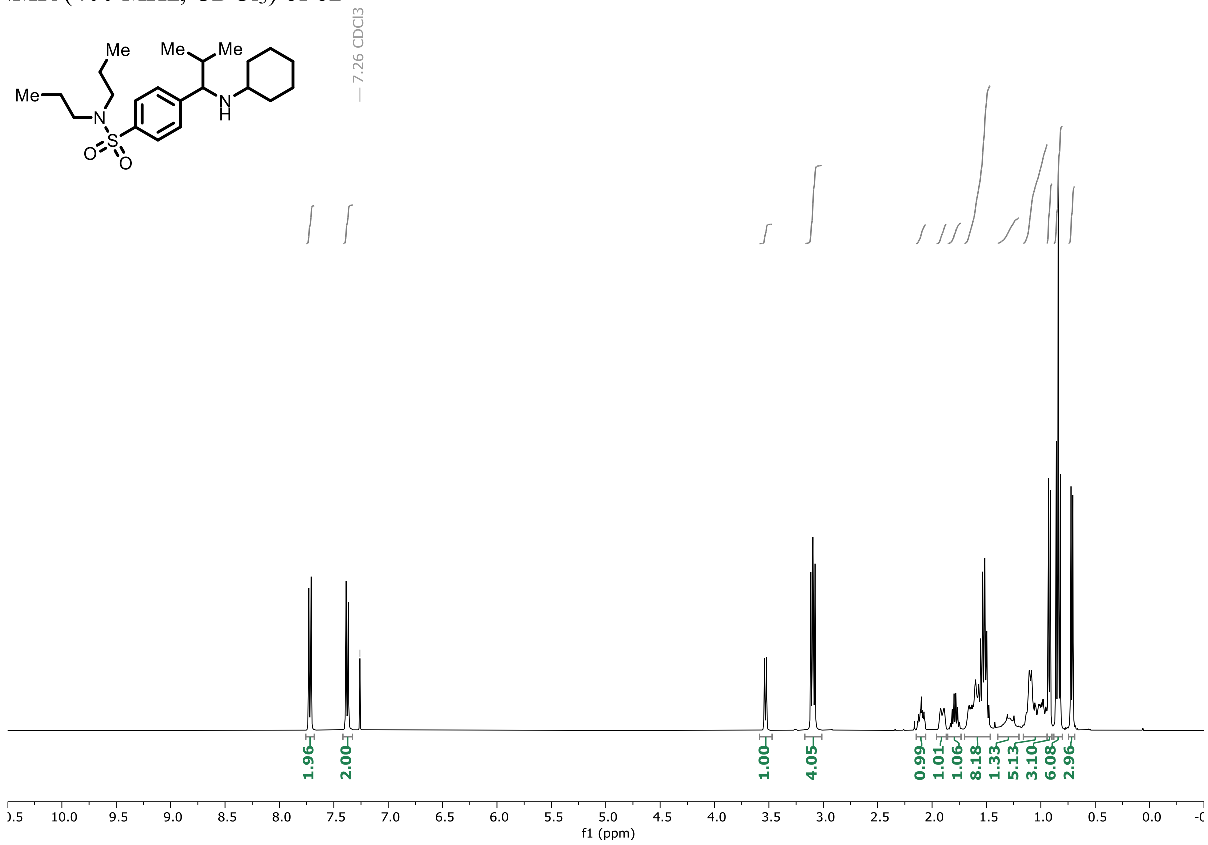

^{13}C NMR (101 MHz, $CDCl_3$) of **62**

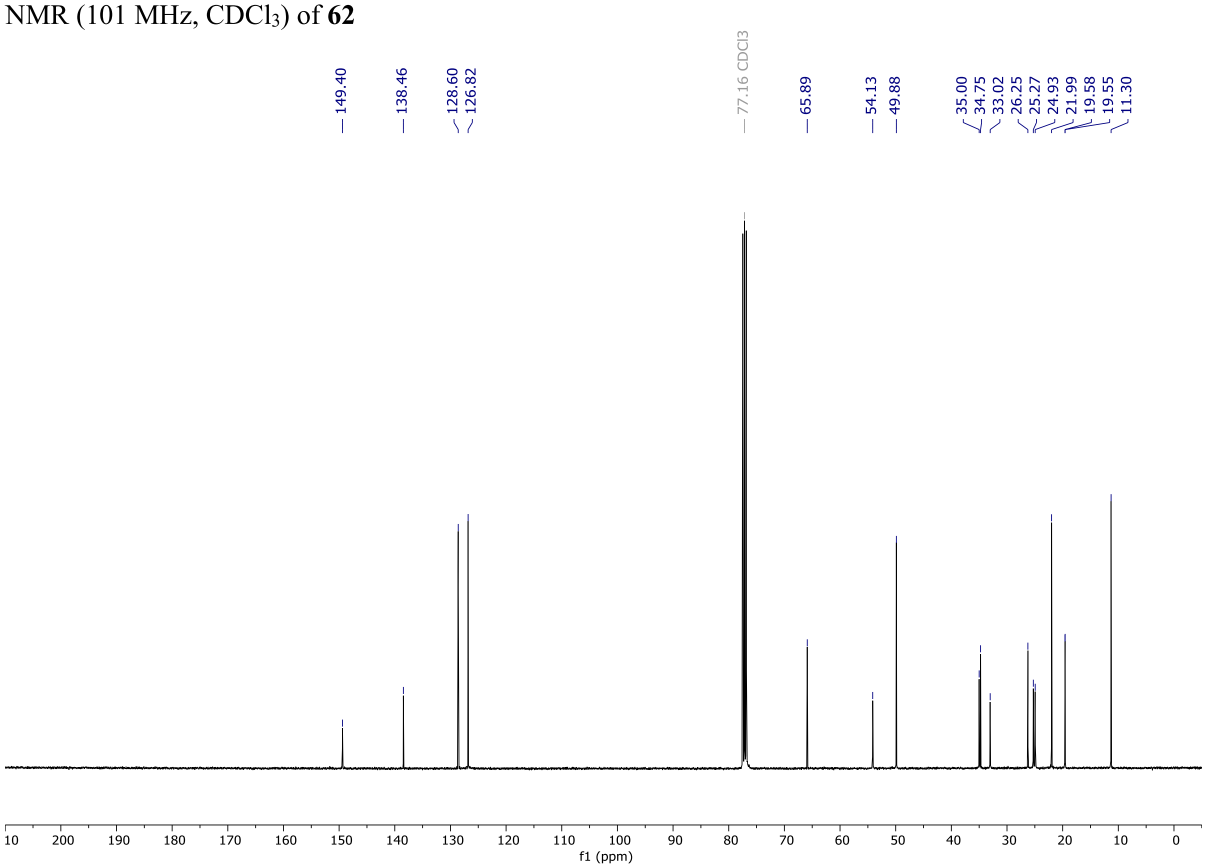

^{1}H NMR (400 MHz, $CDCl_3$) of **63**

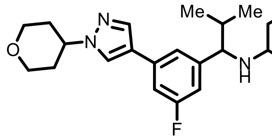

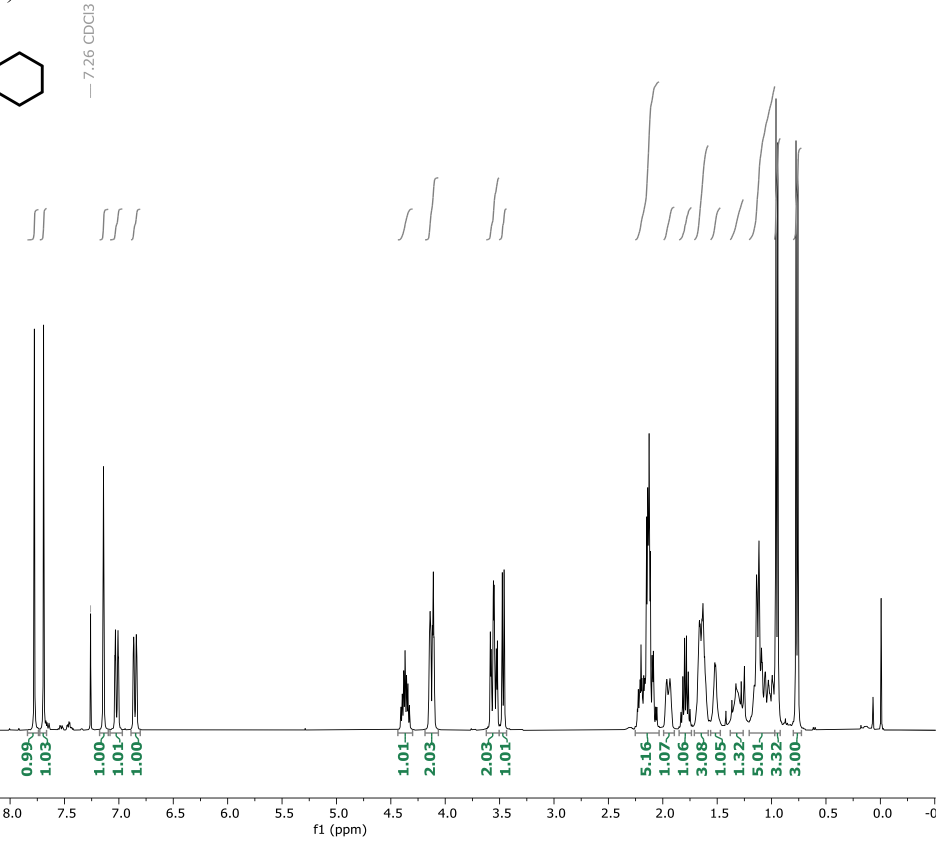

^{13}C NMR (101 MHz, $CDCl_3$) of **63**

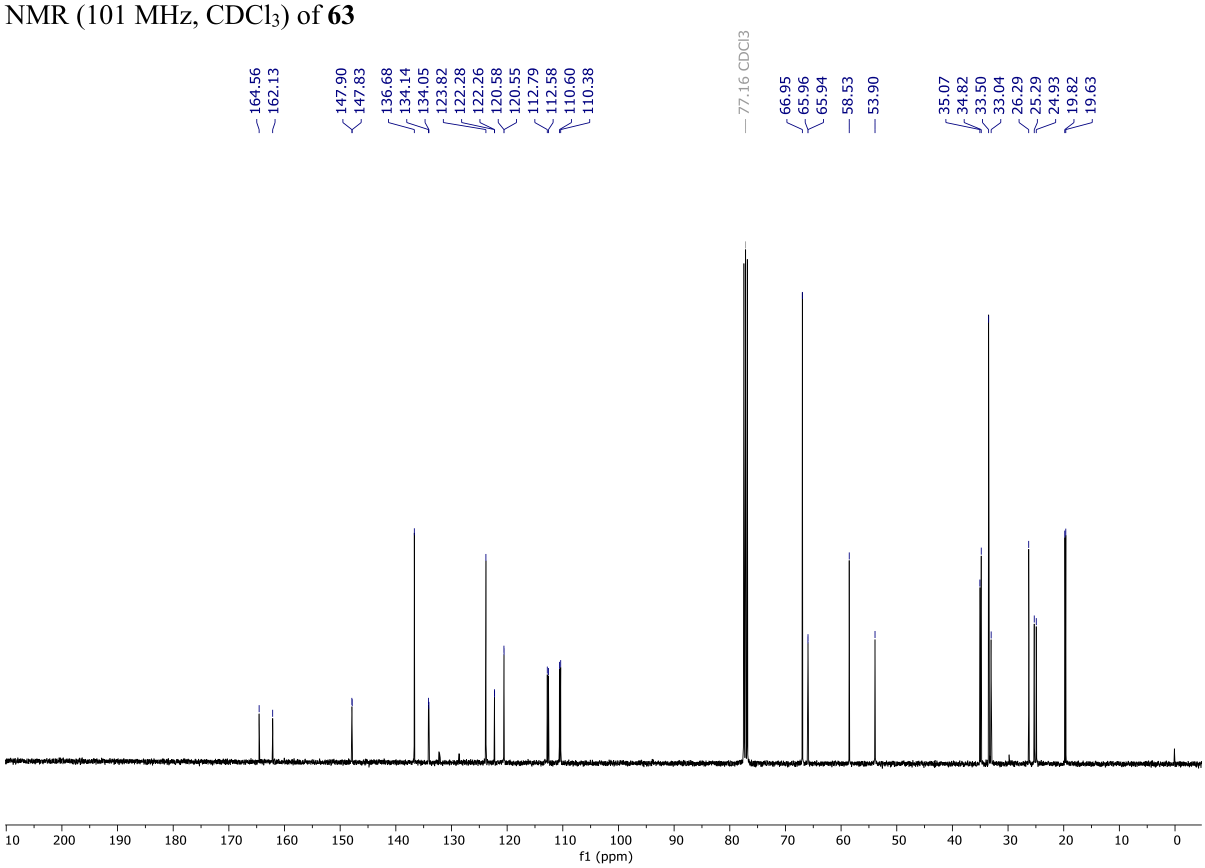

^{19}F NMR (376 MHz, $CDCl_3$) of **63**

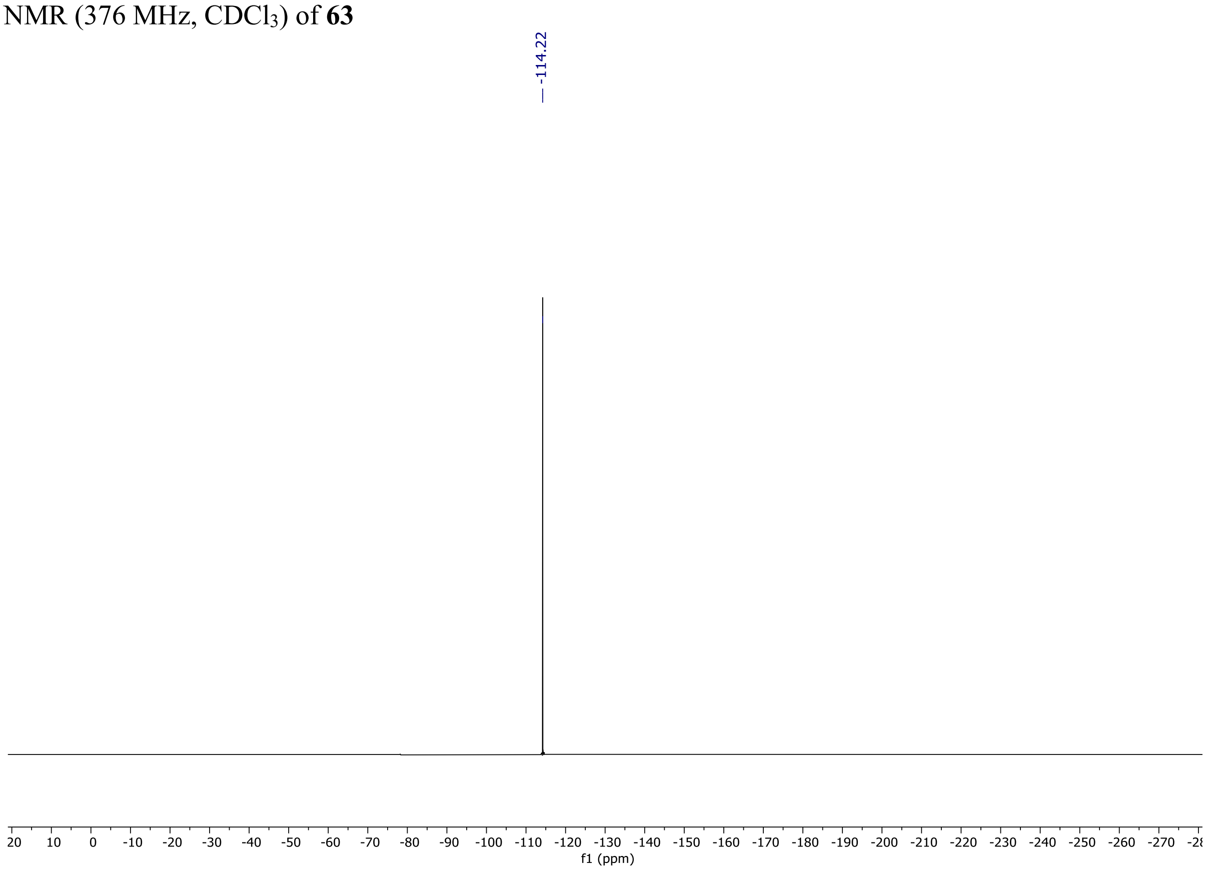

15. NMR spectra of products (64-71, 79)

^{1}H NMR (400 MHz, MeOD) of **64**

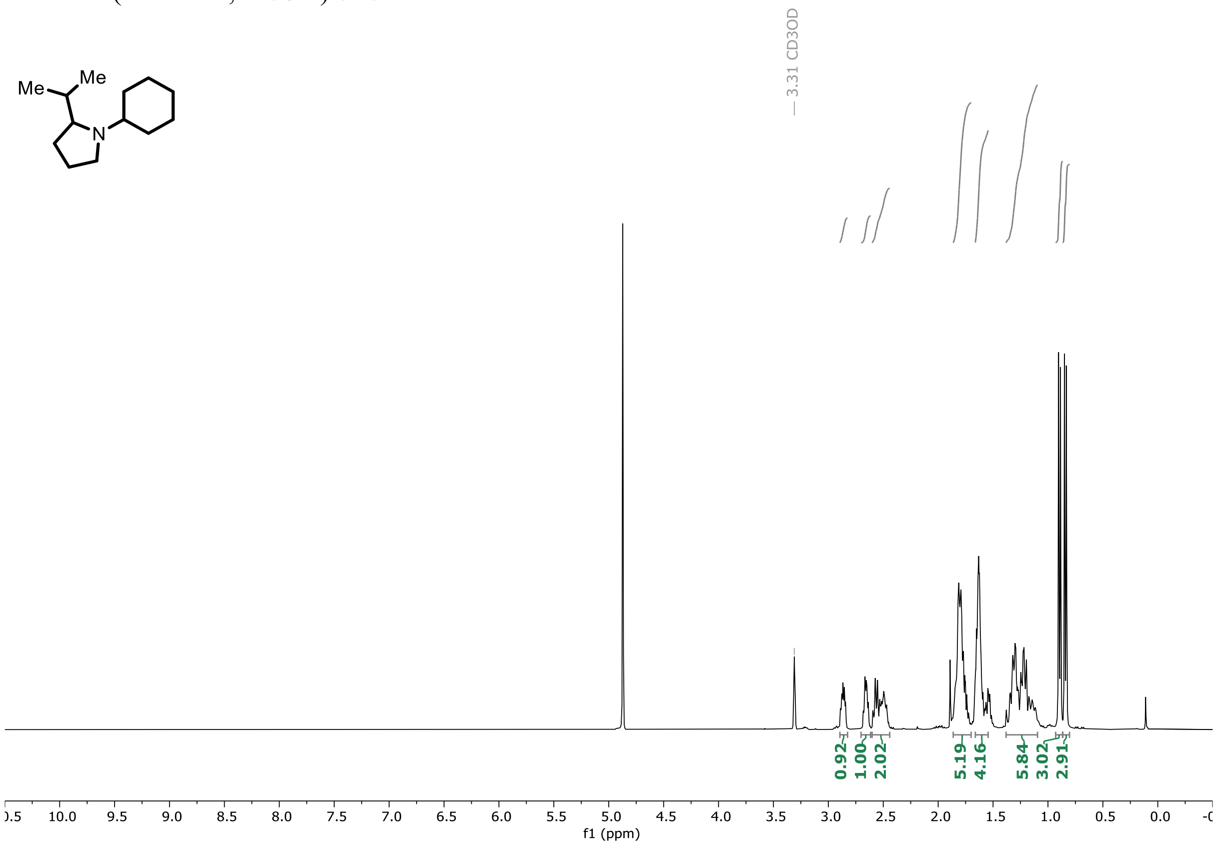

^{13}C NMR (101 MHz, MeOD) of **64**

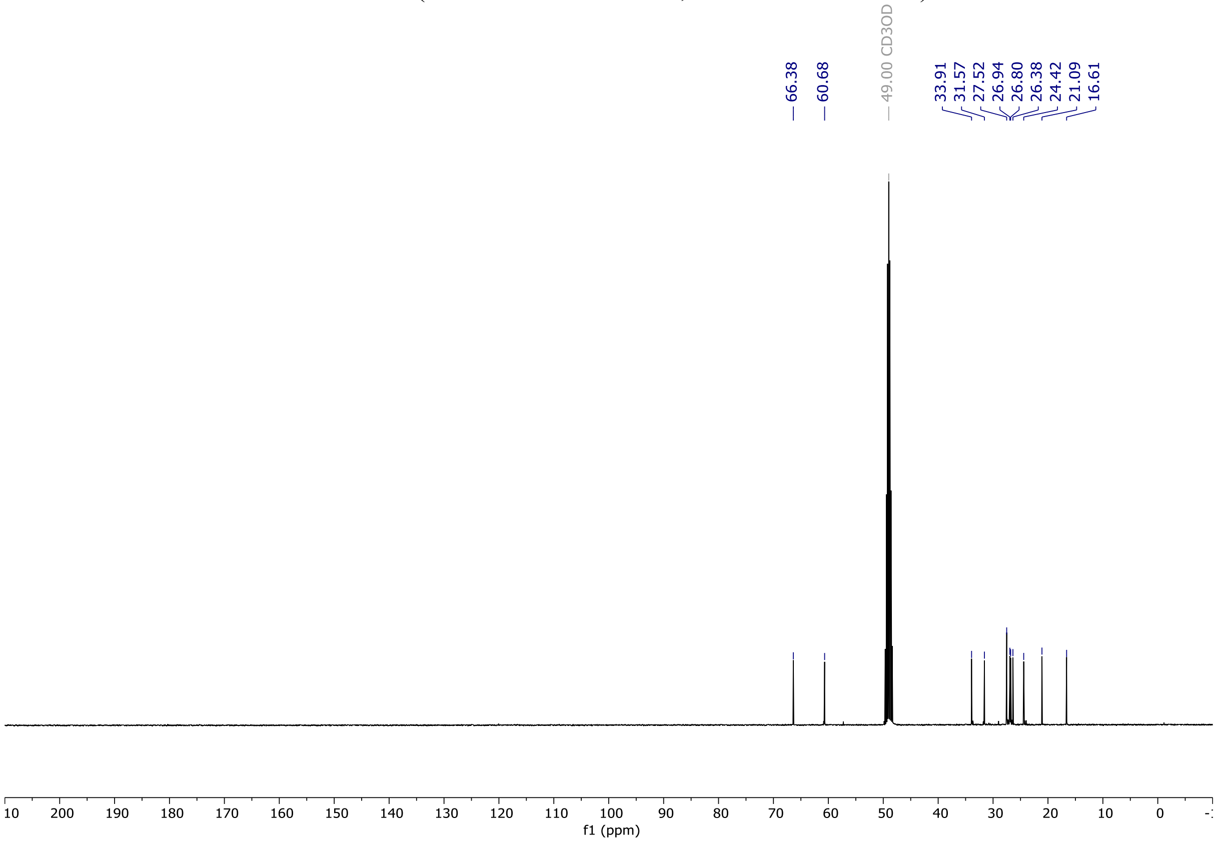

^{1}H NMR (400 MHz, MeOD) of **65**

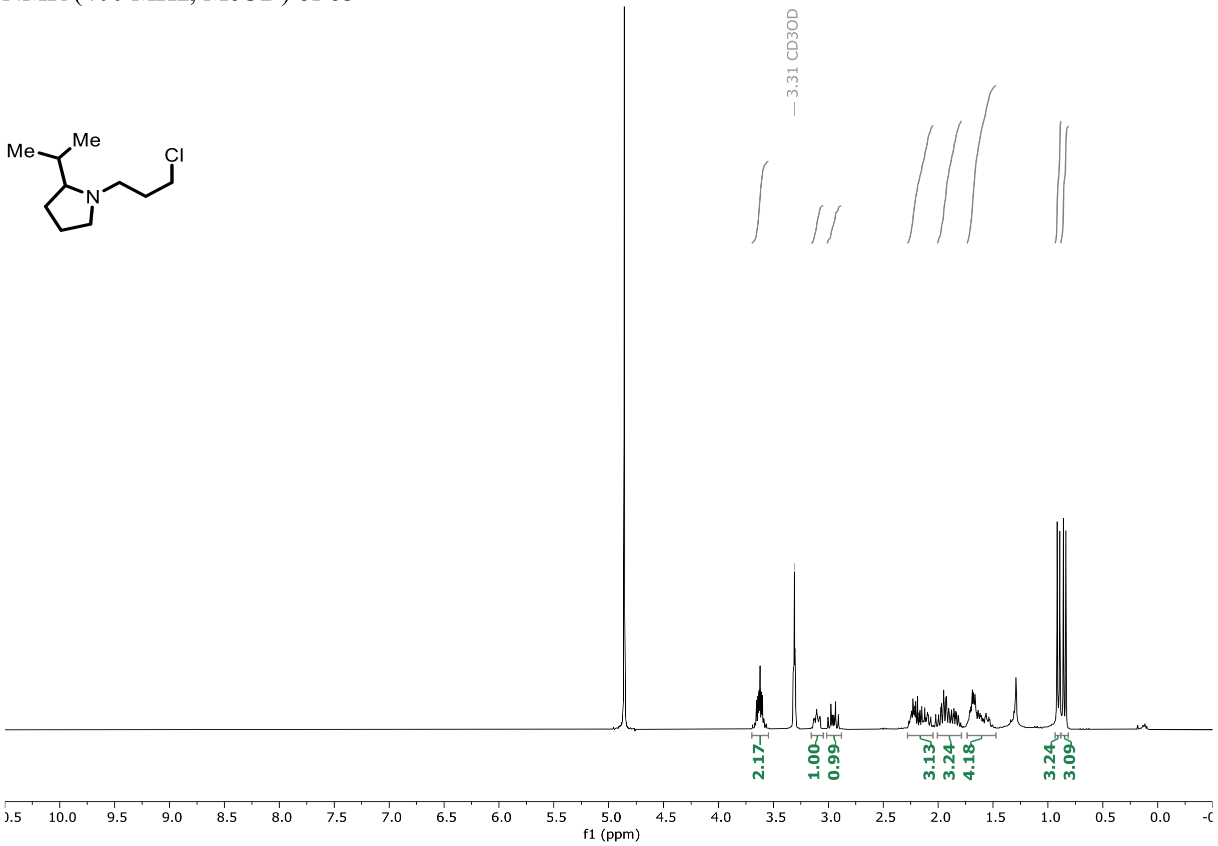

^{13}C NMR (101 MHz, MeOD) of **65**

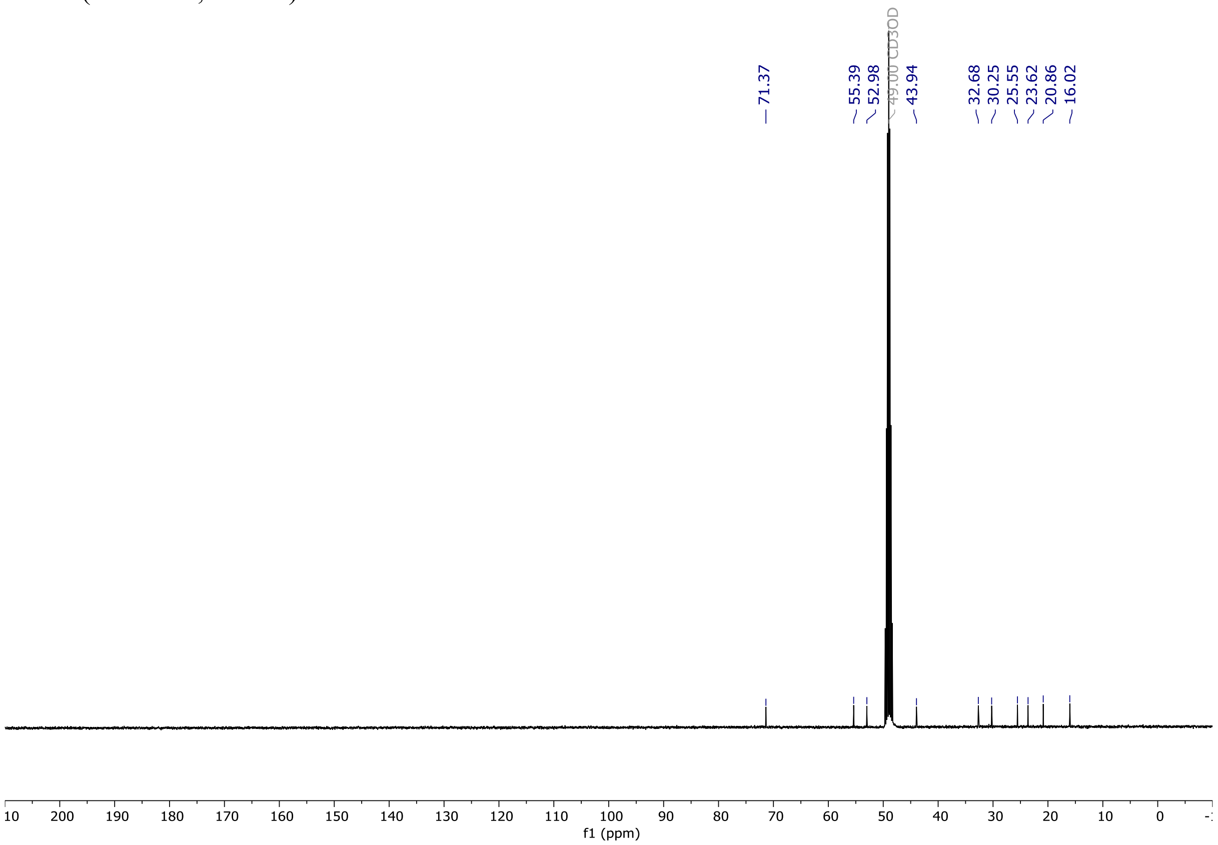

^{1}H NMR (400 MHz, $CDCl_3$) of **66**

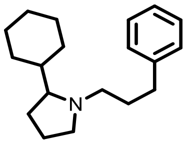

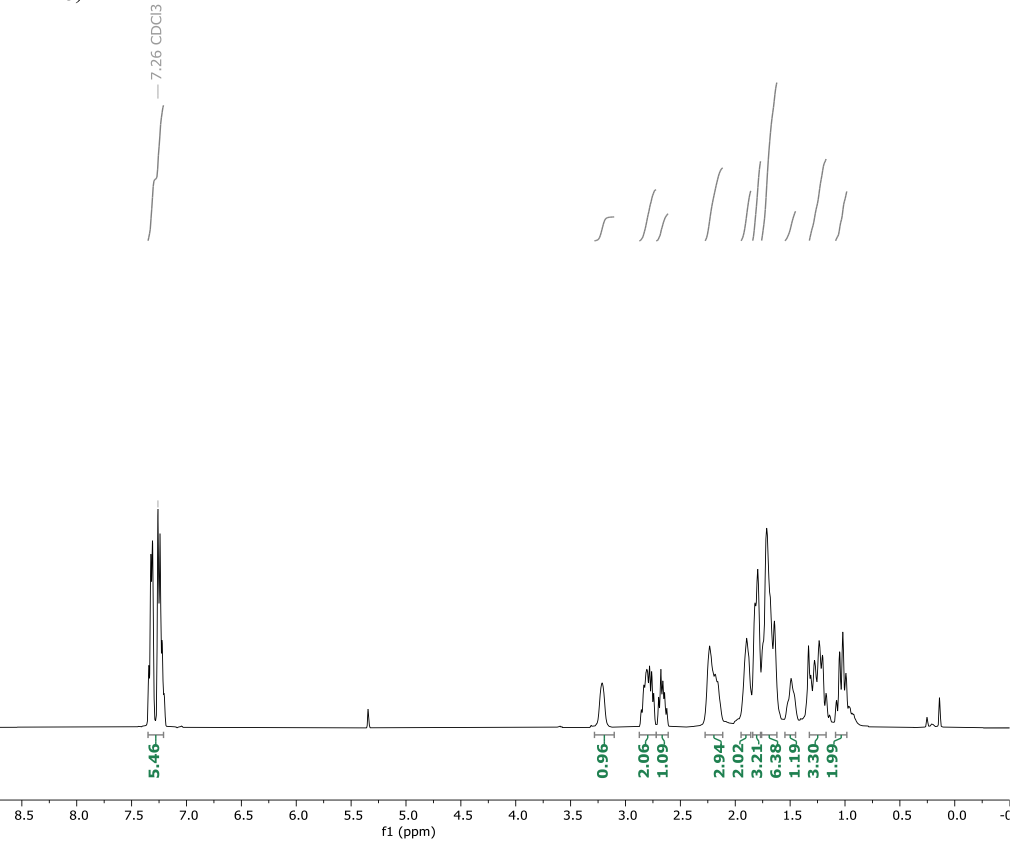

^{13}C NMR (101 MHz, $CDCl_3$) of **66**

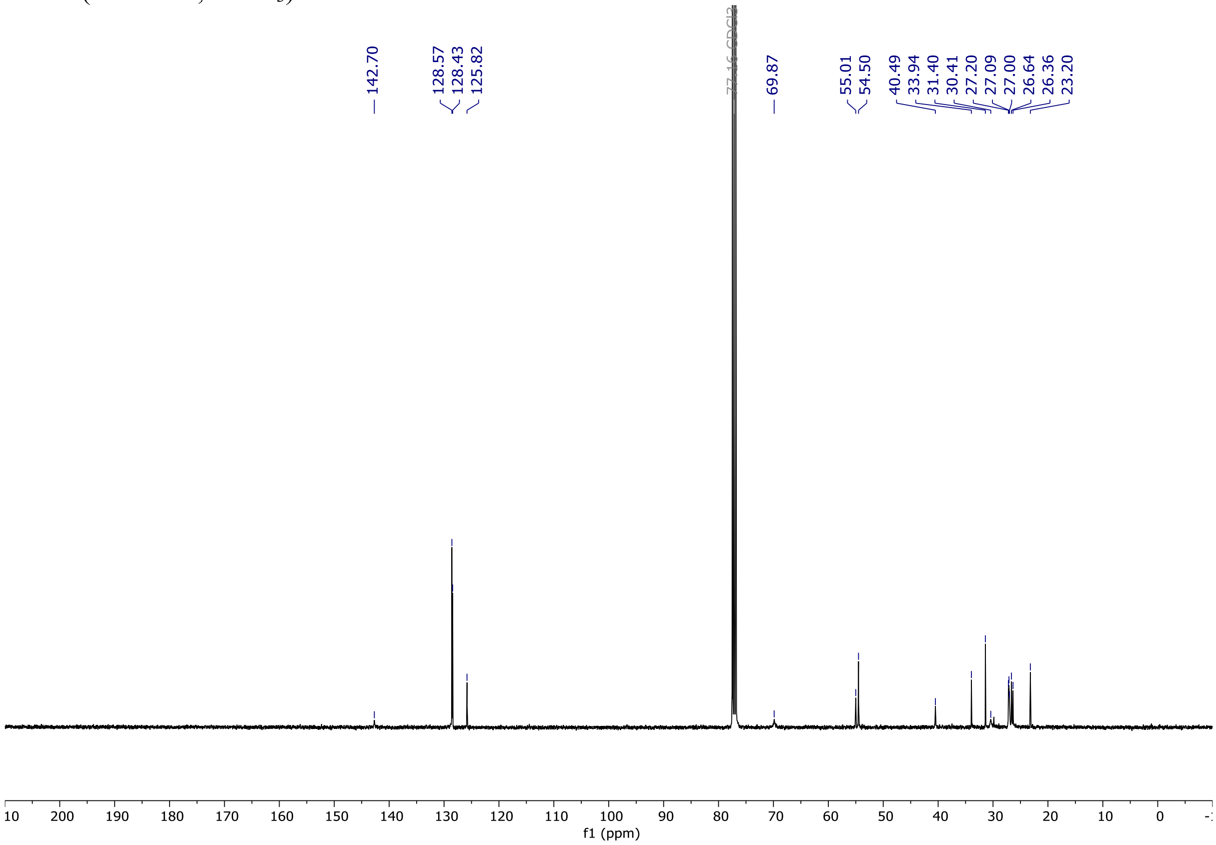

^{1}H NMR (400 MHz, $CDCl_3$) of **67**

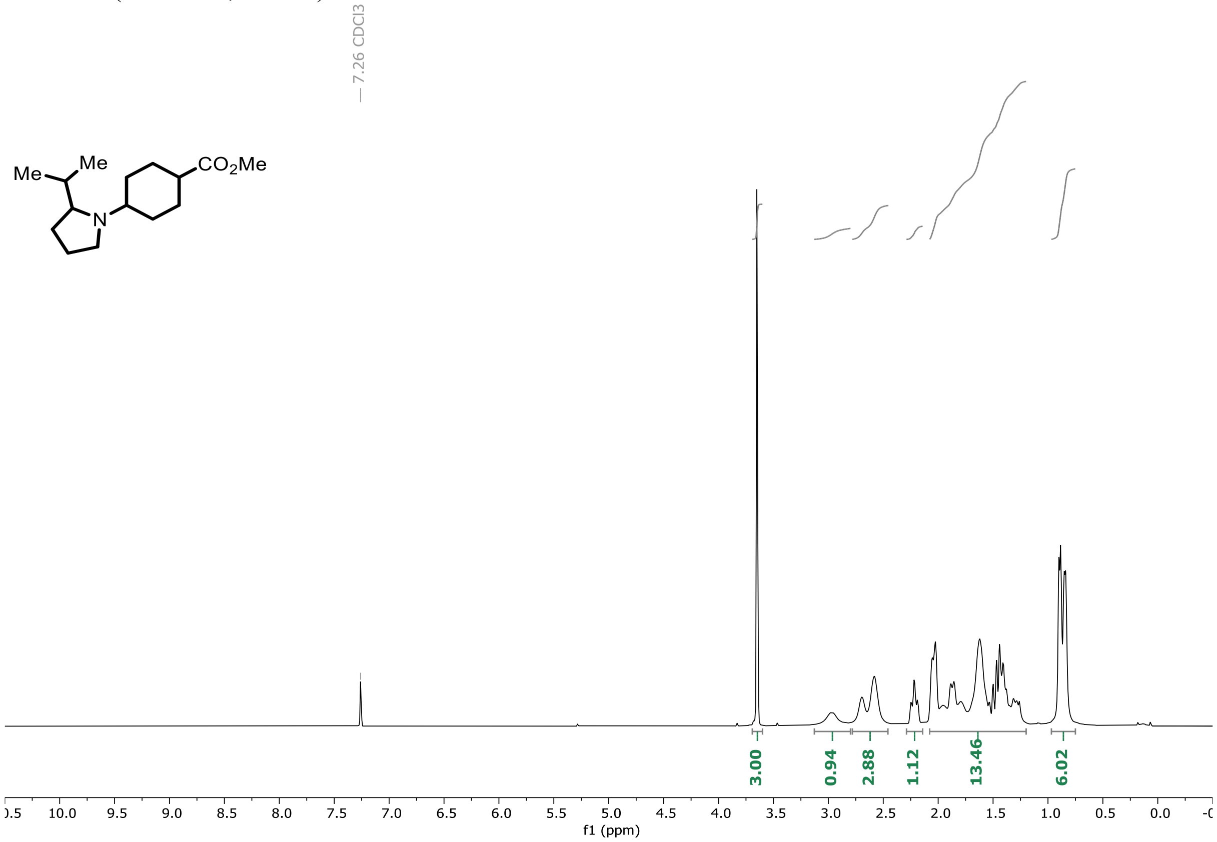

^{13}C NMR (101 MHz, $CDCl_3$) of **67**

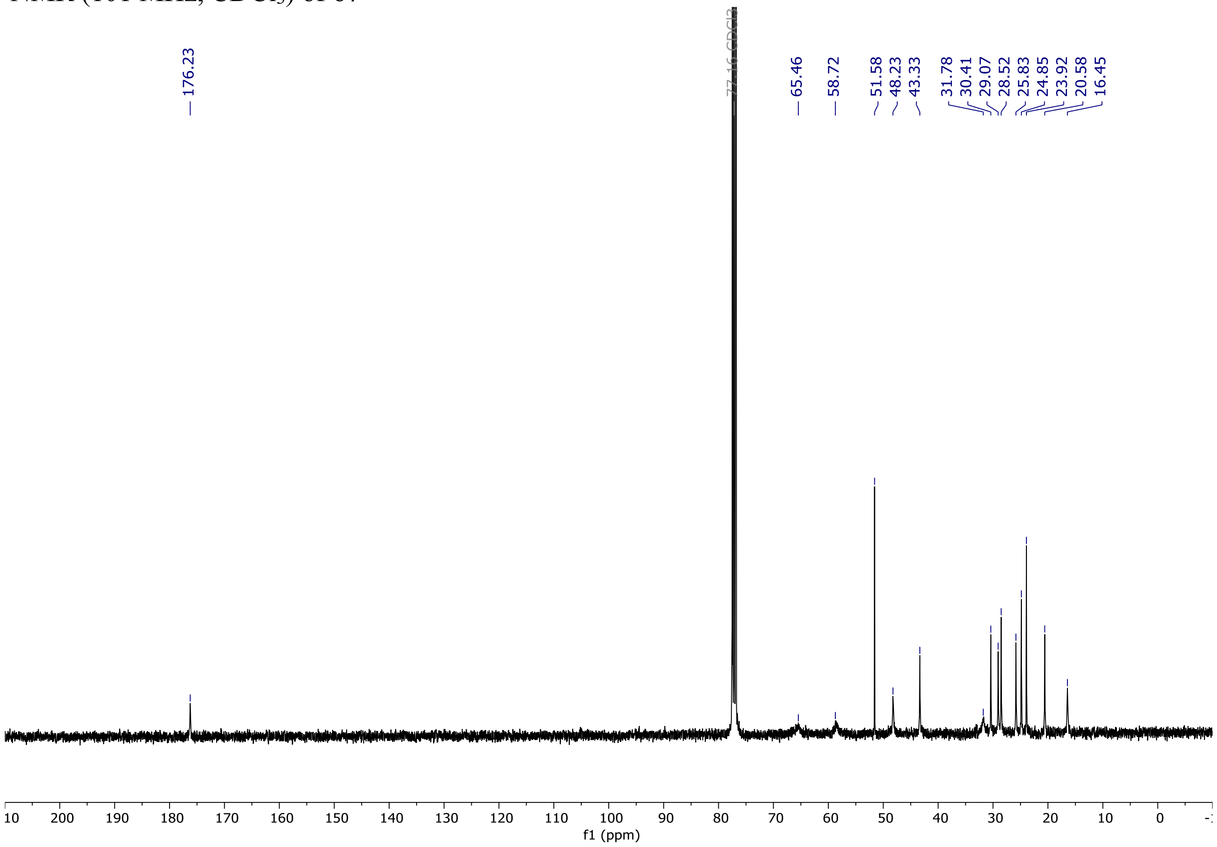

^{1}H NMR (400 MHz, MeOD) of **68**

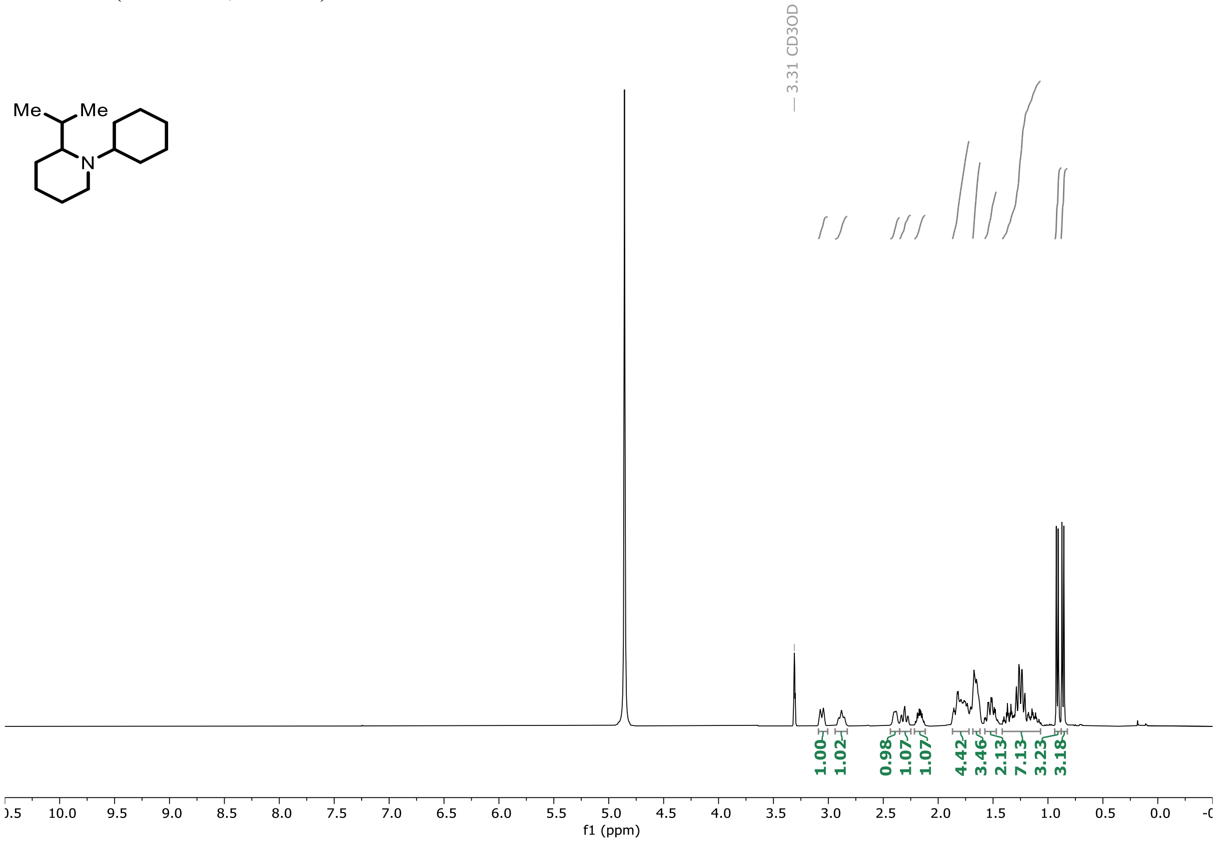

^{13}C NMR (101 MHz, MeOD) of **68**

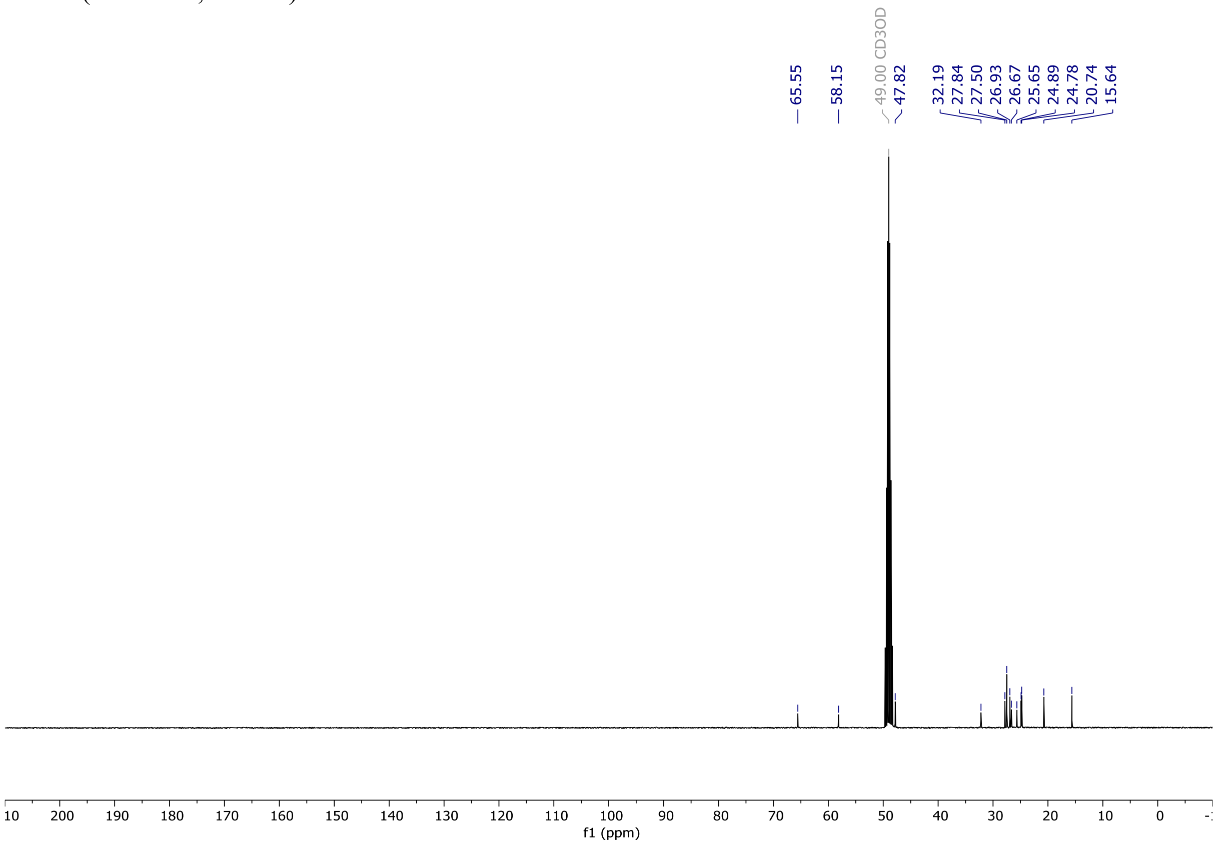

^{1}H NMR (400 MHz, $CDCl_3$) of **69**

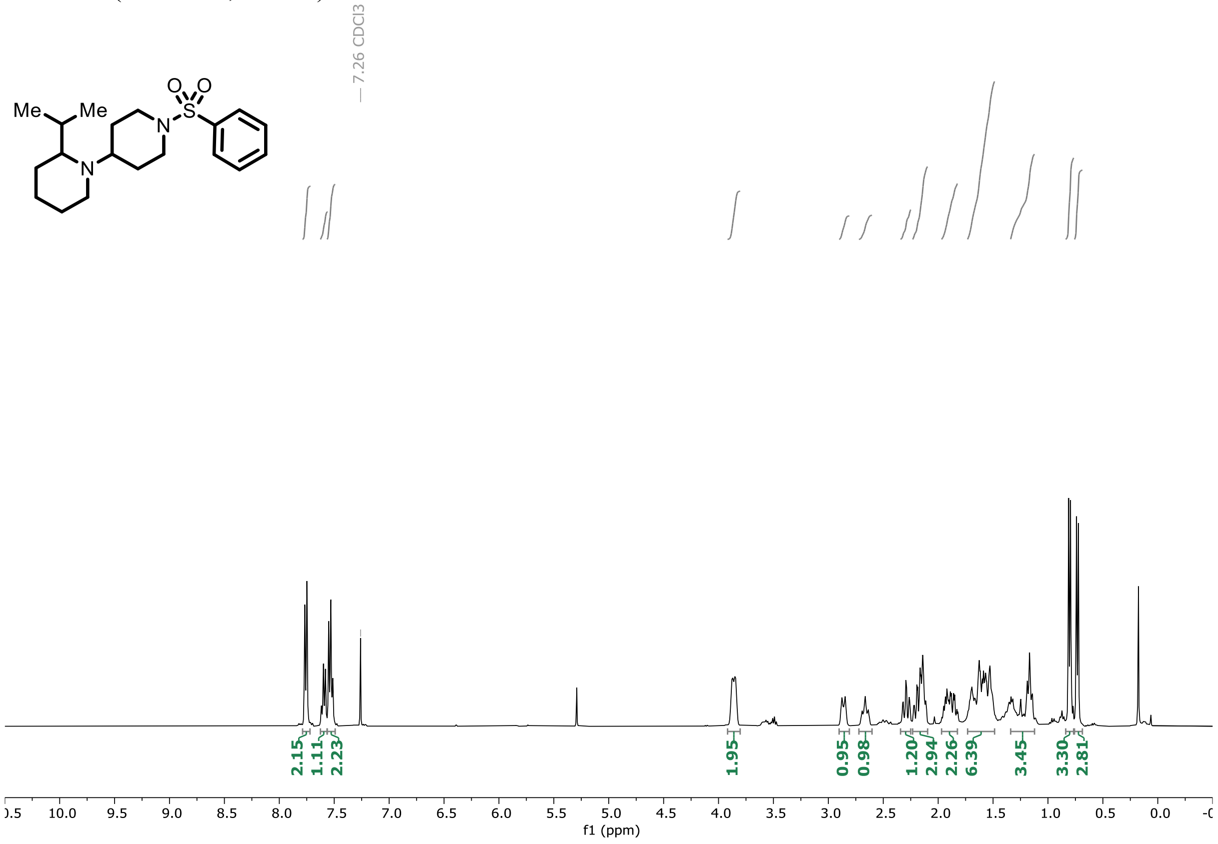

^{13}C NMR (101 MHz, $CDCl_3$) of **69**

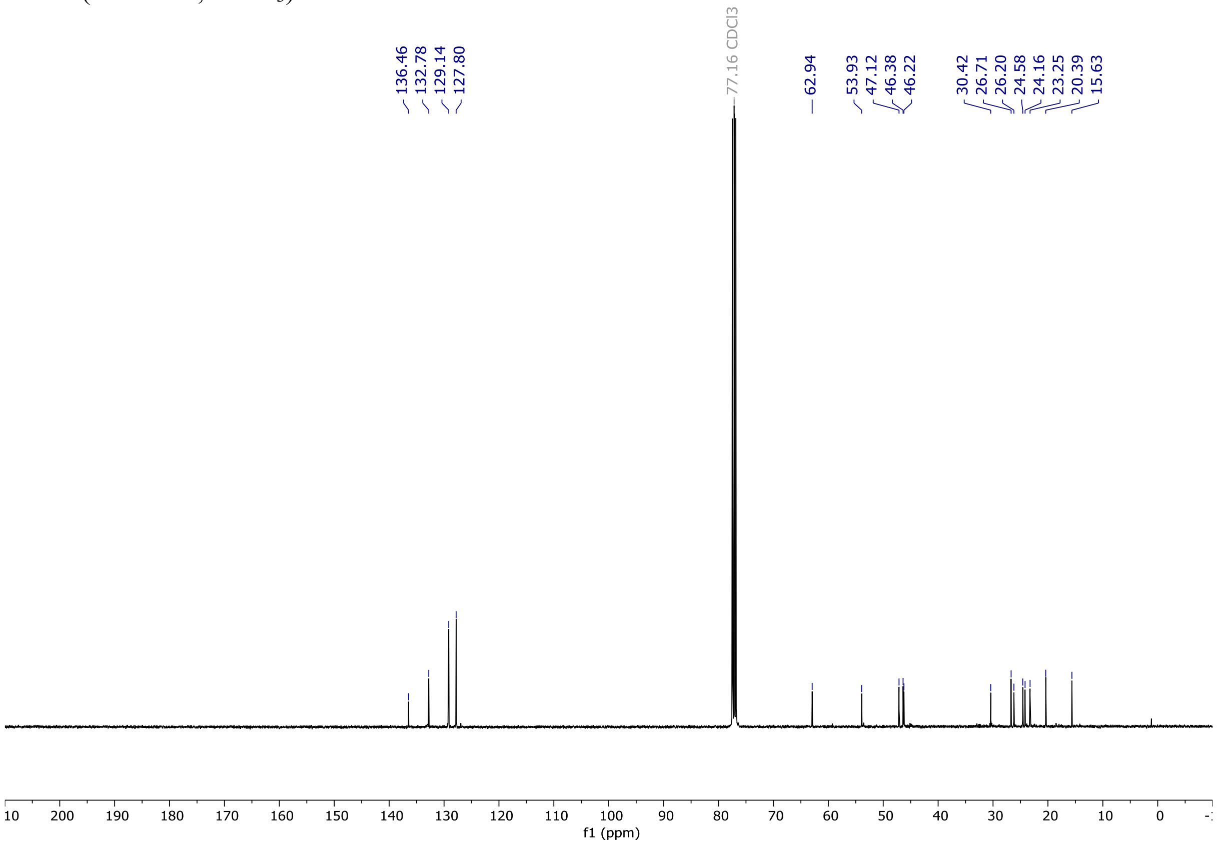

^{1}H NMR (400 MHz, MeOD) of **70**

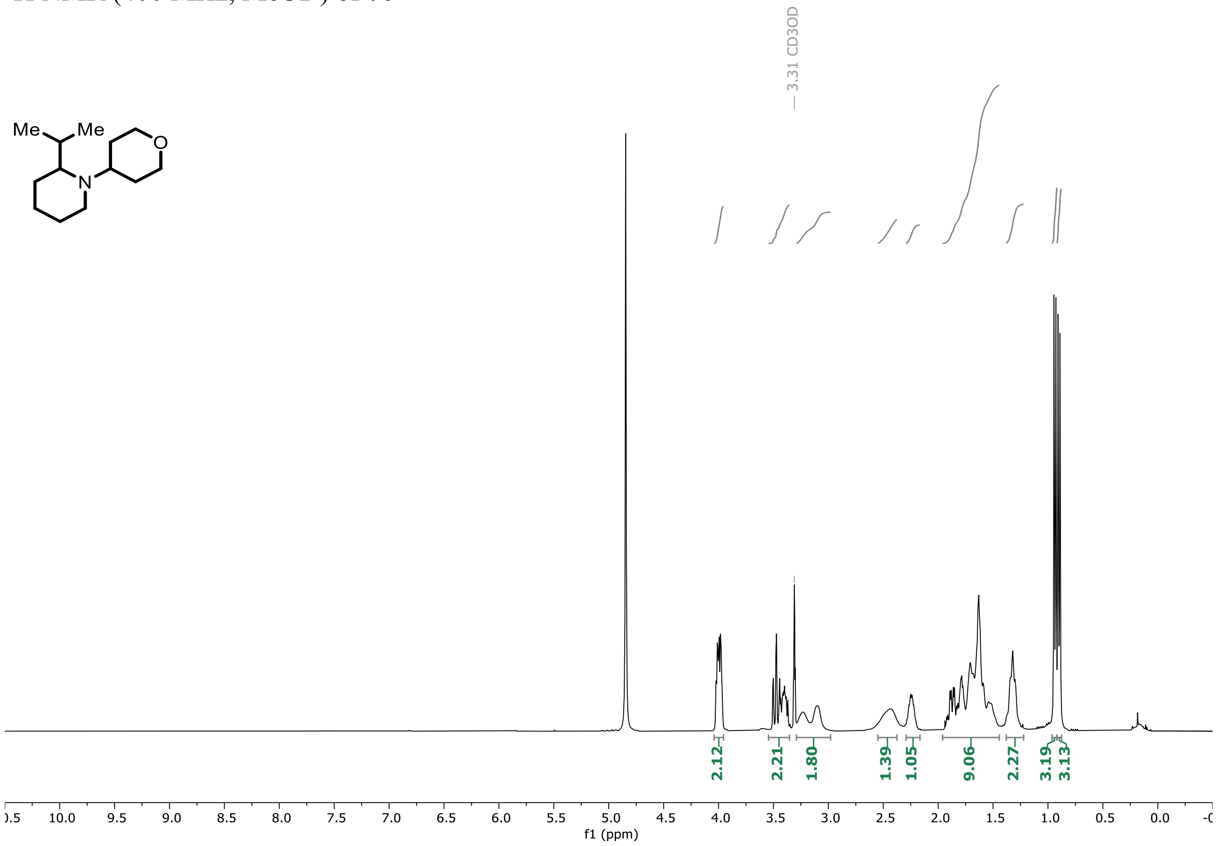

^{13}C NMR (101 MHz, MeOD) of **70**

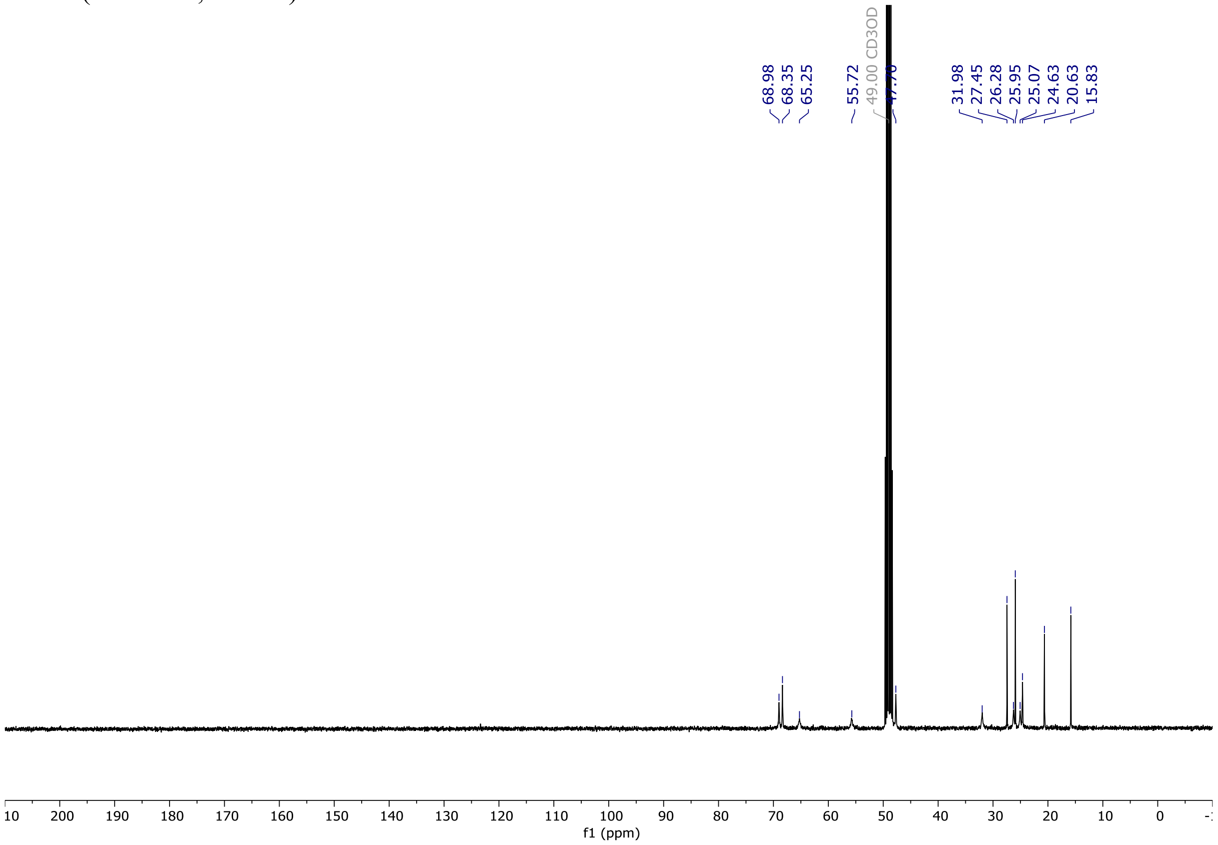

^{1}H NMR (400 MHz, MeOD) of **71**

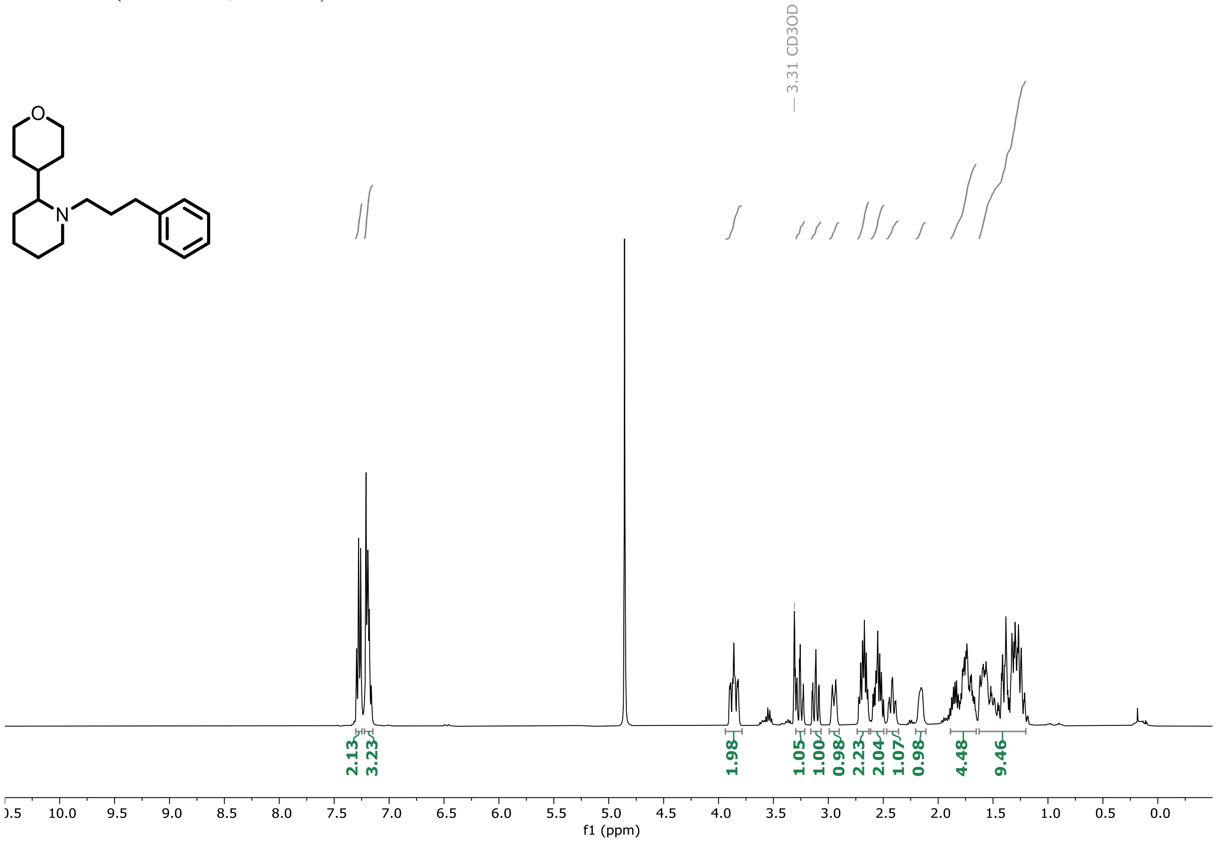

^{13}C NMR (101 MHz, MeOD) of **71**

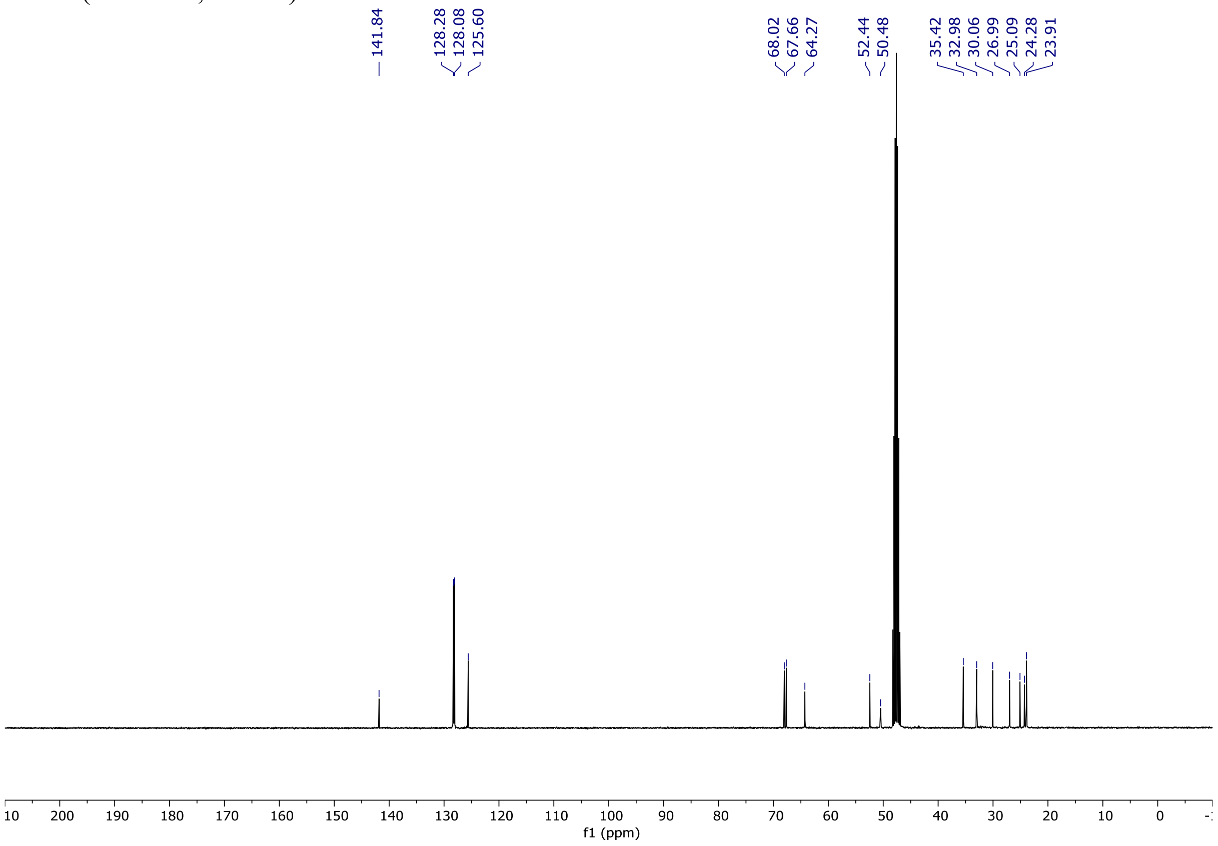

^{1}H NMR (300 MHz, $CDCl_3$) of **79**

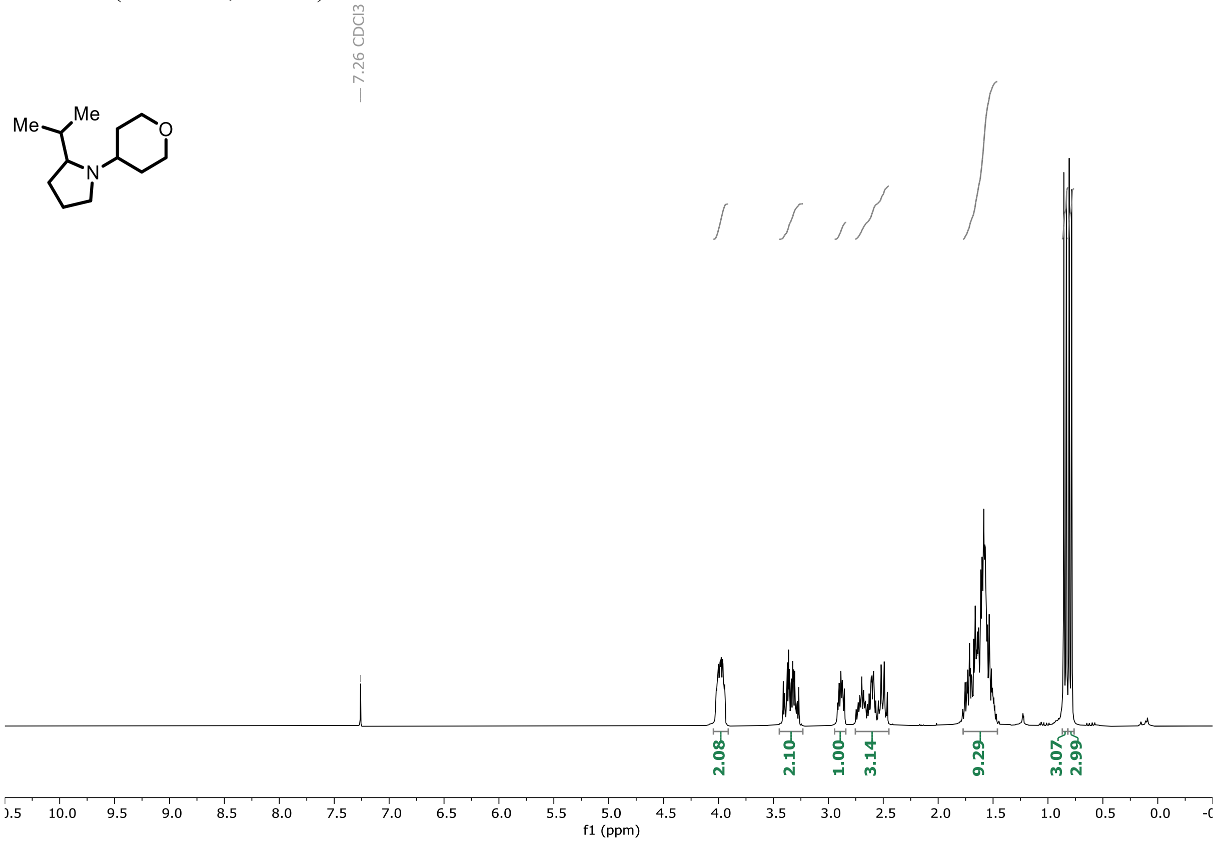

^{13}C NMR (75 MHz, $CDCl_3$) of **79**

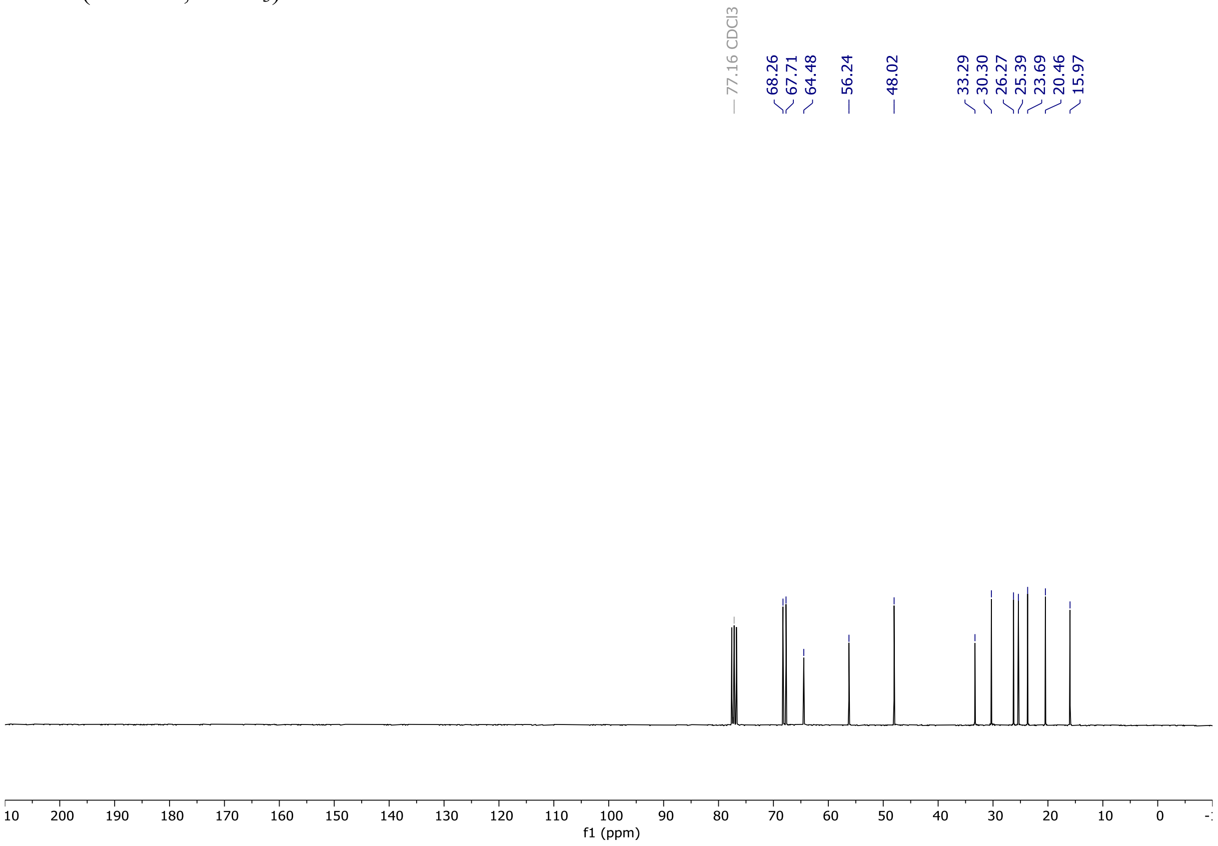

16. References

1. Zhang, Z., Górski, B., and Leonori, D. (2022). Merging Halogen-Atom Transfer (XAT) and Copper Catalysis for the Modular Suzuki–Miyaura-Type Cross-Coupling of Alkyl Iodides and Organoborons. J. Am. Chem. Soc. *144*, 1986-1992. 10.1021/jacs.1c12649.
2. MacMillan, D.S., Murray, J., Sneddon, H.F., Jamieson, C., and Watson, A.J.B. (2013). Evaluation of alternative solvents in common amide coupling reactions: replacement of dichloromethane and N,N-dimethylformamide. Green Chemistry *15*, 596-600. 10.1039/C2GC36900A.
3. Yue, H., Lu, F., Shen, C., and Quan, J.-M. (2015). Structure-based design of benzo[e]isoindole-1,3-dione derivatives as selective GSK-3β inhibitors to activate Wnt/β-catenin pathway. Bioorg. Chem. *61*, 21-27. https://doi.org/10.1016/j.bioorg.2015.05.009.
4. Heiss, C., Leroux, F., and Schlosser, M. (2005). Fluorine-Flanked Congested Sites: Minimal, Though Perceptible Buttressing Effects on the Proton Mobility of Arenes. Eur. J. Org. Chem. *2005*, 5242-5247. https://doi.org/10.1002/ejoc.200500552.
5. Masson, T.M., Zondag, S.D.A., Schuurmans, J.H.A., and Noël, T. (2024). Open-source 3D printed reactors for reproducible batch and continuous-flow photon-induced chemistry: design and characterization. Reaction Chemistry & Engineering *9*, 2218-2225. 10.1039/D4RE00081A.
6. Wan, T., Ciszewski, Ł.W., Ravelli, D., and Capaldo, L. (2024). Photoinduced Intermolecular Radical Hydroalkylation of Olefins via Ligated Boryl Radicals-Mediated Halogen Atom Transfer. Org. Lett. *26*, 5839-5843. 10.1021/acs.orglett.4c02034.
7. Cheng, Y., Mück-Lichtenfeld, C., and Studer, A. (2018). Metal-Free Radical Borylation of Alkyl and Aryl Iodides. Angew. Chem. Int. Ed. *57*, 16832-16836. https://doi.org/10.1002/anie.201810782.
8. Mistry, S., Kumar, R., Lister, A., and Gaunt, M.J. (2022). C(sp3)–C(sp3) coupling of non-activated alkyl-iodides with electron-deficient alkenes via visible-light/silane-mediated alkyl-radical formation. Chemical Science *13*, 13241-13247. 10.1039/D2SC03516B.
9. Wan, T., Wen, Z., Laudadio, G., Capaldo, L., Lammers, R., Rincón, J.A., García-Losada, P., Mateos, C., Frederick, M.O., Broersma, R., and Noël, T. (2022). Accelerated and Scalable C(sp3)–H Amination via Decatungstate Photocatalysis Using a Flow Photoreactor Equipped with High-Intensity LEDs. ACS Central Science *8*, 51-56. 10.1021/acscentsci.1c01109.
10. Xiao, K.-J., Wang, A.-E., and Huang, P.-Q. (2012). Direct Transformation of Secondary Amides into Secondary Amines: Triflic Anhydride Activated Reductive Alkylation. Angew. Chem. Int. Ed. *51*, 8314-8317. https://doi.org/10.1002/anie.201204098.
